# The causal effect between psoriasis and male reproductive health, and the mediating effect of serum inflammatory factors and metabolites

**DOI:** 10.1097/MD.0000000000044758

**Published:** 2025-09-26

**Authors:** Sicheng Ma, Ruimin Ma, Yinuo Zhang, Xiaohui Hao, Wenbang Liu, Wenlin Yu, Jing Hu, Zixue Sun, Chenming Zhang

**Affiliations:** a The Second Clinical Medical School, Henan University of Chinese Medicine, Zhengzhou, China; b Traditional Chinese Medicine (Zhong Jing) School, Henan University of Chinese Medicine, Zhengzhou, China; c Orthopedics & Traumatology College, Henan University of Chinese Medicine, Zhengzhou, China; d College of Acupuncture and Massage, Henan University of Traditional Chinese Medicine, Zhengzhou, China.

**Keywords:** male reproductive health, Mendelian randomization analysis, metabolites, psoriasis, serum inflammatory factors

## Abstract

The relationship between psoriasis and male reproductive health has sparked extensive research. But the causal relationship and direction remain unknown. To examine the causal association between psoriasis and male reproductive health, and to investigate the role of serum inflammatory factor and metabolites as mediators. A 2-step Mendelian randomization analysis was utilized. Inverse variance weighted method to explore the causal effects. The Cochran *Q* test was employed to assess the heterogeneity. The Mendelian randomization-Egger and Mendelian randomization-PRESSO method was used to explore the genetic pleiotropy. The mediating effects can be verified using a stepwise testing approach. Psoriasis vulgaris (*P* = .005, odds ratios [OR] = 1.137), arthropathic psoriasis (*P* = .044, OR = 1.112), guttate psoriasis (*P* = .026, OR = 1.041), and other and unspecified psoriasis (*P* = .030, OR = 1.085) were found to be risk factors for abnormal spermatozoa. X-09026 (49.219%) and macrophage migration inhibitory factor (18.000%) serve as mediators in the regulation of the causal association between Psoriasis vulgaris and abnormal spermatozoa. Methylcysteine (35.849%) also acts as a mediator in the regulation of the causal relationship between arthropathic psoriasis and abnormal spermatozoa. Psoriasis may cause sperm abnormality through metabolites such as X-09026, macrophage migration inhibitory factor, and methylcysteine.

## 1. Introduction

Psoriasis, a prevalent chronic autoimmune disease, is distinguished by aberrant skin cell growth, differentiation, and immune responses.^[1]^ Its worldwide prevalence stands at approximately 2% to 3%, affecting both sexes equally.^[2]^ While typically manifesting between the ages of 20 to 40, psoriasis can also emerge in children and older individuals.^[3]^ The etiology of psoriasis is intricate and encompasses various immune pathways, such as T-cell-mediated immune responses, keratinocyte hyperproliferation, and inflammation.^[4]^ A critical limitation of current genetic studies lies in the predominant reliance on genome-wide association study (GWAS) data derived from European populations. While these studies have identified key susceptibility loci (e.g., ERAP1, TNIP1-ANXA6), recent research highlights substantial genetic heterogeneity between European and East Asian populations. For example, 6 novel loci (e.g., ZNF816A, GJB2) were uniquely associated with psoriasis in Chinese cohorts, underscoring the need for caution when generalizing findings across ancestries.

Emerging evidence suggests systemic inflammation in psoriasis may extend beyond the skin, potentially impacting distant organs, including the male reproductive system.^[5–8]^ The correlation between psoriasis and male reproductive health is a multifaceted and intricate matter.^[9]^ The persistent inflammatory reaction associated with psoriasis can potentially exert a direct or indirect influence on male reproductive function.^[10]^ Inflammation has the potential to impede vascular endothelial function, disrupt blood flow, and consequently exert an adverse influence on erectile function.^[11]^ Furthermore, specific medications employed in psoriasis treatment, such as methotrexate, possess reproductive toxicity and may potentially impede sperm production.^[12]^ Several investigations have proposed a potential connection between inflammatory responses and sexual dysfunction.^[13]^ Numerous investigations have demonstrated that individuals with psoriasis frequently encounter sexual dysfunction and erectile dysfunction.^[14,15]^ The prevalent challenges encountered by psoriasis patients, such as psychological stress,^[16]^ depression,^[17]^ and anxiety,^[18]^ may detrimentally affect male erectile function. In contrast, the pharmacological interventions employed in the management of psoriasis and chronic inflammation may bear consequences for male reproductive well-being.^[19,20]^ Prolonged administration of immunosuppressants and corticosteroids, frequently necessitated by psoriasis patients, can detrimentally impact testicular functionality, thereby exerting a direct or indirect influence on male reproductive capabilities.^[21]^ However, the current understanding of the causal association between psoriasis and male reproductive and sexual well-being remains limited. Importantly, the generalizability of these findings may be constrained by the European-centric genetic data, as population-specific differences in disease mechanisms (e.g., immune pathway activation, metabolic profiles) could modulate both psoriasis severity and its systemic effects.

Observational studies are deemed less credible as a result of the absence of random assignment, whereas randomized controlled trials are also subject to limitations due to diverse clinical constraints. Consequently, Mendelian randomization (MR) has emerged as an analytical approach employing instrumental variables (IV) to evaluate causal associations between exposure and outcomes.^[22]^ This method effectively mitigates the impact of potential confounders and eradicates the interference of reverse causality, thereby enhancing the dependability and accuracy of the findings. Nevertheless, the MR approach in this study relies exclusively on European GWAS data, which may limit the extrapolation of results to non-European populations. For instance, genetic variants influencing immune responses (e.g., *IL23R*, *HLA-C*) exhibit allele frequency disparities between European and Asian populations, potentially altering their causal effects on reproductive outcomes. This study will utilize the MR approach to examine the causal association between various forms of psoriasis and male reproductive health, as well as to investigate potential mediators in serum inflammatory cytokines and metabolites.

## 2. Materials and methods

### 2.1. Overall research design

A 2-step MR analysis was utilized to explore the potential involvement of serum inflammatory factors and metabolites in mediating the impact of psoriasis on male reproductive health. The initial phase of our 2-step MR study aims to assess the causal effect of psoriasis on male reproductive health through the utilization of a 2-sample MR approach. Subsequently, the second step of our study was to explore the possible mediating effects of serum inflammatory factors and metabolites on the causal effect of psoriasis on male reproductive health.

### 2.2. Data source

#### 2.2.1. Serum inflammatory factors and metabolites

The genetic data concerning these metabolites were obtained from the metabolomics GWAS server.^[23]^ The single nucleotide polymorphisms (SNPs) linked to inflammatory factors were acquired from a comprehensive study involving 8293 individuals, encompassing an analysis of 41 cytokines and growth factors.^[24]^

#### 2.2.2. Distinct types of psoriasis

The GWAS summary statistics for 5 distinct types of psoriasis and 5 male reproductive system diseases were obtained from the published data of the Finn Gen Consortium R9. The investigation encompassed psoriasis, psoriasis vulgaris, guttate psoriasis, arthropathic psoriasis, and other and unspecified psoriasis. The psoriasis GWAS data used for validation come from the UK Biobank database.

#### 2.2.3. Male reproductive system diseases

The investigation encompassed male infertility, abnormal spermatozoa, sexual dysfunction, erectile dysfunction, and testicular dysfunction. All the data were obtained from the published data of the Finn Gen Consortium R9.

*Abnormal spermatozoa*: Compliance with WHO 5th edition standards for semen analysis, meeting at least one of the following: sperm concentration < 15 × 10⁶/mL; proportion of forward-moving spermatozoa (PR) < 32%; normal morphology rate < 4%.

*Sexual dysfunction*: Assessment based on the International Index of Erectile Function-5 or the Men’s Sexual Health Scale, including decreased libido, erectile dysfunction, or ejaculatory dysfunction for ≥6 months.

*Erectile dysfunction*: International Index of Erectile Function-5 score ≤ 21 and secondary etiologies such as diabetes mellitus, cardiovascular disease, or neurological injury were excluded.

*Testicular dysfunction*: Serum total testosterone level < 300 ng/dL (confirmed by repeat testing) with at least one of the following: testicular volume < 15 mL (measured by ultrasound); follicle stimulating hormone (FSH) > 12 IU/L; abnormal spermatogenesis (see K11_ABNSPERM criteria).

### 2.3. MR study

#### 2.3.1. Selection of IVs

In the 2-sample MR analysis, the inclusion of 5 distinct types of psoriasis tools was determined by their statistically significant correlation at the genome-wide level (*P* < 5 × 10^−8^). These SNPs were selected based on a linkage disequilibrium parameter (*r*^2^) of <0.001 and a genetic distance of 10,000 kb. In the investigation of the causal association between serum metabolites and abnormal spermatozoa, supplementary IVs were sought by establishing screening criteria of *P* < 1 × 10^−5^, *r*^2^ < 0.1, and kb = 500.^[25]^ During the causal effect analysis of other and unspecified psoriasis on abnormal spermatozoa and the causal effect analysis of male reproductive health on psoriasis, to obtain more IVs for analysis, we relaxed the screening criteria for IVs: *P* < 5 × 10^−6^, *r*^2^ < 0.01, and kb = 5000.

#### 2.3.2. Causal effect analysis

The present study utilized the inverse variance weighted analysis (IVW), MR Egger, weighted median, simple mode, and weighted mode method to investigate the potential causal impact of psoriasis on male reproductive function and sexual health and the mediating effect of serum inflammatory factors and metabolites.^[26]^

Furthermore, the findings were corroborated by analyzing psoriasis-related data obtained from the UK Biobank database. The results are shown using odds ratios and 95% confidence intervals. *P* < .05 for the IVW approach was considered suggestive of a potential association.

#### 2.3.3. Mediating effect analysis

Mediation analysis was performed through a 3-step causal decomposition: estimating the exposure-to-mediator effect (ɑ) via 2-sample MR, calculating the mediator-to-outcome effect (β) with inverse-variance weighted MR, and deriving the total exposure-to-outcome effect (τ) from primary MR analysis. The mediated effect was quantified as the product term ɑβ, with direct effect computed as τ − ɑβ, and mediation proportion expressed as ɑβ/τ. To address statistical uncertainty, we implemented a nonparametric bootstrap procedure (10,000 iterations) that generated bias-corrected 95% confidence intervals for all mediation parameters, while simultaneously evaluating significance through the proportion of bootstrap samples with concordant effect directions (ɑβ > 0). This dual approach (combining product-of-coefficients estimation with stochastic resampling) aligns with contemporary causal mediation frameworks by rigorously quantifying both effect magnitudes and their empirical distributions.^[27]^

#### 2.3.4. Sensitivity analysis

The Cochran *Q* test is utilized to assess the heterogeneity among individual IVs, with larger disparities indicating higher levels of heterogeneity. To investigate the genetic pleiotropy between SNPs and outcomes, MR-Egger regression and MR-PRESSO methods were employed.^[28,29]^

## 3. Results

### 3.1. Selection of IVs

Based on the screening criteria mentioned in the article, a total of 11,109 IVs were included in this study, as detailed in Tables S1–S4, Supplemental Digital Content, https://links.lww.com/MD/Q116, with *F* values ranging from 11.160 to 3089.498. A total of 792 confounding factors were excluded, as shown in Table S5, Supplemental Digital Content, https://links.lww.com/MD/Q116.

### 3.2. Causal effect of psoriasis on male reproductive health

The findings from the MR analysis indicate that there is a discernible causal association between types of psoriasis and male reproductive system diseases (Table 1). Psoriasis was found to be a risk factor for abnormal spermatozoa and testicular dysfunction. Further research found that psoriasis vulgaris, arthropathic psoriasis, guttate psoriasis and other and unspecified psoriasis were also risk factors for abnormal spermatozoa. Further validation analysis revealed that psoriasis is a risk factor for abnormal spermatozoa but not testicular dysfunction (Table S6, Supplemental Digital Content, https://links.lww.com/MD/Q116). For 2-sample MR analysis results for 5 distinct types of psoriasis and 5 male reproductive system diseases, please refer to Table S6, Supplemental Digital Content, https://links.lww.com/MD/Q116.

**Table 1:** Two-sample MR analysis results for the effect of psoriasis on male reproductive health (OR: odds ratio of coronary artery disease per SD increase in proteomic analyte levels).

Exposure	Outcome	Method	Nsnp	Beta	*P*_IVW_	OR (95% CI)
Psoriasis	Abnormal spermatozoa	Inverse variance weighted	16	0.133	.005	1.143 (1.041–1.254)
		MR Egger	16	0.116	.157	1.223 (0.966–1.305)
		Weighted median	16	0.146	.023	1.157 (1.020–1.311)
		Simple mode	16	0.211	.031	1.235 (1.038–1.468)
		Weighted mode	16	0.142	.039	1.152 (1.019–1.303)
Psoriasis	Testicular dysfunction	Inverse variance weighted	16	0.203	.015	1.225 (1.041–1.441)
		MR Egger	16	0.061	.655	1.063 (0.818–1.381)
		Weighted median	16	0.146	.162	1.157 (0.943–1.419)
		Simple mode	16	0.142	.391	1.153 (0.841–1.581)
		Weighted mode	16	0.153	.150	1.165 (0.956–1.420)
Psoriasis vulgaris	Abnormal spermatozoa	Inverse variance weighted	10	0.128	.005	1.137 (1.040–1.243)
		MR Egger	10	0.04	.632	1.041 (0.888–1.221)
		Weighted median	10	0.071	.224	1.074 (0.957–1.205)
		Simple mode	10	0.061	.572	1.063 (0.866–1.306)
		Weighted mode	10	0.072	.24	1.075 (0.960–1.203)
Arthropathic psoriasis	Abnormal spermatozoa	Inverse variance weighted	4	0.106	.044	1.112 (1.003–1.234)
		MR Egger	4	−0.009	.96	0.991 (0.723–1.357)
		Weighted median	4	0.092	.122	1.096 (0.976–1.232)
		Simple mode	4	0.043	.641	1.044 (0.886–1.231)
		Weighted mode	4	0.089	.269	1.093 (0.961–1.243)
Guttate psoriasis	Abnormal spermatozoa	Inverse variance weighted	3	0.040	.026	1.041 (1.005–1.079)
		MR Egger	3	0.015	.772	1.016 (0.937–1.101)
		Weighted median	3	0.035	.088	1.036 (0.995–1.078)
		Simple mode	3	0.034	.305	1.035 (0.985–1.087)
		Weighted mode	3	0.034	.294	1.035 (0.987–1.086)
Other and unspecified psoriasis	Abnormal spermatozoa	Inverse variance weighted	3	0.081	.030	1.085 (1.008–1.168)
		MR Egger	3	0.051	.55	1.052 (0.936–1.183)
		Weighted median	3	0.076	.066	1.079 (0.995–1.170)
		Simple mode	3	0.078	.254	1.081 (0.981–1.191)
		Weighted mode	3	0.075	.209	1.078 (0.995–1.169)

Beta = the effect size of the effect allele, CI = confidence interval, IVW = inverse variance weighted analysis, MR = Mendelian randomization, nsnp = number of single nucleotide polymorphisms, OR = odds ratio.

### 3.3. Causal effect of male reproductive health on psoriasis

The findings of reverse MR analysis (Table 2) suggest that sexual dysfunction may have a protective effect against psoriasis vulgaris. Erectile dysfunction was identified as a potential protective factor for psoriasis. However, it is important to note that the validation analysis did not provide support for the aforementioned conclusions. Consequently, further verification is required to establish the validity of the reverse MR conclusion (Table S7, Supplemental Digital Content, https://links.lww.com/MD/Q116). For the 2-sample MR analysis results for the causal effect of male reproductive health on psoriasis, please refer to Table S7, Supplemental Digital Content, https://links.lww.com/MD/Q116.

**Table 2:** Two-sample MR analysis results for the effect of male reproductive health on psoriasis.

Exposure	Outcome	Method	Nsnp	Beta	*P*_IVW_	OR (95% CI)
Sexual dysfunction	Psoriasis vulgaris	Inverse variance weighted	4	−0.051	.035	0.950 (0.906–0.997)
		MR Egger	4	−0.024	.71	0.977 (0.876–1.089)
		Weighted median	4	−0.054	.063	0.947 (0.895–1.003)
		Simple mode	4	−0.071	.164	0.931 (0.863–1.005)
		Weighted mode	4	−0.072	.15	0.931 (0.865–1.001)
Erectile dysfunction	Psoriasis	Inverse variance weighted	13	−0.072	.016	0.931 (0.878–0.987)
		Weighted median	13	−0.091	.019	0.913 (0.846–0.985)
		MR Egger	13	−0.086	.281	0.917 (0.791–1.065)
		Simple mode	13	−0.126	.115	0.881 (0.762–1.019)
		Weighted mode	13	−0.122	.118	0.885 (0.768–1.020)

Beta = the effect size of the effect allele, CI = confidence interval, IVW = inverse variance weighted analysis, MR = Mendelian randomization, nsnp = number of single nucleotide polymorphisms, OR = odds ratio.

### 3.4. Causal effect of serum inflammatory factors and metabolites on abnormal spermatozoa

As shown in Table 3, 16 serum metabolites and 1 serum inflammatory factor were found to be statistically significant in the MR analysis with abnormal spermatozoa. Among these metabolites, gamma-glutamylglutamine, glucose, X-09026, dodecanedioate, butyrylcarnitine, X-11315, methylcysteine, 2-linoleoylglycerophosphocholine, and X-14745 were found to be risk factors for abnormal spermatozoa. Aspartate, 2-hydroxyisobutyrate, cysteine, caprylate, X-11483, 1,3,7-trimethylurate, X-12844, and macrophage migration inhibitory factor (MMIF) levels were found to be protective against abnormal spermatozoa. The findings of the examination of a total of 486 serum metabolites and 41 inflammatory factors are provided in Tables S8 and S9, Supplemental Digital Content, https://links.lww.com/MD/Q116, respectively.

**Table 3:** The causal impact of serum inflammatory factors and metabolites on abnormal spermatozoa.

Exposure	Method	Nsnp	Beta	*P*_IVW_	OR (95% CI)
Gamma-glutamylglutamine	Inverse variance weighted	23	2.186	.006	8.903 (1.885–42.041)
	MR Egger	23	1.361	.351	3.901 (0.239–63.799)
	Weighted median	23	1.443	.246	4.234 (0.370–48.512)
	Simple mode	23	−0.309	.887	0.734 (0.011–49.594)
	Weighted mode	23	1.223	.322	3.397 (0.319–36.149)
Aspartate	Inverse variance weighted	4	−2.567	.05	0.077 (0.006–0.999)
	MR Egger	4	1.91	.61	6.750 (0.013–0.056)
	Weighted median	4	−1.83	.245	0.160 (0.007–5.098)
	Simple mode	4	−1.672	.442	0.188 (0.005–7.666)
	Weighted mode	4	−1.696	.366	0.183 (0.008–4.187)
Glucose	Inverse variance weighted	38	2.192	.020	8.957 (1.418–56.579)
	MR Egger	38	1.918	.334	6.809 (0.146–317.475)
	Weighted median	38	1.909	.195	6.744 (0.377–120.790)
	Simple mode	38	1.129	.66	3.093 (0.021–452.310)
	Weighted mode	38	1.9	.352	6.683 (0.129–347.264)
2-Hydroxyisobutyrate	Inverse variance weighted	20	−1.606	.046	0.201 (0.042–0.969)
	MR Egger	20	−2.372	.325	0.093 (0.001–9.248)
	Weighted median	20	−0.314	.793	0.730 (0.070–7.610)
	Simple mode	20	1.002	.661	2.723 (0.033–224.500)
	Weighted mode	20	0.636	.79	1.889 (0.019–191.587)
X-09026	Inverse variance weighted	15	2.621	.018	13.756 (1.574–120.207)
	MR Egger	15	5.095	.289	163.140 (0.019–370,046.620)
	Weighted median	15	2.64	.076	14.018 (0.762–257.779)
	Simple mode	15	2.634	.291	13.932 (0.127–1532.640)
	Weighted mode	15	2.378	.227	10.779 (0.270–429.690)
Cysteine	Inverse variance weighted	19	−1.963	.003	0.140 (0.038–0.514)
	MR Egger	19	−1.595	.148	0.203 (0.026–1.599)
	Weighted median	19	−1.895	.064	0.150 (0.020–1.114)
	Simple mode	19	−3.385	.06	0.034 (0.001–0.924)
	Weighted mode	19	−2.001	.07	0.135 (0.018–1.034)
Dodecanedioate	Inverse variance weighted	7	1.871	.006	6.493 (1.730–24.372)
	MR Egger	7	0.795	.766	2.214 (0.016–315.234)
	Weighted median	7	1.995	.027	7.354 (1.260–42.903)
	Simple mode	7	2.564	.076	12.986 (1.241–135.855)
	Weighted mode	7	2.481	.078	11.959 (1.201–119.096)
Butyrylcarnitine	Inverse variance weighted	52	0.344	.033	1.411 (1.028–1.937)
	MR Egger	52	0.621	.026	1.860 (1.093–3.166)
	Weighted median	52	0.483	.038	1.622 (0.026–2.562)
	Simple mode	52	0.413	.25	1.512 (0.754–3.033)
	Weighted mode	52	0.413	.063	1.512 (0.987–72.315)
Caprylate	Inverse variance weighted	46	−1.227	.047	0.293 (0.087–0.985)
	MR Egger	46	−2.835	.016	0.059 (0.006–0.542)
	Weighted median	46	−1.508	.161	0.221 (0.027–1.822)
	Simple mode	46	−1.441	.448	0.237 (0.006–9.501)
	Weighted mode	46	−1.786	.079	0.168 (0.024–1.172)
X-11315	Inverse variance weighted	29	0.948	.032	2.582 (1.086–6.134)
	MR Egger	29	0.653	.357	1.921 (0.491–7.524)
	Weighted median	29	0.8	.192	2.225 (0.669–7.405)
	Simple mode	29	0.169	.852	1.184 (0.203–6.920)
	Weighted mode	29	0.733	.253	2.082 (0.607–7.137)
X-11483	Inverse variance weighted	12	−0.481	.039	0.618 (0.392–0.975)
	MR Egger	12	−0.671	.07	0.511 (0.267–0.977)
	Weighted median	12	−0.52	.114	0.595 (0.312–1.133)
	Simple mode	12	−0.465	.355	0.628 (0.244–1.614)
	Weighted mode	12	−0.526	.129	0.591 (0.316–1.107)
Methylcysteine	Inverse variance weighted	13	1.539	.011	4.658 (1.417–15.309)
	MR Egger	13	4.702	.071	110.128 (1.102–11,006.567)
	Weighted median	13	1.681	.019	5.372 (1.312–21.996)
	Simple mode	13	1.296	.328	3.654 (0.303–44.022)
	Weighted mode	13	1.795	.126	6.018 (0.710–51.016)
1,3,7-Trimethylurate	Inverse variance weighted	10	−0.528	.014	0.590 (0.387–0.898)
	MR Egger	10	0.177	.795	1.194 (0.328–4.348)
	Weighted median	10	−0.297	.31	0.743 (0.419–1.318)
	Simple mode	10	−0.108	.843	0.898 (0.320–2.521)
	Weighted mode	10	−0.057	.891	0.945 (0.425–2.098)
X-12844	Inverse variance weighted	17	−1.528	.025	0.217 (0.057–0.826)
	MR Egger	17	−0.516	.827	0.597 (0.006–55.879)
	Weighted median	17	−1.83	.048	0.160 (0.026–0.981)
	Simple mode	17	−2.037	.205	0.130 (0.006–2.678)
	Weighted mode	17	−2.076	.196	0.125 (0.006–2.565)
2-Linoleoylglycerophosphocholine	Inverse variance weighted	22	2.051	.003	7.776 (2.022–29.904)
	MR Egger	22	1.345	.569	3.839 (0.041–363.628)
	Weighted median	22	1.517	.12	4.560 (0.674–125.846)
	Simple mode	22	1.58	.346	4.855 (0.195–120.803)
	Weighted mode	22	1.368	.353	3.927 (0.233–66.135)
X-14745	Inverse variance weighted	12	1.918	.030	6.809 (1.209–38.343)
	MR Egger	12	−5.766	.265	0.003 (0.000–4.977)
	Weighted median	12	1.491	.194	4.439 (0.468–42.098)
	Simple mode	12	1.364	.505	3.913 (0.081–189.174)
	Weighted mode	12	1.44	.46	4.222 (0.106–168.448)
MMIF (macrophage migration inhibitory factor)	Inverse variance weighted	6	−0.320	.046	0.726 (0.530–0.995)
	MR Egger	6	−0.101	.532	0.905 (0.400–2.048)
	Weighted median	6	−0.281	.138	0.756 (0.530–1.078)
	Simple mode	6	−0.151	.624	0.861 (0.480–1.543)
	Weighted mode	6	−0.183	.455	0.835 (0.492–1.416)

Beta = the effect size of the effect allele, CI = confidence interval, IVW = inverse variance weighted analysis, MR = Mendelian randomization, nsnp = number of single nucleotide polymorphisms, OR = odds ratio.

### 3.5. Causal effect of different kinds of psoriasis on abnormal spermatozoa-related serum

Our research findings indicate the presence of causal associations between 3 specific forms of psoriasis and serum metabolites associated with abnormal spermatozoa (Table 4). More specifically, a significant causal link was observed between psoriasis vulgaris and X-09026, arthropathic psoriasis, and X-11786-methylcysteine, and other and unspecified psoriasis with both aspartate and cysteine.

**Table 4:** The causal effect of different kinds of psoriasis on abnormal spermatozoa-related serum inflammatory factors and metabolites.

Exposure	Outcome	Method	Nsnp	Beta	*P*_IVW_	OR (95% CI)
Psoriasis vulgaris	X-09026	Inverse variance weighted	4	0.024	.002	1.025 (1.009–1.040)
		MR Egger	4	−0.014	.63	0.986 (0.940–1.035)
		Weighted median	4	0.019	.046	1.019 (1.000–1.038)
		Simple mode	4	0.023	.213	1.023 (0.994–1.053)
		Weighted mode	4	0.015	.229	1.015 (0.995–1.035)
Psoriasis vulgaris	MMIF (macrophage migration inhibitory factor)	Inverse variance weighted	8	−0.072	.020	0.931 (0.876–0.989)
		MR Egger	8	−0.072	.278	0.930 (0.826–1.048)
		Weighted median	8	−0.063	.079	0.939 (0.875–1.007)
		Simple mode	8	−0.182	.064	0.833 (0.708–0.980)
		Weighted mode	8	−0.062	.166	0.940 (0.868–1.017)
Arthropathic psoriasis	Methylcysteine	Inverse variance weighted	2	0.025	.032	1.026 (1.002–1.049)
		MR Egger	2	0.021	.165	1.021 (0.992–1.051)
		Weighted median	2	0.023	.048	1.023 (1.000–1.046)
		Simple mode	2	0.019	.257	1.019 (0.987–1.053)
		Weighted mode	2	0.02	.201	1.020 (0.990–1.051)
Other and unspecified psoriasis	Aspartate	Inverse variance weighted	8	0.010	.033	1.010 (1.001–1.020)
		MR Egger	8	0.005	.715	1.005 (0.981–1.029)
		Weighted median	8	0.014	.019	1.014 (1.002–1.026)
		Simple mode	8	0.015	.147	1.015 (0.997–1.033)
		Weighted mode	8	0.015	.107	1.015 (0.999–1.031)
Other and unspecified psoriasis	Cysteine	Inverse variance weighted	8	0.006	.033	1.006 (1.000–1.012)
		MR Egger	8	0.01	.238	1.010 (0.995–1.024)
		Weighted median	8	0.007	.075	1.007 (0.999–1.014)
		Simple mode	8	0.007	.3	1.007 (0.995–1.018)
		Weighted mode	8	0.01	.105	1.010 (0.999–1.022)

Beta = the effect size of the effect allele, CI = confidence interval, IVW = inverse variance weighted analysis, MR = Mendelian randomization, nsnp = number of single nucleotide polymorphisms, OR = odds ratio.

### 3.6. The role of serum inflammatory factors and metabolites in mediating the effect of different kinds of psoriasis on abnormal spermatozoa

Mediation analyses were conducted on 4 serum metabolites, namely, X-09026, methylcysteine, aspartate, and cysteine (Table 5). The findings indicated that X-09026 and MMIF serve as mediators in the regulation of the causal association between Psoriasis vulgaris and abnormal spermatozoa; the mediation effects are 0.063 and 0.023, respectively, and the mediation proportions are 49.219% and 18.000%, respectively. Additionally, methylcysteine also acts as a mediator in the regulation of the causal relationship between arthropathic psoriasis and abnormal spermatozoa, demonstrating a mediation effect of 0.038 and a mediation proportion of 35.849%. However, aspartate and cysteine do not mediate the causal relationship between other and unspecified psoriasis and abnormal spermatozoa, as the direction of their mediation effects is not consistent with the total effect.

**Table 5:** The causal effect of different kinds of psoriasis on abnormal spermatozoa-related serum metabolites.

Exposure	Mediator	Outcome	Total effect	Mediator effect (95% CI)	Mediated proportion (95% CI)
Psoriasis vulgaris	X-09026	Abnormal spermatozoa	0.128	0.063 (0.006, 0.133)	49.22% (4.69%, 103.91%)
Psoriasis vulgaris	MMIF (macrophage migration inhibitory factor)	Abnormal spermatozoa	0.128	0.022 (−0.011, 0.056)	17.5% (−8.6%, 43.8%)
Arthropathic psoriasis	Methylcysteine	Abnormal spermatozoa	0.106	0.039 (0.002, 0.085)	36.3% (2.2%, 80.4%)
Other and unspecified psoriasis	Aspartate	Abnormal spermatozoa	0.081	−0.026	NA
Other and unspecified psoriasis	Cysteine	Abnormal spermatozoa	0.081	−0.012	NA

Beta = the effect size of the effect allele, CI = confidence interval, nsnp = number of single nucleotide polymorphisms, OR = odds ratio.

### 3.7. Sensitivity analysis results

Tables S10 to S13, Supplemental Digital Content, https://links.lww.com/MD/Q116 contain the sensitivity analysis tables. The findings indicate the presence of heterogeneity and genetic pleiotropy in the causal impact of psoriasis on sexual dysfunction (*P*_Cochran Q_ = .016), as well as heterogeneity in the causal effect of psoriasis vulgaris on sexual dysfunction (*P*_Cochran Q_ = .049). Conversely, no heterogeneity or genetic pleiotropy was observed in the remaining causal analysis outcomes (*P*_Cochran Q_ > .05, *P*_MRegger interpreter_ > .05, *P*_MRPRESSO GLOBAL_ > .05).

## 4. Discussion

Psoriasis has been identified as having a correlation with abnormal spermatozoa, albeit with limited research conducted in this domain. Multiple studies have suggested the existence of variances in semen parameters among individuals affected by psoriasis compared to the broader population. There was a significant decrease observed in the total sperm count, sperm motility, and percentage of spermatozoa with normal morphology among the patients in comparison to the normal controls.^[30]^ While traditional views tend to view sexual dysfunction as a psycho-physical sequela resulting from psoriasis, our study suggests, through genetic evidence, that there may be a reverse causal pathway for this association.This contradiction may stem from differences in research methodology: preclinical observational studies are susceptible to confounding factors (e.g., bidirectional effects of depressive state on sexual function and disease activity), whereas the Mendelian randomization method effectively circumvents some of these biases through IV screening.

The precise relationship between sexual dysfunction and psoriasis remains incompletely understood, although potential associations have been identified.^[31]^ Psoriasis is commonly acknowledged as an immune-mediated inflammatory disorder, wherein inflammatory responses significantly contribute to its development.^[32]^ Several investigations have proposed a potential connection between inflammatory responses and sexual dysfunction.^[13]^ Nevertheless, the present study revealed a counterintuitive finding through bidirectional Mendelian randomization analysis: male sexual dysfunction may have a potentially protective effect on the development of psoriasis, rather than a one-way worsening of the relationship as traditionally perceived. This finding poses an important challenge to existing theoretical frameworks and provides new perspectives on the complexity of psoriasis-reproductive health interactions. The precise mechanisms underlying this association, however, remain elusive and require further investigation. Sexual dysfunction and psoriasis are intricate issues involving the interplay of various factors. Further research is needed to better understand this relationship. The precise mechanisms underlying this association, however, remain elusive and require further investigation. Sexual dysfunction and psoriasis are intricate issues involving the interplay of various factors. Further research is needed to better understand this relationship.

Our study identified 3 potential mediators, namely, X-09026, MMIF, and methylcysteine. While the serum metabolite X-09026 remains chemically unannotated in public databases (HMDB, PubChem), our structural predictions using ClassyFire and pathway mapping suggest it is likely a *branched medium-chain fatty acid derivative* with hydroxyl groups, potentially involved in mitochondrial β-oxidation pathways critical for cellular energy metabolism. This aligns with its observed correlation with citrate and acetylcarnitine, metabolites central to sperm motility and ATP production. Mendelian randomization analysis confirmed a causal association between X-09026 and diastolic blood pressure (IVW β = 0.32, *P* = 1.2 × 10^−5^), though its mechanistic role in blood pressure regulation warrants further validation. The interplay between blood pressure dysregulation and abnormal spermatogenesis remains incompletely understood. While epidemiological studies report a 50% to 60% global decline in semen quality over 4 decades 6, direct causal links to hypertension are inconclusive.^[33]^ Our mediation analysis suggests X-09026 may partially explain this association through energy metabolism pathways, as mitochondrial dysfunction is implicated in both hypertension-induced vascular damage and sperm motility defects. However, current evidence remains preliminary.

MMIF is a crucial immunoregulatory factor that significantly contributes to immune and inflammatory responses.^[34]^ Extensive research has demonstrated the significance of MMIF in the maturation and motility of sperm.^[35]^ This factor is secreted by testicular interstitial cells and expressed in both spermatogonia and spermatocytes.^[36]^ The concentration of MMIF serves as a reliable indicator of fertilization capability.^[37]^ However, it is important to note that the involvement of MMIF in inflammation processes may have adverse effects on sperm development.^[38]^ Excessive inflammatory responses and immune reactions can potentially lead to reproductive damage.^[39]^ Furthermore, MMIF is known to exert a substantial influence on the pathophysiological mechanisms underlying psoriasis.^[40–42]^ Current research consistently supports the notion that MMIF levels exhibit a marked elevation in individuals afflicted with psoriasis.^[43]^ MMIF actively participates in the initiation and advancement of psoriasis by modulating the expression of inflammatory mediators.^[44]^ However, it has been observed in certain studies that the expression of MMIF is significantly higher in peripheral blood mononuclear cells, whereas its expression is notably reduced in the epidermis of psoriatic lesions.^[45]^ This finding implies that the association between psoriasis and abnormal spermatozoa may vary depending on the location. Consequently, if psoriatic lesions are situated in the vicinity of the testicles and epididymis, there is a corresponding increase in the likelihood of developing abnormal spermatozoa.

Methylcysteine, a compound derived from cysteine, is an amino acid that serves as a prevalent constituent in proteins. Cysteine is involved in the biosynthesis of glutathione, a vital antioxidant that safeguards sperm from oxidative stress, thereby preserving their structural and functional integrity.^[46,47]^ At present, there is no conclusive scientific evidence to establish a direct correlation between cysteine and psoriatic arthritis. Nevertheless, the metabolic byproduct of cysteine, homocysteine,^[48]^ could potentially be linked to the underlying mechanisms of psoriasis. It is hypothesized that the presence of methylcysteine may disrupt the regular functioning of cysteine, consequently affecting the process of sperm development.

Nevertheless, it is important to acknowledge the limitations of this study. First, there were inconsistencies in the inclusion criteria for IVs, with more lenient screening criteria employed in the reverse MR and causal effect analysis of serum metabolites on abnormal spermatozoa. Second, the study exclusively utilized the IVW method as the MR analysis technique due to its superior statistical power. Third, the mediation analysis in this study employed a stepwise approach, which offers a more comprehensive understanding of the clinical implications. In addition, the chemical properties of X-09026 are unknown and need to be further characterized by targeted metabolomics. It is important to note that the entirety of the data utilized in this study was obtained exclusively from European populations. Consequently, additional research is imperative to ascertain the extent to which these findings can be extrapolated to other populations.

## 5. Conclusion

After in-depth study and observation, it was found that psoriasis, a skin disease and sperm abnormalities seem to exist between a subtle link. Specifically, psoriasis may have adverse effects on sperm through metabolites such as X-09026, MMIF, and methylcysteine, leading to sperm abnormalities. These metabolites are often present at abnormal levels in patients with psoriasis and may interfere with the normal production, development and function of sperm through a complex series of biochemical reactions. This finding not only reveals a potential link between psoriasis and sperm abnormalities, but also provides new clues to our deeper understanding of the pathogenesis of psoriasis. At present, the specific mechanism between these metabolites and sperm abnormality is not completely clear, and further research and exploration are needed.

## Author contributions

**Conceptualization:** Sicheng Ma, Wenbang Liu.

**Funding acquisition:** Zixue Sun, Chenming Zhang.

**Methodology:** Wenlin Yu, Jing Hu.

**Resources:** Ruimin Ma, Xiaohui Hao.

**Writing – original draft:** Sicheng Ma, Ruimin Ma.

**Writing – review & editing:** Yinuo Zhang.

## Supplementary Material
