# Supplementary material for: The causal effect between psoriasis and male reproductive health, and the mediating effect of serum inflammatory factors and metabolites
Source: Medicine (Baltimore). 2025 Sep 26;104(39):e44758. doi: 10.1097/MD.0000000000044758 (PMC13593155; doi:10.1097/MD.0000000000044758)
Supplement: Supplementary file 1 [file medi-104-e44758-s001.pdf]

Supplementary Table 1: The instrumental variables associated with psoriasis.

EXPOSURE	SNP	EA	NEA	EAF	BETA	SE	P	F
finn-b-L12_PSORIASIS	rs12713428	C	A	0.2487	0.1694	0.0261	8.1077E-11	42.12557068
finn-b-L12_PSORIASIS	rs17728338	A	G	0.07331	0.3092	0.0439	1.762E-12	49.60779573
finn-b-L12_PSORIASIS	rs12188300	T	A	0.05898	0.4331	0.0495	2.2439E-18	76.55366187
finn-b-L12_PSORIASIS	rs674451	C	T	0.343	0.1307	0.0235	2.823E-08	30.93253056
finn-b-L12_PSORIASIS	rs13210419	A	G	0.05858	1.1157	0.0511	1.104E-105	476.7086868
finn-b-L12_PSORIASIS	rs28752856	G	C	0.1106	0.833	0.0393	5.901E-100	449.2673957
finn-b-L12_PSORIASIS	rs4947309	T	A	0.2334	0.5524	0.0274	3.8619E-90	406.4491449
finn-b-L12_PSORIASIS	rs4713605	A	T	0.3321	0.1526	0.0241	2.347E-10	40.09359343
finn-b-L12_PSORIASIS	rs9481169	T	G	0.07817	0.2515	0.0422	2.469E-09	35.51821051
finn-b-L12_PSORIASIS	rs1611309	T	C	0.6266	0.241	0.0267	1.6021E-19	81.47259746
finn-b-L12_PSORIASIS	rs60600003	G	T	0.1017	0.2128	0.0372	1.033E-08	32.72332062
finn-b-L12_PSORIASIS	rs181316459	C	G	0.04736	0.3544	0.0553	1.497E-10	41.07117842
finn-b-L12_PSORIASIS	rs10829130	A	G	0.11	0.1965	0.0359	4.241E-08	29.95961391
finn-b-L12_PSORIASIS	rs138009430	A	C	0.07849	0.2538	0.0423	1.938E-09	36
finn-b-L12_PSORIASIS	rs2021511	T	C	0.2664	-0.1387	0.0254	4.747E-08	29.81847914
finn-b-L12_PSORIASIS	rs28998802	A	G	0.1851	0.1672	0.0289	7.407E-09	33.47162989
finn-b-L12_PSORI_VULG	rs12713428	C	A	0.2485	0.1867	0.0325	9.1489E-09	33.00060592
finn-b-L12_PSORI_VULG	rs74817271	A	G	0.07347	0.3599	0.0547	4.7457E-11	43.29014502
finn-b-L12_PSORI_VULG	rs139298380	A	G	0.02713	0.649	0.0915	1.3259E-12	50.30917615
finn-b-L12_PSORI_VULG	rs6556423	T	C	0.6437	-0.2482	0.0292	1.8051E-17	72.25
finn-b-L12_PSORI_VULG	rs28749130	A	T	0.3396	0.2928	0.0298	8.4004E-23	96.54051619
finn-b-L12_PSORI_VULG	rs16898116	C	T	0.01566	0.6609	0.1172	1.718E-08	31.79920631
finn-b-L12_PSORI_VULG	rs4947309	G	A	0.3972	-0.3175	0.0333	1.3621E-21	90.90734879
finn-b-L12_PSORI_VULG	rs28752856	G	C	0.1102	1.036	0.0501	5.4979E-95	427.6062645
finn-b-L12_PSORI_VULG	rs28732140	A	G	0.1213	0.9597	0.0462	1.05E-95	431.5061983
finn-b-L12_PSORI_VULG	rs74630264	A	G	0.08167	0.2904	0.0518	2.036E-08	31.42922735
finn-b-L12_PSORI_NAS	rs28751318	A	A	0.1656	0.9745	0.0853	3.1732E-30	130.5165618
finn-b-L12_PSORI_NAS	rs1611309	T	C	0.6259	0.3366	0.0616	4.5951E-08	29.85841837
finn-b-L12_PSORI_NAS	rs28732092	T	G	0.05576	2.2912	0.1409	1.7869E-59	264.4259145
finn-b-L12_PSORI_ARTH	rs17728338	A	G	0.07309	0.3874	0.0704	3.828E-08	30.28125807
finn-b-L12_PSORI_GUTT	rs12199223	A	T	0.05946	3.9737	0.3155	2.2983E-36	158.6322286
finn-b-L12_PSORI_GUTT	rs28752856	G	C	0.1091	2.5277	0.2258	4.4555E-29	125.3150356
finn-b-L12_PSORI_GUTT	rs9265203	T	G	0.2312	1.2755	0.145	1.4031E-18	77.37932224
finn-b-L12_PSORI_NAS	rs28751318	A	A	0.1656	0.9745	0.0853	3.1732E-30	130.5165618
finn-b-L12_PSORI_NAS	rs1611309	T	C	0.6259	0.3366	0.0616	4.5951E-08	29.85841837
finn-b-L12_PSORI_NAS	rs28732092	T	G	0.05576	2.2912	0.1409	1.7869E-59	264.4259145
ukb-b-10537	rs11581607	A	G	0.06676	-0.0026	0.00044	5.2E-09	34.10764232
ukb-b-10537	rs4112787	T	C	0.65853	0.0016	0.00023	1.5999E-12	49.86327082
ukb-b-10537	rs842636	A	G	0.43542	-0.0013	0.00022	2.5E-09	35.54447158
ukb-b-10537	rs2111485	G	A	0.60673	0.0014	0.00023	1E-09	37.26953164
ukb-b-10537	rs11135059	A	G	0.32907	-0.0025	0.00024	1.3999E-26	113.8258817
ukb-b-10537	rs848	C	A	0.81619	0.0018	0.00029	2.8E-10	39.81814355
ukb-b-10537	rs12188300	T	A	0.09347	0.0051	0.00038	1.5999E-41	182.1626789
ukb-b-10537	rs12189871	T	C	0.09053	0.0214	0.00038	1E-200	3089.497665
ukb-b-10537	rs28367705	A	G	0.11093	0.007	0.00045	1.9999E-55	245.9573587
ukb-b-10537	rs9277937	C	T	0.09632	0.0029	0.00038	3.1996E-14	57.61686885
ukb-b-10537	rs33980500	T	C	0.07439	0.0028	0.00042	2.2999E-11	44.67289573
ukb-b-10537	rs2735003	G	T	0.20799	-0.0026	0.00027	1.1E-21	91.46079101
ukb-b-10537	rs582757	T	C	0.72624	-0.0014	0.00025	2.5E-08	31.03173247
ukb-b-10537	rs9265203	T	G	0.45295	0.0062	0.00025	3.199E-134	607.885671
ukb-b-10537	rs13191494	C	G	0.10022	0.0044	0.00039	2.7002E-28	121.7017866
ukb-b-10537	rs11795343	C	T	0.40133	-0.0014	0.00023	1.8E-10	40.70860256
ukb-b-10537	rs7951925	G	A	0.36863	-0.0013	0.00023	2.4E-08	31.12546875
ukb-b-10537	rs8016947	G	T	0.56182	0.0016	0.00022	4.0004E-13	52.64538821
ukb-b-10537	rs28998802	A	G	0.14048	0.0019	0.00032	6.5999E-09	33.64637007
ukb-b-10537	rs11085725	T	C	0.2924	-0.0017	0.00024	7.7002E-12	46.84896948

ukb-b-10537 rs632376	G	A	0.41992	-0.0013	0.00022	4.3E-09	34.49308093

Supplementary Table 2: The instrumental variables associated with male reproductive health.

EXPOSURE	SNP	EA	NEA	EAF	BETA	SE	P	F
finn-b-N14_MALEINFERT	rs1334881	C	G	0.6003	-0.2624	0.0563	3.13199E-06	21.72255331
finn-b-N14_MALEINFERT	rs55841791	T	C	0.1261	0.4242	0.0864	9.24209E-07	24.1053723
finn-b-N14_MALEINFERT	rs35240977	A	G	0.2927	-0.2828	0.0605	2.95202E-06	21.84982993
finn-b-N14_MALEINFERT	rs74607884	C	T	0.0242	0.9149	0.1932	0.000002174	22.42502888
finn-b-N14_MALEINFERT	rs114146352	A	G	0.0528	0.7104	0.1329	8.931E-08	28.57300674
finn-b-N14_MALEINFERT	rs75935512	A	G	0.0104	1.5296	0.3161	0.000001309	23.41567913
finn-b-N14_MALEINFERT	rs144301968	A	G	0.0047	2.265	0.4929	4.31698E-06	21.11634634
finn-b-N14_MALEINFERT	rs12803569	T	C	0.0133	1.2955	0.271	1.75198E-06	22.85263341
finn-b-N14_MALEINFERT	rs4943344	C	T	0.2577	0.3055	0.064	1.79602E-06	22.78570557
finn-b-N14_MALEINFERT	rs41301797	C	T	0.0121	1.3358	0.2904	4.21599E-06	21.1587328
finn-b-N14_MALEINFERT	rs113678355	T	C	0.2402	0.3246	0.066	8.76294E-07	24.1885124
finn-b-N14_MALEINFERT	rs12967471	A	G	0.3042	0.2943	0.0616	0.000001737	22.82543694
finn-b-N14_MALEINFERT	rs78211661	C	T	0.0039	2.5161	0.5191	1.25101E-06	23.49382545
finn-b-N14_MALEINFERT	rs139074528	C	G	0.0034	2.8357	0.603	2.56998E-06	22.114949
finn-b-R18_ABNORMAL_SPERMATOZ	rs12031361	A	G	0.509	-0.2323	0.0479	0.000001226	23.51946252
finn-b-R18_ABNORMAL_SPERMATOZ	rs16863772	T	C	0.055	0.5087	0.1045	1.13399E-06	23.696865
finn-b-R18_ABNORMAL_SPERMATOZ	rs6547871	C	T	0.9704	-0.7761	0.1586	9.91791E-07	23.9457807
finn-b-R18_ABNORMAL_SPERMATOZ	rs12386663	C	T	0.0932	-0.4011	0.0858	2.94999E-06	21.85399042
finn-b-R18_ABNORMAL_SPERMATOZ	rs117045401	T	A	0.0256	0.7244	0.1554	3.15697E-06	21.72975623
finn-b-R18_ABNORMAL_SPERMATOZ	rs1793827	C	G	0.4468	0.2226	0.0486	4.59896E-06	20.97866179
finn-b-R18_ABNORMAL_SPERMATOZ	rs28564768	G	A	0.0608	0.516	0.1056	1.01801E-06	23.87654959
finn-b-R18_ABNORMAL_SPERMATOZ	rs12899909	T	G	0.1441	0.335	0.069	0.000001191	23.57172863
finn-b-R18_ABNORMAL_SPERMATOZ	rs116931900	T	G	0.0213	0.8178	0.1666	9.14998E-07	24.09595915
finn-b-R18_ABNORMAL_SPERMATOZ	rs149985919	A	G	0.0034	2.0424	0.4308	0.000002133	22.47657917
finn-b-R18_ABNORMAL_SPERMATOZ	rs10407351	T	C	0.5128	-0.2224	0.0478	3.30103E-06	21.64780028
finn-b-R18_ABNORMAL_SPERMATOZ	rs73930587	A	G	0.2175	-0.2675	0.0585	4.88304E-06	20.90912411
finn-b-R18_ABNORMAL_SPERMATOZ	rs8131512	G	A	0.0595	0.4766	0.1038	4.42599E-06	21.08207573
finn-b-F5_SEXDYS	rs354840	T	C	0.4458	-0.5206	0.1138	4.77705E-06	20.92781095
finn-b-F5_SEXDYS	rs2154047	A	C	0.9117	-0.9911	0.2131	3.29701E-06	21.63057443
finn-b-F5_SEXDYS	rs76021898	A	G	0.0243	2.2058	0.4579	1.45201E-06	23.20550253
finn-b-F5_SEXDYS	rs462458	G	A	0.7257	-0.6373	0.1305	1.03901E-06	23.84881549
finn-b-ERECTILE_DYSFUNCTION	rs6765401	G	A	0.3273	0.2121	0.0452	2.74701E-06	22.01934862
finn-b-ERECTILE_DYSFUNCTION	rs11736880	C	T	0.2066	-0.2518	0.0527	0.000001758	22.8291752
finn-b-ERECTILE_DYSFUNCTION	rs67376322	A	T	0.1786	-0.2664	0.056	1.95299E-06	22.63040816
finn-b-ERECTILE_DYSFUNCTION	rs6936413	G	C	0.2527	0.2247	0.0489	4.29596E-06	21.11487071
finn-b-ERECTILE_DYSFUNCTION	rs140901789	A	T	0.0252	0.651	0.1411	3.97603E-06	21.28668089
finn-b-ERECTILE_DYSFUNCTION	rs10241701	G	A	0.2436	0.2368	0.0498	0.000002037	22.61021596
finn-b-ERECTILE_DYSFUNCTION	rs13258548	G	A	0.2362	-0.2307	0.0501	0.000004048	21.2040948
finn-b-ERECTILE_DYSFUNCTION	rs6983267	T	G	0.4767	-0.2173	0.0424	3.08901E-07	26.265625
finn-b-ERECTILE_DYSFUNCTION	rs78093953	C	G	0.0291	0.6131	0.1342	4.92006E-06	20.87168927
finn-b-ERECTILE_DYSFUNCTION	rs3133073	A	T	0.7146	-0.2404	0.0475	4.26098E-07	25.61425374
finn-b-ERECTILE_DYSFUNCTION	rs145922598	T	C	0.0125	1.0376	0.2056	4.51305E-07	25.46910627
finn-b-ERECTILE_DYSFUNCTION	rs116902888	T	C	0.0191	0.7984	0.1682	2.05499E-06	22.53144648
finn-b-ERECTILE_DYSFUNCTION	rs35929622	G	A	0.4062	-0.2291	0.0438	1.67201E-07	27.35910948
finn-b-E4_TESTIDYS	rs11573628	T	C	0.0529	0.9586	0.2033	2.42298E-06	22.23310362
finn-b-E4_TESTIDYS	rs148318232	G	A	0.0188	1.69	0.3693	4.72596E-06	20.94183791
finn-b-E4_TESTIDYS	rs116865925	A	G	0.0235	1.7227	0.3344	2.58601E-07	26.53913538
finn-b-E4_TESTIDYS	rs142700974	A	G	0.0289	1.3154	0.2877	4.81704E-06	20.90431549

Supplementary Table 3: The instrumental variables associated with serum metabolites.

EXPOSURE	SNP	EA	NEA	EAF	BETA	SE	P	F
M00053.m	rs11596604	A	G	0.0236	0.0323	0.0069	2.52E-06	21.9133
M00053.m	rs16977047	T	C	0.7034	-0.0056	0.0011	1.08E-06	25.9174
M00053.m	rs2199619	T	C	0.2787	0.0054	0.0012	5.23E-06	20.25
M00053.m	rs2657879	A	G	0.8179	0.015	0.0017	6.15E-18	77.8547
M00053.m	rs2748991	T	C	0.446	-0.007	0.0015	2.34E-06	21.7778
M00053.m	rs7078003	T	C	0.1654	0.0087	0.0018	1.84E-06	23.3611
M00053.m	rs7394051	T	C	0.7014	0.0055	0.0012	2.18E-06	21.0069
M00053.m	rs780093	T	C	0.3995	-0.0058	0.0011	1.93E-07	27.8017
M00054.m	rs1000137	T	C	0.475	-0.005	0.001	5.73E-07	25
M00054.m	rs10007704	T	C	0.6418	-0.0044	0.0009	1.24E-06	23.9012
M00054.m	rs1013667	A	G	0.6087	-0.0041	0.0009	5.67E-06	20.7531
M00054.m	rs1015795	A	G	0.5122	0.0043	0.0009	2.26E-06	22.8272
M00054.m	rs1015993	T	C	0.4684	0.0041	0.0009	7.31E-06	20.7531
M00054.m	rs1016522	A	G	0.5803	0.0058	0.0009	1.59E-10	41.5309
M00054.m	rs10171387	A	G	0.9051	0.008	0.0018	5.14E-06	19.7531
M00054.m	rs10225863	T	C	0.3949	-0.004	0.0009	9.75E-06	19.7531
M00054.m	rs10495209	T	C	0.4862	0.004	0.0009	8.02E-06	19.7531
M00054.m	rs10498266	T	G	0.4674	0.0043	0.0009	2.35E-06	22.8272
M00054.m	rs10750415	T	G	0.3959	0.0042	0.0009	2.77E-06	21.7778
M00054.m	rs10784496	A	G	0.5375	0.0045	0.0009	5.09E-07	25
M00054.m	rs10805503	T	C	0.6272	0.0043	0.0009	2E-06	22.8272
M00054.m	rs10807322	A	T	0.5866	0.0043	0.0009	1.97E-06	22.8272
M00054.m	rs10822056	T	C	0.5141	0.0042	0.0009	2.65E-06	21.7778
M00054.m	rs10836364	T	C	0.607	0.005	0.001	5.73E-07	25
M00054.m	rs10846667	T	C	0.4861	0.0045	0.0009	1.65E-06	25
M00054.m	rs10881004	T	C	0.4824	0.0041	0.0009	3.39E-06	20.7531
M00054.m	rs10900784	A	C	0.4158	-0.0044	0.0009	9.74E-07	23.9012
M00054.m	rs10927101	A	C	0.3738	-0.0046	0.0009	2.72E-07	26.1235
M00054.m	rs10931041	T	C	0.5756	-0.0042	0.0009	3.36E-06	21.7778
M00054.m	rs11678851	T	C	0.4138	0.0041	0.0009	5.94E-06	20.7531
M00054.m	rs11720862	T	G	0.6034	-0.0046	0.0009	3.58E-07	26.1235
M00054.m	rs11729831	T	G	0.6133	0.004	0.0009	8.61E-06	19.7531
M00054.m	rs1179636	A	C	0.5356	-0.0045	0.001	1.83E-06	20.25
M00054.m	rs11802417	T	C	0.5816	-0.0041	0.0009	4.47E-06	20.7531
M00054.m	rs11804149	T	C	0.4678	0.0041	0.0009	4.61E-06	20.7531
M00054.m	rs11937375	C	G	0.68	-0.018	0.004	6.8E-06	20.25
M00054.m	rs1220605	A	G	0.6312	-0.0046	0.0009	9.29E-07	26.1235
M00054.m	rs12278762	T	C	0.5229	0.0043	0.0009	1.56E-06	22.8272
M00054.m	rs12468084	T	C	0.5436	-0.0044	0.0009	1.25E-06	23.9012
M00054.m	rs1249489	A	G	0.3741	0.0042	0.0009	3.68E-06	21.7778
M00054.m	rs12533939	A	T	0.3887	0.0045	0.0009	6.52E-07	25
M00054.m	rs1260326	T	C	0.404	0.0042	0.0009	2.74E-06	21.7778
M00054.m	rs13001325	T	C	0.3823	-0.004	0.0009	7.45E-06	19.7531
M00054.m	rs13008096	A	C	0.4038	-0.0042	0.0009	3.32E-06	21.7778
M00054.m	rs13056963	T	C	0.4376	0.0043	0.0009	5.58E-06	22.8272
M00054.m	rs13122250	T	C	0.5542	0.0062	0.0009	8.95E-12	47.4568
M00054.m	rs1330918	A	C	0.6132	0.0042	0.0009	2.21E-06	21.7778
M00054.m	rs1349704	A	G	0.4611	0.0044	0.0009	8.31E-07	23.9012
M00054.m	rs1373962	T	C	0.5973	0.005	0.0009	2.71E-08	30.8642
M00054.m	rs1412972	T	G	0.5006	0.0097	0.002	1.63E-06	23.5225
M00054.m	rs1418942	A	C	0.5751	-0.004	0.0009	7.13E-06	19.7531
M00054.m	rs1501803	T	C	0.4152	0.0042	0.0009	4.57E-06	21.7778
M00054.m	rs1508513	C	G	0.6354	0.0042	0.0009	2.36E-06	21.7778

M00054.m	rs1519309	T	C	0.6544	-0.0044	0.0009	1.21E-06	23.9012
M00054.m	rs154246	T	C	0.6103	-0.0049	0.0009	6.7E-08	29.642
M00054.m	rs1549225	T	C	0.607	0.0045	0.0009	6.06E-07	25
M00054.m	rs1559063	C	G	0.6209	0.0052	0.0009	7.82E-09	33.3827
M00054.m	rs1663501	A	G	0.4634	-0.0047	0.0009	2.44E-07	27.2716
M00054.m	rs16823952	T	G	0.9069	-0.0083	0.0018	5.08E-06	21.2623
M00054.m	rs16836288	A	C	0.044	0.0131	0.0029	5.6E-06	20.4055
M00054.m	rs1697901	A	G	0.3501	0.0049	0.0009	6.31E-08	29.642
M00054.m	rs17114354	A	G	0.9874	0.0248	0.0053	3.52E-06	21.8953
M00054.m	rs17698249	A	C	0.5682	-0.0041	0.0009	5.5E-06	20.7531
M00054.m	rs17744500	T	C	0.4582	-0.0041	0.0009	4.16E-06	20.7531
M00054.m	rs1790398	T	C	0.5227	0.0048	0.0009	5.79E-08	28.4444
M00054.m	rs1871053	A	G	0.4514	-0.0044	0.0009	7.68E-07	23.9012
M00054.m	rs1995960	A	G	0.5949	-0.0047	0.0009	1.63E-07	27.2716
M00054.m	rs1997238	A	G	0.5117	0.0045	0.0009	6.07E-07	25
M00054.m	rs201262	T	G	0.5563	0.0047	0.0009	1.36E-07	27.2716
M00054.m	rs2063601	A	C	0.2487	-0.0107	0.0023	5.24E-06	21.6427
M00054.m	rs207938	A	G	0.3963	-0.0043	0.0009	1.32E-06	22.8272
M00054.m	rs2091289	A	G	0.5943	0.0043	0.0009	1.5E-06	22.8272
M00054.m	rs2111118	T	C	0.6207	0.0051	0.0009	1.21E-08	32.1111
M00054.m	rs2126498	T	G	0.6176	-0.0042	0.0009	2.84E-06	21.7778
M00054.m	rs2178260	T	C	0.4251	0.0042	0.0009	3.59E-06	21.7778
M00054.m	rs2216061	A	C	0.512	0.0043	0.0009	2.21E-06	22.8272
M00054.m	rs2230532	T	C	0.349	0.005	0.001	5.73E-07	25
M00054.m	rs2349729	A	G	0.5141	-0.0041	0.0009	6.45E-06	20.7531
M00054.m	rs2383131	T	C	0.4403	-0.0043	0.0009	3.05E-06	22.8272
M00054.m	rs2432	T	C	0.4713	0.0046	0.0009	4.28E-07	26.1235
M00054.m	rs2484178	T	C	0.6063	0.0043	0.0009	1.75E-06	22.8272
M00054.m	rs2505644	T	C	0.558	0.005	0.001	5.73E-07	25
M00054.m	rs2510595	A	G	0.6113	-0.0045	0.0009	4.99E-07	25
M00054.m	rs2520117	A	G	0.4545	0.0045	0.0009	5.06E-07	25
M00054.m	rs2580344	T	C	0.6238	0.0044	0.0009	7.98E-07	23.9012
M00054.m	rs2604365	T	G	0.6371	0.0044	0.0009	1.45E-06	23.9012
M00054.m	rs2658761	T	C	0.4343	-0.0048	0.0009	6.75E-08	28.4444
M00054.m	rs2662269	A	C	0.417	0.0045	0.0009	7.12E-07	25
M00054.m	rs2688858	T	C	0.5742	0.0043	0.0009	2.84E-06	22.8272
M00054.m	rs2717586	A	G	0.3962	0.0043	0.0009	2.31E-06	22.8272
M00054.m	rs2722407	A	G	0.587	0.0041	0.0009	6.47E-06	20.7531
M00054.m	rs2769419	A	G	0.4777	0.004	0.0009	6.16E-06	19.7531
M00054.m	rs2798184	T	C	0.4314	-0.0049	0.0009	6.37E-08	29.642
M00054.m	rs2813760	A	G	0.5775	0.0044	0.0009	1.19E-06	23.9012
M00054.m	rs2834164	A	C	0.5041	-0.0045	0.0009	4.76E-07	25
M00054.m	rs284191	A	G	0.615	-0.006	0.001	1.97E-09	36
M00054.m	rs2868072	A	G	0.4829	0.004	0.0009	9.04E-06	19.7531
M00054.m	rs295142	A	G	0.5909	-0.0048	0.0009	1.54E-07	28.4444
M00054.m	rs2966079	T	C	0.5425	-0.0042	0.0009	2.53E-06	21.7778
M00054.m	rs2970335	A	C	0.4039	0.0044	0.0009	1.02E-06	23.9012
M00054.m	rs2977272	T	C	0.341	0.005	0.001	5.73E-07	25
M00054.m	rs33980	C	G	0.9071	-0.0077	0.0017	9.11E-06	20.5156
M00054.m	rs341973	T	C	0.493	0.005	0.001	5.73E-07	25
M00054.m	rs3742929	T	C	0.512	-0.0044	0.0009	1.01E-06	23.9012
M00054.m	rs3751181	A	G	0.6085	0.0041	0.0009	5.19E-06	20.7531
M00054.m	rs3772343	A	C	0.987	0.028	0.006	3.06E-06	21.7778
M00054.m	rs3773729	T	C	0.6291	0.004	0.0009	8.57E-06	19.7531
M00054.m	rs3780494	T	C	0.9855	-0.023	0.0051	7.02E-06	20.3383

M00054.m	rs38271	A	G	0.5925	-0.0051	0.0009	1.19E-08	32.1111
M00054.m	rs3851229	T	C	0.43	0.0045	0.0009	4.5E-07	25
M00054.m	rs3927971	T	C	0.5178	0.0042	0.0009	8.98E-06	21.7778
M00054.m	rs4063769	T	C	0.5301	-0.0043	0.0009	1.3E-06	22.8272
M00054.m	rs4144737	A	G	0.5818	0.004	0.0009	9.86E-06	19.7531
M00054.m	rs42162	T	C	0.5492	0.004	0.0009	8.22E-06	19.7531
M00054.m	rs428185	T	C	0.5851	-0.0045	0.0009	6.25E-07	25
M00054.m	rs4306882	T	G	0.6153	-0.0057	0.0009	2.52E-10	40.1111
M00054.m	rs430727	T	C	0.4628	0.0041	0.0009	5.13E-06	20.7531
M00054.m	rs4611118	A	G	0.609	-0.0044	0.0009	9.41E-07	23.9012
M00054.m	rs4615256	A	G	0.5327	0.0049	0.0009	4.99E-08	29.642
M00054.m	rs4684599	T	C	0.6319	-0.0042	0.0009	2.89E-06	21.7778
M00054.m	rs4686980	T	C	0.361	0.005	0.001	5.73E-07	25
M00054.m	rs4695289	A	G	0.3489	-0.0045	0.0009	7.95E-07	25
M00054.m	rs4702007	A	G	0.3929	-0.0041	0.0009	6.11E-06	20.7531
M00054.m	rs4751282	T	C	0.5421	0.0045	0.0009	7.41E-07	25
M00054.m	rs4755291	A	G	0.4644	-0.0041	0.0009	5.25E-06	20.7531
M00054.m	rs4849291	C	G	0.6092	0.0041	0.0009	5.93E-06	20.7531
M00054.m	rs4870304	A	G	0.5823	0.0041	0.0009	8.31E-06	20.7531
M00054.m	rs4880888	A	G	0.4785	-0.0046	0.0009	3.24E-07	26.1235
M00054.m	rs4926571	T	C	0.5231	-0.0048	0.0009	9.03E-08	28.4444
M00054.m	rs4958379	A	G	0.433	-0.005	0.0009	2.18E-08	30.8642
M00054.m	rs4972588	A	G	0.5138	-0.0046	0.0009	3.74E-07	26.1235
M00054.m	rs4973952	C	G	0.415	-0.0041	0.0009	6.47E-06	20.7531
M00054.m	rs4989308	A	G	0.3746	-0.0045	0.0009	8.43E-07	25
M00054.m	rs502654	T	C	0.559	-0.0046	0.0009	2.6E-07	26.1235
M00054.m	rs575257	A	G	0.6005	-0.0042	0.0009	3.51E-06	21.7778
M00054.m	rs5997110	T	C	0.523	-0.005	0.001	5.73E-07	25
M00054.m	rs6001129	T	C	0.134	-0.009	0.002	6.8E-06	20.25
M00054.m	rs603446	T	C	0.4448	0.0051	0.0009	1.38E-08	32.1111
M00054.m	rs6077611	T	G	0.4794	-0.0041	0.0009	5.32E-06	20.7531
M00054.m	rs6110544	T	C	0.4394	0.004	0.0009	7.01E-06	19.7531
M00054.m	rs6130271	T	C	0.5352	-0.004	0.0009	7.4E-06	19.7531
M00054.m	rs6435723	T	C	0.8977	-0.008	0.0018	6.91E-06	19.7531
M00054.m	rs6454864	T	C	0.3705	0.004	0.0009	8.87E-06	19.7531
M00054.m	rs6480970	A	G	0.6159	-0.0049	0.0009	4.29E-08	29.642
M00054.m	rs6517254	T	C	0.3971	-0.0042	0.0009	3.12E-06	21.7778
M00054.m	rs6534667	C	G	0.4186	-0.0043	0.0009	1.49E-06	22.8272
M00054.m	rs6537156	T	C	0.4485	0.004	0.0009	8.09E-06	19.7531
M00054.m	rs6586235	A	C	0.4598	0.0043	0.0009	1.62E-06	22.8272
M00054.m	rs6685230	T	C	0.3976	-0.0042	0.0009	7.78E-06	21.7778
M00054.m	rs6730726	A	G	0.5312	-0.004	0.0009	7.17E-06	19.7531
M00054.m	rs6747650	A	G	0.5825	-0.0041	0.0009	4.36E-06	20.7531
M00054.m	rs6759991	T	G	0.8623	0.0076	0.0016	2.52E-06	22.5625
M00054.m	rs6819555	A	G	0.5862	-0.0043	0.0009	3.69E-06	22.8272
M00054.m	rs682359	T	G	0.5812	0.0044	0.0009	6.54E-07	23.9012
M00054.m	rs6826247	T	C	0.5071	0.0048	0.0009	8.8E-08	28.4444
M00054.m	rs688627	T	C	0.5878	0.0042	0.0009	3.62E-06	21.7778
M00054.m	rs6910731	A	G	0.4497	0.0041	0.0009	6.68E-06	20.7531
M00054.m	rs6935961	A	G	0.5578	-0.0056	0.0009	3.75E-10	38.716
M00054.m	rs699856	T	C	0.3661	0.004	0.0009	8.01E-06	19.7531
M00054.m	rs7006068	T	C	0.6264	0.0045	0.0009	4.8E-07	25
M00054.m	rs7009696	T	C	0.6243	0.0041	0.0009	6.17E-06	20.7531
M00054.m	rs710580	A	C	0.3566	-0.005	0.0009	3.57E-08	30.8642
M00054.m	rs7109477	A	G	0.4588	-0.0047	0.0009	1.89E-07	27.2716

M00054.m	rs719564	A	G	0.6101	0.0044	0.0009	1.28E-06	23.9012
M00054.m	rs7198470	T	C	0.6081	0.004	0.0009	9E-06	19.7531
M00054.m	rs7230740	A	C	0.6655	0.0044	0.0009	1.56E-06	23.9012
M00054.m	rs7246393	A	G	0.5222	0.0043	0.0009	1.55E-06	22.8272
M00054.m	rs7463805	T	C	0.5213	-0.0053	0.0009	4.6E-09	34.679
M00054.m	rs7505177	T	G	0.4132	-0.0043	0.0009	5.86E-06	22.8272
M00054.m	rs7584842	T	C	0.5377	-0.005	0.0009	4.15E-08	30.8642
M00054.m	rs7594598	A	C	0.4257	0.004	0.0009	9.5E-06	19.7531
M00054.m	rs7638383	A	G	0.3984	0.0047	0.0009	1.64E-07	27.2716
M00054.m	rs7810390	A	G	0.5928	-0.0045	0.0009	5.61E-07	25
M00054.m	rs7843149	T	C	0.3896	-0.0041	0.0009	6.55E-06	20.7531
M00054.m	rs8006663	A	G	0.4556	-0.0046	0.0009	3.02E-07	26.1235
M00054.m	rs8021242	T	C	0.5899	0.0049	0.0009	5.18E-08	29.642
M00054.m	rs8088963	A	G	0.6387	0.0044	0.0009	8.29E-07	23.9012
M00054.m	rs8110890	T	G	0.4741	0.0045	0.0009	7.51E-07	25
M00054.m	rs860916	A	G	0.4232	-0.0047	0.0009	1.51E-07	27.2716
M00054.m	rs875184	T	C	0.403	-0.0041	0.0009	5.69E-06	20.7531
M00054.m	rs875283	A	G	0.4896	-0.0043	0.0009	2.34E-06	22.8272
M00054.m	rs881562	T	G	0.4978	0.0044	0.0009	1.45E-06	23.9012
M00054.m	rs893922	A	G	0.366	0.0042	0.0009	3.95E-06	21.7778
M00054.m	rs915281	T	C	0.5076	-0.0041	0.0009	4.42E-06	20.7531
M00054.m	rs928963	A	C	0.3985	0.0042	0.0009	3.62E-06	21.7778
M00054.m	rs9315440	A	G	0.5843	0.0048	0.0009	6.71E-08	28.4444
M00054.m	rs9330454	A	G	0.4006	-0.0066	0.0015	6.04E-06	19.36
M00054.m	rs937382	T	C	0.4466	-0.0046	0.0009	2.31E-07	26.1235
M00054.m	rs938040	T	C	0.107	0.0074	0.0017	9.12E-06	18.9481
M00054.m	rs9464137	A	C	0.5592	0.0041	0.0009	4.45E-06	20.7531
M00054.m	rs9511152	A	G	0.559	-0.005	0.0009	2.99E-08	30.8642
M00054.m	rs9517869	T	C	0.3661	-0.0046	0.0009	3.46E-07	26.1235
M00054.m	rs9523030	T	G	0.6269	0.0043	0.0009	5.65E-06	22.8272
M00054.m	rs9541214	T	C	0.653	-0.0042	0.0009	3.8E-06	21.7778
M00054.m	rs9543391	T	G	0.3993	0.0041	0.0009	6.39E-06	20.7531
M00054.m	rs9610322	A	G	0.5195	-0.0043	0.0009	1.12E-06	22.8272
M00054.m	rs9646527	A	T	0.3795	0.004	0.0009	9E-06	19.7531
M00054.m	rs9649963	T	C	0.634	0.0041	0.0009	5.84E-06	20.7531
M00054.m	rs972459	T	C	0.5787	0.005	0.0009	1.97E-08	30.8642
M00054.m	rs9878578	A	G	0.6016	0.0041	0.0009	5.32E-06	20.7531
M00054.m	rs993927	T	G	0.3773	0.0042	0.0009	2.64E-06	21.7778
M00054.m	rs9948912	A	G	0.3616	0.004	0.0009	9.58E-06	19.7531
M00054.m	rs9950125	A	G	0.4521	-0.004	0.0009	7.72E-06	19.7531
M00054.m	rs9989564	A	G	0.5142	-0.0042	0.0009	3.1E-06	21.7778
M00059.m	rs1527683	T	C	0.8	0.0061	0.0013	2.17E-06	22.0178
M00059.m	rs16868246	C	G	0.2113	0.0097	0.0013	1.78E-14	55.6746
M00059.m	rs2176664	T	C	0.2846	-0.0059	0.0012	2.03E-06	24.1736
M00059.m	rs4288369	C	G	0.0134	-0.0453	0.0102	9.31E-06	19.724
M00059.m	rs4768741	C	G	0.9153	-0.015	0.0032	3.52E-06	21.9727
M00059.m	rs7014133	A	G	0.0119	-0.0561	0.0109	2.74E-07	26.4894
M00059.m	rs715	T	C	0.7135	0.0059	0.0012	1.65E-06	24.1736
M00060.m	rs10004285	A	G	0.374	0.005	0.001	5.73E-07	25
M00060.m	rs1003887	T	C	0.651	-0.005	0.001	5.73E-07	25
M00060.m	rs10190188	T	C	0.662	0.005	0.001	5.73E-07	25
M00060.m	rs1025466	T	C	0.4062	-0.0041	0.0009	6.25E-06	20.7531
M00060.m	rs10258601	A	G	0.4382	0.0044	0.0009	6.73E-07	23.9012
M00060.m	rs1038555	T	C	0.4023	-0.004	0.0009	7.99E-06	19.7531
M00060.m	rs1043991	T	C	0.6375	0.0048	0.0009	8.33E-08	28.4444

M00060.m	rs10487377	A	G	0.5764	0.0046	0.0009	4.09E-07	26.1235
M00060.m	rs10500907	A	G	0.3597	0.0042	0.0009	3.55E-06	21.7778
M00060.m	rs10736117	A	G	0.6265	-0.004	0.0009	8.5E-06	19.7531
M00060.m	rs10763843	A	G	0.4283	-0.0042	0.0009	3.45E-06	21.7778
M00060.m	rs10763940	A	G	0.3654	0.0048	0.0009	1.17E-07	28.4444
M00060.m	rs10798580	A	G	0.4216	-0.0042	0.0009	3.64E-06	21.7778
M00060.m	rs10810580	T	C	0.5583	0.0046	0.0009	3.28E-07	26.1235
M00060.m	rs10828678	A	G	0.5105	0.004	0.0009	7.2E-06	19.7531
M00060.m	rs10887020	T	C	0.5133	0.004	0.0009	7.29E-06	19.7531
M00060.m	rs10911251	A	C	0.5759	-0.0047	0.0009	1.58E-07	27.2716
M00060.m	rs10923016	A	G	0.6191	0.0053	0.0009	2.88E-09	34.679
M00060.m	rs10937762	A	G	0.5674	0.0044	0.0009	1.53E-06	23.9012
M00060.m	rs10954411	A	T	0.3772	0.0043	0.0009	1.81E-06	22.8272
M00060.m	rs10973683	A	C	0.4002	-0.0041	0.0009	6.54E-06	20.7531
M00060.m	rs11007082	T	G	0.3779	0.0043	0.0009	1.84E-06	22.8272
M00060.m	rs1104999	T	C	0.4822	0.0041	0.0009	3.51E-06	20.7531
M00060.m	rs11117808	A	C	0.4457	0.004	0.0009	8.4E-06	19.7531
M00060.m	rs11124743	A	G	0.6354	0.0043	0.0009	1.91E-06	22.8272
M00060.m	rs11144511	T	C	0.4497	-0.0042	0.0009	8.42E-06	21.7778
M00060.m	rs11183620	A	G	0.4676	0.0041	0.0009	4.68E-06	20.7531
M00060.m	rs11203041	A	G	0.4458	-0.0043	0.0009	1.95E-06	22.8272
M00060.m	rs11578928	A	C	0.4246	0.0041	0.0009	4.75E-06	20.7531
M00060.m	rs11657125	T	C	0.5604	0.0041	0.0009	5.71E-06	20.7531
M00060.m	rs11868948	A	G	0.6435	-0.0042	0.0009	4.63E-06	21.7778
M00060.m	rs11952637	A	G	0.4212	0.0043	0.0009	4.31E-06	22.8272
M00060.m	rs11988092	T	C	0.6185	-0.0046	0.0009	3.29E-07	26.1235
M00060.m	rs11999261	A	T	0.0943	-0.0087	0.0017	6.56E-07	26.1903
M00060.m	rs12047618	T	G	0.3271	0.0046	0.0009	4.25E-07	26.1235
M00060.m	rs12196614	A	T	0.9386	0.0107	0.0024	6.42E-06	19.8767
M00060.m	rs12344504	T	C	0.5957	-0.004	0.0009	6.7E-06	19.7531
M00060.m	rs12468540	A	G	0.5563	0.0041	0.0009	5.76E-06	20.7531
M00060.m	rs12498703	A	G	0.5998	-0.0042	0.0009	2.86E-06	21.7778
M00060.m	rs12517096	A	G	0.422	0.0041	0.0009	8.41E-06	20.7531
M00060.m	rs12612007	A	G	0.3461	0.0044	0.0009	1.2E-06	23.9012
M00060.m	rs1278312	T	C	0.4008	-0.0042	0.0009	2.87E-06	21.7778
M00060.m	rs1292666	T	C	0.4667	0.0045	0.0009	6.44E-07	25
M00060.m	rs13130810	T	C	0.4401	0.004	0.0009	7.79E-06	19.7531
M00060.m	rs13147572	A	G	0.5809	-0.0047	0.0009	1.99E-07	27.2716
M00060.m	rs13165077	A	G	0.3821	0.0041	0.0009	8.98E-06	20.7531
M00060.m	rs1334318	A	G	0.3572	0.0044	0.0009	1.01E-06	23.9012
M00060.m	rs1360820	A	G	0.4963	-0.0042	0.0009	2.15E-06	21.7778
M00060.m	rs1368532	T	C	0.3842	-0.004	0.0009	8.86E-06	19.7531
M00060.m	rs1428203	T	G	0.4117	0.0045	0.0009	5.51E-07	25
M00060.m	rs1440581	T	C	0.4592	-0.0081	0.0009	1.44E-19	81
M00060.m	rs1444212	A	G	0.6413	0.004	0.0009	9.48E-06	19.7531
M00060.m	rs1463301	T	C	0.5742	-0.0049	0.0009	7.49E-08	29.642
M00060.m	rs1519309	T	C	0.6543	-0.0043	0.0009	1.87E-06	22.8272
M00060.m	rs1541245	T	C	0.5318	-0.0048	0.0009	7.99E-08	28.4444
M00060.m	rs1558789	A	C	0.51	0.0045	0.0009	3.52E-07	25
M00060.m	rs1564480	A	G	0.4765	0.0045	0.0009	6.68E-07	25
M00060.m	rs1654495	A	C	0.5709	0.0048	0.0009	1.04E-07	28.4444
M00060.m	rs167648	A	C	0.4439	-0.0041	0.0009	4.52E-06	20.7531
M00060.m	rs1677193	A	C	0.6122	-0.0044	0.0009	1.25E-06	23.9012
M00060.m	rs1705832	T	C	0.395	0.0043	0.0009	1.9E-06	22.8272
M00060.m	rs17107974	T	C	0.1068	-0.0082	0.0017	1.5E-06	23.2664

M00060.m	rs17173197	T	C	0.3872	-0.0044	0.0009	2.46E-06	23.9012
M00060.m	rs17238008	T	C	0.9385	-0.0115	0.0024	1.43E-06	22.9601
M00060.m	rs1733198	A	G	0.6308	0.0049	0.0009	7.62E-08	29.642
M00060.m	rs174476	T	C	0.4174	-0.0042	0.0009	5.7E-06	21.7778
M00060.m	rs1795964	A	G	0.4091	-0.0044	0.0009	1.98E-06	23.9012
M00060.m	rs1818106	A	C	0.501	0.005	0.001	5.73E-07	25
M00060.m	rs1822834	C	G	0.0629	0.0108	0.0024	6.59E-06	20.25
M00060.m	rs1862710	A	G	0.5489	0.0047	0.0009	1.17E-07	27.2716
M00060.m	rs1871053	A	G	0.4513	-0.005	0.0009	2.4E-08	30.8642
M00060.m	rs1872800	A	T	0.6438	-0.004	0.0009	8.25E-06	19.7531
M00060.m	rs1884639	T	C	0.3865	0.0047	0.0009	2.32E-07	27.2716
M00060.m	rs1931001	T	G	0.3503	-0.0043	0.0009	1.62E-06	22.8272
M00060.m	rs1952935	A	C	0.5806	-0.0048	0.0009	1.07E-07	28.4444
M00060.m	rs1971105	T	G	0.4305	-0.0041	0.0009	6.27E-06	20.7531
M00060.m	rs1995150	T	C	0.6102	0.004	0.0009	7.84E-06	19.7531
M00060.m	rs1999120	A	G	0.5692	-0.0041	0.0009	4.04E-06	20.7531
M00060.m	rs2057435	A	G	0.535	0.0041	0.0009	7.95E-06	20.7531
M00060.m	rs2060490	T	C	0.474	-0.0044	0.0009	9.1E-07	23.9012
M00060.m	rs2146498	A	G	0.5787	0.004	0.0009	8.04E-06	19.7531
M00060.m	rs2273875	C	G	0.37	0.0043	0.0009	1.82E-06	22.8272
M00060.m	rs2285137	T	C	0.9436	-0.0108	0.0024	5.35E-06	20.25
M00060.m	rs2302423	A	G	0.55	0.0042	0.0009	2.82E-06	21.7778
M00060.m	rs2357713	T	G	0.447	-0.0042	0.0009	3E-06	21.7778
M00060.m	rs2373642	A	G	0.0138	-0.0536	0.0117	4.72E-06	20.9874
M00060.m	rs242082	T	C	0.3867	0.0048	0.0009	1.38E-07	28.4444
M00060.m	rs2454043	A	C	0.588	-0.0047	0.0009	1.85E-07	27.2716
M00060.m	rs2465250	A	C	0.5503	0.0048	0.0009	7.76E-08	28.4444
M00060.m	rs2471650	T	C	0.5252	-0.0048	0.0009	1.16E-07	28.4444
M00060.m	rs2478832	T	C	0.3996	-0.0041	0.0009	8.05E-06	20.7531
M00060.m	rs248065	A	G	0.5081	-0.0048	0.0009	1.13E-07	28.4444
M00060.m	rs2513896	A	G	0.3526	0.0042	0.001	9.65E-06	17.64
M00060.m	rs2624695	T	C	0.5102	-0.004	0.0009	8.94E-06	19.7531
M00060.m	rs2667698	A	C	0.3681	0.0041	0.0009	7.07E-06	20.7531
M00060.m	rs2713737	A	G	0.44	-0.006	0.001	1.97E-09	36
M00060.m	rs2722276	A	G	0.523	0.0048	0.0009	8.7E-08	28.4444
M00060.m	rs2819322	T	C	0.4589	-0.0043	0.0009	1.79E-06	22.8272
M00060.m	rs2834578	T	C	0.3986	0.0045	0.0009	7.24E-07	25
M00060.m	rs2834803	A	G	0.5707	0.004	0.0009	8.13E-06	19.7531
M00060.m	rs2898645	T	C	0.545	-0.005	0.001	5.73E-07	25
M00060.m	rs2933284	T	C	0.3898	0.0042	0.0009	2.86E-06	21.7778
M00060.m	rs308780	T	C	0.4655	-0.0042	0.0009	2.86E-06	21.7778
M00060.m	rs3102519	A	G	0.375	0.0048	0.0009	7.83E-08	28.4444
M00060.m	rs348869	A	G	0.5108	-0.0043	0.0009	1.95E-06	22.8272
M00060.m	rs3775574	A	G	0.6047	-0.0044	0.0009	3.13E-06	23.9012
M00060.m	rs37959	T	C	0.3912	0.0044	0.0009	1.71E-06	23.9012
M00060.m	rs3802681	T	C	0.6144	0.0045	0.0009	5.44E-07	25
M00060.m	rs390836	A	T	0.415	0.0045	0.0009	8.06E-07	25
M00060.m	rs3924223	A	G	0.549	-0.0041	0.0009	6.18E-06	20.7531
M00060.m	rs4016189	C	G	0.4191	0.0045	0.0009	4.74E-07	25
M00060.m	rs4016189	C	G	0.4191	0.0045	0.0009	4.74E-07	25
M00060.m	rs4074961	T	C	0.4317	-0.0044	0.001	4.23E-06	19.36
M00060.m	rs4077470	T	C	0.5403	-0.0045	0.0009	4.65E-07	25
M00060.m	rs4148593	A	G	0.624	0.005	0.001	5.73E-07	25
M00060.m	rs417144	T	C	0.3507	0.0046	0.0009	2.57E-07	26.1235
M00060.m	rs4276770	A	G	0.3999	-0.0041	0.0009	5.11E-06	20.7531

M00060.m	rs4499783	T	C	0.5958	-0.004	0.0009	7.58E-06	19.7531
M00060.m	rs4531478	A	G	0.4713	0.0041	0.0009	4.45E-06	20.7531
M00060.m	rs4681618	A	G	0.5793	-0.0042	0.0009	3.15E-06	21.7778
M00060.m	rs4726308	T	G	0.3944	-0.0042	0.0009	3.35E-06	21.7778
M00060.m	rs4741887	A	G	0.4584	-0.0047	0.0009	1.68E-07	27.2716
M00060.m	rs4758270	A	G	0.6483	-0.0048	0.0009	2.01E-07	28.4444
M00060.m	rs4767788	A	G	0.4189	0.0047	0.0009	5.74E-07	27.2716
M00060.m	rs477992	A	G	0.3228	-0.0047	0.0009	2E-07	27.2716
M00060.m	rs4795552	A	G	0.3894	-0.0047	0.0009	1.55E-07	27.2716
M00060.m	rs481781	T	C	0.5409	0.0044	0.0009	9.14E-07	23.9012
M00060.m	rs4851946	A	G	0.5706	0.0042	0.0009	2.2E-06	21.7778
M00060.m	rs4875397	A	G	0.5599	-0.0042	0.0009	3.04E-06	21.7778
M00060.m	rs4889294	T	C	0.5449	-0.0041	0.0009	3.65E-06	20.7531
M00060.m	rs4920094	A	G	0.3912	0.004	0.0009	7.18E-06	19.7531
M00060.m	rs4921412	C	G	0.3864	-0.0042	0.0009	2.78E-06	21.7778
M00060.m	rs4935115	A	G	0.529	0.0045	0.0009	5.75E-07	25
M00060.m	rs4968791	A	G	0.5344	-0.0043	0.0009	1.48E-06	22.8272
M00060.m	rs6003916	T	C	0.8966	0.0087	0.0018	1.06E-06	23.3611
M00060.m	rs606798	T	G	0.3722	0.004	0.0009	9.9E-06	19.7531
M00060.m	rs6103894	T	G	0.5939	-0.0043	0.0009	2.71E-06	22.8272
M00060.m	rs618001	T	C	0.5857	0.0041	0.0009	4.18E-06	20.7531
M00060.m	rs623111	A	C	0.3678	0.0041	0.0009	6.38E-06	20.7531
M00060.m	rs631750	T	C	0.3544	0.0041	0.0009	7.51E-06	20.7531
M00060.m	rs637828	A	C	0.5424	0.0041	0.0009	5.81E-06	20.7531
M00060.m	rs6470194	A	G	0.406	0.005	0.001	5.73E-07	25
M00060.m	rs6475359	A	G	0.4127	-0.0046	0.0009	3.57E-07	26.1235
M00060.m	rs6518309	A	C	0.6195	0.0043	0.0009	1.78E-06	22.8272
M00060.m	rs6543342	T	C	0.3778	0.004	0.0009	8.56E-06	19.7531
M00060.m	rs6562776	A	G	0.6116	-0.0043	0.0009	1.65E-06	22.8272
M00060.m	rs6588636	T	C	0.611	-0.004	0.0009	9.94E-06	19.7531
M00060.m	rs6735596	A	C	0.6267	-0.0052	0.0009	1.15E-08	33.3827
M00060.m	rs6737109	T	C	0.5761	0.0051	0.0009	1.45E-08	32.1111
M00060.m	rs679563	A	G	0.4837	-0.0045	0.0009	7.13E-07	25
M00060.m	rs6820011	T	G	0.5152	-0.0051	0.0009	1.18E-08	32.1111
M00060.m	rs6820313	T	G	0.5169	0.0049	0.0009	4.65E-08	29.642
M00060.m	rs6822279	A	C	0.3969	-0.0041	0.0009	4.79E-06	20.7531
M00060.m	rs682359	T	G	0.5813	0.004	0.0009	6.34E-06	19.7531
M00060.m	rs6833720	A	G	0.4766	-0.0046	0.0009	2.22E-07	26.1235
M00060.m	rs697311	A	G	0.3792	0.0041	0.0009	4.58E-06	20.7531
M00060.m	rs6982408	T	G	0.4435	-0.0042	0.0009	2.32E-06	21.7778
M00060.m	rs699856	T	C	0.3661	0.004	0.0009	7.45E-06	19.7531
M00060.m	rs7073430	A	C	0.4008	0.004	0.0009	9.95E-06	19.7531
M00060.m	rs7121554	T	C	0.2573	-0.007	0.0016	6.31E-06	19.1406
M00060.m	rs7137008	A	G	0.4468	0.0046	0.0009	2.6E-07	26.1235
M00060.m	rs720293	A	G	0.4312	0.0043	0.0009	2.53E-06	22.8272
M00060.m	rs720625	A	G	0.6267	-0.0046	0.0009	4.66E-07	26.1235
M00060.m	rs720816	A	G	0.5425	0.004	0.0009	7.76E-06	19.7531
M00060.m	rs7301176	T	C	0.4603	0.0043	0.0009	1.86E-06	22.8272
M00060.m	rs7329192	T	C	0.5277	-0.0042	0.0009	3.17E-06	21.7778
M00060.m	rs7366464	T	C	0.1258	-0.0075	0.0017	7.78E-06	19.4637
M00060.m	rs7513382	T	G	0.5171	-0.004	0.0009	7.05E-06	19.7531
M00060.m	rs757378	A	T	0.6095	-0.0042	0.0009	3.57E-06	21.7778
M00060.m	rs7574695	T	C	0.4769	-0.0041	0.0009	5.97E-06	20.7531
M00060.m	rs7584322	A	C	0.4742	-0.0048	0.0009	7.03E-08	28.4444
M00060.m	rs7584842	T	C	0.5379	-0.0051	0.0009	1.89E-08	32.1111

M00060.m	rs759456	T	G	0.6263	0.0042	0.0009	3.73E-06	21.7778
M00060.m	rs7622687	A	G	0.4032	-0.0041	0.0009	5.21E-06	20.7531
M00060.m	rs7625991	A	C	0.345	-0.0042	0.0009	5.9E-06	21.7778
M00060.m	rs7647029	A	G	0.6479	0.0053	0.0009	1.02E-08	34.679
M00060.m	rs7669967	A	G	0.6167	0.004	0.0009	6.83E-06	19.7531
M00060.m	rs7685009	A	G	0.6582	-0.0045	0.0009	6.44E-07	25
M00060.m	rs7689825	T	C	0.643	0.0046	0.0009	3.97E-07	26.1235
M00060.m	rs769630	T	C	0.6016	0.0041	0.0009	5.06E-06	20.7531
M00060.m	rs7738860	T	C	0.4518	-0.0044	0.0009	1.31E-06	23.9012
M00060.m	rs7740614	A	G	0.4838	-0.0049	0.0009	4.75E-08	29.642
M00060.m	rs7795093	A	G	0.3813	-0.0044	0.0009	1.47E-06	23.9012
M00060.m	rs7825809	T	C	0.4319	-0.0047	0.0009	1.55E-07	27.2716
M00060.m	rs7911876	T	C	0.4276	0.0043	0.0009	2.76E-06	22.8272
M00060.m	rs7915	A	C	0.6374	-0.0049	0.0009	5.82E-08	29.642
M00060.m	rs7923885	A	G	0.5257	-0.0044	0.0009	8.08E-07	23.9012
M00060.m	rs7968823	T	C	0.4688	-0.004	0.0009	8.71E-06	19.7531
M00060.m	rs797348	A	G	0.6447	0.004	0.0009	9.67E-06	19.7531
M00060.m	rs7996613	T	C	0.4683	-0.004	0.0009	9.67E-06	19.7531
M00060.m	rs8003702	T	G	0.536	0.0042	0.0009	2.45E-06	21.7778
M00060.m	rs8012205	A	G	0.4966	-0.0042	0.0009	2.56E-06	21.7778
M00060.m	rs8023420	A	C	0.0972	-0.0076	0.0017	8.85E-06	19.9862
M00060.m	rs8131355	A	G	0.5488	-0.0041	0.0009	5.68E-06	20.7531
M00060.m	rs825885	A	G	0.1469	-0.0097	0.002	1.56E-06	23.5225
M00060.m	rs849327	A	G	0.3492	0.0042	0.0009	3.45E-06	21.7778
M00060.m	rs891878	T	G	0.4844	-0.0041	0.0009	6.03E-06	20.7531
M00060.m	rs899273	T	C	0.5202	0.0042	0.0009	3.33E-06	21.7778
M00060.m	rs900532	T	C	0.4524	-0.0044	0.0009	8.99E-07	23.9012
M00060.m	rs911981	A	G	0.5283	-0.0047	0.0009	2.4E-07	27.2716
M00060.m	rs923818	A	G	0.5072	0.0041	0.0009	6.23E-06	20.7531
M00060.m	rs9289408	T	C	0.4701	0.0047	0.0009	2.45E-07	27.2716
M00060.m	rs9292230	T	C	0.6218	0.0041	0.0009	4.63E-06	20.7531
M00060.m	rs9322464	T	C	0.47	0.004	0.0009	8.27E-06	19.7531
M00060.m	rs9369741	T	G	0.5822	0.0043	0.0009	2.42E-06	22.8272
M00060.m	rs9394683	T	C	0.5195	-0.004	0.0009	9.05E-06	19.7531
M00060.m	rs9403527	T	C	0.3456	0.0045	0.0009	9.91E-07	25
M00060.m	rs9541214	T	C	0.6529	-0.005	0.0009	3.4E-08	30.8642
M00060.m	rs979242	T	C	0.3692	-0.0043	0.0009	2.45E-06	22.8272
M00060.m	rs982790	T	C	0.4106	-0.0044	0.0009	3.74E-06	23.9012
M00060.m	rs9837954	T	C	0.6418	-0.0043	0.0009	2.64E-06	22.8272
M00060.m	rs9889080	A	G	0.1641	0.0074	0.0016	4.21E-06	21.3906
M00060.m	rs989960	T	C	0.4175	0.0041	0.0009	4.66E-06	20.7531
M00060.m	rs9903421	T	C	0.5633	-0.0041	0.0009	7.39E-06	20.7531
M00063.m	rs10011091	T	C	0.8159	0.0086	0.0018	2.15E-06	22.8272
M00063.m	rs1005596	A	G	0.1014	0.0115	0.0025	4.2E-06	21.16
M00063.m	rs10207315	T	G	0.2432	0.0078	0.0017	4.79E-06	21.0519
M00063.m	rs10493549	A	G	0.7989	-0.009	0.0017	2.27E-07	28.0277
M00063.m	rs1077834	T	C	0.7912	-0.0113	0.0023	6.87E-07	24.138
M00063.m	rs11022174	A	G	0.295	0.0101	0.0023	7.36E-06	19.2836
M00063.m	rs11106131	T	G	0.2379	-0.0089	0.0019	1.81E-06	21.9418
M00063.m	rs11111293	T	C	0.7914	-0.008	0.0018	6.12E-06	19.7531
M00063.m	rs1124515	A	G	0.2539	-0.0078	0.0017	6.01E-06	21.0519
M00063.m	rs11610936	T	C	0.0989	-0.0121	0.0025	1.34E-06	23.4256
M00063.m	rs11813145	A	G	0.3414	0.0076	0.0017	5.87E-06	19.9862
M00063.m	rs12648765	A	G	0.9861	0.0564	0.0122	3.57E-06	21.3717
M00063.m	rs12881791	T	G	0.7603	-0.0083	0.0018	5.91E-06	21.2623

M00063.m	rs12896813	T	C	0.1853	-0.0082	0.0018	3.52E-06	20.7531
M00063.m	rs1411983	A	T	0.594	-0.0073	0.0017	9.21E-06	18.4394
M00063.m	rs1433904	T	C	0.0228	0.0442	0.0097	5.06E-06	20.7635
M00063.m	rs1479402	T	C	0.0137	0.0306	0.0068	6.85E-06	20.25
M00063.m	rs17182784	T	C	0.253	-0.0082	0.0017	2.1E-06	23.2664
M00063.m	rs17293443	T	C	0.7718	0.0078	0.0017	8.09E-06	21.0519
M00063.m	rs17683567	T	C	0.2146	0.0078	0.0017	7.57E-06	21.0519
M00063.m	rs17728920	A	C	0.1768	0.0081	0.0018	5.3E-06	20.25
M00063.m	rs17765253	A	G	0.1845	0.0096	0.0018	8.27E-08	28.4444
M00063.m	rs2026590	T	G	0.2176	0.008	0.0017	3.76E-06	22.1453
M00063.m	rs2050223	A	G	0.2016	-0.0078	0.0017	7.22E-06	21.0519
M00063.m	rs2130712	A	C	0.92	0.0165	0.0032	2.43E-07	26.5869
M00063.m	rs2259571	T	G	0.6893	-0.0078	0.0017	2.86E-06	21.0519
M00063.m	rs2407893	A	G	0.427	0.0079	0.0016	1.29E-06	24.3789
M00063.m	rs2869804	A	G	0.2489	0.0078	0.0017	6.5E-06	21.0519
M00063.m	rs2870664	T	C	0.4767	0.0147	0.0031	1.6E-06	22.486
M00063.m	rs3747924	T	C	0.1781	0.0083	0.0018	4.24E-06	21.2623
M00063.m	rs3802195	T	G	0.1886	0.0086	0.0018	1.13E-06	22.8272
M00063.m	rs3869132	T	C	0.2494	0.0079	0.0017	4.06E-06	21.5952
M00063.m	rs3897960	A	T	0.2083	-0.0084	0.0017	1.29E-06	24.4152
M00063.m	rs4401492	T	G	0.1795	0.0082	0.0018	4.52E-06	20.7531
M00063.m	rs445925	A	G	0.079	-0.025	0.004	4.11E-10	39.0625
M00063.m	rs4602115	T	G	0.7133	0.0146	0.0033	7.65E-06	19.5739
M00063.m	rs4941743	T	C	0.2179	0.0087	0.0017	3.28E-07	26.1903
M00063.m	rs602633	T	G	0.2163	-0.0102	0.0017	5.02E-09	36
M00063.m	rs6438950	C	G	0.9036	0.0131	0.0025	1.54E-07	27.4576
M00063.m	rs6511720	T	G	0.1197	-0.0136	0.0027	3.69E-07	25.3717
M00063.m	rs6573766	T	G	0.8061	-0.0085	0.0018	1.55E-06	22.2994
M00063.m	rs6603141	A	G	0.258	-0.0081	0.0017	2.61E-06	22.7024
M00063.m	rs6970076	T	C	0.7679	-0.0079	0.0017	4.37E-06	21.5952
M00063.m	rs7595385	T	C	0.9759	0.0237	0.0051	3.83E-06	21.5952
M00063.m	rs810551	C	G	0.7904	0.008	0.0017	4.66E-06	22.1453
M00063.m	rs871748	T	C	0.182	-0.0082	0.0018	8.56E-06	20.7531
M00063.m	rs886282	T	C	0.1989	0.0081	0.0018	3.71E-06	20.25
M00063.m	rs927961	T	G	0.7597	-0.0079	0.0017	5.06E-06	21.5952
M00064.m	rs10050054	T	C	0.9828	-0.0325	0.0069	2.58E-06	22.1855
M00064.m	rs1498694	A	G	0.4035	0.0075	0.0011	7.34E-11	46.4876
M00064.m	rs1565225	A	G	0.245	0.007	0.0015	2.34E-06	21.7778
M00064.m	rs1722400	A	C	0.8799	0.01	0.0021	1.29E-06	22.6757
M00064.m	rs2905873	A	C	0.6858	0.0055	0.0012	2.97E-06	21.0069
M00064.m	rs6831907	T	C	0.0395	0.0206	0.0046	7.05E-06	20.0548
M00513.m	rs10426415	A	G	0.878	-0.0086	0.0018	3.11E-06	22.8272
M00513.m	rs10519445	A	G	0.5338	-0.0045	0.0009	1.85E-06	25
M00513.m	rs11054328	T	C	0.1072	0.0088	0.0018	1.46E-06	23.9012
M00513.m	rs1116866	T	C	0.1035	0.0094	0.0018	2.87E-07	27.2716
M00513.m	rs11638382	T	C	0.1875	-0.0171	0.0038	5.06E-06	20.25
M00513.m	rs11694230	T	C	0.3363	-0.0075	0.0017	9E-06	19.4637
M00513.m	rs12175103	A	C	0.4153	0.0048	0.0009	4.86E-07	28.4444
M00513.m	rs12324230	T	C	0.5209	0.0042	0.0009	8.68E-06	21.7778
M00513.m	rs12801550	T	C	0.8705	0.0088	0.0018	9.64E-07	23.9012
M00513.m	rs13031396	T	G	0.2473	-0.0076	0.0017	9.17E-06	19.9862
M00513.m	rs1374355	A	T	0.1077	0.0083	0.0019	8.59E-06	19.0831
M00513.m	rs1498350	A	C	0.4954	0.0045	0.001	1.88E-06	20.25
M00513.m	rs17533525	T	C	0.1534	0.0082	0.0018	5.04E-06	20.7531
M00513.m	rs1955135	A	C	0.8659	0.008	0.0018	9.99E-06	19.7531

M00513.m	rs1990178	T	C	0.1943	-0.0164	0.0036	6.27E-06	20.7531
M00513.m	rs2042499	A	C	0.4837	0.0052	0.0009	4.88E-08	33.3827
M00513.m	rs2048277	A	G	0.8956	0.0083	0.0019	8.75E-06	19.0831
M00513.m	rs2289804	A	G	0.9343	-0.0143	0.0027	1.25E-07	28.0508
M00513.m	rs2521310	T	C	0.874	0.009	0.002	6.8E-06	20.25
M00513.m	rs394282	A	T	0.1112	-0.0163	0.0033	1.02E-06	24.3976
M00513.m	rs4460629	T	C	0.5224	-0.0045	0.001	2.21E-06	20.25
M00513.m	rs6502247	A	G	0.119	-0.009	0.002	6.8E-06	20.25
M00513.m	rs6663614	A	C	0.2085	-0.0104	0.0022	3.06E-06	22.3471
M00513.m	rs9312777	T	C	0.4788	0.0043	0.0009	4.68E-06	22.8272
M00513.m	rs9456538	A	G	0.1493	-0.0082	0.0018	4.2E-06	20.7531
M00513.m	rs9477021	C	G	0.0178	-0.0236	0.0053	7.73E-06	19.8277
M00527.m	rs10788232	T	C	0.6825	-0.0111	0.0024	2.46E-06	21.3906
M00527.m	rs1193345	A	G	0.9357	-0.0216	0.0045	1.43E-06	23.04
M00527.m	rs1330523	A	G	0.2326	-0.0117	0.0024	1.56E-06	23.7656
M00527.m	rs13403441	A	T	0.8588	-0.0235	0.0048	1.02E-06	23.9692
M00527.m	rs16937124	A	C	0.9471	-0.021	0.0046	5.62E-06	20.8412
M00527.m	rs1876100	A	T	0.2626	-0.0106	0.0024	9.2E-06	19.5069
M00527.m	rs2000812	T	C	0.8223	0.0111	0.0025	9.7E-06	19.7136
M00527.m	rs3785024	A	G	0.9469	-0.0223	0.0049	5.56E-06	20.7118
M00527.m	rs4635489	A	G	0.0177	0.0364	0.008	5.66E-06	20.7025
M00527.m	rs4718532	T	C	0.5052	-0.016	0.0035	6.61E-06	20.898
M00527.m	rs723526	A	G	0.1994	-0.011	0.0024	6.62E-06	21.0069
M00527.m	rs780093	T	C	0.4	0.0117	0.0023	1.89E-07	25.8771
M00542.m	rs11224003	A	G	0.177	0.0419	0.0093	7.07E-06	20.2984
M00542.m	rs11719883	C	G	0.8445	-0.0524	0.0103	3.86E-07	25.8814
M00542.m	rs13288937	A	C	0.0205	0.2488	0.0526	2.29E-06	22.3733
M00542.m	rs2295758	T	C	0.9754	-0.1137	0.0255	8.57E-06	19.8811
M00542.m	rs2711506	A	G	0.5343	-0.0326	0.0072	6.35E-06	20.5008
M00542.m	rs4297577	T	C	0.0384	0.0977	0.0209	2.94E-06	21.8523
M00542.m	rs4838537	T	C	0.0133	0.3226	0.0699	3.96E-06	21.2997
M00542.m	rs6493912	T	C	0.7431	-0.0368	0.0081	5.36E-06	20.6408
M00542.m	rs7314742	T	G	0.2662	-0.0358	0.008	8.51E-06	20.0256
M00542.m	rs8000457	T	C	0.9661	-0.2093	0.0433	1.35E-06	23.3648
M00553.x	rs1176413	T	C	0.1873	-0.1032	0.0219	2.55E-06	22.206
M00553.x	rs12440014	C	G	0.7673	0.1008	0.0213	2.18E-06	22.3956
M00553.x	rs1556938	T	G	0.0466	-0.1815	0.041	9.59E-06	19.5968
M00553.x	rs1605457	T	C	0.9643	0.3731	0.08	3.1E-06	21.7506
M00553.x	rs16830773	C	G	0.0197	-0.5244	0.1007	1.92E-07	27.1185
M00553.x	rs17800513	T	G	0.1085	-0.1308	0.0273	1.58E-06	22.9557
M00553.x	rs1818902	T	C	0.9701	0.5718	0.1255	5.21E-06	20.7587
M00553.x	rs2075317	T	C	0.5417	0.0732	0.0165	9.19E-06	19.6813
M00553.x	rs2211372	T	C	0.985	0.3317	0.0696	1.87E-06	22.7129
M00553.x	rs2234233	A	G	0.135	-0.1234	0.0248	6.38E-07	24.7586
M00553.x	rs6468437	T	C	0.8334	0.1246	0.0277	6.86E-06	20.2338
M00553.x	rs6816298	A	G	0.8254	0.1842	0.0403	4.76E-06	20.8915
M00553.x	rs7359970	C	G	0.8975	0.2147	0.0451	1.93E-06	22.6627
M00553.x	rs7482087	T	C	0.0254	-0.5162	0.1064	1.24E-06	23.5371
M00553.x	rs7701941	C	G	0.0194	-0.5561	0.1115	6.1E-07	24.8746
M00553.x	rs9614985	A	G	0.4177	-0.0855	0.0177	1.28E-06	23.3338
M00569.x	rs1039293	A	G	0.4948	0.0709	0.0153	3.39E-06	21.4738
M00569.x	rs10935899	C	G	0.8197	-0.0506	0.011	4.54E-06	21.16
M00569.x	rs1397829	A	G	0.5328	0.042	0.0085	7.08E-07	24.4152
M00569.x	rs16902976	T	G	0.0232	-0.1325	0.0294	6.36E-06	20.3113
M00569.x	rs2446427	T	C	0.8371	-0.0554	0.0122	5.21E-06	20.6205

M00569.x	rs2472297	T	C	0.2362	-0.0668	0.0104	1.5E-10	41.2559
M00569.x	rs2493277	C	G	0.6552	0.0605	0.0127	1.91E-06	22.6936
M00569.x	rs28393181	T	C	0.7961	-0.0927	0.0184	4.71E-07	25.3819
M00569.x	rs384814	A	G	0.0255	0.1452	0.0326	8.35E-06	19.838
M00569.x	rs6603123	T	G	0.3939	0.0865	0.0193	7.84E-06	20.0871
M00569.x	rs6968554	A	G	0.3756	0.0644	0.0086	8.68E-14	56.0757
M00569.x	rs8096497	A	G	0.097	0.089	0.0163	4.84E-08	29.8129
M00569.x	rs946712	A	C	0.9559	0.1039	0.0212	9.59E-07	24.0192
M00575.m	rs12091176	A	G	0.0264	0.127	0.0271	2.81E-06	21.9618
M00575.m	rs12426131	A	G	0.1446	0.0246	0.0051	1.68E-06	23.2664
M00575.m	rs33976862	C	G	0.0674	0.0602	0.0135	7.76E-06	19.885
M00575.m	rs526744	T	C	0.243	-0.021	0.0042	7.14E-07	25
M00575.m	rs7790791	T	C	0.0873	0.0594	0.013	4.67E-06	20.8779
M00577.m	rs2456584	A	G	0.5393	-0.0179	0.0037	1.64E-06	23.4047
M00577.m	rs4663119	A	G	0.0773	0.0331	0.0074	7.32E-06	20.0075
M00577.m	rs661454	A	G	0.0848	0.0317	0.0065	1.31E-06	23.7844
M00584.m	rs10736	T	C	0.2011	-0.0118	0.0026	4.73E-06	20.5976
M00584.m	rs10982244	A	G	0.6098	-0.0177	0.004	8.93E-06	19.5806
M00584.m	rs11676911	A	G	0.0216	-0.0429	0.0084	3.66E-07	26.0829
M00584.m	rs1181723	A	G	0.2086	0.0116	0.0026	5.63E-06	19.9053
M00584.m	rs12218544	C	G	0.0836	-0.019	0.0039	1.28E-06	23.7344
M00584.m	rs12414366	C	G	0.7696	0.0214	0.0047	4.89E-06	20.7316
M00584.m	rs12474099	A	G	0.0649	-0.0202	0.0043	1.97E-06	22.0681
M00584.m	rs1260326	T	C	0.4088	-0.0439	0.0024	1.3E-77	334.585
M00584.m	rs13016086	T	C	0.8778	-0.0162	0.0033	1.09E-06	24.0992
M00584.m	rs13116473	A	G	0.9853	-0.0448	0.0096	2.93E-06	21.7778
M00584.m	rs16932803	T	G	0.1673	-0.0121	0.0026	5.01E-06	21.6583
M00584.m	rs17061914	A	G	0.8581	-0.0146	0.0033	8.77E-06	19.5739
M00584.m	rs17706100	T	G	0.9553	0.0246	0.0051	1.41E-06	23.2664
M00584.m	rs1841535	T	G	0.1482	0.0252	0.0056	7E-06	20.25
M00584.m	rs2141371	A	G	0.682	-0.0198	0.0024	3.27E-16	68.0625
M00584.m	rs2270451	A	G	0.8941	0.0152	0.0034	7.53E-06	19.9862
M00584.m	rs2272172	T	C	0.0226	-0.0648	0.0145	7.51E-06	19.9717
M00584.m	rs2289017	T	C	0.6562	0.0113	0.0025	4.11E-06	20.4304
M00584.m	rs2304684	T	C	0.0182	-0.0438	0.0094	3.02E-06	21.7116
M00584.m	rs2723713	T	C	0.0254	-0.0356	0.008	9.19E-06	19.8025
M00584.m	rs435315	A	T	0.2033	-0.0121	0.0026	2.18E-06	21.6583
M00584.m	rs6547811	A	T	0.1583	0.0222	0.0032	7.1E-12	48.1289
M00584.m	rs7007251	A	G	0.1057	-0.015	0.0034	8.76E-06	19.4637
M00584.m	rs7349418	T	C	0.5484	0.0154	0.0024	1.85E-10	41.1736
M00584.m	rs7583698	T	C	0.0483	0.0338	0.0049	8.55E-12	47.5818
M00584.m	rs7596471	A	C	0.3083	0.0137	0.0025	7.47E-08	30.0304
M00584.m	rs7747345	A	T	0.8153	0.0122	0.0026	2.52E-06	22.0178
M00584.m	rs937813	T	C	0.9025	0.0271	0.0039	2.65E-12	48.2847
M00584.m	rs991670	T	C	0.6624	-0.0111	0.0025	7.48E-06	19.7136
M00599.m	rs10237319	T	C	0.1467	0.0226	0.0051	9.79E-06	19.6371
M00599.m	rs11038739	A	T	0.7937	-0.0203	0.0043	3.01E-06	22.2872
M00599.m	rs11216267	T	C	0.6435	0.0168	0.0035	1.56E-06	23.04
M00599.m	rs11259923	A	G	0.8139	0.042	0.0091	3.92E-06	21.3018
M00599.m	rs1133034	T	C	0.0757	0.032	0.0069	3.91E-06	21.5081
M00599.m	rs12515830	T	G	0.8744	0.0244	0.0054	5.79E-06	20.417
M00599.m	rs13195863	A	C	0.2043	0.022	0.0044	4.13E-07	25
M00599.m	rs2833657	A	G	0.8254	0.0239	0.0051	3.02E-06	21.9612
M00599.m	rs2964063	T	C	0.9853	-0.0735	0.0155	2.25E-06	22.486
M00599.m	rs3925033	T	G	0.4591	-0.0164	0.0035	2.59E-06	21.9559

M00599.m	rs4648464	A	G	0.5522	-0.0161	0.0036	9.37E-06	20.0008
M00599.m	rs6895278	T	C	0.3354	0.0168	0.0037	4.13E-06	20.6165
M00599.m	rs7941871	T	G	0.8353	-0.024	0.005	1.56E-06	23.04
M00599.m	rs924483	A	G	0.0916	-0.0289	0.0062	3.25E-06	21.7276
M00606.m	rs1007797	T	C	0.879	-0.0092	0.0018	6.49E-07	26.1235
M00606.m	rs11033504	A	G	0.9307	0.0143	0.0027	9.46E-08	28.0508
M00606.m	rs11607704	T	C	0.8783	0.0093	0.0019	6.94E-07	23.9584
M00606.m	rs11705028	A	G	0.0859	-0.0206	0.0046	8.85E-06	20.0548
M00606.m	rs12524738	A	G	0.069	0.0156	0.0035	6.93E-06	19.8661
M00606.m	rs12544733	C	G	0.8378	-0.0084	0.0018	2.74E-06	21.7778
M00606.m	rs12580704	T	C	0.1328	0.0085	0.0018	4.55E-06	22.2994
M00606.m	rs131794	A	C	0.2074	0.0146	0.0018	4.37E-16	65.7901
M00606.m	rs1482928	T	G	0.7912	0.0081	0.0018	4.42E-06	20.25
M00606.m	rs163425	A	G	0.8734	0.0086	0.0018	2.62E-06	22.8272
M00606.m	rs2686789	A	C	0.1304	-0.0092	0.0018	5.32E-07	26.1235
M00606.m	rs2686796	T	C	0.4524	0.0094	0.0017	1.89E-08	30.5744
M00606.m	rs2904665	A	G	0.861	-0.0089	0.0018	9.19E-07	24.4475
M00606.m	rs2982907	T	G	0.1465	-0.0088	0.0018	1.28E-06	23.9012
M00606.m	rs4286846	C	G	0.7232	0.0083	0.0017	1.26E-06	23.8374
M00606.m	rs532545	T	C	0.3065	-0.0095	0.0017	3.41E-08	31.2284
M00606.m	rs6988242	T	G	0.769	0.0094	0.0018	1.31E-07	27.2716
M00606.m	rs7593385	A	C	0.9685	0.0359	0.008	6.87E-06	20.1377
M00606.m	rs787496	A	G	0.1702	0.0081	0.0018	5.98E-06	20.25
M00606.m	rs7948201	T	C	0.9623	-0.023	0.0046	6.77E-07	25
M00606.m	rs9601054	T	C	0.162	0.0083	0.0018	4.24E-06	21.2623
M00606.m	rs977712	A	C	0.8633	-0.0085	0.0019	6.32E-06	20.0139
M01105.m	rs11719883	C	G	0.8444	-0.0174	0.0034	3.57E-07	26.1903
M01105.m	rs12238083	T	C	0.1389	0.0152	0.0033	3.01E-06	21.2158
M01105.m	rs12336893	T	G	0.9588	0.0289	0.0062	2.68E-06	21.7276
M01105.m	rs13225022	A	G	0.6784	-0.0123	0.0026	1.4E-06	22.3802
M01105.m	rs13280152	T	G	0.1202	0.0162	0.0033	1.22E-06	24.0992
M01105.m	rs1477025	T	C	0.6545	0.0109	0.0024	7.63E-06	20.6267
M01105.m	rs1502520	T	G	0.7334	0.0119	0.0025	1.63E-06	22.6576
M01105.m	rs16911296	A	G	0.0771	-0.0209	0.0045	4.18E-06	21.5709
M01105.m	rs17087487	A	G	0.053	-0.0231	0.0052	8E-06	19.7341
M01105.m	rs1956129	T	C	0.7589	-0.0127	0.0025	3.66E-07	25.8064
M01105.m	rs2158555	C	G	0.0331	-0.0337	0.0075	7.91E-06	20.19
M01105.m	rs2242415	A	C	0.6233	0.0109	0.0024	8.24E-06	20.6267
M01105.m	rs4654501	A	G	0.7671	0.0111	0.0025	9.23E-06	19.7136
M01105.m	rs6075489	A	G	0.7177	0.0113	0.0025	9.51E-06	20.4304
M01105.m	rs7014120	A	G	0.1341	0.0148	0.0033	5.67E-06	20.1139
M01105.m	rs7115681	T	C	0.4668	-0.0131	0.0024	8.29E-08	29.7934
M01105.m	rs7898173	A	G	0.1419	-0.0143	0.0032	6.81E-06	19.9697
M01107.m	rs10492266	T	C	0.4255	-0.0167	0.0038	8.78E-06	19.3137
M01107.m	rs10771223	A	G	0.78	0.0237	0.0046	2.87E-07	26.5449
M01107.m	rs11147404	T	G	0.8872	0.0282	0.0063	8.69E-06	20.0363
M01107.m	rs12132798	C	G	0.9749	0.0615	0.013	2.09E-06	22.3802
M01107.m	rs12189555	A	T	0.7684	0.021	0.0046	5.06E-06	20.8412
M01107.m	rs12718598	T	C	0.5178	0.018	0.0037	1.49E-06	23.6669
M01107.m	rs1320934	A	T	0.9599	0.1215	0.0253	1.51E-06	23.0628
M01107.m	rs1431793	A	C	0.7526	-0.0216	0.0046	2.9E-06	22.0491
M01107.m	rs1447307	T	G	0.227	-0.0211	0.0046	5.69E-06	21.0402
M01107.m	rs1499094	T	G	0.7686	0.0208	0.0046	7.68E-06	20.4461
M01107.m	rs167277	A	C	0.781	-0.0207	0.0046	7.92E-06	20.25
M01107.m	rs1799412	C	G	0.8394	-0.0262	0.0055	2.28E-06	22.6922

M01107.m	rs2293319	T	C	0.7852	-0.0219	0.0047	3.59E-06	21.7116
M01107.m	rs2549842	T	C	0.786	-0.0206	0.0046	8.68E-06	20.0548
M01107.m	rs4579742	A	T	0.1251	0.029	0.0064	5.16E-06	20.5322
M01107.m	rs8108144	T	G	0.788	-0.0232	0.0047	6.44E-07	24.3658
M01107.m	rs8184921	T	C	0.5087	0.0165	0.0037	9.8E-06	19.8868
M01107.m	rs972709	A	C	0.6548	0.0483	0.0107	6.73E-06	20.3764
M01107.m	rs9834988	T	C	0.5297	0.0185	0.0038	7.98E-07	23.7015
M01110.m	rs1010069	A	G	0.4623	0.0081	0.0018	8.58E-06	20.25
M01110.m	rs10241964	A	G	0.0952	0.0182	0.0035	2.25E-07	27.04
M01110.m	rs10514491	T	C	0.6782	0.012	0.0026	2.86E-06	21.3018
M01110.m	rs11837423	A	G	0.088	0.0385	0.008	1.52E-06	23.1602
M01110.m	rs12352829	A	G	0.1506	-0.0134	0.0027	5.84E-07	24.631
M01110.m	rs12532113	T	C	0.961	-0.028	0.006	3.06E-06	21.7778
M01110.m	rs1430209	T	C	0.5313	-0.0086	0.0018	1.87E-06	22.8272
M01110.m	rs17099239	A	G	0.8327	-0.012	0.0027	6.74E-06	19.7531
M01110.m	rs174548	C	G	0.7005	0.0488	0.0025	1.43E-84	381.03
M01110.m	rs174602	T	C	0.8702	0.0353	0.004	2.42E-18	77.8806
M01110.m	rs17764324	T	G	0.086	-0.0241	0.0041	3.31E-09	34.5515
M01110.m	rs1800009	T	C	0.6444	0.0125	0.0025	4.91E-07	25
M01110.m	rs180022	T	C	0.8328	0.0117	0.0026	9.43E-06	20.25
M01110.m	rs198476	A	G	0.482	0.0122	0.0024	5.14E-07	25.8403
M01110.m	rs2275351	T	C	0.1657	0.012	0.0027	7.06E-06	19.7531
M01110.m	rs2727261	T	C	0.09	-0.0189	0.0037	3.44E-07	26.0928
M01110.m	rs3798211	A	C	0.4247	-0.0083	0.0018	4.58E-06	21.2623
M01110.m	rs412334	T	C	0.157	0.022	0.003	2.25E-13	53.7778
M01110.m	rs472031	A	G	0.0883	0.0175	0.0035	4.43E-07	25
M01110.m	rs4739402	T	C	0.5172	-0.0085	0.0019	5.61E-06	20.0139
M01110.m	rs4982495	T	C	0.4275	-0.0084	0.0018	3.27E-06	21.7778
M01110.m	rs526126	C	G	0.8409	0.0249	0.0039	2.11E-10	40.7633
M01110.m	rs532098	A	G	0.461	0.009	0.002	6.8E-06	20.25
M01110.m	rs6535974	A	G	0.8465	-0.0122	0.0027	5.8E-06	20.417
M01110.m	rs6568389	T	C	0.8061	0.0157	0.0033	2.16E-06	22.6345
M01110.m	rs712916	T	C	0.9875	-0.0456	0.0102	8.19E-06	19.9862
M01110.m	rs7169869	A	G	0.5404	-0.0194	0.0043	6.59E-06	20.3548
M01110.m	rs741886	T	G	0.0829	-0.0187	0.0036	1.85E-07	26.9823
M01110.m	rs8123073	A	G	0.983	-0.0372	0.0082	5.94E-06	20.5806
M01110.m	rs9679675	A	C	0.4732	0.0092	0.0018	4.17E-07	26.1235
M01114.m	rs11159894	T	C	0.3295	0.032	0.0067	1.77E-06	22.8113
M01114.m	rs12405017	T	C	0.9817	-0.1891	0.0426	9.18E-06	19.7044
M01114.m	rs17131011	A	G	0.0404	-0.0781	0.0174	6.88E-06	20.1467
M01114.m	rs17175231	A	G	0.9348	0.0593	0.0125	1.99E-06	22.5055
M01114.m	rs17641342	C	G	0.1639	0.0725	0.0159	5.26E-06	20.7913
M01114.m	rs2002212	A	G	0.049	-0.0691	0.0155	7.82E-06	19.8743
M01114.m	rs2140798	T	C	0.0986	-0.0519	0.0117	8.8E-06	19.6772
M01114.m	rs2391088	A	G	0.6873	-0.0341	0.0073	2.92E-06	21.8204
M01114.m	rs3752241	C	G	0.8684	0.0574	0.0126	5.52E-06	20.7531
M01114.m	rs4244808	T	G	0.5307	0.0305	0.0068	8.3E-06	20.1179
M01114.m	rs4579412	A	C	0.1469	-0.0414	0.0091	4.77E-06	20.6975
M01114.m	rs696155	A	G	0.1888	-0.0412	0.0088	2.92E-06	21.9194
M01114.m	rs7262824	T	C	0.2062	0.0357	0.008	8.7E-06	19.9139
M01114.m	rs7269193	A	G	0.9092	-0.0528	0.0116	5.03E-06	20.7182
M01114.m	rs7427777	A	C	0.0483	0.1291	0.0286	6.36E-06	20.3761
M01114.m	rs8192870	T	G	0.3498	-0.0365	0.0067	3.99E-08	29.6781
M01114.m	rs9851393	A	G	0.5199	0.0297	0.0066	7.38E-06	20.25
M01121.m	rs11118120	A	C	0.8501	-0.019	0.004	1.97E-06	22.5625

M01121.m	rs11940037	T	C	0.842	0.019	0.0041	2.82E-06	21.4753
M01121.m	rs1466228	T	C	0.0348	-0.0361	0.0078	3.21E-06	21.4203
M01121.m	rs17351329	A	G	0.8651	-0.0185	0.0041	5.37E-06	20.3599
M01121.m	rs1822906	A	G	0.2048	0.018	0.0034	9.61E-08	28.0277
M01121.m	rs4470674	A	T	0.7182	-0.0141	0.0031	6.64E-06	20.6878
M01121.m	rs603424	A	G	0.1865	0.0292	0.0038	1.56E-14	59.0471
M01121.m	rs7583745	T	C	0.884	-0.0348	0.0075	3.06E-06	21.5296
M01123.m	rs10489	A	C	0.0243	0.1743	0.038	4.6E-06	21.0391
M01123.m	rs11616655	A	G	0.9444	-0.1069	0.0237	6.37E-06	20.345
M01123.m	rs1415753	T	C	0.3236	-0.0551	0.0111	7.6E-07	24.6409
M01123.m	rs17077586	A	G	0.9187	0.1032	0.021	9.2E-07	24.1502
M01123.m	rs17224277	A	G	0.0778	0.0951	0.0199	1.83E-06	22.8378
M01123.m	rs17741813	T	C	0.015	0.538	0.1192	6.39E-06	20.371
M01123.m	rs2846723	T	G	0.5442	-0.0476	0.0105	5.76E-06	20.5511
M01123.m	rs4384764	A	G	0.2371	-0.0568	0.0127	7.9E-06	20.0027
M01123.m	rs494562	A	G	0.8988	-0.1182	0.0167	1.34E-12	50.0959
M01123.m	rs7297956	C	G	0.7301	0.0597	0.0134	7.69E-06	19.849
M01123.m	rs7795118	T	C	0.2305	0.0905	0.0186	1.21E-06	23.674
M01123.m	rs9359674	A	T	0.0565	0.1142	0.0218	1.57E-07	27.4422
M01123.m	rs9450282	A	G	0.3626	-0.0717	0.0108	2.74E-11	44.0748
M01125.m	rs10024717	A	G	0.3772	0.0085	0.0016	7.5E-08	28.2227
M01125.m	rs10063205	T	C	0.1559	0.008	0.0017	4.06E-06	22.1453
M01125.m	rs11128879	T	C	0.1208	-0.0078	0.0018	9.56E-06	18.7778
M01125.m	rs12707434	A	G	0.9639	-0.0366	0.008	4.65E-06	20.9306
M01125.m	rs161503	T	C	0.1176	0.008	0.0018	6.87E-06	19.7531
M01125.m	rs1618996	A	G	0.1298	0.0091	0.0018	3.78E-07	25.5586
M01125.m	rs1895999	A	G	0.1699	-0.0151	0.0032	3.43E-06	22.2666
M01125.m	rs2397972	A	G	0.054	0.012	0.0026	4.05E-06	21.3018
M01125.m	rs2470439	A	G	0.11	0.0083	0.0018	5.69E-06	21.2623
M01125.m	rs3760605	T	C	0.8912	0.0079	0.0018	9.8E-06	19.2623
M01125.m	rs4086666	T	G	0.8804	0.0083	0.0018	2.42E-06	21.2623
M01125.m	rs4347830	A	G	0.337	0.01	0.002	5.73E-07	25
M01125.m	rs4562384	T	C	0.7166	0.0075	0.0017	5.5E-06	19.4637
M01125.m	rs503360	A	G	0.8799	0.0081	0.0018	4.79E-06	20.25
M01125.m	rs6084839	A	C	0.9446	-0.0123	0.0027	3.99E-06	20.7531
M01125.m	rs6101153	A	G	0.8897	-0.008	0.0018	8.26E-06	19.7531
M01125.m	rs7340968	A	C	0.027	0.0333	0.0075	9.05E-06	19.7136
M01125.m	rs7530270	A	C	0.2	-0.0085	0.0018	1.51E-06	22.2994
M01284.m	rs10234936	A	G	0.035	0.028	0.0061	4.88E-06	21.0696
M01284.m	rs1043011	T	G	0.1722	0.0172	0.0034	5.21E-07	25.5917
M01284.m	rs10433674	T	C	0.9584	0.0264	0.0054	1.24E-06	23.9012
M01284.m	rs10795360	C	G	0.6957	0.0114	0.0025	3.76E-06	20.7936
M01284.m	rs10798574	T	G	0.2212	-0.0142	0.003	2.03E-06	22.4044
M01284.m	rs10993160	A	G	0.9587	0.0295	0.0062	1.68E-06	22.6392
M01284.m	rs13242474	A	G	0.7395	0.0111	0.0024	5.47E-06	21.3906
M01284.m	rs1466785	T	C	0.4409	-0.0106	0.0024	5.93E-06	19.5069
M01284.m	rs17092955	T	G	0.1024	-0.0175	0.0034	3.39E-07	26.4922
M01284.m	rs17268257	T	C	0.1349	-0.0159	0.0035	7.31E-06	20.6376
M01284.m	rs202490	T	C	0.3533	-0.0111	0.0024	4.45E-06	21.3906
M01284.m	rs3857856	A	G	0.8885	0.0152	0.0033	4.93E-06	21.2158
M01284.m	rs42041	C	G	0.7449	-0.0109	0.0025	9.33E-06	19.0096
M01284.m	rs4516857	C	G	0.0991	0.0205	0.0045	4.93E-06	20.7531
M01284.m	rs4822129	A	G	0.194	0.0122	0.0026	3.76E-06	22.0178
M01284.m	rs715	T	C	0.7125	0.0113	0.0024	4.19E-06	22.1684
M01284.m	rs7220150	A	C	0.8227	0.0147	0.0033	6.71E-06	19.843

M01284.m	rs780093	T	C	0.3998	-0.0108	0.0024	3.98E-06	20.25
M01284.m	rs892051	T	C	0.2576	0.011	0.0025	7.67E-06	19.36
M01284.m	rs9403794	A	C	0.5963	0.0175	0.0039	8.09E-06	20.1348
M01299.m	rs10033622	T	C	0.6115	0.0126	0.0026	1.35E-06	23.4852
M01299.m	rs10516469	T	C	0.1	-0.0114	0.0025	5.89E-06	20.7936
M01299.m	rs10873157	T	C	0.0933	-0.0109	0.0024	8.62E-06	20.6267
M01299.m	rs11243929	T	C	0.7731	-0.0076	0.0017	8.33E-06	19.9862
M01299.m	rs12728678	T	C	0.2003	-0.0096	0.0017	2.19E-08	31.8893
M01299.m	rs12891	C	G	0.4177	0.0078	0.0018	8.78E-06	18.7778
M01299.m	rs13237436	A	G	0.193	-0.0076	0.0017	7.26E-06	19.9862
M01299.m	rs1475943	T	C	0.2215	0.0079	0.0017	3.69E-06	21.5952
M01299.m	rs1512342	T	C	0.3384	0.0072	0.0016	7.01E-06	20.25
M01299.m	rs1532775	A	G	0.7796	0.0081	0.0017	1.04E-06	22.7024
M01299.m	rs1536564	T	C	0.2644	0.0075	0.0017	7.05E-06	19.4637
M01299.m	rs172650	A	C	0.6543	0.0102	0.0016	2.82E-10	40.6406
M01299.m	rs17293820	T	G	0.8016	0.0081	0.0017	2.01E-06	22.7024
M01299.m	rs174408	A	G	0.149	0.0129	0.0023	1.41E-08	31.4575
M01299.m	rs1851740	A	C	0.185	-0.008	0.0017	2.74E-06	22.1453
M01299.m	rs2879560	T	C	0.2226	-0.0081	0.0017	3.48E-06	22.7024
M01299.m	rs2891733	T	C	0.8941	-0.0109	0.0024	8.47E-06	20.6267
M01299.m	rs4347830	A	G	0.337	0.009	0.002	6.8E-06	20.25
M01299.m	rs4363868	A	G	0.3182	0.0073	0.0016	7.59E-06	20.8164
M01299.m	rs4808739	T	C	0.7578	0.0083	0.0018	2.42E-06	21.2623
M01299.m	rs4863214	T	C	0.7398	-0.0149	0.0032	3.98E-06	21.6807
M01299.m	rs6493	A	G	0.1195	0.0114	0.0024	2.35E-06	22.5625
M01299.m	rs703014	A	G	0.326	0.0135	0.0027	7.19E-07	25
M01299.m	rs7151615	T	G	0.273	-0.0079	0.0016	1.47E-06	24.3789
M01299.m	rs7195332	T	C	0.2152	0.0077	0.0017	4.05E-06	20.5156
M01299.m	rs7641634	A	G	0.266	-0.0077	0.0016	2.56E-06	23.1602
M01299.m	rs7834337	A	G	0.0904	-0.0113	0.0025	4.71E-06	20.4304
M01299.m	rs8035304	T	G	0.81	0.009	0.002	6.8E-06	20.25
M01299.m	rs8111703	A	T	0.2142	-0.0084	0.0017	1.47E-06	24.4152
M01299.m	rs906466	A	G	0.1846	0.0076	0.0017	8.84E-06	19.9862
M01299.m	rs9327433	T	G	0.5134	-0.0076	0.0016	2.53E-06	22.5625
M01299.m	rs936632	A	C	0.1787	-0.0089	0.0017	2.69E-07	27.4083
M01299.m	rs9400467	T	C	0.7059	-0.0121	0.0016	6.54E-14	57.1914
M01299.m	rs9497139	A	G	0.0155	-0.029	0.0061	1.97E-06	22.6015
M01299.m	rs9594421	A	T	0.2757	0.0075	0.0016	4.59E-06	21.9727
M01301.m	rs10095414	A	G	0.8619	0.0086	0.0018	2.29E-06	22.8272
M01301.m	rs10491431	A	C	0.1435	0.0082	0.0018	3.8E-06	20.7531
M01301.m	rs10738383	A	G	0.6175	-0.0131	0.0029	7.39E-06	20.4055
M01301.m	rs10850904	T	C	0.127	0.0085	0.0018	2.74E-06	22.2994
M01301.m	rs10917910	A	C	0.8597	-0.0086	0.0018	1.65E-06	22.8272
M01301.m	rs11151130	A	G	0.1379	0.0085	0.0018	1.79E-06	22.2994
M01301.m	rs11600689	A	T	0.1616	-0.0103	0.0023	9.14E-06	20.0548
M01301.m	rs11659807	C	G	0.1964	-0.0082	0.0017	2.16E-06	23.2664
M01301.m	rs1179979	A	G	0.9214	-0.0128	0.0026	9.49E-07	24.2367
M01301.m	rs12268257	T	C	0.1339	-0.0082	0.0018	5.59E-06	20.7531
M01301.m	rs12927959	A	G	0.7248	0.0089	0.0018	3.76E-07	24.4475
M01301.m	rs13302218	A	G	0.932	-0.0118	0.0026	6.8E-06	20.5976
M01301.m	rs13339263	A	G	0.8587	-0.0081	0.0018	6.52E-06	20.25
M01301.m	rs1402325	A	G	0.1437	-0.008	0.0018	8.23E-06	19.7531
M01301.m	rs1573544	T	C	0.1484	0.0087	0.0018	9.64E-07	23.3611
M01301.m	rs1894947	T	C	0.1801	0.0083	0.0017	1.85E-06	23.8374
M01301.m	rs2383208	A	G	0.8223	0.0078	0.0017	8.79E-06	21.0519

M01301.m	rs2863979	A	G	0.7241	0.0143	0.0017	1.44E-17	70.7578
M01301.m	rs3024536	T	C	0.1443	-0.0085	0.0018	1.69E-06	22.2994
M01301.m	rs4317453	A	G	0.3363	-0.0077	0.0017	9.12E-06	20.5156
M01301.m	rs4426541	A	G	0.2229	-0.0079	0.0017	5.4E-06	21.5952
M01301.m	rs6587731	T	G	0.1626	-0.0093	0.0018	1.96E-07	26.6944
M01301.m	rs7572149	T	C	0.133	0.0088	0.0018	1E-06	23.9012
M01301.m	rs7583413	T	C	0.8245	0.0082	0.0017	2.62E-06	23.2664
M01301.m	rs9642758	A	G	0.1578	0.0086	0.0018	1.28E-06	22.8272
M01302.m	rs10066453	C	G	0.0387	0.0165	0.0035	1.93E-06	22.2245
M01302.m	rs11232103	A	C	0.1151	0.0085	0.0017	3.7E-07	25
M01302.m	rs11760350	A	G	0.1238	-0.0075	0.0017	7.77E-06	19.4637
M01302.m	rs16839317	T	C	0.8726	-0.0078	0.0017	2.75E-06	21.0519
M01302.m	rs1742639	A	G	0.8865	0.0077	0.0017	7.94E-06	20.5156
M01302.m	rs17478241	T	C	0.168	-0.0076	0.0016	2.75E-06	22.5625
M01302.m	rs2066415	T	C	0.3567	0.0065	0.0015	8.63E-06	18.7778
M01302.m	rs2418886	A	G	0.0489	0.0223	0.0047	1.63E-06	22.512
M01302.m	rs2780242	T	C	0.1138	-0.0083	0.0018	3.85E-06	21.2623
M01302.m	rs320485	T	C	0.1324	0.0094	0.0017	4.97E-08	30.5744
M01302.m	rs3740996	A	G	0.1208	0.0078	0.0017	4.33E-06	21.0519
M01302.m	rs4693555	T	C	0.2131	-0.0069	0.0016	8.43E-06	18.5977
M01302.m	rs487946	T	G	0.8292	-0.0074	0.0016	3.1E-06	21.3906
M01302.m	rs6008767	C	G	0.8182	-0.0077	0.0017	4.12E-06	20.5156
M01302.m	rs6071991	T	C	0.1495	-0.0075	0.0016	3.81E-06	21.9727
M01302.m	rs6533609	T	C	0.1429	-0.0077	0.0017	3.78E-06	20.5156
M01302.m	rs6766715	A	G	0.128	-0.009	0.002	6.8E-06	20.25
M01302.m	rs6960830	T	C	0.8851	0.0078	0.0017	4.16E-06	21.0519
M01302.m	rs7570141	A	G	0.104	-0.0119	0.0026	6.21E-06	20.9482
M01302.m	rs885076	A	G	0.8716	-0.0074	0.0017	8.75E-06	18.9481
M01302.m	rs9410970	A	C	0.2201	-0.0068	0.0015	5.49E-06	20.5511
M01302.m	rs9598459	A	G	0.896	0.0079	0.0017	5.17E-06	21.5952
M01303.m	rs10166484	T	C	0.3157	-0.0121	0.0027	5.29E-06	20.0837
M01303.m	rs10899384	A	G	0.3819	0.0139	0.0026	1.27E-07	28.5814
M01303.m	rs10990303	T	C	0.2309	-0.0129	0.0028	3.94E-06	21.2258
M01303.m	rs12713120	T	C	0.7564	0.0122	0.0027	6.74E-06	20.417
M01303.m	rs1939471	A	G	0.7209	-0.0123	0.0027	4.06E-06	20.7531
M01303.m	rs2026395	A	T	0.6013	-0.0116	0.0026	9.82E-06	19.9053
M01303.m	rs2288867	T	C	0.2334	-0.0132	0.0027	1.02E-06	23.9012
M01303.m	rs26724	A	G	0.3507	0.014	0.0026	1.26E-07	28.9941
M01303.m	rs2869990	T	C	0.7192	-0.0125	0.0027	2.83E-06	21.4335
M01303.m	rs4768795	T	G	0.3587	0.0195	0.0043	6.19E-06	20.5652
M01303.m	rs690191	T	C	0.7821	-0.0121	0.0027	9.23E-06	20.0837
M01303.m	rs7010256	C	G	0.0158	0.1003	0.0224	7.58E-06	20.0496
M01303.m	rs726900	T	G	0.8497	0.0173	0.0036	1.59E-06	23.0934
M01303.m	rs7503429	A	C	0.4255	-0.016	0.0026	1.64E-09	37.8698
M01303.m	rs8073225	T	C	0.7657	-0.013	0.0028	3.94E-06	21.5561
M01303.m	rs912170	A	G	0.8773	-0.0186	0.0036	2.09E-07	26.6944
M01303.m	rs9471841	T	C	0.0526	-0.0275	0.0062	9.84E-06	19.6735
M01336.m	rs10112003	A	G	0.0782	0.0158	0.0033	1.73E-06	22.9238
M01336.m	rs10199787	T	C	0.6173	0.0077	0.0017	6.2E-06	20.5156
M01336.m	rs10242171	A	G	0.1355	-0.0121	0.0026	3.73E-06	21.6583
M01336.m	rs11118120	A	C	0.8495	-0.0137	0.0029	3.16E-06	22.3175
M01336.m	rs11133892	A	G	0.4427	0.0089	0.0017	1.56E-07	27.4083
M01336.m	rs11193635	T	C	0.6724	0.0087	0.0018	7.34E-07	23.3611
M01336.m	rs11222474	T	G	0.6036	-0.0082	0.0017	1.42E-06	23.2664
M01336.m	rs11940037	T	C	0.8408	0.0121	0.0025	1.24E-06	23.4256

M01336.m	rs12147529	A	G	0.4485	0.0078	0.0017	5.92E-06	21.0519
M01336.m	rs12200468	A	G	0.8245	0.0111	0.0025	6.85E-06	19.7136
M01336.m	rs12353451	A	G	0.0373	-0.0229	0.0049	3.12E-06	21.8413
M01336.m	rs12931268	T	C	0.6329	-0.0087	0.0017	2.91E-07	26.1903
M01336.m	rs13355979	A	G	0.5663	-0.0078	0.0017	3.94E-06	21.0519
M01336.m	rs1348656	T	C	0.798	-0.0117	0.0024	9.07E-07	23.7656
M01336.m	rs1466228	T	C	0.035	-0.0234	0.0051	4.19E-06	21.0519
M01336.m	rs2190223	A	G	0.5733	0.0076	0.0017	7.69E-06	19.9862
M01336.m	rs2931383	T	G	0.5816	0.0078	0.0017	4.68E-06	21.0519
M01336.m	rs4408	A	G	0.1844	0.0107	0.0024	9.96E-06	19.8767
M01336.m	rs4420815	A	G	0.143	-0.015	0.003	5.73E-07	25
M01336.m	rs4470674	A	T	0.7184	-0.0106	0.0022	2.02E-06	23.2149
M01336.m	rs4957697	A	G	0.4208	-0.0081	0.0017	1.73E-06	22.7024
M01336.m	rs577266	A	C	0.637	-0.009	0.002	6.8E-06	20.25
M01336.m	rs603424	A	G	0.1836	0.0151	0.0024	2.46E-10	39.5851
M01336.m	rs6540294	A	C	0.451	-0.008	0.0017	2.87E-06	22.1453
M01336.m	rs7565567	A	C	0.982	-0.042	0.009	3.06E-06	21.7778
M01336.m	rs802993	A	G	0.0685	-0.0278	0.0063	9.11E-06	19.4719
M01336.m	rs942888	A	G	0.4776	-0.0076	0.0017	6.29E-06	19.9862
M01336.m	rs9809122	A	G	0.5296	-0.008	0.0017	2.45E-06	22.1453
M01356.m	rs10021069	T	C	0.9664	0.031	0.0067	4.07E-06	21.4079
M01356.m	rs10156335	A	G	0.8473	-0.0153	0.0034	7.17E-06	20.25
M01356.m	rs11118895	T	C	0.4675	0.0202	0.0045	7.16E-06	20.1501
M01356.m	rs11674467	T	C	0.4181	0.0148	0.003	7.01E-07	24.3378
M01356.m	rs11780703	T	C	0.7557	-0.0127	0.0026	9.89E-07	23.8595
M01356.m	rs11824733	T	C	0.0212	0.072	0.0161	7.64E-06	19.9992
M01356.m	rs1483739	A	C	0.2369	0.0117	0.0026	9.51E-06	20.25
M01356.m	rs17066836	C	G	0.0202	-0.0707	0.0157	6.24E-06	20.2787
M01356.m	rs17511568	T	C	0.8423	0.0152	0.0033	5.26E-06	21.2158
M01356.m	rs1801838	T	C	0.9655	-0.0469	0.0105	8.51E-06	19.9511
M01356.m	rs281216	T	G	0.9184	0.0205	0.0043	1.7E-06	22.7285
M01356.m	rs4472784	T	C	0.3048	-0.0118	0.0026	4.78E-06	20.5976
M01356.m	rs603424	A	G	0.1833	0.0182	0.0032	2.23E-08	32.3477
M01356.m	rs6486528	C	G	0.9236	0.0228	0.0049	3.3E-06	21.651
M01356.m	rs9291034	T	C	0.4068	-0.0117	0.0026	5.42E-06	20.25
M01356.m	rs9543361	T	C	0.0422	-0.0514	0.011	3.18E-06	21.8344
M01358.m	rs10021069	T	C	0.9663	0.0238	0.005	1.81E-06	22.6576
M01358.m	rs10152773	T	C	0.0425	-0.0224	0.0049	4.73E-06	20.898
M01358.m	rs10160169	A	G	0.6329	0.0084	0.0018	3.78E-06	21.7778
M01358.m	rs10242171	A	G	0.1356	-0.012	0.0026	4.25E-06	21.3018
M01358.m	rs10498606	A	C	0.4534	-0.0077	0.0017	6.4E-06	20.5156
M01358.m	rs1075227	T	C	0.552	0.009	0.002	6.8E-06	20.25
M01358.m	rs10757839	T	C	0.6521	0.0079	0.0017	4.74E-06	21.5952
M01358.m	rs10853932	T	C	0.345	0.008	0.0018	5.45E-06	19.7531
M01358.m	rs11118120	A	C	0.8531	-0.0121	0.0025	1.18E-06	23.4256
M01358.m	rs11940037	T	C	0.8408	0.0125	0.0025	7.01E-07	25
M01358.m	rs12353451	A	G	0.0373	-0.0223	0.0049	6.06E-06	20.7118
M01358.m	rs1237044	T	C	0.5836	0.0081	0.0018	5.1E-06	20.25
M01358.m	rs13355979	A	G	0.5665	-0.0076	0.0017	6.23E-06	19.9862
M01358.m	rs1348656	T	C	0.798	-0.0137	0.0024	1.17E-08	32.5851
M01358.m	rs1426441	A	G	0.1423	0.0113	0.0025	4.87E-06	20.4304
M01358.m	rs1477025	T	C	0.6547	0.0087	0.0017	4.83E-07	26.1903
M01358.m	rs1502593	A	G	0.3909	0.0081	0.0017	2.5E-06	22.7024
M01358.m	rs1662999	T	C	0.6325	0.0079	0.0017	4.21E-06	21.5952
M01358.m	rs1922419	A	G	0.7807	-0.0182	0.0039	3.37E-06	21.7778

M01358.m	rs2100884	T	C	0.4375	-0.0084	0.0017	8.6E-07	24.4152
M01358.m	rs2115366	T	C	0.4418	-0.0079	0.0017	3.93E-06	21.5952
M01358.m	rs216283	A	C	0.4171	0.008	0.0017	2.63E-06	22.1453
M01358.m	rs2280248	A	G	0.4808	-0.0077	0.0017	5.55E-06	20.5156
M01358.m	rs2489020	T	C	0.5262	-0.0081	0.0017	2.3E-06	22.7024
M01358.m	rs281216	T	G	0.9187	0.0149	0.0033	8.26E-06	20.3866
M01358.m	rs3087751	A	C	0.423	0.0078	0.0017	5.23E-06	21.0519
M01358.m	rs3748771	T	G	0.9877	-0.0671	0.0149	6.35E-06	20.2802
M01358.m	rs3793900	A	C	0.6337	-0.0078	0.0017	5.73E-06	21.0519
M01358.m	rs3863373	A	C	0.3335	-0.008	0.0017	4.21E-06	22.1453
M01358.m	rs4420815	A	G	0.143	-0.014	0.003	3.06E-06	21.7778
M01358.m	rs4507103	A	G	0.397	0.0077	0.0017	6.89E-06	20.5156
M01358.m	rs4710948	A	G	0.3382	-0.0085	0.0017	6.94E-07	25
M01358.m	rs4750059	C	G	0.7956	-0.0134	0.003	6.49E-06	19.9511
M01358.m	rs603424	A	G	0.1835	0.0225	0.0024	6.06E-21	87.8906
M01358.m	rs6453594	A	G	0.2196	-0.0108	0.0024	7.01E-06	20.25
M01358.m	rs6733405	A	G	0.3867	-0.0082	0.0017	2.42E-06	23.2664
M01358.m	rs6883481	T	C	0.4737	0.0077	0.0017	6.17E-06	20.5156
M01358.m	rs727892	A	G	0.5021	0.0083	0.0017	8.15E-07	23.8374
M01358.m	rs7583745	T	C	0.8839	-0.0262	0.0055	1.49E-06	22.6922
M01358.m	rs7681360	A	G	0.5825	0.0078	0.0017	4.19E-06	21.0519
M01358.m	rs7996008	A	G	0.2935	0.0079	0.0017	5.69E-06	21.5952
M01358.m	rs954820	A	G	0.5619	0.0083	0.0018	2.57E-06	21.2623
M01358.m	rs9568232	T	C	0.1152	0.0126	0.0028	5.06E-06	20.25
M01358.m	rs9809122	A	G	0.5295	-0.0087	0.0017	3.92E-07	26.1903
M01359.m	rs10112003	A	G	0.0783	0.019	0.004	2.41E-06	22.5625
M01359.m	rs10242171	A	G	0.135	-0.0154	0.0033	4.32E-06	21.7778
M01359.m	rs10746614	A	G	0.2333	-0.0114	0.0025	4.05E-06	20.7936
M01359.m	rs11118120	A	C	0.8514	-0.0142	0.0031	6.01E-06	20.9823
M01359.m	rs11133892	A	G	0.4423	0.012	0.0023	2.65E-07	27.2212
M01359.m	rs12451448	A	G	0.2677	-0.0116	0.0025	2.3E-06	21.5296
M01359.m	rs13019537	C	G	0.841	0.0157	0.0032	7.52E-07	24.0713
M01359.m	rs1348656	T	C	0.7976	-0.0172	0.003	1.06E-08	32.8711
M01359.m	rs17495226	A	G	0.0566	0.0216	0.0047	5.67E-06	21.1209
M01359.m	rs1922578	A	G	0.9821	-0.0391	0.0088	9.36E-06	19.7419
M01359.m	rs2017823	A	G	0.841	0.0142	0.0031	4.85E-06	20.9823
M01359.m	rs35350	A	G	0.9692	0.0376	0.0083	5.44E-06	20.522
M01359.m	rs4287543	A	G	0.8771	0.015	0.0033	4.54E-06	20.6612
M01359.m	rs4353846	T	C	0.5643	-0.0112	0.0023	1.7E-06	23.7127
M01359.m	rs4573205	T	G	0.8698	-0.0146	0.0033	8.72E-06	19.5739
M01359.m	rs4654501	A	G	0.7667	0.011	0.0025	7.41E-06	19.36
M01359.m	rs6453591	A	T	0.771	0.0133	0.0025	8.55E-08	28.3024
M01359.m	rs7586280	A	T	0.3011	-0.011	0.0024	4.11E-06	21.0069
M01359.m	rs7898173	A	G	0.1431	-0.0138	0.0031	8.96E-06	19.8169
M01359.m	rs7951538	C	G	0.7061	-0.0107	0.0024	8.09E-06	19.8767
M01359.m	rs9861865	C	G	0.3196	-0.0107	0.0024	8.06E-06	19.8767
M01361.m	rs10509840	A	G	0.6879	-0.0119	0.0026	4.23E-06	20.9482
M01361.m	rs11036893	T	C	0.3592	-0.013	0.0025	3.01E-07	27.04
M01361.m	rs11245200	T	C	0.1119	0.018	0.004	8.94E-06	20.25
M01361.m	rs11791164	T	G	0.0803	0.0196	0.0043	4.35E-06	20.7766
M01361.m	rs16830012	T	G	0.9719	-0.0328	0.0073	7.41E-06	20.1884
M01361.m	rs17056226	T	G	0.1416	0.0153	0.0034	7.07E-06	20.25
M01361.m	rs17385848	A	G	0.6443	0.0122	0.0025	1.16E-06	23.8144
M01361.m	rs17773390	A	G	0.2915	0.0123	0.0026	3.25E-06	22.3802
M01361.m	rs1830241	A	G	0.3431	0.0118	0.0026	7.5E-06	20.5976

M01361.m	rs3135095	A	G	0.2993	-0.0117	0.0025	3.92E-06	21.9024
M01361.m	rs3900774	T	G	0.988	0.061	0.013	2.7E-06	22.0178
M01361.m	rs4960640	T	C	0.9667	-0.0285	0.0064	9.7E-06	19.8303
M01361.m	rs6471261	T	G	0.2806	0.0115	0.0025	6.16E-06	21.16
M01361.m	rs6472299	A	G	0.8381	-0.0149	0.0033	7.87E-06	20.3866
M01361.m	rs6783294	A	G	0.4281	-0.0213	0.0045	2.41E-06	22.4044
M01361.m	rs6887589	A	G	0.7787	-0.0142	0.0026	4.33E-08	29.8284
M01361.m	rs7710912	A	G	0.0728	-0.0189	0.0042	8.3E-06	20.25
M01361.m	rs7791503	T	C	0.269	0.0126	0.0026	9.65E-07	23.4852
M01361.m	rs8098317	T	G	0.7857	-0.0119	0.0026	6.64E-06	20.9482
M01361.m	rs961959	A	C	0.1925	-0.0121	0.0027	5.26E-06	20.0837
M01365.m	rs10005295	A	G	0.0267	-0.0634	0.0136	3.4E-06	21.7321
M01365.m	rs10164158	A	G	0.0577	0.0229	0.005	4.18E-06	20.9764
M01365.m	rs10494825	T	C	0.2783	-0.0114	0.0026	9.59E-06	19.2249
M01365.m	rs10831497	A	G	0.2209	0.0126	0.0028	4.97E-06	20.25
M01365.m	rs11118120	A	C	0.8534	-0.0175	0.0033	1.71E-07	28.1221
M01365.m	rs12250605	T	C	0.751	0.0122	0.0026	2.02E-06	22.0178
M01365.m	rs12426539	T	C	0.0306	0.0405	0.0091	9.31E-06	19.8074
M01365.m	rs12967799	A	C	0.0794	0.0193	0.0043	6.13E-06	20.1455
M01365.m	rs13393013	A	G	0.0218	0.0415	0.0085	1.08E-06	23.8374
M01365.m	rs17155949	T	C	0.9746	0.0698	0.0153	4.75E-06	20.8127
M01365.m	rs1936396	A	G	0.2253	-0.0118	0.0026	5.67E-06	20.5976
M01365.m	rs314241	T	C	0.4625	-0.0114	0.0025	5.25E-06	20.7936
M01365.m	rs35350	A	G	0.9722	0.0397	0.0089	8.1E-06	19.8976
M01365.m	rs3759526	T	C	0.0806	-0.0209	0.0043	8.93E-07	23.6241
M01365.m	rs4109037	A	T	0.146	0.0162	0.0033	9.49E-07	24.0992
M01365.m	rs4327548	A	G	0.291	-0.0121	0.0026	2.25E-06	21.6583
M01365.m	rs4420815	A	G	0.143	-0.019	0.004	2.03E-06	22.5625
M01365.m	rs453612	T	C	0.3549	0.0114	0.0025	5.85E-06	20.7936
M01365.m	rs4710948	A	G	0.3383	-0.0115	0.0025	4.85E-06	21.16
M01365.m	rs603424	A	G	0.1832	0.0162	0.0032	5.76E-07	25.6289
M01365.m	rs6467872	A	C	0.6087	0.0114	0.0025	5.64E-06	20.7936
M01365.m	rs6665988	A	C	0.7127	-0.0115	0.0026	9.11E-06	19.5636
M01365.m	rs670365	A	G	0.0903	0.039	0.0088	9.48E-06	19.641
M01365.m	rs7016645	T	C	0.253	-0.0117	0.0026	8.44E-06	20.25
M01365.m	rs7796330	T	C	0.2727	0.0116	0.0026	7.93E-06	19.9053
M01444.m	rs11184997	A	T	0.2894	0.0179	0.0039	6.01E-06	21.0657
M01444.m	rs11718405	T	C	0.8358	0.0216	0.0048	8.43E-06	20.25
M01444.m	rs12725284	T	C	0.1606	0.0315	0.0056	1.72E-08	31.6406
M01444.m	rs1476414	C	G	0.0535	0.0397	0.0081	9.3E-07	24.0221
M01444.m	rs3010320	A	T	0.232	-0.0205	0.0042	1E-06	23.8237
M01444.m	rs35421463	A	G	0.4033	0.0277	0.006	4.15E-06	21.3136
M01444.m	rs3915237	T	G	0.7005	-0.0182	0.004	5.52E-06	20.7025
M01444.m	rs4443239	T	G	0.7453	0.031	0.0069	7.58E-06	20.1848
M01444.m	rs4590377	T	C	0.7362	-0.0178	0.004	8.79E-06	19.8025
M01444.m	rs4717457	A	C	0.1392	0.0233	0.0052	6.8E-06	20.0773
M01444.m	rs6497209	A	G	0.2139	0.019	0.0042	4.78E-06	20.4649
M01444.m	rs699474	C	G	0.0134	0.1282	0.0264	1.16E-06	23.5813
M01444.m	rs7561932	T	C	0.7721	0.0184	0.0042	9.55E-06	19.1927
M01444.m	rs7871735	T	G	0.2953	0.0184	0.004	3.4E-06	21.16
M01444.m	rs9295427	A	G	0.9408	0.0356	0.0077	3.81E-06	21.3756
M01444.m	rs9460045	T	C	0.9224	-0.0529	0.0119	8.91E-06	19.7614
M01493.m	rs10497426	T	C	0.8064	0.012	0.0026	4.47E-06	21.3018
M01493.m	rs10900040	T	C	0.8351	-0.0144	0.0031	3.4E-06	21.5775
M01493.m	rs10945970	T	C	0.3103	-0.011	0.0025	7.38E-06	19.36

M01493.m	rs11701867	A	C	0.1761	-0.0136	0.0027	2.75E-07	25.3717
M01493.m	rs1192521	A	T	0.7755	-0.0163	0.0031	1.4E-07	27.6472
M01493.m	rs12300345	A	T	0.132	-0.0151	0.0034	7.45E-06	19.724
M01493.m	rs13424082	A	G	0.1735	-0.0125	0.0026	1.8E-06	23.1139
M01493.m	rs2351725	A	T	0.7947	-0.0119	0.0026	5.84E-06	20.9482
M01493.m	rs4764034	A	G	0.6869	-0.011	0.0024	7.67E-06	21.0069
M01493.m	rs4812400	A	T	0.9092	0.0178	0.0039	4.89E-06	20.831
M01493.m	rs6975921	A	G	0.9095	0.0343	0.0071	1.25E-06	23.3384
M01494.m	rs1001210	T	C	0.8353	-0.0106	0.0023	3.8E-06	21.2401
M01494.m	rs10853533	A	C	0.8743	0.0103	0.0023	6.09E-06	20.0548
M01494.m	rs10890517	T	C	0.0433	-0.0197	0.0043	3.45E-06	20.9892
M01494.m	rs11582143	C	G	0.1443	-0.0112	0.0023	9.32E-07	23.7127
M01494.m	rs12143589	A	G	0.1428	0.0118	0.0023	3.38E-07	26.3214
M01494.m	rs12294182	T	C	0.0987	0.014	0.0029	1.07E-06	23.3056
M01494.m	rs12550729	T	C	0.9233	0.0637	0.0029	6E-110	482.484
M01494.m	rs12611788	T	C	0.6267	-0.007	0.0015	5.51E-06	21.7778
M01494.m	rs13013224	C	G	0.1794	0.0113	0.0023	7.04E-07	24.138
M01494.m	rs13159409	T	G	0.0652	-0.0148	0.0032	2.74E-06	21.3906
M01494.m	rs1578743	A	C	0.6068	0.0075	0.0016	1.7E-06	21.9727
M01494.m	rs2068157	T	C	0.9144	0.0137	0.0031	8.99E-06	19.5307
M01494.m	rs2115151	A	T	0.8627	0.0103	0.0022	3.75E-06	21.9194
M01494.m	rs2592916	T	C	0.1013	-0.0123	0.0027	6.55E-06	20.7531
M01494.m	rs306676	A	G	0.1304	0.0112	0.0024	2.48E-06	21.7778
M01494.m	rs3936211	T	C	0.424	-0.0119	0.0015	1.34E-14	62.9378
M01494.m	rs4646693	T	C	0.435	-0.009	0.002	6.8E-06	20.25
M01494.m	rs5764925	A	G	0.0772	-0.016	0.0031	1.91E-07	26.6389
M01494.m	rs7561255	A	G	0.7086	-0.0072	0.0016	4.72E-06	20.25
M01494.m	rs764206	A	G	0.6546	0.0071	0.0015	4.58E-06	22.4044
M01494.m	rs7973508	A	G	0.2831	-0.0073	0.0016	5.4E-06	20.8164
M01494.m	rs8092658	A	C	0.389	-0.009	0.002	6.8E-06	20.25
M01494.m	rs9460424	T	G	0.8437	-0.0097	0.0022	9.16E-06	19.4401
M01494.m	rs9964014	T	C	0.1115	-0.0132	0.0025	1.8E-07	27.8784
M01494.m	rs9987070	C	G	0.9808	-0.028	0.0059	2.43E-06	22.5223
M01508.m	rs10919047	A	C	0.1408	0.0176	0.0035	6.75E-07	25.2865
M01508.m	rs11719633	A	G	0.0945	-0.0206	0.0044	3.01E-06	21.9194
M01508.m	rs12544560	A	G	0.148	0.0164	0.0035	3.32E-06	21.9559
M01508.m	rs13212846	A	C	0.9252	-0.0241	0.0052	2.88E-06	21.4797
M01508.m	rs1350858	A	C	0.1783	0.0152	0.0034	8.21E-06	19.9862
M01508.m	rs1395	A	G	0.7034	0.0172	0.0027	8.14E-11	40.5816
M01508.m	rs17049936	T	C	0.8912	-0.0187	0.0042	9.58E-06	19.8237
M01508.m	rs17082600	T	C	0.0193	0.0476	0.0094	4.08E-07	25.6424
M01508.m	rs17751805	A	G	0.9645	0.0628	0.0135	3.4E-06	21.6397
M01508.m	rs1975271	T	C	0.5872	0.0116	0.0026	8.68E-06	19.9053
M01508.m	rs2173615	T	G	0.3153	0.0125	0.0026	1.69E-06	23.1139
M01508.m	rs2526477	A	G	0.609	-0.012	0.0026	2.99E-06	21.3018
M01508.m	rs2967437	A	G	0.6902	-0.0126	0.0027	1.88E-06	21.7778
M01508.m	rs3949865	C	G	0.6187	-0.0115	0.0026	9.49E-06	19.5636
M01508.m	rs4269521	A	G	0.8431	0.0168	0.0034	1.03E-06	24.4152
M01508.m	rs4746017	A	C	0.713	-0.0122	0.0027	5.1E-06	20.417
M01508.m	rs6034318	A	G	0.013	0.1083	0.0236	4.52E-06	21.0588
M01508.m	rs6078500	T	C	0.5117	-0.0118	0.0026	4.47E-06	20.5976
M01508.m	rs6774965	A	G	0.8582	-0.0163	0.0035	3.16E-06	21.689
M01508.m	rs6848070	A	G	0.3521	-0.0117	0.0027	9.91E-06	18.7778
M01508.m	rs7009776	T	C	0.3342	-0.0268	0.006	7.12E-06	19.9511
M01508.m	rs7458704	T	G	0.984	-0.0544	0.0109	5.6E-07	24.9083

M01508.m	rs7574257	T	C	0.325	0.0118	0.0026	7.76E-06	20.5976
M01508.m	rs8065435	A	G	0.327	-0.0128	0.0028	5.03E-06	20.898
M01508.m	rs9365106	A	G	0.6824	-0.0117	0.0026	8.06E-06	20.25
M01515.x	rs10205265	T	C	0.2056	-0.0901	0.0202	7.79E-06	19.8951
M01515.x	rs10402106	T	C	0.0165	0.9529	0.1924	7.35E-07	24.5293
M01515.x	rs10458721	A	G	0.609	-0.0786	0.0175	7.23E-06	20.1729
M01515.x	rs10471169	T	C	0.0105	0.9362	0.1854	4.43E-07	25.4987
M01515.x	rs10749553	A	G	0.3074	-0.0868	0.0194	7.55E-06	20.0187
M01515.x	rs10977627	T	C	0.0168	0.8146	0.1524	9.06E-08	28.5706
M01515.x	rs10984575	A	C	0.1164	0.1144	0.0257	8.27E-06	19.8146
M01515.x	rs11256026	A	G	0.6357	-0.2061	0.0409	4.6E-07	25.3927
M01515.x	rs1164673	C	G	0.3743	0.0754	0.0168	7.27E-06	20.143
M01515.x	rs12005082	A	T	0.1814	0.1232	0.0265	3.35E-06	21.6137
M01515.x	rs12075728	T	G	0.9442	-0.4196	0.0922	5.39E-06	20.7114
M01515.x	rs16832600	T	C	0.0297	0.2205	0.0469	2.58E-06	22.104
M01515.x	rs16862165	T	C	0.99	-0.413	0.089	3.48E-06	21.5338
M01515.x	rs17682068	A	G	0.9248	-0.1299	0.029	7.45E-06	20.0642
M01515.x	rs400214	T	C	0.0185	0.3258	0.0723	6.6E-06	20.3061
M01515.x	rs7701675	A	G	0.9839	-0.7943	0.1733	4.56E-06	21.0074
M01515.x	rs8178289	A	C	0.8497	-0.1064	0.0233	5.1E-06	20.8531
M01515.x	rs9453415	A	G	0.011	0.374	0.083	6.61E-06	20.3043
M01558.m	rs10004130	A	G	0.581	0.009	0.002	6.8E-06	20.25
M01558.m	rs10122824	T	G	0.3503	0.0087	0.0019	2.79E-06	20.9668
M01558.m	rs10416031	A	G	0.3202	0.0085	0.0018	3.84E-06	22.2994
M01558.m	rs1041983	T	C	0.3233	-0.0161	0.0018	2.14E-19	80.0031
M01558.m	rs10496826	A	G	0.2948	-0.008	0.0018	7.77E-06	19.7531
M01558.m	rs11023380	A	G	0.4591	0.0088	0.0018	5.37E-07	23.9012
M01558.m	rs1113643	T	C	0.2769	0.0094	0.0018	2.24E-07	27.2716
M01558.m	rs11170032	T	C	0.64	-0.008	0.0018	7.18E-06	19.7531
M01558.m	rs11650615	C	G	0.6907	0.0081	0.0018	5.99E-06	20.25
M01558.m	rs1280717	A	G	0.2512	0.0094	0.0018	2.01E-07	27.2716
M01558.m	rs13164411	T	C	0.7246	0.0082	0.0018	5.33E-06	20.7531
M01558.m	rs13271577	T	C	0.1383	0.0219	0.0026	2.56E-17	70.9482
M01558.m	rs1493031	T	C	0.0434	0.0208	0.0044	1.96E-06	22.3471
M01558.m	rs1538330	T	G	0.0213	-0.0306	0.0064	1.73E-06	22.8604
M01558.m	rs1556880	T	C	0.2976	0.0081	0.0018	6.04E-06	20.25
M01558.m	rs1604290	A	C	0.3851	-0.0079	0.0018	8.13E-06	19.2623
M01558.m	rs164742	A	G	0.4697	-0.0079	0.0018	7.38E-06	19.2623
M01558.m	rs17642859	A	T	0.1138	-0.0173	0.0033	1.16E-07	27.483
M01558.m	rs1812125	T	G	0.7578	-0.0085	0.0018	3.48E-06	22.2994
M01558.m	rs2029830	T	C	0.3011	0.0098	0.0018	3.49E-08	29.642
M01558.m	rs2055056	T	C	0.968	-0.0244	0.0055	8.57E-06	19.6813
M01558.m	rs2144834	A	G	0.2535	0.008	0.0018	7.97E-06	19.7531
M01558.m	rs2252585	T	C	0.7349	-0.0081	0.0018	5.97E-06	20.25
M01558.m	rs2290156	C	G	0.2806	0.0088	0.0018	1.05E-06	23.9012
M01558.m	rs2604548	T	C	0.6463	-0.0086	0.0018	1.29E-06	22.8272
M01558.m	rs304171	T	C	0.0624	-0.0168	0.0036	2.17E-06	21.7778
M01558.m	rs3813810	A	C	0.1334	0.0117	0.0026	5.73E-06	20.25
M01558.m	rs4443239	T	G	0.7446	0.02	0.0042	2.27E-06	22.6757
M01558.m	rs4687937	T	C	0.3741	-0.0079	0.0018	9.47E-06	19.2623
M01558.m	rs4692012	A	G	0.5389	-0.0082	0.0018	3.35E-06	20.7531
M01558.m	rs4923082	T	C	0.2518	0.0081	0.0018	6.95E-06	20.25
M01558.m	rs604388	T	C	0.6845	0.0086	0.0018	1.56E-06	22.8272
M01558.m	rs6500864	A	G	0.2762	-0.0083	0.0018	4.34E-06	21.2623
M01558.m	rs721399	T	C	0.7176	-0.0289	0.0018	3.95E-58	257.781

M01558.m	rs7228099	T	C	0.4364	-0.0097	0.0018	3.61E-08	29.0401
M01558.m	rs7817770	T	C	0.3971	0.0094	0.0018	9.42E-08	27.2716
M01558.m	rs7943587	A	C	0.1515	-0.0115	0.0026	6.33E-06	19.5636
M01558.m	rs890984	A	G	0.1092	-0.012	0.0027	6.91E-06	19.7531
M01558.m	rs9354329	T	C	0.6322	-0.0081	0.0018	5.1E-06	20.25
M01558.m	rs9657488	T	C	0.3749	0.0124	0.0018	3.53E-12	47.4568
M01558.m	rs9940567	T	C	0.7319	0.0095	0.0018	1.57E-07	27.8549
M01561.m	rs10163969	T	G	0.0412	-0.0352	0.008	9.39E-06	19.36
M01561.m	rs10245705	T	C	0.0181	-0.0663	0.0127	1.95E-07	27.2533
M01561.m	rs10935814	A	G	0.1006	-0.037	0.0083	9.44E-06	19.8723
M01561.m	rs11145330	A	C	0.8908	0.0324	0.0068	1.95E-06	22.7024
M01561.m	rs11992435	A	G	0.9488	0.0327	0.0072	6.38E-06	20.6267
M01561.m	rs1404410	C	G	0.7853	-0.0236	0.0052	4.57E-06	20.5976
M01561.m	rs1532701	A	G	0.5456	0.0137	0.003	5.07E-06	20.8544
M01561.m	rs2074731	A	C	0.1654	-0.0184	0.0039	2.31E-06	22.259
M01561.m	rs261342	C	G	0.794	-0.0169	0.0037	5.41E-06	20.8627
M01561.m	rs7238006	T	C	0.9262	0.0281	0.0057	6.77E-07	24.3032
M01561.m	rs7930821	T	C	0.0202	0.0666	0.0149	7.53E-06	19.9791
M01564.m	rs10043015	T	C	0.7113	0.0082	0.0018	5.58E-06	20.7531
M01564.m	rs10514735	A	G	0.985	0.0346	0.0077	6.51E-06	20.1916
M01564.m	rs10787308	A	G	0.2701	-0.009	0.0018	6.04E-07	25
M01564.m	rs10992722	A	C	0.0163	0.0326	0.0069	2.44E-06	22.3222
M01564.m	rs11120725	A	G	0.4596	-0.0181	0.0039	4.61E-06	21.5391
M01564.m	rs1125471	A	C	0.2928	-0.0085	0.0018	4.47E-06	22.2994
M01564.m	rs12121428	T	C	0.0535	-0.0197	0.0044	6.09E-06	20.046
M01564.m	rs12625828	T	G	0.3366	0.0086	0.0018	2.06E-06	22.8272
M01564.m	rs12803308	A	T	0.6752	-0.0081	0.0018	5.97E-06	20.25
M01564.m	rs13130021	T	C	0.4312	-0.0089	0.0018	1.41E-06	24.4475
M01564.m	rs1380634	A	G	0.7425	-0.0083	0.0018	5.17E-06	21.2623
M01564.m	rs13966	T	C	0.467	0.0082	0.0019	9.99E-06	18.626
M01564.m	rs1404373	T	C	0.6525	-0.0084	0.0018	2.92E-06	21.7778
M01564.m	rs1509123	T	C	0.0476	-0.0248	0.0043	5.2E-09	33.2634
M01564.m	rs1530219	A	G	0.4797	-0.0078	0.0018	9.94E-06	18.7778
M01564.m	rs170149	A	G	0.074	0.0256	0.0035	1.42E-13	53.4988
M01564.m	rs17034684	T	G	0.2881	0.0081	0.0018	7.9E-06	20.25
M01564.m	rs2143327	T	C	0.0129	0.0392	0.0085	4.28E-06	21.2684
M01564.m	rs218682	T	C	0.3999	0.0093	0.0018	2.98E-07	26.6944
M01564.m	rs2236299	A	G	0.3065	0.0087	0.0018	1.69E-06	23.3611
M01564.m	rs2239337	C	G	0.0356	0.023	0.0051	7.51E-06	20.3383
M01564.m	rs2370136	T	C	0.4875	-0.0086	0.0018	1.25E-06	22.8272
M01564.m	rs2534835	T	G	0.2407	-0.0083	0.0018	6.23E-06	21.2623
M01564.m	rs3193970	T	C	0.5801	0.0081	0.0018	6.07E-06	20.25
M01564.m	rs4235093	A	G	0.2874	-0.0083	0.0018	5.04E-06	21.2623
M01564.m	rs4589188	A	G	0.2827	0.0122	0.0026	3.34E-06	22.0178
M01564.m	rs4779800	A	G	0.8831	-0.0122	0.0027	6.06E-06	20.417
M01564.m	rs4999864	C	G	0.9329	0.0179	0.0035	4.77E-07	26.1559
M01564.m	rs588973	A	G	0.646	0.009	0.002	6.8E-06	20.25
M01564.m	rs6077792	T	G	0.3	-0.0085	0.0018	2.82E-06	22.2994
M01564.m	rs6507399	A	G	0.6713	-0.0101	0.0018	1.7E-08	31.4846
M01564.m	rs6678076	A	G	0.0339	-0.0252	0.0052	1.08E-06	23.4852
M01564.m	rs7105815	A	G	0.2968	0.0091	0.0018	4.93E-07	25.5586
M01564.m	rs7152965	A	G	0.4052	0.0079	0.0018	9E-06	19.2623
M01564.m	rs7584253	A	C	0.7047	0.0083	0.0018	5.04E-06	21.2623
M01564.m	rs7710027	T	C	0.2886	0.0081	0.0018	9.16E-06	20.25
M01564.m	rs7851531	A	G	0.6594	-0.009	0.0018	6.47E-07	25

M01564.m	rs7919076	T	C	0.3416	-0.0081	0.0018	7.79E-06	20.25
M01564.m	rs8017368	A	C	0.6452	-0.0083	0.0018	3.85E-06	21.2623
M01564.m	rs807670	A	C	0.421	0.011	0.0018	6.84E-10	37.3457
M01564.m	rs835154	A	G	0.5753	-0.0114	0.0018	1.15E-10	40.1111
M01564.m	rs868395	T	G	0.6653	0.0101	0.0018	2.03E-08	31.4846
M01564.m	rs889128	T	G	0.6665	0.0082	0.0018	6.75E-06	20.7531
M01564.m	rs906749	A	G	0.7158	0.0105	0.0018	1.08E-08	34.0278
M01564.m	rs910616	T	C	0.4991	-0.0093	0.0018	1.97E-07	26.6944
M01564.m	rs9327706	A	C	0.5937	0.0081	0.0018	5.61E-06	20.25
M01564.m	rs9372398	T	C	0.5453	0.0081	0.0018	6.74E-06	20.25
M01564.m	rs9389071	A	G	0.1579	-0.012	0.0026	5.64E-06	21.3018
M01564.m	rs940697	A	G	0.2701	0.0082	0.0018	5.71E-06	20.7531
M01572.m	rs11061416	A	G	0.6428	-0.0125	0.0024	1.76E-07	27.1267
M01572.m	rs11618222	T	C	0.1008	-0.0182	0.004	6E-06	20.7025
M01572.m	rs11724197	A	G	0.8734	0.0159	0.0034	2.29E-06	21.8694
M01572.m	rs12507784	A	G	0.2395	-0.012	0.0025	1.78E-06	23.04
M01572.m	rs12866131	A	C	0.2805	0.011	0.0024	5.62E-06	21.0069
M01572.m	rs13102848	C	G	0.8686	-0.0152	0.0033	3.13E-06	21.2158
M01572.m	rs17011367	T	C	0.7325	0.0115	0.0026	7.38E-06	19.5636
M01572.m	rs17086746	T	C	0.02	-0.046	0.01	4.23E-06	21.16
M01572.m	rs17131975	A	G	0.0607	-0.0216	0.0048	8.18E-06	20.25
M01572.m	rs2643891	A	G	0.165	-0.018	0.004	6.8E-06	20.25
M01572.m	rs2907828	C	G	0.1375	-0.0188	0.004	2.74E-06	22.09
M01572.m	rs338526	T	C	0.7181	-0.0111	0.0024	5.61E-06	21.3906
M01572.m	rs4836200	A	G	0.6785	-0.0109	0.0024	5.91E-06	20.6267
M01572.m	rs6743210	T	C	0.6199	0.0123	0.0026	2.21E-06	22.3802
M01572.m	rs6896132	C	G	0.219	0.0121	0.0025	1.8E-06	23.4256
M01572.m	rs7861435	C	G	0.7685	-0.0115	0.0025	4.01E-06	21.16
M01572.m	rs884870	C	G	0.0689	-0.0202	0.0045	7.4E-06	20.1501
M01573.m	rs11141909	A	C	0.0405	0.0808	0.0182	9.02E-06	19.7097
M01573.m	rs12052900	A	G	0.9783	-0.1115	0.0242	4.27E-06	21.2285
M01573.m	rs1577941	A	G	0.1187	0.0531	0.0114	3.49E-06	21.696
M01573.m	rs16887484	A	G	0.9585	-0.0991	0.0201	8.82E-07	24.3083
M01573.m	rs16933423	A	G	0.9651	0.0891	0.02	8.31E-06	19.847
M01573.m	rs17224277	A	G	0.0789	0.0633	0.0134	2.29E-06	22.315
M01573.m	rs2257243	A	G	0.6612	0.0354	0.0075	2.19E-06	22.2784
M01573.m	rs2846365	A	G	0.4476	0.0336	0.0071	1.98E-06	22.3956
M01573.m	rs4661299	T	G	0.4551	0.0319	0.0071	7.63E-06	20.1867
M01573.m	rs4948051	T	C	0.1328	0.0528	0.0115	4.84E-06	21.0801
M01573.m	rs7137298	C	G	0.176	-0.0457	0.0103	8.82E-06	19.686
M01573.m	rs7438272	T	C	0.1691	0.0589	0.0123	1.8E-06	22.9309
M01573.m	rs9353315	A	G	0.8731	-0.0476	0.0105	5.86E-06	20.5511
M01585.m	rs10090064	T	C	0.8026	0.0077	0.0017	5.93E-06	20.5156
M01585.m	rs10125203	C	G	0.8698	0.009	0.0018	4.11E-07	25
M01585.m	rs10757735	A	C	0.132	-0.008	0.0018	7.91E-06	19.7531
M01585.m	rs10850426	T	G	0.1199	0.0081	0.0018	6.85E-06	20.25
M01585.m	rs10988352	T	G	0.1244	0.0084	0.0018	3.09E-06	21.7778
M01585.m	rs11045425	A	G	0.1266	0.0086	0.0018	1.83E-06	22.8272
M01585.m	rs11144136	T	G	0.1375	0.0097	0.0018	4.86E-08	29.0401
M01585.m	rs11606364	T	C	0.1149	0.0084	0.0019	9.27E-06	19.5457
M01585.m	rs1554074	A	G	0.1925	0.0086	0.0017	6.39E-07	25.5917
M01585.m	rs2019107	T	C	0.6257	-0.0078	0.0017	6.66E-06	21.0519
M01585.m	rs2486925	T	G	0.1462	-0.0082	0.0018	3.47E-06	20.7531
M01585.m	rs2596194	A	T	0.1255	-0.0083	0.0018	2.59E-06	21.2623
M01585.m	rs266742	T	G	0.7307	0.0082	0.0016	5.68E-07	26.2656

M01585.m	rs2993308	A	G	0.338	0.0075	0.0017	6.83E-06	19.4637
M01585.m	rs513209	A	C	0.8824	-0.0082	0.0018	6.6E-06	20.7531
M01585.m	rs6019504	A	G	0.8586	0.008	0.0018	6.69E-06	19.7531
M01585.m	rs6462723	A	G	0.121	-0.0085	0.0018	1.92E-06	22.2994
M01585.m	rs662760	A	C	0.8723	0.0079	0.0018	8.04E-06	19.2623
M01585.m	rs6690829	T	C	0.24	-0.008	0.0016	1.24E-06	25
M01585.m	rs7178551	T	C	0.1545	0.0081	0.0017	2.66E-06	22.7024
M01585.m	rs7250750	T	G	0.8583	0.0081	0.0018	7.03E-06	20.25
M01585.m	rs732426	A	G	0.8218	0.0077	0.0017	7.54E-06	20.5156
M01585.m	rs9663814	A	G	0.6175	0.0076	0.0016	1.95E-06	22.5625
M01604.m	rs1001217	C	G	0.6383	0.0082	0.0016	2.74E-07	26.2656
M01604.m	rs10490734	A	G	0.8314	0.0077	0.0017	7.94E-06	20.5156
M01604.m	rs10516194	T	C	0.9827	0.0269	0.006	6.88E-06	20.1003
M01604.m	rs10844317	T	G	0.1637	-0.0086	0.0018	1.12E-06	22.8272
M01604.m	rs10939663	T	G	0.7279	-0.0161	0.0017	5.57E-22	89.692
M01604.m	rs11130013	T	C	0.153	-0.0081	0.0018	5.09E-06	20.25
M01604.m	rs11731100	A	T	0.3502	-0.0116	0.0016	9.97E-13	52.5625
M01604.m	rs11737601	A	G	0.6847	0.0104	0.0016	1.34E-10	42.25
M01604.m	rs11741189	T	C	0.177	-0.0087	0.0017	3.78E-07	26.1903
M01604.m	rs12579148	T	C	0.1887	-0.0077	0.0017	5.92E-06	20.5156
M01604.m	rs1286087	T	C	0.8367	0.0077	0.0017	8.08E-06	20.5156
M01604.m	rs13318012	T	C	0.8378	-0.0078	0.0017	8.36E-06	21.0519
M01604.m	rs16953268	A	C	0.0689	-0.0115	0.0026	7.73E-06	19.5636
M01604.m	rs1825043	A	G	0.9571	0.019	0.0038	5.26E-07	25
M01604.m	rs2231142	T	G	0.1106	0.0119	0.0024	8.43E-07	24.5851
M01604.m	rs233340	A	G	0.0707	-0.0125	0.0026	2.54E-06	23.1139
M01604.m	rs235320	A	G	0.8241	-0.0079	0.0017	3.68E-06	21.5952
M01604.m	rs4697910	A	G	0.2866	-0.0124	0.0017	8.72E-14	53.2042
M01604.m	rs510032	T	C	0.169	0.009	0.002	6.8E-06	20.25
M01604.m	rs6731691	A	G	0.1705	-0.008	0.0018	5.3E-06	19.7531
M01604.m	rs6927639	A	G	0.8458	-0.0078	0.0018	9.27E-06	18.7778
M01604.m	rs7906282	T	G	0.3075	0.008	0.0016	1.11E-06	25
M01604.m	rs872114	A	C	0.9736	-0.0208	0.0044	2.29E-06	22.3471
M01604.m	rs938554	C	G	0.2256	-0.0348	0.0017	5.85E-93	419.045
M01604.m	rs9549021	T	C	0.6112	-0.0135	0.0029	2.94E-06	21.6706
M01604.m	rs9557545	A	T	0.032	0.018	0.004	7.83E-06	20.25
M01605.m	rs10174534	A	G	0.5571	-0.028	0.0063	7.57E-06	19.7531
M01605.m	rs11736497	T	C	0.0214	0.1146	0.0252	5.56E-06	20.6808
M01605.m	rs12587564	A	G	0.1148	0.0456	0.0097	2.63E-06	22.0997
M01605.m	rs17118371	T	C	0.2298	0.0343	0.0071	1.44E-06	23.3384
M01605.m	rs2421668	A	G	0.644	0.034	0.0073	2.87E-06	21.6926
M01605.m	rs2720445	T	C	0.536	0.033	0.0073	5.56E-06	20.4354
M01605.m	rs312306	C	G	0.2278	-0.0654	0.0145	6.23E-06	20.3432
M01605.m	rs3760877	T	C	0.4762	0.0577	0.013	9.55E-06	19.6999
M01605.m	rs6763802	T	G	0.0798	-0.0576	0.0116	6.5E-07	24.6564
M01605.m	rs7463380	C	G	0.8421	0.0763	0.0169	6.4E-06	20.3834
M01605.m	rs9573265	T	C	0.0158	0.2216	0.0488	5.55E-06	20.6205
M01638.m	rs10922038	T	C	0.4354	0.0102	0.0023	7.57E-06	19.6673
M01638.m	rs11114987	T	C	0.0439	-0.0324	0.0067	1.41E-06	23.3852
M01638.m	rs1153903	A	G	0.3856	-0.0106	0.0023	4.19E-06	21.2401
M01638.m	rs12714263	A	G	0.5733	-0.0107	0.0023	3.98E-06	21.6427
M01638.m	rs12985777	T	C	0.2402	-0.0141	0.0032	8.86E-06	19.415
M01638.m	rs17706531	A	G	0.6331	0.0105	0.0023	6.97E-06	20.8412
M01638.m	rs2180566	T	C	0.6433	-0.0109	0.0023	2.11E-06	22.4594
M01638.m	rs2246012	T	C	0.8388	-0.0166	0.0032	2.23E-07	26.9102

M01638.m	rs2916148	A	G	0.4534	-0.0101	0.0023	9.24E-06	19.2836
M01638.m	rs2933871	A	G	0.6938	0.0178	0.004	7.65E-06	19.8025
M01638.m	rs3011362	A	G	0.0334	-0.0349	0.0074	2.37E-06	22.2427
M01638.m	rs6479442	A	C	0.4837	0.0101	0.0023	9.59E-06	19.2836
M01638.m	rs6920504	T	C	0.7729	-0.0135	0.003	7.53E-06	20.25
M01638.m	rs7036499	T	C	0.8519	0.0217	0.0049	8.86E-06	19.6122
M01638.m	rs715	T	C	0.7064	0.0144	0.0028	2.9E-07	26.449
M01638.m	rs720118	T	C	0.6152	-0.0105	0.0023	4.55E-06	20.8412
M01638.m	rs7535809	A	G	0.8331	0.0138	0.0031	7.28E-06	19.8169
M01638.m	rs7717247	A	C	0.8061	0.0162	0.0035	3.35E-06	21.4237
M01638.m	rs837763	T	C	0.5528	0.0114	0.0025	6.03E-06	20.7936
M01638.m	rs901272	A	C	0.6207	-0.0105	0.0023	6.49E-06	20.8412
M01640.m	rs11167905	T	C	0.8545	0.0804	0.0164	9.83E-07	24.0339
M01640.m	rs13069990	T	C	0.3781	-0.0506	0.011	4.44E-06	21.16
M01640.m	rs13103690	T	G	0.5359	-0.0474	0.0104	5.2E-06	20.7726
M01640.m	rs2070006	T	C	0.3713	0.0512	0.0112	4.76E-06	20.898
M01640.m	rs577596	A	G	0.3267	-0.0567	0.0114	6.68E-07	24.7375
M01640.m	rs6713914	T	C	0.5741	0.0593	0.0116	3.22E-07	26.1332
M01640.m	rs6826474	T	C	0.0393	-0.1383	0.0288	1.56E-06	23.06
M01640.m	rs6834631	T	G	0.9596	0.1306	0.0267	1.03E-06	23.9257
M01640.m	rs7112460	T	C	0.0658	0.1084	0.0223	1.14E-06	23.6292
M01640.m	rs8057559	T	C	0.0312	0.1395	0.0314	9.1E-06	19.7374
M01640.m	rs808686	A	G	0.6086	0.0598	0.0128	3.01E-06	21.8264
M01640.m	rs8105491	T	G	0.154	-0.0698	0.0148	2.3E-06	22.2427
M01640.m	rs9419004	C	G	0.186	-0.254	0.0563	6.53E-06	20.354
M01640.m	rs9606290	A	G	0.241	0.159	0.0352	6.32E-06	20.4037
M01644.m	rs10092877	T	C	0.4173	0.0087	0.0018	1.25E-06	23.3611
M01644.m	rs10153405	A	G	0.5342	-0.0103	0.0018	9.27E-09	32.7438
M01644.m	rs10266726	T	C	0.4261	-0.0083	0.0018	4E-06	21.2623
M01644.m	rs10492225	T	C	0.8498	-0.0128	0.0027	1.6E-06	22.4746
M01644.m	rs10740323	T	C	0.5935	0.0084	0.0018	4.1E-06	21.7778
M01644.m	rs10848196	A	G	0.5584	-0.0098	0.0018	5.25E-08	29.642
M01644.m	rs11872932	A	T	0.201	0.0128	0.0027	1.49E-06	22.4746
M01644.m	rs12953375	A	T	0.8162	0.0126	0.0026	1.34E-06	23.4852
M01644.m	rs13002121	A	G	0.766	0.0115	0.0026	6.94E-06	19.5636
M01644.m	rs13083714	T	C	0.5762	0.0194	0.0042	4.84E-06	21.3356
M01644.m	rs13109093	A	C	0.614	0.0085	0.0018	4.25E-06	22.2994
M01644.m	rs17002476	T	C	0.1459	-0.0119	0.0027	8.32E-06	19.4252
M01644.m	rs17287933	T	C	0.4687	0.0083	0.0018	5.24E-06	21.2623
M01644.m	rs17520583	A	G	0.332	0.0093	0.0018	3.6E-07	26.6944
M01644.m	rs17739107	A	G	0.942	-0.0198	0.0044	8.09E-06	20.25
M01644.m	rs1836862	A	C	0.6051	-0.008	0.0018	9.37E-06	19.7531
M01644.m	rs1866411	A	C	0.3473	-0.0083	0.0018	5.23E-06	21.2623
M01644.m	rs1889463	T	C	0.628	0.0089	0.0019	3.36E-06	21.9418
M01644.m	rs203591	T	C	0.3697	-0.0086	0.0018	2.01E-06	22.8272
M01644.m	rs2073805	T	C	0.407	-0.009	0.002	6.8E-06	20.25
M01644.m	rs2091331	A	G	0.4127	0.0082	0.0018	5.71E-06	20.7531
M01644.m	rs2291199	A	G	0.5373	0.0083	0.0018	5.18E-06	21.2623
M01644.m	rs269194	A	C	0.4386	-0.0081	0.0018	9.01E-06	20.25
M01644.m	rs4618485	A	G	0.595	-0.0085	0.0018	3.04E-06	22.2994
M01644.m	rs498266	A	G	0.5852	0.0082	0.0018	6.15E-06	20.7531
M01644.m	rs6051643	A	G	0.986	0.0441	0.0095	3.76E-06	21.5491
M01644.m	rs630782	T	C	0.4713	0.0093	0.0018	2.32E-07	26.6944
M01644.m	rs676094	A	G	0.4178	0.0191	0.0042	6.61E-06	20.6808
M01644.m	rs6860349	A	G	0.4108	-0.0089	0.0018	8.67E-07	24.4475

M01644.m	rs7436874	T	C	0.6135	0.0083	0.0019	9.89E-06	19.0831
M01644.m	rs8006663	A	G	0.4556	-0.0086	0.0018	1.79E-06	22.8272
M01644.m	rs8079173	A	G	0.1402	0.0118	0.0026	8.93E-06	20.5976
M01644.m	rs864428	T	C	0.4058	0.0101	0.0018	2.99E-08	31.4846
M01644.m	rs9607028	T	G	0.4268	-0.0084	0.0019	7.29E-06	19.5457
M01644.m	rs989248	A	G	0.6475	-0.0085	0.0018	2.96E-06	22.2994
M01644.m	rs990054	T	C	0.5955	-0.0087	0.0018	1.33E-06	23.3611
M01644.m	rs9997602	A	C	0.5717	-0.0081	0.0018	7.57E-06	20.25
M01645.m	rs10039731	A	G	0.5787	0.0198	0.004	8.47E-07	24.5025
M01645.m	rs11036901	A	G	0.433	-0.0079	0.0018	7.86E-06	19.2623
M01645.m	rs11193635	T	C	0.6725	0.0084	0.0018	3.8E-06	21.7778
M01645.m	rs11589855	T	C	0.3809	-0.0084	0.0018	2.22E-06	21.7778
M01645.m	rs11731117	A	G	0.5958	0.0081	0.0018	4.32E-06	20.25
M01645.m	rs11857879	T	G	0.1952	0.0112	0.0025	8.18E-06	20.0704
M01645.m	rs11978728	A	T	0.6876	-0.0089	0.0018	5.18E-07	24.4475
M01645.m	rs12466053	A	G	0.402	0.0087	0.0018	8.44E-07	23.3611
M01645.m	rs12862464	T	C	0.0842	0.0146	0.0033	9.83E-06	19.5739
M01645.m	rs12940605	A	C	0.6059	-0.0096	0.0018	1.82E-07	28.4444
M01645.m	rs13393013	A	G	0.0213	0.0314	0.0065	1.31E-06	23.3363
M01645.m	rs1487052	T	C	0.3063	0.008	0.0018	7.66E-06	19.7531
M01645.m	rs1530439	T	G	0.2946	0.009	0.0018	6.08E-07	25
M01645.m	rs16942446	A	G	0.0314	-0.0259	0.0056	3.32E-06	21.3906
M01645.m	rs16990450	T	C	0.0722	0.0161	0.0035	4.18E-06	21.16
M01645.m	rs2069151	A	G	0.7083	0.009	0.0018	4.68E-07	25
M01645.m	rs2140495	T	C	0.0158	0.0638	0.0144	9.51E-06	19.6298
M01645.m	rs2255059	T	C	0.5192	-0.008	0.0018	7.4E-06	19.7531
M01645.m	rs2304767	A	G	0.3402	0.0091	0.0019	1.22E-06	22.9391
M01645.m	rs2423542	A	G	0.8036	0.0153	0.0032	1.91E-06	22.8604
M01645.m	rs2491274	A	G	0.9732	-0.0297	0.0065	4.63E-06	20.8779
M01645.m	rs287276	A	G	0.3317	0.0084	0.0018	2.65E-06	21.7778
M01645.m	rs369895	A	G	0.574	-0.0114	0.0023	1.08E-06	24.5671
M01645.m	rs4327548	A	G	0.2904	-0.0093	0.0018	2.16E-07	26.6944
M01645.m	rs4333677	A	G	0.5848	0.008	0.0018	5.99E-06	19.7531
M01645.m	rs4680878	T	G	0.6552	0.0079	0.0018	9.16E-06	19.2623
M01645.m	rs4789698	T	C	0.4508	-0.0079	0.0018	6.83E-06	19.2623
M01645.m	rs4930046	A	G	0.4652	0.0088	0.0019	2.72E-06	21.4515
M01645.m	rs6112257	A	G	0.6843	-0.0092	0.0018	3.39E-07	26.1235
M01645.m	rs6441206	A	G	0.4638	0.0099	0.0018	2.09E-08	30.25
M01645.m	rs6502896	A	G	0.7619	-0.0187	0.0042	9.7E-06	19.8237
M01645.m	rs6765113	T	C	0.5408	0.0082	0.0018	8.16E-06	20.7531
M01645.m	rs696979	T	C	0.6026	0.0084	0.0018	2.05E-06	21.7778
M01645.m	rs6973824	A	G	0.3374	0.009	0.0018	4.02E-07	25
M01645.m	rs699015	A	G	0.955	0.0391	0.0089	9.93E-06	19.3007
M01645.m	rs7011528	A	C	0.4515	-0.0082	0.0018	4.01E-06	20.7531
M01645.m	rs853907	T	C	0.4778	0.0079	0.0018	7.64E-06	19.2623
M01645.m	rs891531	T	G	0.1846	0.0113	0.0024	3.81E-06	22.1684
M01645.m	rs9563502	A	G	0.7115	-0.01	0.0018	2.56E-08	30.8642
M01645.m	rs9815886	A	C	0.4756	-0.0083	0.0018	2.55E-06	21.2623
M01645.m	rs9852606	C	G	0.3771	-0.0085	0.0018	1.48E-06	22.2994
M01649.m	rs10050054	T	C	0.9828	-0.0336	0.0072	3.18E-06	21.7778
M01649.m	rs10181551	A	G	0.296	-0.0055	0.0012	3.88E-06	21.0069
M01649.m	rs12552913	A	T	0.2932	-0.0059	0.0012	1.16E-06	24.1736
M01649.m	rs1440581	T	C	0.4577	-0.0081	0.0012	2.56E-12	45.5625
M01649.m	rs2373642	A	G	0.0138	-0.054	0.011	9.78E-07	24.0992
M01649.m	rs3762466	T	C	0.6819	0.0058	0.0012	1.06E-06	23.3611

M01670.m	rs10075187	T	G	0.9687	0.0316	0.0068	3.44E-06	21.5952
M01670.m	rs12654812	A	G	0.2913	0.0118	0.0025	3.36E-06	22.2784
M01670.m	rs1569823	T	C	0.8757	-0.0152	0.0034	8.25E-06	19.9862
M01670.m	rs1792544	A	G	0.8963	-0.0155	0.0034	5.83E-06	20.7829
M01670.m	rs2049664	C	G	0.1916	0.0123	0.0026	3.01E-06	22.3802
M01670.m	rs2170700	A	G	0.8663	0.0326	0.0069	2.43E-06	22.3222
M01670.m	rs2424200	T	C	0.1163	0.0152	0.0034	6.43E-06	19.9862
M01670.m	rs6074893	A	C	0.7512	-0.0115	0.0026	8.52E-06	19.5636
M01670.m	rs6753621	A	G	0.2183	-0.0117	0.0026	7.02E-06	20.25
M01670.m	rs686346	T	C	0.7658	0.0128	0.0027	1.44E-06	22.4746
M01670.m	rs7739842	T	G	0.8202	0.0134	0.0027	8E-07	24.631
M01670.m	rs7990832	A	G	0.1792	0.0122	0.0026	3.87E-06	22.0178
M01670.m	rs9646626	T	C	0.948	-0.0226	0.0051	7.98E-06	19.6371
M01670.m	rs980922	C	G	0.8056	-0.012	0.0026	3.57E-06	21.3018
M01712.m	rs1010874	A	G	0.0629	-0.0491	0.0096	3.17E-07	26.159
M01712.m	rs11013837	A	T	0.2463	-0.015	0.0034	8.16E-06	19.4637
M01712.m	rs1173842	A	G	0.567	-0.0217	0.0049	8.34E-06	19.6122
M01712.m	rs11855136	A	G	0.0406	-0.0618	0.0121	2.99E-07	26.0859
M01712.m	rs11987983	T	C	0.099	-0.0457	0.0102	7.16E-06	20.0739
M01712.m	rs12210538	A	G	0.8442	0.0254	0.0057	9.19E-06	19.8572
M01712.m	rs12883490	T	C	0.6656	0.0166	0.0033	3.76E-07	25.3039
M01712.m	rs1381274	T	C	0.4672	-0.0146	0.0032	3.63E-06	20.8164
M01712.m	rs1448767	T	C	0.6289	-0.0144	0.0033	9.95E-06	19.0413
M01712.m	rs1491606	A	T	0.2992	0.0182	0.004	5.8E-06	20.7025
M01712.m	rs4439706	T	C	0.7156	0.0151	0.0033	3.78E-06	20.9376
M01712.m	rs9446392	T	C	0.9533	-0.0331	0.0075	9.27E-06	19.4775
M01712.m	rs9533798	C	G	0.3667	-0.0142	0.0032	8.36E-06	19.6914
M01769.m	rs10102742	T	C	0.4035	0.0078	0.0018	8.73E-06	18.7778
M01769.m	rs10454657	T	C	0.5849	0.0083	0.0017	1.64E-06	23.8374
M01769.m	rs10824300	A	G	0.383	0.009	0.002	6.8E-06	20.25
M01769.m	rs10851734	A	G	0.6345	0.0078	0.0017	6.05E-06	21.0519
M01769.m	rs10938815	T	G	0.5932	0.0099	0.0017	8.38E-09	33.9135
M01769.m	rs1115509	A	C	0.0138	-0.0999	0.0211	2.13E-06	22.4164
M01769.m	rs11532668	A	G	0.8339	0.0168	0.0038	9.75E-06	19.5457
M01769.m	rs1173830	T	C	0.6542	-0.0171	0.0034	6.6E-07	25.295
M01769.m	rs13280205	A	C	0.805	0.0121	0.0025	2.02E-06	23.4256
M01769.m	rs1334235	C	G	0.3644	0.0077	0.0017	9.97E-06	20.5156
M01769.m	rs1438564	T	C	0.8089	0.0114	0.0025	5.1E-06	20.7936
M01769.m	rs17248331	T	C	0.5848	-0.0089	0.0017	1.7E-07	27.4083
M01769.m	rs2058473	A	G	0.6494	-0.0077	0.0017	7.48E-06	20.5156
M01769.m	rs2118096	A	C	0.6068	0.0084	0.0017	1.13E-06	24.4152
M01769.m	rs219918	C	G	0.0224	-0.0299	0.0063	2.48E-06	22.5248
M01769.m	rs2272663	A	G	0.5321	0.0077	0.0017	9.39E-06	20.5156
M01769.m	rs227421	T	C	0.1489	0.0116	0.0025	4.04E-06	21.5296
M01769.m	rs2325727	T	C	0.4423	-0.0076	0.0017	9.34E-06	19.9862
M01769.m	rs2351174	T	G	0.6063	-0.0081	0.0017	2.63E-06	22.7024
M01769.m	rs2364598	A	C	0.149	-0.014	0.003	3.06E-06	21.7778
M01769.m	rs3095571	A	G	0.4465	0.0078	0.0017	6.74E-06	21.0519
M01769.m	rs4470135	A	C	0.5203	0.0088	0.0018	5.17E-07	23.9012
M01769.m	rs4653629	T	C	0.5584	-0.0084	0.0017	1.25E-06	24.4152
M01769.m	rs4717222	A	C	0.4686	-0.0086	0.0017	4.34E-07	25.5917
M01769.m	rs4930767	T	C	0.4862	-0.0079	0.0017	3.69E-06	21.5952
M01769.m	rs527793	T	G	0.1085	-0.0149	0.0033	4.67E-06	20.3866
M01769.m	rs6017596	T	C	0.5825	-0.0084	0.0017	8.31E-07	24.4152
M01769.m	rs6539409	T	G	0.5663	-0.0078	0.0017	4.95E-06	21.0519

M01769.m	rs6910731	A	G	0.4495	0.0087	0.0018	9.06E-07	23.3611
M01769.m	rs7562418	A	C	0.5075	0.0077	0.0017	6.81E-06	20.5156
M01769.m	rs7661472	A	C	0.4922	-0.0082	0.0017	1.38E-06	23.2664
M01769.m	rs7670890	A	G	0.6344	-0.0076	0.0017	9.64E-06	19.9862
M01769.m	rs7814638	A	G	0.141	-0.0133	0.0025	1.23E-07	28.3024
M01769.m	rs7837792	T	C	0.5936	-0.0081	0.0017	2.78E-06	22.7024
M01769.m	rs7915877	T	C	0.5509	-0.0078	0.0017	4.42E-06	21.0519
M01769.m	rs9379820	T	C	0.5914	-0.0076	0.0017	8.32E-06	19.9862
M01769.m	rs9391679	A	C	0.157	0.0123	0.0025	5.82E-07	24.2064
M01769.m	rs940511	A	G	0.4652	-0.0077	0.0017	6.71E-06	20.5156
M01769.m	rs960381	A	C	0.5958	0.008	0.0017	3.56E-06	22.1453
M01769.m	rs9674799	A	G	0.8784	0.0157	0.0032	7.47E-07	24.0713
M01898.m	rs10183099	A	G	0.6391	-0.0084	0.0018	2.44E-06	21.7778
M01898.m	rs10501294	T	C	0.8715	-0.0124	0.0027	3.11E-06	21.0919
M01898.m	rs10512634	A	G	0.1405	0.0117	0.0026	8.59E-06	20.25
M01898.m	rs10747856	A	G	0.5243	-0.0079	0.0017	6.03E-06	21.5952
M01898.m	rs10954090	T	C	0.1522	0.013	0.0026	3.97E-07	25
M01898.m	rs11075967	T	C	0.6699	-0.0087	0.0018	8.67E-07	23.3611
M01898.m	rs11183620	A	G	0.4679	0.0082	0.0017	2.34E-06	23.2664
M01898.m	rs11784679	T	G	0.7763	-0.0116	0.0025	3.62E-06	21.5296
M01898.m	rs11848063	A	G	0.5861	0.0087	0.0018	7.14E-07	23.3611
M01898.m	rs12243290	A	G	0.8997	-0.0157	0.0034	0.000004	21.3227
M01898.m	rs1394836	T	C	0.6032	0.0083	0.0018	2.23E-06	21.2623
M01898.m	rs1564080	A	T	0.324	0.0079	0.0018	7.53E-06	19.2623
M01898.m	rs1615586	T	G	0.7674	-0.0196	0.0043	4.18E-06	20.7766
M01898.m	rs175866	T	G	0.5784	-0.0086	0.0018	1.14E-06	22.8272
M01898.m	rs1888182	T	C	0.4159	0.0079	0.0018	7.93E-06	19.2623
M01898.m	rs2185865	A	C	0.9245	0.0177	0.0034	1.7E-07	27.1012
M01898.m	rs2518802	A	C	0.903	-0.0534	0.0033	4.15E-59	261.851
M01898.m	rs2935623	A	G	0.6235	-0.0079	0.0018	8.71E-06	19.2623
M01898.m	rs4580644	T	C	0.4937	0.0157	0.0035	8.16E-06	20.1216
M01898.m	rs4978836	A	G	0.391	0.009	0.002	6.8E-06	20.25
M01898.m	rs535633	A	G	0.5847	-0.0086	0.0018	1.05E-06	22.8272
M01898.m	rs6085132	T	C	0.9725	-0.0272	0.0059	4.01E-06	21.2537
M01898.m	rs6769362	C	G	0.6423	-0.0079	0.0018	7.76E-06	19.2623
M01898.m	rs6798346	A	G	0.627	-0.0167	0.0038	8.78E-06	19.3137
M01898.m	rs7029664	T	C	0.5057	0.0077	0.0017	9.66E-06	20.5156
M01898.m	rs715544	A	G	0.093	-0.0199	0.0036	2.44E-08	30.5563
M01898.m	rs7208186	T	C	0.8619	0.0139	0.0026	6.26E-08	28.5814
M01898.m	rs7296105	A	C	0.8773	0.0262	0.0055	2.35E-06	22.6922
M01898.m	rs7560860	A	G	0.4253	0.0079	0.0018	6.43E-06	19.2623
M01898.m	rs7858706	T	C	0.4952	-0.0077	0.0017	9.66E-06	20.5156
M01898.m	rs893175	A	C	0.5884	-0.0086	0.0018	1.53E-06	22.8272
M01898.m	rs9360494	T	C	0.5977	-0.0083	0.0018	2.69E-06	21.2623
M01898.m	rs950755	T	C	0.8596	-0.0116	0.0026	8.51E-06	19.9053
M01898.m	rs9540462	T	G	0.8756	-0.012	0.0026	4.8E-06	21.3018
M01898.m	rs9605907	A	G	0.1623	-0.0229	0.0026	9.7E-19	77.5754
M01898.m	rs9680846	T	C	0.2825	-0.0144	0.0024	2.8E-09	36
M02132.m	rs10164524	A	G	0.6775	-0.01	0.0017	1.06E-08	34.6021
M02132.m	rs10234187	T	C	0.3185	0.0085	0.0017	1.07E-06	25
M02132.m	rs10477454	T	C	0.0169	-0.0374	0.0082	4.65E-06	20.8025
M02132.m	rs10489008	A	C	0.4919	-0.0076	0.0017	7.37E-06	19.9862
M02132.m	rs10858075	T	C	0.4061	-0.0091	0.0017	1.12E-07	28.654
M02132.m	rs10901047	T	C	0.5877	-0.009	0.0018	2.96E-07	25
M02132.m	rs10901320	A	G	0.4313	0.0087	0.0017	4.34E-07	26.1903

M02132.m	rs11119951	T	C	0.1175	0.0119	0.0026	4.36E-06	20.9482
M02132.m	rs11993007	A	G	0.3167	-0.0127	0.0028	5.24E-06	20.5727
M02132.m	rs12147171	C	G	0.1169	-0.0118	0.0026	4.76E-06	20.5976
M02132.m	rs12701265	A	G	0.3919	0.0086	0.0017	7.37E-07	25.5917
M02132.m	rs13105113	A	C	0.8769	0.0114	0.0026	9.18E-06	19.2249
M02132.m	rs13208172	T	C	0.9562	0.0216	0.0049	9.92E-06	19.4319
M02132.m	rs13267180	A	G	0.4794	0.0081	0.0017	3.78E-06	22.7024
M02132.m	rs1437588	A	G	0.479	0.0078	0.0018	7.67E-06	18.7778
M02132.m	rs1509820	A	G	0.5299	0.0096	0.0017	1.75E-08	31.8893
M02132.m	rs1511589	A	G	0.4559	0.0077	0.0017	7.55E-06	20.5156
M02132.m	rs1610360	A	T	0.6678	0.008	0.0018	4.63E-06	19.7531
M02132.m	rs16841570	T	C	0.9726	0.0248	0.0056	8.59E-06	19.6122
M02132.m	rs17142889	T	G	0.1339	0.0111	0.0025	9.91E-06	19.7136
M02132.m	rs172139	A	G	0.6401	0.0083	0.0017	1.55E-06	23.8374
M02132.m	rs17681684	A	G	0.3158	0.0085	0.0017	1.04E-06	25
M02132.m	rs17821771	T	G	0.3485	-0.0082	0.0017	1.92E-06	23.2664
M02132.m	rs1920325	T	C	0.6425	-0.0081	0.0017	3.66E-06	22.7024
M02132.m	rs1935815	A	T	0.3513	0.0111	0.0017	1.55E-10	42.6332
M02132.m	rs229084	A	G	0.4727	-0.0077	0.0017	6.57E-06	20.5156
M02132.m	rs2350885	A	T	0.3481	-0.0079	0.0018	8.72E-06	19.2623
M02132.m	rs2487410	T	C	0.4696	-0.0081	0.0017	1.84E-06	22.7024
M02132.m	rs2506864	T	G	0.4103	-0.0076	0.0017	8.81E-06	19.9862
M02132.m	rs310101	A	G	0.9579	0.0367	0.0079	3.86E-06	21.5813
M02132.m	rs4768769	T	C	0.6012	-0.0078	0.0017	6.64E-06	21.0519
M02132.m	rs539904	A	G	0.7056	-0.0086	0.0018	1.51E-06	22.8272
M02132.m	rs554710	T	C	0.7074	-0.0084	0.0018	1.59E-06	21.7778
M02132.m	rs6040877	A	G	0.2715	-0.0081	0.0018	4.01E-06	20.25
M02132.m	rs6510448	T	G	0.6691	0.0077	0.0017	8.14E-06	20.5156
M02132.m	rs682103	T	C	0.7055	-0.0097	0.0018	4.26E-08	29.0401
M02132.m	rs6889120	T	C	0.9378	-0.034	0.0076	7.44E-06	20.0139
M02132.m	rs6994363	A	G	0.8662	-0.0121	0.0026	2.5E-06	21.6583
M02132.m	rs7012395	T	C	0.3041	-0.0081	0.0018	4.96E-06	20.25
M02132.m	rs758186	T	C	0.6536	-0.0077	0.0017	8.69E-06	20.5156
M02132.m	rs7597477	A	G	0.9327	-0.0155	0.0034	6.8E-06	20.7829
M02132.m	rs7615965	T	C	0.7121	0.0086	0.0019	4.15E-06	20.4875
M02132.m	rs779463	T	G	0.3012	-0.0077	0.0017	9.46E-06	20.5156
M02132.m	rs8021336	A	G	0.313	-0.0086	0.0018	9.89E-07	22.8272
M02132.m	rs8033140	T	C	0.3547	0.008	0.0017	4.32E-06	22.1453
M02132.m	rs879126	T	G	0.3286	-0.0086	0.0017	7.47E-07	25.5917
M02132.m	rs9354336	T	C	0.8733	0.0113	0.0025	8.57E-06	20.4304
M02132.m	rs9400834	T	C	0.7024	0.008	0.0018	5.62E-06	19.7531
M02137.m	rs10170160	T	C	0.5675	0.0338	0.0057	2.71E-09	35.1628
M02137.m	rs1019595	T	C	0.9326	-0.0345	0.0076	5.46E-06	20.6068
M02137.m	rs1076454	T	C	0.1081	0.0325	0.0065	5.65E-07	25
M02137.m	rs12371604	T	C	0.7709	-0.0205	0.0043	2.18E-06	22.7285
M02137.m	rs12496969	A	G	0.7844	0.0202	0.0044	4.28E-06	21.0764
M02137.m	rs12615708	A	G	0.0251	0.0591	0.0124	1.94E-06	22.716
M02137.m	rs13383300	C	G	0.9722	0.054	0.0113	1.91E-06	22.8366
M02137.m	rs17863762	A	G	0.0288	-0.0632	0.0119	1.19E-07	28.2059
M02137.m	rs17864661	T	C	0.0891	0.054	0.007	1.56E-14	59.5102
M02137.m	rs17864714	A	G	0.0342	0.0523	0.0117	8.41E-06	19.9817
M02137.m	rs17868378	A	C	0.932	-0.034	0.0075	6.49E-06	20.5511
M02137.m	rs1891053	A	G	0.0145	0.1831	0.0371	8.03E-07	24.3573
M02137.m	rs2219067	C	G	0.7612	0.0397	0.0044	1.34E-19	81.4096
M02137.m	rs2349945	T	C	0.3863	-0.0347	0.0077	7.25E-06	20.3085

M02137.m	rs2760277	A	G	0.7946	0.0197	0.0044	8.26E-06	20.046
M02137.m	rs280194	T	C	0.7311	-0.0197	0.0043	5.77E-06	20.9892
M02137.m	rs28900371	A	G	0.0352	0.089	0.01	7.65E-19	79.21
M02137.m	rs28948069	C	G	0.8649	-0.0317	0.006	1.05E-07	27.9136
M02137.m	rs2924450	C	G	0.1956	-0.0272	0.005	7.08E-08	29.5936
M02137.m	rs4944238	A	G	0.8712	-0.0241	0.0053	4.88E-06	20.6768
M02137.m	rs4964090	A	G	0.5654	-0.019	0.0036	1.18E-07	27.8549
M02137.m	rs6503066	T	C	0.0984	0.0317	0.0065	9.49E-07	23.7844
M02137.m	rs6717546	A	G	0.3648	-0.0395	0.0036	3.93E-28	120.39
M02137.m	rs6823453	A	G	0.2233	0.0231	0.0044	1.78E-07	27.5625
M02137.m	rs6837370	A	G	0.1004	0.0645	0.0122	1.15E-07	27.9512
M02137.m	rs7045137	A	G	0.3947	-0.0169	0.0037	4E-06	20.8627
M02137.m	rs741382	T	C	0.2234	-0.0472	0.0093	3.74E-07	25.7584
M02137.m	rs7440035	T	G	0.7877	-0.0405	0.0091	9.29E-06	19.8074
M02137.m	rs7608713	A	G	0.24	-0.0415	0.0044	7.35E-21	88.9592
M02137.m	rs838705	A	G	0.6134	0.0266	0.0036	1.23E-13	54.5957
M02137.m	rs887829	T	C	0.3411	0.1131	0.0041	2.5E-168	760.952
M02137.m	rs988344	A	C	0.1251	0.0329	0.0059	2.4E-08	31.0948
M02342.m	rs10516056	T	C	0.1978	-0.0222	0.0045	6.99E-07	24.3378
M02342.m	rs10881427	T	G	0.5558	-0.015	0.0032	3.69E-06	21.9727
M02342.m	rs10953038	T	C	0.8497	0.022	0.0048	4.12E-06	21.0069
M02342.m	rs11781043	T	C	0.6731	-0.0187	0.0042	8.81E-06	19.8237
M02342.m	rs12646122	T	C	0.0308	-0.0497	0.0104	1.8E-06	22.8374
M02342.m	rs17708932	A	G	0.0376	-0.0766	0.0171	7.22E-06	20.0662
M02342.m	rs2036136	T	C	0.4587	-0.0256	0.0056	5.11E-06	20.898
M02342.m	rs2742351	C	G	0.2209	-0.0235	0.0046	3.23E-07	26.0988
M02342.m	rs3753556	T	C	0.4325	0.015	0.0032	3.66E-06	21.9727
M02342.m	rs5764174	T	C	0.2445	-0.0194	0.004	9.61E-07	23.5225
M02342.m	rs6466265	A	G	0.7977	0.0186	0.004	4.15E-06	21.6225
M02342.m	rs7868774	A	G	0.2709	-0.0188	0.0039	1.78E-06	23.2373
M02342.m	rs8113773	A	G	0.5616	0.016	0.0033	1.12E-06	23.5078
M02342.m	rs825278	T	C	0.0201	-0.0913	0.0192	2.06E-06	22.612
M02342.m	rs988694	A	G	0.477	-0.0145	0.0033	8.39E-06	19.3067
M02342.m	rs9953702	A	G	0.3601	0.0146	0.0033	9.22E-06	19.5739
M02730.m	rs10432570	A	G	0.0145	0.0738	0.0138	9.5E-08	28.5992
M02730.m	rs10497174	T	C	0.2732	0.0103	0.0022	4.16E-06	21.9194
M02730.m	rs11151406	A	T	0.9444	0.0301	0.0066	5.61E-06	20.7991
M02730.m	rs1260326	T	C	0.4122	-0.0121	0.0021	5.61E-09	33.1995
M02730.m	rs12737843	T	C	0.7976	0.012	0.0023	1.96E-07	27.2212
M02730.m	rs17041868	T	C	0.939	0.0196	0.0042	4.02E-06	21.7778
M02730.m	rs1751956	T	G	0.8647	0.016	0.0029	2.35E-08	30.44
M02730.m	rs17602430	T	C	0.079	0.0172	0.0036	1.57E-06	22.8272
M02730.m	rs1832664	T	C	0.2818	-0.0097	0.0022	7.74E-06	19.4401
M02730.m	rs192598	A	C	0.3185	0.0103	0.0022	1.99E-06	21.9194
M02730.m	rs2085545	T	G	0.2883	0.0098	0.0022	9.86E-06	19.843
M02730.m	rs2657879	A	G	0.8262	0.0238	0.0027	5.85E-19	77.701
M02730.m	rs3094600	A	G	0.2637	-0.0099	0.0022	6.21E-06	20.25
M02730.m	rs35592	T	C	0.7618	0.0107	0.0022	1.24E-06	23.655
M02730.m	rs3931670	C	G	0.2941	0.0099	0.0021	4.34E-06	22.2245
M02730.m	rs4587207	A	G	0.8566	-0.0129	0.0028	3.19E-06	21.2258
M02730.m	rs4880550	T	C	0.1292	0.015	0.003	6.32E-07	25
M02730.m	rs4981468	A	G	0.0509	0.0207	0.0045	4.76E-06	21.16
M02730.m	rs6488270	A	G	0.4076	0.0096	0.0021	4.95E-06	20.898
M02730.m	rs7068278	T	G	0.1968	0.0116	0.0023	6.55E-07	25.4367
M02730.m	rs832404	T	C	0.8684	-0.013	0.0029	7.53E-06	20.0951

M02730.m	rs849039	T	G	0.779	-0.0135	0.0028	1.41E-06	23.2462
M02730.m	rs9436667	T	C	0.1138	0.0135	0.003	7.3E-06	20.25
M02734.m	rs10013737	A	G	0.6839	0.0087	0.0018	7.9E-07	23.3611
M02734.m	rs10498270	A	G	0.6222	0.0088	0.0017	4.73E-07	26.7958
M02734.m	rs10774085	A	C	0.6817	0.008	0.0018	7.6E-06	19.7531
M02734.m	rs11165454	T	C	0.5583	0.0089	0.0017	2.79E-07	27.4083
M02734.m	rs11199176	A	C	0.5221	0.0077	0.0017	9.33E-06	20.5156
M02734.m	rs11776904	C	G	0.1522	0.0214	0.0048	7.47E-06	19.8767
M02734.m	rs12192718	A	T	0.1412	0.0142	0.0025	2.1E-08	32.2624
M02734.m	rs12276109	T	C	0.4352	-0.0084	0.0017	1.56E-06	24.4152
M02734.m	rs12429206	A	G	0.3376	0.0086	0.0018	1.85E-06	22.8272
M02734.m	rs12546042	T	C	0.4197	0.0077	0.0017	8.35E-06	20.5156
M02734.m	rs1340971	T	C	0.6654	-0.008	0.0017	4.66E-06	22.1453
M02734.m	rs1418942	A	C	0.5768	-0.0077	0.0017	9.85E-06	20.5156
M02734.m	rs1512342	T	C	0.3393	0.0095	0.0017	4.66E-08	31.2284
M02734.m	rs151743	T	C	0.426	-0.009	0.002	6.8E-06	20.25
M02734.m	rs1560792	A	G	0.3354	0.009	0.0018	3.33E-07	25
M02734.m	rs17100078	A	G	0.8356	0.0122	0.0025	1.04E-06	23.8144
M02734.m	rs17115614	T	C	0.3631	0.0081	0.0017	3.98E-06	22.7024
M02734.m	rs17558863	A	G	0.955	-0.0222	0.005	8.01E-06	19.7136
M02734.m	rs17593458	C	G	0.8978	0.0144	0.0032	7.09E-06	20.25
M02734.m	rs17616555	A	G	0.1597	-0.0112	0.0025	9.58E-06	20.0704
M02734.m	rs17720371	T	C	0.4737	-0.0078	0.0017	6.33E-06	21.0519
M02734.m	rs1864151	A	C	0.5575	0.0089	0.0017	2.4E-07	27.4083
M02734.m	rs1864151	A	C	0.5575	0.0089	0.0017	2.4E-07	27.4083
M02734.m	rs2111718	A	G	0.8615	-0.0227	0.005	5E-06	20.6116
M02734.m	rs2250515	T	G	0.5346	-0.0083	0.0017	1.6E-06	23.8374
M02734.m	rs2254174	T	C	0.8981	-0.0148	0.0033	7.44E-06	20.1139
M02734.m	rs2396461	T	C	0.121	0.0117	0.0026	8.38E-06	20.25
M02734.m	rs2445976	A	C	0.4652	0.0079	0.0017	6.25E-06	21.5952
M02734.m	rs2504282	A	G	0.9338	0.0191	0.0042	6.7E-06	20.6808
M02734.m	rs2933568	T	C	0.3125	-0.008	0.0018	6.23E-06	19.7531
M02734.m	rs322614	A	G	0.3892	0.0084	0.0017	1.7E-06	24.4152
M02734.m	rs353620	A	G	0.6613	-0.0077	0.0017	9.8E-06	20.5156
M02734.m	rs3819535	T	C	0.618	-0.0079	0.0017	5.02E-06	21.5952
M02734.m	rs3936152	T	C	0.6051	0.0079	0.0018	5.92E-06	19.2623
M02734.m	rs4465961	T	C	0.3479	0.0083	0.0018	3.12E-06	21.2623
M02734.m	rs4487134	T	G	0.355	-0.0083	0.0017	1.99E-06	23.8374
M02734.m	rs4572660	T	G	0.6118	-0.0088	0.0017	4.69E-07	26.7958
M02734.m	rs5010651	T	C	0.8332	-0.0132	0.0025	1.77E-07	27.8784
M02734.m	rs5751902	T	C	0.3762	0.012	0.0025	2.38E-06	23.04
M02734.m	rs6456786	T	C	0.3582	-0.008	0.0018	5.67E-06	19.7531
M02734.m	rs8013373	A	G	0.3428	-0.0091	0.0018	4.99E-07	25.5586
M02734.m	rs8132248	T	C	0.0986	0.018	0.0037	1.43E-06	23.6669
M02734.m	rs848767	T	C	0.0696	-0.0179	0.0039	3.46E-06	21.0657
M02734.m	rs875740	A	C	0.6656	0.0102	0.0018	6.35E-09	32.1111
M02734.m	rs9310707	A	G	0.3473	-0.0082	0.0018	6.38E-06	20.7531
M02734.m	rs956966	A	G	0.4858	-0.0101	0.0018	1.48E-08	31.4846
M02734.m	rs9895157	T	C	0.6536	-0.0079	0.0018	6.33E-06	19.2623
M02734.m	rs9940990	A	T	0.3545	-0.0104	0.0018	4.75E-09	33.3827
M03127.m	rs10847679	T	C	0.4091	-0.0176	0.004	9.53E-06	19.36
M03127.m	rs10878072	A	G	0.6887	0.0137	0.003	7.14E-06	20.8544
M03127.m	rs113515	C	G	0.5982	-0.0109	0.0024	8.09E-06	20.6267
M03127.m	rs11595079	T	G	0.0951	-0.0214	0.0045	1.89E-06	22.6153
M03127.m	rs1433818	T	C	0.0808	0.0206	0.0046	6.99E-06	20.0548

M03127.m	rs1587235	A	G	0.3312	-0.0132	0.0029	6.47E-06	20.7182
M03127.m	rs16948294	A	T	0.0694	-0.041	0.0085	1.35E-06	23.2664
M03127.m	rs17280018	A	T	0.2083	-0.0234	0.0052	6.91E-06	20.25
M03127.m	rs1824583	A	G	0.7906	-0.0145	0.0031	3.38E-06	21.8783
M03127.m	rs2113305	T	G	0.2651	-0.0134	0.0029	4.95E-06	21.3508
M03127.m	rs2145985	T	C	0.16	-0.0152	0.0033	3.91E-06	21.2158
M03127.m	rs2247330	T	C	0.6524	0.0124	0.0025	4.56E-07	24.6016
M03127.m	rs275721	A	C	0.509	-0.016	0.003	9.64E-08	28.4444
M03127.m	rs2900071	T	C	0.2178	-0.014	0.0031	7.46E-06	20.3954
M03127.m	rs2939707	A	C	0.5698	0.0109	0.0024	7.39E-06	20.6267
M03127.m	rs3793568	A	T	0.2657	-0.0133	0.003	8.89E-06	19.6544
M03127.m	rs4147184	A	G	0.6082	0.0112	0.0025	6.73E-06	20.0704
M03127.m	rs4676313	T	C	0.8778	0.0179	0.0039	3.54E-06	21.0657
M03127.m	rs4776752	A	G	0.1512	0.0178	0.0039	4.23E-06	20.831
M03127.m	rs6459467	A	G	0.3888	0.0202	0.0025	1.93E-16	65.2864
M03127.m	rs6567408	C	G	0.3922	0.0134	0.003	8.07E-06	19.9511
M03127.m	rs7644390	A	T	0.3478	0.0129	0.0025	1.76E-07	26.6256
M03127.m	rs7924591	T	C	0.5985	0.0108	0.0024	9.52E-06	20.25
M03127.m	rs9379061	C	G	0.9562	-0.0456	0.0099	3.92E-06	21.2158
M03141.m	rs10153066	T	G	0.4356	0.0085	0.0018	2.12E-06	22.2994
M03141.m	rs11943623	A	G	0.1709	-0.0115	0.0026	8.4E-06	19.5636
M03141.m	rs12151827	T	C	0.8159	-0.0141	0.0026	6.35E-08	29.4098
M03141.m	rs12190141	A	G	0.9723	0.0616	0.0139	9.9E-06	19.6396
M03141.m	rs12569219	A	C	0.1612	-0.0118	0.0026	5.82E-06	20.5976
M03141.m	rs13021675	T	C	0.1956	-0.0123	0.0026	1.84E-06	22.3802
M03141.m	rs1535039	T	C	0.912	0.0175	0.0035	4.49E-07	25
M03141.m	rs163124	T	G	0.712	0.0121	0.0024	7.08E-07	25.4184
M03141.m	rs164372	T	C	0.8036	0.012	0.0025	2.64E-06	23.04
M03141.m	rs16876394	T	C	0.8993	0.0304	0.0034	1.49E-19	79.9446
M03141.m	rs17595065	A	G	0.9724	0.0347	0.0078	9.73E-06	19.7911
M03141.m	rs17750747	T	C	0.9165	0.0164	0.0035	2.89E-06	21.9559
M03141.m	rs185077	T	C	0.4986	-0.0193	0.0023	1.54E-16	70.414
M03141.m	rs2291919	A	C	0.3011	-0.0148	0.0025	2.58E-09	35.0464
M03141.m	rs2396726	T	C	0.5395	-0.0179	0.0039	4.52E-06	21.0657
M03141.m	rs3797248	A	G	0.1651	0.012	0.0026	4.62E-06	21.3018
M03141.m	rs4327704	A	C	0.9547	-0.0219	0.005	9.94E-06	19.1844
M03141.m	rs4646359	T	C	0.5297	-0.0083	0.0018	3.2E-06	21.2623
M03141.m	rs495360	A	C	0.497	-0.019	0.003	2.4E-10	40.1111
M03141.m	rs6860725	T	C	0.3227	-0.0126	0.0024	1.79E-07	27.5625
M03141.m	rs6908726	C	G	0.9098	0.0155	0.0035	7.17E-06	19.6122
M03141.m	rs7126003	T	C	0.6259	0.0185	0.0039	2.58E-06	22.5016
M03141.m	rs715	T	C	0.713	0.0221	0.0025	2.08E-19	78.1456
M03141.m	rs7668550	T	G	0.0157	-0.0368	0.0081	5.54E-06	20.6408
M03141.m	rs7781104	T	C	0.2683	0.0208	0.0047	7.76E-06	19.5853
M03141.m	rs7896866	A	C	0.2982	0.0108	0.0024	8.34E-06	20.25
M03141.m	rs9462856	T	C	0.4711	-0.0093	0.0018	2E-07	26.6944
M03141.m	rs9886534	T	C	0.5101	-0.0079	0.0018	8.74E-06	19.2623
M03141.m	rs9981490	A	G	0.5207	-0.0079	0.0018	8.29E-06	19.2623
M03147.m	rs11250123	A	G	0.043	-0.0494	0.0097	3.83E-07	25.9364
M03147.m	rs4673167	A	G	0.889	-0.021	0.0044	1.7E-06	22.7789
M03147.m	rs4694292	T	C	0.1089	0.0201	0.0045	6.44E-06	19.9511
M10642.m	rs10514491	T	C	0.6781	0.0094	0.0017	4.46E-08	30.5744
M10642.m	rs11196097	A	G	0.1128	0.0111	0.0025	9.83E-06	19.7136
M10642.m	rs11664190	T	C	0.592	-0.0082	0.0016	6.47E-07	26.2656
M10642.m	rs12026398	T	C	0.8893	-0.014	0.0029	9.43E-07	23.3056

M10642.m	rs12136055	T	C	0.0129	-0.0731	0.0153	1.75E-06	22.8272
M10642.m	rs12586821	T	C	0.1222	0.0129	0.0025	1.62E-07	26.6256
M10642.m	rs13380271	T	C	0.4149	-0.0077	0.0016	2.9E-06	23.1602
M10642.m	rs1391933	T	G	0.4399	-0.0075	0.0016	5.79E-06	21.9727
M10642.m	rs1530919	A	G	0.3006	0.0076	0.0017	5.92E-06	19.9862
M10642.m	rs1549556	T	C	0.3719	-0.0075	0.0017	7.95E-06	19.4637
M10642.m	rs1576171	A	T	0.984	-0.043	0.009	1.77E-06	22.8272
M10642.m	rs17169194	A	C	0.8806	-0.0117	0.0026	6.65E-06	20.25
M10642.m	rs17616891	A	T	0.6964	-0.0076	0.0017	4.65E-06	19.9862
M10642.m	rs17695897	T	C	0.153	0.011	0.0025	8.03E-06	19.36
M10642.m	rs2720692	A	G	0.0648	-0.0272	0.006	5.63E-06	20.5511
M10642.m	rs2725779	T	C	0.4523	0.0077	0.0017	3.27E-06	20.5156
M10642.m	rs2938330	T	C	0.8105	-0.0117	0.0025	2.15E-06	21.9024
M10642.m	rs3781273	A	G	0.5139	0.0074	0.0017	8.52E-06	18.9481
M10642.m	rs4436895	C	G	0.252	-0.01	0.0022	3.47E-06	20.6612
M10642.m	rs4483514	T	G	0.357	0.009	0.002	6.8E-06	20.25
M10642.m	rs4812805	C	G	0.8421	-0.0109	0.0024	5.88E-06	20.6267
M10642.m	rs4818244	T	C	0.0939	0.0136	0.003	6.61E-06	20.5511
M10642.m	rs569114	A	G	0.6199	-0.0079	0.0017	1.99E-06	21.5952
M10642.m	rs6540619	T	C	0.434	0.0076	0.0016	3.71E-06	22.5625
M10642.m	rs6557106	T	C	0.6715	0.0076	0.0017	5.42E-06	19.9862
M10642.m	rs6828152	T	G	0.359	0.009	0.002	6.8E-06	20.25
M10642.m	rs7735310	T	G	0.7188	-0.0078	0.0017	3.38E-06	21.0519
M10642.m	rs7859597	A	G	0.737	-0.009	0.002	6.8E-06	20.25
M10642.m	rs8043217	A	G	0.524	0.009	0.0016	2.79E-08	31.6406
M10642.m	rs8128639	A	C	0.1698	-0.0113	0.0025	6.51E-06	20.4304
M10642.m	rs947099	A	G	0.3625	-0.008	0.0017	1.22E-06	22.1453
M10642.m	rs9529827	T	C	0.9843	-0.0397	0.0087	5.14E-06	20.823
M11438.m	rs10404980	A	G	0.9877	-0.0744	0.0158	2.34E-06	22.1734
M11438.m	rs1466522	T	C	0.7645	-0.0067	0.0013	3.15E-07	26.5621
M11438.m	rs4641759	T	C	0.7521	-0.0135	0.003	6.66E-06	20.25
M11438.m	rs6126354	T	C	0.7906	-0.0134	0.0029	5.46E-06	21.3508
M12017.m	rs10012175	T	C	0.1065	0.0194	0.0043	5.78E-06	20.3548
M12017.m	rs10800623	A	G	0.2767	-0.0262	0.0058	6.17E-06	20.4055
M12017.m	rs10963172	A	G	0.9724	0.0352	0.0078	6.99E-06	20.3655
M12017.m	rs11192702	A	G	0.0456	-0.0275	0.0062	7.88E-06	19.6735
M12017.m	rs11564710	A	C	0.3886	0.0166	0.0034	9.79E-07	23.8374
M12017.m	rs12131781	A	G	0.7091	0.0126	0.0027	2.59E-06	21.7778
M12017.m	rs12538514	C	G	0.3714	0.0124	0.0026	2.43E-06	22.7456
M12017.m	rs12815706	A	G	0.2806	0.019	0.0039	9.1E-07	23.7344
M12017.m	rs339707	T	G	0.6768	-0.0142	0.0026	6.35E-08	29.8284
M12017.m	rs6442530	T	C	0.3254	-0.026	0.0055	2.75E-06	22.3471
M12017.m	rs6592988	T	C	0.1439	0.0167	0.0035	2.4E-06	22.7665
M12017.m	rs7253109	T	G	0.3395	0.0125	0.0026	2.34E-06	23.1139
M12017.m	rs7514881	C	G	0.2524	0.012	0.0027	8.53E-06	19.7531
M12017.m	rs7751499	A	G	0.684	0.0124	0.0026	2.57E-06	22.7456
M12017.m	rs7908166	A	G	0.9724	-0.0356	0.0079	7.68E-06	20.307
M12017.m	rs9323203	A	C	0.7331	-0.0118	0.0027	9.5E-06	19.1001
M12035.m	rs10435813	A	G	0.8033	0.0114	0.0026	8.31E-06	19.2249
M12035.m	rs10514491	T	C	0.6801	0.0122	0.0024	6.73E-07	25.8403
M12035.m	rs10738508	A	G	0.3472	-0.008	0.0018	6.61E-06	19.7531
M12035.m	rs10848204	T	C	0.5451	0.0093	0.0018	1.26E-07	26.6944
M12035.m	rs10967809	C	G	0.5893	0.0082	0.0017	2.64E-06	23.2664
M12035.m	rs1097296	T	C	0.3933	0.0078	0.0018	9.22E-06	18.7778
M12035.m	rs11734565	T	C	0.0402	0.0248	0.0056	9.29E-06	19.6122

M12035.m	rs11895467	T	G	0.3807	0.0094	0.0018	8.43E-08	27.2716
M12035.m	rs11937702	A	G	0.1122	0.0157	0.0033	2.39E-06	22.6345
M12035.m	rs1229590	A	G	0.505	-0.0084	0.0018	5.23E-06	21.7778
M12035.m	rs1440492	A	G	0.7102	0.0179	0.004	8.02E-06	20.0256
M12035.m	rs1447842	A	G	0.5745	0.0082	0.0017	2.85E-06	23.2664
M12035.m	rs16886123	A	G	0.8616	-0.0119	0.0026	3.71E-06	20.9482
M12035.m	rs16944339	A	T	0.1545	-0.0228	0.0049	3.75E-06	21.651
M12035.m	rs1846975	A	G	0.3554	0.0083	0.0018	2.56E-06	21.2623
M12035.m	rs1866411	A	C	0.3468	-0.0085	0.0018	1.41E-06	22.2994
M12035.m	rs1938479	T	C	0.6445	-0.01	0.0018	1.78E-08	30.8642
M12035.m	rs2049534	T	C	0.5044	-0.0082	0.0018	3.18E-06	20.7531
M12035.m	rs2147618	A	C	0.5036	-0.0081	0.0018	5.31E-06	20.25
M12035.m	rs2286396	T	C	0.1505	0.0117	0.0026	5.6E-06	20.25
M12035.m	rs2863202	T	C	0.178	0.0114	0.0025	6.93E-06	20.7936
M12035.m	rs4646880	A	G	0.5434	-0.0085	0.0018	1.5E-06	22.2994
M12035.m	rs4834846	A	G	0.5501	0.0079	0.0018	6.52E-06	19.2623
M12035.m	rs4848532	T	C	0.4842	-0.0079	0.0018	9.76E-06	19.2623
M12035.m	rs511545	T	C	0.583	0.0081	0.0018	4.63E-06	20.25
M12035.m	rs630782	T	C	0.4728	0.0077	0.0017	9.38E-06	20.5156
M12035.m	rs6864461	C	G	0.3644	0.0088	0.0018	6.76E-07	23.9012
M12035.m	rs7002819	T	C	0.5269	0.008	0.0018	6.15E-06	19.7531
M12035.m	rs7136574	T	C	0.4345	0.0093	0.0018	1.33E-07	26.6944
M12035.m	rs7214330	T	C	0.1256	0.0144	0.0032	9.76E-06	20.25
M12035.m	rs7642054	T	C	0.5887	-0.0171	0.0037	4.64E-06	21.3594
M12035.m	rs7809262	A	G	0.425	-0.0088	0.0017	4.41E-07	26.7958
M12035.m	rs8015724	T	C	0.3913	-0.0078	0.0018	9.16E-06	18.7778
M12035.m	rs8079173	A	G	0.1414	0.0119	0.0026	3.42E-06	20.9482
M12035.m	rs899227	A	G	0.3958	-0.0084	0.0018	2.97E-06	21.7778
M12035.m	rs9033	A	G	0.5521	0.0082	0.0018	4.24E-06	20.7531
M12035.m	rs9513489	T	C	0.5888	0.0078	0.0018	8.48E-06	18.7778
M12067.m	rs10435813	A	G	0.8067	0.0081	0.0018	4.01E-06	20.25
M12067.m	rs10520198	A	G	0.0296	0.0186	0.0041	5.64E-06	20.5806
M12067.m	rs10839561	C	G	0.9453	-0.0177	0.0039	6.46E-06	20.5976
M12067.m	rs1122530	A	G	0.8519	-0.0094	0.0018	1.49E-07	27.2716
M12067.m	rs11912450	A	T	0.1939	-0.0078	0.0018	8.58E-06	18.7778
M12067.m	rs12116440	T	C	0.0144	-0.0679	0.0138	9.28E-07	24.2093
M12067.m	rs1216886	A	G	0.858	-0.008	0.0018	6.88E-06	19.7531
M12067.m	rs12826254	T	C	0.1364	0.0091	0.0018	7.05E-07	25.5586
M12067.m	rs1408276	A	C	0.955	-0.043	0.0094	4.59E-06	20.9258
M12067.m	rs16883391	A	C	0.1921	-0.0084	0.0017	1.2E-06	24.4152
M12067.m	rs17031632	A	G	0.9284	0.0133	0.0027	5.69E-07	24.2647
M12067.m	rs170631	C	G	0.8913	-0.0208	0.0043	1.78E-06	23.3986
M12067.m	rs17094565	A	T	0.9357	-0.0125	0.0026	1.88E-06	23.1139
M12067.m	rs17103129	T	C	0.9595	-0.0154	0.0035	8.98E-06	19.36
M12067.m	rs17145331	T	C	0.8525	-0.0091	0.0018	3.58E-07	25.5586
M12067.m	rs17233321	A	G	0.9519	-0.0175	0.0034	3.74E-07	26.4922
M12067.m	rs1864609	A	C	0.8606	-0.0091	0.0018	3.27E-07	25.5586
M12067.m	rs196885	T	C	0.8501	-0.0086	0.0018	1.22E-06	22.8272
M12067.m	rs2471075	A	G	0.9318	-0.0176	0.0036	8.39E-07	23.9012
M12067.m	rs2944772	C	G	0.8193	-0.0079	0.0018	7.04E-06	19.2623
M12067.m	rs4519482	A	T	0.291	-0.0091	0.0017	8.79E-08	28.654
M12067.m	rs6140831	A	C	0.5484	0.0089	0.0016	5.4E-08	30.9414
M12067.m	rs686321	T	C	0.8232	0.0082	0.0017	2.14E-06	23.2664
M12067.m	rs6967282	A	G	0.797	0.0088	0.0017	3.96E-07	26.7958
M12067.m	rs7069652	A	C	0.1869	-0.0088	0.0017	4.03E-07	26.7958

M12067.m	rs7134822	A	G	0.9597	-0.0164	0.0035	3.87E-06	21.9559
M12067.m	rs7279224	A	T	0.6952	0.0077	0.0017	6.87E-06	20.5156
M12067.m	rs7666900	T	C	0.1418	0.0089	0.0018	6.93E-07	24.4475
M12129.m	rs10494270	A	G	0.7114	0.0119	0.0027	8.63E-06	19.4252
M12129.m	rs10945649	A	T	0.1287	0.0212	0.0036	4.29E-09	34.679
M12129.m	rs11605057	T	C	0.8787	-0.0202	0.0043	2.24E-06	22.0681
M12129.m	rs1185260	A	G	0.1225	-0.0191	0.0036	1.14E-07	28.1489
M12129.m	rs11858062	T	C	0.9142	0.0213	0.0044	1.65E-06	23.4344
M12129.m	rs11869714	A	G	0.7762	-0.0126	0.0027	4.25E-06	21.7778
M12129.m	rs12325299	C	G	0.6932	0.0122	0.0027	6.94E-06	20.417
M12129.m	rs12950972	T	C	0.6497	-0.0133	0.0027	9.84E-07	24.2647
M12129.m	rs1337278	A	G	0.6107	0.0124	0.0027	3.68E-06	21.0919
M12129.m	rs1448817	A	G	0.7393	0.0124	0.0027	4.56E-06	21.0919
M12129.m	rs17386586	A	C	0.2495	-0.0134	0.0027	9.74E-07	24.631
M12129.m	rs2130791	A	G	0.7132	0.0121	0.0027	7.96E-06	20.0837
M12129.m	rs3209233	A	C	0.8814	0.041	0.0093	9.95E-06	19.4358
M12129.m	rs3778889	T	C	0.0616	0.0246	0.0053	3.42E-06	21.5436
M12129.m	rs6477873	T	C	0.6394	0.0131	0.0027	9.51E-07	23.5405
M12129.m	rs6952850	A	G	0.6998	-0.012	0.0027	6.88E-06	19.7531
M12129.m	rs7178596	A	G	0.3535	-0.012	0.0027	7.92E-06	19.7531
M12129.m	rs7446718	A	G	0.8591	-0.0417	0.0082	3.83E-07	25.8609
M12129.m	rs7544738	A	G	0.4114	0.0132	0.0027	7.15E-07	23.9012
M12129.m	rs7660017	A	G	0.3331	-0.0131	0.0028	2.34E-06	21.889
M12129.m	rs789195	A	G	0.7657	-0.0128	0.0027	2.53E-06	22.4746
M12129.m	rs943251	T	G	0.3712	0.0118	0.0027	9.9E-06	19.1001
M12129.m	rs9460599	T	C	0.2443	0.0302	0.0066	4.86E-06	20.9376
M12129.m	rs9811126	A	G	0.0922	0.0216	0.0044	6.87E-07	24.0992
M12129.m	rs9841498	T	C	0.2663	0.0145	0.0027	7.12E-08	28.8409
M12261.m	rs10975331	T	C	0.0361	0.1208	0.0273	9.6E-06	19.5798
M12261.m	rs11241933	A	T	0.2705	-0.061	0.0125	1.05E-06	23.8144
M12261.m	rs1153699	T	G	0.2385	0.0602	0.013	3.89E-06	21.444
M12261.m	rs11949031	A	G	0.1629	0.0662	0.0138	1.52E-06	23.0122
M12261.m	rs17022499	A	T	0.2267	0.054	0.0122	9.71E-06	19.5915
M12261.m	rs17403795	A	G	0.1166	0.073	0.0161	5.61E-06	20.5586
M12261.m	rs1898906	A	G	0.0296	0.1378	0.0299	4.09E-06	21.2401
M12261.m	rs2032297	C	G	0.7857	0.057	0.0123	3.85E-06	21.4753
M12261.m	rs4632709	T	C	0.95	-0.1504	0.0297	4E-07	25.6438
M12261.m	rs4896370	A	G	0.3542	0.0555	0.0121	4.19E-06	21.0385
M12261.m	rs7184864	A	G	0.8047	-0.0583	0.0131	9.14E-06	19.8059
M12261.m	rs7961079	A	G	0.6241	0.0527	0.0109	1.46E-06	23.3759
M12261.m	rs8137495	A	G	0.7393	0.0602	0.0135	7.8E-06	19.885
M12593.m	rs10132580	T	G	0.1734	0.0094	0.0017	5.67E-08	30.5744
M12593.m	rs10279326	T	C	0.9522	-0.0148	0.0033	6.52E-06	20.1139
M12593.m	rs10483826	A	G	0.7932	-0.0078	0.0017	4.6E-06	21.0519
M12593.m	rs10937658	A	G	0.2676	0.0077	0.0017	3.5E-06	20.5156
M12593.m	rs11715915	T	C	0.2789	-0.0076	0.0016	3.77E-06	22.5625
M12593.m	rs11874996	T	C	0.0175	-0.0523	0.0115	5.62E-06	20.6827
M12593.m	rs12042948	A	G	0.8045	-0.0084	0.0017	1.4E-06	24.4152
M12593.m	rs12444778	T	C	0.0607	-0.0156	0.0032	1.61E-06	23.7656
M12593.m	rs12896482	T	C	0.8919	-0.0109	0.0024	4.17E-06	20.6267
M12593.m	rs1379459	T	C	0.8083	0.0079	0.0017	3.14E-06	21.5952
M12593.m	rs1556988	A	G	0.7923	-0.0077	0.0017	5.86E-06	20.5156
M12593.m	rs1615157	T	C	0.5764	0.0076	0.0016	2.22E-06	22.5625
M12593.m	rs17562832	T	C	0.0769	0.0118	0.0026	4.17E-06	20.5976
M12593.m	rs2422370	A	G	0.8094	-0.0084	0.0018	5.96E-06	21.7778

M12593.m	rs2705962	A	G	0.8068	0.0078	0.0017	4.63E-06	21.0519
M12593.m	rs6129758	T	G	0.843	0.0078	0.0017	7.88E-06	21.0519
M12593.m	rs6543699	A	G	0.5118	0.0116	0.0026	8.92E-06	19.9053
M12593.m	rs6924754	A	G	0.0606	0.0154	0.0034	6.43E-06	20.5156
M12593.m	rs699191	T	C	0.1916	0.0076	0.0017	9.91E-06	19.9862
M12593.m	rs7068714	A	C	0.1774	0.008	0.0017	2.97E-06	22.1453
M12593.m	rs7914384	T	C	0.1806	-0.0082	0.0017	1.85E-06	23.2664
M12593.m	rs8046494	A	G	0.9288	0.0138	0.003	4.08E-06	21.16
M12593.m	rs905508	T	C	0.1655	0.0079	0.0017	5.94E-06	21.5952
M12593.m	rs9396681	T	C	0.9072	-0.012	0.0024	6.25E-07	25
M12593.m	rs9861011	A	G	0.6558	-0.0074	0.0016	3.8E-06	21.3906
M12626.m	rs10426856	A	G	0.8949	-0.0157	0.0035	8.06E-06	20.1216
M12626.m	rs16940342	A	G	0.88	0.0158	0.0035	5.36E-06	20.3788
M12626.m	rs17150563	T	C	0.7256	0.0117	0.0026	4.61E-06	20.25
M12626.m	rs4978089	A	G	0.2125	0.0117	0.0026	8.78E-06	20.25
M12626.m	rs684748	C	G	0.9142	0.018	0.0041	8.62E-06	19.2742
M12626.m	rs6904862	T	C	0.7427	0.0115	0.0026	9.43E-06	19.5636
M12626.m	rs729761	T	G	0.2479	-0.0139	0.0031	8.52E-06	20.1051
M12626.m	rs7844733	T	C	0.1881	-0.0126	0.0027	2.68E-06	21.7778
M12626.m	rs8105499	A	C	0.2909	0.0123	0.0026	1.57E-06	22.3802
M12768.m	rs10416177	T	G	0.592	0.0119	0.0026	6.93E-06	20.9482
M12768.m	rs10512645	A	G	0.6525	-0.0121	0.0027	5.52E-06	20.0837
M12768.m	rs10787426	T	C	0.4783	-0.0123	0.0026	2.98E-06	22.3802
M12768.m	rs10945327	A	G	0.6184	0.0124	0.0026	2.32E-06	22.7456
M12768.m	rs11200954	T	G	0.9527	-0.0278	0.0062	8.12E-06	20.1051
M12768.m	rs11734331	A	G	0.3779	-0.0119	0.0027	7.81E-06	19.4252
M12768.m	rs12230393	A	C	0.9032	-0.02	0.0045	7.42E-06	19.7531
M12768.m	rs12671954	A	G	0.5605	-0.0118	0.0026	7.27E-06	20.5976
M12768.m	rs1671347	A	G	0.0462	0.0311	0.0069	7.26E-06	20.3153
M12768.m	rs16985223	T	C	0.9655	0.1077	0.0225	1.69E-06	22.9122
M12768.m	rs1748947	T	G	0.9114	-0.0264	0.0055	1.37E-06	23.04
M12768.m	rs2487411	T	C	0.3154	-0.0122	0.0027	4.68E-06	20.417
M12768.m	rs300250	A	G	0.639	0.0118	0.0026	5.82E-06	20.5976
M12768.m	rs3114834	T	C	0.1017	0.0213	0.0048	9.86E-06	19.6914
M12768.m	rs4239564	A	G	0.1652	-0.0155	0.0035	7.62E-06	19.6122
M12768.m	rs6464030	T	C	0.591	-0.0123	0.0026	2.99E-06	22.3802
M12768.m	rs7127507	T	C	0.6814	0.0122	0.0027	4.95E-06	20.417
M12768.m	rs7145793	T	G	0.0337	-0.1012	0.0224	6.27E-06	20.411
M12768.m	rs7700710	A	G	0.3246	0.012	0.0027	6.73E-06	19.7531
M12768.m	rs7766963	T	C	0.5211	0.0116	0.0026	8.56E-06	19.9053
M12774.m	rs10088340	A	G	0.0749	0.0238	0.0053	6.75E-06	20.1652
M12774.m	rs10096922	A	T	0.8346	0.0121	0.0027	5.42E-06	20.0837
M12774.m	rs10186957	T	C	0.837	-0.0124	0.0026	2.42E-06	22.7456
M12774.m	rs10738342	T	C	0.5346	0.0082	0.0018	3.7E-06	20.7531
M12774.m	rs10822248	T	C	0.8488	-0.0136	0.0026	2.84E-07	27.3609
M12774.m	rs10899249	T	C	0.5316	-0.0081	0.0018	7.02E-06	20.25
M12774.m	rs11947970	T	C	0.1515	-0.0119	0.0026	5.03E-06	20.9482
M12774.m	rs12416136	A	C	0.4756	-0.0082	0.0018	3.44E-06	20.7531
M12774.m	rs1260326	T	C	0.4088	0.0133	0.0024	1.83E-08	30.7101
M12774.m	rs1289240	T	C	0.5712	-0.0084	0.0018	2.41E-06	21.7778
M12774.m	rs1929672	T	C	0.5201	0.0087	0.0018	1.15E-06	23.3611
M12774.m	rs1930038	T	C	0.5477	0.008	0.0018	7.29E-06	19.7531
M12774.m	rs2187375	A	G	0.1651	-0.0221	0.0026	2.65E-17	72.25
M12774.m	rs2285943	T	G	0.4941	0.0088	0.0018	7.6E-07	23.9012
M12774.m	rs2451284	T	C	0.4026	-0.0084	0.0018	2.75E-06	21.7778

M12774.m	rs2862982	A	G	0.8794	-0.0149	0.0033	5.01E-06	20.3866
M12774.m	rs2980872	A	G	0.461	-0.009	0.002	6.8E-06	20.25
M12774.m	rs3219095	T	C	0.8471	0.0117	0.0026	9.79E-06	20.25
M12774.m	rs3786248	T	C	0.954	-0.0237	0.0051	3.42E-06	21.5952
M12774.m	rs400839	T	C	0.4443	-0.008	0.0018	7.8E-06	19.7531
M12774.m	rs4236893	T	C	0.1865	-0.0116	0.0025	4.78E-06	21.5296
M12774.m	rs623068	A	C	0.9375	0.0352	0.0079	8.23E-06	19.8532
M12774.m	rs6679002	T	G	0.5914	0.0141	0.0018	2.16E-15	61.3611
M12774.m	rs716140	T	C	0.4892	0.0079	0.0018	8.3E-06	19.2623
M12774.m	rs7520876	A	G	0.9879	0.044	0.0094	3.15E-06	21.9104
M12774.m	rs782378	A	G	0.5252	-0.0089	0.0018	6.01E-07	24.4475
M12774.m	rs7941822	A	G	0.494	0.0079	0.0018	8.03E-06	19.2623
M12774.m	rs8025505	T	C	0.2344	0.0116	0.0026	7.76E-06	19.9053
M12774.m	rs8078118	A	C	0.4238	0.0085	0.0019	6.98E-06	20.0139
M12774.m	rs964184	C	G	0.8617	-0.0252	0.0032	7.95E-15	62.0156
M12774.m	rs9855331	A	T	0.7473	0.0147	0.0027	3.81E-08	29.642
M12774.m	rs988143	T	C	0.4828	0.0081	0.0018	5.25E-06	20.25
M12774.m	rs998933	T	C	0.9528	0.0252	0.0051	7.95E-07	24.4152
M15122.m	rs10036528	A	G	0.0757	-0.0209	0.0043	9.58E-07	23.6241
M15122.m	rs10216545	A	G	0.4694	-0.0214	0.0047	5.19E-06	20.7316
M15122.m	rs10839972	T	G	0.1872	0.0121	0.0027	7.91E-06	20.0837
M15122.m	rs12337470	T	C	0.8324	-0.0123	0.0027	5.9E-06	20.7531
M15122.m	rs13106354	A	T	0.2123	-0.0127	0.0027	2.31E-06	22.1248
M15122.m	rs13216754	A	G	0.0117	0.0555	0.0105	1.31E-07	27.9388
M15122.m	rs1614303	T	G	0.8159	0.0138	0.0027	2.4E-07	26.1235
M15122.m	rs2076799	C	G	0.0853	0.0166	0.0036	3.89E-06	21.2623
M15122.m	rs2259038	A	G	0.203	-0.0216	0.0048	7.51E-06	20.25
M15122.m	rs2501242	T	G	0.223	-0.0136	0.0026	1.84E-07	27.3609
M15122.m	rs2941840	A	G	0.0691	0.0221	0.0048	3.71E-06	21.1984
M15122.m	rs4436910	A	T	0.8036	0.0118	0.0027	9.13E-06	19.1001
M15122.m	rs5022234	A	C	0.6303	0.0221	0.0047	2.15E-06	22.11
M15122.m	rs5759809	T	C	0.011	0.069	0.015	4.23E-06	21.16
M15122.m	rs641939	A	G	0.7987	0.0127	0.0027	2.13E-06	22.1248
M15122.m	rs806214	A	G	0.2175	0.0123	0.0026	2.94E-06	22.3802
M15122.m	rs9374302	A	G	0.1744	-0.0141	0.0027	1.72E-07	27.2716
M15122.m	rs9878149	A	G	0.2674	0.0117	0.0026	5.48E-06	20.25
M15140.m	rs10085935	T	C	0.3782	-0.0103	0.0017	3.33E-09	36.7093
M15140.m	rs10857319	T	C	0.3481	-0.0086	0.0017	9.45E-07	25.5917
M15140.m	rs10937762	A	G	0.5679	0.0078	0.0018	8.16E-06	18.7778
M15140.m	rs11057695	T	C	0.0566	0.0206	0.0046	8.03E-06	20.0548
M15140.m	rs11162752	A	G	0.5972	-0.0077	0.0017	8.08E-06	20.5156
M15140.m	rs11593042	A	T	0.0919	0.0151	0.0033	4.55E-06	20.9376
M15140.m	rs11646849	A	G	0.5005	-0.0086	0.0017	6.4E-07	25.5917
M15140.m	rs12082398	T	C	0.5855	0.0117	0.0024	9.33E-07	23.7656
M15140.m	rs12490394	T	G	0.4967	0.0084	0.0019	8.01E-06	19.5457
M15140.m	rs12802337	A	G	0.0776	-0.0161	0.0036	5.95E-06	20.0008
M15140.m	rs12937634	T	C	0.5397	-0.0082	0.0017	2.28E-06	23.2664
M15140.m	rs1426134	C	G	0.3715	-0.0081	0.0017	3.84E-06	22.7024
M15140.m	rs1496635	A	C	0.3407	-0.0091	0.0018	2.12E-07	25.5586
M15140.m	rs16924894	A	T	0.024	0.0814	0.0146	2.33E-08	31.0844
M15140.m	rs16974854	T	C	0.038	-0.0234	0.0048	1.15E-06	23.7656
M15140.m	rs17630235	A	G	0.4183	0.0131	0.0017	3.41E-14	59.3806
M15140.m	rs180050	T	G	0.5821	0.008	0.0018	7.65E-06	19.7531
M15140.m	rs21327	A	G	0.286	0.0095	0.0018	1.25E-07	27.8549
M15140.m	rs2320536	T	C	0.4131	0.0081	0.0017	3.32E-06	22.7024

M15140.m	rs2375475	T	C	0.3236	-0.0084	0.0017	1.41E-06	24.4152
M15140.m	rs2491294	A	G	0.31	-0.01	0.002	5.73E-07	25
M15140.m	rs2651516	T	C	0.6585	0.008	0.0017	4.3E-06	22.1453
M15140.m	rs3184504	T	C	0.4851	0.0149	0.0017	6.05E-18	76.8201
M15140.m	rs3788706	A	G	0.1302	-0.0117	0.0026	6.1E-06	20.25
M15140.m	rs3789978	A	G	0.8469	0.0123	0.0025	1.32E-06	24.2064
M15140.m	rs3809198	A	C	0.0199	0.0554	0.012	4.28E-06	21.3136
M15140.m	rs4820242	A	G	0.6201	-0.0085	0.0017	9.03E-07	25
M15140.m	rs511797	A	C	0.1261	0.012	0.0026	3.77E-06	21.3018
M15140.m	rs6575634	T	C	0.6184	-0.0158	0.0035	4.85E-06	20.3788
M15140.m	rs669764	T	C	0.3176	-0.0078	0.0018	7.73E-06	18.7778
M15140.m	rs6770323	T	C	0.4299	-0.008	0.0017	3.33E-06	22.1453
M15140.m	rs6815057	A	G	0.5786	0.0085	0.0017	1.12E-06	25
M15140.m	rs6946693	T	G	0.7069	0.0102	0.0023	9.18E-06	19.6673
M15140.m	rs739744	T	C	0.5529	-0.0082	0.0017	1.88E-06	23.2664
M15140.m	rs750950	A	C	0.6442	0.0144	0.0018	2.34E-16	64
M15140.m	rs7548008	A	G	0.504	0.016	0.0035	3.56E-06	20.898
M15140.m	rs759507	T	C	0.366	0.008	0.0018	5.18E-06	19.7531
M15140.m	rs7619096	A	C	0.6886	0.008	0.0017	4.92E-06	22.1453
M15140.m	rs7619568	A	G	0.94	0.0177	0.004	9.53E-06	19.5806
M15140.m	rs7843776	T	C	0.4038	-0.0084	0.0019	7.41E-06	19.5457
M15140.m	rs7906282	T	G	0.3095	0.0094	0.0018	8.1E-08	27.2716
M15140.m	rs8051149	A	G	0.2127	0.0258	0.0025	9.07E-26	106.502
M15140.m	rs886125	A	G	0.463	0.0082	0.0017	1.86E-06	23.2664
M15140.m	rs9533582	A	G	0.0351	-0.0222	0.005	1E-05	19.7136
M15140.m	rs9857268	A	G	0.411	0.008	0.0017	3.07E-06	22.1453
M15335.m	rs10017961	A	G	0.2731	0.0486	0.0107	5.62E-06	20.6303
M15335.m	rs10811658	A	G	0.2599	-0.0611	0.0135	6.22E-06	20.484
M15335.m	rs11487092	A	G	0.012	0.248	0.053	2.88E-06	21.8953
M15335.m	rs16967757	T	C	0.3037	0.0495	0.0103	1.43E-06	23.096
M15335.m	rs1998053	A	G	0.0855	0.0724	0.0159	5.6E-06	20.734
M15335.m	rs2365522	A	C	0.7137	0.0442	0.0098	6.7E-06	20.3419
M15335.m	rs4404993	T	G	0.1848	0.0964	0.0207	3.22E-06	21.6877
M15335.m	rs488225	A	G	0.3902	-0.0411	0.009	4.87E-06	20.8544
M15335.m	rs4917405	T	C	0.0437	0.0972	0.0215	6.28E-06	20.4388
M15335.m	rs6434811	T	C	0.9256	-0.0785	0.0169	3.52E-06	21.5758
M15335.m	rs6657899	T	G	0.0322	0.1908	0.0427	7.96E-06	19.9665
M15335.m	rs7184992	T	C	0.3003	0.0807	0.0167	1.31E-06	23.3515
M15335.m	rs7240261	T	C	0.562	0.0565	0.0126	7.52E-06	20.1074
M15365.m	rs11020587	A	G	0.9798	-0.0362	0.008	7.02E-06	20.4756
M15365.m	rs11062138	A	G	0.1724	0.0131	0.0027	1.46E-06	23.5405
M15365.m	rs1159317	C	G	0.2439	-0.0149	0.0034	9.27E-06	19.205
M15365.m	rs13120638	T	G	0.9213	0.0443	0.0096	3.96E-06	21.2944
M15365.m	rs1813982	T	C	0.2209	-0.0122	0.0027	6.59E-06	20.417
M15365.m	rs2517897	A	C	0.1141	0.0171	0.0035	1.23E-06	23.8702
M15365.m	rs333383	A	G	0.8384	-0.0127	0.0027	3.38E-06	22.1248
M15365.m	rs4075155	T	C	0.7961	-0.012	0.0027	8.88E-06	19.7531
M15365.m	rs4816048	C	G	0.1794	0.0123	0.0027	7.13E-06	20.7531
M15365.m	rs6130362	C	G	0.2071	0.0124	0.0027	5.62E-06	21.0919
M15365.m	rs7813124	A	T	0.9866	-0.0433	0.0098	9.41E-06	19.522
M15365.m	rs7867387	A	G	0.9051	0.0167	0.0036	4.16E-06	21.5193
M15365.m	rs874758	A	G	0.1855	-0.0134	0.0028	1.43E-06	22.9031
M15488.m	rs10490240	A	G	0.8318	0.0078	0.0017	5.17E-06	21.0519
M15488.m	rs10933558	A	G	0.794	-0.0074	0.0017	9.25E-06	18.9481
M15488.m	rs11539202	A	G	0.6685	-0.007	0.0016	9.77E-06	19.1406

M15488.m	rs11843722	T	C	0.9644	0.019	0.0039	9.47E-07	23.7344
M15488.m	rs12119234	T	C	0.2374	0.0095	0.0018	7.11E-08	27.8549
M15488.m	rs12482635	T	C	0.2693	0.0075	0.0016	5.4E-06	21.9727
M15488.m	rs1493749	A	G	0.17	0.0086	0.0019	3.73E-06	20.4875
M15488.m	rs2044899	A	C	0.3787	0.0074	0.0016	3.54E-06	21.3906
M15488.m	rs2799635	T	C	0.3644	0.0089	0.0016	1.96E-08	30.9414
M15488.m	rs302121	A	T	0.819	0.0094	0.0021	8.61E-06	20.0363
M15488.m	rs3904667	A	T	0.2059	-0.0078	0.0017	3.2E-06	21.0519
M15488.m	rs4970834	T	C	0.1821	-0.0098	0.0017	1.06E-08	33.2318
M15488.m	rs6455128	A	C	0.1757	0.0081	0.0017	2.21E-06	22.7024
M15488.m	rs7288834	T	G	0.3986	-0.0083	0.0016	1.51E-07	26.9102
M15488.m	rs9572809	A	C	0.97	-0.0223	0.005	1E-05	19.8916
M15488.m	rs9811522	A	G	0.174	0.01	0.002	5.73E-07	25
M15488.m	rs9818690	T	C	0.6477	0.0071	0.0016	7.07E-06	19.6914
M15488.m	rs9998852	A	G	0.6611	0.0076	0.0016	2.77E-06	22.5625
M15500.m	rs10111368	A	G	0.5456	-0.0041	0.0009	4.98E-06	20.7531
M15500.m	rs10130689	T	C	0.1587	-0.0075	0.0017	4.99E-06	19.4637
M15500.m	rs10136635	A	G	0.5453	0.0041	0.0009	0.000006	20.7531
M15500.m	rs10239136	T	C	0.5959	0.0044	0.0009	1.49E-06	23.9012
M15500.m	rs10275028	T	C	0.6522	0.0046	0.0009	4.9E-07	26.1235
M15500.m	rs1028981	A	G	0.385	-0.005	0.001	5.73E-07	25
M15500.m	rs10481187	T	G	0.3707	0.0045	0.0009	9.69E-07	25
M15500.m	rs10489008	A	C	0.4897	0.0045	0.0009	4.62E-07	25
M15500.m	rs10784558	T	C	0.4845	-0.0048	0.0009	1.06E-07	28.4444
M15500.m	rs10789970	T	C	0.4061	-0.0041	0.0009	6.63E-06	20.7531
M15500.m	rs10802528	T	G	0.4544	-0.0047	0.0009	1.95E-07	27.2716
M15500.m	rs10818924	T	C	0.6254	-0.0045	0.0009	6.08E-07	25
M15500.m	rs10820588	T	G	0.5616	0.0048	0.0009	1.64E-07	28.4444
M15500.m	rs10869018	T	C	0.5113	-0.0041	0.0009	5.2E-06	20.7531
M15500.m	rs10875102	A	G	0.5422	-0.0042	0.0009	4.63E-06	21.7778
M15500.m	rs10920115	T	G	0.5507	0.0044	0.0009	1.64E-06	23.9012
M15500.m	rs10956672	A	C	0.5436	0.004	0.0009	8.76E-06	19.7531
M15500.m	rs10993993	T	C	0.9709	0.0163	0.0033	9.8E-07	24.3976
M15500.m	rs11085752	A	C	0.5802	-0.004	0.0009	9.54E-06	19.7531
M15500.m	rs11085914	T	G	0.5541	-0.0042	0.0009	2.88E-06	21.7778
M15500.m	rs1109773	C	G	0.3724	0.004	0.0009	9.03E-06	19.7531
M15500.m	rs1114130	T	C	0.3401	-0.0042	0.0009	4.28E-06	21.7778
M15500.m	rs11183620	A	G	0.4676	0.005	0.0009	3E-08	30.8642
M15500.m	rs11223211	A	G	0.4713	0.0047	0.0009	1.71E-07	27.2716
M15500.m	rs1144963	T	C	0.5594	-0.0046	0.0009	2.48E-07	26.1235
M15500.m	rs1155457	T	C	0.4623	0.0041	0.0009	5.67E-06	20.7531
M15500.m	rs11589858	T	G	0.3573	-0.0041	0.0009	5.26E-06	20.7531
M15500.m	rs11601438	A	G	0.3912	-0.0042	0.0009	2.89E-06	21.7778
M15500.m	rs11620955	A	G	0.4767	-0.0051	0.0009	1.63E-08	32.1111
M15500.m	rs11620973	A	G	0.6326	-0.0051	0.0009	2.35E-08	32.1111
M15500.m	rs11630130	A	G	0.1395	0.0092	0.002	5.78E-06	21.16
M15500.m	rs11707952	A	G	0.5684	-0.0046	0.0009	3.93E-07	26.1235
M15500.m	rs1171618	T	C	0.6192	-0.0151	0.0009	1.58E-59	281.494
M15500.m	rs11915024	T	G	0.402	-0.0044	0.0009	9.59E-07	23.9012
M15500.m	rs11930385	T	C	0.4025	-0.0043	0.0009	2.05E-06	22.8272
M15500.m	rs11942631	A	C	0.0227	-0.0182	0.004	5.55E-06	20.7025
M15500.m	rs12197879	T	C	0.5976	-0.0044	0.0009	1.23E-06	23.9012
M15500.m	rs12201109	A	G	0.3703	-0.0045	0.0009	1.14E-06	25
M15500.m	rs12215339	T	C	0.9719	0.0321	0.0072	7.5E-06	19.8767
M15500.m	rs12245254	T	C	0.4414	0.0043	0.0009	1.92E-06	22.8272

M15500.m	rs12246131	A	T	0.778	0.008	0.0015	2.41E-07	28.4444
M15500.m	rs1225019	A	G	0.5791	-0.0043	0.0009	1.59E-06	22.8272
M15500.m	rs12356193	A	G	0.8362	0.0274	0.0016	3.69E-63	293.266
M15500.m	rs12417365	T	C	0.5209	-0.004	0.0009	7.82E-06	19.7531
M15500.m	rs1246267	A	G	0.591	0.0045	0.0009	1.09E-06	25
M15500.m	rs12484	T	C	0.427	-0.005	0.001	5.73E-07	25
M15500.m	rs1250070	A	G	0.3913	-0.0042	0.0009	8.56E-06	21.7778
M15500.m	rs12503744	A	C	0.0196	-0.0336	0.0073	4.82E-06	21.1852
M15500.m	rs12510909	T	C	0.5338	-0.0043	0.0009	1.84E-06	22.8272
M15500.m	rs12607576	T	C	0.5351	-0.004	0.0009	6.67E-06	19.7531
M15500.m	rs12627165	A	G	0.3466	0.0044	0.0009	3.46E-06	23.9012
M15500.m	rs12709393	T	G	0.6286	0.0053	0.0009	6.41E-09	34.679
M15500.m	rs12725366	A	G	0.4844	-0.0041	0.0009	5.55E-06	20.7531
M15500.m	rs12938026	T	C	0.6104	0.0046	0.0009	2.77E-07	26.1235
M15500.m	rs12980652	A	T	0.0318	-0.0145	0.0033	9.45E-06	19.3067
M15500.m	rs13162168	A	G	0.3813	-0.0041	0.0009	6.37E-06	20.7531
M15500.m	rs1317661	A	G	0.4794	0.0043	0.0009	1.86E-06	22.8272
M15500.m	rs13182512	A	T	0.3806	-0.005	0.0009	3.36E-08	30.8642
M15500.m	rs131919	T	C	0.3349	-0.0046	0.0009	9.01E-07	26.1235
M15500.m	rs13231650	T	C	0.4296	0.0041	0.0009	4.78E-06	20.7531
M15500.m	rs13284789	T	C	0.1397	0.0079	0.0018	9.19E-06	19.2623
M15500.m	rs1368388	A	G	0.4238	-0.0041	0.0009	4.62E-06	20.7531
M15500.m	rs140572	A	C	0.5115	0.0047	0.0009	2.19E-07	27.2716
M15500.m	rs1424937	A	G	0.4576	0.0042	0.0009	2.32E-06	21.7778
M15500.m	rs1430435	T	C	0.4932	0.0044	0.0009	1.26E-06	23.9012
M15500.m	rs1446555	A	C	0.9028	0.008	0.0017	3.21E-06	22.1453
M15500.m	rs1466788	A	G	0.4068	-0.0074	0.0009	3.05E-16	67.6049
M15500.m	rs1479614	A	G	0.6092	-0.0048	0.0009	9.93E-08	28.4444
M15500.m	rs1482567	T	C	0.6212	0.004	0.0009	9.3E-06	19.7531
M15500.m	rs1507101	A	G	0.5673	0.004	0.0009	9.63E-06	19.7531
M15500.m	rs1509657	T	C	0.4602	0.0041	0.0009	6.28E-06	20.7531
M15500.m	rs1537385	T	G	0.9609	0.0317	0.0065	1.19E-06	23.7844
M15500.m	rs1544412	T	C	0.3552	-0.0043	0.0009	2.68E-06	22.8272
M15500.m	rs1544811	A	G	0.4576	0.004	0.0009	8.99E-06	19.7531
M15500.m	rs1610649	T	C	0.6073	-0.0049	0.0009	7.92E-08	29.642
M15500.m	rs17065231	T	C	0.094	-0.0092	0.0017	7.16E-08	29.2872
M15500.m	rs17370344	A	C	0.6374	-0.0047	0.0009	1.66E-07	27.2716
M15500.m	rs1760903	A	G	0.4432	-0.0045	0.0009	1.01E-06	25
M15500.m	rs1769463	A	G	0.6289	-0.0042	0.0009	7.4E-06	21.7778
M15500.m	rs1866570	C	G	0.5838	-0.004	0.0009	7.76E-06	19.7531
M15500.m	rs1925611	A	G	0.4682	-0.0048	0.0009	2E-07	28.4444
M15500.m	rs1929425	T	C	0.3937	0.0043	0.0009	2.08E-06	22.8272
M15500.m	rs1936602	T	C	0.4194	0.0043	0.0009	2.53E-06	22.8272
M15500.m	rs1981138	T	G	0.433	0.0046	0.0009	3.31E-07	26.1235
M15500.m	rs2000364	T	G	0.6346	0.0041	0.0009	5.72E-06	20.7531
M15500.m	rs2068985	C	G	0.3461	-0.0041	0.0009	6.76E-06	20.7531
M15500.m	rs2114713	T	G	0.58	-0.005	0.0009	2.67E-08	30.8642
M15500.m	rs2130882	T	C	0.4864	0.0044	0.0009	1.11E-06	23.9012
M15500.m	rs2133813	T	C	0.4438	0.0041	0.0009	9.47E-06	20.7531
M15500.m	rs2164183	T	G	0.5129	-0.004	0.0009	9.8E-06	19.7531
M15500.m	rs2170082	A	G	0.5174	-0.0047	0.0009	2.16E-07	27.2716
M15500.m	rs220168	A	G	0.5152	-0.004	0.0009	9.33E-06	19.7531
M15500.m	rs2225978	A	C	0.5215	-0.004	0.0009	9.08E-06	19.7531
M15500.m	rs2279014	T	C	0.367	-0.005	0.0009	4.86E-08	30.8642
M15500.m	rs2286460	A	G	0.5399	-0.0045	0.0009	8.36E-07	25

M15500.m	rs2292871	A	G	0.5826	0.0045	0.0009	7.68E-07	25
M15500.m	rs2309339	C	G	0.6482	0.0041	0.0009	7.06E-06	20.7531
M15500.m	rs2327855	A	C	0.5796	0.004	0.0009	9.55E-06	19.7531
M15500.m	rs2362839	A	G	0.5974	-0.0041	0.0009	5.15E-06	20.7531
M15500.m	rs238340	A	C	0.569	-0.0042	0.0009	4.76E-06	21.7778
M15500.m	rs2394298	A	G	0.5879	0.0047	0.0009	1.51E-07	27.2716
M15500.m	rs2396004	A	G	0.4389	0.0049	0.0009	4.32E-08	29.642
M15500.m	rs2432560	T	G	0.6276	0.0042	0.0009	6.09E-06	21.7778
M15500.m	rs2451996	A	G	0.5799	0.0043	0.0009	1.67E-06	22.8272
M15500.m	rs246946	T	C	0.6832	0.0041	0.0009	9.26E-06	20.7531
M15500.m	rs2492788	T	C	0.5457	0.0049	0.0009	4.58E-08	29.642
M15500.m	rs2504927	T	C	0.4387	-0.0041	0.0009	4.35E-06	20.7531
M15500.m	rs2508305	T	C	0.8912	-0.0079	0.0017	4.05E-06	21.5952
M15500.m	rs2544371	A	G	0.5575	0.004	0.0009	8.69E-06	19.7531
M15500.m	rs2577264	A	G	0.3826	0.0043	0.0009	2.12E-06	22.8272
M15500.m	rs2595993	A	C	0.4967	-0.0042	0.0009	2.88E-06	21.7778
M15500.m	rs262273	C	G	0.3946	-0.0042	0.0009	2.76E-06	21.7778
M15500.m	rs2767290	A	G	0.3468	0.004	0.0009	8.33E-06	19.7531
M15500.m	rs2777788	A	G	0.636	-0.005	0.001	5.73E-07	25
M15500.m	rs2789599	A	G	0.5029	0.0043	0.0009	2.16E-06	22.8272
M15500.m	rs2793471	T	C	0.5669	0.0041	0.0009	7.23E-06	20.7531
M15500.m	rs288332	A	G	0.6294	-0.0043	0.0009	1.83E-06	22.8272
M15500.m	rs294280	T	C	0.3901	0.0042	0.0009	3.43E-06	21.7778
M15500.m	rs2961253	T	C	0.55	-0.005	0.001	5.73E-07	25
M15500.m	rs2962321	T	C	0.8968	-0.0077	0.0017	6.01E-06	20.5156
M15500.m	rs3127274	T	C	0.4172	0.0043	0.0009	2.09E-06	22.8272
M15500.m	rs3128061	T	G	0.3975	-0.0049	0.0009	5.74E-08	29.642
M15500.m	rs3736438	T	G	0.3889	-0.0057	0.0009	3.19E-10	40.1111
M15500.m	rs378999	T	C	0.6369	-0.0044	0.0009	1.2E-06	23.9012
M15500.m	rs3848713	T	C	0.4065	-0.0044	0.0009	1.11E-06	23.9012
M15500.m	rs3905228	A	C	0.4682	0.0041	0.0009	8.96E-06	20.7531
M15500.m	rs4078258	T	C	0.6332	-0.0041	0.0009	6.08E-06	20.7531
M15500.m	rs419291	T	C	0.3667	0.0079	0.0009	3.1E-18	77.0494
M15500.m	rs4284335	A	G	0.4121	-0.0046	0.0009	3.53E-07	26.1235
M15500.m	rs4305127	T	C	0.404	-0.005	0.001	5.73E-07	25
M15500.m	rs4436868	T	C	0.4973	0.0045	0.0009	4.29E-07	25
M15500.m	rs4504898	T	C	0.3869	-0.0041	0.0009	7.03E-06	20.7531
M15500.m	rs4525910	A	T	0.4052	-0.0043	0.0009	2.2E-06	22.8272
M15500.m	rs4531372	A	G	0.4345	-0.0045	0.0009	7.13E-07	25
M15500.m	rs459625	T	C	0.4343	-0.0049	0.0009	5.18E-08	29.642
M15500.m	rs4633738	A	C	0.4809	0.0041	0.0009	5.38E-06	20.7531
M15500.m	rs4634560	T	C	0.906	-0.009	0.002	6.8E-06	20.25
M15500.m	rs4665406	T	C	0.3652	-0.0043	0.0009	1.94E-06	22.8272
M15500.m	rs4669392	A	G	0.4262	-0.004	0.0009	9.29E-06	19.7531
M15500.m	rs4672450	T	C	0.4757	0.0044	0.0009	1.36E-06	23.9012
M15500.m	rs4674438	T	C	0.3643	0.0046	0.0009	2.93E-07	26.1235
M15500.m	rs4711152	T	C	0.543	0.0041	0.0009	5.06E-06	20.7531
M15500.m	rs4713396	A	C	0.4478	-0.0044	0.0009	1.15E-06	23.9012
M15500.m	rs4722438	A	G	0.4414	0.0044	0.0009	1.16E-06	23.9012
M15500.m	rs4764292	T	C	0.5797	0.0044	0.0009	9.76E-07	23.9012
M15500.m	rs478982	A	G	0.3378	0.004	0.0009	9.11E-06	19.7531
M15500.m	rs4793395	T	C	0.4703	0.0041	0.0009	6.58E-06	20.7531
M15500.m	rs480662	A	G	0.5952	-0.004	0.0009	9.73E-06	19.7531
M15500.m	rs4811828	T	C	0.8787	0.0077	0.0017	5.89E-06	20.5156
M15500.m	rs482275	T	C	0.5688	0.0041	0.0009	7.61E-06	20.7531

M15500.m	rs4860022	T	C	0.3362	-0.0055	0.0009	9.6E-10	37.3457
M15500.m	rs4881523	T	C	0.5372	0.0045	0.0009	5.95E-07	25
M15500.m	rs4888504	T	C	0.4223	0.0046	0.0009	2.68E-07	26.1235
M15500.m	rs4933391	A	G	0.3768	0.0042	0.0009	6E-06	21.7778
M15500.m	rs4940563	A	G	0.526	-0.005	0.001	5.73E-07	25
M15500.m	rs496150	A	G	0.6256	-0.004	0.0009	9.88E-06	19.7531
M15500.m	rs549386	T	C	0.6142	-0.0043	0.0009	1.63E-06	22.8272
M15500.m	rs561895	T	C	0.6577	-0.0042	0.0009	6.63E-06	21.7778
M15500.m	rs5999027	T	C	0.4322	-0.004	0.0009	9.52E-06	19.7531
M15500.m	rs6471514	T	C	0.5379	-0.0042	0.0009	2.73E-06	21.7778
M15500.m	rs6479648	A	G	0.4199	-0.0087	0.0009	8.4E-22	93.4444
M15500.m	rs6506736	A	G	0.5205	0.0042	0.0009	2.56E-06	21.7778
M15500.m	rs6535870	T	C	0.6539	-0.005	0.0009	5.5E-08	30.8642
M15500.m	rs6539588	T	C	0.3875	0.0043	0.0009	1.92E-06	22.8272
M15500.m	rs6554156	T	C	0.4037	0.0044	0.0009	1.12E-06	23.9012
M15500.m	rs6581712	A	G	0.6103	0.0043	0.0009	2.51E-06	22.8272
M15500.m	rs6596361	T	C	0.5945	0.0044	0.0009	9.19E-07	23.9012
M15500.m	rs6602640	T	G	0.8847	0.008	0.0017	2.5E-06	22.1453
M15500.m	rs6709816	A	G	0.1494	-0.0077	0.0016	1.86E-06	23.1602
M15500.m	rs6721330	A	C	0.5542	-0.0041	0.0009	4.92E-06	20.7531
M15500.m	rs6793266	T	C	0.3866	-0.0043	0.0009	2.92E-06	22.8272
M15500.m	rs6852374	A	G	0.5054	0.0041	0.0009	4.44E-06	20.7531
M15500.m	rs6862024	A	G	0.3691	-0.0055	0.0009	8.99E-10	37.3457
M15500.m	rs6866597	T	G	0.5194	0.0042	0.0009	3.56E-06	21.7778
M15500.m	rs6908972	T	G	0.082	-0.009	0.0018	7.99E-07	25
M15500.m	rs6937910	T	C	0.5268	-0.0043	0.0009	1.41E-06	22.8272
M15500.m	rs700760	T	G	0.5499	-0.0042	0.0009	2.57E-06	21.7778
M15500.m	rs7026148	T	C	0.386	-0.0045	0.0009	4.47E-07	25
M15500.m	rs7038757	T	C	0.6526	0.0041	0.0009	6.13E-06	20.7531
M15500.m	rs7098081	A	G	0.0469	0.0184	0.0025	3.1E-13	54.1696
M15500.m	rs7162803	A	G	0.5029	0.0048	0.0009	8.5E-08	28.4444
M15500.m	rs724752	T	C	0.3696	0.0043	0.0009	2.65E-06	22.8272
M15500.m	rs735315	T	C	0.4943	0.0054	0.0009	1.87E-09	36
M15500.m	rs7355681	A	G	0.5872	0.0041	0.0009	4.91E-06	20.7531
M15500.m	rs7435034	A	G	0.3617	-0.0042	0.0009	3.44E-06	21.7778
M15500.m	rs7461025	T	C	0.5871	0.0043	0.0009	2.7E-06	22.8272
M15500.m	rs7604464	A	G	0.9726	0.0152	0.0034	5.87E-06	19.9862
M15500.m	rs7632967	A	G	0.5465	-0.0043	0.0009	1.73E-06	22.8272
M15500.m	rs7662505	A	T	0.4045	-0.004	0.0009	9.98E-06	19.7531
M15500.m	rs7686890	T	C	0.3938	0.004	0.0009	7.99E-06	19.7531
M15500.m	rs769809	A	G	0.5723	0.0046	0.0009	3.5E-07	26.1235
M15500.m	rs7732790	A	C	0.59	-0.0042	0.0009	3.72E-06	21.7778
M15500.m	rs7737937	A	G	0.1512	-0.0109	0.0017	4.91E-11	41.1107
M15500.m	rs7760148	A	G	0.3554	-0.0042	0.0009	3.39E-06	21.7778
M15500.m	rs7799978	A	G	0.8174	0.0072	0.0016	4.54E-06	20.25
M15500.m	rs7837480	A	G	0.4784	-0.004	0.0009	9.3E-06	19.7531
M15500.m	rs7929841	T	C	0.6502	0.0043	0.0009	2.1E-06	22.8272
M15500.m	rs7940551	T	C	0.4352	-0.004	0.0009	8.33E-06	19.7531
M15500.m	rs7991989	T	C	0.6115	0.0041	0.0009	4.3E-06	20.7531
M15500.m	rs8043243	T	C	0.3641	-0.004	0.0009	8.41E-06	19.7531
M15500.m	rs8099879	A	C	0.383	0.005	0.001	5.73E-07	25
M15500.m	rs8127674	A	G	0.59	-0.0041	0.0009	5.85E-06	20.7531
M15500.m	rs8134847	T	C	0.397	0.0041	0.0009	8.38E-06	20.7531
M15500.m	rs8179836	C	G	0.6288	0.0048	0.0009	1.49E-07	28.4444
M15500.m	rs844016	T	C	0.5766	0.0047	0.0009	2.22E-07	27.2716

M15500.m	rs865488	T	C	0.6815	-0.0041	0.0009	6.65E-06	20.7531
M15500.m	rs879067	T	C	0.4361	0.004	0.0009	7.54E-06	19.7531
M15500.m	rs899584	T	C	0.356	-0.0047	0.0009	2.89E-07	27.2716
M15500.m	rs923075	T	C	0.6135	-0.0041	0.0009	4.81E-06	20.7531
M15500.m	rs9313491	C	G	0.643	0.0047	0.0009	6.09E-07	27.2716
M15500.m	rs9383693	T	C	0.5534	-0.0042	0.0009	3.57E-06	21.7778
M15500.m	rs9419445	T	C	0.5296	0.0042	0.0009	3.46E-06	21.7778
M15500.m	rs9467703	A	G	0.3745	-0.0045	0.0009	8.25E-07	25
M15500.m	rs948113	A	G	0.4012	-0.0043	0.0009	2.08E-06	22.8272
M15500.m	rs9530362	T	C	0.5726	-0.0044	0.0009	1.17E-06	23.9012
M15500.m	rs9530484	T	C	0.3535	0.0042	0.0009	4.45E-06	21.7778
M15500.m	rs9557806	A	G	0.5506	-0.0042	0.0009	2.4E-06	21.7778
M15500.m	rs9613019	T	C	0.4063	-0.0041	0.0009	7.42E-06	20.7531
M15500.m	rs968103	T	C	0.4463	0.0043	0.0009	1.5E-06	22.8272
M15500.m	rs977041	A	G	0.4355	0.0044	0.0009	1.27E-06	23.9012
M15500.m	rs9784110	T	C	0.6096	-0.0042	0.0009	3.97E-06	21.7778
M15500.m	rs9819702	T	C	0.3613	-0.0042	0.0009	5.18E-06	21.7778
M15500.m	rs9838540	A	C	0.6102	0.0047	0.0009	1.8E-07	27.2716
M15500.m	rs9842133	T	C	0.6649	0.0064	0.0009	4.2E-12	50.5679
M15500.m	rs9863328	T	C	0.433	0.004	0.0009	9.08E-06	19.7531
M15500.m	rs9880736	A	C	0.5431	-0.0044	0.0009	1.51E-06	23.9012
M15500.m	rs989681	T	C	0.5177	0.0041	0.0009	7.36E-06	20.7531
M15500.m	rs9942113	A	C	0.3764	-0.0049	0.0009	6E-08	29.642
M15500.m	rs9947578	A	G	0.5631	0.0043	0.0009	3.94E-06	22.8272
M15500.m	rs9960949	A	G	0.4788	-0.0042	0.0009	8.83E-06	21.7778
M15500.m	rs996408	T	C	0.5159	-0.0046	0.0009	3.67E-07	26.1235
M15500.m	rs9997562	T	C	0.5196	0.0043	0.0009	1.86E-06	22.8272
M15506.m	rs10194073	A	C	0.7918	-0.007	0.0015	6.33E-06	21.7778
M15506.m	rs11058334	A	G	0.0228	-0.0217	0.0047	4.26E-06	21.3169
M15506.m	rs11656215	T	C	0.498	-0.0079	0.0015	7.48E-08	27.7378
M15506.m	rs11930490	A	G	0.803	-0.0076	0.0016	1.52E-06	22.5625
M15506.m	rs12039115	T	C	0.1289	-0.0088	0.0017	1.93E-07	26.7958
M15506.m	rs12105001	A	G	0.0487	-0.0131	0.0029	7.55E-06	20.4055
M15506.m	rs1417497	C	G	0.072	-0.0106	0.0023	5.35E-06	21.2401
M15506.m	rs17497383	T	G	0.0722	0.0157	0.0034	5.34E-06	21.3227
M15506.m	rs17642749	T	G	0.9518	-0.0165	0.0035	2.68E-06	22.2245
M15506.m	rs2080224	A	G	0.9304	-0.0112	0.0024	1.97E-06	21.7778
M15506.m	rs2297615	A	T	0.7752	0.007	0.0016	8.17E-06	19.1406
M15506.m	rs2824226	A	G	0.8186	0.0074	0.0016	3.19E-06	21.3906
M15506.m	rs3786284	A	G	0.1481	0.0077	0.0016	2.04E-06	23.1602
M15506.m	rs4242454	A	T	0.1377	0.0077	0.0016	3.25E-06	23.1602
M15506.m	rs4293599	C	G	0.1775	0.0071	0.0016	5.93E-06	19.6914
M15506.m	rs4487482	A	G	0.8039	0.0081	0.0015	1.82E-07	29.16
M15506.m	rs4538138	T	G	0.1884	0.0071	0.0016	6.09E-06	19.6914
M15506.m	rs4784175	A	T	0.8586	-0.0091	0.002	8.64E-06	20.7025
M15506.m	rs715	T	C	0.7088	0.008	0.0015	9.11E-08	28.4444
M15506.m	rs7215931	A	G	0.2013	0.0079	0.0016	1.29E-06	24.3789
M15506.m	rs7687295	A	G	0.0249	0.0211	0.0046	4.32E-06	21.0402
M15506.m	rs8003323	C	G	0.987	0.031	0.007	9.49E-06	19.6122
M15506.m	rs8033100	T	C	0.8565	-0.0083	0.0016	4.94E-07	26.9102
M15506.m	rs9321388	A	G	0.1772	-0.0072	0.0016	7.58E-06	20.25
M15506.m	rs9663814	A	G	0.6163	0.0069	0.0014	1.1E-06	24.2908
M15630.m	rs10027133	T	C	0.7027	-0.0203	0.0043	2.09E-06	22.2872
M15630.m	rs1036113	T	C	0.2847	-0.1052	0.0043	9.8E-135	598.542
M15630.m	rs11679923	A	G	0.0316	-0.1239	0.0227	4.99E-08	29.7914

M15630.m	rs11692971	T	C	0.0955	-0.0352	0.0071	8.4E-07	24.5793
M15630.m	rs12644208	A	G	0.6293	-0.0197	0.0043	5.12E-06	20.9892
M15630.m	rs13415017	T	C	0.69	0.034	0.006	1.46E-08	32.1111
M15630.m	rs1355074	A	G	0.2565	-0.0193	0.0043	8.81E-06	20.1455
M15630.m	rs1509525	A	G	0.2972	-0.019	0.0043	7.59E-06	19.5241
M15630.m	rs1653259	A	G	0.0653	0.0491	0.0089	4.18E-08	30.4357
M15630.m	rs17075971	A	G	0.1666	0.0264	0.0056	2.23E-06	22.2245
M15630.m	rs17314234	T	C	0.1023	-0.0324	0.0069	2.43E-06	22.0491
M15630.m	rs17348756	T	C	0.1081	0.0475	0.0067	1.87E-12	50.2618
M15630.m	rs17349853	T	G	0.9298	-0.0738	0.009	1.82E-16	67.24
M15630.m	rs1881244	A	G	0.063	-0.1907	0.0085	2.9E-112	503.342
M15630.m	rs1937336	A	T	0.6597	0.0189	0.0043	9.07E-06	19.3191
M15630.m	rs2028364	A	G	0.9783	0.0807	0.0181	8.27E-06	19.8788
M15630.m	rs2043099	A	G	0.2713	-0.0514	0.0043	1.09E-32	142.886
M15630.m	rs2268365	T	C	0.8731	0.0289	0.0059	1.11E-06	23.9934
M15630.m	rs4852937	T	C	0.8587	-0.0268	0.0059	5.29E-06	20.6332
M15630.m	rs6414862	A	G	0.6975	-0.0202	0.0044	3.34E-06	21.0764
M15630.m	rs6546815	A	T	0.8703	-0.0366	0.0063	4.6E-09	33.7506
M15630.m	rs7309949	A	G	0.0184	-0.132	0.0277	1.93E-06	22.7085
M15630.m	rs7553988	A	G	0.2898	0.0203	0.0043	2.22E-06	22.2872
M15630.m	rs7573275	A	G	0.2739	-0.1511	0.0042	1E-200	1294.29
M15630.m	rs9610113	A	G	0.3153	-0.0208	0.0043	1.25E-06	23.3986
M15650.m	rs10513863	A	G	0.2661	0.0055	0.0012	7.33E-06	21.0069
M15650.m	rs12662536	A	T	0.2919	-0.0056	0.0012	6.66E-06	21.7778
M15650.m	rs1490672	T	C	0.2361	-0.0108	0.0022	1.13E-06	24.0992
M15650.m	rs315988	T	C	0.2367	0.0072	0.0013	1.08E-08	30.6746
M15650.m	rs369402	A	G	0.3892	-0.0055	0.0012	5.86E-06	21.0069
M15650.m	rs414540	A	G	0.782	-0.0057	0.0013	6.97E-06	19.2249
M15650.m	rs6094988	C	G	0.234	0.0059	0.0013	3.14E-06	20.5976
M15650.m	rs6108469	A	G	0.7569	0.0056	0.0012	6.36E-06	21.7778
M15676.m	rs10082580	A	T	0.0409	-0.0259	0.005	1.88E-07	26.8324
M15676.m	rs10508868	A	G	0.8446	0.0118	0.0025	1.64E-06	22.2784
M15676.m	rs10520795	T	C	0.0131	-0.0842	0.0189	8.27E-06	19.8473
M15676.m	rs11084396	T	C	0.544	-0.011	0.002	3.8E-08	30.25
M15676.m	rs11563662	A	G	0.0136	0.0657	0.0139	2.28E-06	22.3409
M15676.m	rs11615860	A	G	0.3917	-0.009	0.0017	1.34E-07	28.0277
M15676.m	rs11954603	T	C	0.9767	0.0306	0.0067	4.83E-06	20.859
M15676.m	rs1196687	T	C	0.6211	0.0078	0.0017	5.82E-06	21.0519
M15676.m	rs12220364	A	G	0.4107	-0.0077	0.0017	8.34E-06	20.5156
M15676.m	rs12455580	T	C	0.3104	0.0117	0.0023	4.39E-07	25.8771
M15676.m	rs1260326	T	C	0.406	0.01	0.0017	4.57E-09	34.6021
M15676.m	rs12616616	A	G	0.5527	0.0079	0.0017	3.83E-06	21.5952
M15676.m	rs1440581	T	C	0.4586	-0.014	0.0017	1.21E-16	67.8201
M15676.m	rs16870228	A	G	0.1456	0.0129	0.0025	3.23E-07	26.6256
M15676.m	rs17329328	T	C	0.4292	0.0081	0.0017	3.49E-06	22.7024
M15676.m	rs1902781	T	G	0.4272	0.0076	0.0017	6.71E-06	19.9862
M15676.m	rs219908	T	C	0.407	-0.0076	0.0017	8.79E-06	19.9862
M15676.m	rs2404519	A	G	0.1587	0.0117	0.0024	1.6E-06	23.7656
M15676.m	rs2825115	T	C	0.568	0.009	0.002	6.8E-06	20.25
M15676.m	rs429946	T	C	0.0602	0.0178	0.004	8.06E-06	19.8025
M15676.m	rs4570993	T	C	0.5322	0.0081	0.0017	2.43E-06	22.7024
M15676.m	rs6596867	A	C	0.543	0.009	0.002	6.8E-06	20.25
M15676.m	rs6846111	T	C	0.1508	-0.012	0.0026	5.46E-06	21.3018
M15676.m	rs699530	T	G	0.5636	-0.008	0.0017	2.78E-06	22.1453
M15676.m	rs7006068	T	C	0.6266	0.0082	0.0017	2.08E-06	23.2664

M15676.m	rs703147	T	C	0.3801	0.0081	0.0017	2.41E-06	22.7024
M15676.m	rs7222769	T	C	0.8765	0.0141	0.003	2.97E-06	22.09
M15676.m	rs7310929	T	C	0.6256	-0.0077	0.0017	9.38E-06	20.5156
M15676.m	rs7405977	T	C	0.3918	-0.0114	0.0023	1.17E-06	24.5671
M15676.m	rs7479540	T	C	0.4495	-0.0081	0.0017	2.35E-06	22.7024
M15676.m	rs7648992	T	C	0.3947	0.0083	0.0017	1.53E-06	23.8374
M15676.m	rs7761964	T	C	0.6305	0.0175	0.0033	1.71E-07	28.1221
M15676.m	rs7986369	A	C	0.3795	-0.008	0.0017	2.97E-06	22.1453
M15676.m	rs938391	A	G	0.3682	0.0079	0.0018	7.23E-06	19.2623
M15676.m	rs963913	A	G	0.4455	-0.0076	0.0017	9.04E-06	19.9862
M15677.m	rs11174282	T	C	0.0957	-0.0615	0.0137	6.89E-06	20.1516
M15677.m	rs17241253	T	C	0.8117	0.0481	0.0107	7.07E-06	20.208
M15677.m	rs2126638	A	G	0.9266	0.0816	0.0163	5.78E-07	25.0614
M15677.m	rs2969612	T	C	0.1583	-0.0496	0.0108	4.75E-06	21.0919
M15677.m	rs3783141	T	C	0.8708	0.0559	0.0125	7.75E-06	19.9988
M15677.m	rs7151608	A	G	0.8127	0.0497	0.0106	2.86E-06	21.9837
M15677.m	rs8048366	A	G	0.4796	-0.0363	0.0082	9.74E-06	19.5968
M15677.m	rs9915627	A	C	0.1829	0.1117	0.0243	4.29E-06	21.1297
M15749.m	rs10431948	A	G	0.354	0.02	0.0045	7.26E-06	19.7531
M15749.m	rs10493278	A	T	0.8124	0.0243	0.0054	5.83E-06	20.25
M15749.m	rs11647589	A	G	0.7151	0.0303	0.0045	2.85E-11	45.3378
M15749.m	rs11847947	T	C	0.3031	-0.0217	0.0045	1.54E-06	23.2538
M15749.m	rs11983172	A	G	0.1593	-0.0326	0.0073	8.63E-06	19.943
M15749.m	rs2441128	A	T	0.2361	0.0239	0.0054	8.57E-06	19.5888
M15749.m	rs4772056	C	G	0.907	-0.034	0.0076	8.45E-06	20.0139
M15749.m	rs584630	A	C	0.1052	-0.0318	0.007	6.27E-06	20.6376
M15749.m	rs609236	C	G	0.1512	0.028	0.0062	5.57E-06	20.3954
M15749.m	rs6497490	T	G	0.882	-0.0477	0.0075	1.91E-10	40.4496
M15749.m	rs6851666	A	T	0.9632	-0.0526	0.0114	4.06E-06	21.2893
M15749.m	rs6891705	A	C	0.6963	0.02	0.0045	9.37E-06	19.7531
M15749.m	rs763753	A	G	0.1367	0.028	0.0063	8.88E-06	19.7531
M15749.m	rs9355342	A	G	0.9692	0.1456	0.0281	2.19E-07	26.8479
M15749.m	rs9402497	A	G	0.252	0.024	0.0054	8.93E-06	19.7531
M15749.m	rs987313	A	G	0.0159	0.1845	0.0416	9.18E-06	19.6701
M15753.x	rs10400902	A	G	0.3089	-0.0251	0.0052	1.62E-06	23.2992
M15753.x	rs10847782	A	G	0.2367	0.0277	0.006	4.28E-06	21.3136
M15753.x	rs11535191	A	G	0.8135	0.0277	0.0062	7.48E-06	19.9607
M15753.x	rs12466421	A	G	0.6945	0.0279	0.006	3.96E-06	21.6225
M15753.x	rs12660977	T	G	0.1536	0.0405	0.0089	5.67E-06	20.7076
M15753.x	rs1343188	T	C	0.5646	-0.0263	0.0057	4.17E-06	21.2893
M15753.x	rs1878105	T	C	0.0183	0.0966	0.0216	7.38E-06	20.0008
M15753.x	rs2102	A	G	0.0768	0.0425	0.0096	9.45E-06	19.5991
M15753.x	rs3892034	A	T	0.7703	-0.0273	0.006	4.75E-06	20.7025
M15753.x	rs4338651	A	G	0.026	-0.088	0.018	1.01E-06	23.9012
M15753.x	rs4524676	A	G	0.1852	-0.0278	0.0063	9.31E-06	19.4719
M15753.x	rs475322	A	C	0.016	0.117	0.024	1.09E-06	23.7656
M15753.x	rs554900	A	G	0.0339	0.062	0.014	9.28E-06	19.6122
M15753.x	rs6739717	T	C	0.6235	0.0441	0.0099	8.94E-06	19.843
M15753.x	rs7102716	A	T	0.7654	0.027	0.006	7.85E-06	20.25
M15778.x	rs10515641	A	G	0.2493	-0.0086	0.0019	4.37E-06	20.4875
M15778.x	rs10745735	A	G	0.907	0.0133	0.0027	1.15E-06	24.2647
M15778.x	rs11013865	T	C	0.214	0.0084	0.0019	5.42E-06	19.5457
M15778.x	rs1151659	T	C	0.2122	-0.0219	0.0049	8.24E-06	19.9754
M15778.x	rs11838539	A	G	0.0205	0.0284	0.0061	3.38E-06	21.6759
M15778.x	rs12132780	T	C	0.3331	0.0082	0.0018	6.82E-06	20.7531

M15778.x	rs12150754	C	G	0.7851	0.0083	0.0019	7.7E-06	19.0831
M15778.x	rs12637854	A	T	0.8926	-0.0138	0.0027	4.33E-07	26.1235
M15778.x	rs12783167	T	C	0.2909	-0.0081	0.0018	6.56E-06	20.25
M15778.x	rs12899069	T	C	0.7665	0.0131	0.0029	8.58E-06	20.4055
M15778.x	rs13151688	T	C	0.3407	0.0085	0.0018	3.81E-06	22.2994
M15778.x	rs159486	T	C	0.1205	0.012	0.0027	5.91E-06	19.7531
M15778.x	rs16876152	T	G	0.2054	-0.0089	0.0019	1.65E-06	21.9418
M15778.x	rs1791600	T	C	0.2072	-0.0087	0.0019	4.09E-06	20.9668
M15778.x	rs1866411	A	C	0.3472	-0.0085	0.0018	2.81E-06	22.2994
M15778.x	rs2317834	T	G	0.9041	0.0129	0.0027	2.31E-06	22.8272
M15778.x	rs247616	T	C	0.322	0.0098	0.0019	1.32E-07	26.6039
M15778.x	rs310528	A	G	0.2233	0.0086	0.0018	2.59E-06	22.8272
M15778.x	rs310597	A	G	0.6532	-0.0088	0.0018	1.3E-06	23.9012
M15778.x	rs3112496	T	C	0.1331	0.0127	0.0026	1.13E-06	23.8595
M15778.x	rs3131535	A	G	0.9652	-0.0205	0.0045	6.3E-06	20.7531
M15778.x	rs3765035	A	G	0.3438	0.0092	0.0018	3.76E-07	26.1235
M15778.x	rs4353947	A	G	0.2304	-0.0082	0.0018	8.7E-06	20.7531
M15778.x	rs4386719	T	C	0.7737	-0.0083	0.0019	7.38E-06	19.0831
M15778.x	rs4515826	T	G	0.1021	-0.0132	0.0027	1.31E-06	23.9012
M15778.x	rs4672488	A	G	0.2854	0.0086	0.0019	4.08E-06	20.4875
M15778.x	rs4741625	A	G	0.2777	0.0085	0.0018	3.5E-06	22.2994
M15778.x	rs4850051	A	G	0.2082	-0.0086	0.0019	3.84E-06	20.4875
M15778.x	rs4852620	A	G	0.1096	0.0141	0.0027	1.73E-07	27.2716
M15778.x	rs6841005	T	C	0.3264	0.0088	0.0018	1.15E-06	23.9012
M15778.x	rs724165	A	G	0.5944	-0.008	0.0018	7.54E-06	19.7531
M15778.x	rs7283154	T	C	0.2516	-0.0087	0.0018	2.32E-06	23.3611
M15778.x	rs7513165	T	C	0.7526	0.0086	0.0018	2.83E-06	22.8272
M15778.x	rs7561849	A	G	0.2168	0.009	0.0018	1.01E-06	25
M15778.x	rs8008040	T	C	0.7276	-0.0087	0.0018	2.06E-06	23.3611
M15778.x	rs9314172	C	G	0.6969	-0.0084	0.0018	4.5E-06	21.7778
M15778.x	rs9382939	T	C	0.9661	0.0255	0.0058	1E-05	19.3297
M15778.x	rs9394526	A	G	0.0529	-0.0187	0.0037	4.28E-07	25.5435
M15778.x	rs9514739	C	G	0.8962	-0.0122	0.0027	6.1E-06	20.417
M15778.x	rs969075	T	C	0.3346	-0.0086	0.0018	2.63E-06	22.8272
M15778.x	rs9859467	A	G	0.037	-0.0203	0.0045	5.75E-06	20.3501
M15958.m	rs10520388	T	G	0.1733	0.0219	0.0048	4.92E-06	20.8164
M15958.m	rs16885269	T	C	0.1333	0.0255	0.0057	8.07E-06	20.0139
M15958.m	rs17683199	A	G	0.0385	-0.0446	0.0094	1.88E-06	22.512
M15958.m	rs201060	A	G	0.9465	0.0367	0.0083	9.75E-06	19.5513
M15958.m	rs2133896	T	G	0.0416	0.0469	0.0099	2.36E-06	22.4427
M15958.m	rs2345834	T	C	0.6652	0.0176	0.0039	5.45E-06	20.3655
M15958.m	rs2579931	A	G	0.1044	0.0269	0.0059	4.23E-06	20.7874
M15958.m	rs4296931	A	G	0.0244	0.1523	0.0334	5.1E-06	20.7925
M15958.m	rs7243187	A	C	0.2661	-0.0455	0.0099	3.87E-06	21.1228
M15958.m	rs7580111	A	T	0.3967	0.0213	0.0047	4.94E-06	20.5383
M15990.m	rs1076302	A	G	0.201	0.018	0.004	6.8E-06	20.25
M15990.m	rs11025430	A	G	0.4231	-0.0118	0.0026	8.33E-06	20.5976
M15990.m	rs1379617	T	C	0.7847	-0.0156	0.0035	6.15E-06	19.8661
M15990.m	rs16876953	A	G	0.0818	0.0232	0.0052	8.69E-06	19.9053
M15990.m	rs170555	T	C	0.5607	-0.0253	0.0055	3.84E-06	21.16
M15990.m	rs17312926	A	G	0.3493	0.016	0.0033	1.42E-06	23.5078
M15990.m	rs1923140	A	G	0.5322	0.0131	0.0027	8.57E-07	23.5405
M15990.m	rs1955742	A	T	0.599	-0.012	0.0026	6.24E-06	21.3018
M15990.m	rs1978450	A	C	0.017	0.1329	0.0291	4.75E-06	20.8576
M15990.m	rs2834399	T	G	0.4704	0.0124	0.0027	3.23E-06	21.0919

M15990.m	rs309293	T	C	0.4315	0.0256	0.0057	6.9E-06	20.1711
M15990.m	rs382559	A	G	0.3861	0.0262	0.0059	9.98E-06	19.7196
M15990.m	rs633114	T	C	0.3884	-0.0152	0.0033	3.91E-06	21.2158
M15990.m	rs7260033	T	C	0.4757	-0.0117	0.0026	8.79E-06	20.25
M15990.m	rs7563729	A	G	0.5161	0.0116	0.0026	9.89E-06	19.9053
M15990.m	rs7711255	A	G	0.7968	-0.0173	0.0034	4.92E-07	25.8901
M15990.m	rs7738342	A	G	0.2185	-0.0153	0.0035	9.17E-06	19.1094
M15990.m	rs7788421	A	G	0.9134	-0.0245	0.0054	5.18E-06	20.5847
M15990.m	rs7795076	T	C	0.8166	-0.017	0.0035	1.62E-06	23.5918
M15996.m	rs12954899	A	G	0.8905	-0.0254	0.0057	8.84E-06	19.8572
M15996.m	rs4690523	T	C	0.5243	0.0182	0.0037	1.04E-06	24.1958
M15996.m	rs6854832	T	C	0.1513	0.0472	0.0103	4.98E-06	20.9995
M15996.m	rs7150776	T	C	0.1105	0.0279	0.0057	9.22E-07	23.9584
M16634.m	rs10415535	A	G	0.167	0.036	0.008	6.8E-06	20.25
M16634.m	rs11747325	T	C	0.2853	-0.0228	0.0051	6.45E-06	19.9862
M16634.m	rs12197075	C	G	0.9687	0.0598	0.0134	8.05E-06	19.9156
M16634.m	rs12949918	T	C	0.584	-0.0201	0.0043	2.36E-06	21.8502
M16634.m	rs1598817	T	G	0.9624	0.0541	0.012	7.1E-06	20.3251
M16634.m	rs16959796	T	C	0.951	0.058	0.013	8.14E-06	19.9053
M16634.m	rs2216140	A	G	0.0341	-0.1026	0.0219	2.73E-06	21.9486
M16634.m	rs7857624	T	C	0.9289	0.0395	0.0087	6.22E-06	20.6137
M16634.m	rs8061140	A	G	0.9587	-0.0498	0.0112	8.36E-06	19.7707
M16634.m	rs9904521	A	G	0.9525	0.0892	0.02	7.93E-06	19.8916
M16816.m	rs10254175	T	C	0.2329	-0.0157	0.0034	3.93E-06	21.3227
M16816.m	rs10974528	A	G	0.2038	0.0155	0.0035	8.88E-06	19.6122
M16816.m	rs10980324	T	C	0.9677	0.0411	0.0084	9.21E-07	23.9401
M16816.m	rs11935679	A	G	0.202	-0.0155	0.0034	6.8E-06	20.7829
M16816.m	rs12629505	A	G	0.3983	0.0128	0.0026	1.1E-06	24.2367
M16816.m	rs1517397	T	C	0.3986	0.0122	0.0027	4.44E-06	20.417
M16816.m	rs1552422	A	G	0.5052	-0.0119	0.0026	6E-06	20.9482
M16816.m	rs17655603	T	C	0.9567	0.0904	0.0205	9.91E-06	19.4459
M16816.m	rs1861007	C	G	0.1925	-0.0181	0.004	7.81E-06	20.4756
M16816.m	rs1861371	A	G	0.1774	0.0165	0.0035	2.35E-06	22.2245
M16816.m	rs2473097	T	C	0.7894	0.0154	0.0034	5.9E-06	20.5156
M16816.m	rs4665701	T	C	0.4689	-0.0121	0.0027	4.9E-06	20.0837
M16816.m	rs6810964	A	G	0.5495	0.0125	0.0026	2.08E-06	23.1139
M16816.m	rs8105530	T	C	0.5286	0.0128	0.0028	4.85E-06	20.898
M16818.m	rs10966194	A	G	0.7354	0.0159	0.0036	9.77E-06	19.5069
M16818.m	rs11022215	T	C	0.9308	0.0572	0.0126	5.33E-06	20.6087
M16818.m	rs11047488	A	T	0.131	-0.044	0.0094	3.14E-06	21.9104
M16818.m	rs11597065	T	C	0.7513	-0.018	0.0036	5.27E-07	25
M16818.m	rs12314479	C	G	0.0853	0.0283	0.006	2.76E-06	22.2469
M16818.m	rs3113185	T	C	0.6254	-0.0175	0.0036	9.5E-07	23.6304
M16818.m	rs4268384	A	G	0.0624	0.029	0.0064	5.01E-06	20.5322
M16818.m	rs4332594	T	C	0.246	-0.0177	0.0036	1.11E-06	24.1736
M16818.m	rs4862124	A	T	0.945	0.0348	0.0073	1.76E-06	22.7255
M16818.m	rs4888946	A	T	0.7754	-0.0161	0.0036	8.78E-06	20.0008
M16818.m	rs7084948	T	C	0.067	-0.0322	0.0067	1.54E-06	23.0973
M16821.m	rs10043	A	C	0.1898	-0.0157	0.0035	6.4E-06	20.1216
M16821.m	rs10104759	T	G	0.6428	-0.0124	0.0027	3.57E-06	21.0919
M16821.m	rs11643275	C	G	0.7236	-0.0128	0.0027	2.04E-06	22.4746
M16821.m	rs12446555	T	G	0.0671	-0.029	0.0065	9.02E-06	19.9053
M16821.m	rs12552851	T	C	0.7391	0.0131	0.0027	1.74E-06	23.5405
M16821.m	rs13357681	T	C	0.2441	0.0129	0.0027	1.96E-06	22.8272
M16821.m	rs13440146	T	G	0.0579	-0.0292	0.006	1.29E-06	23.6844

M16821.m	rs17450296	T	G	0.9051	-0.0219	0.0045	1.11E-06	23.6844
M16821.m	rs1781619	T	C	0.751	0.016	0.003	9.64E-08	28.4444
M16821.m	rs1879207	C	G	0.2501	0.0128	0.0027	2.73E-06	22.4746
M16821.m	rs1939990	T	C	0.2409	-0.0136	0.0027	6.91E-07	25.3717
M16821.m	rs2043660	A	T	0.8396	0.036	0.0078	4.03E-06	21.3018
M16821.m	rs2642169	A	G	0.5682	0.0118	0.0027	8.63E-06	19.1001
M16821.m	rs2863060	A	G	0.2677	-0.0132	0.0027	1.42E-06	23.9012
M16821.m	rs3778464	T	C	0.9795	0.0455	0.0096	2.18E-06	22.4637
M16821.m	rs4510422	T	C	0.196	-0.0158	0.0035	7.04E-06	20.3788
M16821.m	rs4976897	T	C	0.7073	0.0133	0.0027	1.16E-06	24.2647
M16821.m	rs6012412	A	G	0.6677	-0.0128	0.0028	4.01E-06	20.898
M16821.m	rs7604529	A	G	0.8443	-0.0161	0.0036	8.04E-06	20.0008
M16821.m	rs7938161	A	G	0.514	0.0145	0.0033	9.94E-06	19.3067
M16821.m	rs9890574	A	T	0.7604	0.0132	0.0028	2.99E-06	22.2245
M16821.m	rs9926816	A	G	0.8582	-0.0164	0.0036	5.19E-06	20.7531
M16822.m	rs10065951	T	C	0.3122	0.0112	0.0025	9.19E-06	20.0704
M16822.m	rs1009184	T	G	0.5453	-0.0123	0.0025	6E-07	24.2064
M16822.m	rs1030420	T	C	0.1884	-0.0221	0.0032	1.02E-11	47.6963
M16822.m	rs11856353	T	C	0.8796	-0.0162	0.0035	2.87E-06	21.4237
M16822.m	rs13332924	A	C	0.2301	0.0132	0.0026	4.76E-07	25.7751
M16822.m	rs1525549	A	G	0.1421	0.0166	0.0034	1.11E-06	23.8374
M16822.m	rs2333040	A	G	0.4575	0.0115	0.0025	4.16E-06	21.16
M16822.m	rs2372342	A	G	0.9307	0.0217	0.0049	9.44E-06	19.6122
M16822.m	rs2632020	T	C	0.4506	0.0109	0.0025	9.83E-06	19.0096
M16822.m	rs4273762	T	C	0.1	-0.0182	0.0041	8.75E-06	19.7049
M16822.m	rs4325745	T	C	0.596	-0.014	0.003	3.06E-06	21.7778
M16822.m	rs504192	A	G	0.8399	-0.0151	0.0034	7.68E-06	19.724
M16822.m	rs504899	T	C	0.0734	-0.0232	0.0048	1.42E-06	23.3611
M16822.m	rs6024831	A	G	0.9232	-0.0189	0.0042	7.09E-06	20.25
M16822.m	rs6748268	T	C	0.6942	0.0112	0.0025	8.73E-06	20.0704
M16822.m	rs6959584	T	C	0.1278	0.0173	0.0035	6.1E-07	24.4318
M16822.m	rs7699551	T	C	0.5871	-0.0118	0.0025	3.31E-06	22.2784
M16822.m	rs8112502	A	G	0.0918	-0.0212	0.0041	2.64E-07	26.7365
M16822.m	rs9393768	A	G	0.3579	0.0114	0.0025	6.24E-06	20.7936
M16822.m	rs9525877	A	G	0.7816	-0.0118	0.0026	6.5E-06	20.5976
M16822.m	rs9604751	A	G	0.1344	0.018	0.0038	2.04E-06	22.4377
M16823.m	rs11111705	A	G	0.6141	-0.0492	0.0111	8.9E-06	19.6465
M16823.m	rs11721313	T	C	0.0704	-0.0959	0.0217	9.83E-06	19.5307
M16823.m	rs12584638	A	G	0.6398	-0.0945	0.0209	6.24E-06	20.4442
M16823.m	rs13336955	T	C	0.0541	0.1061	0.0233	5.48E-06	20.7357
M16823.m	rs1373765	T	C	0.7973	-0.0637	0.0141	6.42E-06	20.4099
M16823.m	rs17579664	T	G	0.2583	-0.0601	0.0126	1.84E-06	22.7514
M16823.m	rs2442790	T	C	0.0288	0.3427	0.0767	7.99E-06	19.9635
M16823.m	rs269263	T	C	0.3981	-0.0487	0.0105	3.32E-06	21.5119
M16823.m	rs343836	T	C	0.9796	-0.1801	0.0385	2.83E-06	21.883
M16823.m	rs6008912	A	G	0.159	0.1439	0.0296	1.19E-06	23.6341
M16823.m	rs7679137	T	C	0.9857	-0.2217	0.0455	1.11E-06	23.7415
M17799.x	rs10005912	T	G	0.9533	0.1151	0.0252	5.09E-06	20.8617
M17799.x	rs10015080	A	C	0.9289	0.1094	0.0176	5.05E-10	38.6375
M17799.x	rs10068843	A	C	0.046	-0.1072	0.0239	7.43E-06	20.1184
M17799.x	rs1015859	T	C	0.8882	0.0847	0.0168	4.41E-07	25.4184
M17799.x	rs10421478	T	C	0.153	-0.0639	0.0143	7.68E-06	19.9678
M17799.x	rs10425230	T	C	0.0447	-0.1209	0.0239	4.21E-07	25.5892
M17799.x	rs10816183	A	G	0.9623	0.1493	0.0287	1.97E-07	27.0617
M17799.x	rs10875231	T	G	0.2715	-0.0515	0.0113	5.43E-06	20.771

M17799.x	rs10900676	C	G	0.987	-0.305	0.067	5.31E-06	20.7229
M17799.x	rs10976597	T	C	0.92	0.1141	0.0235	1.16E-06	23.5741
M17799.x	rs10988783	T	C	0.04	-0.1623	0.0299	5.62E-08	29.4642
M17799.x	rs10998867	A	G	0.96	0.1623	0.0299	5.62E-08	29.4642
M17799.x	rs11127379	A	G	0.0764	-0.101	0.0196	2.44E-07	26.554
M17799.x	rs11129686	T	C	0.96	0.1623	0.0299	5.62E-08	29.4642
M17799.x	rs11158062	A	G	0.5	-0.0638	0.0141	5.94E-06	20.474
M17799.x	rs11161725	A	C	0.04	-0.1623	0.0299	5.62E-08	29.4642
M17799.x	rs11265416	A	G	0.9118	0.0995	0.0202	8.3E-07	24.2629
M17799.x	rs1138253	A	G	0.4619	0.0579	0.0129	7.88E-06	20.1455
M17799.x	rs11602382	A	G	0.0779	-0.1147	0.0224	3.2E-07	26.2199
M17799.x	rs11612437	A	G	0.0367	-0.1389	0.0307	5.91E-06	20.4705
M17799.x	rs1165210	T	C	0.04	-0.1214	0.027	6.71E-06	20.2167
M17799.x	rs11703458	A	G	0.036	-0.1963	0.0432	5.46E-06	20.6478
M17799.x	rs11803192	T	C	0.9061	0.1002	0.0172	6.1E-09	33.9374
M17799.x	rs11819046	T	C	0.0689	-0.1237	0.0205	1.68E-09	36.4109
M17799.x	rs12133462	A	G	0.5082	-0.0547	0.0124	9.52E-06	19.4595
M17799.x	rs12238450	A	G	0.9559	0.1431	0.0307	3.09E-06	21.7271
M17799.x	rs12327976	A	G	0.9239	0.1079	0.0195	3.28E-08	30.6178
M17799.x	rs12622555	T	C	0.04	-0.1623	0.0299	5.62E-08	29.4642
M17799.x	rs12684749	A	G	0.96	0.1623	0.0299	5.62E-08	29.4642
M17799.x	rs12983955	A	G	0.1002	-0.1301	0.0271	1.6E-06	23.0471
M17799.x	rs13007817	A	G	0.0685	-0.114	0.0218	1.62E-07	27.3462
M17799.x	rs13035089	T	G	0.8393	0.063	0.0143	9.81E-06	19.4093
M17799.x	rs13041562	T	C	0.8472	0.0697	0.0151	3.76E-06	21.3065
M17799.x	rs1353529	T	G	0.3696	-0.0569	0.0127	7.46E-06	20.0732
M17799.x	rs1382668	A	G	0.9273	0.0927	0.0206	7.01E-06	20.25
M17799.x	rs1408273	A	G	0.44	-0.056	0.0127	9.45E-06	19.4432
M17799.x	rs1429342	A	G	0.9623	0.1439	0.0284	4.15E-07	25.6735
M17799.x	rs1462176	T	C	0.0469	-0.139	0.0285	1.07E-06	23.787
M17799.x	rs1498721	T	C	0.953	0.1222	0.0241	3.9E-07	25.7104
M17799.x	rs1523531	T	C	0.2154	-0.0595	0.0132	7.06E-06	20.3182
M17799.x	rs1561803	T	G	0.9623	0.1304	0.028	3.28E-06	21.689
M17799.x	rs1608368	T	C	0.04	-0.1623	0.0299	5.62E-08	29.4642
M17799.x	rs16828389	T	C	0.0405	-0.165	0.0303	5.01E-08	29.654
M17799.x	rs16834234	T	C	0.0372	-0.1418	0.0321	9.92E-06	19.5138
M17799.x	rs16835642	A	G	0.0996	-0.1067	0.0237	6.92E-06	20.269
M17799.x	rs16855172	T	C	0.0406	-0.1636	0.0302	6.12E-08	29.3463
M17799.x	rs16874680	T	C	0.9573	0.1181	0.025	2.38E-06	22.3162
M17799.x	rs16883128	A	G	0.0886	-0.1003	0.0199	4.45E-07	25.4036
M17799.x	rs16947929	A	G	0.0406	-0.1419	0.0282	4.87E-07	25.3202
M17799.x	rs16962386	A	C	0.0799	0.1009	0.0226	8.29E-06	19.9327
M17799.x	rs16986678	A	G	0.0614	-0.1548	0.0224	5.22E-12	47.758
M17799.x	rs17025221	A	T	0.0832	-0.0944	0.0195	1.32E-06	23.4355
M17799.x	rs17069415	A	G	0.0383	-0.1552	0.0286	5.66E-08	29.4477
M17799.x	rs17098098	T	C	0.0738	-0.0961	0.0171	1.93E-08	31.5831
M17799.x	rs17224513	A	G	0.0812	-0.0911	0.0192	2.08E-06	22.513
M17799.x	rs17303404	C	G	0.9617	0.1424	0.0265	7.46E-08	28.8754
M17799.x	rs17488959	T	G	0.0776	-0.1116	0.0218	3.19E-07	26.2069
M17799.x	rs17506661	T	C	0.0465	-0.1171	0.0235	6.22E-07	24.8301
M17799.x	rs17557803	T	C	0.9267	0.0971	0.0213	5.39E-06	20.7816
M17799.x	rs17817565	T	C	0.0397	-0.1686	0.0373	6.09E-06	20.4314
M17799.x	rs1790642	A	G	0.16	-0.0732	0.0165	8.93E-06	19.6813
M17799.x	rs1792131	A	G	0.9141	0.1008	0.0208	1.21E-06	23.4852
M17799.x	rs187090	T	C	0.04	-0.1623	0.0299	5.62E-08	29.4642

M17799.x	rs1896703	T	G	0.0817	-0.1538	0.0226	9.67E-12	46.3122
M17799.x	rs1954097	T	C	0.9146	0.0926	0.0202	4.64E-06	21.0145
M17799.x	rs2041389	C	G	0.05	-0.1506	0.0318	2.25E-06	22.4283
M17799.x	rs2155977	T	C	0.9371	0.0964	0.0195	8.18E-07	24.4391
M17799.x	rs217763	T	C	0.4911	0.0552	0.0108	3.23E-07	26.1235
M17799.x	rs2374150	C	G	0.0418	-0.1422	0.0282	4.55E-07	25.4273
M17799.x	rs2469235	A	G	0.948	0.1221	0.0204	2.09E-09	35.8237
M17799.x	rs2473138	A	G	0.9395	0.1053	0.0218	1.42E-06	23.3316
M17799.x	rs2486578	A	G	0.9524	0.1523	0.0326	2.99E-06	21.8255
M17799.x	rs2493147	T	C	0.0608	-0.0912	0.0184	7.18E-07	24.5671
M17799.x	rs2623167	A	C	0.9047	0.094	0.021	7.57E-06	20.0363
M17799.x	rs268125	C	G	0.9647	0.1492	0.0302	7.71E-07	24.4075
M17799.x	rs268424	A	C	0.0941	-0.1129	0.0237	1.83E-06	22.693
M17799.x	rs276642	A	G	0.9591	0.1626	0.0299	5.62E-08	29.5732
M17799.x	rs2861997	A	G	0.9163	0.0897	0.0187	1.54E-06	23.0092
M17799.x	rs2866604	T	C	0.8329	0.0644	0.0137	2.55E-06	22.0969
M17799.x	rs28951670	T	C	0.0414	-0.1627	0.0299	5.15E-08	29.6096
M17799.x	rs297291	T	C	0.01	0.393	0.088	7.97E-06	19.9443
M17799.x	rs3925748	A	C	0.1468	-0.0826	0.0182	5.55E-06	20.5976
M17799.x	rs4141286	C	G	0.9514	0.1065	0.0231	3.95E-06	21.2557
M17799.x	rs4434498	A	G	0.5074	-0.0554	0.012	4.23E-06	21.3136
M17799.x	rs4454728	A	G	0.0408	-0.1672	0.0307	5.21E-08	29.6617
M17799.x	rs4488147	T	C	0.04	-0.1623	0.0299	5.62E-08	29.4642
M17799.x	rs463183	A	G	0.0833	-0.1164	0.0232	4.98E-07	25.1727
M17799.x	rs4733038	A	C	0.0208	-0.1718	0.0387	9.03E-06	19.7072
M17799.x	rs4768766	A	C	0.0417	-0.1376	0.0243	1.58E-08	32.0645
M17799.x	rs4849717	A	T	0.9643	0.1359	0.0268	4.04E-07	25.714
M17799.x	rs4875362	T	G	0.0309	-0.1646	0.0355	3.43E-06	21.4982
M17799.x	rs4905451	T	C	0.0399	-0.1551	0.0279	2.57E-08	30.904
M17799.x	rs5809847	T	C	0.9106	0.1145	0.025	4.6E-06	20.9764
M17799.x	rs600711	A	G	0.9334	0.1004	0.0179	2.08E-08	31.4602
M17799.x	rs6070814	T	C	0.1104	-0.0813	0.0165	7.88E-07	24.278
M17799.x	rs6506569	T	C	0.5349	0.0541	0.0105	2.4E-07	26.547
M17799.x	rs6559174	A	C	0.9569	0.1289	0.0242	9.59E-08	28.371
M17799.x	rs665540	T	G	0.118	-0.1368	0.0266	2.77E-07	26.449
M17799.x	rs6783548	T	C	0.6219	-0.0779	0.0162	1.62E-06	23.123
M17799.x	rs6801674	T	C	0.595	0.0568	0.012	2.16E-06	22.4044
M17799.x	rs7069613	T	C	0.9576	0.1271	0.0262	1.18E-06	23.5336
M17799.x	rs7144659	A	G	0.8457	0.0554	0.0113	9.86E-07	24.036
M17799.x	rs7423047	T	C	0.0375	-0.1451	0.0268	6.31E-08	29.3133
M17799.x	rs7454011	T	G	0.0448	-0.1313	0.0235	2.34E-08	31.2172
M17799.x	rs7616688	A	G	0.0408	-0.163	0.03	5.5E-08	29.5211
M17799.x	rs7688643	T	C	0.9032	0.083	0.0186	8.23E-06	19.9127
M17799.x	rs7692251	A	G	0.9573	0.133	0.028	1.97E-06	22.5625
M17799.x	rs7701924	A	G	0.0923	-0.0907	0.0191	2.08E-06	22.5501
M17799.x	rs7708256	T	C	0.0274	-0.1541	0.0348	9.6E-06	19.6086
M17799.x	rs771788	A	G	0.0921	-0.0881	0.0187	2.52E-06	22.1957
M17799.x	rs7751252	T	C	0.9491	0.105	0.0227	3.9E-06	21.3957
M17799.x	rs7755387	T	G	0.0397	-0.1252	0.0259	1.3E-06	23.3673
M17799.x	rs7785951	T	G	0.0389	-0.1396	0.0267	1.67E-07	27.3368
M17799.x	rs7969423	A	G	0.0833	-0.1138	0.0237	1.56E-06	23.0562
M17799.x	rs8051340	C	G	0.9164	0.104	0.0207	5.01E-07	25.2421
M17799.x	rs8101619	T	C	0.5013	-0.0618	0.0131	2.28E-06	22.2553
M17799.x	rs8118890	A	G	0.9185	0.1147	0.0235	1.06E-06	23.8227
M17799.x	rs831541	T	C	0.2066	-0.0567	0.0116	9.87E-07	23.8919

M17799.x	rs836533	A	T	0.96	0.1623	0.0299	5.62E-08	29.4642
M17799.x	rs845861	T	C	0.2713	-0.064	0.0143	7.96E-06	20.0303
M17799.x	rs886236	C	G	0.7262	0.0749	0.0167	7.71E-06	20.1155
M17799.x	rs928907	T	C	0.0952	-0.0749	0.0166	6.04E-06	20.3586
M17799.x	rs9341191	T	C	0.0476	-0.1636	0.0301	5.68E-08	29.5416
M17799.x	rs945493	A	G	0.0433	-0.1625	0.0301	6.45E-08	29.1456
M17799.x	rs9497904	A	G	0.4345	-0.0543	0.012	6.36E-06	20.4756
M17799.x	rs9557045	A	T	0.087	-0.1164	0.0234	6.28E-07	24.7442
M17799.x	rs9861255	A	G	0.92	0.1156	0.0231	5.74E-07	25.0433
M17799.x	rs997892	A	C	0.9349	0.0925	0.0181	3.14E-07	26.1172
M17799.x	rs9999552	A	G	0.9321	0.1464	0.0213	5.7E-12	47.2414
M17805.m	rs1027175	A	G	0.8735	0.0221	0.005	9.56E-06	19.5364
M17805.m	rs1348656	T	C	0.798	-0.0202	0.0042	1.52E-06	23.1315
M17805.m	rs1477025	T	C	0.6547	0.0164	0.0034	2.01E-06	23.2664
M17805.m	rs16839086	A	C	0.2302	0.0329	0.0074	8.81E-06	19.7664
M17805.m	rs1709851	A	C	0.6588	-0.0155	0.0035	7.62E-06	19.6122
M17805.m	rs3793900	A	C	0.6359	-0.0154	0.0034	7.41E-06	20.5156
M17805.m	rs6557244	T	C	0.9101	0.0302	0.0067	7.32E-06	20.3172
M17805.m	rs6738713	T	C	0.9295	-0.0308	0.0066	3.38E-06	21.7778
M17805.m	rs9560579	A	G	0.5193	-0.0153	0.0034	6.41E-06	20.25
M17807.m	rs13222543	T	C	0.0194	-0.0941	0.021	7.57E-06	20.0789
M17807.m	rs1390947	A	C	0.4471	-0.0272	0.005	5.72E-08	29.5936
M17807.m	rs1398802	T	G	0.6877	-0.026	0.0057	6.12E-06	20.8064
M17807.m	rs16934512	A	C	0.5653	-0.0233	0.0052	7.36E-06	20.0773
M17807.m	rs17091567	T	C	0.1249	0.0332	0.0074	7.53E-06	20.1286
M17807.m	rs17289436	A	G	0.5235	-0.0248	0.0056	9.63E-06	19.6122
M17807.m	rs1864468	A	G	0.6161	-0.0251	0.005	5.94E-07	25.2004
M17807.m	rs2048428	T	C	0.133	-0.036	0.0081	9.52E-06	19.7531
M17807.m	rs2199680	A	T	0.8328	-0.0415	0.0065	2.17E-10	40.7633
M17807.m	rs2847326	A	T	0.2376	0.0259	0.0058	9.28E-06	19.9408
M17807.m	rs296381	T	C	0.8282	0.039	0.0067	4.83E-09	33.8828
M17807.m	rs4149452	T	C	0.2609	0.029	0.0058	7.2E-07	25
M17807.m	rs4393440	T	C	0.987	-0.129	0.028	4.08E-06	21.2258
M17807.m	rs450940	T	C	0.5116	-0.0251	0.005	5.04E-07	25.2004
M17807.m	rs480891	A	G	0.6432	-0.0235	0.0051	4.15E-06	21.2322
M17807.m	rs6603803	A	G	0.4879	-0.0253	0.0049	2.93E-07	26.6593
M17807.m	rs6900835	T	C	0.2163	-0.0284	0.0059	1.44E-06	23.1704
M17807.m	rs7610725	A	G	0.9698	-0.1714	0.0373	4.42E-06	21.1156
M17807.m	rs878983	T	C	0.2282	-0.0269	0.006	8.15E-06	20.1003
M17807.m	rs895569	T	C	0.458	0.0221	0.005	8.67E-06	19.5364
M17807.m	rs932412	A	C	0.3987	-0.0227	0.005	5.84E-06	20.6116
M17807.m	rs9552597	A	G	0.1845	0.0306	0.0065	2.15E-06	22.1624
M17807.m	rs9632722	A	G	0.9087	0.0571	0.0085	1.52E-11	45.1268
M17807.m	rs9972424	A	G	0.6602	-0.0242	0.0051	2.04E-06	22.516
M17945.m	rs10443953	A	G	0.382	0.0086	0.0018	2.6E-06	22.8272
M17945.m	rs10841406	A	G	0.3963	-0.0087	0.0018	2.27E-06	23.3611
M17945.m	rs10892951	A	G	0.4059	-0.0088	0.0018	1.66E-06	23.9012
M17945.m	rs11150606	T	C	0.989	-0.072	0.014	2.71E-07	26.449
M17945.m	rs11210537	A	G	0.3117	0.0085	0.0019	4.96E-06	20.0139
M17945.m	rs11263804	C	G	0.2495	0.0122	0.0026	2.76E-06	22.0178
M17945.m	rs12208243	A	G	0.9516	0.0236	0.0053	8.25E-06	19.8277
M17945.m	rs12536678	C	G	0.3198	-0.0096	0.0019	2.04E-07	25.5291
M17945.m	rs12639715	T	C	0.6649	0.0085	0.0019	4.18E-06	20.0139
M17945.m	rs12975371	A	G	0.5367	-0.0081	0.0018	9.15E-06	20.25
M17945.m	rs13238296	T	C	0.6533	0.0083	0.0019	8.38E-06	19.0831

M17945.m	rs16909189	T	C	0.0502	0.0217	0.0045	1.39E-06	23.2538
M17945.m	rs1799977	A	G	0.6748	0.0086	0.0019	3.91E-06	20.4875
M17945.m	rs2272588	A	G	0.5093	0.0085	0.0018	4.18E-06	22.2994
M17945.m	rs2555231	T	C	0.6247	-0.0094	0.0018	3.22E-07	27.2716
M17945.m	rs2589314	A	G	0.5476	-0.0083	0.0018	6.42E-06	21.2623
M17945.m	rs2745001	A	G	0.3976	-0.0081	0.0018	9.25E-06	20.25
M17945.m	rs2946398	T	C	0.3787	-0.0083	0.0018	7.64E-06	21.2623
M17945.m	rs3796622	T	C	0.3747	-0.0089	0.0019	1.35E-06	21.9418
M17945.m	rs3822742	A	C	0.363	-0.0098	0.002	8.48E-07	24.01
M17945.m	rs6033014	T	C	0.3688	-0.0087	0.0018	2.6E-06	23.3611
M17945.m	rs6035729	T	C	0.6886	0.0083	0.0019	7E-06	19.0831
M17945.m	rs629487	T	G	0.5697	-0.0087	0.0018	2.21E-06	23.3611
M17945.m	rs6462013	A	G	0.5116	-0.0082	0.0018	7.14E-06	20.7531
M17945.m	rs6564154	T	C	0.6689	0.0096	0.0019	2.57E-07	25.5291
M17945.m	rs6905811	T	C	0.343	0.009	0.0018	1.01E-06	25
M17945.m	rs697635	A	G	0.3292	0.0084	0.0019	5.15E-06	19.5457
M17945.m	rs7192844	T	C	0.0322	-0.0274	0.006	5.21E-06	20.8544
M17945.m	rs7283743	T	C	0.1245	-0.0128	0.0028	5.01E-06	20.898
M17945.m	rs7322113	T	C	0.9889	-0.0453	0.0098	3.65E-06	21.367
M17945.m	rs757202	T	C	0.5096	-0.009	0.0018	8.35E-07	25
M17945.m	rs7652277	T	G	0.4645	0.0082	0.0018	8E-06	20.7531
M17945.m	rs913416	T	C	0.5313	-0.0082	0.0018	7.52E-06	20.7531
M17945.m	rs9302365	A	C	0.4684	-0.0096	0.0019	2.18E-07	25.5291
M17945.m	rs9408014	A	T	0.8976	-0.0158	0.0034	4.1E-06	21.5952
M17945.m	rs9715724	T	C	0.448	0.009	0.002	6.8E-06	20.25
M18254.x	rs10791723	A	T	0.3242	-0.0658	0.0141	2.96E-06	21.7778
M18254.x	rs11915650	A	G	0.1557	-0.0474	0.01	2.22E-06	22.4676
M18254.x	rs12341812	T	C	0.9564	-0.093	0.0188	7.44E-07	24.4709
M18254.x	rs12895893	A	G	0.8278	-0.0443	0.0093	1.96E-06	22.6904
M18254.x	rs13387835	A	T	0.9417	-0.0622	0.0138	6.24E-06	20.3153
M18254.x	rs17777937	T	C	0.0548	-0.0744	0.0165	6.2E-06	20.3319
M18254.x	rs2059238	A	C	0.1958	-0.0435	0.0087	6.6E-07	25
M18254.x	rs29456	T	C	0.2189	-0.0356	0.0077	4.1E-06	21.3756
M18254.x	rs3768372	C	G	0.8499	-0.0808	0.0182	8.72E-06	19.7097
M18254.x	rs3846408	C	G	0.6348	0.0303	0.0068	9.48E-06	19.8549
M18254.x	rs4661452	T	C	0.9194	-0.0577	0.0127	5.18E-06	20.6416
M18254.x	rs7558224	T	C	0.7832	0.0376	0.0084	8.56E-06	20.0363
M18254.x	rs946712	A	C	0.9563	0.084	0.0165	3.29E-07	25.9174
M18281.x	rs10886877	T	C	0.9072	-0.1704	0.0356	1.75E-06	22.9107
M18281.x	rs11824477	T	C	0.5787	0.0951	0.0215	9.59E-06	19.5652
M18281.x	rs1484976	T	C	0.9823	-0.3427	0.0774	9.53E-06	19.6041
M18281.x	rs17021783	A	G	0.9857	-0.3999	0.0838	1.84E-06	22.7727
M18281.x	rs17682068	A	G	0.9239	-0.1836	0.0349	1.45E-07	27.6754
M18281.x	rs1951656	T	C	0.986	-0.42	0.0887	2.2E-06	22.4208
M18281.x	rs219633	A	G	0.121	0.1483	0.0297	5.96E-07	24.9327
M18281.x	rs37058	A	T	0.0686	0.1894	0.0416	5.25E-06	20.7288
M18281.x	rs3800052	T	C	0.0846	0.1715	0.0377	5.46E-06	20.6941
M18281.x	rs7016707	T	G	0.099	0.1439	0.0317	5.81E-06	20.6064
M18281.x	rs735507	A	G	0.8134	-0.1128	0.0246	4.6E-06	21.0256
M18281.x	rs7663798	A	G	0.0486	0.1966	0.0438	7.23E-06	20.1474
M18281.x	rs919028	A	T	0.9514	-0.5485	0.1233	8.69E-06	19.7891
M18283.m	rs12340032	T	G	0.4693	-0.0273	0.006	5.59E-06	20.7025
M18283.m	rs12764831	A	G	0.6005	0.0253	0.0055	4.46E-06	21.16
M18283.m	rs1321208	T	C	0.1913	-0.0355	0.0073	1.05E-06	23.6489
M18283.m	rs1392786	T	C	0.4017	-0.0282	0.0059	1.93E-06	22.8452

M18283.m	rs16865409	A	T	0.7722	-0.0385	0.0074	1.78E-07	27.0681
M18283.m	rs2035344	A	G	0.6893	0.0254	0.0056	6.74E-06	20.5727
M18283.m	rs4708792	A	G	0.1015	-0.0673	0.0143	2.61E-06	22.1492
M18283.m	rs6725791	T	C	0.4058	0.0259	0.0054	1.81E-06	23.0045
M18283.m	rs9565748	T	G	0.6241	0.0246	0.0055	6.97E-06	20.0053
M18283.m	rs9854875	A	G	0.3185	0.0266	0.0056	2.05E-06	22.5625
M18335.x	rs17209311	C	G	0.1068	0.0723	0.0157	4.17E-06	21.2069
M18335.x	rs2726698	C	G	0.6936	-0.0791	0.0152	2.12E-07	27.0811
M18335.x	rs521504	T	C	0.0327	0.1015	0.0229	9.54E-06	19.6454
M18335.x	rs6955908	T	C	0.4325	0.0386	0.0083	3.5E-06	21.6281
M18335.x	rs6999144	T	C	0.915	0.0668	0.015	8.46E-06	19.8322
M18335.x	rs7088140	T	G	0.5864	0.0353	0.0079	7.26E-06	19.9662
M18349.m	rs10159475	A	G	0.8276	0.0157	0.0034	4.09E-06	21.3227
M18349.m	rs1055689	A	G	0.2969	-0.0135	0.0027	4.26E-07	25
M18349.m	rs11623441	T	C	0.6919	0.0121	0.0027	7.84E-06	20.0837
M18349.m	rs11647808	A	C	0.0735	0.0249	0.0051	8.57E-07	23.8374
M18349.m	rs12526522	T	C	0.0349	-0.0369	0.0078	2.55E-06	22.3802
M18349.m	rs12880087	A	C	0.1577	-0.0192	0.0043	9.9E-06	19.9373
M18349.m	rs15676	A	G	0.7123	0.0191	0.0027	1.33E-12	50.0425
M18349.m	rs17073667	A	G	0.023	0.0856	0.018	1.84E-06	22.6153
M18349.m	rs2511986	C	G	0.7684	0.0153	0.0034	7.63E-06	20.25
M18349.m	rs2689672	T	G	0.7286	0.0119	0.0027	9.5E-06	19.4252
M18349.m	rs2932525	A	G	0.8432	-0.0157	0.0035	7.55E-06	20.1216
M18349.m	rs314707	A	G	0.8305	-0.0159	0.0035	4.43E-06	20.6376
M18349.m	rs4719964	A	G	0.2554	-0.0123	0.0027	4.46E-06	20.7531
M18349.m	rs6910570	A	G	0.5538	0.0125	0.0026	1.84E-06	23.1139
M18349.m	rs7503709	T	C	0.4574	-0.0129	0.0028	3.3E-06	21.2258
M18349.m	rs7727858	A	G	0.8891	-0.0205	0.0042	1.08E-06	23.8237
M18349.m	rs7805747	A	G	0.2679	0.0131	0.0028	3.77E-06	21.889
M18349.m	rs940008	A	G	0.4416	0.0251	0.0054	2.64E-06	21.6053
M18357.m	rs17092360	A	G	0.9096	-0.0671	0.0143	2.61E-06	22.0178
M18357.m	rs17498524	T	C	0.7903	0.0422	0.0095	9.65E-06	19.7323
M18357.m	rs1772858	A	G	0.6211	-0.0341	0.0076	7.98E-06	20.1318
M18357.m	rs3846242	T	C	0.5792	0.0383	0.0086	8.04E-06	19.8336
M18357.m	rs4905425	A	C	0.5066	0.0377	0.0075	5.72E-07	25.2674
M18357.m	rs7349940	A	T	0.1269	-0.0611	0.0132	3.59E-06	21.4257
M18357.m	rs7697449	T	C	0.5636	0.0383	0.0085	6.18E-06	20.303
M18369.m	rs10426971	A	G	0.4387	0.0076	0.0017	8.18E-06	19.9862
M18369.m	rs11151740	A	G	0.839	-0.0115	0.0026	9.28E-06	19.5636
M18369.m	rs11866781	T	C	0.4199	0.0075	0.0017	9.43E-06	19.4637
M18369.m	rs12707434	A	G	0.9639	-0.0501	0.0101	7.66E-07	24.6055
M18369.m	rs1292666	T	C	0.4654	0.0079	0.0017	2.74E-06	21.5952
M18369.m	rs13188217	A	C	0.4184	0.0081	0.0017	1.77E-06	22.7024
M18369.m	rs13189579	A	G	0.9299	-0.0192	0.0043	7.06E-06	19.9373
M18369.m	rs1382190	A	G	0.9891	-0.0401	0.0091	9.32E-06	19.4181
M18369.m	rs17185878	A	G	0.8515	-0.0122	0.0025	8.68E-07	23.8144
M18369.m	rs17652614	A	G	0.5436	-0.0078	0.0017	7.29E-06	21.0519
M18369.m	rs17683450	A	T	0.8525	0.0112	0.0024	4.14E-06	21.7778
M18369.m	rs17794361	A	C	0.847	0.024	0.005	1.59E-06	23.04
M18369.m	rs200449	A	G	0.569	0.009	0.002	6.8E-06	20.25
M18369.m	rs2043769	T	C	0.8032	-0.0111	0.0025	8.72E-06	19.7136
M18369.m	rs2201453	A	G	0.438	0.0082	0.0017	1.14E-06	23.2664
M18369.m	rs2203995	A	G	0.6055	0.0077	0.0017	7.26E-06	20.5156
M18369.m	rs2312933	T	C	0.9259	-0.015	0.0034	8.49E-06	19.4637
M18369.m	rs2413113	T	C	0.4667	-0.0076	0.0017	5.74E-06	19.9862

M18369.m	rs2573124	A	C	0.4579	-0.0082	0.0017	1.13E-06	23.2664
M18369.m	rs2819322	T	C	0.4589	-0.009	0.0017	1.5E-07	28.0277
M18369.m	rs3747115	T	C	0.4954	0.0079	0.0017	2.32E-06	21.5952
M18369.m	rs37959	T	C	0.3919	0.0077	0.0017	8.94E-06	20.5156
M18369.m	rs3799105	A	C	0.5951	0.0076	0.0017	6.08E-06	19.9862
M18369.m	rs3846286	A	G	0.9275	-0.0307	0.0068	6.64E-06	20.3826
M18369.m	rs4767788	A	G	0.4177	0.0082	0.0018	5.21E-06	20.7531
M18369.m	rs4926707	A	G	0.4735	-0.0076	0.0017	6.22E-06	19.9862
M18369.m	rs6470195	A	T	0.89	0.019	0.004	2.03E-06	22.5625
M18369.m	rs6768610	T	C	0.914	0.0144	0.0032	7.08E-06	20.25
M18369.m	rs6797094	T	G	0.611	0.009	0.002	6.8E-06	20.25
M18369.m	rs6820313	T	G	0.5183	0.0089	0.0017	1.08E-07	27.4083
M18369.m	rs6833720	A	G	0.4772	-0.0084	0.0017	6.09E-07	24.4152
M18369.m	rs6951006	A	G	0.4884	-0.008	0.0017	3.53E-06	22.1453
M18369.m	rs715	T	C	0.7093	0.011	0.0023	1.3E-06	22.8733
M18369.m	rs7574847	A	G	0.1481	-0.0118	0.0026	3.98E-06	20.5976
M18369.m	rs7678928	T	C	0.4678	0.0076	0.0017	5.49E-06	19.9862
M18369.m	rs7830772	A	C	0.9671	-0.0399	0.0085	2.95E-06	22.0347
M18369.m	rs9827101	A	C	0.5393	0.008	0.0017	2.99E-06	22.1453
M18392.x	rs2038607	A	G	0.7821	-0.037	0.0079	2.42E-06	21.9356
M18392.x	rs2081811	A	G	0.7543	0.0327	0.0072	5.81E-06	20.6267
M18392.x	rs3846406	A	C	0.4896	0.0299	0.0062	1.46E-06	23.2573
M18392.x	rs4410790	T	C	0.3695	0.0313	0.0064	8.96E-07	23.9182
M18392.x	rs9349565	A	T	0.088	0.0478	0.0108	9.44E-06	19.5888
M18394.x	rs10899433	T	C	0.4203	-0.0236	0.0053	9.17E-06	19.8277
M18394.x	rs11660053	T	C	0.8153	-0.0326	0.007	3.68E-06	21.689
M18394.x	rs11734001	T	C	0.5271	-0.0256	0.0054	1.77E-06	22.4746
M18394.x	rs12895893	A	G	0.8282	-0.0375	0.0078	1.49E-06	23.1139
M18394.x	rs1397829	A	G	0.535	0.0268	0.0053	4.53E-07	25.5692
M18394.x	rs17678706	A	G	0.1871	0.0511	0.0114	7.88E-06	20.0924
M18394.x	rs2059238	A	C	0.1965	-0.0328	0.0071	4.31E-06	21.3418
M18394.x	rs2472297	T	C	0.238	-0.0346	0.0071	9.64E-07	23.7485
M18394.x	rs2564483	T	G	0.6043	0.024	0.0053	6.42E-06	20.5055
M18394.x	rs27667	T	G	0.6338	-0.0564	0.012	2.65E-06	22.09
M18394.x	rs6779362	T	C	0.7633	-0.0288	0.0063	5.74E-06	20.898
M18394.x	rs755677	T	G	0.87	0.0754	0.0164	4.51E-06	21.1376
M18394.x	rs7927424	A	G	0.0147	-0.115	0.0251	4.79E-06	20.9917
M18394.x	rs8096497	A	G	0.097	0.053	0.0101	1.46E-07	27.5365
M18394.x	rs915529	A	G	0.0187	-0.1861	0.0405	4.23E-06	21.1146
M18467.m	rs12583004	T	C	0.0723	-0.0318	0.0067	1.75E-06	22.5271
M18467.m	rs16859272	A	G	0.0503	-0.0637	0.0143	9.09E-06	19.843
M18467.m	rs174556	T	C	0.299	-0.0356	0.0037	1.97E-22	92.5756
M18467.m	rs17736501	C	G	0.0125	-0.0684	0.0153	7.68E-06	19.9862
M18467.m	rs179976	T	C	0.7581	-0.0198	0.0043	3.92E-06	21.2028
M18467.m	rs2068592	A	G	0.0144	0.071	0.0152	2.96E-06	21.8187
M18467.m	rs2290140	A	G	0.3486	-0.0165	0.0036	5.8E-06	21.0069
M18467.m	rs2364754	A	G	0.0751	-0.032	0.0069	3.69E-06	21.5081
M18467.m	rs6468384	A	C	0.5691	0.0179	0.0036	5.63E-07	24.723
M18467.m	rs7005406	T	C	0.8036	0.0353	0.0077	5.29E-06	21.0169
M18467.m	rs703811	T	C	0.7585	0.017	0.0037	4.2E-06	21.1103
M18467.m	rs8107558	T	C	0.9541	0.0665	0.0146	5.52E-06	20.7462
M18474.m	rs1026775	T	C	0.4495	-0.0591	0.0119	6.4E-07	24.665
M18474.m	rs10515456	A	G	0.1032	0.0868	0.0194	7.98E-06	20.0187
M18474.m	rs11736691	A	C	0.9602	-0.24	0.0491	1.04E-06	23.8924
M18474.m	rs11854497	A	G	0.0972	0.0869	0.0192	6.16E-06	20.4851

M18474.m	rs12483707	C	G	0.3543	0.0849	0.0192	9.19E-06	19.553
M18474.m	rs12526152	A	C	0.0139	0.4507	0.0939	1.61E-06	23.038
M18474.m	rs12905081	A	G	0.4166	-0.0574	0.0122	2.58E-06	22.1363
M18474.m	rs1599081	T	G	0.788	-0.0691	0.0145	1.9E-06	22.7102
M18474.m	rs16904525	T	C	0.0297	0.1461	0.033	9.4E-06	19.6007
M18474.m	rs2912546	T	C	0.0501	0.2679	0.0469	1.15E-08	32.6287
M18474.m	rs4877694	T	C	0.301	0.0579	0.013	8.74E-06	19.8367
M18474.m	rs7279572	T	C	0.9644	-0.2727	0.0585	3.09E-06	21.7299
M18474.m	rs7546527	A	G	0.012	0.4654	0.0991	2.64E-06	22.0549
M18476.m	rs10738265	T	C	0.1746	0.0354	0.0073	1.13E-06	23.5159
M18476.m	rs12590118	T	G	0.9201	-0.0463	0.01	3.84E-06	21.4369
M18476.m	rs17107026	T	C	0.9608	-0.0781	0.016	1.08E-06	23.8266
M18476.m	rs17807835	A	G	0.8027	0.0343	0.0072	1.71E-06	22.6946
M18476.m	rs2718107	A	C	0.5408	0.0257	0.0057	5.85E-06	20.329
M18476.m	rs3807360	A	G	0.2011	0.0735	0.0164	7.44E-06	20.0857
M18476.m	rs7837471	A	G	0.015	0.3403	0.0731	3.27E-06	21.6715
M18476.m	rs795543	T	C	0.4091	-0.025	0.0055	5.19E-06	20.6612
M18476.m	rs9914239	A	G	0.9123	-0.1056	0.0228	3.64E-06	21.4515
M18477.m	rs17746543	A	G	0.5073	-0.0883	0.0191	3.67E-06	21.3725
M18477.m	rs2420201	A	G	0.0494	0.1473	0.0309	1.9E-06	22.7242
M18477.m	rs4338010	T	C	0.0788	-0.1084	0.0239	5.73E-06	20.5713
M18477.m	rs6585252	C	G	0.9571	-0.1538	0.0312	8.55E-07	24.2998
M18477.m	rs7856830	T	C	0.1533	-0.0891	0.0188	2.27E-06	22.4615
M18477.m	rs806784	T	C	0.7156	-0.0952	0.0198	1.48E-06	23.1176
M18477.m	rs9301881	T	C	0.5692	0.0593	0.0131	5.79E-06	20.4912
M18494.m	rs11121663	T	C	0.7415	0.0308	0.0067	3.95E-06	21.1325
M18494.m	rs12104496	A	T	0.1572	-0.0395	0.0084	2.7E-06	22.1124
M18494.m	rs1608165	C	G	0.1352	-0.0406	0.0092	9.61E-06	19.475
M18494.m	rs16950349	A	G	0.9243	-0.0507	0.0112	6.36E-06	20.4918
M18494.m	rs1851722	A	G	0.2338	0.0354	0.0075	2.01E-06	22.2784
M18494.m	rs2992907	T	C	0.6664	0.0296	0.0065	6.16E-06	20.7375
M18494.m	rs392488	T	C	0.1984	-0.0337	0.0075	7.26E-06	20.19
M18494.m	rs4896923	A	G	0.5134	0.0361	0.0072	5.31E-07	25.1391
M18494.m	rs6465368	A	G	0.0343	0.185	0.0416	8.51E-06	19.7768
M18494.m	rs6858711	T	C	0.8875	-0.1096	0.0246	8.28E-06	19.8496
M18494.m	rs7432211	T	C	0.5934	0.0294	0.0064	4.93E-06	21.1025
M18494.m	rs7568568	T	C	0.9251	0.1481	0.0306	1.31E-06	23.4243
M18494.m	rs7848609	T	C	0.0168	0.1108	0.0244	5.69E-06	20.6205
M18497.m	rs10514476	A	G	0.4619	0.0391	0.0082	1.8E-06	22.7366
M18497.m	rs10766562	T	C	0.4367	0.0389	0.0082	1.97E-06	22.5046
M18497.m	rs10813358	A	G	0.4043	0.0373	0.0082	5.62E-06	20.6914
M18497.m	rs12070179	T	C	0.951	-0.2041	0.0432	2.31E-06	22.3213
M18497.m	rs12984452	T	C	0.3058	-0.0413	0.009	4.71E-06	21.0579
M18497.m	rs16955093	T	C	0.015	0.3552	0.0782	5.61E-06	20.6316
M18497.m	rs17169191	A	T	0.9347	-0.1825	0.0405	6.61E-06	20.3056
M18497.m	rs1948095	A	C	0.8599	-0.1423	0.0286	6.39E-07	24.7558
M18497.m	rs226212	A	G	0.5696	-0.0384	0.0084	4.84E-06	20.898
M18497.m	rs309873	C	G	0.065	0.0743	0.0164	5.63E-06	20.5253
M18497.m	rs7108724	A	G	0.6246	-0.0389	0.0082	2.13E-06	22.5046
M18497.m	rs7139744	T	G	0.3014	0.0423	0.0092	3.87E-06	21.14
M18497.m	rs7171216	T	G	0.4139	-0.0442	0.0096	4.41E-06	21.1984
M18497.m	rs7809441	A	G	0.0807	0.0748	0.0147	3.8E-07	25.8922
M18497.m	rs9815713	A	G	0.9315	-0.0917	0.0192	1.79E-06	22.8106
M18497.m	rs9962219	T	G	0.99	-0.4251	0.0954	8.28E-06	19.8557
M18929.m	rs11061544	A	G	0.95	-0.0241	0.0051	2.11E-06	22.3303

M18929.m	rs12803289	A	G	0.2119	-0.0121	0.0026	4.31E-06	21.6583
M18929.m	rs16828708	A	G	0.7407	0.0135	0.0027	9.08E-07	25
M18929.m	rs1799405	A	T	0.9643	0.032	0.0061	1.81E-07	27.5195
M18929.m	rs2028973	T	C	0.1086	0.0162	0.0034	2.66E-06	22.7024
M18929.m	rs225998	A	C	0.7547	-0.0114	0.0026	9.01E-06	19.2249
M18929.m	rs2363698	A	T	0.7857	0.0118	0.0026	5.19E-06	20.5976
M18929.m	rs2806302	T	G	0.688	0.0125	0.0025	6.71E-07	25
M18929.m	rs4904262	T	C	0.1727	-0.0121	0.0026	4.91E-06	21.6583
M18929.m	rs6871105	A	G	0.2423	-0.012	0.0026	4.22E-06	21.3018
M18929.m	rs746764	T	C	0.1676	-0.0236	0.0052	6.63E-06	20.5976
M18929.m	rs7607746	T	C	0.1582	-0.0333	0.0062	8.35E-08	28.8473
M18929.m	rs9500850	A	G	0.9644	0.0555	0.0124	8.12E-06	20.0328
M19323.m	rs10063514	T	C	0.0701	0.0474	0.0106	8.19E-06	19.9961
M19323.m	rs10788823	T	G	0.1884	0.0369	0.0081	5.96E-06	20.7531
M19323.m	rs12583004	T	C	0.0716	-0.0288	0.0064	5.76E-06	20.25
M19323.m	rs1808751	A	G	0.7375	0.0164	0.0035	3.58E-06	21.9559
M19323.m	rs728634	T	C	0.0303	0.0688	0.0154	7.44E-06	19.9588
M19323.m	rs913342	C	G	0.1392	0.0229	0.0051	8.19E-06	20.1619
M19324.m	rs10491073	T	C	0.0175	0.122	0.0233	1.66E-07	27.4162
M19324.m	rs10501089	T	C	0.0309	0.0836	0.0176	2.1E-06	22.5625
M19324.m	rs11844504	C	G	0.9853	-0.1381	0.0277	6.28E-07	24.8558
M19324.m	rs11872932	A	T	0.1999	0.0202	0.0045	6.61E-06	20.1501
M19324.m	rs1925409	T	C	0.3294	0.0294	0.0066	8.32E-06	19.843
M19324.m	rs2241216	C	G	0.199	0.0252	0.0053	2.32E-06	22.6073
M19324.m	rs8107558	T	C	0.9545	0.0652	0.0146	8.38E-06	19.943
M19362.m	rs11119860	T	C	0.4341	-0.0079	0.0017	5.45E-06	21.5952
M19362.m	rs11854705	A	G	0.4635	-0.0079	0.0017	4.57E-06	21.5952
M19362.m	rs12119789	A	G	0.4048	-0.0082	0.0017	2.38E-06	23.2664
M19362.m	rs12298661	C	G	0.9	-0.015	0.0032	2.94E-06	21.9727
M19362.m	rs12473913	A	C	0.4341	-0.0084	0.0017	1.13E-06	24.4152
M19362.m	rs12596849	A	G	0.1846	-0.0113	0.0025	3.93E-06	20.4304
M19362.m	rs12703397	T	C	0.9778	0.0314	0.0067	3E-06	21.9639
M19362.m	rs1382606	A	G	0.5044	0.008	0.0017	3.11E-06	22.1453
M19362.m	rs1460542	A	C	0.1671	0.0117	0.0025	2.93E-06	21.9024
M19362.m	rs1602068	T	C	0.5655	-0.0079	0.0017	5.67E-06	21.5952
M19362.m	rs1655155	T	C	0.5977	-0.009	0.0017	1.51E-07	28.0277
M19362.m	rs16864718	A	T	0.0172	0.034	0.0075	6.76E-06	20.5511
M19362.m	rs17096124	A	G	0.9614	0.0435	0.0082	1.06E-07	28.1417
M19362.m	rs2041343	T	C	0.4795	0.0113	0.0023	6.67E-07	24.138
M19362.m	rs2389336	A	T	0.023	-0.038	0.008	2.03E-06	22.5625
M19362.m	rs3844066	T	C	0.8135	0.0113	0.0025	5.67E-06	20.4304
M19362.m	rs4337809	T	C	0.45	-0.0084	0.0017	1.22E-06	24.4152
M19362.m	rs4569257	A	G	0.4136	-0.0111	0.0024	2.62E-06	21.3906
M19362.m	rs5746800	A	G	0.5003	0.0083	0.0018	2.44E-06	21.2623
M19362.m	rs650455	A	G	0.0418	-0.0214	0.0048	9.67E-06	19.8767
M19362.m	rs7118352	A	G	0.8182	-0.0112	0.0025	5.34E-06	20.0704
M19362.m	rs7680308	A	G	0.4854	0.0077	0.0017	9.08E-06	20.5156
M19362.m	rs7802565	C	G	0.024	-0.036	0.008	6.8E-06	20.25
M19364.m	rs10211524	A	G	0.4195	-0.014	0.0026	5.39E-08	28.9941
M19364.m	rs1021452	T	C	0.642	0.014	0.0026	7.83E-08	28.9941
M19364.m	rs12100123	A	G	0.0425	-0.0295	0.0065	5.23E-06	20.5976
M19364.m	rs1260326	T	C	0.4066	0.0122	0.0026	1.71E-06	22.0178
M19364.m	rs1261411	T	C	0.4052	0.0119	0.0026	3.29E-06	20.9482
M19364.m	rs13336260	A	C	0.828	-0.0226	0.0045	5.91E-07	25.2227
M19364.m	rs1758386	A	G	0.0199	-0.0782	0.0172	5.72E-06	20.6708

M19364.m	rs2151539	T	C	0.7279	-0.0122	0.0027	4.57E-06	20.417
M19364.m	rs2846556	T	G	0.3337	-0.0135	0.0026	2.03E-07	26.9601
M19364.m	rs4128864	T	C	0.0303	-0.0405	0.0089	5E-06	20.7076
M19364.m	rs4280872	A	G	0.0491	-0.0269	0.0059	5.48E-06	20.7874
M19364.m	rs4332039	A	G	0.3062	-0.0153	0.0033	3.34E-06	21.4959
M19364.m	rs4472601	A	G	0.3066	-0.0122	0.0028	9.84E-06	18.9847
M19364.m	rs505125	C	G	0.7328	0.0133	0.0026	5.22E-07	26.1672
M19364.m	rs6053126	A	G	0.253	-0.022	0.004	3.8E-08	30.25
M19364.m	rs6579773	T	C	0.933	-0.0472	0.0105	6.47E-06	20.2072
M19364.m	rs7037931	A	G	0.3274	-0.0115	0.0026	9.44E-06	19.5636
M19364.m	rs7415896	A	G	0.0488	0.0304	0.0062	8.77E-07	24.0416
M19364.m	rs7864694	A	G	0.3364	0.0119	0.0026	4.04E-06	20.9482
M19364.m	rs8071524	A	G	0.3003	-0.0119	0.0026	4.55E-06	20.9482
M19364.m	rs947678	A	C	0.5736	-0.0117	0.0026	5.03E-06	20.25
M19364.m	rs9575156	A	G	0.0808	0.0485	0.0109	9.25E-06	19.7984
M19368.m	rs2263487	T	C	0.5184	-0.0119	0.0025	3.05E-06	22.6576
M19368.m	rs2277324	A	G	0.4138	0.0115	0.0026	8.96E-06	19.5636
M19368.m	rs2467042	T	G	0.8651	-0.0186	0.0042	7.84E-06	19.6122
M19368.m	rs2823491	T	C	0.9047	0.038	0.0083	5.27E-06	20.961
M19368.m	rs2826649	T	G	0.3649	-0.0115	0.0026	8.56E-06	19.5636
M19368.m	rs3112726	T	C	0.455	0.0122	0.0026	2.93E-06	22.0178
M19368.m	rs4344410	A	C	0.503	0.0119	0.0026	3.52E-06	20.9482
M19368.m	rs4778640	A	G	0.9704	0.04	0.0083	1.39E-06	23.2254
M19368.m	rs535902	A	G	0.9346	0.0252	0.0056	7.89E-06	20.25
M19368.m	rs606114	T	C	0.1601	-0.0164	0.0034	1.48E-06	23.2664
M19368.m	rs764428	T	C	0.6245	-0.0133	0.0026	2.46E-07	26.1672
M19368.m	rs9494155	A	C	0.5988	0.0123	0.0026	1.99E-06	22.3802
M19396.m	rs12375763	T	C	0.9372	0.0292	0.0065	7.58E-06	20.1808
M19396.m	rs17739309	A	G	0.845	-0.0215	0.0046	2.29E-06	21.8455
M19396.m	rs2100777	A	C	0.0558	0.0349	0.0079	9.98E-06	19.5163
M19396.m	rs3783200	A	C	0.6859	-0.0414	0.0073	1.51E-08	32.1629
M19396.m	rs4884317	A	G	0.211	-0.0211	0.0045	2.44E-06	21.9857
M19396.m	rs4998531	T	C	0.7039	0.0175	0.0037	2.32E-06	22.3703
M19396.m	rs7359707	A	T	0.1937	0.0235	0.0052	5.42E-06	20.4234
M19396.m	rs789736	T	G	0.2464	0.019	0.0038	4.03E-07	25
M19414.m	rs11188538	A	G	0.8687	-0.0221	0.0044	4.85E-07	25.2278
M19414.m	rs11623007	A	C	0.5644	0.012	0.0027	8.62E-06	19.7531
M19414.m	rs12028243	T	C	0.0717	-0.0567	0.0113	5.73E-07	25.1773
M19414.m	rs12152460	T	C	0.1557	0.021	0.0045	3.76E-06	21.7778
M19414.m	rs12961939	A	C	0.7434	0.0159	0.0034	3.77E-06	21.8694
M19414.m	rs1417207	A	G	0.7759	-0.0157	0.0035	6.03E-06	20.1216
M19414.m	rs1742707	T	G	0.4385	0.0122	0.0027	4.88E-06	20.417
M19414.m	rs1959400	T	G	0.5551	0.0122	0.0027	6.67E-06	20.417
M19414.m	rs4415526	T	C	0.4107	0.0137	0.0028	7.7E-07	23.9401
M19414.m	rs477580	A	G	0.2397	0.0168	0.0035	1.27E-06	23.04
M19414.m	rs6894183	A	T	0.8137	0.016	0.0035	6.03E-06	20.898
M19414.m	rs7151771	A	G	0.605	-0.0123	0.0027	5.02E-06	20.7531
M19414.m	rs818525	T	C	0.1436	0.0199	0.0044	6.46E-06	20.4551
M19414.m	rs921640	A	G	0.0331	-0.0732	0.0164	8.25E-06	19.9221
M19415.m	rs12936996	A	G	0.7358	-0.0192	0.0038	5.86E-07	25.5291
M19415.m	rs17422462	T	G	0.8375	-0.0218	0.0048	4.62E-06	20.6267
M19415.m	rs2964431	A	C	0.3897	0.0167	0.0037	7.85E-06	20.3718
M19415.m	rs3130663	A	G	0.9754	-0.1184	0.0247	1.55E-06	22.9779
M19415.m	rs4347230	A	G	0.3906	-0.0169	0.0037	6.04E-06	20.8627
M19415.m	rs7295482	A	G	0.9079	0.0304	0.0064	1.77E-06	22.5625

M19934.m	rs10086673	T	C	0.2938	0.0089	0.0018	5.26E-07	24.4475
M19934.m	rs10112981	A	G	0.287	-0.008	0.0018	6.12E-06	19.7531
M19934.m	rs10502069	A	G	0.7264	-0.0078	0.0018	8.39E-06	18.7778
M19934.m	rs10503714	A	G	0.9815	0.0313	0.0068	4.85E-06	21.1871
M19934.m	rs11787088	A	G	0.0475	0.0221	0.0046	1.63E-06	23.0818
M19934.m	rs11934095	A	G	0.7176	-0.0082	0.0018	3.07E-06	20.7531
M19934.m	rs12191236	C	G	0.6904	0.0083	0.0018	2.27E-06	21.2623
M19934.m	rs12589229	T	C	0.3252	0.0083	0.0018	2.41E-06	21.2623
M19934.m	rs12652381	A	G	0.6121	0.0091	0.0017	1.57E-07	28.654
M19934.m	rs12703488	A	G	0.0182	0.0361	0.0082	9.34E-06	19.3815
M19934.m	rs16996393	A	T	0.6946	-0.0083	0.0018	3.18E-06	21.2623
M19934.m	rs1764963	T	C	0.654	-0.008	0.0017	4.4E-06	22.1453
M19934.m	rs1944518	T	C	0.3068	-0.0086	0.0018	1.28E-06	22.8272
M19934.m	rs2160717	T	C	0.4042	-0.0087	0.0017	4.64E-07	26.1903
M19934.m	rs2489774	T	C	0.0339	0.0213	0.0047	6.46E-06	20.5383
M19934.m	rs2504067	A	G	0.4884	0.0079	0.0018	8.3E-06	19.2623
M19934.m	rs2642588	T	G	0.3129	-0.0079	0.0018	5.77E-06	19.2623
M19934.m	rs265487	A	G	0.4929	-0.0077	0.0017	7.56E-06	20.5156
M19934.m	rs2667755	T	C	0.6304	-0.0083	0.0017	1.84E-06	23.8374
M19934.m	rs2807592	T	G	0.6979	-0.0081	0.0018	4.45E-06	20.25
M19934.m	rs2834059	A	G	0.4831	-0.0091	0.0019	9.96E-07	22.9391
M19934.m	rs3004261	A	T	0.4168	0.0082	0.0017	1.84E-06	23.2664
M19934.m	rs3872668	C	G	0.3688	-0.0177	0.0036	7.34E-07	24.1736
M19934.m	rs4131290	A	C	0.702	-0.0126	0.0023	7.47E-08	30.0113
M19934.m	rs4399682	A	T	0.7244	0.0183	0.0039	2.06E-06	22.0178
M19934.m	rs4431081	T	C	0.4481	0.0164	0.0035	2.18E-06	21.9559
M19934.m	rs4541491	A	C	0.6185	-0.0078	0.0017	6.6E-06	21.0519
M19934.m	rs4659612	A	G	0.7371	0.0174	0.0038	5.92E-06	20.9668
M19934.m	rs4759824	T	C	0.6793	0.0083	0.0017	1.85E-06	23.8374
M19934.m	rs4788439	T	C	0.0772	-0.0271	0.0038	6.59E-13	50.8594
M19934.m	rs4808136	A	G	0.3392	0.0132	0.0018	5.42E-14	53.7778
M19934.m	rs5994710	A	G	0.0773	-0.0165	0.0034	1.16E-06	23.551
M19934.m	rs6500418	T	C	0.3694	-0.0079	0.0018	5.88E-06	19.2623
M19934.m	rs6948277	A	G	0.0135	0.0951	0.0195	1.01E-06	23.7844
M19934.m	rs722419	A	C	0.4468	0.008	0.0017	4.03E-06	22.1453
M19934.m	rs744093	A	G	0.892	-0.0238	0.0053	8.51E-06	20.1652
M19934.m	rs7544659	A	G	0.4048	0.0089	0.0017	3.13E-07	27.4083
M19934.m	rs7837995	T	C	0.4485	0.0089	0.0018	4.28E-07	24.4475
M19934.m	rs9317855	T	C	0.3513	-0.008	0.0017	4.35E-06	22.1453
M19934.m	rs9947181	C	G	0.0304	-0.0275	0.0056	9.24E-07	24.1151
M20489.m	rs10494322	T	C	0.8102	-0.0084	0.0017	1.43E-06	24.4152
M20489.m	rs10828691	A	G	0.7048	-0.0084	0.0017	6.05E-07	24.4152
M20489.m	rs11623335	A	G	0.8018	-0.008	0.0017	3.48E-06	22.1453
M20489.m	rs11787684	T	C	0.7863	-0.0084	0.0018	3.52E-06	21.7778
M20489.m	rs11853577	T	C	0.6055	0.0076	0.0016	3.75E-06	22.5625
M20489.m	rs11921640	A	T	0.8546	0.0113	0.0024	3.44E-06	22.1684
M20489.m	rs12134225	A	G	0.0991	0.012	0.0025	1.63E-06	23.04
M20489.m	rs12534545	A	G	0.1791	0.0091	0.0019	1.22E-06	22.9391
M20489.m	rs12911284	A	C	0.7545	0.0081	0.0017	2.26E-06	22.7024
M20489.m	rs129986	T	C	0.983	-0.0724	0.0145	5.55E-07	24.9311
M20489.m	rs13431652	T	C	0.7174	0.0099	0.0017	4.34E-09	33.9135
M20489.m	rs16856261	T	C	0.1002	0.0116	0.0025	4.06E-06	21.5296
M20489.m	rs17130	T	C	0.6691	-0.0074	0.0017	8.59E-06	18.9481
M20489.m	rs17616642	A	G	0.763	-0.0078	0.0017	7.49E-06	21.0519
M20489.m	rs1857095	T	C	0.9748	-0.023	0.005	3.79E-06	21.16

M20489.m	rs2159491	T	C	0.7764	-0.0155	0.0034	4.44E-06	20.7829
M20489.m	rs2497587	A	G	0.1887	-0.0092	0.0017	1.3E-07	29.2872
M20489.m	rs2501894	A	G	0.1704	-0.0096	0.0018	6.03E-08	28.4444
M20489.m	rs300110	A	G	0.8109	0.0079	0.0018	6.37E-06	19.2623
M20489.m	rs428823	T	C	0.795	0.0078	0.0017	6.48E-06	21.0519
M20489.m	rs4572706	A	G	0.197	-0.0084	0.0018	1.53E-06	21.7778
M20489.m	rs6604664	C	G	0.2321	0.0076	0.0017	8.9E-06	19.9862
M20489.m	rs6732398	T	C	0.9627	-0.034	0.0076	7.82E-06	20.0139
M20489.m	rs6814228	T	C	0.7738	0.0082	0.0017	1.99E-06	23.2664
M20489.m	rs6883962	T	C	0.089	-0.012	0.0025	1.63E-06	23.04
M20489.m	rs741160	A	G	0.8235	0.0093	0.0019	6.82E-07	23.9584
M20489.m	rs7717177	T	C	0.9739	-0.0416	0.0091	4.95E-06	20.898
M20489.m	rs7751392	T	G	0.1712	0.0082	0.0018	3.84E-06	20.7531
M20489.m	rs831831	A	G	0.2007	-0.0081	0.0017	3.2E-06	22.7024
M20489.m	rs888977	T	C	0.2126	0.0079	0.0017	4.4E-06	21.5952
M20489.m	rs9363753	T	C	0.1754	0.0089	0.0018	5.21E-07	24.4475
M20489.m	rs950156	A	G	0.2591	-0.008	0.0017	2.73E-06	22.1453
M20489.m	rs9511834	A	C	0.4232	-0.0073	0.0016	8.94E-06	20.8164
M20489.m	rs9548447	T	G	0.9001	0.0118	0.0025	2.47E-06	22.2784
M20489.m	rs9804190	T	C	0.2232	0.0076	0.0017	8.95E-06	19.9862
M20489.m	rs9837442	A	T	0.7138	-0.0074	0.0017	7.88E-06	18.9481
M20489.m	rs9915927	T	G	0.2687	0.0077	0.0017	5.76E-06	20.5156
M20489.m	rs9927153	T	C	0.2309	-0.0079	0.0017	5.47E-06	21.5952
M20675.m	rs10206655	A	C	0.0403	-0.036	0.008	6.78E-06	20.25
M20675.m	rs10508432	A	G	0.7069	-0.0124	0.0027	4.07E-06	21.0919
M20675.m	rs10746431	A	G	0.9808	0.0558	0.0111	5.1E-07	25.271
M20675.m	rs11119547	A	G	0.4892	0.0126	0.0026	1.85E-06	23.4852
M20675.m	rs1131718	A	G	0.8565	-0.0166	0.0037	5.71E-06	20.1286
M20675.m	rs11564708	C	G	0.8162	0.0155	0.0034	6.4E-06	20.7829
M20675.m	rs11580613	T	C	0.1344	0.0188	0.0042	8.68E-06	20.0363
M20675.m	rs11722368	T	C	0.3602	0.013	0.0027	1.03E-06	23.1824
M20675.m	rs12465802	A	G	0.31	-0.0364	0.0032	3.47E-29	129.391
M20675.m	rs12603532	T	C	0.0394	-0.0368	0.0074	6.62E-07	24.7305
M20675.m	rs12612955	T	C	0.9598	0.0393	0.0085	3.62E-06	21.377
M20675.m	rs12677067	A	G	0.29	-0.015	0.0033	5.51E-06	20.6612
M20675.m	rs13012539	T	G	0.6759	0.0135	0.0027	4.02E-07	25
M20675.m	rs1472578	A	T	0.6894	-0.0126	0.0027	2.44E-06	21.7778
M20675.m	rs1499686	A	G	0.8079	-0.0174	0.0035	7.49E-07	24.7151
M20675.m	rs1545472	A	G	0.5158	0.0118	0.0027	9.22E-06	19.1001
M20675.m	rs1687134	T	C	0.7206	0.0289	0.006	1.58E-06	23.2003
M20675.m	rs17212491	A	G	0.1645	0.016	0.0035	5.93E-06	20.898
M20675.m	rs214247	T	C	0.6068	-0.0119	0.0026	6.46E-06	20.9482
M20675.m	rs244763	A	G	0.3833	0.012	0.0027	8.7E-06	19.7531
M20675.m	rs3793126	A	G	0.6974	0.0123	0.0027	4.78E-06	20.7531
M20675.m	rs3800993	T	C	0.1697	0.0287	0.0036	1.53E-15	63.5563
M20675.m	rs4556997	A	C	0.1388	-0.0189	0.0042	6.4E-06	20.25
M20675.m	rs4671111	T	C	0.5592	-0.0126	0.0027	2E-06	21.7778
M20675.m	rs4742351	T	C	0.393	-0.0125	0.0027	2.81E-06	21.4335
M20675.m	rs4791715	T	G	0.594	-0.0126	0.0026	1.66E-06	23.4852
M20675.m	rs4954573	T	G	0.7291	0.0219	0.0033	2.53E-11	44.0413
M20675.m	rs5027944	T	C	0.5795	0.0122	0.0027	3.93E-06	20.417
M20675.m	rs5757568	A	G	0.388	0.0122	0.0027	5.1E-06	20.417
M20675.m	rs5998272	A	C	0.9257	0.0261	0.0053	1.08E-06	24.251
M20675.m	rs618900	C	G	0.8136	-0.016	0.0035	6E-06	20.898
M20675.m	rs6723890	A	G	0.9457	0.0262	0.0058	6.69E-06	20.4055

M20675.m	rs6736412	A	G	0.0412	-0.0396	0.0072	3.03E-08	30.25
M20675.m	rs7570971	A	C	0.3255	-0.0373	0.0027	7.86E-45	190.849
M20675.m	rs7915784	A	G	0.0662	0.0247	0.0053	2.65E-06	21.7191
M20675.m	rs9556974	C	G	0.3426	0.0135	0.0027	5.36E-07	25
M20675.m	rs9756306	A	G	0.3332	-0.0158	0.0027	3.64E-09	34.2442
M20699.x	rs1053941	T	G	0.2263	-0.0171	0.0026	4.99E-11	43.2559
M20699.x	rs10973759	A	G	0.8139	-0.0117	0.0026	8.25E-06	20.25
M20699.x	rs12137934	A	G	0.7021	0.0133	0.0026	2.05E-07	26.1672
M20699.x	rs12470336	A	T	0.97	-0.038	0.007	5.68E-08	29.4694
M20699.x	rs12629138	T	G	0.0631	0.0234	0.0044	1.29E-07	28.2831
M20699.x	rs12699171	A	G	0.9499	-0.0461	0.0099	3.03E-06	21.6836
M20699.x	rs17267298	T	C	0.7884	-0.0117	0.0026	6.68E-06	20.25
M20699.x	rs17318174	A	G	0.1785	0.0139	0.0026	1.28E-07	28.5814
M20699.x	rs17559164	T	C	0.027	0.045	0.01	6.8E-06	20.25
M20699.x	rs1869957	T	C	0.2369	-0.0114	0.0026	7.55E-06	19.2249
M20699.x	rs2011920	C	G	0.1375	-0.0154	0.0034	5.13E-06	20.5156
M20699.x	rs2165127	A	G	0.0444	-0.0751	0.0162	3.37E-06	21.4907
M20699.x	rs2295357	T	G	0.1554	0.0266	0.006	9.22E-06	19.6544
M20699.x	rs2404571	A	G	0.8739	0.0161	0.0034	2.92E-06	22.423
M20699.x	rs254840	A	G	0.0939	-0.0166	0.0035	3.03E-06	22.4947
M20699.x	rs356119	T	C	0.1068	0.0154	0.0035	9.2E-06	19.36
M20699.x	rs4431603	A	G	0.205	-0.0116	0.0026	8.6E-06	19.9053
M20699.x	rs4687717	T	C	0.4245	0.0116	0.0025	2.99E-06	21.5296
M20699.x	rs6106307	T	C	0.7398	0.0125	0.0026	8.85E-07	23.1139
M20699.x	rs6128265	T	C	0.0155	0.0797	0.0176	6.01E-06	20.5065
M20699.x	rs6552523	T	C	0.8163	-0.012	0.0027	6.28E-06	19.7531
M20699.x	rs6591040	T	G	0.5899	-0.0205	0.0044	3.34E-06	21.7071
M20699.x	rs6821567	C	G	0.9423	-0.0476	0.0104	4.38E-06	20.9482
M20699.x	rs7632086	C	G	0.8243	-0.0126	0.0027	2.47E-06	21.7778
M20699.x	rs7701594	T	C	0.1653	0.012	0.0027	7.45E-06	19.7531
M20699.x	rs780093	T	C	0.397	0.0117	0.0025	2.17E-06	21.9024
M20699.x	rs9814648	A	G	0.2592	0.012	0.0026	4.6E-06	21.3018
M21044.m	rs10161318	T	G	0.9568	0.0315	0.0069	5.1E-06	20.8412
M21044.m	rs11788042	A	G	0.2425	0.0163	0.0034	1.94E-06	22.9836
M21044.m	rs12470565	T	C	0.5681	0.0165	0.0034	1.11E-06	23.551
M21044.m	rs1260326	T	C	0.4046	0.0143	0.0027	7.87E-08	28.0508
M21044.m	rs1509960	A	C	0.0825	0.0259	0.0059	9.64E-06	19.2706
M21044.m	rs17101344	A	G	0.7777	-0.0189	0.0035	5.64E-08	29.16
M21044.m	rs17122565	T	C	0.2523	-0.0162	0.0035	2.75E-06	21.4237
M21044.m	rs2067997	T	C	0.8694	-0.0213	0.0043	9.4E-07	24.537
M21044.m	rs2130909	A	G	0.288	-0.018	0.004	6.8E-06	20.25
M21044.m	rs2861960	T	C	0.8651	-0.0193	0.0043	7.98E-06	20.1455
M21044.m	rs304729	A	G	0.1958	0.0155	0.0035	9.18E-06	19.6122
M21044.m	rs4388837	T	G	0.9828	0.1102	0.0239	4.15E-06	21.2602
M21044.m	rs4521014	A	G	0.4	-0.0166	0.0033	5.23E-07	25.3039
M21044.m	rs4670465	T	G	0.5498	-0.0144	0.0027	6.37E-08	28.4444
M21044.m	rs729253	T	C	0.5812	0.0262	0.0058	7.52E-06	20.4055
M21044.m	rs872745	A	C	0.4114	-0.0135	0.0027	4.93E-07	25
M21044.m	rs876927	T	G	0.2075	0.0158	0.0035	5.42E-06	20.3788
M21044.m	rs9389466	A	T	0.0282	0.0433	0.0097	7.97E-06	19.9266
M21047.m	rs11723606	T	C	0.1817	0.0121	0.0027	8.03E-06	20.0837
M21047.m	rs11905775	A	C	0.1708	0.0111	0.0024	3.96E-06	21.3906
M21047.m	rs12455580	T	C	0.3131	0.0102	0.0022	5.74E-06	21.4959
M21047.m	rs12498138	A	G	0.0744	-0.0199	0.0043	4.35E-06	21.4175
M21047.m	rs12515486	T	C	0.0969	-0.0148	0.0032	4.11E-06	21.3906

M21047.m	rs1391874	A	G	0.9599	0.023	0.0049	2.57E-06	22.0325
M21047.m	rs1440581	T	C	0.4567	-0.0122	0.0021	6.55E-09	33.7506
M21047.m	rs16948533	A	G	0.8805	-0.0224	0.0047	2.23E-06	22.7144
M21047.m	rs17262575	T	C	0.146	-0.0129	0.0029	7.37E-06	19.7872
M21047.m	rs17528586	A	G	0.0514	-0.0246	0.0053	3.1E-06	21.5436
M21047.m	rs1895981	T	C	0.841	0.014	0.003	3.06E-06	21.7778
M21047.m	rs2075383	T	C	0.9371	-0.0176	0.0038	3.86E-06	21.4515
M21047.m	rs209755	C	G	0.302	0.0099	0.0022	8.51E-06	20.25
M21047.m	rs243180	A	G	0.7695	0.0108	0.0023	3.16E-06	22.0491
M21047.m	rs703654	A	G	0.8216	-0.0117	0.0025	2.89E-06	21.9024
M21047.m	rs7814548	T	G	0.0725	0.0164	0.0037	8.81E-06	19.6465
M21047.m	rs791630	A	G	0.1945	-0.0112	0.0024	2.8E-06	21.7778
M21047.m	rs819566	T	C	0.25	0.0101	0.0023	9.41E-06	19.2836
M21049.m	rs10120252	A	G	0.8189	-0.0484	0.0087	3.03E-08	30.9494
M21049.m	rs10232298	A	G	0.3415	0.0321	0.0071	6.03E-06	20.4406
M21049.m	rs10847524	A	G	0.3904	-0.0344	0.0077	9.16E-06	19.9588
M21049.m	rs11205537	T	G	0.043	-0.0779	0.0175	8.33E-06	19.8152
M21049.m	rs12603646	A	C	0.7045	0.0348	0.0078	8.13E-06	19.9053
M21049.m	rs12616674	T	G	0.9778	0.1084	0.0238	5.05E-06	20.7446
M21049.m	rs1582961	T	C	0.2291	0.0423	0.0086	8.08E-07	24.1927
M21049.m	rs16849215	C	G	0.7736	-0.0369	0.0081	5.57E-06	20.7531
M21049.m	rs17065343	C	G	0.9322	-0.1273	0.0286	8.82E-06	19.8118
M21049.m	rs1785007	C	G	0.1024	0.1233	0.0242	3.35E-07	25.9594
M21049.m	rs2118838	C	G	0.8319	0.0422	0.0094	7.09E-06	20.1544
M21049.m	rs2393374	T	G	0.3095	0.0351	0.0078	6.39E-06	20.25
M21049.m	rs6832331	C	G	0.0215	0.2214	0.0495	7.67E-06	20.0053
M21049.m	rs6882058	T	C	0.7724	-0.0741	0.0167	9.37E-06	19.6881
M21049.m	rs9521664	T	C	0.9351	-0.0708	0.0152	3.24E-06	21.696
M21127.m	rs1000137	T	C	0.475	0.014	0.003	3.06E-06	21.7778
M21127.m	rs12145936	T	C	0.5511	0.0287	0.0063	6.21E-06	20.7531
M21127.m	rs1260326	T	C	0.4039	0.0134	0.0027	6.77E-07	24.631
M21127.m	rs12830350	T	G	0.3375	0.0124	0.0027	5.88E-06	21.0919
M21127.m	rs1544542	A	C	0.4642	0.0136	0.0027	5.4E-07	25.3717
M21127.m	rs2584543	A	G	0.0698	0.0246	0.0053	3.24E-06	21.5436
M21127.m	rs400511	T	C	0.6391	0.0125	0.0027	4.17E-06	21.4335
M21127.m	rs430508	A	G	0.4994	-0.0122	0.0027	7.02E-06	20.417
M21127.m	rs4774615	A	G	0.8406	-0.0184	0.0036	4.07E-07	26.1235
M21127.m	rs4821424	A	G	0.5199	-0.0125	0.0027	3.97E-06	21.4335
M21127.m	rs6089908	T	C	0.5596	-0.0122	0.0027	7.19E-06	20.417
M21127.m	rs757717	T	C	0.1121	0.0203	0.0045	8.12E-06	20.3501
M21127.m	rs954050	T	C	0.5912	0.0121	0.0027	7.68E-06	20.0837
M21151.x	rs11220964	A	G	0.0181	0.3839	0.0862	8.42E-06	19.8345
M21151.x	rs11773215	A	G	0.2525	0.0974	0.0219	8.62E-06	19.7802
M21151.x	rs12903092	A	T	0.0282	0.1524	0.0344	9.59E-06	19.627
M21151.x	rs2717871	A	G	0.8155	-0.0618	0.0137	6.64E-06	20.3487
M21151.x	rs3112989	A	G	0.5555	-0.0514	0.0109	2.23E-06	22.2368
M21151.x	rs6016809	T	C	0.9615	-0.1345	0.0282	1.82E-06	22.7482
M21151.x	rs7133595	A	G	0.3868	0.0519	0.0108	1.66E-06	23.0934
M21151.x	rs7628676	A	G	0.8283	-0.0653	0.0139	2.5E-06	22.0697
M21151.x	rs8078549	A	G	0.0266	0.1884	0.0407	3.6E-06	21.4276
M21151.x	rs822406	A	G	0.063	0.1625	0.0332	1.03E-06	23.9569
M21184.m	rs11627546	A	C	0.1703	0.0294	0.0065	6.87E-06	20.4582
M21184.m	rs17071035	A	G	0.8334	-0.0329	0.0067	8.57E-07	24.1125
M21184.m	rs17729999	A	G	0.0612	-0.0492	0.0108	5.23E-06	20.7531
M21184.m	rs182119	C	G	0.7302	-0.0272	0.0057	1.73E-06	22.7713

M21184.m	rs188595	A	C	0.653	0.0258	0.0056	3.49E-06	21.2258
M21184.m	rs1889891	T	C	0.2641	0.0257	0.0057	5.77E-06	20.329
M21184.m	rs2073030	A	T	0.7836	0.0287	0.0064	6.99E-06	20.1096
M21184.m	rs2143069	A	G	0.4261	-0.0234	0.005	2.64E-06	21.9024
M21184.m	rs2529488	T	C	0.8627	-0.0376	0.0073	2.32E-07	26.5296
M21184.m	rs4937147	A	G	0.1341	0.0379	0.0074	3.11E-07	26.231
M21184.m	rs7525018	T	G	0.7223	-0.0268	0.0058	3.64E-06	21.3508
M21184.m	rs7998013	A	G	0.9344	-0.0573	0.0124	4.08E-06	21.3533
M21184.m	rs9296444	T	C	0.7828	0.0267	0.0058	4.79E-06	21.1917
M21184.m	rs9313524	T	C	0.4146	0.0264	0.0059	8.91E-06	20.0218
M21184.m	rs964184	C	G	0.8615	-0.0443	0.0073	1.2E-09	36.8266
M21188.m	rs10739647	A	G	0.4429	-0.0135	0.0027	4.06E-07	25
M21188.m	rs10783765	T	G	0.7251	0.0135	0.0027	6.63E-07	25
M21188.m	rs10811336	A	C	0.8554	0.0169	0.0036	3.18E-06	22.0378
M21188.m	rs10878631	A	G	0.4509	-0.0125	0.0027	3.05E-06	21.4335
M21188.m	rs12026398	T	C	0.898	-0.0197	0.0043	4.1E-06	20.9892
M21188.m	rs12641040	A	G	0.8189	-0.0165	0.0035	2.38E-06	22.2245
M21188.m	rs12713384	A	G	0.7335	0.0123	0.0027	7.86E-06	20.7531
M21188.m	rs1456959	T	C	0.3972	0.013	0.0027	1.3E-06	23.1824
M21188.m	rs17260153	T	C	0.7525	-0.0133	0.0027	1.08E-06	24.2647
M21188.m	rs17436977	T	C	0.492	-0.0119	0.0027	8.1E-06	19.4252
M21188.m	rs17595468	A	G	0.864	0.0163	0.0036	5.89E-06	20.5008
M21188.m	rs2014576	A	G	0.4568	-0.0142	0.0027	1.15E-07	27.6598
M21188.m	rs2049974	A	G	0.296	-0.014	0.003	3.06E-06	21.7778
M21188.m	rs2173635	T	C	0.8642	-0.017	0.0037	3.3E-06	21.1103
M21188.m	rs2633998	T	C	0.0465	-0.0542	0.0122	8.54E-06	19.7369
M21188.m	rs2865986	A	G	0.6566	-0.0129	0.0027	1.63E-06	22.8272
M21188.m	rs3741298	T	C	0.782	-0.0325	0.0067	1.34E-06	23.5297
M21188.m	rs4266604	T	C	0.7099	-0.0121	0.0027	9.19E-06	20.0837
M21188.m	rs4711151	A	G	0.3776	-0.0124	0.0027	3.5E-06	21.0919
M21188.m	rs7032741	T	C	0.0891	-0.0205	0.0045	5.12E-06	20.7531
M21188.m	rs7152341	A	G	0.2566	0.0124	0.0027	5.26E-06	21.0919
M21188.m	rs7504246	A	G	0.2945	-0.0134	0.0027	7.62E-07	24.631
M21188.m	rs7746531	T	C	0.9206	0.0216	0.0045	2E-06	23.04
M21188.m	rs8094941	A	C	0.9697	-0.0371	0.0082	5.42E-06	20.4701
M21188.m	rs9964067	T	C	0.6778	-0.0119	0.0027	9.33E-06	19.4252
M21630.m	rs10140289	A	G	0.0295	0.0429	0.0075	9.59E-09	32.7184
M21630.m	rs12217249	A	T	0.9351	-0.0227	0.005	6.31E-06	20.6116
M21630.m	rs1255463	A	C	0.3988	-0.0113	0.0026	9.3E-06	18.8891
M21630.m	rs1255634	A	G	0.9601	-0.0344	0.0068	4.02E-07	25.5917
M21630.m	rs1271565	T	C	0.7287	-0.0164	0.0026	2.08E-10	39.787
M21630.m	rs12881815	A	G	0.055	0.0389	0.0052	8E-14	55.9619
M21630.m	rs13190937	A	G	0.3445	-0.0117	0.0026	6.58E-06	20.25
M21630.m	rs13255263	T	C	0.2518	-0.0125	0.0026	1.96E-06	23.1139
M21630.m	rs1520218	A	C	0.2558	0.0127	0.0026	1.39E-06	23.8595
M21630.m	rs2035247	T	G	0.8304	-0.016	0.0034	1.94E-06	22.1453
M21630.m	rs2239068	T	C	0.7471	0.0123	0.0027	3.78E-06	20.7531
M21630.m	rs230943	A	C	0.5083	-0.0122	0.0026	1.64E-06	22.0178
M21630.m	rs2314600	A	C	0.0144	-0.1047	0.0232	6.67E-06	20.3665
M21630.m	rs307128	A	G	0.681	-0.013	0.0026	4.33E-07	25
M21630.m	rs3755750	T	C	0.3156	-0.0123	0.0026	1.81E-06	22.3802
M21630.m	rs4719411	T	G	0.5112	-0.0115	0.0025	5.69E-06	21.16
M21630.m	rs4814176	T	C	0.3938	0.0216	0.0026	2.97E-17	69.0178
M21630.m	rs6508364	A	G	0.1404	-0.0159	0.0035	6.52E-06	20.6376
M21630.m	rs7157785	T	G	0.1649	0.0681	0.0034	7.23E-87	401.177

M21630.m	rs7214498	T	G	0.7012	0.0116	0.0026	8.16E-06	19.9053
M21630.m	rs7499814	A	C	0.8177	-0.0285	0.006	2.05E-06	22.5625
M21630.m	rs7512200	A	G	0.39	0.0113	0.0025	9.78E-06	20.4304
M21630.m	rs7522439	A	G	0.8977	0.0208	0.0042	7.34E-07	24.5261
M21630.m	rs7778975	T	C	0.4365	0.0116	0.0026	6.13E-06	19.9053
M21630.m	rs8022917	T	C	0.3151	-0.015	0.0026	5.66E-09	33.284
M21630.m	rs897656	A	T	0.0443	-0.0521	0.0113	4.06E-06	21.2578
M21630.m	rs9418817	T	C	0.311	0.0116	0.0026	6.67E-06	19.9053
M22030.m	rs10153390	T	G	0.4862	-0.0114	0.0025	6.59E-06	20.7936
M22030.m	rs10221174	T	C	0.1464	0.0163	0.0035	2.32E-06	21.689
M22030.m	rs10795164	T	C	0.9261	-0.0233	0.005	3.51E-06	21.7156
M22030.m	rs11160112	T	C	0.7128	-0.0146	0.0026	2.34E-08	31.5325
M22030.m	rs1159893	A	T	0.1347	0.016	0.0035	3.55E-06	20.898
M22030.m	rs11635023	A	G	0.508	-0.0152	0.0028	4.25E-08	29.4694
M22030.m	rs1277442	A	C	0.3106	0.0119	0.0026	3.21E-06	20.9482
M22030.m	rs1457613	A	C	0.8556	0.0167	0.0034	1.23E-06	24.1254
M22030.m	rs1795963	A	G	0.377	-0.0208	0.0026	1.27E-15	64
M22030.m	rs2054299	A	G	0.4546	-0.0114	0.0025	6.3E-06	20.7936
M22030.m	rs2278441	T	G	0.4291	0.0115	0.0025	5.68E-06	21.16
M22030.m	rs4433392	T	C	0.3697	0.0117	0.0026	5.1E-06	20.25
M22030.m	rs4631845	A	G	0.0851	0.0213	0.0043	9.87E-07	24.537
M22030.m	rs493519	T	C	0.1252	-0.0358	0.0041	1.32E-18	76.2427
M22030.m	rs6470588	A	C	0.5487	-0.0125	0.0026	1.34E-06	23.1139
M22030.m	rs674635	T	C	0.0415	0.0275	0.006	3.96E-06	21.0069
M22030.m	rs6860069	T	C	0.2918	0.0115	0.0026	7.73E-06	19.5636
M22030.m	rs7377975	A	G	0.189	0.018	0.004	6.8E-06	20.25
M22030.m	rs7934036	T	G	0.3475	0.0125	0.0025	9.76E-07	25
M22030.m	rs967725	A	T	0.0226	-0.0372	0.0082	6.15E-06	20.5806
M22032.m	rs10031381	A	G	0.9233	0.0254	0.0054	3.02E-06	22.1248
M22032.m	rs10785056	T	G	0.1146	0.0211	0.0046	4.81E-06	21.0402
M22032.m	rs10831643	T	C	0.4756	0.0127	0.0028	4.73E-06	20.5727
M22032.m	rs11170032	T	C	0.64	-0.0158	0.0036	9.43E-06	19.2623
M22032.m	rs12248518	T	C	0.186	0.0168	0.0037	5.15E-06	20.6165
M22032.m	rs12375569	A	G	0.2606	-0.0386	0.0081	2.16E-06	22.7093
M22032.m	rs12493507	T	C	0.1189	0.0201	0.0046	9.97E-06	19.0931
M22032.m	rs1260326	T	C	0.4038	0.0126	0.0028	4.92E-06	20.25
M22032.m	rs17806262	T	C	0.1745	-0.0168	0.0038	8.41E-06	19.5457
M22032.m	rs2059405	A	G	0.798	0.0167	0.0036	4.73E-06	21.5193
M22032.m	rs333063	T	C	0.0879	0.029	0.006	1.49E-06	23.3611
M22032.m	rs354468	T	C	0.1829	0.0172	0.0037	4.59E-06	21.6099
M22032.m	rs4147977	T	C	0.4483	-0.0132	0.0028	1.82E-06	22.2245
M22032.m	rs739167	A	G	0.8667	-0.0476	0.0102	3.25E-06	21.7778
M22032.m	rs7397059	T	C	0.5732	-0.0135	0.0028	1.22E-06	23.2462
M22032.m	rs815198	T	C	0.4745	-0.0126	0.0028	6.02E-06	20.25
M22116.m	rs10741500	A	T	0.041	-0.026	0.005	2.57E-07	27.04
M22116.m	rs10947808	T	C	0.8118	-0.012	0.0025	1.39E-06	23.04
M22116.m	rs11136678	A	G	0.715	-0.0119	0.0026	7.49E-06	20.9482
M22116.m	rs11564087	T	C	0.053	-0.028	0.006	3.06E-06	21.7778
M22116.m	rs12455580	T	C	0.3194	0.0122	0.0027	9.39E-06	20.417
M22116.m	rs1440581	T	C	0.4571	-0.0141	0.0022	2.43E-10	41.0764
M22116.m	rs17411832	A	C	0.7981	-0.0114	0.0025	3.21E-06	20.7936
M22116.m	rs276166	T	G	0.1052	-0.0243	0.0053	5.33E-06	21.0214
M22116.m	rs2933405	A	G	0.1507	0.0221	0.0049	5.88E-06	20.3419
M22116.m	rs320265	T	C	0.0765	-0.0174	0.0039	9.4E-06	19.9053
M22116.m	rs3798221	T	G	0.1914	0.0109	0.0024	8.31E-06	20.6267

M22116.m	rs4941615	T	C	0.2119	0.0133	0.0024	4.54E-08	30.7101
M22116.m	rs6569976	A	C	0.6201	0.0157	0.0035	7.33E-06	20.1216
M22116.m	rs791623	T	G	0.2041	-0.0116	0.0024	1.68E-06	23.3611
M22130.m	rs12155900	T	C	0.1217	-0.0174	0.0039	7.34E-06	19.9053
M22130.m	rs12355427	A	G	0.277	-0.0121	0.0027	9.2E-06	20.0837
M22130.m	rs12637114	T	C	0.541	0.0262	0.0059	9.65E-06	19.7196
M22130.m	rs12970549	A	G	0.7921	-0.0124	0.0028	8.16E-06	19.6122
M22130.m	rs13415138	T	C	0.2763	-0.0129	0.0027	2.9E-06	22.8272
M22130.m	rs1471834	A	G	0.7493	0.0136	0.0028	1.45E-06	23.5918
M22130.m	rs154440	T	C	0.6007	-0.0145	0.0027	9.4E-08	28.8409
M22130.m	rs1657179	T	C	0.5396	-0.0208	0.0027	9.38E-15	59.3471
M22130.m	rs17042584	T	G	0.7446	0.0127	0.0028	4.75E-06	20.5727
M22130.m	rs1821685	T	C	0.3252	0.013	0.0027	1.71E-06	23.1824
M22130.m	rs2032995	T	G	0.7853	0.0132	0.0027	1.35E-06	23.9012
M22130.m	rs4637923	T	C	0.3101	-0.012	0.0027	9.42E-06	19.7531
M22130.m	rs4784054	A	G	0.1063	0.0349	0.0037	2.31E-21	88.9708
M22130.m	rs4799200	A	G	0.0528	0.0252	0.0055	3.79E-06	20.9931
M22130.m	rs4872094	T	C	0.8154	-0.0327	0.0073	8.22E-06	20.0655
M22130.m	rs4952029	C	G	0.0436	0.0286	0.0062	3.71E-06	21.2789
M22130.m	rs6966840	A	G	0.7671	0.0133	0.0028	2.01E-06	22.5625
M22130.m	rs747902	A	G	0.6122	-0.0129	0.0027	2.13E-06	22.8272
M22130.m	rs7689395	T	C	0.0682	-0.0246	0.0052	2.34E-06	22.3802
M22130.m	rs9467632	A	G	0.8773	-0.0183	0.0037	8.5E-07	24.4624
M22138.m	rs11880965	T	C	0.7491	-0.0219	0.0045	1.28E-06	23.6844
M22138.m	rs12615551	A	G	0.9347	-0.0569	0.0125	5.47E-06	20.7207
M22138.m	rs2215015	T	C	0.3426	-0.0195	0.004	1.25E-06	23.7656
M22138.m	rs2243617	A	G	0.8747	-0.0284	0.0059	1.62E-06	23.1704
M22138.m	rs4452760	A	G	0.569	-0.0182	0.004	4.33E-06	20.7025
M22138.m	rs6534307	T	G	0.8647	-0.0248	0.0056	8.24E-06	19.6122
M22138.m	rs7989427	A	T	0.0528	0.0492	0.011	7.84E-06	20.0053
M22138.m	rs8101881	T	C	0.613	0.0325	0.0039	1.43E-16	69.4444
M22175.m	rs12143106	T	C	0.5922	0.0232	0.0051	4.77E-06	20.6936
M22175.m	rs13057057	T	C	0.0156	-0.0943	0.0212	8.37E-06	19.7857
M22175.m	rs16973789	A	G	0.036	-0.073	0.016	5.06E-06	20.8164
M22175.m	rs2031504	T	G	0.4374	0.0225	0.005	8.14E-06	20.25
M22175.m	rs2073824	A	G	0.6617	-0.0238	0.0051	2.8E-06	21.7778
M22175.m	rs4351	A	G	0.4754	-0.0455	0.005	1.24E-19	82.81
M22175.m	rs495828	T	G	0.217	-0.0312	0.0059	1.43E-07	27.9644
M22175.m	rs4968748	A	G	0.232	-0.0307	0.0059	1.83E-07	27.0753
M22177.m	rs10162458	T	C	0.7178	0.0087	0.0019	8.5E-06	20.9668
M22177.m	rs10514491	T	C	0.6719	0.0088	0.002	7.29E-06	19.36
M22177.m	rs109843	A	T	0.5886	-0.0087	0.0019	7.15E-06	20.9668
M22177.m	rs11152903	T	G	0.296	-0.0089	0.002	5.7E-06	19.8025
M22177.m	rs11222082	C	G	0.8851	-0.0137	0.0029	2.73E-06	22.3175
M22177.m	rs11776902	A	G	0.6567	-0.0091	0.0019	3.21E-06	22.9391
M22177.m	rs12406448	T	C	0.983	0.1301	0.0292	8.13E-06	19.8513
M22177.m	rs12498629	T	C	0.2767	-0.0086	0.002	9.34E-06	18.49
M22177.m	rs12616207	A	G	0.2818	0.0099	0.002	4.16E-07	24.5025
M22177.m	rs12787323	T	G	0.6649	0.0093	0.002	1.65E-06	21.6225
M22177.m	rs12871836	A	C	0.4654	-0.0093	0.0019	1.53E-06	23.9584
M22177.m	rs12996267	C	G	0.6148	-0.0103	0.0019	1.23E-07	29.3878
M22177.m	rs13268221	T	C	0.5305	-0.0088	0.0019	6.22E-06	21.4515
M22177.m	rs13278849	A	G	0.73	-0.013	0.002	2.79E-11	42.25
M22177.m	rs13395555	T	C	0.4714	0.0086	0.0019	8.25E-06	20.4875
M22177.m	rs1389052	T	C	0.7199	0.0087	0.002	8.95E-06	18.9225

M22177.m	rs1457192	A	G	0.272	-0.009	0.002	6.8E-06	20.25
M22177.m	rs17118209	T	C	0.9694	-0.0291	0.0066	9.68E-06	19.4401
M22177.m	rs17589032	T	G	0.0236	-0.0338	0.0076	8.7E-06	19.7791
M22177.m	rs1769582	A	G	0.2711	0.0089	0.002	5.58E-06	19.8025
M22177.m	rs1876088	A	C	0.6596	-0.0088	0.0019	5.77E-06	21.4515
M22177.m	rs1998125	T	G	0.5805	-0.0088	0.0019	5.75E-06	21.4515
M22177.m	rs2256490	A	T	0.2844	0.0087	0.0019	7.73E-06	20.9668
M22177.m	rs227375	T	C	0.4802	0.0097	0.0019	5.76E-07	26.0637
M22177.m	rs2286707	T	G	0.2687	-0.0087	0.002	9.5E-06	18.9225
M22177.m	rs2434210	A	T	0.8346	-0.0139	0.0029	1.3E-06	22.9738
M22177.m	rs2514587	A	G	0.6223	0.01	0.0019	3E-07	27.7008
M22177.m	rs2781537	T	C	0.5547	0.0087	0.0019	7.72E-06	20.9668
M22177.m	rs2870925	C	G	0.6398	-0.0088	0.0019	5.84E-06	21.4515
M22177.m	rs302149	A	G	0.9691	0.0258	0.0058	9.34E-06	19.7872
M22177.m	rs328370	T	G	0.722	0.0098	0.0019	5.3E-07	26.6039
M22177.m	rs3742766	A	G	0.0394	-0.0215	0.0048	8.44E-06	20.0629
M22177.m	rs3845671	C	G	0.4044	-0.0087	0.0019	7.34E-06	20.9668
M22177.m	rs4147331	A	G	0.393	0.0087	0.0019	7.47E-06	20.9668
M22177.m	rs4298477	T	C	0.534	0.0087	0.0019	8.4E-06	20.9668
M22177.m	rs4434479	A	G	0.2901	0.0096	0.0019	8.84E-07	25.5291
M22177.m	rs446125	T	G	0.1991	-0.0128	0.0029	9.27E-06	19.4816
M22177.m	rs4878628	T	C	0.2753	0.0106	0.002	6.18E-08	28.09
M22177.m	rs534408	T	G	0.7263	0.0088	0.0019	6.07E-06	21.4515
M22177.m	rs571139	A	G	0.3354	0.0093	0.0019	1.62E-06	23.9584
M22177.m	rs598236	T	C	0.7138	-0.0092	0.0019	2.28E-06	23.446
M22177.m	rs624947	A	G	0.6784	-0.0094	0.002	1.9E-06	22.09
M22177.m	rs625676	T	C	0.6649	-0.009	0.0019	3.67E-06	22.4377
M22177.m	rs6441104	A	G	0.1267	0.0139	0.0029	1.73E-06	22.9738
M22177.m	rs6476317	T	C	0.0729	0.0186	0.0039	1.68E-06	22.7456
M22177.m	rs6513545	A	G	0.6085	0.0088	0.0019	5.25E-06	21.4515
M22177.m	rs6738212	A	G	0.3037	-0.0089	0.0019	4.29E-06	21.9418
M22177.m	rs6874723	T	C	0.3661	0.0091	0.0019	3.17E-06	22.9391
M22177.m	rs6919834	T	C	0.1237	0.0144	0.0029	8.27E-07	24.6564
M22177.m	rs711995	T	C	0.4481	0.0108	0.0019	2.7E-08	32.3102
M22177.m	rs7194277	C	G	0.6775	-0.0087	0.0019	8.33E-06	20.9668
M22177.m	rs727233	A	G	0.7351	-0.0089	0.002	6.49E-06	19.8025
M22177.m	rs7541235	T	G	0.6521	-0.0103	0.0019	1.15E-07	29.3878
M22177.m	rs7677906	T	C	0.5094	0.0087	0.0019	7.07E-06	20.9668
M22177.m	rs7776725	T	C	0.7339	-0.0086	0.0019	9.27E-06	20.4875
M22177.m	rs7800932	T	G	0.4133	0.0088	0.0019	5.52E-06	21.4515
M22177.m	rs8180794	A	G	0.4133	0.0092	0.0019	2.02E-06	23.446
M22177.m	rs892857	T	C	0.6572	-0.0091	0.0019	2.83E-06	22.9391
M22177.m	rs922048	A	T	0.4049	-0.009	0.0019	3.33E-06	22.4377
M22177.m	rs9539356	A	C	0.7252	0.0089	0.0019	4.75E-06	21.9418
M22177.m	rs9547604	A	G	0.2551	-0.0087	0.002	7.72E-06	18.9225
M22189.m	rs10425252	T	C	0.6216	-0.0171	0.0039	9.23E-06	19.2249
M22189.m	rs1188835	T	C	0.5436	-0.0155	0.0034	4.09E-06	20.7829
M22189.m	rs12501542	A	C	0.8955	-0.0253	0.0051	7.87E-07	24.6094
M22189.m	rs155394	A	G	0.9262	-0.0291	0.0064	5.4E-06	20.6741
M22189.m	rs17157875	A	T	0.9213	-0.0284	0.006	2.32E-06	22.4044
M22189.m	rs2347322	A	G	0.9369	0.059	0.0133	8.88E-06	19.6789
M22189.m	rs2485904	A	C	0.1564	0.0206	0.0044	2.88E-06	21.9194
M22189.m	rs486786	T	G	0.535	0.0149	0.0034	9.09E-06	19.205
M22189.m	rs6738800	A	G	0.1176	0.0261	0.0057	5.2E-06	20.9668
M22481.m	rs1012455	A	G	0.5085	-0.0097	0.0019	2.16E-07	26.0637

M22481.m	rs10451238	A	G	0.8872	-0.0161	0.0036	6.15E-06	20.0008
M22481.m	rs10877921	A	G	0.0869	-0.0163	0.0036	8.25E-06	20.5008
M22481.m	rs10917314	T	C	0.4608	-0.0083	0.0019	9.53E-06	19.0831
M22481.m	rs10938307	T	C	0.4663	-0.0106	0.0019	1.48E-08	31.1247
M22481.m	rs12694202	T	C	0.4344	0.0093	0.0019	6.54E-07	23.9584
M22481.m	rs1284408	T	G	0.5194	-0.0269	0.0055	7.95E-07	23.921
M22481.m	rs12995493	A	G	0.5359	0.009	0.0019	1.84E-06	22.4377
M22481.m	rs13011429	C	G	0.1183	-0.0189	0.0035	6.9E-08	29.16
M22481.m	rs13021675	T	C	0.1941	0.0157	0.0028	1.2E-08	31.4401
M22481.m	rs16851514	C	G	0.0337	0.0272	0.0061	9.27E-06	19.8828
M22481.m	rs17699658	T	C	0.2136	0.0141	0.0027	2.28E-07	27.2716
M22481.m	rs2202185	A	G	0.3139	-0.0119	0.0026	6.16E-06	20.9482
M22481.m	rs2268943	A	T	0.0682	-0.0203	0.0045	7.1E-06	20.3501
M22481.m	rs2405867	A	G	0.1362	-0.0162	0.0036	5.44E-06	20.25
M22481.m	rs2671471	A	G	0.1641	-0.0131	0.0028	2.67E-06	21.889
M22481.m	rs278815	T	C	0.989	-0.1249	0.0275	5.73E-06	20.6281
M22481.m	rs2850874	A	G	0.0817	0.0171	0.0038	7.56E-06	20.25
M22481.m	rs2863821	A	G	0.4839	-0.0091	0.0019	1.41E-06	22.9391
M22481.m	rs3104893	T	C	0.5566	-0.0102	0.0019	6.03E-08	28.8199
M22481.m	rs3213827	A	G	0.5701	-0.0091	0.0019	1.1E-06	22.9391
M22481.m	rs3734747	A	G	0.164	-0.0127	0.0028	4.31E-06	20.5727
M22481.m	rs3798343	C	G	0.9771	-0.0362	0.0076	2.07E-06	22.6877
M22481.m	rs4279521	A	C	0.475	0.009	0.0019	1.42E-06	22.4377
M22481.m	rs4702556	T	C	0.3237	0.0129	0.0027	1.99E-06	22.8272
M22481.m	rs5768188	A	G	0.9868	0.0467	0.0097	1.44E-06	23.1788
M22481.m	rs625842	A	C	0.5072	0.0089	0.0019	1.85E-06	21.9418
M22481.m	rs6450714	A	G	0.981	-0.0871	0.0196	9.24E-06	19.748
M22481.m	rs6861890	T	C	0.5008	0.0083	0.0019	7.59E-06	19.0831
M22481.m	rs715	T	C	0.7175	-0.0327	0.0027	7.15E-35	146.679
M22481.m	rs7990504	A	C	0.8066	0.0128	0.0027	3.13E-06	22.4746
M22481.m	rs8056954	T	G	0.1734	-0.0121	0.0027	8.27E-06	20.0837
M22481.m	rs847852	A	G	0.4707	0.0087	0.0019	3E-06	20.9668
M22481.m	rs9375450	A	G	0.5099	0.0085	0.0019	5.63E-06	20.0139
M22481.m	rs942358	T	C	0.1543	-0.0132	0.0028	1.94E-06	22.2245
M22481.m	rs957671	A	G	0.9045	0.0168	0.0036	3.02E-06	21.7778
M22481.m	rs9668088	T	C	0.4178	-0.009	0.0019	1.28E-06	22.4377
M22481.m	rs9789183	T	C	0.517	0.0088	0.0019	3.27E-06	21.4515
M22548.m	rs1054634	A	G	0.8558	-0.016	0.0034	2.6E-06	22.1453
M22548.m	rs11117784	T	C	0.6373	-0.0119	0.0026	3.1E-06	20.9482
M22548.m	rs11134486	T	C	0.8302	-0.0154	0.0035	9.08E-06	19.36
M22548.m	rs11157393	T	C	0.5826	0.0117	0.0025	3.69E-06	21.9024
M22548.m	rs11675864	T	C	0.7367	0.0119	0.0026	5.84E-06	20.9482
M22548.m	rs11865472	A	G	0.506	-0.0146	0.0027	6.71E-08	29.2401
M22548.m	rs13160919	A	G	0.8729	0.0167	0.0035	2.35E-06	22.7665
M22548.m	rs1472923	T	C	0.1656	-0.0165	0.0033	8.09E-07	25
M22548.m	rs2820865	T	C	0.749	0.0114	0.0026	8.98E-06	19.2249
M22548.m	rs4073054	A	C	0.6227	0.013	0.0025	2.74E-07	27.04
M22548.m	rs6078040	T	C	0.9256	-0.0203	0.0044	4.82E-06	21.2856
M22548.m	rs628238	A	C	0.2548	-0.0144	0.0032	6.03E-06	20.25
M22548.m	rs6890028	T	C	0.2604	0.0258	0.0054	1.73E-06	22.8272
M22548.m	rs710719	C	G	0.3209	-0.0139	0.0026	5.58E-08	28.5814
M22548.m	rs805780	A	C	0.7634	0.0121	0.0026	3.47E-06	21.6583
M22649.m	rs10208071	A	G	0.689	0.0123	0.0026	1.51E-06	22.3802
M22649.m	rs10810813	A	G	0.1837	-0.0121	0.0027	5.2E-06	20.0837
M22649.m	rs11065301	T	C	0.3445	0.0113	0.0025	8.59E-06	20.4304

M22649.m	rs11776015	T	C	0.8309	0.0125	0.0027	3.52E-06	21.4335
M22649.m	rs13163520	A	G	0.8339	0.0121	0.0027	8.11E-06	20.0837
M22649.m	rs1929842	C	G	0.2049	0.013	0.0027	1.94E-06	23.1824
M22649.m	rs2186912	A	G	0.0475	0.023	0.0052	8.72E-06	19.5636
M22649.m	rs2280289	A	G	0.8397	-0.0131	0.0027	1.38E-06	23.5405
M22649.m	rs249462	A	T	0.3223	-0.0122	0.0026	2.45E-06	22.0178
M22649.m	rs2818310	T	C	0.8108	0.012	0.0027	6.45E-06	19.7531
M22649.m	rs603424	A	G	0.1806	-0.0121	0.0027	5.3E-06	20.0837
M22649.m	rs774211	T	C	0.8303	0.0137	0.0027	5.19E-07	25.7462
M22649.m	rs9477741	C	G	0.0113	-0.0851	0.0189	6.89E-06	20.2738
M22842.m	rs10999482	T	G	0.7102	-0.0496	0.011	7.28E-06	20.3319
M22842.m	rs1523437	A	G	0.8196	-0.0619	0.0132	2.75E-06	21.9904
M22842.m	rs16823145	A	G	0.9736	-0.2614	0.0587	8.58E-06	19.8306
M22842.m	rs17723514	A	G	0.6672	-0.0481	0.0102	2.3E-06	22.2377
M22842.m	rs1868259	A	G	0.5541	0.0818	0.0183	7.67E-06	19.9804
M22842.m	rs2689327	A	G	0.7541	0.0492	0.011	7.74E-06	20.0053
M22842.m	rs310271	A	G	0.2583	-0.0533	0.0117	5.53E-06	20.7531
M22842.m	rs6595543	A	G	0.3766	0.074	0.0165	7.19E-06	20.1139
M22842.m	rs9510021	A	C	0.5753	-0.0468	0.0103	5.77E-06	20.6451
M24074.m	rs10817879	T	C	0.5666	0.0116	0.0024	1.46E-06	23.3611
M24074.m	rs11106514	A	G	0.9495	-0.0331	0.0071	3.52E-06	21.734
M24074.m	rs11236928	C	G	0.1592	-0.0172	0.0033	1.5E-07	27.1662
M24074.m	rs12516773	A	G	0.5206	0.0118	0.0024	1.26E-06	24.1736
M24074.m	rs1534979	T	C	0.3253	-0.0112	0.0025	8.14E-06	20.0704
M24074.m	rs17260469	T	C	0.9112	0.0361	0.008	5.59E-06	20.3627
M24074.m	rs17756479	C	G	0.7131	0.0122	0.0025	1.2E-06	23.8144
M24074.m	rs2007443	A	T	0.1321	0.028	0.006	2.87E-06	21.7778
M24074.m	rs2034360	T	C	0.8625	0.0174	0.0037	2.73E-06	22.1154
M24074.m	rs2049216	C	G	0.1233	0.0201	0.0044	5.43E-06	20.8683
M24074.m	rs4075958	A	G	0.262	0.0115	0.0025	5.28E-06	21.16
M24074.m	rs4302786	A	T	0.0131	-0.0501	0.0112	7.43E-06	20.0096
M24074.m	rs4765356	A	G	0.8548	0.0149	0.0033	7.08E-06	20.3866
M24074.m	rs4798481	A	G	0.1447	-0.0153	0.0033	2.96E-06	21.4959
M24074.m	rs4811661	T	C	0.3137	-0.0113	0.0025	6.59E-06	20.4304
M24074.m	rs5995601	A	G	0.8544	0.0177	0.0037	1.7E-06	22.8846
M24074.m	rs6832163	A	G	0.2711	-0.016	0.0034	3.48E-06	22.1453
M24074.m	rs7945891	A	G	0.0587	0.0392	0.0088	8.09E-06	19.843
M24074.m	rs7988423	T	C	0.952	0.0397	0.009	9.28E-06	19.4579
M24074.m	rs8137744	A	G	0.3814	0.0123	0.0027	5.55E-06	20.7531
M25459.m	rs10209735	A	G	0.7536	-0.0127	0.0027	2.21E-06	22.1248
M25459.m	rs10227139	T	C	0.435	0.01	0.002	5.73E-07	25
M25459.m	rs1050457	A	G	0.531	-0.01	0.002	5.73E-07	25
M25459.m	rs10802883	A	G	0.3775	-0.0082	0.0018	6.65E-06	20.7531
M25459.m	rs11891474	A	G	0.974	0.063	0.0142	8.9E-06	19.6836
M25459.m	rs13127926	A	G	0.5059	-0.0091	0.0018	4.9E-07	25.5586
M25459.m	rs13222124	A	G	0.9863	-0.1154	0.0237	1.09E-06	23.7091
M25459.m	rs16936280	A	G	0.59	0.01	0.002	5.73E-07	25
M25459.m	rs17109682	C	G	0.9619	-0.0275	0.0062	9.48E-06	19.6735
M25459.m	rs17463576	A	G	0.4831	0.0084	0.0018	3.37E-06	21.7778
M25459.m	rs1760904	A	G	0.5414	-0.0081	0.0018	7.9E-06	20.25
M25459.m	rs1800775	A	C	0.4986	0.0108	0.0019	7.96E-09	32.3102
M25459.m	rs2223810	A	C	0.8498	-0.0128	0.0027	2.18E-06	22.4746
M25459.m	rs2895714	A	G	0.6755	0.0112	0.0025	9.64E-06	20.0704
M25459.m	rs289715	A	T	0.0895	0.0193	0.004	1.24E-06	23.2806
M25459.m	rs2920043	A	C	0.6233	-0.0084	0.0018	3.77E-06	21.7778

M25459.m	rs4073054	A	C	0.6255	0.0163	0.0018	4.42E-19	82.0031
M25459.m	rs4697525	T	C	0.546	-0.0091	0.0018	5.6E-07	25.5586
M25459.m	rs4937602	A	G	0.5448	0.0089	0.0018	9.63E-07	24.4475
M25459.m	rs575528	A	G	0.5409	0.0083	0.0018	4.6E-06	21.2623
M25459.m	rs5758064	T	C	0.5194	-0.0083	0.0018	3.91E-06	21.2623
M25459.m	rs6475805	T	C	0.514	-0.0087	0.0018	1.81E-06	23.3611
M25459.m	rs6566995	T	C	0.3657	-0.0083	0.0019	7.15E-06	19.0831
M25459.m	rs6986204	A	C	0.4755	-0.0082	0.0018	5.63E-06	20.7531
M25459.m	rs7144509	A	G	0.4147	-0.0089	0.0018	1.37E-06	24.4475
M25459.m	rs7160179	C	G	0.9807	-0.0701	0.0145	1.28E-06	23.3722
M25459.m	rs7242260	A	C	0.8606	0.0277	0.006	3.52E-06	21.3136
M25459.m	rs746764	T	C	0.1678	-0.0237	0.0052	6.12E-06	20.7726
M25459.m	rs7467889	T	C	0.4331	0.0081	0.0018	8.84E-06	20.25
M25459.m	rs7523745	T	C	0.4559	-0.0093	0.0018	3.42E-07	26.6944
M25459.m	rs7731057	T	C	0.7517	-0.0116	0.0026	6.37E-06	19.9053
M25459.m	rs884518	A	G	0.5041	-0.0087	0.0019	4.78E-06	20.9668
M25599.m	rs10078652	T	C	0.7163	0.0128	0.0027	2.85E-06	22.4746
M25599.m	rs10151267	T	C	0.3406	0.0142	0.0028	5.77E-07	25.7194
M25599.m	rs11864324	T	G	0.7671	0.0327	0.0074	9.39E-06	19.5268
M25599.m	rs12665495	A	G	0.9441	0.029	0.006	1.28E-06	23.3611
M25599.m	rs12946796	A	G	0.8939	0.0234	0.0044	8.83E-08	28.2831
M25599.m	rs13025976	A	T	0.6885	0.0123	0.0028	9.61E-06	19.2972
M25599.m	rs13049280	C	G	0.6544	0.0125	0.0027	4.13E-06	21.4335
M25599.m	rs13073181	C	G	0.3048	0.0128	0.0027	3.02E-06	22.4746
M25599.m	rs13095561	T	C	0.7452	0.013	0.0027	2.07E-06	23.1824
M25599.m	rs1429219	A	G	0.0353	0.033	0.0072	4.53E-06	21.0069
M25599.m	rs17526006	A	G	0.2579	0.0135	0.0028	1.04E-06	23.2462
M25599.m	rs2123489	A	G	0.7611	0.0123	0.0028	8.27E-06	19.2972
M25599.m	rs2211843	T	G	0.2444	-0.0126	0.0027	4.23E-06	21.7778
M25599.m	rs2934216	A	G	0.7472	-0.0128	0.0028	3.1E-06	20.898
M25599.m	rs3751834	A	G	0.8707	0.0167	0.0037	8.17E-06	20.3718
M25599.m	rs4073054	A	C	0.6266	0.0162	0.0027	2.15E-09	36
M25599.m	rs6681770	A	G	0.762	-0.0141	0.0028	3.91E-07	25.3584
M25599.m	rs6688960	A	G	0.9856	-0.1911	0.0362	1.34E-07	27.8679
M25599.m	rs6767775	A	G	0.4648	-0.0311	0.0061	4.06E-07	25.9933
M25599.m	rs7181047	T	C	0.2848	0.0123	0.0028	8.02E-06	19.2972
M25599.m	rs8057091	T	C	0.2657	-0.0123	0.0028	7.66E-06	19.2972
M27256.m	rs10124314	T	C	0.235	-0.01	0.002	5.73E-07	25
M27256.m	rs10467207	A	G	0.9094	-0.0126	0.0026	1.77E-06	23.4852
M27256.m	rs10489529	A	G	0.0955	0.0125	0.0026	1.47E-06	23.1139
M27256.m	rs11068516	A	C	0.1958	-0.0082	0.0018	3.71E-06	20.7531
M27256.m	rs1107270	T	C	0.71	0.0079	0.0017	4.44E-06	21.5952
M27256.m	rs11201158	T	C	0.0306	0.022	0.0048	5.42E-06	21.0069
M27256.m	rs11631900	T	C	0.424	-0.0192	0.0043	8.95E-06	19.9373
M27256.m	rs11855284	T	C	0.8068	-0.0088	0.0018	1.93E-06	23.9012
M27256.m	rs11915489	T	C	0.7958	0.0085	0.0018	1.87E-06	22.2994
M27256.m	rs12523450	A	G	0.8055	0.008	0.0018	6.99E-06	19.7531
M27256.m	rs1323688	T	C	0.2268	0.0087	0.0018	6.9E-07	23.3611
M27256.m	rs1330292	T	G	0.8039	-0.0082	0.0018	4.26E-06	20.7531
M27256.m	rs1379560	T	C	0.1931	-0.0085	0.0018	2.27E-06	22.2994
M27256.m	rs2159022	T	C	0.8241	0.0085	0.0018	1.96E-06	22.2994
M27256.m	rs2213261	T	C	0.9061	0.0115	0.0025	5.93E-06	21.16
M27256.m	rs2287011	A	G	0.2273	-0.009	0.0018	4.84E-07	25
M27256.m	rs2531899	A	G	0.1941	-0.0091	0.0018	4.86E-07	25.5586
M27256.m	rs278013	A	C	0.8065	0.0081	0.0018	5.51E-06	20.25

M27256.m	rs2876814	T	G	0.185	-0.0087	0.0018	1.08E-06	23.3611
M27256.m	rs445925	A	G	0.079	-0.018	0.004	6.8E-06	20.25
M27256.m	rs453766	C	G	0.7548	0.008	0.0017	4.23E-06	22.1453
M27256.m	rs4767915	A	G	0.0876	0.012	0.0027	7.95E-06	19.7531
M27256.m	rs4832148	T	C	0.8088	0.0086	0.0018	2.65E-06	22.8272
M27256.m	rs4899091	A	G	0.7678	-0.0083	0.0018	2.47E-06	21.2623
M27256.m	rs6126354	T	C	0.7911	-0.0194	0.004	9.75E-07	23.5225
M27256.m	rs660240	T	C	0.2096	-0.0082	0.0018	3.61E-06	20.7531
M27256.m	rs6894193	T	G	0.7848	0.0083	0.0018	2.92E-06	21.2623
M27256.m	rs708161	A	G	0.6965	-0.0076	0.0017	8.75E-06	19.9862
M27256.m	rs7200721	A	G	0.8128	-0.0079	0.0018	9.18E-06	19.2623
M27256.m	rs723078	C	G	0.9499	-0.0182	0.0037	6.84E-07	24.1958
M27256.m	rs7956757	T	C	0.8055	0.0081	0.0018	5.03E-06	20.25
M27256.m	rs7963469	A	G	0.2262	-0.0078	0.0018	8.49E-06	18.7778
M27256.m	rs807531	T	C	0.9382	-0.0152	0.0034	8.06E-06	19.9862
M27256.m	rs9518163	A	G	0.7373	-0.0085	0.0018	2.37E-06	22.2994
M27256.m	rs9520114	A	G	0.2732	0.0079	0.0017	3.7E-06	21.5952
M27256.m	rs9836183	T	C	0.2976	-0.0078	0.0017	6.82E-06	21.0519
M27256.m	rs984326	A	G	0.2537	0.0083	0.0017	1.5E-06	23.8374
M27273.m	rs11576061	A	G	0.1125	-0.0158	0.0034	3.19E-06	21.5952
M27273.m	rs11642383	A	G	0.753	0.0113	0.0025	7.79E-06	20.4304
M27273.m	rs13420395	A	G	0.0957	0.0154	0.0034	5.91E-06	20.5156
M27273.m	rs1421165	T	C	0.7334	0.0112	0.0025	8.15E-06	20.0704
M27273.m	rs1456374	T	C	0.6002	-0.0176	0.0039	7.04E-06	20.3655
M27273.m	rs17141888	C	G	0.9716	-0.0314	0.0065	1.13E-06	23.3363
M27273.m	rs17141888	C	G	0.9716	-0.0314	0.0065	1.13E-06	23.3363
M27273.m	rs2269713	A	G	0.9761	0.034	0.0075	5.65E-06	20.5511
M27273.m	rs2694777	A	G	0.8261	0.0138	0.003	5.6E-06	21.16
M27273.m	rs4287862	A	G	0.1739	0.0123	0.0026	2.16E-06	22.3802
M27273.m	rs6012418	A	G	0.0853	-0.0177	0.0035	3.82E-07	25.5747
M27273.m	rs6941976	T	G	0.0447	0.0447	0.0097	3.9E-06	21.2359
M27273.m	rs7184060	A	G	0.1847	0.0123	0.0026	1.8E-06	22.3802
M27273.m	rs7811011	C	G	0.0471	0.0219	0.0049	8.09E-06	19.9754
M27278.m	rs10140289	A	G	0.0294	0.0354	0.0069	2.9E-07	26.3214
M27278.m	rs11030066	T	C	0.1273	-0.0167	0.0035	2.13E-06	22.7665
M27278.m	rs11856508	T	C	0.9892	0.1008	0.0228	9.64E-06	19.5457
M27278.m	rs11890704	T	C	0.9322	-0.0257	0.0051	4.83E-07	25.3937
M27278.m	rs12337498	T	C	0.2157	-0.0129	0.0028	3.81E-06	21.2258
M27278.m	rs12768523	T	C	0.2137	-0.0131	0.0027	1.06E-06	23.5405
M27278.m	rs12965690	T	C	0.7726	-0.0125	0.0027	2.76E-06	21.4335
M27278.m	rs13117091	A	G	0.1954	0.0125	0.0027	4.89E-06	21.4335
M27278.m	rs13149458	T	C	0.0539	-0.0293	0.0064	4.34E-06	20.9592
M27278.m	rs17046742	A	G	0.1133	0.0161	0.0035	4.58E-06	21.16
M27278.m	rs17304995	A	G	0.7653	0.0123	0.0026	3.1E-06	22.3802
M27278.m	rs17531033	C	G	0.2505	0.0127	0.0026	1.32E-06	23.8595
M27278.m	rs1994851	A	G	0.0758	-0.0203	0.0043	2.69E-06	22.2872
M27278.m	rs322129	T	C	0.2023	-0.012	0.0027	7.74E-06	19.7531
M27278.m	rs3762660	T	G	0.8935	0.0199	0.0036	2.68E-08	30.5563
M27278.m	rs3849097	T	G	0.8494	-0.0161	0.0034	1.73E-06	22.423
M27278.m	rs4462847	T	G	0.6479	0.0221	0.005	9.82E-06	19.5364
M27278.m	rs4739066	A	G	0.9318	-0.0195	0.0044	9.51E-06	19.641
M27278.m	rs4814176	T	C	0.394	0.0157	0.0026	1.04E-09	36.463
M27278.m	rs4902243	A	G	0.8624	-0.0442	0.0035	3.41E-37	159.481
M27278.m	rs4949008	A	G	0.6154	0.012	0.0025	2.48E-06	23.04
M27278.m	rs6498634	T	G	0.7656	0.0118	0.0026	7.87E-06	20.5976

M27278.m	rs7160317	A	G	0.365	0.0118	0.0026	7.72E-06	20.5976
M27447.m	rs10237924	A	G	0.749	0.0382	0.0075	3.11E-07	25.942
M27447.m	rs10501071	A	G	0.7994	0.0359	0.0078	4.42E-06	21.1836
M27447.m	rs10824944	A	C	0.2569	-0.0326	0.0073	7.71E-06	19.943
M27447.m	rs11183846	A	G	0.9518	-0.0965	0.0212	5.49E-06	20.7197
M27447.m	rs11236675	T	C	0.3167	0.0348	0.0074	2.58E-06	22.1154
M27447.m	rs11825181	A	G	0.0766	0.0761	0.0114	2.93E-11	44.5615
M27447.m	rs11960972	A	G	0.9502	0.0781	0.0176	8.98E-06	19.6914
M27447.m	rs2130722	T	C	0.0738	-0.0666	0.0147	6.28E-06	20.5264
M27447.m	rs503905	C	G	0.5975	-0.029	0.0065	8.55E-06	19.9053
M27447.m	rs5997236	T	C	0.4	0.0298	0.0065	5.28E-06	21.0187
M27447.m	rs7490538	T	C	0.8854	-0.0447	0.0101	8.79E-06	19.5872
M27447.m	rs7804260	A	G	0.4115	0.0292	0.0065	7.01E-06	20.1808
M27447.m	rs804441	T	C	0.29	-0.0327	0.0072	5.87E-06	20.6267
M27447.m	rs9827772	A	G	0.9746	0.1061	0.0237	7.7E-06	20.0417
M27513.m	rs1013612	A	G	0.4965	-0.0125	0.0027	4.48E-06	21.4335
M27513.m	rs11043286	T	C	0.0188	0.057	0.0116	8.02E-07	24.1454
M27513.m	rs11242955	A	G	0.5528	-0.013	0.0027	1.67E-06	23.1824
M27513.m	rs11650196	A	G	0.0782	0.03	0.0061	7.59E-07	24.187
M27513.m	rs12591854	A	G	0.02	0.1135	0.0256	8.89E-06	19.6568
M27513.m	rs1458095	T	C	0.0913	-0.0235	0.0053	8.74E-06	19.66
M27513.m	rs16994074	T	C	0.8549	0.0199	0.0045	7.86E-06	19.556
M27513.m	rs17788495	A	G	0.9087	0.0234	0.0053	9.29E-06	19.4931
M27513.m	rs1886152	A	G	0.3789	0.0122	0.0027	8.34E-06	20.417
M27513.m	rs2002614	A	G	0.0114	0.0651	0.0142	4.55E-06	21.0177
M27513.m	rs2166764	T	C	0.4647	0.0123	0.0028	9.65E-06	19.2972
M27513.m	rs2494523	T	C	0.492	-0.014	0.003	3.06E-06	21.7778
M27513.m	rs289999	T	C	0.6577	-0.018	0.0035	1.91E-07	26.449
M27513.m	rs404632	T	C	0.1335	-0.0202	0.0044	5.36E-06	21.0764
M27513.m	rs4144140	T	C	0.4524	0.0125	0.0027	4.75E-06	21.4335
M27513.m	rs426434	T	C	0.1192	0.0203	0.0045	6.99E-06	20.3501
M27513.m	rs4324578	C	G	0.6112	0.013	0.0027	1.99E-06	23.1824
M27513.m	rs6497490	T	G	0.8823	-0.0305	0.0055	3.49E-08	30.7521
M27513.m	rs7197091	A	G	0.3026	-0.0178	0.0036	5.77E-07	24.4475
M27513.m	rs7809234	A	T	0.8755	0.0285	0.0045	1.6E-10	40.1111
M27513.m	rs792935	A	C	0.2048	-0.0165	0.0037	6.86E-06	19.8868
M27513.m	rs9319010	A	G	0.0156	0.1201	0.0262	4.44E-06	21.0128
M27531.m	rs10280608	A	C	0.8277	-0.0396	0.0087	4.96E-06	20.7182
M27531.m	rs10518972	T	C	0.11	0.0505	0.0105	1.35E-06	23.1315
M27531.m	rs10774496	A	G	0.1908	0.0397	0.0087	5.39E-06	20.823
M27531.m	rs10865016	A	G	0.1816	0.0453	0.0086	1.36E-07	27.7459
M27531.m	rs10954322	T	C	0.026	0.2208	0.0412	8.46E-08	28.7213
M27531.m	rs12527048	T	C	0.0167	0.1239	0.027	4.61E-06	21.0579
M27531.m	rs17651499	A	G	0.9423	0.1034	0.0211	9.13E-07	24.0146
M27531.m	rs2484454	A	G	0.8988	-0.1096	0.0222	7.71E-07	24.3733
M27531.m	rs2708754	A	G	0.3968	0.0312	0.0069	6.52E-06	20.4461
M27531.m	rs6024969	T	C	0.9884	-0.3856	0.0825	2.97E-06	21.8457
M27531.m	rs6450853	A	G	0.652	-0.0316	0.007	5.73E-06	20.3788
M27531.m	rs6957252	T	C	0.6329	0.0739	0.0149	6.84E-07	24.5989
M27531.m	rs6997473	A	G	0.9538	-0.0729	0.0161	6.29E-06	20.5023
M27531.m	rs7084667	T	C	0.3248	-0.0318	0.0071	6.47E-06	20.0603
M27531.m	rs7276515	T	C	0.8161	-0.0454	0.0087	2E-07	27.2316
M27531.m	rs7539563	T	G	0.2058	0.0397	0.0087	5.47E-06	20.823
M27531.m	rs9549759	T	C	0.4298	0.0337	0.0075	7.25E-06	20.19
M27672.m	rs10031835	T	C	0.6953	-0.034	0.0075	5.81E-06	20.5511

M27672.m	rs3847067	A	C	0.2877	-0.0316	0.007	6.69E-06	20.3788
M27672.m	rs527140	A	G	0.7481	-0.0329	0.0073	6.77E-06	20.3117
M27672.m	rs9379897	T	C	0.8707	-0.0261	0.0055	2.04E-06	22.5193
M27672.m	rs9489956	T	C	0.782	0.0395	0.0079	5.28E-07	25
M27710.m	rs10896833	T	C	0.9822	0.087	0.0186	3.04E-06	21.8783
M27710.m	rs11030392	A	G	0.3845	-0.0434	0.0092	2.61E-06	22.2538
M27710.m	rs12328639	A	G	0.85	0.0342	0.0062	2.75E-08	30.4277
M27710.m	rs12468557	T	C	0.373	-0.0293	0.0044	1.84E-11	44.3435
M27710.m	rs12470532	T	C	0.6097	0.0228	0.005	5.45E-06	20.7936
M27710.m	rs12661239	A	G	0.61	0.0244	0.005	1.26E-06	23.8144
M27710.m	rs13011429	C	G	0.1226	-0.0349	0.0073	1.71E-06	22.8563
M27710.m	rs13021675	T	C	0.1968	0.0485	0.006	5.6E-16	65.3403
M27710.m	rs1367053	T	C	0.4544	0.0306	0.0044	4.88E-12	48.3657
M27710.m	rs16844777	T	C	0.1255	0.0316	0.007	5.52E-06	20.3788
M27710.m	rs1701843	A	G	0.6074	-0.0194	0.0044	8.9E-06	19.4401
M27710.m	rs17709912	A	G	0.7757	0.0472	0.0105	6.41E-06	20.2072
M27710.m	rs1990797	C	G	0.1707	-0.0486	0.0061	1.33E-15	63.4765
M27710.m	rs2997201	A	G	0.614	-0.0195	0.0044	7.42E-06	19.641
M27710.m	rs472847	T	C	0.3494	0.0243	0.0054	7.14E-06	20.25
M27710.m	rs4774192	T	C	0.1306	0.0709	0.0137	2.06E-07	26.7825
M27710.m	rs715	T	C	0.7096	-0.091	0.0057	6.66E-58	254.878
M27710.m	rs780245	T	C	0.1757	0.0289	0.0059	1.15E-06	23.9934
M27710.m	rs7948073	T	G	0.5702	-0.0256	0.0044	4.6E-09	33.8512
M27710.m	rs8057910	T	C	0.0712	-0.0391	0.0088	8.52E-06	19.7419
M27710.m	rs805936	A	G	0.9841	-0.0897	0.0184	1.16E-06	23.7656
M27710.m	rs840381	T	C	0.6429	0.0194	0.0044	9.76E-06	19.4401
M27716.m	rs10170160	T	C	0.5654	0.0398	0.0086	3.54E-06	21.4175
M27716.m	rs10215516	A	G	0.5372	0.0499	0.0108	3.85E-06	21.3478
M27716.m	rs1076454	T	C	0.1076	0.0506	0.01	4.75E-07	25.6036
M27716.m	rs12479240	T	C	0.1693	-0.0549	0.0083	3.11E-11	43.7511
M27716.m	rs12622156	A	G	0.1411	0.0436	0.0091	1.45E-06	22.9557
M27716.m	rs13287132	A	G	0.8214	0.0656	0.0143	4.8E-06	21.0444
M27716.m	rs249619	T	C	0.5601	-0.0533	0.0112	1.79E-06	22.6474
M27716.m	rs28900385	T	C	0.9671	-0.1406	0.0161	2.85E-18	76.2639
M27716.m	rs3771342	T	G	0.1117	-0.0612	0.0094	6.38E-11	42.3884
M27716.m	rs3796092	A	C	0.0978	0.0797	0.0098	4.53E-16	66.14
M27716.m	rs3848289	A	G	0.0421	-0.0694	0.0157	9.41E-06	19.5398
M27716.m	rs4738080	A	G	0.0687	0.0555	0.012	3.44E-06	21.3906
M27716.m	rs4918207	T	C	0.4099	0.0361	0.0079	4.36E-06	20.8814
M27716.m	rs550866	T	G	0.4015	0.0263	0.0059	8.55E-06	19.8704
M27716.m	rs6717546	A	G	0.3655	-0.0565	0.0059	2.02E-21	91.705
M27716.m	rs6760588	A	C	0.7482	0.0597	0.0068	1.13E-18	77.0781
M27716.m	rs7659567	A	G	0.2037	0.0337	0.0074	5.01E-06	20.7394
M27716.m	rs838718	A	G	0.4853	0.0386	0.0058	3.45E-11	44.2913
M27716.m	rs887829	T	C	0.3265	0.1677	0.0064	2.2E-152	686.604
M27718.m	rs10900120	A	G	0.7361	-0.0383	0.008	1.74E-06	22.9202
M27718.m	rs10932349	C	G	0.9216	0.03	0.0066	6.51E-06	20.6612
M27718.m	rs1145077	T	G	0.3769	0.0187	0.0037	3.99E-07	25.5435
M27718.m	rs11694063	T	C	0.1962	-0.0224	0.0046	1.37E-06	23.7127
M27718.m	rs1230164	C	G	0.721	0.0176	0.0038	3.08E-06	21.4515
M27718.m	rs12820743	A	G	0.5528	-0.0168	0.0037	5.06E-06	20.6165
M27718.m	rs3111102	A	G	0.0953	-0.048	0.0107	7E-06	20.124
M27718.m	rs3817670	T	C	0.0384	-0.0743	0.0167	8.42E-06	19.7945
M27718.m	rs4130275	T	G	0.8327	0.0214	0.0047	5.4E-06	20.7316
M27718.m	rs715	T	C	0.7113	-0.0451	0.0044	9.63E-25	105.063

M27718.m	rs7319920	A	G	0.1522	-0.0219	0.0048	4.39E-06	20.8164
M27722.m	rs11891974	A	G	0.3109	-0.0183	0.0038	1.35E-06	23.1918
M27722.m	rs12216333	T	G	0.1744	-0.0211	0.0047	5.99E-06	20.1544
M27722.m	rs17046195	A	C	0.9366	0.0324	0.0073	8.2E-06	19.699
M27722.m	rs4692675	T	C	0.0135	0.0861	0.0187	4.36E-06	21.1994
M27722.m	rs6486548	C	G	0.8143	-0.0502	0.0109	4.35E-06	21.2107
M27722.m	rs7289147	T	G	0.3413	0.0168	0.0038	8.31E-06	19.5457
M27722.m	rs934668	T	C	0.7739	0.0252	0.0056	6.78E-06	20.25
M27722.m	rs9418711	T	G	0.2281	0.0205	0.0046	7.49E-06	19.8606
M27728.x	rs10117043	T	G	0.156	0.0161	0.0036	6.13E-06	20.0008
M27728.x	rs10953489	T	C	0.7961	-0.0157	0.0036	9.69E-06	19.0193
M27728.x	rs1105769	A	C	0.3077	-0.0135	0.0027	7.19E-07	25
M27728.x	rs11078249	T	C	0.6743	-0.0125	0.0027	3.67E-06	21.4335
M27728.x	rs1234223	C	G	0.0908	0.0211	0.0045	3.02E-06	21.9857
M27728.x	rs1317833	A	G	0.6631	-0.0125	0.0027	4.38E-06	21.4335
M27728.x	rs1401635	C	G	0.3132	-0.0125	0.0027	4.91E-06	21.4335
M27728.x	rs17359373	T	C	0.261	-0.0323	0.0072	7.85E-06	20.1252
M27728.x	rs2041992	T	C	0.3478	-0.0122	0.0027	8.55E-06	20.417
M27728.x	rs210331	A	G	0.8206	-0.0179	0.0035	3.53E-07	26.1559
M27728.x	rs2114588	A	G	0.3114	-0.0122	0.0027	6.99E-06	20.417
M27728.x	rs2235396	A	G	0.695	-0.012	0.0027	9.66E-06	19.7531
M27728.x	rs2253834	T	C	0.5521	-0.0125	0.0027	4.55E-06	21.4335
M27728.x	rs2422239	A	G	0.542	-0.0118	0.0027	9.52E-06	19.1001
M27728.x	rs2982376	T	C	0.4212	-0.0132	0.0028	2.93E-06	22.2245
M27728.x	rs4236964	T	C	0.3256	-0.0123	0.0027	5.74E-06	20.7531
M27728.x	rs4860118	T	C	0.9631	0.0716	0.0151	2.04E-06	22.4839
M27728.x	rs4901357	A	G	0.3396	-0.0134	0.0028	1.25E-06	22.9031
M27728.x	rs498494	C	G	0.9184	0.0258	0.0056	4.27E-06	21.2258
M27728.x	rs552279	A	G	0.6732	-0.0129	0.0028	4.9E-06	21.2258
M27728.x	rs6699475	A	G	0.245	-0.0152	0.0034	8.91E-06	19.9862
M27728.x	rs7095787	A	T	0.788	0.0185	0.0035	1.58E-07	27.9388
M27728.x	rs7121554	T	C	0.2534	-0.016	0.0035	5.21E-06	20.898
M27728.x	rs7275210	A	T	0.4077	0.012	0.0027	9.31E-06	19.7531
M27728.x	rs734567	T	C	0.5906	0.0134	0.0027	7.83E-07	24.631
M27728.x	rs7428286	A	G	0.0142	-0.0561	0.0121	3.88E-06	21.4959
M27728.x	rs757704	A	G	0.5037	0.0138	0.0027	2.22E-07	26.1235
M27728.x	rs7929077	A	G	0.4899	0.0121	0.0027	6.63E-06	20.0837
M27728.x	rs8124907	A	G	0.7514	0.0307	0.0068	5.85E-06	20.3826
M27728.x	rs908690	T	C	0.8554	-0.0161	0.0036	7.83E-06	20.0008
M27728.x	rs9471691	T	C	0.6461	0.0139	0.0027	2.77E-07	26.5034
M27728.x	rs9608300	C	G	0.0264	-0.137	0.0281	1.06E-06	23.77
M27738.m	rs10097949	A	G	0.9488	0.0475	0.0102	3.41E-06	21.6864
M27738.m	rs10512185	T	C	0.6502	0.0166	0.0036	3.32E-06	21.2623
M27738.m	rs11914582	T	C	0.3531	-0.0307	0.0066	2.89E-06	21.6366
M27738.m	rs12447958	A	G	0.7028	0.0188	0.0042	7.57E-06	20.0363
M27738.m	rs12779976	A	G	0.9388	0.0347	0.0072	1.26E-06	23.227
M27738.m	rs13163920	T	C	0.3017	0.016	0.0036	9.51E-06	19.7531
M27738.m	rs2257061	T	C	0.6471	-0.0162	0.0036	6.33E-06	20.25
M27738.m	rs2303010	T	C	0.0102	-0.1471	0.0311	2.26E-06	22.372
M27738.m	rs306472	T	C	0.2222	0.0197	0.0044	6.69E-06	20.046
M27738.m	rs3766606	T	G	0.1659	0.0207	0.0045	4.93E-06	21.16
M27738.m	rs576266	T	C	0.9071	-0.0284	0.0063	5.97E-06	20.3215
M27738.m	rs7132285	T	C	0.0108	0.1371	0.0303	6.12E-06	20.4734
M27738.m	rs7216075	A	G	0.2494	-0.0215	0.0043	4.39E-07	25
M27738.m	rs7332927	A	G	0.9802	0.1384	0.0298	3.37E-06	21.5695

M27738.m	rs7993666	T	C	0.1714	0.0207	0.0045	3.67E-06	21.16
M27738.m	rs8031609	T	G	0.8038	0.0198	0.0044	7.45E-06	20.25
M27738.m	rs892429	A	G	0.9571	0.0839	0.019	9.86E-06	19.4992
M27738.m	rs9572687	A	G	0.7063	-0.0162	0.0036	6.55E-06	20.25
M30805.m	rs10495696	A	G	0.9526	-0.0288	0.0064	6.35E-06	20.25
M30805.m	rs11893386	T	C	0.9023	0.0172	0.0038	6.62E-06	20.4875
M30805.m	rs12522646	T	C	0.7914	-0.0141	0.0028	7.2E-07	25.3584
M30805.m	rs12728857	A	G	0.5552	0.0133	0.0028	2.04E-06	22.5625
M30805.m	rs12994495	T	G	0.9488	0.0248	0.0056	8.17E-06	19.6122
M30805.m	rs13115084	A	G	0.792	0.0145	0.0028	3.1E-07	26.8176
M30805.m	rs1545442	T	C	0.8127	0.0134	0.0029	2.87E-06	21.3508
M30805.m	rs1562445	A	G	0.1935	0.0125	0.0028	9.56E-06	19.9298
M30805.m	rs17694346	A	C	0.9783	0.0479	0.0104	3.96E-06	21.2131
M30805.m	rs2016976	T	C	0.2607	-0.0142	0.0028	4.1E-07	25.7194
M30805.m	rs281499	A	G	0.5873	0.0149	0.0028	7.82E-08	28.3176
M30805.m	rs4148088	C	G	0.926	0.025	0.005	5.73E-07	25
M30805.m	rs559135	T	C	0.2066	-0.0141	0.0028	6.72E-07	25.3584
M30805.m	rs6890263	T	C	0.9379	0.0221	0.0047	3.18E-06	22.11
M30805.m	rs7397233	A	G	0.0335	0.098	0.0214	4.47E-06	20.9713
M30805.m	rs8081052	A	G	0.1073	0.0174	0.0038	4.64E-06	20.9668
M30805.m	rs845251	A	C	0.9036	-0.0171	0.0039	8.7E-06	19.2249
M31453.m	rs10926975	T	C	0.2524	-0.0125	0.0027	2.66E-06	21.4335
M31453.m	rs10976932	A	G	0.7662	-0.0117	0.0026	8.61E-06	20.25
M31453.m	rs11713161	A	G	0.8575	-0.017	0.0034	5.63E-07	25
M31453.m	rs12052126	T	C	0.1205	0.0389	0.0085	4.18E-06	20.9441
M31453.m	rs13179336	A	T	0.2062	0.0154	0.0034	4.85E-06	20.5156
M31453.m	rs13293620	T	C	0.0166	0.1054	0.0216	1.07E-06	23.8108
M31453.m	rs13378579	A	C	0.0303	-0.0711	0.0156	4.81E-06	20.7726
M31453.m	rs13378814	T	C	0.982	0.0381	0.0086	9.68E-06	19.627
M31453.m	rs1470963	A	G	0.6088	0.0208	0.0047	8.97E-06	19.5853
M31453.m	rs1948930	A	G	0.297	0.012	0.0026	4.21E-06	21.3018
M31453.m	rs2314026	A	G	0.9644	0.0283	0.006	2.53E-06	22.2469
M31453.m	rs2710833	T	C	0.1979	-0.0132	0.0027	1.2E-06	23.9012
M31453.m	rs4602889	A	G	0.8859	0.016	0.0035	3.57E-06	20.898
M31453.m	rs6003971	T	G	0.9363	0.0231	0.0052	8.1E-06	19.7341
M31453.m	rs6920798	A	G	0.787	-0.0117	0.0026	9.51E-06	20.25
M31453.m	rs7127674	A	G	0.0147	-0.0482	0.0104	3.33E-06	21.4797
M31453.m	rs7932167	T	G	0.7711	0.0119	0.0026	6.66E-06	20.9482
M31453.m	rs9572772	T	G	0.0129	-0.0902	0.0197	4.65E-06	20.9643
M31453.m	rs9607409	A	G	0.1215	0.0156	0.0035	7.25E-06	19.8661
M31522.m	rs11603058	A	T	0.0414	0.1122	0.024	2.84E-06	21.8556
M31522.m	rs13398058	A	G	0.6148	0.038	0.0084	5.76E-06	20.4649
M31522.m	rs241323	A	G	0.1166	-0.0794	0.0167	1.88E-06	22.6052
M31522.m	rs4677337	A	G	0.3536	-0.0534	0.012	8.99E-06	19.8025
M31536.m	rs10072432	T	C	0.0175	0.3673	0.0754	1.1E-06	23.7301
M31536.m	rs10822916	A	C	0.8247	-0.0884	0.0199	8.78E-06	19.7332
M31536.m	rs10995717	T	G	0.0683	0.1194	0.0262	5.23E-06	20.7685
M31536.m	rs11158090	A	G	0.1877	0.0787	0.0178	9.64E-06	19.5483
M31536.m	rs11227132	A	G	0.9845	-0.4069	0.0876	3.4E-06	21.5758
M31536.m	rs12540531	A	C	0.1129	0.1022	0.0227	6.74E-06	20.2698
M31536.m	rs12759216	A	G	0.9097	-0.1242	0.0255	1.05E-06	23.7226
M31536.m	rs1418200	T	C	0.0408	0.2117	0.0426	6.63E-07	24.6958
M31536.m	rs17180489	C	G	0.012	0.611	0.137	8.2E-06	19.8903
M31536.m	rs2073779	T	C	0.9759	-0.2562	0.0514	6.09E-07	24.8446
M31536.m	rs2277503	A	G	0.0502	0.1667	0.0302	3.43E-08	30.4689

M31536.m	rs2838057	A	C	0.9626	-0.1987	0.0368	6.91E-08	29.1541
M31536.m	rs3123713	A	G	0.0862	0.114	0.0249	4.88E-06	20.961
M31536.m	rs3742253	C	G	0.1923	0.0849	0.0191	8.78E-06	19.7583
M31536.m	rs3794836	A	G	0.0439	0.1656	0.0351	2.42E-06	22.259
M31536.m	rs4246848	T	C	0.15	-0.0888	0.0201	9.53E-06	19.5179
M31536.m	rs4384785	A	G	0.1018	0.1372	0.0294	3.16E-06	21.7778
M31536.m	rs626720	A	G	0.1195	0.1018	0.0214	1.86E-06	22.6291
M31536.m	rs6994108	A	G	0.0236	0.2358	0.0509	3.68E-06	21.4611
M31536.m	rs7039553	T	C	0.6695	-0.0694	0.015	3.56E-06	21.406
M31536.m	rs7165202	T	C	0.9469	-0.1564	0.0323	1.29E-06	23.446
M31536.m	rs7315595	T	G	0.7851	-0.0838	0.0182	3.95E-06	21.2005
M31536.m	rs737853	A	G	0.617	-0.0736	0.0158	3.05E-06	21.6991
M31536.m	rs749429	T	G	0.9406	-0.142	0.0299	2.07E-06	22.5546
M31536.m	rs7638353	A	C	0.5531	-0.1359	0.0301	6.48E-06	20.3848
M31536.m	rs7789832	T	C	0.0751	0.1267	0.0286	9.21E-06	19.6255
M31536.m	rs7797678	T	C	0.0159	0.2817	0.0629	7.6E-06	20.0573
M31536.m	rs873107	T	C	0.6472	-0.0677	0.0146	3.64E-06	21.5016
M31536.x	rs10072432	T	C	0.0175	0.3673	0.0754	1.1E-06	23.7301
M31536.x	rs11227132	A	G	0.9845	-0.4069	0.0876	3.4E-06	21.5758
M31536.x	rs11246881	C	G	0.9677	-0.1783	0.0382	3.03E-06	21.7859
M31536.x	rs12780044	T	C	0.971	-0.1675	0.036	3.24E-06	21.6483
M31536.x	rs13316446	A	G	0.2814	0.0553	0.0121	5.32E-06	20.8872
M31536.x	rs1418200	T	C	0.0408	0.2117	0.0426	6.63E-07	24.6958
M31536.x	rs1506811	T	C	0.3955	-0.067	0.0146	4.32E-06	21.0593
M31536.x	rs1707082	T	G	0.9681	-0.1567	0.0341	4.33E-06	21.1169
M31536.x	rs17809746	A	G	0.0519	0.1197	0.0258	3.48E-06	21.5253
M31536.x	rs2277503	A	G	0.0494	0.1351	0.0287	2.47E-06	22.1588
M31536.x	rs2501985	A	C	0.1715	-0.0684	0.0145	2.3E-06	22.2524
M31536.x	rs259783	A	G	0.0574	0.1144	0.0249	4.56E-06	21.1083
M31536.x	rs260728	A	G	0.9159	-0.1014	0.0198	2.86E-07	26.2268
M31536.x	rs4323189	A	T	0.0351	0.1403	0.03	2.88E-06	21.8712
M31536.x	rs4837770	T	C	0.0486	0.1533	0.0301	3.51E-07	25.9389
M31536.x	rs4943128	A	G	0.0108	0.2901	0.0551	1.38E-07	27.7199
M31536.x	rs587284	T	C	0.2273	0.0628	0.0127	7.97E-07	24.4519
M31536.x	rs6751915	T	G	0.3226	-0.0563	0.0121	3.46E-06	21.6494
M31536.x	rs6982420	T	C	0.0316	0.1648	0.036	4.71E-06	20.956
M31536.x	rs6994108	A	G	0.0236	0.2358	0.0509	3.68E-06	21.4611
M31536.x	rs7638353	A	C	0.5531	-0.1359	0.0301	6.48E-06	20.3848
M31536.x	rs9538444	T	C	0.5367	-0.0553	0.0117	2.36E-06	22.3398
M31536.x	rs9812344	A	G	0.205	0.064	0.0144	9.46E-06	19.7531
M31548.m	rs10495052	A	G	0.9683	-0.0872	0.0186	2.77E-06	21.979
M31548.m	rs10799701	A	G	0.435	0.0308	0.0066	2.69E-06	21.7778
M31548.m	rs11564552	A	G	0.4808	0.0303	0.0066	3.87E-06	21.0764
M31548.m	rs11933242	A	G	0.3129	-0.0341	0.0075	5.31E-06	20.6722
M31548.m	rs12206258	T	C	0.6865	-0.0317	0.0071	8.64E-06	19.9343
M31548.m	rs13105073	A	T	0.9776	-0.1258	0.0281	7.43E-06	20.0424
M31548.m	rs16828992	A	C	0.0715	-0.0687	0.0154	8.21E-06	19.9009
M31548.m	rs2274629	A	G	0.2873	0.0333	0.0071	3.01E-06	21.9974
M31548.m	rs2327511	A	T	0.2333	0.035	0.0075	3.44E-06	21.7778
M31548.m	rs388600	A	C	0.868	0.0448	0.0097	4E-06	21.3311
M31548.m	rs4699296	A	G	0.1693	0.0483	0.0106	4.78E-06	20.7626
M31548.m	rs4957657	T	C	0.1104	0.0482	0.0105	4.53E-06	21.0725
M31548.m	rs6879079	T	G	0.8273	-0.0388	0.0087	7.53E-06	19.8895
M31555.m	rs11125947	T	C	0.3238	0.0177	0.004	9.82E-06	19.5806
M31555.m	rs11758777	T	C	0.7686	0.0184	0.0041	7.67E-06	20.1404

M31555.m	rs12445174	A	C	0.8312	-0.0226	0.0049	3.61E-06	21.2728
M31555.m	rs13142707	T	G	0.0571	0.0406	0.0091	7.32E-06	19.9053
M31555.m	rs1431844	T	C	0.0256	0.0563	0.0119	2.3E-06	22.3832
M31555.m	rs1927938	T	C	0.154	-0.0237	0.0049	1.39E-06	23.394
M31555.m	rs2197296	A	G	0.2529	0.0207	0.0045	4.75E-06	21.16
M31555.m	rs400120	A	G	0.6474	0.0195	0.004	1.3E-06	23.7656
M31555.m	rs4674858	T	C	0.0101	0.1484	0.0329	6.61E-06	20.3459
M31555.m	rs6752425	T	C	0.0314	0.0972	0.021	3.79E-06	21.4237
M31555.m	rs7531679	T	G	0.897	0.0492	0.0111	8.96E-06	19.6465
M31555.m	rs7742715	A	G	0.356	0.0292	0.0065	6.59E-06	20.1808
M31591.m	rs10278040	A	G	0.0419	-0.3609	0.016	8.8E-113	508.784
M31591.m	rs10416477	T	G	0.0705	-0.0544	0.0122	7.75E-06	19.8828
M31591.m	rs10785657	A	G	0.1202	-0.0434	0.0098	8.96E-06	19.6122
M31591.m	rs12371604	T	C	0.7706	-0.0333	0.0073	4.96E-06	20.8086
M31591.m	rs13222543	T	C	0.0199	-0.477	0.0262	6.8E-74	331.462
M31591.m	rs1474102	T	C	0.337	-0.0339	0.0066	3.32E-07	26.3822
M31591.m	rs16958654	T	C	0.9423	0.0647	0.0135	1.68E-06	22.9689
M31591.m	rs17342647	T	C	0.12	0.054	0.012	6.8E-06	20.25
M31591.m	rs17523627	A	T	0.8853	0.0471	0.0102	3.57E-06	21.3227
M31591.m	rs17638060	T	G	0.2983	-0.0359	0.0076	2.12E-06	22.3132
M31591.m	rs182420	T	C	0.7553	0.051	0.0074	6.24E-12	47.4982
M31591.m	rs2141160	T	G	0.2465	0.0434	0.0073	2.49E-09	35.3455
M31591.m	rs2910400	T	G	0.6931	-0.0342	0.0065	1.73E-07	27.6838
M31591.m	rs349022	T	C	0.6757	0.0297	0.0066	7.81E-06	20.25
M31591.m	rs4236541	T	G	0.7287	0.0423	0.0072	5.09E-09	34.5156
M31591.m	rs4439540	A	G	0.7407	0.0574	0.0124	3.8E-06	21.4279
M31591.m	rs474229	T	G	0.6014	0.0631	0.0065	2.92E-22	94.2393
M31591.m	rs6465737	T	G	0.222	-0.05	0.0074	1.55E-11	45.6538
M31591.m	rs6595423	T	C	0.8849	0.044	0.0099	8.52E-06	19.7531
M31591.m	rs6948536	T	C	0.2462	-0.0327	0.0074	9.69E-06	19.5268
M31591.m	rs6963509	C	G	0.0423	-0.0731	0.0158	3.46E-06	21.4053
M31591.m	rs7152358	A	G	0.8887	0.0465	0.01	3.4E-06	21.6225
M31591.m	rs7778571	A	G	0.7544	0.0632	0.0073	3.5E-18	74.9529
M31591.m	rs7859457	T	C	0.3974	-0.0519	0.0111	2.93E-06	21.8619
M31591.m	rs807019	T	C	0.1496	0.0425	0.0091	3.18E-06	21.812
M31591.m	rs9676475	T	C	0.2123	0.0387	0.0081	1.72E-06	22.8272
M31591.m	rs9742609	A	G	0.9804	0.1725	0.0388	8.77E-06	19.7658
M31591.m	rs9824080	T	C	0.1602	0.0438	0.0091	1.62E-06	23.1668
M31591.m	rs9969217	A	G	0.0921	-0.0484	0.0105	4.16E-06	21.2477
M31591.m	rs9986780	A	G	0.2264	0.0335	0.0076	9.6E-06	19.4295
M31787.m	rs1015592	T	C	0.7352	-0.0404	0.0086	3.01E-06	22.0681
M31787.m	rs10185968	T	C	0.0527	0.0754	0.0168	6.83E-06	20.143
M31787.m	rs10493831	T	G	0.7644	-0.0406	0.0087	3.2E-06	21.7778
M31787.m	rs11754288	A	G	0.4322	0.0488	0.0077	2.25E-10	40.166
M31787.m	rs12458645	T	C	0.0419	0.1377	0.0279	7.92E-07	24.359
M31787.m	rs12655321	A	G	0.5227	0.0733	0.0154	2.02E-06	22.6551
M31787.m	rs17521564	A	G	0.803	-0.0472	0.0095	6.44E-07	24.6852
M31787.m	rs2125644	A	G	0.0317	-0.1928	0.0416	3.66E-06	21.4797
M31787.m	rs2553679	A	T	0.8642	0.0537	0.0117	4.25E-06	21.0657
M31787.m	rs2813524	A	G	0.6401	0.0705	0.0152	3.31E-06	21.5125
M31787.m	rs4482789	T	G	0.124	0.058	0.013	8.14E-06	19.9053
M31787.m	rs6904596	A	G	0.1218	-0.0531	0.0118	7.36E-06	20.25
M31787.m	rs6947392	T	C	0.2858	0.0486	0.0095	2.81E-07	26.1713
M31787.m	rs7776941	T	C	0.8002	0.043	0.0095	6.33E-06	20.4875
M32197.m	rs1036244	T	C	0.5441	0.0116	0.0026	7.8E-06	19.9053

M32197.m	rs12853559	A	G	0.088	0.0228	0.005	4.63E-06	20.7936
M32197.m	rs1552425	A	T	0.5806	-0.0123	0.0026	1.54E-06	22.3802
M32197.m	rs1574228	A	G	0.7061	0.0119	0.0026	6.71E-06	20.9482
M32197.m	rs17010170	C	G	0.9497	-0.0292	0.0059	6.61E-07	24.4941
M32197.m	rs17101404	A	G	0.0774	0.0245	0.0051	1.85E-06	23.0777
M32197.m	rs17389253	T	C	0.9163	-0.0214	0.0044	1.03E-06	23.655
M32197.m	rs17631990	T	C	0.0805	0.0237	0.0053	6.81E-06	19.9961
M32197.m	rs2817479	T	G	0.5278	0.0126	0.0028	6.92E-06	20.25
M32197.m	rs3749572	T	C	0.0283	0.0808	0.0172	2.62E-06	22.0681
M32197.m	rs4625783	T	C	0.4268	-0.0187	0.0026	3.9E-13	51.7293
M32197.m	rs4641779	A	C	0.1417	0.0165	0.0035	2.37E-06	22.2245
M32197.m	rs4746077	A	T	0.87	-0.0316	0.0071	9.27E-06	19.8088
M32197.m	rs4775770	A	G	0.1512	-0.0156	0.0035	8.26E-06	19.8661
M32197.m	rs6691915	A	G	0.2814	0.013	0.0027	8.95E-07	23.1824
M32197.m	rs6699507	T	C	0.818	-0.0159	0.0034	3.25E-06	21.8694
M32197.m	rs6919482	T	C	0.0207	-0.0826	0.0186	9.2E-06	19.7212
M32197.m	rs6942571	A	T	0.079	0.0214	0.0048	8.61E-06	19.8767
M32197.m	rs718377	A	G	0.3343	-0.0126	0.0026	1.29E-06	23.4852
M32197.m	rs7204324	A	T	0.9189	-0.0236	0.005	2.12E-06	22.2784
M32197.m	rs7728541	A	G	0.0167	0.0455	0.0101	7.09E-06	20.2946
M32197.m	rs8024	A	C	0.2975	0.0126	0.0026	1.58E-06	23.4852
M32197.m	rs9973135	T	C	0.0499	-0.0251	0.0056	8.32E-06	20.0896
M32198.m	rs10037469	T	G	0.968	0.0302	0.006	5.97E-07	25.3344
M32198.m	rs10735937	T	C	0.1559	-0.0147	0.0033	8E-06	19.843
M32198.m	rs10989419	T	C	0.1927	0.0125	0.0027	3.82E-06	21.4335
M32198.m	rs11599171	A	G	0.2372	0.0123	0.0026	2.79E-06	22.3802
M32198.m	rs1171614	T	C	0.2231	-0.0279	0.0028	3.38E-23	99.287
M32198.m	rs12615875	A	T	0.172	-0.0122	0.0027	6.2E-06	20.417
M32198.m	rs13184373	T	C	0.1519	-0.0124	0.0027	6.38E-06	21.0919
M32198.m	rs13257229	T	C	0.2418	0.0205	0.0046	9.88E-06	19.8606
M32198.m	rs1348656	T	C	0.7989	-0.012	0.0027	6.52E-06	19.7531
M32198.m	rs1655902	A	G	0.8197	-0.0295	0.0061	1.29E-06	23.3875
M32198.m	rs17065805	T	C	0.9378	-0.0223	0.005	8.24E-06	19.8916
M32198.m	rs2228070	T	C	0.2316	0.0118	0.0027	8.2E-06	19.1001
M32198.m	rs274567	T	C	0.3709	0.0176	0.0025	2.85E-12	49.5616
M32198.m	rs2764907	C	G	0.0855	0.0189	0.0043	9.95E-06	19.3191
M32198.m	rs4976950	A	C	0.0296	-0.068	0.0147	3.66E-06	21.3985
M32198.m	rs6583751	C	G	0.5964	-0.0117	0.0025	3.6E-06	21.9024
M32198.m	rs662138	C	G	0.8297	0.0139	0.0027	2.95E-07	26.5034
M32198.m	rs6810706	A	G	0.5523	0.0221	0.0049	5.44E-06	20.3419
M32198.m	rs7332276	T	G	0.4699	-0.0178	0.0038	3.14E-06	21.9418
M32198.m	rs7716905	A	G	0.9654	0.0316	0.006	1.53E-07	27.7378
M32198.m	rs9289719	T	G	0.811	0.012	0.0027	7.38E-06	19.7531
M32198.m	rs9348257	A	T	0.2629	0.0121	0.0026	2.74E-06	21.6583
M32315.m	rs1001972	C	G	0.8248	0.0112	0.0025	8.36E-06	20.0704
M32315.m	rs10426856	A	G	0.895	-0.0149	0.0032	4.23E-06	21.6807
M32315.m	rs1042725	T	C	0.4977	-0.0085	0.0017	7.98E-07	25
M32315.m	rs10812054	A	G	0.5108	-0.0083	0.0018	2.56E-06	21.2623
M32315.m	rs10923876	A	G	0.1487	0.0161	0.0031	2.81E-07	26.9729
M32315.m	rs10923895	T	C	0.9802	-0.0388	0.0074	1.82E-07	27.4916
M32315.m	rs1163251	T	C	0.6	0.0188	0.0017	7.05E-27	122.298
M32315.m	rs11982782	A	T	0.0817	-0.0169	0.0038	7.91E-06	19.7791
M32315.m	rs12378125	T	C	0.8196	0.0117	0.0025	3.91E-06	21.9024
M32315.m	rs12663563	C	G	0.0469	-0.0212	0.0048	8.23E-06	19.5069
M32315.m	rs12992925	A	G	0.5789	0.0084	0.0017	1.31E-06	24.4152

M32315.m	rs13033420	T	C	0.4434	0.0082	0.0017	1.92E-06	23.2664
M32315.m	rs17154498	A	C	0.1953	-0.011	0.0024	6.29E-06	21.0069
M32315.m	rs1874795	A	G	0.4303	-0.0083	0.0018	2.26E-06	21.2623
M32315.m	rs197442	A	G	0.5962	0.0104	0.0023	7.6E-06	20.4461
M32315.m	rs2269713	A	G	0.9758	0.0318	0.0065	1.14E-06	23.9347
M32315.m	rs2277251	T	G	0.4578	0.0079	0.0018	9.7E-06	19.2623
M32315.m	rs2538054	A	G	0.8443	0.0118	0.0025	2.47E-06	22.2784
M32315.m	rs2762343	A	G	0.5133	0.0083	0.0017	1.22E-06	23.8374
M32315.m	rs342563	A	G	0.471	-0.0104	0.0023	7.86E-06	20.4461
M32315.m	rs3828090	A	G	0.2682	0.0126	0.0024	9.97E-08	27.5625
M32315.m	rs403636	A	C	0.1506	-0.0116	0.0026	5.94E-06	19.9053
M32315.m	rs4130275	T	G	0.8327	0.0125	0.0025	7.99E-07	25
M32315.m	rs4241779	A	G	0.5776	0.0082	0.0017	2.83E-06	23.2664
M32315.m	rs4659015	A	C	0.484	-0.008	0.0017	4.36E-06	22.1453
M32315.m	rs4663379	A	G	0.8937	0.0142	0.0032	7.76E-06	19.6914
M32315.m	rs4947534	T	C	0.2479	-0.0183	0.0024	1.96E-14	58.1406
M32315.m	rs580333	A	G	0.4934	0.0082	0.0017	1.81E-06	23.2664
M32315.m	rs6431176	T	C	0.36	-0.0102	0.0023	8.33E-06	19.6673
M32315.m	rs6564181	A	G	0.4506	0.0078	0.0018	8.16E-06	18.7778
M32315.m	rs6740983	T	C	0.7752	-0.0117	0.0024	1.21E-06	23.7656
M32315.m	rs715	T	C	0.7111	-0.0223	0.0024	2.69E-21	86.3351
M32315.m	rs7908297	A	G	0.1133	0.0156	0.0033	1.56E-06	22.3471
M32315.m	rs841845	T	C	0.7848	0.0108	0.0024	9.21E-06	20.25
M32315.m	rs921516	A	G	0.5075	0.0079	0.0018	8.44E-06	19.2623
M32315.m	rs9373846	A	G	0.807	0.011	0.0025	8.86E-06	19.36
M32315.m	rs941348	T	C	0.2024	-0.0111	0.0025	7.05E-06	19.7136
M32315.m	rs9874508	A	G	0.4625	-0.0084	0.0017	1.42E-06	24.4152
M32319.m	rs10906914	A	C	0.7034	0.0172	0.0038	4.81E-06	20.4875
M32319.m	rs1357743	T	G	0.5585	0.0322	0.0069	2.79E-06	21.7778
M32319.m	rs6926138	T	C	0.6583	-0.0337	0.0075	6.26E-06	20.19
M32319.m	rs7342491	T	C	0.0167	-0.1192	0.0264	6.49E-06	20.3866
M32319.m	rs7429997	T	C	0.3794	-0.0161	0.0037	9.94E-06	18.9343
M32319.m	rs9863850	A	G	0.6414	-0.0177	0.004	8.55E-06	19.5806
M32322.m	rs10879778	T	C	0.3441	-0.0181	0.0035	2.59E-07	26.7437
M32322.m	rs2037458	A	G	0.6676	0.0188	0.0035	8.78E-08	28.8522
M32328.m	rs10095671	T	C	0.9066	-0.0238	0.0052	4.02E-06	20.9482
M32328.m	rs10873878	T	G	0.8609	-0.0216	0.0045	1.4E-06	23.04
M32328.m	rs11161521	T	C	0.6983	0.0703	0.0033	6.4E-100	453.819
M32328.m	rs11224138	T	C	0.1324	-0.0409	0.009	5.31E-06	20.652
M32328.m	rs1171615	T	C	0.7778	0.0246	0.0037	4.05E-11	44.2045
M32328.m	rs11753995	A	G	0.1568	-0.0229	0.0042	7.24E-08	29.7285
M32328.m	rs16852535	A	G	0.3193	0.016	0.0035	3.83E-06	20.898
M32328.m	rs170734	A	G	0.7263	-0.0154	0.0034	6.79E-06	20.5156
M32328.m	rs17097975	A	C	0.0661	-0.029	0.0056	2.53E-07	26.8176
M32328.m	rs17304141	T	G	0.9795	-0.1282	0.0121	2.46E-26	112.255
M32328.m	rs1998881	T	C	0.2091	-0.0167	0.0035	1.33E-06	22.7665
M32328.m	rs2070630	C	G	0.2948	-0.021	0.0034	6.76E-10	38.1488
M32328.m	rs272869	A	G	0.3706	0.0224	0.0033	1.4E-11	46.0753
M32328.m	rs2915420	T	C	0.1595	-0.0199	0.0042	2.1E-06	22.4495
M32328.m	rs3769423	A	C	0.0195	0.0515	0.0116	9.81E-06	19.7105
M32328.m	rs4691530	A	T	0.6951	-0.0153	0.0034	6.95E-06	20.25
M32328.m	rs5745515	T	C	0.0942	0.0428	0.0056	3.12E-14	58.4133
M32328.m	rs5745542	C	G	0.8977	-0.0314	0.0051	4.98E-10	37.907
M32328.m	rs6553778	T	C	0.2295	-0.0171	0.0035	1.18E-06	23.8702
M32328.m	rs658591	T	G	0.798	-0.019	0.004	2.03E-06	22.5625

M32328.m	rs721204	A	G	0.1115	0.0496	0.005	3E-23	98.4064
M32328.m	rs7534288	T	C	0.027	-0.0565	0.0095	2.51E-09	35.3712
M32328.m	rs7745587	A	G	0.7438	-0.0156	0.0035	6.74E-06	19.8661
M32328.m	rs7991563	A	G	0.7977	-0.0164	0.0035	2.71E-06	21.9559
M32328.m	rs883352	T	C	0.7693	0.0189	0.0042	7.55E-06	20.25
M32338.m	rs10932349	C	G	0.9212	0.0312	0.0048	7.45E-11	42.25
M32338.m	rs11111501	T	C	0.0757	-0.0214	0.0047	5.92E-06	20.7316
M32338.m	rs1123227	A	C	0.7555	0.0171	0.0039	9.8E-06	19.2249
M32338.m	rs11578	T	C	0.2919	0.0142	0.0027	1.93E-07	27.6598
M32338.m	rs11942622	T	G	0.9829	0.0712	0.0152	2.68E-06	21.9418
M32338.m	rs12328639	A	G	0.849	0.0317	0.0033	8.5E-22	92.2764
M32338.m	rs12349613	T	C	0.0551	0.0338	0.0074	5.62E-06	20.8627
M32338.m	rs13005552	T	C	0.8808	-0.0196	0.0039	6.14E-07	25.2571
M32338.m	rs13011429	C	G	0.129	-0.0292	0.0037	5.38E-15	62.282
M32338.m	rs13021675	T	C	0.1973	0.0346	0.0032	3.88E-27	116.91
M32338.m	rs13203202	T	C	0.5841	0.0107	0.0024	9.26E-06	19.8767
M32338.m	rs13401425	A	G	0.3643	-0.0215	0.0024	6.88E-19	80.2517
M32338.m	rs16845044	A	C	0.8323	0.0211	0.0032	6E-11	43.4775
M32338.m	rs16917276	A	G	0.0322	-0.0311	0.007	8.96E-06	19.739
M32338.m	rs1990797	C	G	0.1691	-0.0356	0.0033	6.96E-28	116.378
M32338.m	rs1992854	T	C	0.3742	-0.011	0.0025	6.98E-06	19.36
M32338.m	rs2287598	A	G	0.166	-0.0142	0.0032	6.62E-06	19.6914
M32338.m	rs229914	A	G	0.5846	-0.0115	0.0024	2.4E-06	22.9601
M32338.m	rs2474711	C	G	0.7239	-0.0133	0.003	9.35E-06	19.6544
M32338.m	rs2719966	A	C	0.4515	0.0216	0.0024	7.32E-19	81
M32338.m	rs3012739	A	G	0.4834	0.0116	0.0024	2.13E-06	23.3611
M32338.m	rs329319	A	G	0.4276	0.0127	0.0025	6.5E-07	25.8064
M32338.m	rs4761007	T	C	0.7354	0.0157	0.0031	4.13E-07	25.6493
M32338.m	rs491847	A	G	0.3457	-0.0113	0.0025	5.33E-06	20.4304
M32338.m	rs583776	T	C	0.6229	-0.0186	0.0042	8.19E-06	19.6122
M32338.m	rs6435568	A	G	0.4362	-0.012	0.0024	5.68E-07	25
M32338.m	rs6951820	A	G	0.4338	0.0111	0.0024	4.77E-06	21.3906
M32338.m	rs715	T	C	0.708	-0.076	0.0029	1.6E-147	686.801
M32338.m	rs7582822	A	G	0.388	0.0113	0.0024	3.84E-06	22.1684
M32338.m	rs7608258	A	G	0.3249	-0.0109	0.0025	9.98E-06	19.0096
M32338.m	rs7709785	T	C	0.148	-0.0176	0.0037	1.99E-06	22.6267
M32338.m	rs804895	T	C	0.0781	-0.0235	0.0048	8.44E-07	23.9692
M32338.m	rs8082170	T	C	0.1804	0.0153	0.0031	1.07E-06	24.359
M32338.m	rs8453	T	G	0.158	0.0144	0.0032	8.06E-06	20.25
M32338.m	rs952045	T	C	0.9494	0.0247	0.0055	7.34E-06	20.1683
M32338.m	rs983498	C	G	0.7106	-0.0197	0.0044	7.98E-06	20.046
M32339.m	rs10086950	A	C	0.3284	-0.008	0.0018	6.47E-06	19.7531
M32339.m	rs10211524	A	G	0.419	-0.0104	0.0023	7.99E-06	20.4461
M32339.m	rs11183620	A	G	0.4677	0.0098	0.0017	1.55E-08	33.2318
M32339.m	rs11652554	T	C	0.3265	0.0087	0.0018	1.52E-06	23.3611
M32339.m	rs11704957	A	C	0.0362	-0.0231	0.0049	2.85E-06	22.2245
M32339.m	rs11893991	A	G	0.4228	0.0089	0.0018	8.89E-07	24.4475
M32339.m	rs1260326	T	C	0.4051	0.0131	0.0017	5.56E-14	59.3806
M32339.m	rs1289671	A	G	0.6005	-0.0084	0.0018	1.56E-06	21.7778
M32339.m	rs1376492	A	T	0.6524	0.009	0.0018	3.59E-07	25
M32339.m	rs1440327	T	C	0.4308	-0.0082	0.0018	6E-06	20.7531
M32339.m	rs1566951	T	C	0.3943	-0.0082	0.0018	4.52E-06	20.7531
M32339.m	rs17106647	T	C	0.0296	0.0282	0.0057	8.85E-07	24.4765
M32339.m	rs17520130	T	C	0.5973	-0.0088	0.0018	6.85E-07	23.9012
M32339.m	rs1850267	T	C	0.5685	0.0079	0.0018	9.56E-06	19.2623

M32339.m	rs2064272	A	G	0.346	-0.0082	0.0018	4.49E-06	20.7531
M32339.m	rs211962	T	C	0.5372	-0.012	0.0026	5.31E-06	21.3018
M32339.m	rs2295689	A	G	0.578	-0.009	0.002	6.8E-06	20.25
M32339.m	rs2762343	A	G	0.5134	0.0077	0.0017	9.63E-06	20.5156
M32339.m	rs4317609	A	G	0.3375	-0.0079	0.0018	7.98E-06	19.2623
M32339.m	rs465152	A	G	0.5429	-0.0087	0.0017	6.45E-07	26.1903
M32339.m	rs485878	A	G	0.4238	-0.0081	0.0018	4.67E-06	20.25
M32339.m	rs4980554	A	G	0.4561	0.0082	0.0017	2.87E-06	23.2664
M32339.m	rs6053126	A	G	0.253	-0.014	0.003	3.06E-06	21.7778
M32339.m	rs6461542	A	T	0.3911	-0.0079	0.0018	6.95E-06	19.2623
M32339.m	rs6497167	T	G	0.5296	0.0079	0.0018	7.64E-06	19.2623
M32339.m	rs651158	A	G	0.6393	0.0087	0.0018	8.12E-07	23.3611
M32339.m	rs6706043	T	G	0.6182	-0.0079	0.0018	7.33E-06	19.2623
M32339.m	rs6839120	A	G	0.6771	0.0082	0.0018	4.03E-06	20.7531
M32339.m	rs6962350	T	G	0.417	0.008	0.0018	5.68E-06	19.7531
M32339.m	rs703462	C	G	0.3569	0.0093	0.0018	1.06E-07	26.6944
M32339.m	rs7191435	T	C	0.6525	0.0084	0.0018	2.53E-06	21.7778
M32339.m	rs7571558	C	G	0.7972	0.0119	0.0025	1.6E-06	22.6576
M32339.m	rs7712111	A	G	0.6383	0.0084	0.0018	1.63E-06	21.7778
M32339.m	rs7791060	A	G	0.9237	-0.0167	0.0034	6.96E-07	24.1254
M32339.m	rs785896	A	G	0.563	0.009	0.002	6.8E-06	20.25
M32339.m	rs890230	T	C	0.4592	0.0086	0.0018	1.06E-06	22.8272
M32339.m	rs9367164	A	G	0.6161	0.0077	0.0017	9.17E-06	20.5156
M32339.m	rs9582849	A	G	0.7575	0.02	0.0041	1.34E-06	23.7954
M32339.m	rs9646969	T	G	0.6076	-0.008	0.0018	5.03E-06	19.7531
M32339.m	rs9866434	A	T	0.4113	0.008	0.0018	5.55E-06	19.7531
M32339.m	rs9876174	T	C	0.8297	0.0117	0.0026	0.000007	20.25
M32346.m	rs10757367	T	C	0.8313	0.0874	0.0197	8.89E-06	19.683
M32346.m	rs1105842	A	C	0.1435	-0.048	0.0105	4.44E-06	20.898
M32346.m	rs11709500	A	G	0.2757	-0.0351	0.0079	9.03E-06	19.7406
M32346.m	rs12456097	A	G	0.7235	-0.0395	0.0086	4.75E-06	21.0959
M32346.m	rs1602290	A	G	0.1276	0.0488	0.0105	3.25E-06	21.6004
M32346.m	rs2044048	A	G	0.2224	-0.0417	0.0089	2.63E-06	21.9529
M32346.m	rs2078391	T	G	0.4558	-0.0331	0.007	2.56E-06	22.3594
M32346.m	rs4870490	A	G	0.066	0.0857	0.0145	3.14E-09	34.9322
M32346.m	rs7645550	T	C	0.4017	-0.0322	0.0071	5.84E-06	20.5681
M32346.m	rs7793797	A	T	0.7018	0.038	0.0078	1.26E-06	23.7344
M32346.m	rs7991229	T	G	0.584	-0.0316	0.0071	7.38E-06	19.8088
M32346.m	rs803294	C	G	0.234	-0.0411	0.0093	9.21E-06	19.5307
M32346.m	rs9297619	A	G	0.0979	0.1272	0.0244	1.93E-07	27.1766
M32346.m	rs9551641	T	C	0.0894	-0.1241	0.0262	2.24E-06	22.4358
M32348.m	rs10022462	T	C	0.452	-0.0117	0.0018	4.55E-11	42.25
M32348.m	rs10211524	A	G	0.4188	0.0189	0.0023	5.59E-16	67.5255
M32348.m	rs10491285	A	G	0.658	-0.0082	0.0018	3.73E-06	20.7531
M32348.m	rs10790077	T	C	0.611	-0.009	0.0018	3.15E-07	25
M32348.m	rs10898007	T	C	0.5314	0.0083	0.0018	2.3E-06	21.2623
M32348.m	rs1152592	A	G	0.6183	-0.0092	0.0018	1.57E-07	26.1235
M32348.m	rs11879267	A	G	0.17	-0.0138	0.0026	8.7E-08	28.1716
M32348.m	rs11885653	T	C	0.485	-0.0084	0.0018	1.76E-06	21.7778
M32348.m	rs11954900	T	C	0.8579	0.012	0.0026	3.6E-06	21.3018
M32348.m	rs12991423	A	G	0.9274	-0.0198	0.004	8.59E-07	24.5025
M32348.m	rs13256255	A	G	0.1668	-0.0126	0.0026	8.3E-07	23.4852
M32348.m	rs13288466	T	C	0.9192	-0.0157	0.0034	5.26E-06	21.3227
M32348.m	rs1334592	T	C	0.5205	-0.0084	0.0018	4.01E-06	21.7778
M32348.m	rs1420426	A	G	0.6202	0.0081	0.0018	4.16E-06	20.25

M32348.m	rs16826003	T	C	0.9608	-0.0239	0.005	1.72E-06	22.8484
M32348.m	rs17101315	T	C	0.973	-0.0284	0.0063	5.95E-06	20.3215
M32348.m	rs17668963	A	C	0.0383	0.0326	0.006	5.33E-08	29.5211
M32348.m	rs17763479	A	C	0.0905	0.0167	0.0034	9.81E-07	24.1254
M32348.m	rs1864774	T	C	0.586	0.0093	0.0017	1.15E-07	29.9273
M32348.m	rs192812	T	C	0.4356	0.0083	0.0018	2.89E-06	21.2623
M32348.m	rs2017320	T	C	0.1056	-0.017	0.0033	1.99E-07	26.5381
M32348.m	rs2018507	A	G	0.3629	-0.0088	0.0018	7.06E-07	23.9012
M32348.m	rs2291699	A	G	0.4835	0.0079	0.0018	7.24E-06	19.2623
M32348.m	rs2635351	A	C	0.5749	-0.0088	0.0018	5.95E-07	23.9012
M32348.m	rs2651929	T	C	0.6688	-0.008	0.0018	7.04E-06	19.7531
M32348.m	rs2853187	A	T	0.6023	-0.0078	0.0018	9.42E-06	18.7778
M32348.m	rs37379	A	G	0.0868	0.0168	0.0034	7.94E-07	24.4152
M32348.m	rs3961919	A	G	0.4308	0.0085	0.0018	1.48E-06	22.2994
M32348.m	rs405945	T	C	0.4396	-0.0082	0.0018	3.15E-06	20.7531
M32348.m	rs4315324	A	G	0.5036	0.0092	0.0018	1.39E-07	26.1235
M32348.m	rs4685337	T	C	0.5355	-0.0078	0.0018	8.64E-06	18.7778
M32348.m	rs4717210	A	G	0.082	0.019	0.004	2.03E-06	22.5625
M32348.m	rs4722590	A	C	0.4637	0.008	0.0018	5.8E-06	19.7531
M32348.m	rs4815285	A	G	0.4616	-0.0085	0.0018	1.19E-06	22.2994
M32348.m	rs597467	T	C	0.3784	0.0087	0.0018	8.06E-07	23.3611
M32348.m	rs6425496	T	C	0.544	-0.0079	0.0018	7.82E-06	19.2623
M32348.m	rs6486771	T	C	0.3983	-0.0082	0.0018	3.45E-06	20.7531
M32348.m	rs6844227	T	C	0.4886	-0.0078	0.0018	9.02E-06	18.7778
M32348.m	rs6970514	A	G	0.9601	-0.0233	0.0053	9.02E-06	19.3268
M32348.m	rs6972344	T	C	0.3962	0.0094	0.0018	1.11E-07	27.2716
M32348.m	rs7286668	A	C	0.827	-0.0113	0.0025	7.86E-06	20.4304
M32348.m	rs7310323	A	C	0.5455	0.0088	0.0017	4.07E-07	26.7958
M32348.m	rs755403	T	C	0.5969	0.0087	0.0018	9.52E-07	23.3611
M32348.m	rs758132	A	C	0.4319	0.008	0.0018	5.18E-06	19.7531
M32348.m	rs7582468	A	C	0.468	0.0079	0.0018	6.52E-06	19.2623
M32348.m	rs879138	C	G	0.3538	0.0079	0.0018	9.3E-06	19.2623
M32348.m	rs9323955	A	G	0.4396	0.0083	0.0018	2.7E-06	21.2623
M32348.m	rs9821422	T	G	0.5535	-0.0085	0.0018	1.37E-06	22.2994
M32379.m	rs12079356	A	G	0.7102	-0.0291	0.0065	8.42E-06	20.0428
M32379.m	rs17058047	A	G	0.0554	-0.044	0.0098	7.33E-06	20.1583
M32379.m	rs17177078	T	C	0.0648	-0.0454	0.008	1.65E-08	32.2056
M32379.m	rs2574985	A	G	0.2764	-0.021	0.0043	1.27E-06	23.8507
M32379.m	rs2575043	A	T	0.2748	-0.02	0.0043	3.63E-06	21.6333
M32379.m	rs2761735	T	C	0.8145	-0.024	0.0051	2.67E-06	22.1453
M32379.m	rs4694161	A	G	0.0297	0.0513	0.0114	7.19E-06	20.25
M32379.m	rs4787294	A	T	0.9278	0.075	0.008	9.64E-21	87.8906
M32379.m	rs4931472	A	G	0.3954	-0.0186	0.0041	7.3E-06	20.5806
M32379.m	rs6829935	T	C	0.2937	-0.0203	0.0043	2.57E-06	22.2872
M32379.m	rs6950034	A	G	0.0206	0.1663	0.0352	2.25E-06	22.3203
M32379.m	rs780241	A	G	0.2584	0.0209	0.0043	1.24E-06	23.6241
M32379.m	rs9284892	T	C	0.0588	-0.0382	0.0085	7.03E-06	20.1971
M32388.m	rs11126153	A	G	0.7711	-0.0205	0.0044	3.22E-06	21.7071
M32388.m	rs1539549	T	C	0.305	-0.0369	0.0079	2.72E-06	21.8172
M32388.m	rs1572603	A	G	0.13	-0.036	0.0064	1.46E-08	31.6406
M32388.m	rs16846919	T	C	0.7553	-0.0398	0.0084	2.45E-06	22.4495
M32388.m	rs2903593	C	G	0.6229	-0.0374	0.0081	4.52E-06	21.3193
M32388.m	rs602609	A	G	0.0476	-0.0391	0.0088	9.28E-06	19.7419
M32388.m	rs7499321	T	C	0.3347	0.0192	0.0037	2.14E-07	26.9277
M32393.m	rs1083801	A	G	0.7066	-0.0139	0.0024	8.78E-09	33.5434

M32393.m	rs12046664	A	G	0.1454	0.0142	0.0032	7.69E-06	19.6914
M32393.m	rs12604647	A	C	0.2439	-0.0112	0.0025	6.05E-06	20.0704
M32393.m	rs16885487	A	G	0.0207	-0.0355	0.0079	7.48E-06	20.1931
M32393.m	rs17768548	T	C	0.8282	-0.014	0.0031	8.33E-06	20.3954
M32393.m	rs2164397	C	G	0.2279	-0.0124	0.0025	8.73E-07	24.6016
M32393.m	rs3780970	T	C	0.2697	0.0113	0.0025	3.76E-06	20.4304
M32393.m	rs4661470	T	G	0.1974	-0.0152	0.0033	5.44E-06	21.2158
M32393.m	rs6094988	C	G	0.2342	0.0116	0.0025	4.46E-06	21.5296
M32393.m	rs708891	T	C	0.8739	-0.0152	0.0033	3.8E-06	21.2158
M32393.m	rs715	T	C	0.7083	0.0142	0.0029	1.07E-06	23.9762
M32393.m	rs7271447	A	G	0.3777	-0.0107	0.0024	6.67E-06	19.8767
M32393.m	rs756506	T	C	0.4018	-0.0202	0.0042	1.96E-06	23.1315
M32393.m	rs878947	A	G	0.7845	-0.0115	0.0025	5.34E-06	21.16
M32393.m	rs9894902	A	G	0.2086	0.0119	0.0025	2.35E-06	22.6576
M32405.m	rs11721811	T	C	0.6543	0.0174	0.0038	3.42E-06	20.9668
M32405.m	rs13173207	A	G	0.5657	0.0168	0.0037	7.35E-06	20.6165
M32405.m	rs13399191	C	G	0.9418	0.0369	0.0083	8.26E-06	19.765
M32405.m	rs1394678	T	C	0.2785	-0.0354	0.0038	1.7E-20	86.7839
M32405.m	rs1561277	A	C	0.2892	0.0175	0.0038	4.39E-06	21.2084
M32405.m	rs17138024	A	G	0.0597	-0.0926	0.0208	8.58E-06	19.8196
M32405.m	rs2324600	T	C	0.1179	0.0266	0.0057	3.47E-06	21.7778
M32405.m	rs2878559	T	C	0.6236	-0.0167	0.0038	8.9E-06	19.3137
M32405.m	rs377483	T	C	0.6051	-0.018	0.0038	1.6E-06	22.4377
M32405.m	rs4954419	C	G	0.7089	-0.0179	0.0038	1.89E-06	22.1891
M32405.m	rs6011040	A	G	0.3141	-0.018	0.0038	2.72E-06	22.4377
M32405.m	rs6497490	T	G	0.8821	-0.0589	0.0068	6.26E-18	75.0262
M32405.m	rs6957421	A	G	0.1573	-0.0468	0.0102	4.57E-06	21.0519
M32405.m	rs6957675	T	C	0.2559	-0.0179	0.0039	3.51E-06	21.0657
M32405.m	rs8093526	T	C	0.4585	0.0355	0.0073	1.02E-06	23.6489
M32405.m	rs900429	T	C	0.8442	0.0215	0.0048	7.22E-06	20.0629
M32405.m	rs9636231	A	G	0.2952	0.018	0.0038	2.13E-06	22.4377
M32405.m	rs9807015	T	C	0.6014	0.0324	0.0073	9.56E-06	19.699
M32412.m	rs10019724	A	G	0.0575	0.0388	0.0085	4.97E-06	20.8365
M32412.m	rs10019739	T	C	0.7419	-0.0191	0.0042	6.45E-06	20.6808
M32412.m	rs10979172	T	C	0.0218	-0.1703	0.0385	9.66E-06	19.5663
M32412.m	rs11043310	T	C	0.0238	-0.0629	0.0129	9.88E-07	23.7751
M32412.m	rs11065176	T	C	0.037	0.1404	0.011	2.42E-37	162.91
M32412.m	rs11065202	T	C	0.5822	-0.1297	0.004	1E-200	1051.38
M32412.m	rs11065208	A	G	0.0336	-0.0683	0.0106	1.17E-10	41.5174
M32412.m	rs11065390	A	G	0.0316	-0.0678	0.0115	3.23E-09	34.7587
M32412.m	rs11610379	T	C	0.0272	0.0891	0.0129	5.09E-12	47.7063
M32412.m	rs11611311	A	T	0.8579	0.0463	0.0058	1.65E-15	63.7244
M32412.m	rs1169961	A	C	0.8781	-0.0277	0.006	3.2E-06	21.3136
M32412.m	rs1171617	T	G	0.7771	0.0358	0.0047	1.61E-14	58.019
M32412.m	rs1179368	T	C	0.9551	-0.0533	0.0096	2.84E-08	30.8256
M32412.m	rs1186055	A	C	0.2939	-0.0344	0.0043	1.3E-15	64
M32412.m	rs12304179	C	G	0.8539	-0.0243	0.0052	2.4E-06	21.8376
M32412.m	rs12368199	A	G	0.1462	0.1211	0.0052	1.7E-121	542.352
M32412.m	rs12369156	A	G	0.0246	-0.0724	0.0126	8.37E-09	33.0169
M32412.m	rs12423303	A	G	0.948	0.0403	0.0086	2.61E-06	21.959
M32412.m	rs1493755	A	G	0.876	0.0291	0.0058	5.34E-07	25.1727
M32412.m	rs1653624	A	T	0.0284	-0.0754	0.0115	4.55E-11	42.988
M32412.m	rs16950755	C	G	0.0829	-0.0533	0.0072	1.66E-13	54.8011
M32412.m	rs17129837	T	C	0.0473	0.0443	0.0089	6E-07	24.7758
M32412.m	rs1718120	T	G	0.4853	-0.0263	0.0048	5.05E-08	30.0213

M32412.m	rs17762815	A	G	0.089	-0.0629	0.0127	8.08E-07	24.5298
M32412.m	rs206967	T	C	0.3497	-0.0323	0.0063	2.81E-07	26.286
M32412.m	rs2071190	A	T	0.2498	-0.0748	0.0043	8.44E-68	302.598
M32412.m	rs208294	T	C	0.4348	0.0315	0.0036	1.08E-18	76.5625
M32412.m	rs2440901	T	C	0.478	0.019	0.0035	6.46E-08	29.4694
M32412.m	rs2469211	A	T	0.8921	-0.0342	0.006	1.06E-08	32.49
M32412.m	rs2708092	A	G	0.8226	0.0408	0.0064	1.53E-10	40.6406
M32412.m	rs274567	T	C	0.3712	0.024	0.0041	6.23E-09	34.2653
M32412.m	rs278136	T	C	0.234	-0.0259	0.0043	1.96E-09	36.2796
M32412.m	rs4587804	A	G	0.6151	-0.0164	0.0035	3.21E-06	21.9559
M32412.m	rs4638383	C	G	0.1182	-0.0702	0.0059	5.54E-33	141.57
M32412.m	rs4766962	A	T	0.6544	0.0516	0.0043	1.7E-33	144
M32412.m	rs4767918	T	C	0.5895	0.0845	0.0038	2E-109	494.477
M32412.m	rs494632	T	C	0.4788	0.1072	0.0035	1E-200	938.109
M32412.m	rs6462769	T	C	0.707	0.0194	0.0043	5.69E-06	20.3548
M32412.m	rs648238	A	G	0.8822	0.027	0.0059	4.53E-06	20.9423
M32412.m	rs662138	C	G	0.831	0.0277	0.0052	8.62E-08	28.3761
M32412.m	rs6668509	A	T	0.0372	0.0464	0.0099	3.06E-06	21.9667
M32412.m	rs6696978	A	G	0.7253	0.0192	0.0043	9.33E-06	19.9373
M32412.m	rs728242	A	G	0.4989	-0.017	0.0038	6.34E-06	20.0139
M32412.m	rs7304812	A	T	0.2207	0.0819	0.0059	2.99E-43	192.692
M32412.m	rs7312642	T	G	0.0717	-0.0362	0.0076	1.76E-06	22.6877
M32412.m	rs7465584	T	C	0.5082	-0.0186	0.0041	6E-06	20.5806
M32412.m	rs7965649	T	C	0.1587	-0.0766	0.0052	7.23E-50	216.996
M32412.m	rs8006419	A	G	0.4783	-0.0158	0.0035	7.32E-06	20.3788
M32412.m	rs938107	T	C	0.3906	0.0166	0.0035	2.8E-06	22.4947
M32412.m	rs9576264	A	G	0.5497	0.016	0.0035	5.57E-06	20.898
M32412.m	rs9586807	A	T	0.1438	-0.0243	0.0052	3.37E-06	21.8376
M32412.m	rs9657933	A	C	0.9392	0.0442	0.0091	1.05E-06	23.5918
M32418.m	rs1009343	A	C	0.9622	0.1003	0.0214	2.69E-06	21.9672
M32418.m	rs10098688	A	C	0.3371	0.0267	0.006	7.3E-06	19.8025
M32418.m	rs11728793	T	C	0.7845	-0.021	0.0042	7.15E-07	25
M32418.m	rs12539054	T	C	0.2501	-0.0175	0.004	9.44E-06	19.1406
M32418.m	rs1508782	A	C	0.1332	0.0232	0.0049	2.09E-06	22.4173
M32418.m	rs35350	A	G	0.9682	0.0622	0.0132	2.33E-06	22.2041
M32418.m	rs4129516	T	C	0.7269	-0.0182	0.004	5.51E-06	20.7025
M32418.m	rs4612338	A	G	0.1596	-0.0367	0.0078	2.44E-06	22.1382
M32418.m	rs4755163	A	G	0.5331	0.0152	0.0033	3.51E-06	21.2158
M32418.m	rs603424	A	G	0.1851	-0.0368	0.0046	7.16E-16	64
M32418.m	rs6075489	A	G	0.7177	0.0185	0.0041	5.76E-06	20.3599
M32418.m	rs677603	T	C	0.2536	0.0224	0.004	2.74E-08	31.36
M32418.m	rs7100861	T	C	0.5232	-0.0268	0.0058	3.53E-06	21.3508
M32418.m	rs8088457	A	G	0.0323	0.0496	0.0103	1.59E-06	23.1894
M32418.m	rs9691574	T	C	0.0695	0.03	0.0067	8.79E-06	20.049
M32418.m	rs9888349	T	C	0.7742	-0.0192	0.004	2.02E-06	23.04
M32425.m	rs10045690	T	C	0.691	-0.027	0.006	6.8E-06	20.25
M32425.m	rs10236826	T	C	0.9564	-0.0585	0.0125	2.68E-06	21.9024
M32425.m	rs10764712	T	G	0.4349	0.0219	0.0049	9.62E-06	19.9754
M32425.m	rs11761528	T	C	0.0736	-0.0675	0.009	6.7E-14	56.25
M32425.m	rs12121588	A	G	0.2068	-0.0289	0.0058	5.43E-07	24.8279
M32425.m	rs12601482	T	C	0.8888	-0.0342	0.0075	4.43E-06	20.7936
M32425.m	rs13072891	A	G	0.628	0.0243	0.0049	7.95E-07	24.5935
M32425.m	rs13222543	T	C	0.0197	-0.1015	0.0199	3.49E-07	26.0151
M32425.m	rs1390947	A	C	0.4481	-0.0242	0.0049	6.96E-07	24.3915
M32425.m	rs17223039	A	G	0.0863	0.037	0.0083	8.92E-06	19.8723

M32425.m	rs1834260	T	C	0.7096	0.0231	0.0052	7.3E-06	19.7341
M32425.m	rs2048428	T	C	0.1358	-0.0361	0.0078	3.55E-06	21.4203
M32425.m	rs2932766	T	G	0.7067	-0.0275	0.0055	4.85E-07	25
M32425.m	rs296396	T	C	0.1646	-0.0462	0.0065	1.17E-12	50.5193
M32425.m	rs356296	A	G	0.016	-0.0882	0.0194	5.57E-06	20.6697
M32425.m	rs3802037	A	G	0.7114	-0.022	0.005	9.93E-06	19.36
M32425.m	rs628137	A	C	0.3719	-0.0221	0.0049	6.69E-06	20.3419
M32425.m	rs6900835	T	C	0.2168	-0.0273	0.0057	2.02E-06	22.9391
M32425.m	rs794614	A	G	0.7931	0.0292	0.0058	5.44E-07	25.346
M32425.m	rs878983	T	C	0.2265	-0.0261	0.0059	9.5E-06	19.5694
M32425.m	rs9316766	A	G	0.7962	-0.027	0.0058	2.82E-06	21.6706
M32425.m	rs932412	A	C	0.3987	-0.0222	0.0049	5.62E-06	20.5264
M32425.m	rs9972424	A	G	0.6603	-0.0228	0.005	4.63E-06	20.7936
M32445.x	rs10754948	T	C	0.7336	-0.034	0.0062	3.39E-08	30.0728
M32445.x	rs11177273	A	T	0.8925	0.0355	0.0079	7.29E-06	20.1931
M32445.x	rs11648287	A	G	0.1198	0.0355	0.008	8.63E-06	19.6914
M32445.x	rs12443663	C	G	0.1372	-0.0356	0.0077	4.4E-06	21.3756
M32445.x	rs12603748	T	C	0.88	-0.0389	0.0083	2.95E-06	21.9656
M32445.x	rs17071208	A	G	0.7844	-0.0312	0.0061	3.92E-07	26.1607
M32445.x	rs2026089	A	G	0.8438	-0.0314	0.0071	9.67E-06	19.5588
M32445.x	rs214785	T	C	0.8379	0.0629	0.0142	9.3E-06	19.6212
M32445.x	rs2182325	A	G	0.2818	-0.0378	0.0069	4.34E-08	30.0113
M32445.x	rs35251711	A	C	0.1112	-0.0865	0.0161	8.3E-08	28.8656
M32445.x	rs4668464	A	G	0.3316	0.0272	0.0053	2.93E-07	26.3382
M32445.x	rs4753364	A	C	0.2753	-0.0244	0.0054	7.34E-06	20.417
M32445.x	rs6517442	T	C	0.6652	0.0252	0.0056	5.94E-06	20.25
M32445.x	rs6834891	T	C	0.6263	0.0252	0.0053	2.2E-06	22.6073
M32452.m	rs10128501	A	G	0.5803	0.0108	0.0018	5.06E-09	36
M32452.m	rs10818924	T	C	0.6238	-0.0087	0.0018	2.35E-06	23.3611
M32452.m	rs11004179	T	C	0.9683	0.0274	0.0061	8.15E-06	20.1763
M32452.m	rs11057695	T	C	0.0534	0.0255	0.0057	8.01E-06	20.0139
M32452.m	rs11239545	A	T	0.2416	-0.0132	0.0026	5.22E-07	25.7751
M32452.m	rs1125232	C	G	0.1533	-0.0126	0.0027	2.44E-06	21.7778
M32452.m	rs11873891	A	G	0.6166	0.0085	0.0018	3.34E-06	22.2994
M32452.m	rs12029258	A	C	0.1421	0.0132	0.0028	1.57E-06	22.2245
M32452.m	rs12202350	T	C	0.9123	0.0191	0.0036	1.14E-07	28.1489
M32452.m	rs12356193	A	G	0.836	0.0328	0.0027	1.96E-33	147.578
M32452.m	rs1389939	C	G	0.4085	0.0098	0.0018	1.13E-07	29.642
M32452.m	rs1417985	A	G	0.9726	0.0315	0.0062	4.65E-07	25.813
M32452.m	rs1420139	C	G	0.1735	-0.0125	0.0027	3.28E-06	21.4335
M32452.m	rs1500394	T	C	0.5727	-0.0088	0.0018	1.45E-06	23.9012
M32452.m	rs1613813	A	G	0.5082	-0.0082	0.0018	8.3E-06	20.7531
M32452.m	rs17015424	C	G	0.5942	-0.022	0.0048	4.1E-06	21.0069
M32452.m	rs2164187	T	G	0.4788	0.0082	0.0018	9.33E-06	20.7531
M32452.m	rs2643926	A	G	0.4333	0.0084	0.0019	5.61E-06	19.5457
M32452.m	rs2715311	A	G	0.3884	-0.0107	0.0018	6.57E-09	35.3364
M32452.m	rs2893807	T	C	0.7325	-0.0141	0.0026	5.95E-08	29.4098
M32452.m	rs3766553	A	G	0.5153	0.0089	0.0018	1.05E-06	24.4475
M32452.m	rs3798167	T	G	0.2046	0.0121	0.0027	5.19E-06	20.0837
M32452.m	rs3798180	A	G	0.6267	-0.01	0.0018	5.8E-08	30.8642
M32452.m	rs3806851	T	C	0.4918	-0.0081	0.0018	9.85E-06	20.25
M32452.m	rs3829681	C	G	0.8259	-0.0141	0.0027	1.68E-07	27.2716
M32452.m	rs4458381	C	G	0.967	-0.0343	0.0076	6.25E-06	20.3686
M32452.m	rs6102466	T	C	0.7812	-0.0264	0.0056	2.68E-06	22.2245
M32452.m	rs6560147	T	C	0.1449	0.0126	0.0027	4.2E-06	21.7778

M32452.m	rs662138	C	G	0.8299	0.0206	0.0027	3.15E-14	58.2112
M32452.m	rs6881997	A	G	0.8282	-0.0127	0.0027	2.06E-06	22.1248
M32452.m	rs7028146	A	G	0.6217	0.0082	0.0018	7.77E-06	20.7531
M32452.m	rs7542755	A	G	0.4451	0.0085	0.0018	4.11E-06	22.2994
M32452.m	rs7545139	A	G	0.5822	0.0081	0.0018	9.14E-06	20.25
M32452.m	rs7587616	A	G	0.4743	-0.0084	0.0018	4.71E-06	21.7778
M32452.m	rs7727544	T	C	0.5553	-0.0148	0.0018	6.61E-16	67.6049
M32452.m	rs8181126	T	C	0.4188	0.0094	0.0018	2.73E-07	27.2716
M32452.m	rs842636	A	G	0.4429	-0.009	0.0018	9.72E-07	25
M32452.m	rs9399110	T	C	0.0497	0.0267	0.0053	4.29E-07	25.3788
M32452.m	rs9450092	A	G	0.886	0.037	0.0071	2.11E-07	27.1573
M32452.m	rs9503551	A	G	0.0954	-0.0164	0.0036	3.75E-06	20.7531
M32489.m	rs10011590	T	C	0.8351	0.0223	0.0049	5.43E-06	20.7118
M32489.m	rs1023115	A	G	0.5301	-0.0084	0.0018	2.68E-06	21.7778
M32489.m	rs10399665	T	C	0.5444	-0.0081	0.0018	6.59E-06	20.25
M32489.m	rs10505065	T	C	0.4005	-0.0085	0.0018	1.85E-06	22.2994
M32489.m	rs1054053	A	G	0.4028	0.0083	0.0018	3.82E-06	21.2623
M32489.m	rs10955257	T	C	0.4286	-0.008	0.0018	8.67E-06	19.7531
M32489.m	rs11895467	T	G	0.38	0.008	0.0018	9.71E-06	19.7531
M32489.m	rs11933479	T	G	0.3025	-0.0082	0.0018	6.54E-06	20.7531
M32489.m	rs12358575	T	C	0.7176	-0.0088	0.0018	1.53E-06	23.9012
M32489.m	rs1250171	T	C	0.3105	0.0082	0.0018	6.05E-06	20.7531
M32489.m	rs12582988	A	G	0.1673	-0.012	0.0026	4.13E-06	21.3018
M32489.m	rs1293768	T	G	0.3221	-0.0081	0.0018	9.31E-06	20.25
M32489.m	rs13083714	T	C	0.5761	0.0207	0.0041	3.34E-07	25.4902
M32489.m	rs1333190	T	C	0.5895	0.0091	0.0019	8.9E-07	22.9391
M32489.m	rs1360501	T	C	0.6909	-0.0084	0.0018	3.49E-06	21.7778
M32489.m	rs1413536	T	C	0.52	0.008	0.0018	7.94E-06	19.7531
M32489.m	rs1441039	A	G	0.3872	-0.0079	0.0018	8.64E-06	19.2623
M32489.m	rs16832822	T	C	0.6632	-0.0091	0.0018	5.52E-07	25.5586
M32489.m	rs17435369	T	C	0.1195	-0.0125	0.0027	3.2E-06	21.4335
M32489.m	rs17489748	A	T	0.1166	0.0143	0.0027	1.53E-07	28.0508
M32489.m	rs2103340	T	C	0.6181	-0.0081	0.0018	7.41E-06	20.25
M32489.m	rs2325358	T	C	0.7049	0.0082	0.0018	5.59E-06	20.7531
M32489.m	rs2467041	T	C	0.6501	-0.0082	0.0018	5.71E-06	20.7531
M32489.m	rs2467261	A	G	0.735	-0.009	0.002	6.8E-06	20.25
M32489.m	rs2846444	A	G	0.5854	-0.0081	0.0018	6.34E-06	20.25
M32489.m	rs331721	T	C	0.7235	0.0087	0.0018	1.85E-06	23.3611
M32489.m	rs4520471	A	G	0.5346	0.008	0.0018	8.32E-06	19.7531
M32489.m	rs4704495	T	C	0.6216	0.0082	0.0018	4.89E-06	20.7531
M32489.m	rs511319	C	G	0.3878	0.0081	0.0018	7.27E-06	20.25
M32489.m	rs6494904	A	G	0.7315	0.0087	0.0018	1.91E-06	23.3611
M32489.m	rs6498908	T	C	0.3408	-0.0082	0.0018	5.76E-06	20.7531
M32489.m	rs6711493	A	G	0.8695	-0.012	0.0026	3.76E-06	21.3018
M32489.m	rs6813957	A	G	0.6235	-0.0079	0.0018	9.14E-06	19.2623
M32489.m	rs6886152	A	C	0.5983	0.0081	0.0018	6.12E-06	20.25
M32489.m	rs6925339	A	G	0.3097	0.009	0.0018	6.67E-07	25
M32489.m	rs7617041	A	G	0.6691	0.0085	0.0018	2.9E-06	22.2994
M32489.m	rs8087127	A	G	0.5695	0.0086	0.0018	1.62E-06	22.8272
M32492.m	rs10030679	C	G	0.0419	0.0408	0.0092	8.29E-06	19.6673
M32492.m	rs10047335	A	G	0.6773	-0.0093	0.0018	2.43E-07	26.6944
M32492.m	rs10096225	A	G	0.4743	0.008	0.0018	6.74E-06	19.7531
M32492.m	rs10153405	A	G	0.5344	-0.0078	0.0018	9.07E-06	18.7778
M32492.m	rs10435813	A	G	0.8044	0.0116	0.0026	6.8E-06	19.9053
M32492.m	rs10514491	T	C	0.6751	0.0103	0.0018	1.59E-08	32.7438

M32492.m	rs10762497	A	C	0.3563	0.0084	0.0018	2.25E-06	21.7778
M32492.m	rs11102898	A	G	0.4032	-0.0083	0.0018	2.68E-06	21.2623
M32492.m	rs11758990	A	C	0.8954	-0.0147	0.0033	7.35E-06	19.843
M32492.m	rs11815675	A	G	0.1429	-0.0124	0.0026	1.93E-06	22.7456
M32492.m	rs12026398	T	C	0.8948	-0.0143	0.0032	8.89E-06	19.9697
M32492.m	rs12478033	T	C	0.6526	-0.0086	0.0018	2.79E-06	22.8272
M32492.m	rs12588574	A	G	0.2168	0.0113	0.0025	6.12E-06	20.4304
M32492.m	rs13083714	T	C	0.5761	0.0178	0.0039	4.1E-06	20.831
M32492.m	rs13242418	A	G	0.337	-0.0079	0.0018	8.31E-06	19.2623
M32492.m	rs13254365	T	C	0.1852	0.0115	0.0025	5.57E-06	21.16
M32492.m	rs1373469	T	C	0.5235	0.0081	0.0018	4.85E-06	20.25
M32492.m	rs1576171	A	T	0.984	-0.045	0.01	6.8E-06	20.25
M32492.m	rs16844437	A	G	0.9276	-0.0191	0.0041	2.72E-06	21.702
M32492.m	rs17287933	T	C	0.4694	0.0086	0.0018	1.48E-06	22.8272
M32492.m	rs2049791	A	G	0.7537	-0.0115	0.0025	3.3E-06	21.16
M32492.m	rs2239855	T	C	0.986	-0.047	0.01	2.6E-06	22.09
M32492.m	rs2414622	A	G	0.6434	-0.0084	0.0018	2.93E-06	21.7778
M32492.m	rs2704668	T	C	0.6202	-0.0087	0.0018	8.77E-07	23.3611
M32492.m	rs2706095	A	G	0.3359	-0.0086	0.0018	2.38E-06	22.8272
M32492.m	rs351755	A	G	0.5614	-0.0082	0.0018	3.53E-06	20.7531
M32492.m	rs3795579	T	G	0.3479	0.0091	0.0018	3.69E-07	25.5586
M32492.m	rs4704495	T	C	0.6216	0.0081	0.0018	5.41E-06	20.25
M32492.m	rs4834846	A	G	0.5503	0.0089	0.0018	4.7E-07	24.4475
M32492.m	rs4858696	T	C	0.5733	-0.0084	0.0018	2.15E-06	21.7778
M32492.m	rs4898707	T	C	0.4396	-0.0082	0.0018	4.35E-06	20.7531
M32492.m	rs621340	T	G	0.1298	-0.0136	0.0027	4.7E-07	25.3717
M32492.m	rs6454349	T	C	0.9192	0.0153	0.0035	9.32E-06	19.1094
M32492.m	rs6860349	A	G	0.4109	-0.0092	0.0018	2.57E-07	26.1235
M32492.m	rs6913428	T	C	0.8901	0.015	0.0034	9.11E-06	19.4637
M32492.m	rs7136574	T	C	0.4342	0.0087	0.0018	8.27E-07	23.3611
M32492.m	rs7172565	T	C	0.0289	0.0705	0.0153	3.93E-06	21.2322
M32492.m	rs7200919	A	G	0.4101	-0.0088	0.0018	7.75E-07	23.9012
M32492.m	rs7669416	A	G	0.549	-0.009	0.002	6.8E-06	20.25
M32492.m	rs7712191	T	G	0.6607	0.0081	0.0018	7.1E-06	20.25
M32492.m	rs8059670	T	C	0.4437	-0.0097	0.0018	3.72E-08	29.0401
M32492.m	rs9384386	T	C	0.3966	0.0083	0.0018	3.26E-06	21.2623
M32492.m	rs9403095	A	C	0.3305	-0.0088	0.0018	9.32E-07	23.9012
M32492.m	rs940597	T	C	0.3479	-0.0084	0.0018	3E-06	21.7778
M32492.m	rs9852606	C	G	0.377	-0.0082	0.0018	3.97E-06	20.7531
M32492.m	rs9924102	A	G	0.6426	-0.0082	0.0018	4.25E-06	20.7531
M32497.m	rs10837883	T	G	0.6036	-0.0122	0.0026	4.08E-06	22.0178
M32497.m	rs11213924	A	G	0.0489	0.0298	0.0067	7.82E-06	19.7826
M32497.m	rs11265023	A	G	0.4733	0.0125	0.0026	1.85E-06	23.1139
M32497.m	rs11680604	C	G	0.7095	0.0168	0.0033	3.48E-07	25.9174
M32497.m	rs11704875	T	C	0.1373	-0.0186	0.0042	8.55E-06	19.6122
M32497.m	rs12360462	A	G	0.5969	0.0118	0.0026	5.69E-06	20.5976
M32497.m	rs12435939	T	C	0.0335	-0.0686	0.0155	9.8E-06	19.5878
M32497.m	rs12806225	A	G	0.024	-0.1127	0.0251	7.05E-06	20.1605
M32497.m	rs13373881	T	C	0.9016	-0.0232	0.0045	2.17E-07	26.5798
M32497.m	rs1558852	A	G	0.7951	0.0154	0.0034	7.03E-06	20.5156
M32497.m	rs17103030	A	C	0.9649	-0.0347	0.0078	8.5E-06	19.7911
M32497.m	rs17103322	T	C	0.9443	-0.0294	0.0067	9.77E-06	19.2551
M32497.m	rs17685	A	G	0.2781	-0.0168	0.0034	6.44E-07	24.4152
M32497.m	rs1863824	A	G	0.613	0.0117	0.0026	9.64E-06	20.25
M32497.m	rs2042807	T	C	0.573	-0.0237	0.0052	5.4E-06	20.7726

M32497.m	rs2055498	T	C	0.0472	0.03	0.0066	6.31E-06	20.6612
M32497.m	rs2373319	A	T	0.7383	-0.015	0.0033	5.87E-06	20.6612
M32497.m	rs2494248	T	C	0.6709	-0.0251	0.0032	1.19E-14	61.5244
M32497.m	rs2968858	T	C	0.5283	-0.0118	0.0026	7.25E-06	20.5976
M32497.m	rs3828128	A	G	0.2536	-0.0161	0.0033	1.35E-06	23.8026
M32497.m	rs590582	C	G	0.9437	0.03	0.0061	7.95E-07	24.187
M32497.m	rs6062362	A	G	0.5705	0.0122	0.0026	3.38E-06	22.0178
M32497.m	rs6077678	A	G	0.848	-0.0228	0.0041	3.38E-08	30.9244
M32497.m	rs6801666	A	G	0.4209	-0.0122	0.0026	2.89E-06	22.0178
M32497.m	rs7013605	T	C	0.1199	0.02	0.0042	2.42E-06	22.6757
M32497.m	rs7131362	A	G	0.4077	0.0119	0.0027	9.12E-06	19.4252
M32497.m	rs7468101	T	C	0.9497	0.0299	0.0067	7.9E-06	19.9156
M32497.m	rs7556052	A	T	0.6721	-0.0346	0.0034	5.5E-24	103.561
M32497.m	rs780093	T	C	0.395	-0.0132	0.0026	4.29E-07	25.7751
M32497.m	rs8054389	T	C	0.493	-0.0118	0.0026	6.42E-06	20.5976
M32497.m	rs838532	T	C	0.9416	-0.0293	0.0065	6.89E-06	20.3193
M32497.m	rs9333029	A	G	0.8669	-0.0695	0.0042	1.52E-61	273.824
M32497.m	rs998531	A	G	0.8796	0.02	0.0042	1.89E-06	22.6757
M32504.m	rs10788823	T	G	0.1886	0.0419	0.0086	1.13E-06	23.7373
M32504.m	rs1201686	C	G	0.0467	-0.0422	0.0088	1.6E-06	22.9964
M32504.m	rs12999714	A	T	0.6299	-0.0153	0.0034	8.38E-06	20.25
M32504.m	rs16839086	A	C	0.2301	0.0348	0.0076	5.42E-06	20.9668
M32504.m	rs16936904	A	G	0.954	-0.0709	0.0154	4.09E-06	21.1959
M32504.m	rs16972160	T	C	0.9515	0.0369	0.0083	8.72E-06	19.765
M32504.m	rs174538	A	G	0.3025	-0.0264	0.0035	8.9E-14	56.8947
M32504.m	rs2114655	A	G	0.0708	0.0299	0.0068	9.82E-06	19.3341
M32504.m	rs3793900	A	C	0.6356	-0.0169	0.0035	1.12E-06	23.3151
M32504.m	rs6868629	C	G	0.0728	0.0392	0.0088	8.71E-06	19.843
M32504.m	rs6957801	A	G	0.0217	0.0612	0.0138	9.04E-06	19.6673
M32518.m	rs10084901	A	T	0.5872	0.008	0.0018	7.46E-06	19.7531
M32518.m	rs10468017	T	C	0.289	0.0108	0.0018	3.45E-09	36
M32518.m	rs10924121	A	G	0.8146	-0.0084	0.0019	7.07E-06	19.5457
M32518.m	rs11614616	A	C	0.7392	-0.0083	0.0018	5.84E-06	21.2623
M32518.m	rs11885422	A	T	0.2326	0.0084	0.0018	4.34E-06	21.7778
M32518.m	rs12577969	A	G	0.1103	-0.0127	0.0027	2.85E-06	22.1248
M32518.m	rs13262434	T	G	0.308	-0.008	0.0018	9.95E-06	19.7531
M32518.m	rs13420201	A	C	0.7652	-0.0084	0.0018	5.55E-06	21.7778
M32518.m	rs1388331	A	G	0.913	0.014	0.003	3.06E-06	21.7778
M32518.m	rs16916075	T	G	0.806	0.0089	0.0019	2.69E-06	21.9418
M32518.m	rs17579748	A	G	0.9713	0.0292	0.0063	3.87E-06	21.4825
M32518.m	rs1771823	A	G	0.706	0.0092	0.0018	4.94E-07	26.1235
M32518.m	rs2053595	A	G	0.312	0.0086	0.0018	2.98E-06	22.8272
M32518.m	rs2295308	A	C	0.9608	0.0209	0.0045	3.07E-06	21.5709
M32518.m	rs2426412	A	G	0.8152	-0.0084	0.0019	6.7E-06	19.5457
M32518.m	rs2431704	A	C	0.8029	-0.009	0.0019	1.04E-06	22.4377
M32518.m	rs2434532	A	G	0.1919	-0.0095	0.0019	2.84E-07	25
M32518.m	rs2831549	T	C	0.7077	-0.009	0.0018	4.9E-07	25
M32518.m	rs2935461	A	G	0.81	-0.011	0.002	3.8E-08	30.25
M32518.m	rs2991721	A	G	0.3833	-0.0085	0.0018	3.43E-06	22.2994
M32518.m	rs345513	A	G	0.6132	-0.0084	0.0018	2.86E-06	21.7778
M32518.m	rs35441	T	C	0.3871	0.0085	0.0018	2.12E-06	22.2994
M32518.m	rs3784934	A	C	0.0856	0.0126	0.0028	5.64E-06	20.25
M32518.m	rs3799488	T	C	0.8844	-0.0125	0.0028	7.31E-06	19.9298
M32518.m	rs409950	A	C	0.1931	-0.0088	0.0019	4.89E-06	21.4515
M32518.m	rs4355401	A	C	0.4341	0.008	0.0018	8.93E-06	19.7531

M32518.m	rs4516432	T	C	0.7795	0.0082	0.0018	7.61E-06	20.7531
M32518.m	rs4724070	T	C	0.2039	0.0084	0.0018	4.96E-06	21.7778
M32518.m	rs4814243	A	G	0.8087	-0.0091	0.0019	1E-06	22.9391
M32518.m	rs4841120	T	G	0.714	0.0088	0.0018	1.47E-06	23.9012
M32518.m	rs4904578	T	C	0.3808	0.0082	0.0018	6.29E-06	20.7531
M32518.m	rs4936882	T	G	0.2446	0.0083	0.0018	5.44E-06	21.2623
M32518.m	rs6880807	T	G	0.633	-0.008	0.0018	7.68E-06	19.7531
M32518.m	rs6946189	A	G	0.2499	-0.0088	0.0018	1.52E-06	23.9012
M32518.m	rs696836	T	G	0.8989	-0.012	0.0027	8.28E-06	19.7531
M32518.m	rs7349641	T	C	0.8878	0.0127	0.0027	2.72E-06	22.1248
M32518.m	rs7959422	T	C	0.888	0.0128	0.0027	2.04E-06	22.4746
M32518.m	rs8033940	A	G	0.274	0.0104	0.0018	1.19E-08	33.3827
M32518.m	rs9322458	A	T	0.8754	-0.0118	0.0026	7.6E-06	20.5976
M32518.m	rs9367334	A	C	0.1437	0.0119	0.0027	7.63E-06	19.4252
M32518.m	rs9471920	T	G	0.0252	0.026	0.0059	9.33E-06	19.4197
M32518.m	rs9622216	C	G	0.8978	0.0125	0.0028	6.88E-06	19.9298
M32549.m	rs10844144	A	C	0.8691	-0.0464	0.0097	1.84E-06	22.8819
M32549.m	rs11188148	A	G	0.8756	-0.066	0.01	3.87E-11	43.56
M32549.m	rs1165196	A	G	0.549	-0.0409	0.0066	4.8E-10	38.4024
M32549.m	rs12248434	T	C	0.8404	0.0467	0.0089	1.53E-07	27.533
M32549.m	rs2071426	T	C	0.7107	0.0547	0.0073	6.87E-14	56.1473
M32549.m	rs2187662	A	G	0.0332	0.0939	0.019	7.61E-07	24.4244
M32549.m	rs2199680	A	T	0.8322	0.0526	0.0088	2.18E-09	35.7278
M32549.m	rs2272861	A	G	0.7054	-0.0342	0.0074	3.45E-06	21.3594
M32549.m	rs3736083	T	C	0.5544	0.0304	0.0066	3.98E-06	21.2158
M32549.m	rs4726900	T	G	0.6601	0.031	0.0067	3.89E-06	21.4079
M32549.m	rs7311964	T	G	0.7481	-0.0357	0.0075	1.79E-06	22.6576
M32553.m	rs10963621	A	T	0.8663	-0.0324	0.0073	9.78E-06	19.699
M32553.m	rs11133365	T	C	0.9007	-0.0715	0.016	7.62E-06	19.9697
M32553.m	rs11779285	A	T	0.3845	0.0231	0.0051	6.5E-06	20.5156
M32553.m	rs13195040	A	G	0.9137	-0.0373	0.008	3.59E-06	21.7389
M32553.m	rs13198474	A	G	0.0945	0.042	0.0081	2.24E-07	26.8861
M32553.m	rs13211507	T	C	0.8862	-0.0327	0.0073	6.87E-06	20.0655
M32553.m	rs1452040	A	C	0.7973	-0.0539	0.0116	3.68E-06	21.5904
M32553.m	rs16901784	A	C	0.1157	0.0365	0.0071	2.64E-07	26.4283
M32553.m	rs17628016	A	G	0.1561	0.0316	0.0069	4.12E-06	20.9737
M32553.m	rs17642590	C	G	0.8943	-0.0337	0.0072	2.67E-06	21.9076
M32553.m	rs3010305	T	C	0.1318	0.0327	0.0071	4.44E-06	21.2119
M32553.m	rs4324798	A	G	0.1136	0.0354	0.0073	1.08E-06	23.5159
M32553.m	rs6916131	T	G	0.9697	-0.0631	0.0137	4.45E-06	21.2138
M32553.m	rs7047770	A	G	0.6563	-0.024	0.0052	4.49E-06	21.3018
M32553.m	rs7118058	T	G	0.0603	0.0474	0.0106	8.13E-06	19.9961
M32553.m	rs7714057	T	C	0.0685	0.0456	0.0095	1.75E-06	23.04
M32553.m	rs9682960	T	C	0.8843	-0.0388	0.0083	3.36E-06	21.8528
M32557.m	rs10503463	C	G	0.8052	-0.0338	0.0072	3.14E-06	22.0378
M32557.m	rs12804160	C	G	0.1235	0.0376	0.0082	5.13E-06	21.0256
M32557.m	rs12920537	A	G	0.7386	0.0308	0.0067	4.37E-06	21.1325
M32557.m	rs1344215	A	G	0.4834	0.0418	0.0088	1.76E-06	22.5625
M32557.m	rs1447149	A	G	0.2486	-0.0311	0.0064	1.08E-06	23.6135
M32557.m	rs16957268	C	G	0.9068	-0.0443	0.0091	1.09E-06	23.6987
M32557.m	rs1740074	C	G	0.9615	-0.0697	0.0156	8.29E-06	19.9626
M32557.m	rs17578989	T	C	0.6347	0.0259	0.0056	3.1E-06	21.3906
M32557.m	rs2834622	T	C	0.2733	0.0302	0.0064	2.34E-06	22.2666
M32557.m	rs4549297	A	G	0.8104	-0.0327	0.0073	7.83E-06	20.0655
M32557.m	rs6692597	A	C	0.0597	-0.0515	0.0111	3.24E-06	21.5263

M32557.m	rs8049811	T	C	0.9741	0.0738	0.0166	8.71E-06	19.765
M32557.m	rs8071320	T	G	0.2306	-0.0294	0.0064	4.16E-06	21.1025
M32557.m	rs883118	A	G	0.6702	0.0254	0.0055	4.22E-06	21.3276
M32560.m	rs11034982	A	G	0.9783	-0.1561	0.0336	3.51E-06	21.5838
M32560.m	rs11053060	T	C	0.0474	0.1636	0.0358	4.84E-06	20.8834
M32560.m	rs11951976	A	G	0.352	-0.0493	0.0106	3.15E-06	21.6313
M32560.m	rs3219080	T	C	0.0219	0.2532	0.0571	9.19E-06	19.6632
M32560.m	rs4151405	T	G	0.9851	-0.2836	0.0639	9.05E-06	19.6975
M32560.m	rs4308236	A	G	0.2636	0.0417	0.0094	9.03E-06	19.6796
M32560.m	rs4630914	T	C	0.1001	0.062	0.0136	4.9E-06	20.7829
M32560.m	rs4650516	T	C	0.5865	-0.0448	0.009	5.75E-07	24.7783
M32560.m	rs4687396	A	C	0.9784	-0.1489	0.0292	3.34E-07	26.003
M32560.m	rs4742370	C	G	0.3392	-0.0465	0.01	3.24E-06	21.6225
M32560.m	rs7103589	T	G	0.6053	0.0388	0.0086	5.72E-06	20.3548
M32560.m	rs899236	A	G	0.2189	-0.0497	0.0107	3.6E-06	21.5747
M32560.m	rs9901192	A	G	0.0959	0.1267	0.0267	2E-06	22.518
M32564.m	rs10044059	A	G	0.0711	0.0646	0.0143	6.59E-06	20.4076
M32564.m	rs10183528	C	G	0.9442	0.0896	0.0178	5.09E-07	25.3382
M32564.m	rs10508243	A	G	0.1246	0.0519	0.0109	1.92E-06	22.6716
M32564.m	rs1125853	C	G	0.1705	0.045	0.0095	2.08E-06	22.4377
M32564.m	rs12820694	A	G	0.2682	-0.0371	0.0084	9.32E-06	19.5069
M32564.m	rs13129658	C	G	0.8162	0.0437	0.0095	4.02E-06	21.16
M32564.m	rs1341146	T	G	0.734	-0.0406	0.0085	1.66E-06	22.8147
M32564.m	rs1853207	T	C	0.0598	0.1085	0.0152	9.58E-13	50.9533
M32564.m	rs1884646	C	G	0.1687	-0.087	0.0188	3.81E-06	21.4152
M32564.m	rs1900804	T	C	0.2616	0.0377	0.0085	8.07E-06	19.6718
M32564.m	rs2051674	T	C	0.8325	0.0434	0.0096	5.89E-06	20.4379
M32564.m	rs2679891	T	G	0.0672	0.0719	0.0158	5.05E-06	20.7083
M32564.m	rs2722639	T	C	0.5475	-0.0318	0.0069	4.43E-06	21.2401
M32564.m	rs2807872	T	C	0.1863	-0.0833	0.0176	2.11E-06	22.4009
M32564.m	rs2884692	T	C	0.3823	-0.0349	0.0077	6.34E-06	20.5433
M32564.m	rs3846714	A	G	0.065	0.0672	0.0149	6.46E-06	20.3407
M32564.m	rs4750519	T	G	0.4178	-0.0324	0.007	4.1E-06	21.4237
M32564.m	rs603677	A	C	0.9724	0.12	0.0258	3.35E-06	21.6333
M32564.m	rs644396	T	C	0.5465	-0.0327	0.0071	4.45E-06	21.2119
M32564.m	rs751903	A	G	0.0733	0.0647	0.0143	6.22E-06	20.4709
M32578.m	rs10111779	A	C	0.7263	-0.0462	0.0103	8E-06	20.1191
M32578.m	rs1043147	A	G	0.8032	0.0239	0.0054	8.25E-06	19.5888
M32578.m	rs1171614	T	C	0.2233	-0.0423	0.0056	6.68E-14	57.0564
M32578.m	rs1205497	A	G	0.6371	-0.0205	0.0045	6.09E-06	20.7531
M32578.m	rs186053	T	G	0.704	-0.0237	0.0052	6.01E-06	20.7726
M32578.m	rs1884917	C	G	0.2335	-0.0256	0.0053	1.51E-06	23.3307
M32578.m	rs312994	A	G	0.5193	-0.0198	0.0044	8.77E-06	20.25
M32578.m	rs3798167	T	G	0.2049	0.0344	0.0053	1.1E-10	42.1274
M32578.m	rs4365292	A	C	0.1924	0.0257	0.0053	1.51E-06	23.5133
M32578.m	rs4854707	T	G	0.5813	-0.0418	0.0094	9.75E-06	19.7741
M32578.m	rs520906	A	G	0.6353	0.0211	0.0045	2.23E-06	21.9857
M32578.m	rs648253	A	G	0.5044	-0.0296	0.0044	2.05E-11	45.2562
M32578.m	rs662138	C	G	0.8307	0.0633	0.0062	9.38E-25	104.238
M32578.m	rs6939741	A	G	0.5419	0.0252	0.0045	1.87E-08	31.36
M32578.m	rs697387	T	C	0.299	-0.023	0.005	4.23E-06	21.16
M32578.m	rs715	T	C	0.7165	0.0272	0.0052	1.81E-07	27.3609
M32578.m	rs759400	T	C	0.8126	0.0246	0.0054	5.26E-06	20.7531
M32578.m	rs7866	T	C	0.0443	-0.1033	0.023	6.95E-06	20.1718
M32578.m	rs9318571	A	G	0.5614	-0.0199	0.0045	7.67E-06	19.556

M32578.m	rs9457843	T	C	0.1187	0.0404	0.007	6.84E-09	33.3094
M32586.m	rs10216545	A	G	0.4695	-0.0452	0.0095	1.93E-06	22.6376
M32586.m	rs10796993	T	C	0.865	0.0374	0.0082	4.81E-06	20.8025
M32586.m	rs10941555	A	G	0.4581	-0.0258	0.0058	9.63E-06	19.7872
M32586.m	rs11736580	T	C	0.7281	-0.0332	0.0064	2.19E-07	26.9102
M32586.m	rs17863762	A	G	0.0291	-0.0802	0.0174	3.84E-06	21.2447
M32586.m	rs1996490	A	G	0.4179	-0.0272	0.0057	2.08E-06	22.7713
M32586.m	rs2219067	C	G	0.761	0.0355	0.0065	5.91E-08	29.8284
M32586.m	rs28900371	A	G	0.0351	0.1052	0.0154	7.07E-12	46.6649
M32586.m	rs3796092	A	C	0.0984	0.0545	0.0094	7.09E-09	33.6153
M32586.m	rs4147547	T	G	0.4337	-0.0255	0.0057	6.87E-06	20.0139
M32586.m	rs4149000	T	C	0.139	0.0402	0.0084	1.72E-06	22.9031
M32586.m	rs6723936	A	G	0.641	0.0474	0.0058	2.41E-16	66.7883
M32586.m	rs730673	C	G	0.6439	-0.0309	0.0057	4.78E-08	29.3878
M32586.m	rs7608713	A	G	0.2383	-0.0447	0.0066	1.28E-11	45.8698
M32586.m	rs7855272	A	G	0.2071	-0.0359	0.0074	1.14E-06	23.5356
M32586.m	rs838718	A	G	0.4862	0.0337	0.0056	1.73E-09	36.2146
M32586.m	rs887829	T	C	0.3295	0.128	0.0062	3.76E-95	426.223
M32586.m	rs917664	A	T	0.3512	-0.0461	0.0096	1.47E-06	23.06
M32587.m	rs11050696	A	G	0.1024	0.0245	0.0055	9.35E-06	19.843
M32587.m	rs11572082	C	G	0.876	0.0284	0.0055	2.16E-07	26.6631
M32587.m	rs12258052	A	G	0.3022	0.0171	0.0037	3.14E-06	21.3594
M32587.m	rs1284443	T	C	0.2702	-0.0171	0.0037	4.97E-06	21.3594
M32587.m	rs1624064	T	C	0.5678	-0.0164	0.0036	6.36E-06	20.7531
M32587.m	rs17450934	A	G	0.0875	-0.0365	0.0077	2.21E-06	22.4701
M32587.m	rs17685	A	G	0.2774	-0.0196	0.0038	1.85E-07	26.6039
M32587.m	rs1811763	A	G	0.144	-0.025	0.0054	3.53E-06	21.4335
M32587.m	rs1934955	A	G	0.713	-0.0301	0.0037	3.33E-16	66.1804
M32587.m	rs2241480	T	C	0.2872	0.0198	0.0037	7.72E-08	28.637
M32587.m	rs4304697	A	G	0.0604	0.0382	0.0072	1.34E-07	28.1489
M32587.m	rs4447076	A	G	0.6152	-0.0162	0.0036	8.58E-06	20.25
M32587.m	rs568167	A	G	0.2282	-0.0196	0.0044	9.38E-06	19.843
M32587.m	rs6490461	A	C	0.0491	0.0401	0.0084	1.6E-06	22.7893
M32593.m	rs10026261	A	T	0.0221	-0.1345	0.0304	9.73E-06	19.5748
M32593.m	rs13417480	T	C	0.8097	-0.0266	0.0058	5.43E-06	21.0333
M32593.m	rs1448663	A	G	0.1756	0.0334	0.0065	3.47E-07	26.4038
M32593.m	rs17148983	C	G	0.0869	0.0361	0.0077	2.52E-06	21.9803
M32593.m	rs1978915	A	C	0.506	0.0198	0.0043	5.48E-06	21.2028
M32593.m	rs2093237	T	C	0.2815	0.0228	0.005	5.93E-06	20.7936
M32593.m	rs2393983	C	G	0.0264	-0.0641	0.0145	9.4E-06	19.5425
M32593.m	rs2698512	A	G	0.0391	-0.0539	0.0117	4.09E-06	21.223
M32593.m	rs2977258	A	G	0.3036	0.0244	0.0049	5.39E-07	24.7963
M32593.m	rs2985714	A	G	0.0943	-0.0342	0.0077	7.65E-06	19.7274
M32593.m	rs7783170	T	C	0.7251	-0.0229	0.0051	7.96E-06	20.1619
M32593.m	rs7942018	T	C	0.4068	-0.0217	0.0048	5.96E-06	20.4379
M32616.m	rs10836109	C	G	0.0682	0.086	0.0181	1.94E-06	22.5756
M32616.m	rs1322374	A	G	0.7628	-0.0455	0.0103	9.72E-06	19.5141
M32616.m	rs1823803	T	C	0.4614	-0.0427	0.0088	1.22E-06	23.5446
M32616.m	rs2104340	C	G	0.2526	0.0617	0.013	2.16E-06	22.526
M32616.m	rs2402228	A	T	0.1901	-0.051	0.0112	5.27E-06	20.735
M32616.m	rs4149056	T	C	0.8373	-0.0537	0.0116	4E-06	21.4305
M32616.m	rs500421	C	G	0.9177	0.0736	0.0165	8.23E-06	19.897
M32616.m	rs6453786	T	G	0.2993	-0.0471	0.0102	3.87E-06	21.3227
M32616.m	rs6879662	T	C	0.4073	-0.0385	0.0087	9.27E-06	19.5832
M32616.m	rs7847689	T	C	0.7003	0.0438	0.0094	3.44E-06	21.7116

M32616.m	rs7997078	A	G	0.388	-0.054	0.0121	8.52E-06	19.9167
M32616.m	rs8130944	A	G	0.3688	-0.0439	0.0098	7.84E-06	20.0667
M32632.m	rs10189004	T	C	0.0531	-0.0703	0.0152	3.66E-06	21.3906
M32632.m	rs10263411	A	T	0.8572	0.0199	0.0043	3.58E-06	21.4175
M32632.m	rs10409299	A	G	0.8011	-0.0162	0.0035	3.97E-06	21.4237
M32632.m	rs10882310	A	G	0.5268	-0.0126	0.0027	2.77E-06	21.7778
M32632.m	rs11256613	T	G	0.8242	0.0347	0.0078	9.27E-06	19.7911
M32632.m	rs11629133	T	C	0.1656	-0.0201	0.0043	3.09E-06	21.8502
M32632.m	rs12748013	A	G	0.0816	-0.0277	0.0061	5.62E-06	20.6205
M32632.m	rs1328411	T	C	0.1803	0.0158	0.0036	9.75E-06	19.2623
M32632.m	rs1474920	T	G	0.1861	-0.0174	0.0036	1.46E-06	23.3611
M32632.m	rs1511782	A	C	0.543	-0.0137	0.0027	3.74E-07	25.7462
M32632.m	rs17236463	A	T	0.1577	0.0189	0.0042	8.26E-06	20.25
M32632.m	rs173365	A	G	0.4432	-0.012	0.0027	8.19E-06	19.7531
M32632.m	rs17382842	A	G	0.9864	0.1202	0.0254	2.28E-06	22.3945
M32632.m	rs17750980	A	G	0.2501	-0.0162	0.0036	6.38E-06	20.25
M32632.m	rs1809148	A	G	0.136	-0.0262	0.0043	9.51E-10	37.1249
M32632.m	rs2416366	A	C	0.4649	0.013	0.0027	1.17E-06	23.1824
M32632.m	rs2449529	A	G	0.5572	-0.0127	0.0027	2.76E-06	22.1248
M32632.m	rs2595191	T	C	0.8884	0.0204	0.0044	2.76E-06	21.4959
M32632.m	rs2776859	T	C	0.5308	0.0124	0.0027	5.68E-06	21.0919
M32632.m	rs2910745	C	G	0.0111	-0.136	0.03	5.7E-06	20.5511
M32632.m	rs2979269	A	G	0.4706	-0.0122	0.0027	4.59E-06	20.417
M32632.m	rs3761097	T	C	0.053	0.057	0.008	1.04E-12	50.7656
M32632.m	rs4327428	A	C	0.1117	-0.031	0.0044	2.81E-12	49.6384
M32632.m	rs4780050	T	C	0.8238	0.0162	0.0036	5.89E-06	20.25
M32632.m	rs4834604	T	C	0.5456	-0.0123	0.0027	4.92E-06	20.7531
M32632.m	rs653349	A	G	0.2307	-0.0312	0.0071	9.51E-06	19.3105
M32632.m	rs6916798	T	C	0.9454	0.0608	0.0136	7.41E-06	19.9862
M32632.m	rs7541494	T	C	0.8786	-0.0201	0.0043	3.45E-06	21.8502
M32632.m	rs9322744	T	C	0.3958	0.0128	0.0027	1.91E-06	22.4746
M32634.m	rs1005075	A	G	0.4668	-0.0081	0.0018	5.84E-06	20.25
M32634.m	rs10129722	A	G	0.3124	-0.0082	0.0018	5.95E-06	20.7531
M32634.m	rs1020066	T	G	0.7374	0.0096	0.0018	1.44E-07	28.4444
M32634.m	rs10224072	T	C	0.2481	-0.0084	0.0018	4.69E-06	21.7778
M32634.m	rs1077834	T	C	0.7961	-0.012	0.0025	2.03E-06	23.04
M32634.m	rs11161851	T	C	0.5897	0.0081	0.0018	5.41E-06	20.25
M32634.m	rs11623007	A	C	0.5662	0.0083	0.0018	3.25E-06	21.2623
M32634.m	rs11651624	A	G	0.321	0.009	0.002	6.8E-06	20.25
M32634.m	rs11832861	A	G	0.1558	0.0273	0.0054	5.08E-07	25.5586
M32634.m	rs12649508	T	G	0.2978	0.0083	0.0018	5.14E-06	21.2623
M32634.m	rs1412853	A	G	0.2693	-0.0081	0.0018	7.23E-06	20.25
M32634.m	rs1532085	A	G	0.3841	0.0089	0.0018	7.06E-07	24.4475
M32634.m	rs17153088	A	G	0.8865	-0.0121	0.0027	5.93E-06	20.0837
M32634.m	rs17444017	A	G	0.8636	0.0117	0.0026	7.81E-06	20.25
M32634.m	rs174578	A	T	0.3433	-0.0081	0.0018	6.73E-06	20.25
M32634.m	rs17836336	C	G	0.9808	0.0335	0.0072	2.99E-06	21.6483
M32634.m	rs1888108	A	G	0.6614	0.0084	0.0018	3.11E-06	21.7778
M32634.m	rs1990385	T	C	0.8651	0.0119	0.0026	4.95E-06	20.9482
M32634.m	rs1991577	T	C	0.5967	-0.0079	0.0018	8.91E-06	19.2623
M32634.m	rs1992232	A	G	0.958	0.0216	0.0045	1.41E-06	23.04
M32634.m	rs1997111	A	C	0.3558	-0.0089	0.0018	8.72E-07	24.4475
M32634.m	rs2098462	A	G	0.7432	0.0097	0.0018	1.04E-07	29.0401
M32634.m	rs284431	A	C	0.1069	0.0124	0.0027	4.58E-06	21.0919
M32634.m	rs288500	A	G	0.5823	-0.0092	0.0018	2.36E-07	26.1235

M32634.m	rs337097	T	C	0.7476	0.0085	0.0018	3.62E-06	22.2994
M32634.m	rs3740779	A	G	0.3688	0.0093	0.0018	2.97E-07	26.6944
M32634.m	rs382543	T	C	0.2625	0.0087	0.0018	1.94E-06	23.3611
M32634.m	rs435614	T	C	0.2744	-0.0085	0.0019	5.56E-06	20.0139
M32634.m	rs439401	T	C	0.351	-0.009	0.002	6.8E-06	20.25
M32634.m	rs5759640	T	C	0.0791	-0.0403	0.009	6.79E-06	20.0505
M32634.m	rs626787	C	G	0.6639	0.0092	0.0018	3.8E-07	26.1235
M32634.m	rs6593265	A	C	0.311	0.011	0.002	3.8E-08	30.25
M32634.m	rs6946189	A	G	0.25	-0.0091	0.0018	6.04E-07	25.5586
M32634.m	rs709189	A	G	0.7218	-0.0086	0.0018	1.6E-06	22.8272
M32634.m	rs7499892	T	C	0.175	-0.02	0.003	2.62E-11	44.4444
M32634.m	rs750658	A	G	0.2567	0.0085	0.0018	2.87E-06	22.2994
M32634.m	rs7576783	T	C	0.7431	0.008	0.0018	9.64E-06	19.7531
M32634.m	rs7670801	T	C	0.3261	-0.008	0.0018	7.52E-06	19.7531
M32634.m	rs7837038	A	G	0.4808	0.0089	0.0018	5.54E-07	24.4475
M32634.m	rs7912716	T	C	0.104	0.015	0.003	5.73E-07	25
M32634.m	rs7952698	A	G	0.2345	0.009	0.0018	8.69E-07	25
M32634.m	rs928563	T	C	0.737	0.0084	0.0018	3.74E-06	21.7778
M32634.m	rs9570619	A	G	0.7337	-0.0082	0.0018	6.39E-06	20.7531
M32634.m	rs981735	T	C	0.759	0.0091	0.0018	6.08E-07	25.5586
M32634.m	rs9964067	T	C	0.6791	-0.0088	0.0018	1.11E-06	23.9012
M32635.m	rs10758151	T	C	0.1408	-0.0222	0.0049	6.21E-06	20.5264
M32635.m	rs10876239	T	G	0.3759	0.0155	0.0035	9.31E-06	19.6122
M32635.m	rs10924919	T	C	0.3733	-0.0158	0.0035	5.48E-06	20.3788
M32635.m	rs11045907	A	T	0.7489	0.0165	0.0036	4.27E-06	21.0069
M32635.m	rs11574452	A	C	0.0424	-0.0379	0.008	1.96E-06	22.4439
M32635.m	rs1260326	T	C	0.4078	0.0179	0.0034	1.64E-07	27.7171
M32635.m	rs174535	T	C	0.6677	-0.044	0.0035	2.82E-36	158.041
M32635.m	rs17764324	T	G	0.087	0.0262	0.0059	9.46E-06	19.7196
M32635.m	rs2070895	A	G	0.21	0.0257	0.0042	1.22E-09	37.4427
M32635.m	rs2199680	A	T	0.8328	-0.0215	0.0043	5.88E-07	25
M32635.m	rs2828209	T	C	0.051	-0.0361	0.0076	1.78E-06	22.5625
M32635.m	rs551488	T	C	0.7412	0.0157	0.0035	7.41E-06	20.1216
M32635.m	rs7210933	A	T	0.7569	0.0163	0.0036	8.17E-06	20.5008
M32635.m	rs7529462	C	G	0.7318	0.0164	0.0036	4.93E-06	20.7531
M32635.m	rs9570044	A	C	0.1057	0.0246	0.0052	2.38E-06	22.3802
M32644.m	rs10028206	T	C	0.1218	-0.0133	0.0027	8.97E-07	24.2647
M32644.m	rs10162953	T	C	0.6424	0.0081	0.0018	9.14E-06	20.25
M32644.m	rs10758335	T	C	0.6855	-0.0084	0.0018	4.46E-06	21.7778
M32644.m	rs10869977	A	G	0.4381	-0.0089	0.0018	9.69E-07	24.4475
M32644.m	rs10927354	C	G	0.3072	0.0085	0.0018	4.41E-06	22.2994
M32644.m	rs11654066	T	C	0.5837	0.009	0.0018	8.68E-07	25
M32644.m	rs11735987	A	G	0.305	0.009	0.002	6.8E-06	20.25
M32644.m	rs11896523	A	G	0.3492	-0.0098	0.0019	1.28E-07	26.6039
M32644.m	rs12086247	A	G	0.1209	-0.0123	0.0027	5.77E-06	20.7531
M32644.m	rs12191817	T	G	0.684	0.0104	0.0018	1.66E-08	33.3827
M32644.m	rs12272002	A	T	0.8447	-0.0124	0.0027	3.68E-06	21.0919
M32644.m	rs12484	T	C	0.427	-0.009	0.002	6.8E-06	20.25
M32644.m	rs13132043	T	C	0.2928	-0.0084	0.0018	4.58E-06	21.7778
M32644.m	rs17279639	A	G	0.8725	-0.0125	0.0027	3.83E-06	21.4335
M32644.m	rs17444017	A	G	0.8637	0.0132	0.0027	9.78E-07	23.9012
M32644.m	rs2581354	T	C	0.5938	0.0088	0.0018	1.37E-06	23.9012
M32644.m	rs2657761	T	C	0.7123	-0.0084	0.0018	4.99E-06	21.7778
M32644.m	rs267583	A	G	0.2882	0.0086	0.0018	2.88E-06	22.8272
M32644.m	rs2897863	T	G	0.697	0.0082	0.0018	7.5E-06	20.7531

M32644.m	rs334698	C	G	0.0445	-0.0246	0.0046	7.72E-08	28.5992
M32644.m	rs3757514	A	T	0.9211	-0.0364	0.0079	3.71E-06	21.2299
M32644.m	rs4541909	T	C	0.674	0.01	0.0018	4.65E-08	30.8642
M32644.m	rs4711082	T	C	0.6337	-0.0083	0.0018	5.92E-06	21.2623
M32644.m	rs4719119	T	C	0.4955	0.0085	0.0018	3E-06	22.2994
M32644.m	rs4766159	A	C	0.7548	-0.022	0.0049	8.37E-06	20.1583
M32644.m	rs6466580	T	C	0.6573	-0.0096	0.0018	1.41E-07	28.4444
M32644.m	rs6557106	T	C	0.6688	0.0089	0.0018	1.08E-06	24.4475
M32644.m	rs6593266	A	G	0.306	0.01	0.002	5.73E-07	25
M32644.m	rs7025315	C	G	0.3317	0.0123	0.0025	6.54E-07	24.2064
M32644.m	rs723967	T	C	0.4929	0.0095	0.0019	3.27E-07	25
M32644.m	rs738715	A	C	0.5394	0.0087	0.0018	1.83E-06	23.3611
M32644.m	rs7552457	A	C	0.7283	-0.0085	0.0019	5.43E-06	20.0139
M32644.m	rs759287	A	G	0.3082	0.0094	0.0019	4.48E-07	24.4765
M32644.m	rs769108	T	C	0.6089	-0.0084	0.0018	3.73E-06	21.7778
M32644.m	rs8082649	A	G	0.3275	0.009	0.0019	1.37E-06	22.4377
M32644.m	rs9388675	T	G	0.6925	-0.0084	0.0019	5.81E-06	19.5457
M32644.m	rs9570627	T	G	0.8432	-0.0121	0.0027	7.47E-06	20.0837
M32644.m	rs992837	C	G	0.6787	-0.0082	0.0018	8.92E-06	20.7531
M32644.m	rs9936831	A	T	0.9266	0.0212	0.0041	2.88E-07	26.7365
M32651.m	rs10037373	T	C	0.5465	0.013	0.0027	1.77E-06	23.1824
M32651.m	rs10744682	A	G	0.3319	0.0133	0.0027	1.25E-06	24.2647
M32651.m	rs10957330	A	G	0.2929	0.0129	0.0028	4.29E-06	21.2258
M32651.m	rs10995865	A	C	0.0589	0.0286	0.0062	3.92E-06	21.2789
M32651.m	rs11166906	A	G	0.3594	0.0124	0.0027	5.6E-06	21.0919
M32651.m	rs12719343	A	G	0.3179	0.0124	0.0027	6.09E-06	21.0919
M32651.m	rs1517049	A	G	0.5559	0.0296	0.0067	9.98E-06	19.5179
M32651.m	rs17035469	T	C	0.3092	-0.015	0.0028	1.2E-07	28.699
M32651.m	rs17142484	T	G	0.0349	0.0391	0.0077	4.07E-07	25.7853
M32651.m	rs1948260	T	C	0.5206	0.0123	0.0027	7.1E-06	20.7531
M32651.m	rs1991401	A	G	0.6758	0.0131	0.0027	1.91E-06	23.5405
M32651.m	rs2555100	C	G	0.6638	0.0123	0.0027	6.86E-06	20.7531
M32651.m	rs3762920	A	G	0.6758	0.0129	0.0028	3.15E-06	21.2258
M32651.m	rs4305648	A	G	0.3639	0.0127	0.0027	2.96E-06	22.1248
M32651.m	rs4316494	A	G	0.4925	0.0121	0.0027	8.33E-06	20.0837
M32651.m	rs452767	T	C	0.5251	0.0125	0.0027	5.24E-06	21.4335
M32651.m	rs4752176	T	C	0.4565	-0.0122	0.0027	7.83E-06	20.417
M32651.m	rs4870458	A	G	0.9115	-0.0204	0.0046	8.31E-06	19.6673
M32651.m	rs6782877	A	G	0.349	0.0132	0.0027	1.08E-06	23.9012
M32651.m	rs6898211	A	C	0.0348	0.037	0.0077	1.62E-06	23.0899
M32651.m	rs6953983	A	G	0.3217	0.0123	0.0027	6.79E-06	20.7531
M32651.m	rs728661	T	C	0.8438	-0.0181	0.0037	1.28E-06	23.9306
M32651.m	rs7924340	T	C	0.4691	0.0124	0.0027	4.63E-06	21.0919
M32651.m	rs9423399	A	G	0.3176	0.0137	0.0028	5.89E-07	23.9401
M32651.m	rs9424148	A	C	0.7912	-0.0201	0.0036	1.61E-08	31.1736
M32651.m	rs9626993	T	C	0.3485	0.0126	0.0028	6.07E-06	20.25
M32654.m	rs10070034	A	G	0.0297	-0.0409	0.0065	4.3E-10	39.5931
M32654.m	rs10792093	T	G	0.1084	0.0164	0.0035	3.15E-06	21.9559
M32654.m	rs11589817	A	G	0.7885	0.0124	0.0026	2.46E-06	22.7456
M32654.m	rs11807	T	C	0.7967	-0.0123	0.0026	2.42E-06	22.3802
M32654.m	rs12138756	A	G	0.3354	0.0116	0.0025	3.6E-06	21.5296
M32654.m	rs12573437	A	C	0.087	0.0377	0.0084	7.96E-06	20.143
M32654.m	rs1578905	T	G	0.977	0.042	0.009	3.06E-06	21.7778
M32654.m	rs17171585	T	G	0.943	0.0331	0.0049	2.06E-11	45.6314
M32654.m	rs17488228	A	T	0.2892	-0.0115	0.0026	6.51E-06	19.5636

M32654.m	rs17768474	T	C	0.0454	-0.0273	0.0057	1.5E-06	22.9391
M32654.m	rs1948709	T	C	0.8952	0.0186	0.0038	7.72E-07	23.9584
M32654.m	rs2186677	A	G	0.2785	-0.0124	0.0027	5.86E-06	21.0919
M32654.m	rs2269340	T	C	0.9246	-0.0222	0.0044	3.58E-07	25.4566
M32654.m	rs2291428	C	G	0.2351	-0.0168	0.0026	8.71E-11	41.7515
M32654.m	rs2610815	T	C	0.9355	-0.0195	0.0043	5.84E-06	20.5652
M32654.m	rs273913	T	C	0.3822	0.0261	0.0025	1.08E-25	108.994
M32654.m	rs316019	A	C	0.0986	-0.0229	0.0041	2.06E-08	31.1963
M32654.m	rs3822311	T	C	0.9654	0.0303	0.0065	3.43E-06	21.7299
M32654.m	rs3914026	A	G	0.5162	-0.0109	0.0025	9.8E-06	19.0096
M32654.m	rs4781555	A	G	0.8048	0.017	0.0036	2.03E-06	22.2994
M32654.m	rs6429601	A	C	0.1819	0.0119	0.0027	7.08E-06	19.4252
M32654.m	rs6691848	A	G	0.9639	0.0288	0.0061	2.12E-06	22.2908
M32654.m	rs6817256	T	C	0.0773	0.0213	0.0045	1.99E-06	22.4044
M32654.m	rs6862720	T	C	0.0785	0.0191	0.0041	4.28E-06	21.702
M32654.m	rs7737937	A	G	0.1512	-0.0323	0.0034	7.89E-22	90.25
M32654.m	rs7819084	A	G	0.2474	0.0123	0.0026	2.45E-06	22.3802
M32654.m	rs927907	A	G	0.235	-0.0115	0.0026	7.73E-06	19.5636
M32654.m	rs9382940	T	C	0.1616	0.0172	0.0033	2.31E-07	27.1662
M32654.m	rs9551761	A	G	0.8809	0.017	0.0035	1.51E-06	23.5918
M32672.m	rs11007183	A	G	0.1131	-0.0246	0.0056	9.79E-06	19.2972
M32672.m	rs1132054	T	C	0.575	0.02	0.0042	2.34E-06	22.6757
M32672.m	rs11593751	T	C	0.0198	0.0663	0.0144	4.18E-06	21.1984
M32672.m	rs11613331	A	G	0.5532	0.0374	0.0036	2.23E-25	107.929
M32672.m	rs11727631	T	C	0.2943	0.017	0.0037	5.18E-06	21.1103
M32672.m	rs11729049	A	G	0.6296	0.0173	0.0037	3.67E-06	21.8619
M32672.m	rs12233879	A	G	0.1383	-0.0273	0.0054	4.46E-07	25.5586
M32672.m	rs12670725	A	G	0.8147	0.0205	0.0045	6.25E-06	20.7531
M32672.m	rs1449167	T	C	0.6415	0.0345	0.0076	5.43E-06	20.6068
M32672.m	rs1600760	A	T	0.663	-0.0216	0.0037	5.12E-09	34.0804
M32672.m	rs17279437	A	G	0.0953	0.059	0.0063	1.25E-20	87.7047
M32672.m	rs17776892	A	G	0.3028	0.0349	0.0076	4.02E-06	21.0874
M32672.m	rs2159892	T	G	0.6432	-0.0202	0.0037	3.63E-08	29.8057
M32672.m	rs2512652	C	G	0.704	-0.0521	0.0115	5.76E-06	20.5248
M32672.m	rs252984	T	C	0.1853	-0.0204	0.0046	7.64E-06	19.6673
M32672.m	rs2551453	T	C	0.2649	-0.0199	0.0044	7.28E-06	20.4551
M32672.m	rs3782867	C	G	0.8517	-0.025	0.0053	2.76E-06	22.2499
M32672.m	rs715	T	C	0.7139	0.0361	0.0044	2.46E-16	67.3146
M32672.m	rs7556040	T	G	0.9548	-0.0411	0.0088	3.38E-06	21.8131
M32672.m	rs9662967	T	C	0.6984	-0.0188	0.0038	7.39E-07	24.4765
M32675.m	rs10511732	C	G	0.8576	-0.0189	0.0039	1.66E-06	23.4852
M32675.m	rs11059810	T	C	0.7772	-0.0075	0.0017	8.87E-06	19.4637
M32675.m	rs11102332	A	G	0.1938	-0.0079	0.0017	5.37E-06	21.5952
M32675.m	rs11626796	A	C	0.0117	0.0276	0.0061	6.35E-06	20.4719
M32675.m	rs11741189	T	C	0.1763	-0.0086	0.0017	8.97E-07	25.5917
M32675.m	rs11753326	T	C	0.9099	0.0112	0.0025	9.63E-06	20.0704
M32675.m	rs1345255	A	G	0.8677	-0.0085	0.0018	2.12E-06	22.2994
M32675.m	rs1511592	A	C	0.8679	-0.0082	0.0018	8.34E-06	20.7531
M32675.m	rs1867017	T	G	0.221	-0.0079	0.0017	2.75E-06	21.5952
M32675.m	rs1950305	T	C	0.1208	0.0088	0.0018	1.72E-06	23.9012
M32675.m	rs1950305	T	C	0.1208	0.0088	0.0018	1.72E-06	23.9012
M32675.m	rs2074597	A	C	0.169	-0.009	0.002	6.8E-06	20.25
M32675.m	rs2101330	A	G	0.5266	0.0135	0.0028	1.08E-06	23.2462
M32675.m	rs2425463	T	G	0.8519	0.0085	0.0019	4.04E-06	20.0139
M32675.m	rs3737294	A	G	0.857	0.0081	0.0018	4.83E-06	20.25

M32675.m	rs3800014	A	C	0.1334	-0.0094	0.0018	2.68E-07	27.2716
M32675.m	rs41070	T	C	0.8499	0.0082	0.0018	4.35E-06	20.7531
M32675.m	rs6434188	T	C	0.2169	0.0083	0.0017	9.92E-07	23.8374
M32675.m	rs6867478	A	G	0.0139	0.0695	0.0125	2.97E-08	30.9136
M32675.m	rs7010049	A	G	0.9352	-0.024	0.0052	3.53E-06	21.3018
M32675.m	rs8007741	A	G	0.8554	0.0082	0.0018	5.06E-06	20.7531
M32675.m	rs926646	T	C	0.9739	-0.0196	0.0044	6.92E-06	19.843
M32675.m	rs9500927	A	G	0.1534	0.009	0.0018	5.79E-07	25
M32675.m	rs9566165	T	C	0.0679	0.0132	0.0026	4.47E-07	25.7751
M32691.m	rs10829186	A	G	0.8267	0.0372	0.0083	7.99E-06	20.0877
M32691.m	rs11140542	A	T	0.1939	0.034	0.0077	8.87E-06	19.4974
M32691.m	rs11780910	C	G	0.9873	0.1939	0.0405	1.68E-06	22.9216
M32691.m	rs12450208	T	C	0.0365	-0.0841	0.0178	2.3E-06	22.323
M32691.m	rs12621494	A	C	0.0275	-0.1005	0.0207	1.27E-06	23.5717
M32691.m	rs12778920	A	C	0.2745	0.0371	0.0068	6.05E-08	29.7667
M32691.m	rs209338	A	T	0.9651	0.078	0.0173	6.39E-06	20.3281
M32691.m	rs2244987	A	T	0.8885	-0.0479	0.0102	2.73E-06	22.0532
M32691.m	rs2932198	A	G	0.3908	0.0305	0.0065	2.5E-06	22.0178
M32691.m	rs3133571	T	C	0.2559	-0.0338	0.0074	5.05E-06	20.8627
M32691.m	rs457273	A	G	0.8172	-0.0597	0.011	5.34E-08	29.4553
M32691.m	rs6563540	T	G	0.1256	0.0428	0.0091	2.72E-06	22.121
M32691.m	rs7477283	A	T	0.8712	-0.0417	0.0094	8.8E-06	19.6796
M32691.m	rs7513605	T	C	0.1575	0.0389	0.0085	4.1E-06	20.9441
M32691.m	rs7843564	T	G	0.9307	0.0554	0.0121	4.64E-06	20.9628
M32691.m	rs7997650	A	G	0.0812	-0.0509	0.011	3.78E-06	21.4117
M32691.m	rs913080	T	C	0.3661	-0.0417	0.0089	2.64E-06	21.9529
M32691.m	rs9287841	A	T	0.9108	0.0462	0.0104	8.71E-06	19.7341
M32698.m	rs1028578	T	C	0.183	0.0114	0.0025	7.52E-06	20.7936
M32698.m	rs11106542	T	C	0.9498	-0.0321	0.0068	2.31E-06	22.284
M32698.m	rs12356193	A	G	0.8359	0.0236	0.0026	8.36E-20	82.3905
M32698.m	rs12465936	A	G	0.3006	0.0115	0.0025	5.13E-06	21.16
M32698.m	rs12659397	T	C	0.7796	0.011	0.0025	8.07E-06	19.36
M32698.m	rs16876782	A	G	0.1222	0.0144	0.0032	8.18E-06	20.25
M32698.m	rs17070627	T	C	0.0388	-0.0426	0.0092	3.88E-06	21.4409
M32698.m	rs1904071	T	G	0.7348	-0.0161	0.0024	3.12E-11	45.0017
M32698.m	rs2590763	T	C	0.4813	0.0106	0.0024	9.84E-06	19.5069
M32698.m	rs295893	T	C	0.8293	-0.0126	0.0026	1.5E-06	23.4852
M32698.m	rs2980973	T	C	0.9084	-0.0157	0.0034	4.96E-06	21.3227
M32698.m	rs3770063	A	T	0.0352	-0.0256	0.0056	5.88E-06	20.898
M32698.m	rs4537895	A	G	0.7962	0.0114	0.0025	6.62E-06	20.7936
M32698.m	rs4857879	A	C	0.8382	0.0225	0.0049	3.56E-06	21.085
M32698.m	rs6996022	C	G	0.1711	-0.012	0.0025	2.35E-06	23.04
M32698.m	rs9457843	T	C	0.1218	0.0159	0.0032	9.53E-07	24.6885
M32698.m	rs9544223	A	C	0.8771	-0.0181	0.0038	2.07E-06	22.6877
M32698.m	rs9809318	T	C	0.7743	0.0125	0.0026	9.84E-07	23.1139
M32709.m	rs1005390	T	G	0.7998	0.0261	0.0034	2.5E-14	58.9282
M32709.m	rs10065535	A	G	0.025	0.046	0.01	4.23E-06	21.16
M32709.m	rs10083422	T	C	0.3043	0.0119	0.0026	6.74E-06	20.9482
M32709.m	rs10771776	A	G	0.5978	0.0132	0.0026	2.91E-07	25.7751
M32709.m	rs10776672	A	G	0.8929	-0.0266	0.0045	3.35E-09	34.9412
M32709.m	rs11101730	A	T	0.9414	-0.0553	0.0076	3.28E-13	52.9448
M32709.m	rs11803189	T	C	0.8545	0.0164	0.0035	3.96E-06	21.9559
M32709.m	rs11978239	A	G	0.7091	0.0145	0.0026	4.75E-08	31.1021
M32709.m	rs12602901	C	G	0.1717	-0.0368	0.0034	1.15E-26	117.149
M32709.m	rs12755479	A	G	0.031	-0.0773	0.0161	1.59E-06	23.0519

M32709.m	rs1352815	A	C	0.6865	-0.0118	0.0026	8.16E-06	20.5976
M32709.m	rs17047802	T	C	0.8891	-0.038	0.0083	4.12E-06	20.961
M32709.m	rs17217996	T	C	0.6869	0.0119	0.0026	7.26E-06	20.9482
M32709.m	rs17810453	A	G	0.6796	-0.0123	0.0026	3.16E-06	22.3802
M32709.m	rs1801394	A	G	0.4675	0.0121	0.0026	3.33E-06	21.6583
M32709.m	rs2184568	A	G	0.6762	-0.0248	0.0055	6.01E-06	20.3319
M32709.m	rs2255024	C	G	0.7203	-0.0173	0.0033	1.3E-07	27.483
M32709.m	rs2256321	T	C	0.6891	-0.0121	0.0027	5.53E-06	20.0837
M32709.m	rs269066	A	G	0.2794	-0.0122	0.0027	5.28E-06	20.417
M32709.m	rs2968858	T	C	0.5284	0.0124	0.0026	2.07E-06	22.7456
M32709.m	rs3798167	T	G	0.2058	0.0203	0.0034	2.02E-09	35.6479
M32709.m	rs4422759	T	C	0.1519	0.0159	0.0035	6.73E-06	20.6376
M32709.m	rs4961335	A	G	0.5531	0.0123	0.0026	1.89E-06	22.3802
M32709.m	rs4973517	T	C	0.7143	-0.0119	0.0027	9.1E-06	19.4252
M32709.m	rs6502844	T	C	0.8435	0.0214	0.0035	6.92E-10	37.3845
M32709.m	rs662138	C	G	0.8307	0.0342	0.0035	1.04E-22	95.4808
M32709.m	rs7165678	A	G	0.6088	-0.0123	0.0026	2.63E-06	22.3802
M32709.m	rs7281501	T	G	0.9362	0.0482	0.0107	7.18E-06	20.2921
M32709.m	rs7806249	T	C	0.6546	0.0122	0.0026	2.62E-06	22.0178
M32709.m	rs7982884	A	G	0.0425	-0.0313	0.007	7.61E-06	19.9937
M32709.m	rs892428	C	G	0.3355	-0.0116	0.0026	9.94E-06	19.9053
M32709.m	rs9959803	T	C	0.96	-0.0291	0.0066	9.74E-06	19.4401
M32729.m	rs10127987	T	C	0.4763	0.0092	0.0019	1.13E-06	23.446
M32729.m	rs10485860	T	C	0.8306	0.0123	0.0025	1.07E-06	24.2064
M32729.m	rs10504821	A	G	0.7046	-0.0095	0.0019	5.81E-07	25
M32729.m	rs10741150	T	C	0.3902	-0.0087	0.0019	4.02E-06	20.9668
M32729.m	rs10753839	A	G	0.4657	0.0088	0.0019	2.49E-06	21.4515
M32729.m	rs10784625	T	C	0.7213	0.0262	0.0058	6.12E-06	20.4055
M32729.m	rs11024074	T	C	0.7057	-0.0088	0.0019	3.28E-06	21.4515
M32729.m	rs11755524	T	C	0.0152	-0.0405	0.0089	5.7E-06	20.7076
M32729.m	rs11772826	A	G	0.323	0.009	0.002	6.8E-06	20.25
M32729.m	rs12251822	A	G	0.6878	-0.009	0.0019	2.08E-06	22.4377
M32729.m	rs12435886	A	G	0.4175	-0.0087	0.0019	4.04E-06	20.9668
M32729.m	rs1274280	T	C	0.1181	0.0129	0.0028	4.95E-06	21.2258
M32729.m	rs12827532	T	G	0.3678	-0.0084	0.0019	8.35E-06	19.5457
M32729.m	rs13111508	T	C	0.6934	0.0088	0.0019	4.16E-06	21.4515
M32729.m	rs1325274	T	C	0.123	-0.0127	0.0029	8.23E-06	19.1784
M32729.m	rs1424532	A	G	0.6678	-0.009	0.0019	1.65E-06	22.4377
M32729.m	rs159111	A	G	0.1263	-0.0125	0.0028	6.79E-06	19.9298
M32729.m	rs17357652	T	C	0.6988	-0.0095	0.0019	5.48E-07	25
M32729.m	rs174892	T	G	0.2779	0.0086	0.0019	6.63E-06	20.4875
M32729.m	rs17570915	A	G	0.1402	0.0127	0.0028	4.93E-06	20.5727
M32729.m	rs1864658	T	C	0.6948	0.0086	0.0019	7.67E-06	20.4875
M32729.m	rs2000813	T	C	0.2942	0.009	0.0019	2.31E-06	22.4377
M32729.m	rs2170489	A	G	0.4041	-0.0099	0.0019	1.57E-07	27.1496
M32729.m	rs2237231	T	C	0.068	0.0178	0.0037	1.85E-06	23.1439
M32729.m	rs2243498	A	G	0.4479	0.0096	0.0019	2.95E-07	25.5291
M32729.m	rs2282040	A	G	0.8981	0.0381	0.0086	9.29E-06	19.627
M32729.m	rs2523096	T	C	0.8553	-0.0124	0.0028	8.95E-06	19.6122
M32729.m	rs261332	A	G	0.1695	0.0121	0.0027	8.88E-06	20.0837
M32729.m	rs2828004	T	G	0.9477	0.0216	0.0045	1.87E-06	23.04
M32729.m	rs2934554	T	C	0.4337	0.0086	0.0019	4.53E-06	20.4875
M32729.m	rs3764261	A	C	0.3354	0.0113	0.0019	3.39E-09	35.3712
M32729.m	rs3913305	A	G	0.4228	-0.0084	0.0019	6.65E-06	19.5457
M32729.m	rs4405345	C	G	0.6648	0.0088	0.0019	2.82E-06	21.4515

M32729.m	rs4542981	A	C	0.6707	-0.0092	0.0019	1.24E-06	23.446
M32729.m	rs622997	T	C	0.4844	-0.0086	0.0019	4.28E-06	20.4875
M32729.m	rs6706970	T	C	0.5982	-0.0091	0.0019	1.25E-06	22.9391
M32729.m	rs6719501	T	G	0.5183	0.0097	0.0019	2.6E-07	26.0637
M32729.m	rs6732028	A	G	0.5425	0.0085	0.0019	7.51E-06	20.0139
M32729.m	rs6791299	A	G	0.534	-0.0095	0.0019	3.71E-07	25
M32729.m	rs7157237	T	C	0.5328	0.0084	0.0019	7.68E-06	19.5457
M32729.m	rs7554924	A	T	0.6149	0.0084	0.0019	8.58E-06	19.5457
M32729.m	rs7819333	C	G	0.3311	-0.0099	0.0019	1.45E-07	27.1496
M32729.m	rs8008122	T	C	0.3667	-0.0085	0.0019	7.87E-06	20.0139
M32729.m	rs809710	A	G	0.5438	0.0083	0.0019	8.49E-06	19.0831
M32729.m	rs9322929	T	C	0.6994	-0.0088	0.0019	3.57E-06	21.4515
M32729.m	rs9393674	A	G	0.6666	-0.0088	0.0019	3.3E-06	21.4515
M32729.m	rs9859230	A	G	0.325	-0.0083	0.0019	9.13E-06	19.0831
M32729.m	rs990482	T	G	0.2786	0.0088	0.0019	4.44E-06	21.4515
M32735.m	rs10110717	T	C	0.688	-0.029	0.0065	9.12E-06	19.9053
M32735.m	rs10198903	A	G	0.295	-0.0342	0.0071	1.61E-06	23.2025
M32735.m	rs12202901	A	T	0.2064	0.0349	0.0078	8.68E-06	20.0199
M32735.m	rs1252109	T	C	0.2679	0.0327	0.0067	8.88E-07	23.8202
M32735.m	rs12787810	T	C	0.814	-0.0372	0.0077	1.23E-06	23.3402
M32735.m	rs13162494	A	G	0.0653	-0.0566	0.0123	4.35E-06	21.175
M32735.m	rs1368616	T	G	0.505	-0.0471	0.0106	8.76E-06	19.7438
M32735.m	rs16897515	A	C	0.1641	-0.0412	0.0083	7.25E-07	24.6399
M32735.m	rs16958414	T	G	0.7925	0.0332	0.0075	9.11E-06	19.5954
M32735.m	rs17446483	T	C	0.1177	-0.0414	0.0093	9.43E-06	19.8169
M32735.m	rs2814818	A	G	0.6985	0.031	0.0065	2.15E-06	22.7456
M32735.m	rs4099	T	G	0.8988	0.0457	0.0103	9.92E-06	19.686
M32735.m	rs4680	A	G	0.5065	-0.0436	0.0059	1.31E-13	54.6096
M32735.m	rs591058	T	C	0.5062	0.0283	0.0059	1.74E-06	23.0075
M32735.m	rs7151027	A	T	0.3207	-0.0294	0.0065	6.92E-06	20.4582
M32735.m	rs831607	T	C	0.5112	-0.0404	0.0089	6.13E-06	20.6055
M32735.m	rs887200	T	C	0.8806	-0.0492	0.0091	6.46E-08	29.2313
M32735.m	rs9308696	A	G	0.9751	-0.1558	0.0316	8.19E-07	24.3086
M32739.m	rs1022039	A	T	0.2991	0.0198	0.0043	5.47E-06	21.2028
M32739.m	rs10750719	T	G	0.877	0.0179	0.004	7.17E-06	20.0256
M32739.m	rs11055626	C	G	0.9286	-0.0232	0.0052	8.62E-06	19.9053
M32739.m	rs1196661	A	G	0.8665	-0.0191	0.0042	5.89E-06	20.6808
M32739.m	rs12357763	T	C	0.0454	0.0343	0.007	8.35E-07	24.01
M32739.m	rs12617815	T	G	0.0449	0.0477	0.0096	7.54E-07	24.6885
M32739.m	rs7946041	A	G	0.0211	0.0454	0.0097	2.75E-06	21.9063
M32739.m	rs9440550	A	G	0.1936	-0.0148	0.0033	6.7E-06	20.1139
M32740.m	rs10762854	A	G	0.6966	-0.0164	0.0036	6.86E-06	20.7531
M32740.m	rs10916685	A	T	0.6493	0.0095	0.0018	8.65E-08	27.8549
M32740.m	rs11054704	A	G	0.1397	-0.0125	0.0026	1.32E-06	23.1139
M32740.m	rs11691877	T	C	0.3084	-0.009	0.0018	2.83E-07	25
M32740.m	rs11778288	A	G	0.3156	0.008	0.0017	5.03E-06	22.1453
M32740.m	rs12477726	A	G	0.2434	0.0175	0.0039	7.57E-06	20.1348
M32740.m	rs12569017	A	T	0.0734	-0.0151	0.0033	6.39E-06	20.9376
M32740.m	rs12712206	A	G	0.4119	0.0077	0.0017	7.69E-06	20.5156
M32740.m	rs12730665	A	G	0.6775	0.0078	0.0017	7.23E-06	21.0519
M32740.m	rs12742877	C	G	0.3923	-0.0082	0.0017	2.7E-06	23.2664
M32740.m	rs12873548	T	C	0.3269	-0.0079	0.0018	6.65E-06	19.2623
M32740.m	rs12899483	A	G	0.7034	0.0084	0.0017	1.59E-06	24.4152
M32740.m	rs13098833	A	G	0.7208	-0.0084	0.0018	1.93E-06	21.7778
M32740.m	rs1620675	T	G	0.3434	0.0079	0.0017	6.02E-06	21.5952

M32740.m	rs17101092	C	G	0.3183	-0.0077	0.0017	9.76E-06	20.5156
M32740.m	rs1719079	A	G	0.9476	0.0186	0.0041	4.92E-06	20.5806
M32740.m	rs17761583	A	G	0.8209	0.011	0.0024	6.91E-06	21.0069
M32740.m	rs1992235	A	G	0.3191	-0.0078	0.0017	7.66E-06	21.0519
M32740.m	rs2029209	A	G	0.5491	0.0081	0.0017	2.08E-06	22.7024
M32740.m	rs2038410	T	C	0.4395	-0.009	0.0017	1.65E-07	28.0277
M32740.m	rs2371276	T	C	0.4783	-0.0093	0.0018	1.91E-07	26.6944
M32740.m	rs2423437	A	G	0.6687	-0.0082	0.0018	3.69E-06	20.7531
M32740.m	rs2869297	A	G	0.7019	-0.0079	0.0018	7.37E-06	19.2623
M32740.m	rs2875878	C	G	0.7045	-0.0079	0.0017	6.17E-06	21.5952
M32740.m	rs3754909	A	G	0.6615	-0.0081	0.0018	3.73E-06	20.25
M32740.m	rs3915879	T	C	0.3166	-0.0085	0.0018	3.91E-06	22.2994
M32740.m	rs4075958	A	G	0.2613	0.0078	0.0018	9.43E-06	18.7778
M32740.m	rs429460	A	T	0.2808	0.0079	0.0018	6.81E-06	19.2623
M32740.m	rs4562058	T	C	0.3093	-0.008	0.0017	3.88E-06	22.1453
M32740.m	rs4755524	C	G	0.3119	0.0079	0.0018	7.9E-06	19.2623
M32740.m	rs4981021	T	C	0.282	-0.0084	0.0018	1.92E-06	21.7778
M32740.m	rs529833	A	G	0.6913	-0.0093	0.0018	1.32E-07	26.6944
M32740.m	rs569137	A	G	0.3585	-0.0079	0.0017	5.13E-06	21.5952
M32740.m	rs6151429	T	C	0.9291	-0.0289	0.0034	6.51E-18	72.25
M32740.m	rs6429032	T	C	0.0905	-0.0273	0.0058	2.12E-06	22.1549
M32740.m	rs6503078	T	G	0.283	-0.009	0.002	6.8E-06	20.25
M32740.m	rs651007	T	C	0.2007	-0.0152	0.0024	2.78E-10	40.1111
M32740.m	rs6545816	A	C	0.4566	-0.0078	0.0017	6.06E-06	21.0519
M32740.m	rs6555389	A	T	0.3307	0.0083	0.0017	2.16E-06	23.8374
M32740.m	rs6832662	A	G	0.4257	-0.0079	0.0017	6.07E-06	21.5952
M32740.m	rs6928245	A	G	0.3637	0.0091	0.0017	1.79E-07	28.654
M32740.m	rs7151149	T	C	0.2898	-0.0082	0.0017	3.1E-06	23.2664
M32740.m	rs743760	C	G	0.3375	-0.0078	0.0017	7.18E-06	21.0519
M32740.m	rs7560697	T	C	0.2811	0.0084	0.0018	1.79E-06	21.7778
M32740.m	rs8005168	T	C	0.3852	0.0077	0.0017	9.31E-06	20.5156
M32740.m	rs890498	C	G	0.6478	-0.0078	0.0017	7.23E-06	21.0519
M32740.m	rs9038	T	C	0.616	0.009	0.002	6.8E-06	20.25
M32740.m	rs930551	A	G	0.069	-0.018	0.004	6.8E-06	20.25
M32740.m	rs9320187	A	G	0.065	-0.0154	0.0034	4.9E-06	20.5156
M32740.m	rs9322898	T	C	0.318	-0.009	0.002	6.8E-06	20.25
M32740.m	rs9397197	A	G	0.2948	0.0083	0.0018	2.21E-06	21.2623
M32753.m	rs10761316	T	C	0.2803	-0.0336	0.0068	7.22E-07	24.4152
M32753.m	rs11185516	A	G	0.3936	0.0305	0.0054	1.39E-08	31.9016
M32753.m	rs1329561	A	G	0.1636	-0.0314	0.007	6.86E-06	20.1216
M32753.m	rs16908213	T	C	0.924	0.0466	0.0104	7.21E-06	20.0773
M32753.m	rs2433292	T	C	0.774	-0.0299	0.0067	7.78E-06	19.9156
M32753.m	rs2488751	A	G	0.4539	-0.0306	0.0066	3.22E-06	21.4959
M32753.m	rs3746024	T	C	0.8273	-0.0324	0.007	3.46E-06	21.4237
M32753.m	rs4843466	A	C	0.9159	-0.0857	0.0192	7.58E-06	19.9232
M32753.m	rs556339	T	C	0.2533	0.0382	0.0061	4.83E-10	39.2163
M32753.m	rs649622	T	G	0.097	0.0444	0.0098	5.89E-06	20.5264
M32753.m	rs7642243	C	G	0.3882	0.0481	0.006	7.5E-16	64.2669
M32753.m	rs7691901	T	G	0.8326	0.0314	0.0071	9.07E-06	19.5588
M32753.m	rs939885	A	G	0.4911	0.0316	0.0059	6.88E-08	28.686
M32754.m	rs10517818	T	C	0.8138	-0.0501	0.0111	6.62E-06	20.3718
M32754.m	rs12435324	C	G	0.0126	-0.4219	0.0908	3.37E-06	21.5897
M32754.m	rs13073145	C	G	0.0616	-0.097	0.0219	9.48E-06	19.618
M32754.m	rs1405215	A	G	0.165	0.0623	0.0116	8E-08	28.8443
M32754.m	rs16926956	T	G	0.1948	-0.052	0.0109	1.77E-06	22.759

M32754.m	rs17040357	A	G	0.9825	0.3091	0.0679	5.38E-06	20.7233
M32754.m	rs17825729	C	G	0.0842	-0.0711	0.0153	3.36E-06	21.5952
M32754.m	rs2460621	T	C	0.9207	0.0795	0.018	9.84E-06	19.5069
M32754.m	rs335810	A	C	0.9883	0.3722	0.0827	6.8E-06	20.2554
M32754.m	rs4919957	A	G	0.0653	0.1629	0.0358	5.4E-06	20.705
M32754.m	rs555851	A	G	0.549	-0.0363	0.0082	9.23E-06	19.5968
M32754.m	rs6415698	A	C	0.7774	0.0548	0.0107	3.1E-07	26.2297
M32754.m	rs7321884	T	C	0.0207	0.1488	0.0316	2.43E-06	22.1734
M32754.m	rs768328	T	C	0.228	-0.0464	0.0099	3.09E-06	21.9667
M32754.m	rs9543952	A	G	0.8491	-0.053	0.0117	5.8E-06	20.5201
M32755.m	rs10004910	A	G	0.5631	0.0179	0.0034	1.62E-07	27.7171
M32755.m	rs11905915	A	G	0.5878	-0.0166	0.0035	1.92E-06	22.4947
M32755.m	rs1365505	T	C	0.681	0.0198	0.004	1.06E-06	24.5025
M32755.m	rs1483739	A	C	0.2365	0.0213	0.0042	5.08E-07	25.7194
M32755.m	rs17130142	A	G	0.0254	0.116	0.0261	8.7E-06	19.7531
M32755.m	rs174605	T	G	0.284	0.0195	0.0042	3.07E-06	21.5561
M32755.m	rs17525557	T	C	0.956	-0.0417	0.0092	6.07E-06	20.5445
M32755.m	rs1860538	T	G	0.5561	0.0163	0.0034	1.67E-06	22.9836
M32755.m	rs2032205	T	C	0.0171	-0.0706	0.0152	3.19E-06	21.5736
M32755.m	rs2395859	A	G	0.8015	0.0199	0.0043	3.88E-06	21.4175
M32755.m	rs2934442	T	C	0.7993	0.0191	0.0043	6.85E-06	19.7301
M32755.m	rs3112734	T	C	0.0221	-0.0604	0.0135	7.79E-06	20.0173
M32755.m	rs4866873	T	C	0.8725	0.0521	0.01	1.71E-07	27.1441
M32755.m	rs4963728	T	C	0.1436	-0.0257	0.005	2.59E-07	26.4196
M32755.m	rs4977264	T	C	0.5535	-0.0162	0.0034	2.47E-06	22.7024
M32755.m	rs6077678	A	G	0.8477	-0.0246	0.005	8.75E-07	24.2064
M32755.m	rs7228173	T	C	0.2694	0.0187	0.0041	6.11E-06	20.8025
M32755.m	rs7337711	T	C	0.1564	0.0408	0.009	5.44E-06	20.5511
M32755.m	rs760955	T	C	0.281	0.0238	0.0049	1.09E-06	23.5918
M32755.m	rs7702466	A	G	0.1021	0.0316	0.0068	3.01E-06	21.5952
M32755.m	rs780094	T	C	0.3929	-0.0164	0.0034	1.61E-06	23.2664
M32755.m	rs9296110	T	C	0.9639	0.0427	0.0096	8.96E-06	19.784
M32755.m	rs944846	T	C	0.0802	0.0575	0.0118	1.19E-06	23.745
M32755.m	rs9513800	A	G	0.0449	-0.041	0.0089	3.85E-06	21.2221
M32757.m	rs11228368	A	G	0.4543	-0.0225	0.005	7.05E-06	20.25
M32757.m	rs11670965	A	G	0.3127	0.0347	0.0055	3.45E-10	39.8046
M32757.m	rs12038241	A	G	0.4301	0.0258	0.0049	1.84E-07	27.7234
M32757.m	rs1366688	C	G	0.6094	0.0226	0.0049	5.15E-06	21.2728
M32757.m	rs1368843	A	G	0.4256	-0.0264	0.0059	6.34E-06	20.0218
M32757.m	rs1543433	A	G	0.366	0.0239	0.005	1.99E-06	22.8484
M32757.m	rs16958664	A	G	0.0523	-0.0509	0.0113	6.26E-06	20.2898
M32757.m	rs17061049	T	C	0.9677	-0.0642	0.014	4.71E-06	21.0288
M32757.m	rs17197676	A	G	0.928	0.0751	0.0166	6.41E-06	20.4674
M32757.m	rs232668	A	G	0.0207	-0.1354	0.0299	5.97E-06	20.5067
M32757.m	rs2828489	T	C	0.6364	0.0234	0.005	2.31E-06	21.9024
M32757.m	rs2852424	A	G	0.9371	0.0554	0.0121	4.71E-06	20.9628
M32757.m	rs2932766	T	G	0.7073	-0.0621	0.0055	1.48E-29	127.485
M32757.m	rs296381	T	C	0.8285	0.1636	0.0064	5.4E-143	653.441
M32757.m	rs3732218	A	G	0.0805	-0.0532	0.0089	2.77E-09	35.7308
M32757.m	rs4715354	A	G	0.478	0.0278	0.0049	1.66E-08	32.1883
M32757.m	rs4802397	T	C	0.8588	-0.0559	0.0078	9.94E-13	51.3611
M32757.m	rs628137	A	C	0.3721	-0.0265	0.0055	1.28E-06	23.2149
M32757.m	rs7703306	C	G	0.3668	0.0221	0.005	9.37E-06	19.5364
M32757.m	rs9362410	T	C	0.3749	0.0262	0.0055	1.83E-06	22.6922
M32757.m	rs9445291	T	C	0.1411	-0.0337	0.0072	3.28E-06	21.9076

M32758.m	rs10175949	A	G	0.865	-0.0325	0.0064	3.73E-07	25.7874
M32758.m	rs1028885	A	G	0.7832	0.042	0.0093	6.06E-06	20.3954
M32758.m	rs10823597	T	C	0.7417	-0.0248	0.005	8.42E-07	24.6016
M32758.m	rs10939890	T	C	0.1641	0.0279	0.0059	2.18E-06	22.3617
M32758.m	rs11206237	A	C	0.1515	0.0263	0.0059	8.56E-06	19.8704
M32758.m	rs12461351	C	G	0.9375	0.0449	0.0095	2.09E-06	22.3381
M32758.m	rs13266017	A	C	0.5953	-0.0201	0.0043	2.35E-06	21.8502
M32758.m	rs17863762	A	G	0.0292	-0.0705	0.0139	3.8E-07	25.7246
M32758.m	rs2219067	C	G	0.7607	0.0353	0.0051	2.91E-12	47.9081
M32758.m	rs2741035	A	G	0.806	0.034	0.007	1.19E-06	23.5918
M32758.m	rs28900384	C	G	0.9662	-0.0977	0.012	3.21E-16	66.2867
M32758.m	rs3133577	T	C	0.6707	-0.0252	0.0048	1.98E-07	27.5625
M32758.m	rs3796092	A	C	0.098	0.0495	0.0073	1.17E-11	45.9795
M32758.m	rs4148324	T	G	0.6706	-0.1091	0.0048	1.5E-116	516.615
M32758.m	rs4430526	A	G	0.5227	0.0194	0.0042	3.72E-06	21.3356
M32758.m	rs4854192	A	G	0.6878	0.0221	0.005	9.69E-06	19.5364
M32758.m	rs504026	A	G	0.9734	0.1099	0.0242	5.53E-06	20.6236
M32758.m	rs6717546	A	G	0.3663	-0.0378	0.0043	1E-18	77.2764
M32758.m	rs7608713	A	G	0.2401	-0.0471	0.0051	1.45E-20	85.2907
M32758.m	rs7858984	A	G	0.3026	-0.0219	0.0049	8.88E-06	19.9754
M32758.m	rs7906674	T	G	0.479	-0.0193	0.0042	4.81E-06	21.1162
M32758.m	rs838718	A	G	0.4859	0.0222	0.0042	1.17E-07	27.9388
M32759.m	rs10135212	T	C	0.8095	-0.0262	0.0057	4.13E-06	21.1277
M32759.m	rs10175949	A	G	0.8647	-0.0346	0.0064	5.43E-08	29.2275
M32759.m	rs10744163	T	C	0.3489	-0.0217	0.0048	8E-06	20.4379
M32759.m	rs1076160	T	C	0.4749	-0.0186	0.0042	8.69E-06	19.6122
M32759.m	rs11564118	A	G	0.9236	0.0395	0.0082	1.58E-06	23.2042
M32759.m	rs12446951	A	G	0.1573	0.0282	0.0063	7.85E-06	20.0363
M32759.m	rs129313	T	G	0.0263	-0.0956	0.0214	8.13E-06	19.9567
M32759.m	rs16889589	C	G	0.0382	0.0719	0.0157	0.000005	20.9729
M32759.m	rs17649503	A	G	0.9227	-0.0416	0.0084	8.11E-07	24.5261
M32759.m	rs17863762	A	G	0.0292	-0.0705	0.0138	3.45E-07	26.0988
M32759.m	rs2219067	C	G	0.7615	0.0317	0.005	3.41E-10	40.1956
M32759.m	rs2268181	T	C	0.8405	-0.0267	0.0058	4.78E-06	21.1917
M32759.m	rs2868035	A	G	0.3422	0.0219	0.0049	7.52E-06	19.9754
M32759.m	rs28899194	T	C	0.0346	0.0903	0.0118	2.31E-14	58.5614
M32759.m	rs3108908	T	C	0.3316	0.0223	0.0048	3.03E-06	21.5838
M32759.m	rs3796092	A	C	0.0989	0.0441	0.0072	1.16E-09	37.5156
M32759.m	rs4148324	T	G	0.6696	-0.109	0.0047	5.4E-117	537.845
M32759.m	rs4711652	A	G	0.5551	0.0188	0.0042	8.56E-06	20.0363
M32759.m	rs6723936	A	G	0.6404	0.0388	0.0048	1.26E-15	65.3403
M32759.m	rs7215713	T	C	0.2296	0.0245	0.005	1.18E-06	24.01
M32759.m	rs7608713	A	G	0.2404	-0.0504	0.005	1.5E-23	101.606
M32759.m	rs7906674	T	G	0.4799	-0.0211	0.0042	5.06E-07	25.2387
M32759.m	rs838705	A	G	0.6145	0.0247	0.0043	6.57E-09	32.9957
M32759.m	rs867335	A	T	0.0201	-0.1441	0.0324	8.46E-06	19.7805
M32759.m	rs9316990	A	G	0.433	0.0189	0.0042	7.24E-06	20.25
M32761.m	rs10199511	A	G	0.1013	-0.0397	0.0057	4.11E-12	48.51
M32761.m	rs12211633	T	C	0.747	0.016	0.0036	6.74E-06	19.7531
M32761.m	rs12478111	A	G	0.2123	-0.0228	0.0046	7.45E-07	24.5671
M32761.m	rs1346644	C	G	0.1432	-0.0296	0.005	3.99E-09	35.0464
M32761.m	rs1711161	T	G	0.7246	-0.0156	0.0035	9.42E-06	19.8661
M32761.m	rs2132551	A	C	0.964	0.054	0.0116	3.56E-06	21.6706
M32761.m	rs2273664	A	G	0.0616	-0.0457	0.0071	9.84E-11	41.4301
M32761.m	rs2279399	T	C	0.4592	0.016	0.0034	3.29E-06	22.1453

M32761.m	rs2954021	A	G	0.5064	0.0172	0.0034	3.45E-07	25.5917
M32761.m	rs3742879	A	G	0.6777	-0.0161	0.0035	4.6E-06	21.16
M32761.m	rs3769545	A	G	0.6672	-0.0172	0.0035	8.13E-07	24.1502
M32761.m	rs458582	T	G	0.4996	0.0163	0.0033	1.04E-06	24.3976
M32761.m	rs6718606	C	G	0.8451	0.0267	0.0055	1.36E-06	23.5666
M32761.m	rs6735065	C	G	0.953	0.0678	0.008	1.99E-17	71.8256
M32761.m	rs7559329	T	C	0.847	0.0198	0.0044	7.79E-06	20.25
M32761.m	rs7604035	T	C	0.3261	0.031	0.0035	9.39E-19	78.449
M32761.m	rs7841945	A	T	0.8698	0.0394	0.0084	2.49E-06	22.0006
M32761.m	rs7974646	A	G	0.0866	-0.0297	0.0067	8.69E-06	19.65
M32761.m	rs798727	T	C	0.8227	-0.0211	0.0047	8E-06	20.1544
M32762.m	rs10491431	A	C	0.1504	0.0807	0.012	1.59E-11	45.2256
M32762.m	rs11174493	A	G	0.3526	0.0433	0.009	1.42E-06	23.1468
M32762.m	rs12238989	C	G	0.0233	0.1605	0.0358	7.51E-06	20.0994
M32762.m	rs1574228	A	G	0.712	-0.0432	0.0094	4.23E-06	21.1209
M32762.m	rs1940528	A	G	0.8479	-0.0649	0.0142	4.71E-06	20.8888
M32762.m	rs2567308	A	G	0.8244	-0.0524	0.0116	5.86E-06	20.4055
M32762.m	rs2592288	T	C	0.9423	-0.092	0.0197	3.15E-06	21.8094
M32762.m	rs2862259	A	C	0.4547	0.0394	0.0089	9.94E-06	19.598
M32762.m	rs296381	T	C	0.8303	0.0663	0.0117	1.35E-08	32.1111
M32762.m	rs4149081	A	G	0.1794	0.068	0.0109	5.22E-10	38.9193
M32762.m	rs532	A	G	0.771	-0.0475	0.0105	6.06E-06	20.4649
M32762.m	rs625532	A	G	0.1697	-0.0561	0.0112	5.31E-07	25.0894
M32762.m	rs6464572	C	G	0.8341	-0.0551	0.0121	5.65E-06	20.7364
M32762.m	rs6850094	T	C	0.4631	-0.0394	0.0087	6.35E-06	20.5094
M32762.m	rs9751295	A	G	0.9778	-0.1428	0.0312	4.68E-06	20.9482
M32769.m	rs1562716	T	G	0.4098	0.0457	0.0103	8.86E-06	19.686
M32769.m	rs300250	A	G	0.635	0.0276	0.0058	2.25E-06	22.6445
M32769.m	rs4633	T	C	0.5168	0.0307	0.0059	1.84E-07	27.0753
M32769.m	rs824764	T	C	0.9563	0.0709	0.0151	2.73E-06	22.0464
M32769.m	rs9348689	T	G	0.5165	0.0339	0.0062	4.41E-08	29.8962
M32786.m	rs10509673	C	G	0.1624	-0.0471	0.0078	1.78E-09	36.463
M32786.m	rs11106131	T	G	0.2423	-0.0381	0.0086	9.37E-06	19.627
M32786.m	rs11188148	A	G	0.8771	-0.0615	0.0094	7.51E-11	42.805
M32786.m	rs11754288	A	G	0.4387	0.0434	0.0062	1.88E-12	49
M32786.m	rs1938489	A	G	0.7847	-0.0316	0.0071	9.34E-06	19.8088
M32786.m	rs2071426	T	C	0.7065	0.0517	0.0068	3.97E-14	57.8047
M32786.m	rs2187662	A	G	0.034	0.0786	0.0172	4.95E-06	20.8828
M32786.m	rs2870709	T	C	0.1734	0.039	0.0085	4.2E-06	21.0519
M32786.m	rs3736083	T	C	0.5551	0.0275	0.0062	8.92E-06	19.6735
M32786.m	rs4149081	A	G	0.171	-0.0437	0.0077	1.66E-08	32.2093
M32786.m	rs4885169	A	G	0.1078	-0.0427	0.0094	5.17E-06	20.6348
M32786.m	rs7311964	T	G	0.7496	-0.0333	0.007	2.26E-06	22.6304
M32786.m	rs7495382	T	G	0.2056	-0.0448	0.0083	7.19E-08	29.134
M32786.m	rs7772658	A	G	0.0782	0.0565	0.0118	1.68E-06	22.9262
M32786.m	rs7787327	A	G	0.05	-0.0686	0.0154	8.84E-06	19.843
M32786.m	rs7810308	T	C	0.1432	-0.0388	0.0087	7.53E-06	19.8895
M32786.m	rs7870790	A	G	0.309	0.0287	0.0064	6.97E-06	20.1096
M32786.m	rs9299150	T	C	0.3005	0.0542	0.0115	2.34E-06	22.2128
M32786.m	rs994645	A	G	0.1146	-0.0417	0.0093	8.18E-06	20.1051
M32787.m	rs10199511	A	G	0.1012	-0.0281	0.0059	1.64E-06	22.6834
M32787.m	rs10798656	A	C	0.4816	0.0184	0.0034	7.63E-08	29.2872
M32787.m	rs10916248	A	T	0.8305	-0.0218	0.0048	5.1E-06	20.6267
M32787.m	rs1475961	A	G	0.6271	0.0162	0.0035	2.69E-06	21.4237
M32787.m	rs176400	A	G	0.331	0.0191	0.0036	1.2E-07	28.1489

M32787.m	rs1939769	A	G	0.0647	0.0352	0.0074	1.77E-06	22.6267
M32787.m	rs206825	T	G	0.0518	0.0369	0.0081	5.97E-06	20.7531
M32787.m	rs2174474	T	C	0.7012	0.0327	0.0064	3.67E-07	26.1057
M32787.m	rs2236525	T	C	0.5285	0.016	0.0035	4.02E-06	20.898
M32787.m	rs2596574	A	G	0.0789	0.0277	0.0062	7.49E-06	19.9607
M32787.m	rs2734331	A	G	0.9651	0.0726	0.0101	6.56E-13	51.6691
M32787.m	rs3135377	A	G	0.2078	0.0216	0.0043	4.57E-07	25.2331
M32787.m	rs3740393	C	G	0.1428	-0.0261	0.005	1.59E-07	27.2484
M32787.m	rs6437043	T	C	0.9712	0.0483	0.0109	8.63E-06	19.6355
M32787.m	rs6735065	C	G	0.9541	0.0422	0.0088	1.59E-06	22.9964
M32787.m	rs6762129	T	C	0.3537	0.0163	0.0035	3.06E-06	21.689
M32787.m	rs6929324	T	G	0.0166	-0.0896	0.0151	3.25E-09	35.2097
M32787.m	rs9264915	A	G	0.9803	0.1082	0.0234	3.86E-06	21.3807
M32787.m	rs9267665	T	C	0.0254	-0.0547	0.0118	3.67E-06	21.4887
M32795.m	rs10431893	A	G	0.0582	-0.0454	0.0099	4.92E-06	21.0301
M32795.m	rs11073264	T	C	0.5956	0.0248	0.0054	5.26E-06	21.0919
M32795.m	rs11647589	A	G	0.7186	0.0404	0.0047	1.3E-17	73.8868
M32795.m	rs12187097	A	G	0.1521	0.0295	0.0066	7.11E-06	19.9782
M32795.m	rs17260438	A	G	0.1739	-0.0269	0.0057	2.15E-06	22.2718
M32795.m	rs17429004	A	G	0.91	-0.0367	0.0081	6.65E-06	20.5287
M32795.m	rs17660864	A	G	0.93	0.175	0.0346	4.37E-07	25.5814
M32795.m	rs3729621	A	G	0.9564	0.0503	0.011	4.92E-06	20.9098
M32795.m	rs3750078	A	G	0.5971	0.021	0.0047	6.84E-06	19.9638
M32795.m	rs3791660	T	G	0.463	-0.0214	0.0047	4.49E-06	20.7316
M32795.m	rs6497490	T	G	0.8816	-0.0824	0.0077	4.95E-27	114.518
M32795.m	rs7520193	A	C	0.1862	0.0283	0.0056	4.55E-07	25.5386
M32795.m	rs9277015	A	G	0.358	0.024	0.005	1.59E-06	23.04
M32795.m	rs930609	A	G	0.7886	-0.028	0.0056	4.98E-07	25
M32800.m	rs1025573	T	C	0.0859	0.0558	0.0114	9.52E-07	23.9584
M32800.m	rs11690780	C	G	0.1054	0.051	0.0109	3.1E-06	21.8921
M32800.m	rs11736674	A	C	0.9635	-0.1881	0.0416	6.11E-06	20.4452
M32800.m	rs12000429	A	G	0.0143	0.3394	0.0731	3.49E-06	21.557
M32800.m	rs17057690	T	C	0.0555	0.0831	0.0142	5E-09	34.2472
M32800.m	rs4437633	T	G	0.468	-0.065	0.0138	2.44E-06	22.1855
M32800.m	rs4674860	T	C	0.4516	-0.0284	0.0063	5.74E-06	20.3215
M32800.m	rs6993325	T	C	0.2645	-0.0315	0.0071	8.93E-06	19.6836
M32800.m	rs7299188	C	G	0.6604	-0.0371	0.0082	6.53E-06	20.4701
M32800.m	rs7305915	A	G	0.3824	-0.0307	0.0063	9.85E-07	23.7463
M32800.m	rs9897258	T	C	0.8542	-0.0463	0.0102	6.07E-06	20.6045
M32800.m	rs9953591	A	T	0.0705	0.0681	0.0129	1.4E-07	27.8686
M32802.m	rs1017275	T	C	0.0898	0.0433	0.009	1.5E-06	23.1468
M32802.m	rs10511016	T	C	0.2764	0.0261	0.0058	6.31E-06	20.25
M32802.m	rs11099711	A	C	0.6983	0.0276	0.0057	1.26E-06	23.446
M32802.m	rs1952618	C	G	0.0297	0.1144	0.0258	9.55E-06	19.6613
M32802.m	rs2231142	T	G	0.1112	0.0368	0.0081	6.34E-06	20.6408
M32802.m	rs307944	A	G	0.205	-0.041	0.0091	5.95E-06	20.2995
M32802.m	rs853725	T	C	0.5592	0.044	0.0089	8.62E-07	24.4414
M32808.m	rs10421673	A	G	0.2392	0.0307	0.0068	7.62E-06	20.3826
M32808.m	rs10505869	T	G	0.6625	-0.0307	0.006	2.96E-07	26.1803
M32808.m	rs11045808	C	G	0.068	0.0627	0.0116	6.6E-08	29.2159
M32808.m	rs11045824	T	G	0.163	-0.0639	0.0079	5.08E-16	65.4256
M32808.m	rs12645107	A	G	0.1473	0.0463	0.0079	4.61E-09	34.3485
M32808.m	rs1293988	A	G	0.4973	-0.0527	0.0107	9.39E-07	24.2579
M32808.m	rs1383432	T	G	0.6573	-0.0284	0.006	2.36E-06	22.4044
M32808.m	rs1473993	T	C	0.6241	0.0272	0.006	5.85E-06	20.5511

M32808.m	rs16868884	T	C	0.9702	-0.1646	0.035	2.55E-06	22.1169
M32808.m	rs4149056	T	C	0.8325	-0.0881	0.0075	3.42E-32	137.984
M32808.m	rs4149137	T	C	0.681	0.038	0.0072	1.23E-07	27.8549
M32808.m	rs514659	A	C	0.6737	0.0272	0.006	6.61E-06	20.5511
M32808.m	rs6540767	A	T	0.6977	-0.0276	0.0061	5.05E-06	20.4719
M32808.m	rs6686996	T	C	0.0256	0.0919	0.0184	5.81E-07	24.9457
M32808.m	rs6977159	A	G	0.5935	-0.0285	0.006	2E-06	22.5625
M32808.m	rs7975166	A	G	0.047	-0.0799	0.014	1.23E-08	32.5715
M32808.m	rs9881563	T	C	0.6543	0.0279	0.0061	4.6E-06	20.9194
M32814.m	rs10075995	A	G	0.578	-0.0113	0.0025	8.73E-06	20.4304
M32814.m	rs10224806	T	C	0.8787	0.0194	0.0043	5.64E-06	20.3548
M32814.m	rs1077028	T	C	0.4875	-0.0117	0.0025	3.79E-06	21.9024
M32814.m	rs11630571	A	C	0.4793	0.0115	0.0026	8.05E-06	19.5636
M32814.m	rs1581762	A	G	0.4121	-0.0121	0.0026	3.28E-06	21.6583
M32814.m	rs2433679	A	G	0.8868	0.0213	0.0041	1.65E-07	26.9893
M32814.m	rs2576722	A	T	0.8553	0.0166	0.0037	9.92E-06	20.1286
M32814.m	rs434943	A	G	0.3163	0.0135	0.003	8.57E-06	20.25
M32814.m	rs550214	A	G	0.2053	-0.0154	0.0032	1.78E-06	23.1602
M32814.m	rs697473	T	C	0.3149	-0.0159	0.0036	8.39E-06	19.5069
M32814.m	rs7095335	A	T	0.6604	-0.0141	0.0029	1.11E-06	23.6397
M32814.m	rs7446005	T	G	0.6132	-0.0114	0.0026	8.7E-06	19.2249
M32814.m	rs7531022	A	G	0.505	-0.0115	0.0026	8.75E-06	19.5636
M32814.m	rs9994990	T	G	0.9009	0.0192	0.0043	8.22E-06	19.9373
M32836.m	rs1192325	A	G	0.9158	-0.0419	0.009	3.3E-06	21.6742
M32836.m	rs17150816	A	C	0.0377	-0.0714	0.0143	6.11E-07	24.9301
M32836.m	rs2007084	A	G	0.091	-0.0683	0.0097	1.77E-12	49.579
M32836.m	rs3133745	T	C	0.1259	-0.0396	0.0085	2.71E-06	21.7046
M32836.m	rs4253236	T	C	0.362	-0.037	0.0072	2.61E-07	26.4082
M32836.m	rs7535263	A	G	0.435	-0.0443	0.0079	2.2E-08	31.4451
M32836.m	rs893616	T	C	0.5365	0.0309	0.0053	6.14E-09	33.9911
M32838.m	rs10023223	T	C	0.8808	-0.0256	0.0056	5.58E-06	20.898
M32838.m	rs11134995	T	C	0.3569	0.0177	0.0038	2.96E-06	21.696
M32838.m	rs1171660	A	G	0.6827	-0.0189	0.0038	6.89E-07	24.7375
M32838.m	rs13037679	A	G	0.7782	-0.0217	0.0047	5.01E-06	21.3169
M32838.m	rs16833668	A	C	0.3149	0.0211	0.0038	2.91E-08	30.8317
M32838.m	rs2234185	T	C	0.5191	-0.0169	0.0038	9.33E-06	19.7791
M32838.m	rs3741298	T	C	0.7854	-0.044	0.0097	6.1E-06	20.576
M32838.m	rs4733166	T	C	0.6812	-0.0198	0.0039	2.84E-07	25.7751
M32838.m	rs662138	C	G	0.8314	0.0292	0.0055	1.4E-07	28.1864
M32838.m	rs9318132	A	C	0.2664	-0.021	0.0046	5.27E-06	20.8412
M32838.m	rs981831	T	C	0.123	0.0267	0.0057	2.48E-06	21.9418
M32846.m	rs10107576	C	G	0.1392	0.046	0.0104	8.75E-06	19.5636
M32846.m	rs10505870	A	G	0.2733	0.0654	0.0071	3.13E-20	84.8475
M32846.m	rs10520327	A	G	0.6558	-0.032	0.0069	3.27E-06	21.5081
M32846.m	rs10770803	T	C	0.9492	-0.2129	0.0363	4.43E-09	34.3984
M32846.m	rs10841797	A	G	0.2615	-0.0604	0.0076	2.44E-15	63.1607
M32846.m	rs11029704	T	C	0.1475	-0.0424	0.0093	5.34E-06	20.7858
M32846.m	rs11045824	T	G	0.1583	-0.1715	0.0089	9.91E-83	371.32
M32846.m	rs11190378	T	C	0.5716	0.0353	0.0069	2.74E-07	26.1729
M32846.m	rs11581375	T	C	0.9792	-0.2174	0.0485	7.48E-06	20.0926
M32846.m	rs12533589	T	C	0.2893	0.0309	0.0069	7.82E-06	20.0548
M32846.m	rs12565684	T	C	0.5838	-0.0311	0.007	8.52E-06	19.739
M32846.m	rs12820524	T	C	0.9833	0.1466	0.0289	3.97E-07	25.7319
M32846.m	rs16902795	C	G	0.0266	0.0922	0.0204	6.42E-06	20.4269
M32846.m	rs17617462	T	C	0.9017	0.0827	0.0112	2.01E-13	54.5224

M32846.m	rs1910202	A	G	0.9652	-0.1837	0.0396	3.41E-06	21.5193
M32846.m	rs4149021	A	G	0.0145	0.2311	0.0283	3.05E-16	66.6848
M32846.m	rs4149056	T	C	0.8182	-0.2952	0.0078	1E-200	1432.33
M32846.m	rs4721104	A	T	0.2583	-0.0341	0.0076	6.93E-06	20.1318
M32846.m	rs4762836	T	C	0.0748	0.0584	0.012	1.01E-06	23.6844
M32846.m	rs7137767	A	C	0.5583	0.049	0.0072	7.8E-12	46.3156
M32846.m	rs7138177	A	G	0.8913	-0.0784	0.0103	3.11E-14	57.9372
M32846.m	rs7150683	A	G	0.2063	-0.0379	0.0078	1.13E-06	23.6096
M32846.m	rs7975166	A	G	0.0459	-0.1407	0.016	1.45E-18	77.33
M32846.m	rs8187710	A	G	0.0529	-0.0664	0.0146	5.04E-06	20.6838
M32846.m	rs974453	A	G	0.1015	0.0629	0.0107	4.15E-09	34.5568
M32847.m	rs12401645	A	G	0.9861	0.1345	0.0275	1.03E-06	23.921
M32847.m	rs12457980	A	G	0.6961	-0.0204	0.0046	9.72E-06	19.6673
M32847.m	rs1353050	A	G	0.7319	-0.0208	0.0047	8.93E-06	19.5853
M32847.m	rs17334856	A	G	0.1082	-0.0299	0.0068	9.32E-06	19.3341
M32847.m	rs17461842	T	C	0.9096	-0.0358	0.0076	2.86E-06	22.1891
M32847.m	rs17863762	A	G	0.0291	-0.0851	0.0131	9.1E-11	42.2004
M32847.m	rs17864714	A	G	0.0349	0.0573	0.0129	8.49E-06	19.7301
M32847.m	rs2018609	T	C	0.7438	0.0299	0.0047	2.04E-10	40.4713
M32847.m	rs2831156	A	G	0.1791	-0.0252	0.0053	2.38E-06	22.6073
M32847.m	rs28900385	T	C	0.9662	-0.0996	0.0115	6.2E-18	75.0107
M32847.m	rs361798	C	G	0.9005	0.0447	0.0101	9.27E-06	19.5872
M32847.m	rs4047189	T	G	0.096	0.0555	0.0073	3.78E-14	57.8017
M32847.m	rs4500414	A	G	0.6497	-0.0203	0.0045	7.44E-06	20.3501
M32847.m	rs6496017	A	C	0.5014	0.0196	0.0044	8.49E-06	19.843
M32847.m	rs6716168	T	C	0.6439	-0.0219	0.0044	8.27E-07	24.7732
M32847.m	rs6723936	A	G	0.642	0.0372	0.0045	2.6E-16	68.3378
M32847.m	rs7608713	A	G	0.2387	-0.0483	0.0048	3.59E-24	101.254
M32847.m	rs7707678	A	G	0.4165	0.0198	0.0044	8.1E-06	20.25
M32847.m	rs792222	C	G	0.7494	-0.0224	0.0048	3.59E-06	21.7778
M32847.m	rs838718	A	G	0.486	0.027	0.0044	6.75E-10	37.655
M32847.m	rs887829	T	C	0.3321	0.1116	0.0044	2E-140	643.314
M32854.m	rs10428305	T	C	0.6101	0.0324	0.0064	4.64E-07	25.6289
M32854.m	rs174548	C	G	0.7165	-0.0341	0.007	1.19E-06	23.7308
M32854.m	rs442288	C	G	0.0654	-0.1078	0.0234	3.99E-06	21.223
M32854.m	rs4899909	A	G	0.8983	0.0482	0.0108	7.93E-06	19.918
M32854.m	rs6723705	A	G	0.5871	0.0411	0.0081	3.46E-07	25.7462
M32854.m	rs6763260	T	C	0.6335	0.03	0.0067	7.76E-06	20.049
M32854.m	rs7559623	A	G	0.0545	0.0631	0.0143	9.58E-06	19.4709
M32855.m	rs10505870	A	G	0.2696	0.0298	0.0044	9.16E-12	45.8698
M32855.m	rs10516440	A	G	0.8742	0.0281	0.0059	2.13E-06	22.6834
M32855.m	rs11045824	T	G	0.1664	-0.0953	0.0051	4.34E-79	349.177
M32855.m	rs11231630	T	C	0.0486	0.0443	0.0099	7.48E-06	20.0234
M32855.m	rs11834359	A	C	0.807	0.0349	0.005	2.65E-12	48.7204
M32855.m	rs11982	A	G	0.0691	0.0385	0.0081	2.26E-06	22.5918
M32855.m	rs12228798	T	C	0.2746	-0.0349	0.0043	6.19E-16	65.874
M32855.m	rs12484077	A	T	0.1107	-0.0285	0.0064	9.09E-06	19.8303
M32855.m	rs1481148	A	C	0.9834	-0.1256	0.0282	8.46E-06	19.8372
M32855.m	rs16869851	A	C	0.87	0.0256	0.0058	8.89E-06	19.4816
M32855.m	rs16923154	A	G	0.936	-0.0493	0.0091	5.57E-08	29.3502
M32855.m	rs1871395	A	G	0.8385	-0.1101	0.0055	1.06E-89	400.728
M32855.m	rs1899256	C	G	0.9149	0.0377	0.0073	2.61E-07	26.6709
M32855.m	rs2108622	T	C	0.2918	-0.0248	0.0043	8.49E-09	33.2634
M32855.m	rs2226274	T	C	0.9342	-0.0403	0.0087	3.33E-06	21.4571
M32855.m	rs2291073	T	G	0.9177	0.0464	0.0073	2.46E-10	40.4008

M32855.m	rs2857468	A	T	0.0955	-0.0712	0.0079	1.22E-19	81.228
M32855.m	rs3857224	T	C	0.3195	0.0188	0.0042	6.31E-06	20.0363
M32855.m	rs4149021	A	G	0.0136	0.082	0.0182	6.47E-06	20.2995
M32855.m	rs4762818	A	G	0.1282	0.0267	0.0059	5.45E-06	20.4795
M32855.m	rs755692	A	T	0.2748	0.0232	0.0048	1.09E-06	23.3611
M32855.m	rs7687485	A	T	0.0947	-0.0324	0.0066	8.28E-07	24.0992
M32855.m	rs910923	A	G	0.5988	0.019	0.0041	3.9E-06	21.4753
M32857.m	rs10428305	T	C	0.6105	0.0309	0.0064	1.38E-06	23.3108
M32857.m	rs11761138	A	T	0.6277	-0.0301	0.0067	6.27E-06	20.1829
M32857.m	rs1578963	C	G	0.0342	0.0846	0.0178	1.91E-06	22.5892
M32857.m	rs498275	A	G	0.3246	0.0338	0.0069	9.93E-07	23.9958
M32857.m	rs6564580	T	C	0.3228	0.0365	0.0082	7.68E-06	19.8134
M32857.m	rs671321	T	G	0.5978	0.0305	0.0066	4.01E-06	21.3556
M32857.m	rs6747014	T	C	0.8967	0.048	0.0103	3.12E-06	21.7174
M32857.m	rs7129619	C	G	0.6449	0.0297	0.0066	7.25E-06	20.25
M32863.m	rs10859956	T	C	0.7233	-0.0522	0.0117	8E-06	19.9053
M32863.m	rs11177672	T	C	0.1109	0.0743	0.0165	7.01E-06	20.2773
M32863.m	rs12479945	T	G	0.9236	-0.0918	0.0197	3.39E-06	21.7147
M32863.m	rs12893753	T	C	0.9005	0.1566	0.0296	1.26E-07	27.9898
M32863.m	rs17483163	A	G	0.6475	-0.0544	0.0109	6.58E-07	24.9083
M32863.m	rs17700286	C	G	0.5903	0.0617	0.0129	1.77E-06	22.8766
M32863.m	rs2022010	T	G	0.9217	-0.1119	0.0252	9.01E-06	19.7178
M32863.m	rs213190	T	G	0.5931	0.0529	0.0112	2.36E-06	22.3088
M32863.m	rs2714775	T	C	0.9695	-0.1526	0.0317	1.52E-06	23.1734
M32863.m	rs4447447	A	G	0.0449	0.2288	0.0485	2.36E-06	22.255
M32863.m	rs465818	T	C	0.963	-0.2195	0.045	1.07E-06	23.7927
M32863.m	rs7539884	T	C	0.749	0.0623	0.0133	2.67E-06	21.9418
M32863.m	rs7856961	A	G	0.1517	-0.0778	0.0164	2.15E-06	22.5046
M32863.m	rs7961980	C	G	0.6612	-0.0493	0.011	6.83E-06	20.0867
M32863.m	rs982503	T	C	0.8001	0.0667	0.0141	2.21E-06	22.3776
M32867.m	rs10492157	T	C	0.7656	0.0065	0.0014	1.92E-06	21.5561
M32867.m	rs1109583	A	G	0.235	-0.0068	0.0014	8.7E-07	23.5918
M32867.m	rs11117195	A	G	0.4944	0.0058	0.0013	5.38E-06	19.9053
M32867.m	rs11181433	A	G	0.8492	-0.0078	0.0018	9.95E-06	18.7778
M32867.m	rs12452541	A	T	0.8568	-0.01	0.0019	7.64E-08	27.7008
M32867.m	rs13161544	T	C	0.5812	-0.0057	0.0012	4.84E-06	22.5625
M32867.m	rs1967908	T	C	0.4168	-0.0056	0.0013	8.98E-06	18.5562
M32867.m	rs2039906	C	G	0.2696	0.0084	0.0018	1.94E-06	21.7778
M32867.m	rs2251468	A	C	0.6513	-0.0059	0.0013	3.43E-06	20.5976
M32867.m	rs2370933	A	G	0.3578	0.0073	0.0016	2.85E-06	20.8164
M32867.m	rs247616	T	C	0.3242	0.0096	0.0014	1.11E-11	47.0204
M32867.m	rs2626332	A	C	0.1274	0.0085	0.0019	4.88E-06	20.0139
M32867.m	rs309575	A	C	0.3321	-0.0058	0.0013	6.15E-06	19.9053
M32867.m	rs6707419	A	G	0.3273	-0.0062	0.0014	5.37E-06	19.6122
M32867.m	rs7037165	A	G	0.0124	0.0277	0.0058	2.03E-06	22.8089
M32867.m	rs770707	A	G	0.7024	-0.0058	0.0013	7.56E-06	19.9053
M32867.m	rs7829432	T	C	0.346	0.0063	0.0013	1.14E-06	23.4852
M32867.m	rs804854	A	T	0.1417	0.0083	0.0018	2.68E-06	21.2623
M32867.m	rs862226	T	G	0.2555	0.0068	0.0013	4.34E-07	27.3609
M32867.m	rs894282	A	T	0.0899	0.013	0.0026	9.04E-07	25
M32867.m	rs931761	C	G	0.755	0.0064	0.0014	2.8E-06	20.898
M32869.m	rs10511991	A	T	0.2723	0.0245	0.0055	7.54E-06	19.843
M32869.m	rs11136430	T	C	0.6198	0.0231	0.0052	8.45E-06	19.7341
M32869.m	rs11636906	A	C	0.0306	0.0623	0.0133	2.84E-06	21.9418
M32869.m	rs11852692	T	C	0.934	-0.0446	0.0099	6.41E-06	20.2955

M32869.m	rs12137475	T	C	0.1187	0.0359	0.008	6.92E-06	20.1377
M32869.m	rs13265102	T	C	0.6244	0.0271	0.0059	4.73E-06	21.0977
M32869.m	rs2071427	T	C	0.2568	0.0304	0.0069	9.14E-06	19.411
M32869.m	rs2324594	T	C	0.1825	-0.0357	0.0075	2.16E-06	22.6576
M32869.m	rs2501628	A	C	0.3167	0.0299	0.0064	2.85E-06	21.8264
M32869.m	rs2732573	T	C	0.2225	-0.0247	0.0056	0.000009	19.4544
M32869.m	rs2899743	C	G	0.6897	-0.0261	0.0056	3.79E-06	21.7223
M32869.m	rs3737137	T	C	0.233	-0.0306	0.0068	7.99E-06	20.25
M32869.m	rs4377848	A	G	0.1394	0.0307	0.0068	6.19E-06	20.3826
M32869.m	rs671307	A	G	0.1553	0.0319	0.0065	7.95E-07	24.0854
M32869.m	rs6769112	T	C	0.2973	0.0251	0.0051	7.28E-07	24.2218
M32869.m	rs6912503	A	G	0.8251	-0.0311	0.0062	6.16E-07	25.1616
M32869.m	rs7828843	T	C	0.0202	-0.1059	0.0234	6.17E-06	20.4814
M32910.m	rs10058247	T	C	0.4208	-0.0078	0.0017	5.47E-06	21.0519
M32910.m	rs1012157	A	G	0.2326	-0.0125	0.0024	1.75E-07	27.1267
M32910.m	rs10162454	T	C	0.2685	-0.008	0.0018	6.66E-06	19.7531
M32910.m	rs10412803	T	C	0.3119	0.0097	0.0018	4.42E-08	29.0401
M32910.m	rs11089325	T	C	0.0613	0.0227	0.004	1.19E-08	32.2056
M32910.m	rs11485264	A	G	0.8872	-0.0119	0.0026	6.74E-06	20.9482
M32910.m	rs11541479	A	G	0.152	-0.014	0.003	3.06E-06	21.7778
M32910.m	rs11949401	T	C	0.686	-0.0079	0.0017	6.79E-06	21.5952
M32910.m	rs1424092	T	G	0.3648	-0.0092	0.0018	1.78E-07	26.1235
M32910.m	rs1480792	A	G	0.3268	0.0084	0.0018	1.84E-06	21.7778
M32910.m	rs1540603	A	C	0.0122	-0.0676	0.015	7E-06	20.31
M32910.m	rs1558688	T	C	0.2928	-0.0089	0.0018	4.34E-07	24.4475
M32910.m	rs1566481	A	G	0.6896	0.0083	0.0019	7.7E-06	19.0831
M32910.m	rs16844839	T	C	0.1846	-0.0113	0.0025	5E-06	20.4304
M32910.m	rs16982844	A	C	0.0627	-0.0423	0.0069	9.08E-10	37.5822
M32910.m	rs1919817	T	C	0.8755	-0.0241	0.0054	7.02E-06	19.918
M32910.m	rs1921984	T	C	0.5754	-0.0085	0.0017	9E-07	25
M32910.m	rs1981409	A	T	0.6036	-0.0086	0.0018	9.93E-07	22.8272
M32910.m	rs1989843	A	G	0.6058	0.0091	0.0018	2.37E-07	25.5586
M32910.m	rs2009217	T	C	0.7257	-0.0081	0.0018	4.91E-06	20.25
M32910.m	rs2188839	A	G	0.8722	0.0115	0.0026	7.47E-06	19.5636
M32910.m	rs2205212	A	G	0.6925	0.0078	0.0018	9.37E-06	18.7778
M32910.m	rs222836	A	G	0.5336	-0.0082	0.0018	3.9E-06	20.7531
M32910.m	rs2295465	A	C	0.8798	-0.0118	0.0026	6.65E-06	20.5976
M32910.m	rs230966	A	G	0.7247	0.0084	0.0018	1.85E-06	21.7778
M32910.m	rs2382376	T	C	0.3053	0.0081	0.0018	6.5E-06	20.25
M32910.m	rs2686184	A	G	0.408	-0.0097	0.0017	1.89E-08	32.5571
M32910.m	rs282145	T	C	0.6353	0.0087	0.0018	7.18E-07	23.3611
M32910.m	rs2904562	A	T	0.9187	0.0159	0.0035	4.67E-06	20.6376
M32910.m	rs2915754	T	G	0.6997	-0.0079	0.0018	5.94E-06	19.2623
M32910.m	rs350729	T	G	0.313	-0.0095	0.0018	6.9E-08	27.8549
M32910.m	rs3804043	A	C	0.2911	0.0178	0.0018	7.76E-24	97.7901
M32910.m	rs3856584	T	C	0.3934	0.0078	0.0017	6.62E-06	21.0519
M32910.m	rs3887893	T	C	0.617	0.009	0.002	6.8E-06	20.25
M32910.m	rs4084164	A	G	0.3942	0.0081	0.0018	4.04E-06	20.25
M32910.m	rs417309	A	G	0.0822	-0.0167	0.0034	9.11E-07	24.1254
M32910.m	rs438798	A	G	0.5346	0.0173	0.0017	1.55E-23	103.561
M32910.m	rs442100	A	G	0.4283	-0.008	0.0018	6.5E-06	19.7531
M32910.m	rs4597638	T	C	0.942	-0.0281	0.0043	6.07E-11	42.7047
M32910.m	rs4680	A	G	0.5069	-0.0486	0.0017	4.7E-178	817.287
M32910.m	rs4846728	T	C	0.2874	-0.0079	0.0018	8.14E-06	19.2623
M32910.m	rs4867926	T	G	0.3914	0.0077	0.0017	8.29E-06	20.5156

M32910.m	rs4983559	A	G	0.6109	-0.0087	0.0018	1.35E-06	23.3611
M32910.m	rs5019028	T	G	0.7021	0.0085	0.0018	2.27E-06	22.2994
M32910.m	rs549114	A	G	0.3334	0.0086	0.0017	7.57E-07	25.5917
M32910.m	rs6824211	A	G	0.3182	-0.0091	0.0017	2.16E-07	28.654
M32910.m	rs696885	T	G	0.4104	-0.009	0.0018	5.82E-07	25
M32910.m	rs7255053	A	C	0.6266	0.0078	0.0017	6.36E-06	21.0519
M32910.m	rs7290062	T	C	0.8635	-0.0168	0.0025	2.24E-11	45.1584
M32910.m	rs7589568	T	C	0.8796	-0.0123	0.0027	5.65E-06	20.7531
M32910.m	rs7707010	A	G	0.9582	0.0252	0.0044	9.61E-09	32.8017
M32910.m	rs7748892	A	G	0.9721	0.0287	0.0057	4.3E-07	25.3521
M32910.m	rs8038921	A	C	0.1345	0.0117	0.0025	4.67E-06	21.9024
M32910.m	rs8180826	C	G	0.6944	-0.008	0.0018	5.21E-06	19.7531
M32910.m	rs866142	A	G	0.605	0.008	0.0017	3.79E-06	22.1453
M32910.m	rs887200	T	C	0.8808	-0.0294	0.0026	8.03E-30	127.864
M32910.m	rs9315310	T	C	0.1535	0.012	0.0025	1.84E-06	23.04
M32910.m	rs9318225	T	C	0.6829	-0.0106	0.0018	1.95E-09	34.679
M32910.m	rs9371494	A	G	0.6805	0.0078	0.0017	7.38E-06	21.0519
M32910.m	rs9558480	A	G	0.6649	-0.0165	0.0037	7.84E-06	19.8868
M32910.m	rs9606212	A	C	0.0586	-0.0318	0.005	2.67E-10	40.4496
M32980.m	rs11206335	A	G	0.9237	-0.0268	0.006	6.72E-06	19.9511
M32980.m	rs11620434	A	C	0.9383	0.033	0.0074	7.31E-06	19.8868
M32980.m	rs12359572	T	G	0.2419	0.0177	0.0036	6.26E-07	24.1736
M32980.m	rs12531203	T	C	0.2025	-0.0162	0.0036	7.24E-06	20.25
M32980.m	rs13192201	A	T	0.7555	0.0165	0.0035	2.34E-06	22.2245
M32980.m	rs1689955	T	C	0.4884	0.0258	0.0056	4.29E-06	21.2258
M32980.m	rs17203317	T	G	0.9652	-0.0739	0.0166	8.81E-06	19.8186
M32980.m	rs174550	T	C	0.6676	0.0347	0.0034	6.79E-24	104.16
M32980.m	rs2521567	A	G	0.3879	-0.0154	0.0034	6.5E-06	20.5156
M32980.m	rs6006250	T	C	0.0613	-0.0532	0.0118	6.49E-06	20.3263
M32980.m	rs6987121	A	G	0.9067	-0.0263	0.0053	7.59E-07	24.6241
M32980.m	rs831471	A	G	0.4088	-0.0162	0.0036	6.44E-06	20.25
M33009.m	rs10192021	T	G	0.0878	-0.0513	0.0114	7.46E-06	20.25
M33009.m	rs11999484	A	G	0.8305	0.0384	0.0085	6.47E-06	20.4091
M33009.m	rs12630300	T	C	0.0603	0.0711	0.0148	1.65E-06	23.0789
M33009.m	rs7838814	T	G	0.9497	-0.0764	0.0167	4.85E-06	20.9293
M33009.m	rs8008553	A	G	0.7132	0.0333	0.0072	3.98E-06	21.3906
M33009.m	rs9635387	A	G	0.5273	0.0324	0.0069	2.72E-06	22.0491
M33009.x	rs11749272	T	C	0.3113	0.0235	0.0052	6.36E-06	20.4234
M33009.x	rs11950562	A	C	0.5264	0.0297	0.0046	6.75E-11	41.6867
M33009.x	rs11966463	A	C	0.9515	-0.0504	0.0109	4.19E-06	21.38
M33009.x	rs12356120	A	G	0.6829	-0.0256	0.0052	8.08E-07	24.2367
M33009.x	rs12630300	T	C	0.0603	0.0711	0.0148	1.65E-06	23.0789
M33009.x	rs1439816	C	G	0.1288	0.0325	0.0072	6.89E-06	20.3752
M33009.x	rs17171380	A	G	0.9131	-0.0426	0.0081	1.56E-07	27.6598
M33009.x	rs2224851	T	C	0.7705	-0.0238	0.0054	8.77E-06	19.4252
M33009.x	rs2285972	T	C	0.6696	-0.0247	0.0053	3.77E-06	21.7191
M33009.x	rs2749920	C	G	0.011	0.156	0.035	8.31E-06	19.8661
M33009.x	rs351584	A	G	0.1393	0.0309	0.0067	4.16E-06	21.27
M33009.x	rs4620231	A	C	0.8136	-0.0266	0.0059	6.59E-06	20.3263
M33009.x	rs4682430	A	T	0.5944	0.0222	0.005	8.95E-06	19.7136
M33009.x	rs4706020	A	G	0.3838	-0.0277	0.0052	1.08E-07	28.3761
M33009.x	rs4838816	A	G	0.1347	0.0329	0.007	2.17E-06	22.09
M33009.x	rs6673836	A	G	0.2903	-0.0248	0.0053	2.55E-06	21.8953
M33009.x	rs6878253	A	G	0.0861	0.047	0.0083	1.37E-08	32.0656
M33009.x	rs725201	T	G	0.5593	-0.0209	0.0046	5.16E-06	20.6432

M33009.x	rs8111253	T	C	0.4988	-0.0238	0.0053	8.16E-06	20.1652
M33009.x	rs9635387	A	G	0.5273	0.0324	0.0069	2.72E-06	22.0491
M33084.m	rs2073827	C	G	0.4127	0.0224	0.0046	1.17E-06	23.7127
M33084.m	rs2324913	T	C	0.12	-0.0411	0.0088	3.31E-06	21.8131
M33084.m	rs2731672	T	C	0.257	-0.081	0.011	1.79E-13	54.2231
M33084.m	rs3793499	C	G	0.8598	-0.0306	0.0067	5.06E-06	20.859
M33084.m	rs4253236	T	C	0.362	-0.071	0.01	1.25E-12	50.41
M33084.m	rs6029632	A	G	0.3782	0.0223	0.0047	2.6E-06	22.512
M33084.m	rs6433823	T	C	0.0203	0.1124	0.0253	8.78E-06	19.7375
M33084.m	rs6480159	A	G	0.2212	0.0255	0.0056	5.33E-06	20.735
M33084.m	rs6496511	C	G	0.8711	-0.0336	0.0076	9.81E-06	19.5457
M33084.m	rs7309973	T	C	0.0502	-0.0701	0.014	5.85E-07	25.0715
M33084.m	rs786272	A	G	0.6761	0.0228	0.0049	3.02E-06	21.651
M33131.m	rs11593247	A	G	0.937	-0.0494	0.0101	1.07E-06	23.9228
M33131.m	rs11633642	A	G	0.3194	-0.0296	0.0063	2.35E-06	22.0751
M33131.m	rs11708412	A	G	0.2657	0.0259	0.0058	8.74E-06	19.9408
M33131.m	rs12491685	C	G	0.7659	-0.0368	0.0074	7.13E-07	24.7305
M33131.m	rs12565464	A	G	0.8419	0.034	0.0069	8.34E-07	24.2806
M33131.m	rs12916428	A	G	0.2058	-0.0274	0.0061	8.06E-06	20.1763
M33131.m	rs2197598	T	C	0.1051	-0.0408	0.0087	2.76E-06	21.9929
M33131.m	rs2408393	T	C	0.866	-0.0403	0.0088	5.01E-06	20.9722
M33131.m	rs2501556	A	G	0.522	-0.0237	0.0051	3.48E-06	21.5952
M33131.m	rs4245188	A	G	0.9485	-0.0528	0.012	9.96E-06	19.36
M33131.m	rs4363506	T	C	0.5678	0.0245	0.0049	6.01E-07	25
M33131.m	rs4798177	A	T	0.3125	0.0236	0.0053	7.11E-06	19.8277
M33131.m	rs7061710	C	G	0.2282	0.0453	0.0061	7.52E-14	55.1489
M33132.m	rs10476724	T	C	0.8453	0.0078	0.0018	9.43E-06	18.7778
M33132.m	rs10920716	A	G	0.8234	-0.0178	0.0038	2.27E-06	21.9418
M33132.m	rs11073472	A	G	0.1492	-0.0092	0.0018	5.55E-07	26.1235
M33132.m	rs11188044	A	G	0.8337	-0.0095	0.0018	6.87E-08	27.8549
M33132.m	rs11572106	A	G	0.9861	0.0766	0.0165	3.48E-06	21.5521
M33132.m	rs11598738	A	G	0.212	0.0085	0.0017	7.22E-07	25
M33132.m	rs11813961	A	G	0.0801	-0.0119	0.0027	9.12E-06	19.4252
M33132.m	rs12104774	T	G	0.0562	-0.0178	0.0033	5.71E-08	29.0946
M33132.m	rs12902975	T	C	0.9093	-0.0115	0.0025	4.19E-06	21.16
M33132.m	rs17009399	C	G	0.0582	-0.0359	0.0027	2.8E-41	176.791
M33132.m	rs17044007	T	G	0.074	-0.014	0.003	3.06E-06	21.7778
M33132.m	rs17110031	T	C	0.9615	0.035	0.0034	1.1E-24	105.969
M33132.m	rs17168815	T	G	0.134	-0.0084	0.0018	3.68E-06	21.7778
M33132.m	rs17349853	T	G	0.9301	-0.0157	0.0028	1.58E-08	31.4401
M33132.m	rs17423838	A	G	0.8836	0.0086	0.0018	3.06E-06	22.8272
M33132.m	rs17490468	A	G	0.0364	-0.0173	0.0036	1.22E-06	23.0934
M33132.m	rs17595027	T	C	0.1176	-0.008	0.0017	4.76E-06	22.1453
M33132.m	rs17718517	T	C	0.77	0.009	0.002	6.8E-06	20.25
M33132.m	rs17725971	A	G	0.8492	0.0145	0.0018	3.68E-16	64.892
M33132.m	rs1891071	T	C	0.6279	-0.0101	0.0016	1.03E-09	39.8477
M33132.m	rs1927544	T	C	0.8475	0.0078	0.0018	9.67E-06	18.7778
M33132.m	rs1934963	T	C	0.8021	0.0293	0.0017	8.69E-65	297.055
M33132.m	rs2009767	T	C	0.8814	0.0092	0.0018	4.14E-07	26.1235
M33132.m	rs2160166	T	C	0.1848	0.0077	0.0017	8.01E-06	20.5156
M33132.m	rs2298037	T	C	0.1673	0.01	0.0018	1.2E-08	30.8642
M33132.m	rs2924762	T	C	0.8777	0.0086	0.0018	2.25E-06	22.8272
M33132.m	rs4521827	A	T	0.1344	0.0085	0.0018	2.17E-06	22.2994
M33132.m	rs4900819	T	C	0.8744	-0.0097	0.0018	6.88E-08	29.0401
M33132.m	rs6507259	T	C	0.1333	-0.0078	0.0018	9.52E-06	18.7778

M33132.m	rs666276	T	C	0.1213	-0.0094	0.0018	2.19E-07	27.2716
M33132.m	rs6969691	A	G	0.8775	-0.0083	0.0018	5.29E-06	21.2623
M33132.m	rs739626	A	C	0.0935	0.013	0.0024	7.96E-08	29.3403
M33132.m	rs7794651	T	C	0.1191	0.0089	0.0018	7.66E-07	24.4475
M33132.m	rs7921420	A	G	0.716	-0.0084	0.0017	7.21E-07	24.4152
M33132.m	rs7992587	T	C	0.2605	-0.0083	0.0017	7.72E-07	23.8374
M33132.m	rs869927	A	G	0.1385	-0.0112	0.0018	3.51E-10	38.716
M33132.m	rs932748	T	G	0.878	0.009	0.002	6.8E-06	20.25
M33132.m	rs9686531	T	C	0.119	0.0081	0.0018	7.74E-06	20.25
M33132.m	rs994713	T	C	0.0245	-0.0204	0.0045	6.37E-06	20.5511
M33137.m	rs11233729	T	C	0.0578	-0.0902	0.0196	4.06E-06	21.1788
M33137.m	rs12464503	T	C	0.167	0.0658	0.0147	7.1E-06	20.0363
M33137.m	rs12632457	T	C	0.6341	0.0459	0.0096	1.93E-06	22.8604
M33137.m	rs12659061	A	G	0.1376	0.0618	0.0138	7.46E-06	20.0548
M33137.m	rs13077305	T	C	0.6692	-0.0553	0.0119	3.55E-06	21.5952
M33137.m	rs2731672	T	C	0.257	-0.12	0.02	1.97E-09	36
M33137.m	rs2926419	T	C	0.2092	-0.0551	0.0111	7.12E-07	24.6409
M33137.m	rs2937754	T	C	0.8251	-0.0597	0.0116	2.91E-07	26.487
M33137.m	rs4253252	T	G	0.4803	-0.0862	0.0089	2.83E-22	93.8068
M33137.m	rs4540481	T	C	0.2868	-0.0459	0.0101	5.04E-06	20.653
M33137.m	rs4581716	A	G	0.6695	0.049	0.0106	3.69E-06	21.3688
M33137.m	rs6534104	C	G	0.0373	-0.1087	0.024	6.15E-06	20.5134
M33137.m	rs6835379	A	G	0.5036	0.0439	0.0093	2.16E-06	22.2825
M33137.m	rs698078	A	G	0.5789	-0.0636	0.0111	1.02E-08	32.8298
M33137.m	rs929493	T	C	0.837	0.0575	0.0129	8.68E-06	19.8681
M33138.m	rs10175949	A	G	0.8649	-0.0337	0.0056	2.23E-09	36.2146
M33138.m	rs10849603	A	T	0.171	-0.0227	0.005	6.51E-06	20.6116
M33138.m	rs1115381	T	C	0.2625	-0.0333	0.0043	5.59E-15	59.9724
M33138.m	rs11563248	T	G	0.4486	-0.0212	0.0042	6.18E-07	25.4785
M33138.m	rs11563251	T	C	0.1081	-0.0342	0.0062	4.07E-08	30.4277
M33138.m	rs11683356	A	G	0.0287	0.0718	0.0128	2.22E-08	31.4651
M33138.m	rs1188542	T	C	0.605	-0.0182	0.0041	7.97E-06	19.7049
M33138.m	rs12479240	T	C	0.1686	-0.0287	0.0051	2.11E-08	31.6682
M33138.m	rs12500784	A	T	0.9481	-0.0395	0.0086	4.76E-06	21.0959
M33138.m	rs13212376	T	G	0.0741	-0.0333	0.0073	4.61E-06	20.8086
M33138.m	rs13383300	C	G	0.9721	0.056	0.0117	1.66E-06	22.9089
M33138.m	rs1398968	A	G	0.6243	0.0182	0.0041	9.59E-06	19.7049
M33138.m	rs17685204	T	C	0.9177	0.0333	0.0074	6.83E-06	20.25
M33138.m	rs17751533	T	C	0.125	-0.0259	0.0058	7.5E-06	19.9408
M33138.m	rs17863762	A	G	0.0289	-0.0552	0.0123	7.21E-06	20.1404
M33138.m	rs28899194	T	C	0.0345	0.072	0.011	5.04E-11	42.843
M33138.m	rs3779259	T	G	0.0881	0.0308	0.0066	2.98E-06	21.7778
M33138.m	rs3923630	A	G	0.333	-0.028	0.005	2.14E-08	31.36
M33138.m	rs4363657	T	C	0.8302	-0.0239	0.005	1.48E-06	22.8484
M33138.m	rs6760588	A	C	0.7477	0.03	0.0043	2.92E-12	48.675
M33138.m	rs7916009	A	G	0.6156	0.0193	0.0042	3.92E-06	21.1162
M33138.m	rs838705	A	G	0.6163	0.0227	0.0042	4.47E-08	29.2115
M33138.m	rs879665	A	G	0.1323	0.0268	0.0058	3.86E-06	21.3508
M33138.m	rs887829	T	C	0.3275	0.0932	0.0041	3E-114	516.731
M33138.m	rs894137	A	G	0.2902	-0.0192	0.0042	4.99E-06	20.898
M33138.m	rs9861865	C	G	0.3202	-0.0195	0.0042	2.79E-06	21.5561
M33140.m	rs10033353	T	G	0.3189	-0.0154	0.0035	8.89E-06	19.36
M33140.m	rs1039444	T	C	0.8895	0.0231	0.005	4.76E-06	21.3444
M33140.m	rs11563191	C	G	0.0722	0.0299	0.0066	5.34E-06	20.5236
M33140.m	rs12893360	A	G	0.7444	-0.0164	0.0035	2.28E-06	21.9559

M33140.m	rs17127410	A	G	0.8728	0.0246	0.005	7.54E-07	24.2064
M33140.m	rs3844297	T	C	0.5092	-0.0149	0.0033	7.1E-06	20.3866
M33140.m	rs429020	A	G	0.0511	-0.0365	0.0077	2.01E-06	22.4701
M33140.m	rs6948511	T	C	0.9206	-0.028	0.006	3.3E-06	21.7778
M33140.m	rs7589832	A	C	0.8207	-0.0213	0.0041	2.08E-07	26.9893
M33140.m	rs798007	T	C	0.9676	-0.0407	0.009	6.32E-06	20.4505
M33140.m	rs8058442	T	C	0.9738	-0.0474	0.0106	8.1E-06	19.9961
M33140.m	rs8089236	T	C	0.7336	-0.0162	0.0034	2.74E-06	22.7024
M33144.m	rs10148857	T	G	0.7171	-0.0421	0.0093	6.13E-06	20.4927
M33144.m	rs1843486	T	G	0.0517	0.088	0.0185	1.97E-06	22.6267
M33144.m	rs2269606	T	C	0.778	-0.0437	0.0098	8.61E-06	19.8843
M33144.m	rs294095	A	G	0.1424	-0.086	0.0185	3.18E-06	21.6099
M33144.m	rs3099557	A	G	0.8504	-0.1484	0.0113	1.83E-39	172.469
M33144.m	rs3916092	A	G	0.2029	0.0511	0.01	3.22E-07	26.1121
M33144.m	rs4364244	A	G	0.7874	-0.0463	0.0103	7.29E-06	20.2063
M33144.m	rs6498426	T	C	0.601	0.0409	0.0088	3.39E-06	21.6014
M33144.m	rs835136	A	G	0.1289	-0.0544	0.0123	9.87E-06	19.5608
M33150.m	rs11207154	C	G	0.0118	0.3171	0.0703	6.5E-06	20.3461
M33150.m	rs12640474	T	C	0.3034	-0.04	0.0088	5.88E-06	20.6612
M33150.m	rs12787375	T	G	0.0163	-0.1486	0.0333	7.9E-06	19.9136
M33150.m	rs13025486	A	G	0.977	0.205	0.045	5.23E-06	20.7531
M33150.m	rs16868779	A	G	0.8835	0.0725	0.0163	8.56E-06	19.7834
M33150.m	rs17022724	T	G	0.0822	-0.0716	0.0141	3.87E-07	25.7862
M33150.m	rs17093348	A	G	0.0344	-0.1043	0.0225	3.47E-06	21.4884
M33150.m	rs2139212	T	C	0.5848	0.0372	0.0084	9.63E-06	19.6122
M33150.m	rs2667615	C	G	0.0264	-0.1185	0.0262	5.89E-06	20.4566
M33150.m	rs2964195	C	G	0.0511	-0.0833	0.0188	9.03E-06	19.6324
M33150.m	rs7325308	T	C	0.3593	0.0422	0.0091	3.46E-06	21.5051
M33163.m	rs10762231	A	G	0.3787	-0.0138	0.0026	1.68E-07	28.1716
M33163.m	rs10910427	T	C	0.4474	-0.0122	0.0026	3.92E-06	22.0178
M33163.m	rs10932835	A	G	0.5337	0.0121	0.0026	3.86E-06	21.6583
M33163.m	rs11107328	C	G	0.9536	0.036	0.0071	3.7E-07	25.7092
M33163.m	rs11848784	A	T	0.2102	-0.0161	0.0035	3.16E-06	21.16
M33163.m	rs12211179	A	G	0.2158	0.0158	0.0034	4.57E-06	21.5952
M33163.m	rs12467106	A	G	0.1697	0.0188	0.0035	8.38E-08	28.8522
M33163.m	rs1336659	A	G	0.5079	0.0135	0.0026	3.01E-07	26.9601
M33163.m	rs239430	A	G	0.7871	-0.0153	0.0034	8.78E-06	20.25
M33163.m	rs2448148	T	C	0.6465	-0.0258	0.0056	4.39E-06	21.2258
M33163.m	rs2454238	T	C	0.5093	0.0241	0.0054	9.67E-06	19.918
M33163.m	rs2463492	T	G	0.4249	0.0118	0.0026	6.95E-06	20.5976
M33163.m	rs3886930	T	C	0.6	0.0117	0.0026	9.18E-06	20.25
M33163.m	rs4130215	A	C	0.1106	0.0238	0.0049	1.32E-06	23.5918
M33163.m	rs4453360	A	G	0.4648	-0.012	0.0026	4.28E-06	21.3018
M33163.m	rs7146383	T	C	0.0124	0.1237	0.0261	2.15E-06	22.4625
M33163.m	rs7652500	A	G	0.2498	0.0151	0.0034	7.41E-06	19.724
M33163.m	rs896401	T	C	0.7318	-0.0162	0.0035	3.43E-06	21.4237
M33163.m	rs9472173	T	C	0.4953	0.0121	0.0027	8.68E-06	20.0837
M33165.m	rs1012601	C	G	0.8372	0.056	0.0111	4.87E-07	25.4525
M33165.m	rs10765737	A	G	0.6388	-0.0197	0.0044	8.25E-06	20.046
M33165.m	rs10941535	A	G	0.6584	0.0401	0.0089	6.29E-06	20.3006
M33165.m	rs163027	A	G	0.5505	0.0169	0.0037	4.45E-06	20.8627
M33165.m	rs1917331	A	T	0.8179	0.0211	0.0047	5.58E-06	20.1544
M33165.m	rs2298423	T	G	0.9286	0.0761	0.0157	1.24E-06	23.4947
M33165.m	rs4322991	A	C	0.2227	0.0206	0.0046	7.7E-06	20.0548
M33165.m	rs439401	T	C	0.351	-0.034	0.0041	1.22E-16	68.7686

M33165.m	rs445925	A	G	0.079	0.065	0.0082	2.46E-15	62.8346
M33165.m	rs7648714	A	G	0.4717	0.018	0.0037	1.06E-06	23.6669
M33165.m	rs7762480	A	T	0.9273	0.0748	0.0165	5.69E-06	20.5511
M33165.m	rs7943834	C	G	0.9792	0.0639	0.0135	2.14E-06	22.4044
M33165.m	rs9650602	T	C	0.197	-0.0238	0.0052	4.43E-06	20.9482
M33165.m	rs986611	C	G	0.6362	-0.0169	0.0037	5.62E-06	20.8627
M33173.x	rs10848757	A	C	0.0197	-0.9526	0.1628	4.85E-09	34.2383
M33173.x	rs11082714	A	G	0.9531	0.3479	0.0726	1.68E-06	22.9634
M33173.x	rs11137198	A	G	0.0263	-0.7694	0.1466	1.53E-07	27.5446
M33173.x	rs11724451	T	C	0.0288	-0.682	0.1543	9.85E-06	19.5361
M33173.x	rs11778790	T	G	0.9856	0.9754	0.2162	6.45E-06	20.3542
M33173.x	rs12426351	T	G	0.0132	-1.192	0.1953	1.04E-09	37.2519
M33173.x	rs12516510	A	T	0.0973	0.1608	0.0353	5.33E-06	20.7502
M33173.x	rs12531802	T	C	0.0263	-0.6855	0.1522	6.65E-06	20.2855
M33173.x	rs12544572	C	G	0.0921	-0.3743	0.0802	3.07E-06	21.7817
M33173.x	rs1263280	T	C	0.0407	-0.783	0.1498	1.73E-07	27.3212
M33173.x	rs12784492	A	T	0.9821	0.9912	0.1968	4.76E-07	25.3672
M33173.x	rs12792005	A	G	0.0653	-0.2024	0.0439	4.01E-06	21.2565
M33173.x	rs12797812	T	C	0.0291	-0.7626	0.1576	1.31E-06	23.4143
M33173.x	rs12960835	T	C	0.0132	-1.1492	0.1988	7.4E-09	33.4163
M33173.x	rs13071058	A	G	0.9863	0.9598	0.2122	6.09E-06	20.4584
M33173.x	rs162882	C	G	0.9145	0.4501	0.0874	2.57E-07	26.5213
M33173.x	rs16892783	C	G	0.04	-0.6355	0.1216	1.73E-07	27.3127
M33173.x	rs16964920	A	G	0.0159	-0.9595	0.2128	6.54E-06	20.3304
M33173.x	rs17022000	A	G	0.0339	-0.7015	0.1313	9.16E-08	28.5447
M33173.x	rs17061716	C	G	0.8406	0.3081	0.0679	5.62E-06	20.5894
M33173.x	rs17095379	A	G	0.9671	0.5603	0.1119	5.45E-07	25.0715
M33173.x	rs1806077	A	G	0.0205	-0.4253	0.0889	1.73E-06	22.8869
M33173.x	rs1911951	A	C	0.934	0.203	0.043	2.35E-06	22.2872
M33173.x	rs1923325	T	C	0.9478	0.5416	0.1117	1.26E-06	23.5099
M33173.x	rs265795	C	G	0.1061	-0.2845	0.0613	3.51E-06	21.5399
M33173.x	rs28409621	A	G	0.0467	-0.4719	0.1019	3.65E-06	21.4463
M33173.x	rs3850788	A	G	0.0225	-0.3028	0.0647	2.84E-06	21.903
M33173.x	rs4279445	A	T	0.0132	-0.9923	0.21	2.3E-06	22.3279
M33173.x	rs4299028	A	G	0.0171	-0.9695	0.2028	1.75E-06	22.8539
M33173.x	rs4516988	A	G	0.7676	0.2638	0.0584	6.37E-06	20.4044
M33173.x	rs4670236	C	G	0.9781	0.8758	0.1698	2.51E-07	26.6032
M33173.x	rs4784988	T	C	0.0216	-0.8149	0.1753	3.35E-06	21.6095
M33173.x	rs4805580	T	C	0.9867	0.964	0.2118	5.35E-06	20.7158
M33173.x	rs4951117	A	T	0.0855	-0.4779	0.0752	2.05E-10	40.3867
M33173.x	rs500237	T	C	0.0117	-1.2925	0.2889	7.67E-06	20.0155
M33173.x	rs575723	A	G	0.0135	-0.9929	0.2101	2.29E-06	22.3336
M33173.x	rs6014654	A	G	0.9697	0.6332	0.1395	5.6E-06	20.6031
M33173.x	rs6085450	T	C	0.0338	-1.0001	0.1964	3.56E-07	25.9301
M33173.x	rs6130478	A	G	0.9803	0.8585	0.1698	4.28E-07	25.5626
M33173.x	rs6664483	T	G	0.9803	1.1464	0.1447	2.32E-15	62.7676
M33173.x	rs690764	A	G	0.0574	-0.6782	0.1147	3.36E-09	34.9614
M33173.x	rs7071224	C	G	0.9005	0.3778	0.0779	1.22E-06	23.5206
M33173.x	rs7082110	A	G	0.8649	0.1386	0.0309	7.1E-06	20.1191
M33173.x	rs7120657	T	G	0.0329	-0.6419	0.1355	2.17E-06	22.4417
M33173.x	rs7193141	C	G	0.0658	-0.4283	0.0649	4.12E-11	43.5519
M33173.x	rs7863822	T	C	0.0132	-1.0516	0.206	3.32E-07	26.0595
M33173.x	rs787878	T	C	0.6233	0.2768	0.0609	5.51E-06	20.6584
M33173.x	rs899311	A	T	0.8971	0.4035	0.084	1.55E-06	23.0743
M33173.x	rs9921618	T	C	0.8611	0.3006	0.068	9.92E-06	19.5416

M33178.x	rs10027220	T	C	0.0209	-0.338	0.0744	5.58E-06	20.6389
M33178.x	rs10041407	C	G	0.021	-0.3374	0.0743	5.57E-06	20.6211
M33178.x	rs10044695	A	G	0.9784	0.3085	0.0599	2.58E-07	26.5251
M33178.x	rs10046447	T	C	0.0771	-0.1182	0.0257	4.2E-06	21.1528
M33178.x	rs10060532	C	G	0.9646	0.153	0.0335	4.88E-06	20.859
M33178.x	rs10063245	C	G	0.9788	0.3379	0.0742	5.34E-06	20.7381
M33178.x	rs10085014	T	C	0.979	0.3374	0.0743	5.58E-06	20.6211
M33178.x	rs10100951	T	G	0.02	-0.3367	0.0741	5.58E-06	20.6467
M33178.x	rs1010102	A	G	0.98	0.3367	0.0741	5.58E-06	20.6467
M33178.x	rs10120414	T	C	0.0259	-0.3416	0.073	2.87E-06	21.8973
M33178.x	rs10131251	A	G	0.02	-0.3367	0.0741	5.58E-06	20.6467
M33178.x	rs10132956	A	G	0.02	-0.3367	0.0741	5.58E-06	20.6467
M33178.x	rs10157030	T	C	0.9774	0.3336	0.0754	9.65E-06	19.5753
M33178.x	rs10220710	T	G	0.9789	0.338	0.0744	5.47E-06	20.6389
M33178.x	rs10229296	A	G	0.98	0.3367	0.0741	5.58E-06	20.6467
M33178.x	rs10252504	T	C	0.0208	-0.339	0.0738	4.37E-06	21.1002
M33178.x	rs10274397	T	G	0.9757	0.348	0.0745	2.99E-06	21.8196
M33178.x	rs10274897	T	G	0.98	0.3367	0.0741	5.58E-06	20.6467
M33178.x	rs10276616	T	G	0.021	-0.3374	0.0743	5.58E-06	20.6211
M33178.x	rs1038697	T	C	0.0435	-0.1708	0.0367	3.24E-06	21.6593
M33178.x	rs10415374	A	G	0.027	-0.3327	0.0723	4.23E-06	21.1753
M33178.x	rs10447967	T	G	0.0207	-0.3411	0.0751	5.58E-06	20.6293
M33178.x	rs10471169	T	C	0.0667	-0.1783	0.036	7.43E-07	24.53
M33178.x	rs10486289	A	G	0.979	0.3373	0.0743	5.62E-06	20.6089
M33178.x	rs10488089	C	G	0.98	0.3367	0.0741	5.58E-06	20.6467
M33178.x	rs10494913	A	G	0.02	-0.3367	0.0741	5.58E-06	20.6467
M33178.x	rs10495116	A	C	0.9818	0.3075	0.063	1.06E-06	23.8237
M33178.x	rs1055375	A	G	0.98	0.3367	0.0741	5.58E-06	20.6467
M33178.x	rs10766002	A	G	0.42	0.1241	0.0263	2.28E-06	22.2655
M33178.x	rs10776859	T	C	0.98	0.3367	0.0741	5.58E-06	20.6467
M33178.x	rs10783298	A	G	0.9565	0.1706	0.0368	3.47E-06	21.4913
M33178.x	rs10805325	C	G	0.3578	-0.0815	0.0184	9.81E-06	19.6191
M33178.x	rs10830676	T	G	0.02	-0.3367	0.0741	5.58E-06	20.6467
M33178.x	rs10838566	T	C	0.98	0.3367	0.0741	5.58E-06	20.6467
M33178.x	rs10841927	C	G	0.6919	0.1066	0.0226	2.38E-06	22.2483
M33178.x	rs10842538	T	C	0.04	-0.1888	0.0415	5.27E-06	20.697
M33178.x	rs10849031	C	G	0.9791	0.3384	0.0745	5.58E-06	20.6323
M33178.x	rs10864476	A	G	0.98	0.3367	0.0741	5.58E-06	20.6467
M33178.x	rs10917221	T	C	0.98	0.3367	0.0741	5.58E-06	20.6467
M33178.x	rs10922741	T	C	0.9783	0.3413	0.0735	3.39E-06	21.5624
M33178.x	rs10934820	T	C	0.02	-0.3367	0.0741	5.58E-06	20.6467
M33178.x	rs10961091	T	C	0.96	0.1683	0.0371	5.58E-06	20.5788
M33178.x	rs10962496	A	G	0.02	-0.3367	0.0741	5.58E-06	20.6467
M33178.x	rs10973779	A	G	0.02	-0.3367	0.0741	5.58E-06	20.6467
M33178.x	rs10994406	T	C	0.979	0.3374	0.0743	5.57E-06	20.6211
M33178.x	rs11004576	T	C	0.02	-0.3367	0.0741	5.58E-06	20.6467
M33178.x	rs11004724	T	G	0.9788	0.3463	0.0757	4.81E-06	20.9273
M33178.x	rs11013791	A	G	0.0327	-0.3467	0.0746	3.4E-06	21.5988
M33178.x	rs11019667	T	C	0.02	-0.3367	0.0741	5.58E-06	20.6467
M33178.x	rs11032476	A	G	0.02	-0.3367	0.0741	5.58E-06	20.6467
M33178.x	rs11038541	T	C	0.9659	-0.2283	0.0505	6.23E-06	20.4376
M33178.x	rs11041611	T	C	0.979	0.3374	0.0743	5.58E-06	20.6211
M33178.x	rs11057970	T	C	0.04	-0.1683	0.0371	5.58E-06	20.5788
M33178.x	rs11067315	T	C	0.02	-0.3367	0.0741	5.58E-06	20.6467
M33178.x	rs11112162	T	C	0.0208	-0.3386	0.0739	4.6E-06	20.9935

M33178.x	rs11121715	T	C	0.9816	0.2845	0.0618	4.12E-06	21.1928
M33178.x	rs11183923	A	C	0.02	-0.3367	0.0741	5.58E-06	20.6467
M33178.x	rs11198080	A	C	0.02	-0.3367	0.0741	5.58E-06	20.6467
M33178.x	rs11202938	A	G	0.98	0.3367	0.0741	5.58E-06	20.6467
M33178.x	rs11228559	T	C	0.02	-0.3367	0.0741	5.58E-06	20.6467
M33178.x	rs11256010	T	C	0.98	0.3367	0.0741	5.58E-06	20.6467
M33178.x	rs11257897	T	C	0.9783	0.3384	0.0743	5.32E-06	20.7435
M33178.x	rs11608731	T	G	0.8494	0.1062	0.0223	1.82E-06	22.6798
M33178.x	rs11612522	T	C	0.021	-0.3374	0.0743	5.58E-06	20.6211
M33178.x	rs11613732	T	C	0.0209	-0.3377	0.0744	5.58E-06	20.6023
M33178.x	rs11620793	A	T	0.705	-0.0857	0.019	6.64E-06	20.3448
M33178.x	rs11664692	A	G	0.04	-0.1683	0.0371	5.58E-06	20.5788
M33178.x	rs11672123	A	G	0.0233	-0.2949	0.0647	5.18E-06	20.775
M33178.x	rs1169758	A	G	0.4496	0.0683	0.0153	8.47E-06	19.9278
M33178.x	rs11698917	A	T	0.021	-0.3374	0.0743	5.58E-06	20.6211
M33178.x	rs1174851	A	G	0.02	-0.3367	0.0741	5.58E-06	20.6467
M33178.x	rs11753364	A	G	0.9783	0.3327	0.0752	9.62E-06	19.5736
M33178.x	rs11753418	A	G	0.0208	-0.3374	0.0741	5.32E-06	20.7326
M33178.x	rs11770198	A	G	0.9703	0.333	0.0753	9.85E-06	19.5568
M33178.x	rs11826948	A	G	0.9739	0.3427	0.0737	3.36E-06	21.6219
M33178.x	rs11856508	T	C	0.98	0.3367	0.0741	5.58E-06	20.6467
M33178.x	rs11876642	T	C	0.0271	-0.348	0.0718	1.23E-06	23.4914
M33178.x	rs11876642	T	C	0.0271	-0.348	0.0718	1.23E-06	23.4914
M33178.x	rs11878031	T	G	0.021	-0.3374	0.0743	5.58E-06	20.6211
M33178.x	rs11893311	A	G	0.0187	0.2928	0.0654	7.7E-06	20.0441
M33178.x	rs11916772	T	C	0.979	0.3374	0.0743	5.58E-06	20.6211
M33178.x	rs11936853	A	T	0.979	0.3374	0.0743	5.58E-06	20.6211
M33178.x	rs11943366	A	G	0.0208	-0.3377	0.0741	5.1E-06	20.7695
M33178.x	rs11948317	A	C	0.02	-0.3367	0.0741	5.58E-06	20.6467
M33178.x	rs11957930	A	G	0.0459	-0.2362	0.0515	4.58E-06	21.0351
M33178.x	rs11964805	A	G	0.021	-0.3374	0.0743	5.58E-06	20.6211
M33178.x	rs12004211	A	T	0.02	-0.3367	0.0741	5.58E-06	20.6467
M33178.x	rs12036516	A	G	0.9782	0.3396	0.0741	4.56E-06	21.0039
M33178.x	rs12122792	A	G	0.038	-0.1811	0.0332	4.87E-08	29.7551
M33178.x	rs12165607	C	G	0.0266	-0.329	0.0725	5.67E-06	20.5928
M33178.x	rs12189492	A	G	0.9787	0.3379	0.0743	5.38E-06	20.6823
M33178.x	rs1219976	A	G	0.02	-0.3367	0.0741	5.58E-06	20.6467
M33178.x	rs12226635	T	C	0.02	-0.3367	0.0741	5.58E-06	20.6467
M33178.x	rs12246360	A	C	0.9792	0.2665	0.0537	6.93E-07	24.6289
M33178.x	rs12285787	T	G	0.02	-0.3367	0.0741	5.58E-06	20.6467
M33178.x	rs12327061	T	C	0.98	0.3367	0.0741	5.58E-06	20.6467
M33178.x	rs12377744	T	C	0.0222	-0.3323	0.0728	4.95E-06	20.8352
M33178.x	rs12405026	A	G	0.04	-0.1683	0.0371	5.58E-06	20.5788
M33178.x	rs12416036	T	C	0.9703	0.3391	0.073	3.36E-06	21.5779
M33178.x	rs12422516	C	G	0.979	0.3374	0.0743	5.58E-06	20.6211
M33178.x	rs12426085	A	G	0.02	-0.3367	0.0741	5.58E-06	20.6467
M33178.x	rs12512668	T	G	0.9791	0.3395	0.0747	5.54E-06	20.6556
M33178.x	rs12583363	T	C	0.9762	0.3419	0.0737	3.46E-06	21.521
M33178.x	rs12590150	T	C	0.98	0.3367	0.0741	5.58E-06	20.6467
M33178.x	rs12599655	C	G	0.9583	0.1693	0.0369	4.6E-06	21.0504
M33178.x	rs12607891	A	G	0.979	0.3374	0.0743	5.58E-06	20.6211
M33178.x	rs12654585	T	C	0.9789	0.3377	0.0742	5.41E-06	20.7135
M33178.x	rs12665113	A	T	0.98	0.3367	0.0741	5.58E-06	20.6467
M33178.x	rs12679602	A	G	0.0561	-0.1582	0.0337	2.72E-06	22.037
M33178.x	rs12758010	A	G	0.0208	-0.3386	0.0739	4.6E-06	20.9935

M33178.x	rs12777389	T	C	0.0211	-0.3403	0.0748	5.32E-06	20.6976
M33178.x	rs12857335	T	G	0.9712	0.3328	0.075	0.000009	19.6899
M33178.x	rs12867467	T	C	0.0311	-0.3372	0.0749	6.77E-06	20.268
M33178.x	rs12902028	A	G	0.02	-0.3367	0.0741	5.58E-06	20.6467
M33178.x	rs12950011	T	C	0.5363	0.0861	0.0188	4.66E-06	20.9745
M33178.x	rs12959082	T	C	0.0184	-0.2744	0.0615	8.09E-06	19.9076
M33178.x	rs12971134	A	T	0.9789	0.3377	0.0742	5.4E-06	20.7135
M33178.x	rs13061003	T	C	0.0223	-0.3406	0.0739	4.07E-06	21.2422
M33178.x	rs13326335	T	C	0.02	-0.3367	0.0741	5.58E-06	20.6467
M33178.x	rs13329052	T	C	0.9711	0.3543	0.0758	2.91E-06	21.8476
M33178.x	rs13337056	A	G	0.0208	-0.339	0.0738	4.37E-06	21.1002
M33178.x	rs13385838	A	G	0.1003	-0.1364	0.029	2.61E-06	22.1224
M33178.x	rs13410411	A	G	0.979	0.3374	0.0743	5.58E-06	20.6211
M33178.x	rs1352545	A	T	0.98	0.3367	0.0741	5.58E-06	20.6467
M33178.x	rs1355540	A	G	0.979	0.3374	0.0743	5.58E-06	20.6211
M33178.x	rs1358551	T	C	0.979	0.3374	0.0743	5.58E-06	20.6211
M33178.x	rs1360391	A	C	0.0217	-0.3404	0.0736	3.79E-06	21.3906
M33178.x	rs1368009	A	G	0.979	0.3374	0.0743	5.58E-06	20.6211
M33178.x	rs1371599	C	G	0.021	-0.3374	0.0743	5.58E-06	20.6211
M33178.x	rs1444113	A	C	0.0191	-0.297	0.064	3.45E-06	21.5354
M33178.x	rs1468284	T	C	0.8846	0.161	0.0346	3.22E-06	21.6521
M33178.x	rs1472170	T	G	0.0212	-0.3405	0.0746	5.02E-06	20.8332
M33178.x	rs1472292	A	G	0.979	0.3374	0.0743	5.58E-06	20.6211
M33178.x	rs1473864	A	C	0.021	-0.3374	0.0743	5.58E-06	20.6211
M33178.x	rs1510969	T	C	0.979	0.3374	0.0743	5.58E-06	20.6211
M33178.x	rs1513215	T	G	0.98	0.3367	0.0741	5.58E-06	20.6467
M33178.x	rs1516626	C	G	0.0239	-0.3444	0.0736	2.87E-06	21.8963
M33178.x	rs1535938	T	C	0.0208	-0.3386	0.0739	4.6E-06	20.9935
M33178.x	rs154554	C	G	0.9783	0.3413	0.0735	3.39E-06	21.5624
M33178.x	rs1581614	C	G	0.98	0.3367	0.0741	5.58E-06	20.6467
M33178.x	rs1585145	A	G	0.96	0.1683	0.0371	5.58E-06	20.5788
M33178.x	rs1609916	A	G	0.02	-0.3367	0.0741	5.58E-06	20.6467
M33178.x	rs1636949	A	C	0.021	-0.3374	0.0743	5.58E-06	20.6211
M33178.x	rs1645773	A	G	0.02	-0.3367	0.0741	5.58E-06	20.6467
M33178.x	rs1676300	T	C	0.0325	-0.3244	0.0699	3.48E-06	21.5381
M33178.x	rs16824664	A	C	0.0208	-0.3386	0.0739	4.6E-06	20.9935
M33178.x	rs16827187	T	C	0.02	-0.3367	0.0741	5.58E-06	20.6467
M33178.x	rs16834028	A	T	0.0905	-0.1197	0.0269	8.4E-06	19.8008
M33178.x	rs16837093	A	G	0.9693	0.3399	0.0729	3.1E-06	21.7394
M33178.x	rs16849237	A	G	0.02	-0.3367	0.0741	5.58E-06	20.6467
M33178.x	rs16854290	C	G	0.98	0.3367	0.0741	5.58E-06	20.6467
M33178.x	rs16856275	T	C	0.9792	0.3383	0.0739	4.75E-06	20.9563
M33178.x	rs16856874	A	G	0.075	-0.1442	0.0324	8.27E-06	19.808
M33178.x	rs16859878	T	C	0.04	-0.1683	0.0371	5.58E-06	20.5788
M33178.x	rs16863208	A	G	0.4783	0.3379	0.0741	5.16E-06	20.7941
M33178.x	rs16864792	T	C	0.0435	-0.1704	0.0368	3.6E-06	21.4409
M33178.x	rs16869484	A	G	0.979	0.3374	0.0743	5.58E-06	20.6211
M33178.x	rs16877016	A	G	0.0243	-0.3396	0.0729	3.18E-06	21.701
M33178.x	rs16890511	T	C	0.98	0.3367	0.0741	5.58E-06	20.6467
M33178.x	rs16894341	T	C	0.9687	0.2045	0.0457	7.65E-06	20.0242
M33178.x	rs16894362	T	C	0.02	-0.3367	0.0741	5.58E-06	20.6467
M33178.x	rs16901935	A	G	0.98	0.3367	0.0741	5.58E-06	20.6467
M33178.x	rs16909586	A	C	0.02	-0.3367	0.0741	5.58E-06	20.6467
M33178.x	rs16913175	C	G	0.979	0.3374	0.0743	5.58E-06	20.6211
M33178.x	rs16926097	T	G	0.9792	0.3386	0.0739	4.6E-06	20.9935

M33178.x	rs16927166	T	C	0.98	0.3367	0.0741	5.58E-06	20.6467
M33178.x	rs16936983	A	G	0.9823	0.2563	0.0541	2.13E-06	22.4441
M33178.x	rs16943260	A	G	0.979	0.3374	0.0743	5.58E-06	20.6211
M33178.x	rs16944662	A	G	0.4773	0.3344	0.075	8.23E-06	19.8797
M33178.x	rs16954769	A	G	0.0207	-0.3423	0.0753	5.53E-06	20.6644
M33178.x	rs16960341	T	C	0.0208	-0.3372	0.0742	5.43E-06	20.6522
M33178.x	rs16978034	C	G	0.02	-0.3367	0.0741	5.58E-06	20.6467
M33178.x	rs16992346	A	G	0.02	-0.3367	0.0741	5.58E-06	20.6467
M33178.x	rs16997544	T	C	0.02	-0.3367	0.0741	5.58E-06	20.6467
M33178.x	rs16998847	A	G	0.02	-0.3367	0.0741	5.58E-06	20.6467
M33178.x	rs17020850	C	G	0.9774	0.3377	0.0735	4.34E-06	21.11
M33178.x	rs17027182	C	G	0.02	-0.3367	0.0741	5.58E-06	20.6467
M33178.x	rs17027472	T	C	0.979	0.3374	0.0743	5.58E-06	20.6211
M33178.x	rs17028753	A	T	0.9792	0.3392	0.0737	4.23E-06	21.1825
M33178.x	rs17029741	T	C	0.04	-0.1683	0.0371	5.58E-06	20.5788
M33178.x	rs17030263	T	G	0.02	-0.3367	0.0741	5.58E-06	20.6467
M33178.x	rs17031781	T	C	0.021	-0.3374	0.0743	5.58E-06	20.6211
M33178.x	rs17050376	C	G	0.02	-0.3367	0.0741	5.58E-06	20.6467
M33178.x	rs17053995	A	G	0.979	0.3374	0.0743	5.58E-06	20.6211
M33178.x	rs17061604	A	G	0.0244	-0.3399	0.0738	4.12E-06	21.2124
M33178.x	rs17068330	A	G	0.9754	0.3398	0.0737	4.05E-06	21.2575
M33178.x	rs17076137	T	C	0.02	-0.3367	0.0741	5.58E-06	20.6467
M33178.x	rs17079498	T	C	0.98	0.3367	0.0741	5.58E-06	20.6467
M33178.x	rs17085716	A	C	0.979	0.3374	0.0743	5.58E-06	20.6211
M33178.x	rs17087115	A	C	0.98	0.3367	0.0741	5.58E-06	20.6467
M33178.x	rs17088496	C	G	0.9762	0.3386	0.0743	5.21E-06	20.7681
M33178.x	rs17091532	A	G	0.0227	-0.3441	0.073	2.46E-06	22.219
M33178.x	rs17093221	T	C	0.0368	-0.1649	0.0348	2.16E-06	22.4534
M33178.x	rs17098188	T	C	0.02	-0.3367	0.0741	5.58E-06	20.6467
M33178.x	rs17098805	T	C	0.9792	0.3374	0.0741	5.32E-06	20.7326
M33178.x	rs17100461	T	G	0.98	0.3367	0.0741	5.58E-06	20.6467
M33178.x	rs17106046	T	C	0.0221	-0.255	0.0513	6.61E-07	24.7085
M33178.x	rs17108927	T	C	0.98	0.3367	0.0741	5.58E-06	20.6467
M33178.x	rs17109605	T	C	0.5217	-0.3399	0.0738	4.06E-06	21.2124
M33178.x	rs17119874	T	C	0.02	-0.3367	0.0741	5.58E-06	20.6467
M33178.x	rs17126321	T	G	0.98	0.3367	0.0741	5.58E-06	20.6467
M33178.x	rs17127107	C	G	0.9792	0.3347	0.0746	7.34E-06	20.1295
M33178.x	rs17140558	A	G	0.9707	0.3375	0.0688	9.24E-07	24.0642
M33178.x	rs17149000	T	G	0.98	0.3367	0.0741	5.58E-06	20.6467
M33178.x	rs17154872	A	G	0.977	0.3418	0.0738	3.64E-06	21.4502
M33178.x	rs17157513	A	G	0.0228	-0.3405	0.0736	3.75E-06	21.4032
M33178.x	rs17164003	T	G	0.98	0.3367	0.0741	5.58E-06	20.6467
M33178.x	rs17167552	T	C	0.9545	0.1704	0.0369	3.81E-06	21.3249
M33178.x	rs17168817	T	C	0.02	-0.3367	0.0741	5.58E-06	20.6467
M33178.x	rs17181818	T	C	0.0231	-0.3433	0.074	3.46E-06	21.5221
M33178.x	rs17208695	T	G	0.98	0.3367	0.0741	5.58E-06	20.6467
M33178.x	rs17209113	C	G	0.02	-0.3367	0.0741	5.58E-06	20.6467
M33178.x	rs17263168	T	C	0.9785	0.3422	0.0746	4.49E-06	21.0418
M33178.x	rs17291576	A	G	0.4773	0.3411	0.0737	3.65E-06	21.4204
M33178.x	rs17314070	T	C	0.95	0.3034	0.0655	3.6E-06	21.456
M33178.x	rs17397601	A	G	0.0212	-0.346	0.0758	4.96E-06	20.836
M33178.x	rs17435401	T	G	0.98	0.3367	0.0741	5.58E-06	20.6467
M33178.x	rs17470657	A	T	0.944	0.3263	0.0679	1.54E-06	23.0938
M33178.x	rs17481016	T	C	0.0237	-0.2574	0.0572	6.78E-06	20.25
M33178.x	rs17576178	T	C	0.979	0.3375	0.0743	5.54E-06	20.6334

M33178.x	rs17595169	A	T	0.98	0.3367	0.0741	5.58E-06	20.6467
M33178.x	rs17628684	A	G	0.9726	0.2143	0.0484	9.75E-06	19.6044
M33178.x	rs17650707	T	G	0.9736	0.3371	0.0752	7.35E-06	20.0947
M33178.x	rs17659760	A	G	0.0217	-0.3372	0.0743	5.61E-06	20.5967
M33178.x	rs17680579	A	G	0.8817	0.1031	0.0233	9.41E-06	19.5797
M33178.x	rs17693330	A	C	0.0314	-0.3291	0.0723	5.23E-06	20.7195
M33178.x	rs17721790	A	G	0.02	-0.3367	0.0741	5.58E-06	20.6467
M33178.x	rs17810433	A	G	0.0258	-0.3362	0.0758	9.24E-06	19.6724
M33178.x	rs17825562	A	G	0.04	-0.1683	0.0371	5.58E-06	20.5788
M33178.x	rs17829368	T	C	0.98	0.3367	0.0741	5.58E-06	20.6467
M33178.x	rs17863803	A	G	0.98	0.3367	0.0741	5.58E-06	20.6467
M33178.x	rs1791280	A	T	0.7577	0.1204	0.0269	7.88E-06	20.0331
M33178.x	rs1799453	C	G	0.9804	0.2531	0.056	6.14E-06	20.4272
M33178.x	rs1800777	A	G	0.02	-0.3367	0.0741	5.58E-06	20.6467
M33178.x	rs1865660	A	G	0.98	0.3367	0.0741	5.58E-06	20.6467
M33178.x	rs1865893	A	G	0.021	-0.3374	0.0743	5.58E-06	20.6211
M33178.x	rs1891251	A	G	0.9757	0.3376	0.073	3.76E-06	21.3875
M33178.x	rs1927455	A	G	0.1058	-0.1462	0.0328	8.11E-06	19.8677
M33178.x	rs1929543	T	C	0.98	0.3367	0.0741	5.58E-06	20.6467
M33178.x	rs2029619	T	C	0.0208	-0.3386	0.0739	4.6E-06	20.9935
M33178.x	rs2047682	T	C	0.9778	0.34	0.0739	4.14E-06	21.1675
M33178.x	rs2053450	T	G	0.9762	0.3394	0.0746	5.33E-06	20.6988
M33178.x	rs2057276	A	T	0.98	0.3367	0.0741	5.58E-06	20.6467
M33178.x	rs2064496	A	T	0.0211	-0.3388	0.0745	5.34E-06	20.6811
M33178.x	rs2113097	T	C	0.02	-0.3367	0.0741	5.58E-06	20.6467
M33178.x	rs2121016	A	T	0.9772	0.3408	0.0737	3.73E-06	21.3828
M33178.x	rs2145526	A	G	0.979	0.3374	0.0743	5.58E-06	20.6211
M33178.x	rs2173225	C	G	0.9786	0.3373	0.074	5.11E-06	20.7763
M33178.x	rs2177959	T	C	0.98	0.3367	0.0741	5.58E-06	20.6467
M33178.x	rs2188978	A	T	0.0243	-0.3291	0.0722	5.12E-06	20.7769
M33178.x	rs2218123	A	T	0.9792	0.3397	0.0748	5.58E-06	20.6247
M33178.x	rs2227580	T	G	0.0209	-0.3384	0.0745	5.58E-06	20.6323
M33178.x	rs2230944	A	G	0.023	-0.3402	0.0753	6.25E-06	20.4117
M33178.x	rs2235380	T	C	0.9753	0.3385	0.0727	3.24E-06	21.6795
M33178.x	rs2236217	A	C	0.0619	0.1637	0.0368	8.7E-06	19.788
M33178.x	rs2241445	T	C	0.02	-0.3367	0.0741	5.58E-06	20.6467
M33178.x	rs2249531	A	G	0.5482	-0.0816	0.0177	4.19E-06	21.2537
M33178.x	rs2250600	A	G	0.98	0.3367	0.0741	5.58E-06	20.6467
M33178.x	rs2273617	A	G	0.96	0.1683	0.0371	5.58E-06	20.5788
M33178.x	rs2280064	A	G	0.979	0.3374	0.0743	5.58E-06	20.6211
M33178.x	rs2284767	A	G	0.98	0.3367	0.0741	5.58E-06	20.6467
M33178.x	rs2299161	A	G	0.02	-0.3367	0.0741	5.58E-06	20.6467
M33178.x	rs2305000	T	C	0.98	0.3367	0.0741	5.58E-06	20.6467
M33178.x	rs231966	T	C	0.02	-0.3367	0.0741	5.58E-06	20.6467
M33178.x	rs2348445	T	C	0.8191	-0.1059	0.0238	8.63E-06	19.7988
M33178.x	rs2361394	A	G	0.9584	0.1413	0.0306	3.75E-06	21.3227
M33178.x	rs2410660	T	C	0.0268	-0.3489	0.0743	2.68E-06	22.0508
M33178.x	rs2429980	A	G	0.98	0.3367	0.0741	5.58E-06	20.6467
M33178.x	rs2455406	T	G	0.78	0.1146	0.0256	7.56E-06	20.0396
M33178.x	rs247858	A	C	0.0218	-0.3421	0.0745	4.43E-06	21.086
M33178.x	rs2513641	A	G	0.979	0.3374	0.0743	5.56E-06	20.6211
M33178.x	rs2647998	T	C	0.02	-0.3367	0.0741	5.58E-06	20.6467
M33178.x	rs275362	A	G	0.4565	-0.1249	0.0245	3.39E-07	25.9892
M33178.x	rs2760672	A	G	0.979	0.3374	0.0743	5.58E-06	20.6211
M33178.x	rs2802568	A	T	0.02	-0.3367	0.0741	5.58E-06	20.6467

M33178.x	rs2804609	T	C	0.0214	-0.2427	0.0541	7.13E-06	20.1254
M33178.x	rs28505465	T	C	0.98	0.3367	0.0741	5.58E-06	20.6467
M33178.x	rs288438	A	G	0.0435	-0.1709	0.0367	3.21E-06	21.6846
M33178.x	rs2891717	A	G	0.981	0.3226	0.0623	2.26E-07	26.8134
M33178.x	rs2964846	A	T	0.9793	0.3425	0.0754	5.58E-06	20.6338
M33178.x	rs2968779	T	C	0.0267	-0.2532	0.0565	7.34E-06	20.0831
M33178.x	rs3008254	T	C	0.0216	-0.3441	0.075	4.42E-06	21.0497
M33178.x	rs3026502	A	G	0.02	-0.3367	0.0741	5.58E-06	20.6467
M33178.x	rs3026770	A	G	0.979	0.3374	0.0743	5.58E-06	20.6211
M33178.x	rs305550	T	C	0.0227	-0.34	0.0739	4.19E-06	21.1675
M33178.x	rs310907	A	T	0.0211	-0.3381	0.0746	5.85E-06	20.5406
M33178.x	rs3169950	A	G	0.409	0.0901	0.0198	5.32E-06	20.7071
M33178.x	rs3219080	T	C	0.0298	-0.315	0.0707	8.29E-06	19.851
M33178.x	rs323090	T	C	0.9792	0.3398	0.0748	5.57E-06	20.6369
M33178.x	rs33469	A	G	0.9553	0.1639	0.0355	4.01E-06	21.3158
M33178.x	rs35875280	T	G	0.02	-0.3367	0.0741	5.58E-06	20.6467
M33178.x	rs368353	A	G	0.98	0.3367	0.0741	5.58E-06	20.6467
M33178.x	rs3763903	A	G	0.02	-0.3367	0.0741	5.58E-06	20.6467
M33178.x	rs3780205	A	G	0.0519	-0.1254	0.0278	6.6E-06	20.3472
M33178.x	rs3781723	T	C	0.9792	0.3386	0.0739	4.6E-06	20.9935
M33178.x	rs3782872	A	G	0.02	-0.3367	0.0741	5.58E-06	20.6467
M33178.x	rs3805014	A	G	0.02	-0.3367	0.0741	5.58E-06	20.6467
M33178.x	rs3811321	A	C	0.9373	0.1266	0.0281	6.78E-06	20.2981
M33178.x	rs3814168	A	G	0.021	-0.3379	0.0743	5.49E-06	20.6823
M33178.x	rs3816015	A	T	0.3923	-0.1041	0.0164	2.2E-10	40.2915
M33178.x	rs3818305	A	C	0.02	-0.3367	0.0741	5.58E-06	20.6467
M33178.x	rs3821143	T	C	0.9793	0.3469	0.0761	5.16E-06	20.7797
M33178.x	rs3889670	T	C	0.0211	-0.3373	0.0743	5.71E-06	20.6089
M33178.x	rs3900763	T	G	0.021	-0.3375	0.0743	5.51E-06	20.6334
M33178.x	rs3928602	A	G	0.021	-0.3381	0.0744	5.56E-06	20.6512
M33178.x	rs4060121	C	G	0.98	0.3367	0.0741	5.58E-06	20.6467
M33178.x	rs4077730	T	C	0.98	0.3367	0.0741	5.58E-06	20.6467
M33178.x	rs4130875	T	C	0.02	-0.3367	0.0741	5.58E-06	20.6467
M33178.x	rs421380	T	C	0.9728	0.353	0.0758	3.24E-06	21.6876
M33178.x	rs4242763	T	C	0.979	0.341	0.0749	5.29E-06	20.7274
M33178.x	rs42441	T	G	0.9799	0.2726	0.0571	1.8E-06	22.7918
M33178.x	rs4244278	A	T	0.9789	0.3375	0.0743	5.57E-06	20.6334
M33178.x	rs4246023	A	G	0.0227	-0.3336	0.0753	9.46E-06	19.6274
M33178.x	rs4294438	A	G	0.9765	0.2511	0.0525	1.72E-06	22.8757
M33178.x	rs4313653	T	C	0.0217	-0.3397	0.0738	4.15E-06	21.1874
M33178.x	rs4333550	A	G	0.9783	0.3411	0.0735	3.47E-06	21.5372
M33178.x	rs436268	T	C	0.0208	-0.339	0.0738	4.37E-06	21.1002
M33178.x	rs4379054	A	C	0.027	-0.2713	0.0603	6.89E-06	20.2425
M33178.x	rs4427044	A	G	0.021	-0.3374	0.0743	5.58E-06	20.6211
M33178.x	rs4441723	T	C	0.9583	0.1695	0.0369	4.32E-06	21.1002
M33178.x	rs4453029	T	C	0.0219	-0.3389	0.0744	5.22E-06	20.749
M33178.x	rs4505105	A	C	0.02	-0.3367	0.0741	5.58E-06	20.6467
M33178.x	rs4507748	A	G	0.98	0.3367	0.0741	5.58E-06	20.6467
M33178.x	rs4545881	T	C	0.9832	0.2706	0.0609	8.95E-06	19.7434
M33178.x	rs4589673	A	G	0.0208	-0.3383	0.0739	4.75E-06	20.9563
M33178.x	rs4599300	T	C	0.02	-0.3367	0.0741	5.58E-06	20.6467
M33178.x	rs4602498	A	G	0.02	-0.3367	0.0741	5.58E-06	20.6467
M33178.x	rs4602520	T	C	0.9527	0.3279	0.0726	6.21E-06	20.399
M33178.x	rs465409	A	C	0.0217	-0.337	0.0743	5.8E-06	20.5723
M33178.x	rs4670236	C	G	0.9779	0.3378	0.0743	5.47E-06	20.6701

M33178.x	rs4674097	A	G	0.9789	0.3395	0.0745	5.16E-06	20.7667
M33178.x	rs4678695	A	C	0.0211	-0.3379	0.0744	5.51E-06	20.6267
M33178.x	rs4683468	T	C	0.02	-0.3367	0.0741	5.58E-06	20.6467
M33178.x	rs4748887	A	G	0.9793	0.3422	0.0753	5.58E-06	20.6524
M33178.x	rs4749376	A	G	0.02	-0.3367	0.0741	5.58E-06	20.6467
M33178.x	rs4766348	A	G	0.98	0.3367	0.0741	5.58E-06	20.6467
M33178.x	rs4786186	A	C	0.9792	0.2679	0.0589	5.34E-06	20.6878
M33178.x	rs4787041	T	C	0.9626	0.1672	0.0352	2.05E-06	22.5625
M33178.x	rs4805151	A	G	0.0347	-0.2498	0.0476	1.5E-07	27.5404
M33178.x	rs4812754	A	G	0.9635	0.3151	0.0692	5.36E-06	20.7341
M33178.x	rs4843884	T	C	0.9792	0.3356	0.0745	6.62E-06	20.2923
M33178.x	rs4847371	T	C	0.02	-0.3367	0.0741	5.58E-06	20.6467
M33178.x	rs4918228	A	T	0.98	0.3367	0.0741	5.58E-06	20.6467
M33178.x	rs4928021	A	T	0.98	0.3367	0.0741	5.58E-06	20.6467
M33178.x	rs4942047	C	G	0.98	0.3367	0.0741	5.58E-06	20.6467
M33178.x	rs4961483	A	G	0.0216	-0.338	0.0743	5.43E-06	20.6945
M33178.x	rs4964992	T	C	0.5326	0.0752	0.0165	5.01E-06	20.7715
M33178.x	rs4965131	T	G	0.125	-0.1551	0.0348	8.35E-06	19.8639
M33178.x	rs4982501	T	G	0.0217	-0.3379	0.0741	5.16E-06	20.7941
M33178.x	rs521361	T	C	0.0209	-0.3377	0.0744	5.58E-06	20.6023
M33178.x	rs545142	A	T	0.02	-0.3367	0.0741	5.58E-06	20.6467
M33178.x	rs547344	A	G	0.0607	0.1872	0.0399	2.75E-06	22.0123
M33178.x	rs5747033	A	G	0.9792	0.3391	0.0738	4.32E-06	21.1127
M33178.x	rs575136	T	C	0.02	-0.3367	0.0741	5.58E-06	20.6467
M33178.x	rs588113	A	G	0.0476	-0.1722	0.0366	2.53E-06	22.1363
M33178.x	rs6001118	A	T	0.02	-0.3367	0.0741	5.58E-06	20.6467
M33178.x	rs6006868	A	G	0.0213	-0.3377	0.0743	5.48E-06	20.6578
M33178.x	rs6040322	A	C	0.8986	0.1155	0.0259	8.27E-06	19.8868
M33178.x	rs6041405	A	G	0.9716	0.319	0.0714	7.96E-06	19.9611
M33178.x	rs6066168	A	G	0.0218	-0.3223	0.0635	3.82E-07	25.7616
M33178.x	rs6115656	T	C	0.98	0.3367	0.0741	5.58E-06	20.6467
M33178.x	rs6122991	A	G	0.0212	-0.3398	0.0745	5.06E-06	20.8034
M33178.x	rs6136119	A	G	0.02	-0.3367	0.0741	5.58E-06	20.6467
M33178.x	rs6137014	A	C	0.021	-0.3377	0.0744	5.56E-06	20.6023
M33178.x	rs628495	C	G	0.9792	0.338	0.074	4.9E-06	20.8627
M33178.x	rs633810	T	C	0.094	-0.1081	0.0236	4.79E-06	20.9811
M33178.x	rs6436032	T	C	0.05	-0.1697	0.0372	4.94E-06	20.8103
M33178.x	rs6436102	T	C	0.1875	-0.1188	0.0264	6.89E-06	20.25
M33178.x	rs6459477	T	C	0.96	0.1683	0.0371	5.58E-06	20.5788
M33178.x	rs6475096	T	C	0.98	0.3367	0.0741	5.58E-06	20.6467
M33178.x	rs6495823	T	C	0.02	-0.3367	0.0741	5.58E-06	20.6467
M33178.x	rs6499642	T	C	0.0253	-0.3419	0.0736	3.34E-06	21.5796
M33178.x	rs6500125	T	G	0.979	0.3374	0.0743	5.58E-06	20.6211
M33178.x	rs6534458	T	C	0.0185	-0.3044	0.0616	7.65E-07	24.419
M33178.x	rs653513	T	G	0.98	0.3367	0.0741	5.58E-06	20.6467
M33178.x	rs654974	A	G	0.0227	-0.3421	0.0735	3.22E-06	21.6636
M33178.x	rs6561867	A	G	0.02	-0.3367	0.0741	5.58E-06	20.6467
M33178.x	rs6578873	T	G	0.0535	-0.3042	0.0649	2.72E-06	21.9699
M33178.x	rs6664636	A	G	0.959	0.1693	0.0373	5.53E-06	20.6014
M33178.x	rs6677918	T	C	0.0208	-0.339	0.0738	4.37E-06	21.1002
M33178.x	rs6685155	A	G	0.9787	0.3336	0.0734	5.44E-06	20.6567
M33178.x	rs6693659	A	C	0.0208	-0.3392	0.0737	4.23E-06	21.1825
M33178.x	rs6711230	T	C	0.98	0.3367	0.0741	5.58E-06	20.6467
M33178.x	rs6716565	C	G	0.9778	0.3381	0.0744	5.49E-06	20.6512
M33178.x	rs6734884	T	G	0.979	0.3374	0.0743	5.58E-06	20.6211

M33178.x	rs674341	A	G	0.021	-0.3374	0.0743	5.55E-06	20.6211
M33178.x	rs6798383	C	G	0.9748	0.3429	0.0736	3.21E-06	21.706
M33178.x	rs6813770	T	C	0.979	0.3374	0.0743	5.58E-06	20.6211
M33178.x	rs6851302	A	G	0.9783	0.3408	0.0736	3.63E-06	21.4409
M33178.x	rs6873660	A	G	0.9683	0.3413	0.0717	1.93E-06	22.6587
M33178.x	rs6903934	T	C	0.0212	-0.338	0.0744	5.46E-06	20.6389
M33178.x	rs6915443	T	C	0.0707	-0.177	0.0384	3.99E-06	21.2463
M33178.x	rs6952699	A	C	0.02	-0.3367	0.0741	5.58E-06	20.6467
M33178.x	rs696686	T	C	0.98	0.3367	0.0741	5.58E-06	20.6467
M33178.x	rs6987404	T	C	0.0588	-0.163	0.0346	2.53E-06	22.1934
M33178.x	rs7015641	A	G	0.0227	-0.3444	0.073	2.38E-06	22.2577
M33178.x	rs7028666	A	G	0.02	-0.3367	0.0741	5.58E-06	20.6467
M33178.x	rs7030552	A	G	0.02	-0.3367	0.0741	5.58E-06	20.6467
M33178.x	rs7036654	T	C	0.02	-0.3367	0.0741	5.58E-06	20.6467
M33178.x	rs7089115	A	G	0.0178	-0.2733	0.0577	2.19E-06	22.4351
M33178.x	rs7118023	A	G	0.98	0.3367	0.0741	5.58E-06	20.6467
M33178.x	rs7121134	T	G	0.0216	-0.3388	0.0741	4.9E-06	20.905
M33178.x	rs712404	T	C	0.9767	0.3403	0.0734	3.58E-06	21.4947
M33178.x	rs7128387	C	G	0.9791	0.3394	0.0746	5.42E-06	20.6988
M33178.x	rs7137664	A	G	0.9791	0.3377	0.0744	5.58E-06	20.6023
M33178.x	rs7144248	T	C	0.0212	-0.3368	0.0744	6.06E-06	20.4927
M33178.x	rs7187710	T	C	0.98	0.3367	0.0741	5.58E-06	20.6467
M33178.x	rs7207538	T	G	0.98	0.3367	0.0741	5.58E-06	20.6467
M33178.x	rs7276997	A	G	0.0245	-0.3408	0.0735	3.51E-06	21.4993
M33178.x	rs7282706	A	G	0.975	0.3395	0.073	3.32E-06	21.6289
M33178.x	rs7295522	T	C	0.0217	-0.3392	0.0739	4.43E-06	21.068
M33178.x	rs7305247	A	G	0.0234	-0.3357	0.0751	7.81E-06	19.9813
M33178.x	rs7306127	A	C	0.021	-0.3374	0.0743	5.58E-06	20.6211
M33178.x	rs7315726	C	G	0.979	0.3374	0.0743	5.56E-06	20.6211
M33178.x	rs733501	T	C	0.98	0.3367	0.0741	5.58E-06	20.6467
M33178.x	rs734054	T	C	0.0258	-0.3449	0.075	4.22E-06	21.1477
M33178.x	rs7529480	T	C	0.0207	-0.3485	0.0764	5.04E-06	20.8074
M33178.x	rs7565704	A	C	0.98	0.3367	0.0741	5.58E-06	20.6467
M33178.x	rs757128	A	G	0.02	-0.3367	0.0741	5.58E-06	20.6467
M33178.x	rs7599592	A	G	0.9773	0.3463	0.0765	5.93E-06	20.4919
M33178.x	rs7622961	A	G	0.021	-0.3374	0.0743	5.58E-06	20.6211
M33178.x	rs7637257	T	C	0.98	0.3367	0.0741	5.58E-06	20.6467
M33178.x	rs7643344	A	G	0.0208	-0.3388	0.0738	4.47E-06	21.0753
M33178.x	rs7674006	T	C	0.9415	0.131	0.0293	7.98E-06	19.9897
M33178.x	rs7675814	A	C	0.9773	0.3441	0.073	2.46E-06	22.219
M33178.x	rs7701941	C	G	0.0231	-0.3413	0.0756	6.34E-06	20.3812
M33178.x	rs7722027	T	C	0.021	-0.3374	0.0743	5.58E-06	20.6211
M33178.x	rs7722181	T	C	0.02	-0.3367	0.0741	5.58E-06	20.6467
M33178.x	rs7728290	A	G	0.021	-0.337	0.0742	5.58E-06	20.6278
M33178.x	rs7746400	T	C	0.04	-0.1683	0.0371	5.58E-06	20.5788
M33178.x	rs7755459	A	C	0.9804	0.3099	0.0632	9.49E-07	24.0441
M33178.x	rs7760073	A	G	0.98	0.3367	0.0741	5.58E-06	20.6467
M33178.x	rs7800783	A	G	0.02	-0.3367	0.0741	5.58E-06	20.6467
M33178.x	rs7822943	A	G	0.98	0.3367	0.0741	5.58E-06	20.6467
M33178.x	rs7854415	T	G	0.0211	-0.3374	0.0743	5.59E-06	20.6211
M33178.x	rs7862319	A	G	0.9721	0.3256	0.0726	7.29E-06	20.1139
M33178.x	rs7912365	A	G	0.9765	0.3439	0.0752	4.85E-06	20.9136
M33178.x	rs7929278	A	G	0.0217	-0.3408	0.0736	3.63E-06	21.4409
M33178.x	rs7941283	A	G	0.979	0.3374	0.0743	5.58E-06	20.6211
M33178.x	rs794174	A	C	0.48	0.3367	0.0741	5.58E-06	20.6467

M33178.x	rs7942841	A	G	0.8086	-0.0917	0.02	4.42E-06	21.0222
M33178.x	rs7944027	A	G	0.0253	-0.3473	0.0743	3E-06	21.849
M33178.x	rs7960522	T	C	0.0208	-0.3405	0.075	5.57E-06	20.6116
M33178.x	rs7963305	T	C	0.98	0.3367	0.0741	5.58E-06	20.6467
M33178.x	rs7967567	A	G	0.9783	0.3404	0.0746	5.01E-06	20.821
M33178.x	rs8001962	A	G	0.02	-0.3367	0.0741	5.58E-06	20.6467
M33178.x	rs8002031	A	G	0.02	-0.3367	0.0741	5.58E-06	20.6467
M33178.x	rs8052201	A	T	0.98	0.3367	0.0741	5.58E-06	20.6467
M33178.x	rs8082603	C	G	0.024	-0.3384	0.0736	4.23E-06	21.14
M33178.x	rs8087408	C	G	0.9606	0.1781	0.0392	5.39E-06	20.6422
M33178.x	rs8089286	A	G	0.0208	-0.3391	0.0738	4.32E-06	21.1127
M33178.x	rs8099023	A	G	0.9783	0.3411	0.0735	3.47E-06	21.5372
M33178.x	rs8127316	A	G	0.98	0.3367	0.0741	5.58E-06	20.6467
M33178.x	rs8141113	A	C	0.02	-0.3367	0.0741	5.58E-06	20.6467
M33178.x	rs8191547	A	G	0.9769	0.3408	0.0751	5.61E-06	20.593
M33178.x	rs8192827	A	G	0.9773	0.3441	0.073	2.46E-06	22.219
M33178.x	rs860640	T	C	0.0483	-0.3427	0.0748	4.69E-06	20.9906
M33178.x	rs872872	T	C	0.02	-0.3367	0.0741	5.58E-06	20.6467
M33178.x	rs875439	A	G	0.98	0.3367	0.0741	5.58E-06	20.6467
M33178.x	rs876255	T	C	0.9524	0.171	0.0368	3.38E-06	21.5922
M33178.x	rs890290	T	C	0.0266	-0.3402	0.0724	2.6E-06	22.0796
M33178.x	rs897187	A	C	0.9783	0.3378	0.0739	4.91E-06	20.8944
M33178.x	rs909782	T	C	0.02	-0.3367	0.0741	5.58E-06	20.6467
M33178.x	rs922955	A	G	0.9783	0.3411	0.0735	3.47E-06	21.5372
M33178.x	rs9347883	A	T	0.02	-0.3367	0.0741	5.58E-06	20.6467
M33178.x	rs9351099	T	C	0.02	-0.3367	0.0741	5.58E-06	20.6467
M33178.x	rs9365901	A	G	0.02	-0.3367	0.0741	5.58E-06	20.6467
M33178.x	rs937589	T	C	0.02	-0.3367	0.0741	5.58E-06	20.6467
M33178.x	rs9389954	T	C	0.8466	0.119	0.0246	1.3E-06	23.4004
M33178.x	rs9395772	A	G	0.0176	-0.2838	0.062	4.67E-06	20.9528
M33178.x	rs9398363	T	C	0.0232	-0.2359	0.0509	3.58E-06	21.4793
M33178.x	rs9401158	A	G	0.0266	-0.2648	0.0522	3.91E-07	25.7333
M33178.x	rs941880	A	C	0.021	-0.337	0.0742	5.58E-06	20.6278
M33178.x	rs9504399	A	G	0.024	-0.3459	0.0756	4.8E-06	20.9343
M33178.x	rs9508747	T	C	0.02	-0.3367	0.0741	5.58E-06	20.6467
M33178.x	rs9513184	A	C	0.979	0.3381	0.0744	5.53E-06	20.6512
M33178.x	rs9530833	T	C	0.7809	0.1003	0.0225	8.47E-06	19.8718
M33178.x	rs9532905	C	G	0.9793	0.3496	0.0765	4.89E-06	20.8843
M33178.x	rs9533206	T	G	0.9773	0.3376	0.0744	5.61E-06	20.5901
M33178.x	rs9546669	A	G	0.9792	0.3374	0.0741	5.32E-06	20.7326
M33178.x	rs9559615	T	G	0.0188	-0.2969	0.064	3.43E-06	21.5209
M33178.x	rs9562411	T	G	0.9478	0.1795	0.0394	5.2E-06	20.7557
M33178.x	rs9574326	A	G	0.9791	0.3391	0.0747	5.58E-06	20.607
M33178.x	rs9574354	T	C	0.02	-0.3367	0.0741	5.58E-06	20.6467
M33178.x	rs9574766	A	G	0.025	-0.3499	0.0722	1.25E-06	23.4862
M33178.x	rs9603511	C	G	0.98	0.3367	0.0741	5.58E-06	20.6467
M33178.x	rs9637871	A	G	0.0212	-0.3376	0.0743	5.51E-06	20.6456
M33178.x	rs9672326	T	G	0.9622	0.155	0.0338	4.44E-06	21.0296
M33178.x	rs9685506	T	C	0.9781	0.3382	0.0743	5.39E-06	20.719
M33178.x	rs9689446	T	C	0.962	0.1635	0.0335	1.04E-06	23.8202
M33178.x	rs974135	T	C	0.2826	-0.0912	0.0183	5.84E-07	24.8363
M33178.x	rs980466	C	G	0.0214	-0.3394	0.0747	5.55E-06	20.6435
M33178.x	rs9838311	C	G	0.9788	0.3378	0.0743	5.39E-06	20.6701
M33178.x	rs9862737	T	G	0.02	-0.3367	0.0741	5.58E-06	20.6467
M33178.x	rs9865616	A	G	0.98	0.2891	0.0541	8.92E-08	28.5563

M33178.x	rs9896355	T	C	0.96	0.1683	0.0371	5.58E-06	20.5788
M33178.x	rs9944753	T	C	0.0208	-0.339	0.0738	4.37E-06	21.1002
M33178.x	rs9957162	A	G	0.979	0.3375	0.0743	5.5E-06	20.6334
M33178.x	rs9995621	A	G	0.02	-0.3367	0.0741	5.58E-06	20.6467
M33178.x	rs9995884	A	G	0.0229	-0.3405	0.074	4.2E-06	21.1724
M33188.m	rs2697339	T	C	0.7684	0.0462	0.0102	6.39E-06	20.5156
M33188.m	rs3780277	A	G	0.0847	0.1767	0.0372	2.07E-06	22.5625
M33188.m	rs8017593	A	C	0.471	-0.0452	0.0101	7.07E-06	20.0278
M33190.m	rs10878448	A	G	0.0829	0.0944	0.0207	5.21E-06	20.7971
M33190.m	rs12004211	A	T	0.0102	0.391	0.0835	2.85E-06	21.9271
M33190.m	rs12104569	T	C	0.0335	0.2036	0.0459	9.25E-06	19.6757
M33190.m	rs12674822	T	G	0.3945	-0.0501	0.0106	2.22E-06	22.339
M33190.m	rs1602827	T	C	0.8337	0.1032	0.0232	8.38E-06	19.7872
M33190.m	rs17083735	A	C	0.8908	0.0941	0.0212	9.34E-06	19.7019
M33190.m	rs2392757	A	G	0.2235	-0.0572	0.0121	2.12E-06	22.3471
M33190.m	rs2486001	T	C	0.1433	0.0702	0.0152	3.91E-06	21.3298
M33190.m	rs2753980	A	G	0.6032	-0.0793	0.0176	6.74E-06	20.3012
M33190.m	rs2822577	T	C	0.3412	0.0512	0.0107	1.56E-06	22.8967
M33190.m	rs2906297	A	G	0.2427	0.0554	0.012	4.13E-06	21.3136
M33190.m	rs2932286	A	G	0.8764	0.0717	0.0159	6.83E-06	20.335
M33190.m	rs4233480	A	G	0.5412	0.0473	0.0104	5.43E-06	20.685
M33190.m	rs4542650	T	C	0.1769	0.0624	0.0134	3.19E-06	21.685
M33190.m	rs593634	C	G	0.1084	0.0763	0.0165	3.5E-06	21.3836
M33190.m	rs6426944	A	G	0.2621	-0.0568	0.0118	1.55E-06	23.1704
M33190.m	rs6765892	T	C	0.0793	0.1002	0.0216	3.63E-06	21.5193
M33190.m	rs6904241	T	G	0.5433	0.0527	0.0105	5.36E-07	25.1908
M33190.m	rs7107926	T	C	0.8864	-0.0663	0.0149	9.06E-06	19.7995
M33190.m	rs9298802	T	C	0.9866	-0.3291	0.073	6.53E-06	20.324
M33190.m	rs944495	T	C	0.5097	-0.0798	0.0174	4.69E-06	21.0333
M33192.m	rs1153452	T	C	0.8392	-0.0616	0.0133	3.62E-06	21.4515
M33192.m	rs11687942	T	G	0.5576	-0.0484	0.0097	6.03E-07	24.897
M33192.m	rs12339963	C	G	0.0852	0.0789	0.0171	3.71E-06	21.2893
M33192.m	rs12529004	A	G	0.9751	0.146	0.0318	4.39E-06	21.0791
M33192.m	rs13374698	T	C	0.0326	0.1171	0.0265	9.89E-06	19.5264
M33192.m	rs17054219	A	C	0.3934	0.0829	0.0172	1.54E-06	23.2302
M33192.m	rs17068261	T	C	0.9803	-0.1633	0.0359	5.33E-06	20.6911
M33192.m	rs4894432	A	G	0.7331	-0.0475	0.0105	6.85E-06	20.4649
M33192.m	rs6507387	T	C	0.3592	-0.0469	0.0104	7.06E-06	20.3366
M33192.m	rs6718711	A	G	0.3941	-0.0432	0.0097	9.03E-06	19.8346
M33192.m	rs6878813	T	C	0.1376	0.0672	0.0143	2.62E-06	22.0834
M33192.m	rs944495	T	C	0.5131	-0.0906	0.0205	9.76E-06	19.5321
M33192.m	rs9892714	T	C	0.031	0.1324	0.0289	4.67E-06	20.9884
M33194.m	rs10485517	A	G	0.4042	-0.0427	0.0095	7.29E-06	20.2027
M33194.m	rs12413610	T	C	0.9528	-0.2541	0.0525	1.29E-06	23.4256
M33194.m	rs12607750	C	G	0.297	-0.0492	0.0111	9.74E-06	19.6465
M33194.m	rs16839632	A	G	0.9359	0.0957	0.0199	1.51E-06	23.1269
M33194.m	rs2377394	A	T	0.0675	-0.1095	0.0243	6.84E-06	20.3056
M33194.m	rs2959857	A	T	0.2731	0.0988	0.0214	3.81E-06	21.315
M33194.m	rs4126458	A	G	0.5107	0.0887	0.0189	2.83E-06	22.0254
M33194.m	rs432097	A	G	0.2928	-0.048	0.0104	4.08E-06	21.3018
M33194.m	rs6507387	T	C	0.3593	-0.0503	0.0103	1.04E-06	23.8485
M33194.m	rs6569557	T	C	0.5446	-0.0843	0.019	9.38E-06	19.6856
M33194.m	rs6574474	A	G	0.5481	0.046	0.0096	1.52E-06	22.9601
M33194.m	rs8059937	T	G	0.8405	-0.0605	0.0135	7.88E-06	20.0837
M33194.m	rs954483	A	G	0.8406	0.0616	0.0137	6.81E-06	20.2172

M33195.m	rs11192313	A	G	0.4433	0.0428	0.0093	3.69E-06	21.1798
M33195.m	rs12566852	T	C	0.7395	0.0515	0.0112	4.7E-06	21.1436
M33195.m	rs17556249	T	C	0.0315	0.1361	0.0302	6.43E-06	20.3096
M33195.m	rs2159190	C	G	0.8227	-0.0552	0.0123	7.4E-06	20.1404
M33195.m	rs2298126	A	C	0.3406	-0.0484	0.0101	1.57E-06	22.964
M33195.m	rs2490700	C	G	0.0343	0.1208	0.0269	7.35E-06	20.1664
M33195.m	rs2493950	T	C	0.9465	-0.2434	0.0497	9.9E-07	23.9844
M33197.m	rs10118949	T	C	0.0815	0.1001	0.0207	1.31E-06	23.3845
M33197.m	rs12927299	A	T	0.7061	-0.05	0.0108	3.99E-06	21.4335
M33197.m	rs1457182	A	G	0.982	-0.194	0.042	3.86E-06	21.3356
M33197.m	rs17826680	A	G	0.8905	-0.0813	0.0179	5.29E-06	20.6289
M33197.m	rs2163086	A	G	0.2974	0.0498	0.0108	3.93E-06	21.2623
M33197.m	rs7595056	T	C	0.7661	0.0533	0.0117	5.38E-06	20.7531
M33197.m	rs7732078	A	G	0.6742	0.0524	0.0108	1.23E-06	23.5405
M33197.m	rs7916966	A	T	0.9755	-0.3985	0.088	5.92E-06	20.5065
M33197.m	rs9656510	A	G	0.0464	0.252	0.0527	1.71E-06	22.8655
M33203.m	rs10433891	A	G	0.8713	-0.079	0.0177	7.95E-06	19.9208
M33203.m	rs10779088	T	C	0.0566	0.1172	0.0263	8.61E-06	19.8584
M33203.m	rs10805908	A	T	0.8889	-0.0823	0.0186	9.86E-06	19.5782
M33203.m	rs11737198	T	C	0.9824	-0.3889	0.0827	2.59E-06	22.1139
M33203.m	rs11783678	C	G	0.8364	-0.0916	0.0201	5.45E-06	20.7682
M33203.m	rs11815032	T	C	0.0578	0.1134	0.0252	6.97E-06	20.25
M33203.m	rs11885171	T	C	0.9738	-0.2846	0.0639	8.51E-06	19.8366
M33203.m	rs12052015	C	G	0.6242	-0.056	0.0125	7.3E-06	20.0704
M33203.m	rs155146	T	C	0.9834	-0.4768	0.0885	7.24E-08	29.0259
M33203.m	rs1710456	A	G	0.292	0.1077	0.0239	6.56E-06	20.3065
M33203.m	rs17122180	T	C	0.9553	-0.1494	0.0313	1.82E-06	22.7831
M33203.m	rs1866724	T	C	0.7129	-0.0688	0.0136	4.25E-07	25.5917
M33203.m	rs2287358	T	C	0.0386	0.1375	0.0307	7.44E-06	20.0599
M33203.m	rs2630821	C	G	0.8534	-0.1273	0.0285	7.69E-06	19.9511
M33203.m	rs7693835	A	G	0.9106	-0.1204	0.0253	2.01E-06	22.6471
M33203.m	rs885479	A	G	0.0535	0.1621	0.028	7E-09	33.5158
M33203.m	rs992907	T	C	0.5555	0.0563	0.0127	9.54E-06	19.6521
M33204.m	rs10952210	T	C	0.1046	0.0282	0.0061	4.38E-06	21.3717
M33204.m	rs11133063	T	G	0.5241	-0.0135	0.003	6.74E-06	20.25
M33204.m	rs1327266	T	C	0.9338	0.0357	0.0077	3.88E-06	21.4959
M33204.m	rs16944339	A	T	0.1526	-0.0248	0.0052	1.99E-06	22.7456
M33204.m	rs16984993	T	C	0.0398	0.0364	0.0079	4.43E-06	21.2299
M33204.m	rs17418798	T	C	0.9675	-0.0535	0.0116	4.24E-06	21.2712
M33204.m	rs17552447	T	C	0.9767	0.0563	0.0125	6.79E-06	20.286
M33204.m	rs2736780	T	C	0.6897	0.0182	0.0041	9.53E-06	19.7049
M33204.m	rs280302	T	C	0.8923	0.0221	0.005	8.36E-06	19.5364
M33204.m	rs4807944	T	C	0.1282	0.0206	0.0044	3.32E-06	21.9194
M33204.m	rs7953025	A	G	0.5419	0.0172	0.0038	7.61E-06	20.4875
M33204.m	rs822531	T	C	0.7832	0.0181	0.0041	9.47E-06	19.489
M33204.m	rs9521845	A	G	0.3291	0.0154	0.0034	7.19E-06	20.5156
M33221.m	rs10006433	T	C	0.2626	-0.0211	0.0046	4.02E-06	21.0402
M33221.m	rs11669776	T	C	0.138	0.0278	0.0062	8.01E-06	20.1051
M33221.m	rs12653732	C	G	0.3139	0.0202	0.0045	8.14E-06	20.1501
M33221.m	rs1442060	A	G	0.5118	-0.0236	0.0052	6.81E-06	20.5976
M33221.m	rs17137638	A	G	0.0135	0.0845	0.0187	6.37E-06	20.4188
M33221.m	rs17330336	C	G	0.0771	-0.0327	0.0073	8.35E-06	20.0655
M33221.m	rs1865099	A	G	0.6476	0.0212	0.0045	2.6E-06	22.1946
M33221.m	rs2108622	T	C	0.2995	0.0231	0.0046	5.3E-07	25.2179
M33221.m	rs2711484	A	G	0.3034	-0.0218	0.0046	1.89E-06	22.4594

M33221.m	rs277311	T	C	0.1536	0.0259	0.0056	4.06E-06	21.3906
M33221.m	rs2816826	T	C	0.2779	0.0213	0.0046	2.82E-06	21.4409
M33221.m	rs4149081	A	G	0.171	0.0243	0.0054	6.66E-06	20.25
M33221.m	rs6904836	T	C	0.093	0.0323	0.0073	9.18E-06	19.5776
M33221.m	rs6921033	A	G	0.0116	0.1937	0.0426	5.34E-06	20.6747
M33221.m	rs7254306	A	G	0.3907	0.0213	0.0046	3.25E-06	21.4409
M33221.m	rs8796	T	G	0.1633	0.0634	0.0136	3.21E-06	21.7321
M33221.m	rs9615728	A	G	0.0399	0.0543	0.0114	2.01E-06	22.6877
M33221.m	rs980833	T	C	0.9438	0.0445	0.0098	5.99E-06	20.619
M33228.m	rs10233803	T	C	0.5881	0.0121	0.0027	7.69E-06	20.0837
M33228.m	rs11202064	A	G	0.906	-0.0202	0.0046	9.5E-06	19.2836
M33228.m	rs11954991	A	T	0.0131	0.121	0.0269	6.85E-06	20.2333
M33228.m	rs12463674	A	G	0.6888	0.015	0.0028	5.14E-08	28.699
M33228.m	rs13254318	T	C	0.2929	0.0122	0.0027	7.58E-06	20.417
M33228.m	rs1692120	A	G	0.4589	0.0188	0.0027	5.82E-12	48.4829
M33228.m	rs169660	A	G	0.3667	-0.0126	0.0029	9.22E-06	18.8775
M33228.m	rs174535	T	C	0.6602	0.0561	0.0027	1.86E-94	431.716
M33228.m	rs1845546	T	C	0.3674	-0.0126	0.0027	3.25E-06	21.7778
M33228.m	rs198435	C	G	0.1051	0.0204	0.0046	8.72E-06	19.6673
M33228.m	rs203846	T	C	0.6027	-0.013	0.0027	1.63E-06	23.1824
M33228.m	rs227769	T	C	0.467	-0.0121	0.0027	9.32E-06	20.0837
M33228.m	rs3738544	T	C	0.8071	0.0203	0.0035	8.05E-09	33.64
M33228.m	rs418874	A	G	0.6931	0.0126	0.0027	3.64E-06	21.7778
M33228.m	rs4392177	A	G	0.547	-0.014	0.003	3.06E-06	21.7778
M33228.m	rs540613	T	C	0.1462	0.0214	0.0045	1.92E-06	22.6153
M33228.m	rs6517062	A	G	0.3577	0.0132	0.0028	1.57E-06	22.2245
M33228.m	rs6784447	A	G	0.3405	0.014	0.0027	2.91E-07	26.8861
M33228.m	rs706041	T	C	0.3351	-0.0125	0.0027	4.45E-06	21.4335
M33228.m	rs7104849	A	G	0.9166	0.0343	0.0052	3.26E-11	43.5092
M33228.m	rs748196	T	G	0.4596	0.0152	0.0027	1.7E-08	31.6927
M33228.m	rs8127620	C	G	0.1786	-0.0223	0.0049	5.93E-06	20.7118
M33228.m	rs823554	A	T	0.8205	-0.0188	0.0036	2.45E-07	27.2716
M33228.m	rs9462177	A	G	0.6032	0.0138	0.0027	4.82E-07	26.1235
M33230.m	rs10255688	T	C	0.4167	-0.0122	0.0028	9.89E-06	18.9847
M33230.m	rs12164231	T	G	0.1331	0.02	0.0045	9.67E-06	19.7531
M33230.m	rs13140287	A	G	0.5842	0.0133	0.0028	1.59E-06	22.5625
M33230.m	rs1572948	A	G	0.4296	-0.0126	0.0028	4.98E-06	20.25
M33230.m	rs16923647	T	C	0.1285	0.0202	0.0044	4.57E-06	21.0764
M33230.m	rs2657880	C	G	0.136	0.021	0.0044	2.09E-06	22.7789
M33230.m	rs3811273	A	G	0.9383	-0.0281	0.0062	5.13E-06	20.5414
M33230.m	rs4240475	A	C	0.0592	0.0286	0.0063	6.21E-06	20.6087
M33230.m	rs569688	T	G	0.2186	0.017	0.0036	2.49E-06	22.2994
M33230.m	rs575528	A	G	0.5408	0.0127	0.0028	4.14E-06	20.5727
M33230.m	rs603424	A	G	0.181	-0.0274	0.0043	2.27E-10	40.6036
M33230.m	rs7301800	A	G	0.2124	0.0162	0.0036	8.73E-06	20.25
M33230.m	rs7603154	A	G	0.5252	0.0134	0.0028	1.18E-06	22.9031
M33230.m	rs9315938	T	C	0.0442	0.0323	0.0072	6.92E-06	20.1252
M33250.m	rs10181532	A	G	0.0285	-0.0633	0.0142	7.97E-06	19.8715
M33250.m	rs10505870	A	G	0.2729	0.0264	0.0048	4.79E-08	30.25
M33250.m	rs11049633	A	G	0.9833	0.0816	0.0176	3.41E-06	21.4959
M33250.m	rs11211406	A	C	0.7964	0.0279	0.0058	1.45E-06	23.1394
M33250.m	rs12370842	A	G	0.175	-0.0588	0.0058	6.67E-24	102.778
M33250.m	rs12629322	T	C	0.7183	-0.0233	0.0048	1.44E-06	23.5629
M33250.m	rs12810446	T	C	0.265	-0.0231	0.0049	1.99E-06	22.2245
M33250.m	rs12820524	T	C	0.9861	0.0959	0.0216	8.77E-06	19.712

M33250.m	rs13432322	T	C	0.016	-0.0933	0.0193	1.27E-06	23.3695
M33250.m	rs17207792	T	C	0.1399	0.0429	0.009	1.91E-06	22.7211
M33250.m	rs17479238	C	G	0.0642	-0.0436	0.0094	3.76E-06	21.5138
M33250.m	rs1994459	A	G	0.9529	-0.1746	0.0389	7.14E-06	20.146
M33250.m	rs2016979	T	C	0.7289	0.0304	0.0068	7.48E-06	19.9862
M33250.m	rs2291073	T	G	0.9272	0.0387	0.0087	7.91E-06	19.7872
M33250.m	rs2526978	T	C	0.2984	-0.0228	0.0049	2.63E-06	21.651
M33250.m	rs2561284	T	G	0.1581	0.1012	0.0226	7.71E-06	20.0514
M33250.m	rs3093198	A	G	0.279	0.024	0.005	1.59E-06	23.04
M33250.m	rs4149056	T	C	0.8367	-0.0827	0.0057	1.08E-47	210.504
M33250.m	rs4676262	T	C	0.4367	0.0798	0.0171	2.91E-06	21.7778
M33250.m	rs4767064	A	G	0.3478	0.0216	0.0048	8.17E-06	20.25
M33250.m	rs4775058	A	G	0.0236	0.0663	0.0144	3.85E-06	21.1984
M33250.m	rs7238314	T	G	0.9792	-0.0775	0.0161	1.49E-06	23.1714
M33250.m	rs7926241	A	G	0.0132	0.0887	0.0199	8.34E-06	19.8674
M33353.m	rs10089042	A	C	0.9474	-0.2008	0.0435	3.84E-06	21.3083
M33353.m	rs11151898	A	G	0.3841	0.0407	0.0092	9.23E-06	19.571
M33353.m	rs11782826	T	C	0.1295	-0.0579	0.0131	9.76E-06	19.5351
M33353.m	rs12050440	A	T	0.656	-0.044	0.0087	4.1E-07	25.578
M33353.m	rs12051797	A	G	0.923	-0.0812	0.0176	4.01E-06	21.2856
M33353.m	rs12441436	A	T	0.0273	-0.1298	0.0282	4.31E-06	21.1861
M33353.m	rs12544916	C	G	0.0372	-0.1079	0.0225	1.72E-06	22.9974
M33353.m	rs13054961	A	G	0.2239	0.05	0.0102	9.07E-07	24.0292
M33353.m	rs130798	A	T	0.1603	0.0578	0.0124	2.99E-06	21.7276
M33353.m	rs1343863	A	G	0.7954	-0.0601	0.0118	3.8E-07	25.9409
M33353.m	rs1681855	T	C	0.2032	0.0507	0.0109	3.43E-06	21.6353
M33353.m	rs17279964	A	G	0.9618	-0.1137	0.0249	4.96E-06	20.8508
M33353.m	rs1836845	T	G	0.6005	-0.0399	0.0085	2.73E-06	22.0347
M33353.m	rs2320173	T	C	0.3067	-0.0493	0.0094	1.35E-07	27.5067
M33353.m	rs2695415	T	C	0.8899	-0.1075	0.0223	1.44E-06	23.2385
M33353.m	rs3751035	A	G	0.1812	0.0802	0.0166	1.4E-06	23.3417
M33353.m	rs4325604	T	C	0.0727	0.0771	0.0161	1.63E-06	22.9328
M33353.m	rs6565887	T	C	0.927	-0.0773	0.0165	2.9E-06	21.9478
M33353.m	rs6760934	T	C	0.9725	-0.1921	0.0385	5.91E-07	24.8962
M33353.m	rs7425298	A	T	0.9846	-0.2286	0.0489	2.88E-06	21.8542
M33353.m	rs911685	T	G	0.9276	-0.0771	0.0166	3.2E-06	21.5721
M33359.m	rs10000111	A	G	0.0351	0.2505	0.0558	7.12E-06	20.1533
M33359.m	rs11594256	A	T	0.0335	-0.1101	0.0242	5.34E-06	20.6987
M33359.m	rs12731852	T	C	0.2589	0.0447	0.0101	9.5E-06	19.5872
M33359.m	rs1681529	A	G	0.0182	0.4453	0.0799	2.49E-08	31.0607
M33359.m	rs2207775	A	G	0.5486	0.0454	0.0091	6.13E-07	24.8902
M33359.m	rs2792707	T	C	0.0427	0.0984	0.0216	5.1E-06	20.7531
M33359.m	rs6938176	A	G	0.4494	0.0842	0.0188	7.45E-06	20.059
M33359.m	rs730947	A	C	0.8606	-0.0658	0.013	4.2E-07	25.6192
M33359.m	rs7799018	T	C	0.427	-0.0433	0.009	1.62E-06	23.1468
M33363.m	rs10732795	T	C	0.1261	-0.025	0.0056	7.34E-06	19.9298
M33363.m	rs12469856	A	T	0.707	-0.0204	0.0043	2.12E-06	22.5073
M33363.m	rs17165467	T	C	0.1252	0.0358	0.0072	7.83E-07	24.723
M33363.m	rs2416328	T	C	0.7592	0.02	0.0044	6.35E-06	20.6612
M33363.m	rs2642911	T	G	0.6151	0.0178	0.0039	4.53E-06	20.831
M33363.m	rs4416818	A	G	0.2365	0.0213	0.0046	2.79E-06	21.4409
M33363.m	rs6903432	C	G	0.0183	-0.069	0.0147	2.77E-06	22.0325
M33363.m	rs7083972	A	T	0.2206	-0.0292	0.0057	3.63E-07	26.2432
M33363.m	rs8011328	A	G	0.0129	0.1081	0.024	6.59E-06	20.2875
M33364.m	rs11881687	A	G	0.779	-0.0273	0.0056	1.06E-06	23.7656

M33364.m	rs12117697	T	C	0.7484	0.0192	0.0042	5.24E-06	20.898
M33364.m	rs12216313	A	T	0.0351	-0.0429	0.0094	5.49E-06	20.8285
M33364.m	rs17133359	A	G	0.9531	-0.0403	0.0083	1.07E-06	23.5751
M33364.m	rs2062528	C	G	0.6616	0.0175	0.0036	9.19E-07	23.6304
M33364.m	rs2291502	T	C	0.0926	-0.0258	0.0058	8.7E-06	19.7872
M33364.m	rs310104	T	C	0.0428	-0.0558	0.0116	1.52E-06	23.1394
M33364.m	rs3791608	A	G	0.1287	-0.0221	0.0049	7E-06	20.3419
M33364.m	rs6902872	T	C	0.6467	0.0163	0.0036	4.89E-06	20.5008
M33364.m	rs7432894	T	C	0.6903	-0.0179	0.0037	1.82E-06	23.4047
M33380.m	rs10234068	A	G	0.012	-0.061	0.0137	8.88E-06	19.8252
M33380.m	rs10986087	T	C	0.3105	-0.0075	0.0017	9.54E-06	19.4637
M33380.m	rs11212873	C	G	0.7979	-0.0088	0.0018	7.82E-07	23.9012
M33380.m	rs1154486	T	C	0.943	0.0183	0.004	3.71E-06	20.9306
M33380.m	rs11578636	T	G	0.0162	-0.0523	0.0116	6.24E-06	20.3277
M33380.m	rs11780588	T	G	0.9029	0.0114	0.0026	9.16E-06	19.2249
M33380.m	rs12283819	A	G	0.1003	-0.0119	0.0026	4.01E-06	20.9482
M33380.m	rs12701943	T	C	0.9372	0.0335	0.0075	8.56E-06	19.9511
M33380.m	rs12821224	T	C	0.0348	0.0226	0.0048	2.11E-06	22.1684
M33380.m	rs12923572	A	G	0.2397	0.0082	0.0017	2.92E-06	23.2664
M33380.m	rs13079159	A	C	0.5539	0.0144	0.0032	5.8E-06	20.25
M33380.m	rs1363574	A	G	0.9545	-0.0195	0.004	1.29E-06	23.7656
M33380.m	rs1509091	T	C	0.9357	0.0151	0.0033	6.3E-06	20.9376
M33380.m	rs1564511	T	C	0.8539	-0.0194	0.0043	6.12E-06	20.3548
M33380.m	rs16945323	A	T	0.2334	-0.0082	0.0018	2.72E-06	20.7531
M33380.m	rs17642254	T	G	0.0886	0.0122	0.0026	4.06E-06	22.0178
M33380.m	rs1874617	A	G	0.7831	-0.0083	0.0018	4.23E-06	21.2623
M33380.m	rs208357	T	G	0.25	-0.009	0.002	6.8E-06	20.25
M33380.m	rs2492943	T	C	0.3964	0.0076	0.0017	8.68E-06	19.9862
M33380.m	rs2500264	A	G	0.2536	0.0084	0.0017	1.39E-06	24.4152
M33380.m	rs28157	T	G	0.3075	-0.0077	0.0017	6.68E-06	20.5156
M33380.m	rs2830527	T	C	0.2444	0.0078	0.0017	5.51E-06	21.0519
M33380.m	rs2830527	T	C	0.2444	0.0078	0.0017	5.51E-06	21.0519
M33380.m	rs307811	A	G	0.3635	-0.008	0.0018	4.35E-06	19.7531
M33380.m	rs4517836	T	C	0.7357	0.0082	0.0018	3E-06	20.7531
M33380.m	rs4624748	A	G	0.2344	-0.0081	0.0018	3.81E-06	20.25
M33380.m	rs4748632	A	G	0.8015	0.0086	0.0018	1.45E-06	22.8272
M33380.m	rs4919062	A	C	0.9652	0.0198	0.0042	3.17E-06	22.2245
M33380.m	rs6080978	T	C	0.299	-0.008	0.0017	3.18E-06	22.1453
M33380.m	rs6605235	T	C	0.7072	-0.008	0.0017	3.54E-06	22.1453
M33380.m	rs688608	A	G	0.778	-0.0083	0.0018	2.61E-06	21.2623
M33380.m	rs7526119	A	G	0.1692	-0.0136	0.0028	1.77E-06	23.5918
M33380.m	rs7624812	T	C	0.1734	-0.0087	0.0019	2.77E-06	20.9668
M33380.m	rs8024828	A	G	0.9056	0.0118	0.0027	8.9E-06	19.1001
M33380.m	rs8058227	T	C	0.4024	0.0081	0.0017	1.35E-06	22.7024
M33380.m	rs8130833	A	G	0.707	-0.01	0.002	5.73E-07	25
M33380.m	rs835800	T	C	0.2665	0.0077	0.0017	9.61E-06	20.5156
M33380.m	rs870031	A	G	0.1164	0.0122	0.0025	1.27E-06	23.8144
M33380.m	rs9370455	T	C	0.8882	-0.0117	0.0025	2.35E-06	21.9024
M33380.m	rs941980	T	C	0.5798	0.0077	0.0017	4.23E-06	20.5156
M33380.m	rs944243	A	G	0.3604	-0.0091	0.0017	1.48E-07	28.654
M33380.m	rs9472134	A	T	0.4101	-0.0104	0.0023	4.55E-06	20.4461
M33384.x	rs10521182	T	C	0.9682	-0.3993	0.0786	3.71E-07	25.808
M33384.x	rs11242046	A	G	0.0216	0.4771	0.1051	5.6E-06	20.6069
M33384.x	rs163589	T	G	0.011	0.6297	0.136	3.63E-06	21.4383
M33384.x	rs16905117	A	T	0.0413	0.3196	0.0686	3.17E-06	21.7053

M33384.x	rs17011024	A	G	0.7728	-0.1149	0.0247	3.3E-06	21.6394
M33384.x	rs2250756	A	G	0.5542	-0.0946	0.0213	9.09E-06	19.7253
M33384.x	rs2722578	T	G	0.1259	0.1411	0.0304	3.38E-06	21.543
M33384.x	rs281083	A	G	0.1001	-0.1634	0.0351	3.24E-06	21.6715
M33384.x	rs308097	A	G	0.0377	0.3482	0.0747	3.16E-06	21.7278
M33384.x	rs3784457	A	G	0.0539	0.2165	0.0473	4.66E-06	20.9505
M33384.x	rs7145855	A	G	0.9691	0.3181	0.0708	7.07E-06	20.1865
M33384.x	rs9416100	T	C	0.0146	0.5637	0.1265	8.4E-06	19.8571
M33389.m	rs10110900	A	G	0.468	-0.0092	0.0018	4.83E-07	26.1235
M33389.m	rs10457440	T	C	0.0849	-0.018	0.0036	4.77E-07	25
M33389.m	rs10949241	T	C	0.5212	0.0087	0.0019	3.69E-06	20.9668
M33389.m	rs10965997	A	G	0.0399	-0.0299	0.0067	8.32E-06	19.9156
M33389.m	rs11577512	A	G	0.4077	0.0085	0.0019	5.28E-06	20.0139
M33389.m	rs11717068	T	C	0.6324	-0.0087	0.0019	2.94E-06	20.9668
M33389.m	rs11772826	A	G	0.323	0.01	0.002	5.73E-07	25
M33389.m	rs11786713	C	G	0.6543	-0.0094	0.0019	4.26E-07	24.4765
M33389.m	rs11898021	T	C	0.7118	0.0091	0.0019	1.06E-06	22.9391
M33389.m	rs12539066	T	C	0.3111	0.0092	0.0019	9.03E-07	23.446
M33389.m	rs1260326	T	C	0.4031	0.0087	0.0018	2.07E-06	23.3611
M33389.m	rs12884100	A	C	0.4607	-0.0089	0.0019	1.84E-06	21.9418
M33389.m	rs12996983	A	G	0.0786	-0.0167	0.0036	3.78E-06	21.5193
M33389.m	rs1532085	A	G	0.3863	0.0091	0.0019	9.24E-07	22.9391
M33389.m	rs1538133	A	G	0.2863	-0.0083	0.0019	8.49E-06	19.0831
M33389.m	rs17197518	A	G	0.6219	-0.0085	0.0019	4.05E-06	20.0139
M33389.m	rs17770168	A	G	0.7199	-0.0083	0.0019	9.28E-06	19.0831
M33389.m	rs1800775	A	C	0.4977	0.0128	0.0019	1.02E-11	45.385
M33389.m	rs1868852	A	C	0.8553	0.0145	0.0028	1.57E-07	26.8176
M33389.m	rs2265217	A	C	0.7395	0.0084	0.0019	8.4E-06	19.5457
M33389.m	rs236403	T	C	0.624	-0.0093	0.0019	5.87E-07	23.9584
M33389.m	rs2373262	T	C	0.705	-0.0087	0.0019	2.8E-06	20.9668
M33389.m	rs2514285	A	C	0.4794	-0.0084	0.0019	6.72E-06	19.5457
M33389.m	rs288514	T	C	0.5866	-0.0085	0.0018	4.14E-06	22.2994
M33389.m	rs2932919	A	G	0.6579	0.0082	0.0019	9.12E-06	18.626
M33389.m	rs2980872	A	G	0.461	-0.009	0.002	6.8E-06	20.25
M33389.m	rs33131	A	G	0.669	-0.0087	0.0019	3.12E-06	20.9668
M33389.m	rs342170	T	C	0.5871	-0.0086	0.0019	4.82E-06	20.4875
M33389.m	rs348661	A	T	0.3458	0.0089	0.0019	1.78E-06	21.9418
M33389.m	rs3807814	T	C	0.681	0.0083	0.0019	9.22E-06	19.0831
M33389.m	rs394811	A	G	0.399	-0.0082	0.0018	9.89E-06	20.7531
M33389.m	rs4144863	T	C	0.38	0.0083	0.0019	8.24E-06	19.0831
M33389.m	rs433110	A	T	0.2962	0.0086	0.0019	3.52E-06	20.4875
M33389.m	rs4602028	A	C	0.3766	-0.0088	0.0019	2.07E-06	21.4515
M33389.m	rs4694010	T	C	0.9318	-0.0163	0.0037	8.81E-06	19.4076
M33389.m	rs4700085	T	C	0.3754	0.0094	0.0018	2.98E-07	27.2716
M33389.m	rs52701	T	C	0.3727	0.0087	0.0019	2.69E-06	20.9668
M33389.m	rs588136	T	C	0.7976	-0.0137	0.0027	2.79E-07	25.7462
M33389.m	rs686189	A	G	0.5854	-0.0085	0.0018	3.86E-06	22.2994
M33389.m	rs6878354	C	G	0.3007	0.0085	0.0019	4.52E-06	20.0139
M33389.m	rs6977786	A	G	0.1753	-0.0124	0.0027	3.23E-06	21.0919
M33389.m	rs707	T	C	0.4558	-0.0083	0.0018	5.95E-06	21.2623
M33389.m	rs709947	C	G	0.6413	0.0086	0.0019	3.41E-06	20.4875
M33389.m	rs7311763	A	C	0.3591	-0.0091	0.0019	9.26E-07	22.9391
M33389.m	rs732787	A	G	0.377	0.0085	0.0019	5.27E-06	20.0139
M33389.m	rs7435399	T	C	0.66	0.0087	0.0019	2.48E-06	20.9668
M33389.m	rs7661478	T	C	0.6299	0.0083	0.0018	6.17E-06	21.2623

M33389.m	rs7760528	T	C	0.68	-0.0091	0.0019	9.17E-07	22.9391
M33389.m	rs928177	T	C	0.146	-0.0135	0.0027	7.14E-07	25
M33389.m	rs9375723	T	C	0.2664	0.0087	0.0019	3.39E-06	20.9668
M33389.m	rs939291	T	C	0.711	0.0089	0.0019	2.63E-06	21.9418
M33389.m	rs9471904	T	C	0.4795	-0.0082	0.0018	7.4E-06	20.7531
M33389.m	rs9567586	A	G	0.6031	0.0083	0.0019	8.32E-06	19.0831
M33389.m	rs962423	A	C	0.4645	-0.0087	0.0019	4.27E-06	20.9668
M33389.m	rs9818859	T	C	0.2937	0.0083	0.0019	7.83E-06	19.0831
M33389.m	rs9822180	T	C	0.7155	0.0099	0.0019	2.58E-07	27.1496
M33389.m	rs989127	T	C	0.7023	-0.0082	0.0019	9.44E-06	18.626
M33390.m	rs10873327	T	G	0.1828	0.0476	0.0103	3.68E-06	21.357
M33390.m	rs11019357	T	C	0.8389	-0.0459	0.0096	1.77E-06	22.8604
M33390.m	rs1160269	T	C	0.4442	-0.0326	0.0071	3.7E-06	21.0823
M33390.m	rs11769121	A	T	0.9758	-0.1068	0.0236	5.83E-06	20.4795
M33390.m	rs12092233	T	G	0.0315	0.1535	0.0321	1.69E-06	22.8669
M33390.m	rs12589666	T	G	0.3791	-0.0382	0.0072	1.26E-07	28.1489
M33390.m	rs16936991	A	G	0.0319	0.1488	0.0321	3.46E-06	21.488
M33390.m	rs16958823	T	C	0.9132	-0.0605	0.0123	9.48E-07	24.1936
M33390.m	rs17097622	T	C	0.9727	-0.104	0.0222	2.72E-06	21.9463
M33390.m	rs17166696	A	C	0.9655	-0.0897	0.0194	3.96E-06	21.3787
M33390.m	rs17463250	T	C	0.0671	0.1105	0.0228	1.31E-06	23.4885
M33390.m	rs17735981	T	C	0.9801	-0.1949	0.0405	1.51E-06	23.1587
M33390.m	rs4148622	A	G	0.2618	0.0398	0.008	7.66E-07	24.7506
M33390.m	rs4980117	T	G	0.4732	0.0314	0.0071	8.38E-06	19.5588
M33390.m	rs6716583	T	C	0.9211	-0.0745	0.0164	5.66E-06	20.636
M33391.m	rs10206020	T	C	0.8184	0.0814	0.0182	8E-06	20.0035
M33391.m	rs10784422	T	C	0.625	0.1355	0.0305	8.93E-06	19.7369
M33391.m	rs1117313	A	G	0.6636	0.068	0.0151	6.81E-06	20.2798
M33391.m	rs11736351	C	G	0.0263	0.516	0.092	2.02E-08	31.4575
M33391.m	rs12508311	A	T	0.9577	-0.4363	0.0793	3.71E-08	30.2708
M33391.m	rs12782140	A	G	0.0137	0.2983	0.0605	8.1E-07	24.3106
M33391.m	rs1336286	T	C	0.9493	-0.1665	0.033	4.54E-07	25.4566
M33391.m	rs1431419	T	C	0.9649	-0.3534	0.076	3.27E-06	21.6225
M33391.m	rs17748106	A	C	0.9287	-0.1185	0.0261	5.58E-06	20.6137
M33391.m	rs194546	A	G	0.9795	-0.4891	0.1065	4.37E-06	21.0909
M33391.m	rs2008827	T	G	0.0302	0.4093	0.0903	5.78E-06	20.5451
M33391.m	rs2927446	A	C	0.9737	-0.4392	0.0943	3.24E-06	21.6921
M33391.m	rs427619	T	C	0.0263	0.3498	0.0719	1.15E-06	23.6691
M33391.m	rs739484	T	C	0.0864	0.1354	0.0272	6.56E-07	24.7799
M33391.m	rs8094976	T	C	0.0131	0.6419	0.1405	4.93E-06	20.8729
M33391.m	rs904720	A	G	0.2414	-0.1106	0.0246	6.61E-06	20.2134
M33408.m	rs10263623	T	C	0.9157	-0.0569	0.0125	5.34E-06	20.7207
M33408.m	rs1518434	T	C	0.4027	-0.031	0.007	8.09E-06	19.6122
M33408.m	rs1609276	C	G	0.9727	0.1088	0.0228	1.83E-06	22.7713
M33408.m	rs16844404	A	G	0.9409	-0.1401	0.0273	2.97E-07	26.3361
M33408.m	rs16845462	T	C	0.9572	-0.0798	0.018	9.29E-06	19.6544
M33408.m	rs17698383	T	C	0.7946	0.0406	0.0085	1.88E-06	22.8147
M33408.m	rs2252553	T	G	0.4533	-0.0358	0.0069	1.82E-07	26.9196
M33408.m	rs296429	A	G	0.728	-0.0356	0.0077	3.52E-06	21.3756
M33408.m	rs6685925	A	G	0.2447	0.0364	0.0078	2.82E-06	21.7778
M33408.m	rs6717460	A	C	0.962	-0.1646	0.0351	2.77E-06	21.991
M33408.m	rs679020	T	C	0.9156	-0.0552	0.0123	7.03E-06	20.1404
M33408.m	rs7512146	T	G	0.5108	0.0332	0.0069	1.38E-06	23.1514
M33408.m	rs7708182	C	G	0.8862	-0.1012	0.022	4.41E-06	21.16
M33408.m	rs7767760	T	C	0.2932	0.0356	0.0076	2.97E-06	21.9418

M33415.m	rs10242455	A	G	0.9335	0.2212	0.0099	1.7E-109	499.229
M33415.m	rs11045825	T	C	0.834	0.0641	0.0067	1.62E-21	91.5306
M33415.m	rs11591344	A	T	0.0643	-0.045	0.0099	5.05E-06	20.6612
M33415.m	rs11644920	A	T	0.6891	-0.0233	0.0052	6.85E-06	20.0773
M33415.m	rs11762932	A	G	0.7833	0.0363	0.0059	6.25E-10	37.8538
M33415.m	rs11768283	A	G	0.0589	0.05	0.011	5.9E-06	20.6612
M33415.m	rs13174155	T	G	0.4549	-0.024	0.005	1.82E-06	23.04
M33415.m	rs13230554	T	C	0.016	-0.1701	0.0209	4.31E-16	66.2393
M33415.m	rs1883349	A	G	0.1609	-0.0318	0.0068	2.48E-06	21.8694
M33415.m	rs2267282	A	G	0.2957	0.0227	0.0051	9.85E-06	19.8112
M33415.m	rs2291073	T	G	0.9198	0.0546	0.0092	2.57E-09	35.2216
M33415.m	rs2857468	A	T	0.0946	-0.0594	0.01	2.45E-09	35.2836
M33415.m	rs296396	T	C	0.1628	-0.0409	0.0067	1.06E-09	37.2646
M33415.m	rs335124	A	G	0.285	0.023	0.0052	8.62E-06	19.5636
M33415.m	rs4110517	A	G	0.2043	-0.029	0.0065	8.63E-06	19.9053
M33415.m	rs4149056	T	C	0.8379	-0.1176	0.0065	1.26E-73	327.332
M33415.m	rs4917639	A	C	0.8015	-0.0362	0.006	1.39E-09	36.4011
M33415.m	rs4968836	A	C	0.594	0.0536	0.0105	3.55E-07	26.0586
M33415.m	rs6465760	A	G	0.3009	0.0416	0.0052	9.5E-16	64
M33415.m	rs6544713	T	C	0.3211	-0.0314	0.0051	8.97E-10	37.907
M33415.m	rs6719679	A	G	0.7751	0.0276	0.0058	2.2E-06	22.6445
M33415.m	rs7138177	A	G	0.8933	-0.0579	0.0084	4.97E-12	47.5115
M33415.m	rs7795644	T	C	0.0648	-0.1065	0.0127	4.76E-17	70.3221
M33415.m	rs7797978	T	G	0.7356	0.0413	0.0058	7.53E-13	50.7042
M33415.m	rs952319	T	C	0.1352	-0.0948	0.0075	1.39E-36	159.77
M33420.m	rs10077932	T	C	0.1376	-0.0402	0.0087	4.08E-06	21.3508
M33420.m	rs1013104	T	C	0.4354	-0.0207	0.0045	3.83E-06	21.16
M33420.m	rs10466757	A	T	0.1578	0.0634	0.0143	9.56E-06	19.6565
M33420.m	rs10492212	T	C	0.1606	-0.0274	0.0062	8.66E-06	19.5307
M33420.m	rs10520845	A	C	0.0165	0.191	0.0419	5.27E-06	20.7797
M33420.m	rs1060467	A	G	0.5903	0.0233	0.0045	2.61E-07	26.8094
M33420.m	rs13336771	A	C	0.1736	0.0624	0.0139	7.39E-06	20.153
M33420.m	rs261301	T	C	0.1314	0.0323	0.0068	2.06E-06	22.5625
M33420.m	rs2794327	T	C	0.6691	-0.0355	0.008	8.78E-06	19.6914
M33420.m	rs5994305	A	G	0.8318	0.0307	0.0062	7.15E-07	24.5185
M33420.m	rs6821770	A	G	0.143	0.038	0.0086	8.92E-06	19.5241
M33420.m	rs7038957	T	C	0.8312	-0.0287	0.0062	3.86E-06	21.4279
M33420.m	rs7350776	C	G	0.697	0.0239	0.0052	3.86E-06	21.1246
M33422.m	rs10116543	A	G	0.0816	0.0123	0.0027	6.81E-06	20.7531
M33422.m	rs10500747	T	C	0.7541	0.0083	0.0018	4.57E-06	21.2623
M33422.m	rs10874521	T	C	0.7176	0.008	0.0018	5.37E-06	19.7531
M33422.m	rs11007082	T	G	0.3768	0.0083	0.0017	1.95E-06	23.8374
M33422.m	rs11588513	T	C	0.955	0.0197	0.0044	9.58E-06	20.046
M33422.m	rs11623367	T	C	0.0594	0.016	0.0035	5.6E-06	20.898
M33422.m	rs119194	A	C	0.2105	-0.0091	0.0018	4.33E-07	25.5586
M33422.m	rs12144277	A	C	0.0297	-0.0635	0.014	5.46E-06	20.5727
M33422.m	rs12312580	A	T	0.8015	0.0089	0.0018	9.62E-07	24.4475
M33422.m	rs12500887	C	G	0.7842	-0.0089	0.0018	7.04E-07	24.4475
M33422.m	rs1503575	A	G	0.1815	0.0083	0.0018	6.18E-06	21.2623
M33422.m	rs1669715	T	C	0.0915	-0.0126	0.0027	2.3E-06	21.7778
M33422.m	rs16937239	A	G	0.9882	0.0405	0.0078	1.8E-07	26.9601
M33422.m	rs1764109	A	G	0.2241	0.0082	0.0018	7.2E-06	20.7531
M33422.m	rs1951768	A	G	0.6648	0.0078	0.0018	8.56E-06	18.7778
M33422.m	rs2241217	T	C	0.7557	0.0086	0.0018	2.73E-06	22.8272
M33422.m	rs2263065	T	G	0.4417	-0.0105	0.0018	3.56E-09	34.0278

M33422.m	rs2276228	A	C	0.2783	0.0081	0.0018	5.35E-06	20.25
M33422.m	rs2298381	C	G	0.2267	-0.0091	0.0018	5.23E-07	25.5586
M33422.m	rs2731506	A	G	0.2438	-0.0083	0.0018	3.78E-06	21.2623
M33422.m	rs2758886	A	G	0.2863	0.0087	0.0018	7.46E-07	23.3611
M33422.m	rs2817817	A	G	0.2809	-0.01	0.0018	2.14E-08	30.8642
M33422.m	rs2874279	A	G	0.7386	-0.0087	0.0018	1.02E-06	23.3611
M33422.m	rs3810252	A	G	0.8089	-0.0094	0.0019	5.21E-07	24.4765
M33422.m	rs40525	A	C	0.7741	0.0081	0.0018	5.94E-06	20.25
M33422.m	rs4396797	T	C	0.7483	0.0081	0.0018	5.39E-06	20.25
M33422.m	rs4524522	C	G	0.0858	-0.0121	0.0027	6.92E-06	20.0837
M33422.m	rs4870483	T	G	0.7193	0.0082	0.0018	3.85E-06	20.7531
M33422.m	rs550336	C	G	0.2034	-0.0081	0.0018	8.68E-06	20.25
M33422.m	rs5751928	A	G	0.4847	-0.0082	0.0017	2.31E-06	23.2664
M33422.m	rs6417039	A	G	0.1602	-0.0121	0.0025	1.11E-06	23.4256
M33422.m	rs7111143	A	G	0.2847	-0.0095	0.0018	5.35E-08	27.8549
M33422.m	rs738873	T	C	0.6892	0.0089	0.0018	5.02E-07	24.4475
M33422.m	rs7616518	A	G	0.4286	0.0081	0.0018	4.23E-06	20.25
M33422.m	rs7901640	A	G	0.3935	-0.0077	0.0017	9.06E-06	20.5156
M33422.m	rs8029902	T	C	0.299	0.0078	0.0018	9.48E-06	18.7778
M33422.m	rs855396	C	G	0.6896	-0.008	0.0018	5.57E-06	19.7531
M33422.m	rs9805690	C	G	0.986	-0.037	0.008	3.75E-06	21.3906
M33423.x	rs10074690	C	G	0.8839	0.5873	0.1215	1.33E-06	23.3651
M33423.x	rs10118205	C	G	0.0667	-0.7381	0.1517	1.14E-06	23.6733
M33423.x	rs10423270	T	G	0.9333	1.0018	0.1561	1.38E-10	41.1866
M33423.x	rs11228158	A	T	0.9654	0.61	0.0865	1.72E-12	49.731
M33423.x	rs11569035	T	C	0.05	-0.9284	0.1968	2.39E-06	22.2546
M33423.x	rs11633975	A	G	0.9833	1.5086	0.3324	5.67E-06	20.5981
M33423.x	rs11697457	A	G	0.974	1.2995	0.2699	1.47E-06	23.1818
M33423.x	rs11773099	C	G	0.9667	0.9721	0.1848	1.45E-07	27.6706
M33423.x	rs1182658	A	T	0.025	-1.2189	0.2756	9.77E-06	19.5604
M33423.x	rs12056370	A	G	0.0169	-1.492	0.3339	7.86E-06	19.9666
M33423.x	rs12417185	A	T	0.0796	-0.2877	0.0613	2.71E-06	22.0272
M33423.x	rs1244356	A	C	0.1	-0.618	0.1355	5.13E-06	20.8017
M33423.x	rs1462054	A	G	0.9746	1.266	0.2721	3.27E-06	21.6476
M33423.x	rs1515977	A	G	0.0594	-0.9112	0.1799	4.06E-07	25.6546
M33423.x	rs16834824	T	C	0.0179	-1.557	0.3291	2.23E-06	22.3832
M33423.x	rs16848159	A	G	0.0199	-1.7999	0.3054	3.79E-09	34.7343
M33423.x	rs16891501	A	G	0.0932	-0.6946	0.155	7.44E-06	20.082
M33423.x	rs16962580	T	C	0.0271	-1.2674	0.2697	2.62E-06	22.0834
M33423.x	rs17033607	A	G	0.0249	-1.5889	0.3178	5.75E-07	24.9969
M33423.x	rs17132995	T	G	0.0177	-1.8613	0.3006	5.95E-10	38.3402
M33423.x	rs17421736	A	G	0.0176	-1.5595	0.3448	6.09E-06	20.4567
M33423.x	rs17616308	A	T	0.0236	-1.4131	0.2951	1.68E-06	22.9302
M33423.x	rs1937053	A	T	0.9239	0.9018	0.1864	1.31E-06	23.4061
M33423.x	rs197623	A	C	0.0417	-0.982	0.2157	5.32E-06	20.7263
M33423.x	rs2028039	A	C	0.0177	-1.5512	0.33	2.58E-06	22.0957
M33423.x	rs2077726	T	G	0.7831	-0.1686	0.0359	2.71E-06	22.056
M33423.x	rs2111849	A	C	0.0175	-1.5433	0.3328	3.54E-06	21.5047
M33423.x	rs2287296	T	C	0.025	-1.2634	0.2721	3.44E-06	21.5588
M33423.x	rs2365913	T	C	0.9519	1.0329	0.2136	1.34E-06	23.3837
M33423.x	rs2494014	A	G	0.8251	-0.1788	0.0372	1.56E-06	23.102
M33423.x	rs2503005	T	C	0.7917	0.4363	0.0977	7.92E-06	19.9426
M33423.x	rs355924	A	G	0.0661	-0.7952	0.1752	5.7E-06	20.6008
M33423.x	rs4240294	T	C	0.0413	-0.3218	0.0686	2.73E-06	22.0051
M33423.x	rs4468280	A	G	0.6096	0.1441	0.0325	9.41E-06	19.659

M33423.x	rs4490621	T	C	0.9824	1.4958	0.3343	7.68E-06	20.0205
M33423.x	rs4516063	T	G	0.9154	0.8886	0.1617	3.92E-08	30.199
M33423.x	rs4527784	A	G	0.9569	1.0631	0.2119	5.25E-07	25.1702
M33423.x	rs4663518	C	G	0.0508	-1.0035	0.1905	1.39E-07	27.7488
M33423.x	rs4767452	T	C	0.0834	-0.7693	0.1668	3.99E-06	21.2716
M33423.x	rs4811103	T	C	0.3103	-0.4766	0.104	4.58E-06	21.0011
M33423.x	rs4838294	A	G	0.0167	-1.8595	0.2997	5.49E-10	38.4963
M33423.x	rs6818342	T	C	0.0593	-0.849	0.1515	2.12E-08	31.4044
M33423.x	rs6940545	A	C	0.9822	1.6279	0.3448	2.35E-06	22.2906
M33423.x	rs7123491	A	C	0.016	-1.7152	0.3637	2.4E-06	22.2404
M33423.x	rs7231643	A	G	0.0297	-0.5075	0.1149	9.97E-06	19.5089
M33423.x	rs7258950	A	G	0.0424	-0.9642	0.2174	9.15E-06	19.6705
M33423.x	rs7279663	T	G	0.9746	1.5318	0.3275	2.9E-06	21.8767
M33423.x	rs7510709	C	G	0.9829	1.7567	0.3275	8.13E-08	28.7722
M33423.x	rs7520433	C	G	0.0583	-0.8675	0.1889	4.38E-06	21.0899
M33423.x	rs7628540	T	G	0.1273	-0.6303	0.1251	4.67E-07	25.3852
M33423.x	rs7663974	A	T	0.2455	0.2125	0.048	9.42E-06	19.5991
M33423.x	rs7672448	A	G	0.0196	-1.6006	0.3587	8.12E-06	19.9114
M33423.x	rs8013127	A	G	0.0184	-1.5304	0.3325	4.16E-06	21.1849
M33423.x	rs8192827	A	G	0.9519	0.7137	0.1588	6.94E-06	20.199
M33423.x	rs986842	T	C	0.0167	-1.5302	0.3307	3.71E-06	21.4106
M33423.x	rs9892632	A	G	0.0169	-1.4923	0.3338	7.83E-06	19.9866
M33423.x	rs9921315	A	G	0.8729	0.7395	0.1281	7.82E-09	33.3256
M33423.x	rs9959639	T	C	0.0678	-0.784	0.1768	9.27E-06	19.6638
M33441.m	rs1146579	T	C	0.6674	-0.0196	0.0036	5.78E-08	29.642
M33441.m	rs1171614	T	C	0.219	-0.0252	0.0048	1.7E-07	27.5625
M33441.m	rs12202350	T	C	0.9102	0.0596	0.0069	5.07E-18	74.6095
M33441.m	rs12629505	A	G	0.3981	0.017	0.0036	2.21E-06	22.2994
M33441.m	rs13262318	A	G	0.9831	0.0668	0.0143	3.03E-06	21.8213
M33441.m	rs2404602	A	G	0.4398	0.0259	0.0036	3.56E-13	51.76
M33441.m	rs3134034	T	C	0.5354	0.031	0.0068	4.51E-06	20.7829
M33441.m	rs3798167	T	G	0.2061	0.0433	0.0044	2.2E-22	96.8435
M33441.m	rs4886484	C	G	0.6217	-0.024	0.0036	2.69E-11	44.4444
M33441.m	rs622591	T	C	0.2154	0.0215	0.0045	1.4E-06	22.8272
M33441.m	rs648253	A	G	0.5074	-0.0413	0.0036	6.02E-31	131.612
M33441.m	rs662138	C	G	0.832	0.0767	0.0052	4.98E-49	217.563
M33441.m	rs7820633	A	G	0.6205	-0.017	0.0036	2.94E-06	22.2994
M33441.m	rs9456496	A	G	0.8811	0.031	0.0055	1.36E-08	31.7686
M33441.m	rs9456506	A	G	0.9196	-0.0321	0.007	5.09E-06	21.0288
M33441.m	rs9457843	T	C	0.1219	0.0495	0.006	1.33E-16	68.0625
M33441.m	rs959046	C	G	0.4	0.0178	0.0037	1.12E-06	23.1439
M33442.m	rs1051331	A	G	0.8435	-0.0088	0.0018	6.47E-07	23.9012
M33442.m	rs10809858	A	G	0.8089	-0.0103	0.0022	4.16E-06	21.9194
M33442.m	rs11047762	T	C	0.0528	-0.0157	0.0033	1.49E-06	22.6345
M33442.m	rs11617124	A	C	0.8238	-0.0081	0.0018	3.78E-06	20.25
M33442.m	rs12041762	T	C	0.2706	-0.0075	0.0017	7.85E-06	19.4637
M33442.m	rs12197031	T	G	0.1805	0.0079	0.0018	7.23E-06	19.2623
M33442.m	rs1287262	C	G	0.1938	0.0083	0.0017	1.16E-06	23.8374
M33442.m	rs12906520	A	T	0.9485	0.0402	0.009	7.54E-06	19.9511
M33442.m	rs13157599	T	C	0.0447	0.0171	0.0035	1.17E-06	23.8702
M33442.m	rs17304639	A	T	0.8349	-0.0094	0.0018	8.64E-08	27.2716
M33442.m	rs17318174	A	G	0.1787	0.008	0.0017	4.2E-06	22.1453
M33442.m	rs2209196	T	G	0.1924	-0.0083	0.0017	2.21E-06	23.8374
M33442.m	rs2465657	A	G	0.1764	-0.0085	0.0017	9.9E-07	25
M33442.m	rs2520361	A	G	0.799	-0.009	0.002	6.8E-06	20.25

M33442.m	rs3853497	T	C	0.2686	-0.0088	0.0017	2.91E-07	26.7958
M33442.m	rs4129608	A	G	0.8509	0.0087	0.0018	7.16E-07	23.3611
M33442.m	rs4697477	T	C	0.8334	-0.0085	0.0018	2.19E-06	22.2994
M33442.m	rs4707716	T	C	0.913	-0.0116	0.0026	6.07E-06	19.9053
M33442.m	rs5749010	A	G	0.0828	-0.0127	0.0028	4.42E-06	20.5727
M33442.m	rs5761606	T	G	0.07	0.0118	0.0026	7.86E-06	20.5976
M33442.m	rs6434188	T	C	0.2171	0.0075	0.0017	8.96E-06	19.4637
M33442.m	rs6543592	A	G	0.9265	-0.0127	0.0026	8.84E-07	23.8595
M33442.m	rs7090877	A	C	0.0784	0.0132	0.0026	2.56E-07	25.7751
M33442.m	rs7939695	T	C	0.0489	-0.0155	0.0033	2.76E-06	22.0615
M33442.m	rs8026775	A	G	0.068	-0.0155	0.0032	1.16E-06	23.4619
M33442.m	rs9500927	A	G	0.1534	0.0081	0.0018	6.56E-06	20.25
M33442.m	rs9540840	T	C	0.8471	0.0083	0.0018	3.82E-06	21.2623
M33442.m	rs9633478	A	G	0.0763	-0.0129	0.0026	5.31E-07	24.6169
M33443.m	rs1037028	T	C	0.9308	0.028	0.0063	9.75E-06	19.7531
M33443.m	rs12432812	A	C	0.9717	0.045	0.01	7.17E-06	20.25
M33443.m	rs1371204	T	C	0.1619	-0.03	0.0067	0.000007	20.049
M33443.m	rs1882545	A	G	0.6567	0.0148	0.0033	9.36E-06	20.1139
M33443.m	rs2129376	A	G	0.7795	0.0164	0.0036	5.67E-06	20.7531
M33443.m	rs4076939	A	C	0.2178	0.0191	0.0042	5.49E-06	20.6808
M33443.m	rs4325575	T	C	0.7167	-0.0175	0.0037	2.75E-06	22.3703
M33443.m	rs6719403	A	G	0.0772	0.0253	0.0057	9.8E-06	19.7011
M33443.m	rs7690478	T	C	0.304	0.015	0.0033	6.43E-06	20.6612
M33443.m	rs7692407	T	C	0.9336	-0.0293	0.0064	5.36E-06	20.9592
M33447.m	rs10098688	A	C	0.3373	0.0278	0.0063	9.68E-06	19.4719
M33447.m	rs11728793	T	C	0.7844	-0.0215	0.0044	1.1E-06	23.8765
M33447.m	rs13019537	C	G	0.8416	0.0228	0.005	5.34E-06	20.7936
M33447.m	rs1558852	A	G	0.7952	0.0197	0.0043	4.48E-06	20.9892
M33447.m	rs1922578	A	G	0.9821	-0.0653	0.0142	4.34E-06	21.147
M33447.m	rs2916164	T	C	0.334	0.0154	0.0035	9.19E-06	19.36
M33447.m	rs4287543	A	G	0.8771	0.0232	0.0052	7.16E-06	19.9053
M33447.m	rs603424	A	G	0.1849	-0.0367	0.0048	1.31E-14	58.4588
M33447.m	rs677603	T	C	0.2538	0.022	0.0042	1.56E-07	27.4376
M33447.m	rs868028	C	G	0.3352	-0.0155	0.0035	8.08E-06	19.6122
M33447.m	rs9880033	T	C	0.9838	0.117	0.0262	7.74E-06	19.942
M33453.m	rs1043909	T	C	0.1912	-0.0244	0.0051	2.19E-06	22.8897
M33453.m	rs12725757	A	T	0.6521	-0.0198	0.0042	2.96E-06	22.2245
M33453.m	rs1296812	C	G	0.8004	0.0225	0.0051	9.12E-06	19.4637
M33453.m	rs17022573	C	G	0.1953	0.02	0.0045	7.48E-06	19.7531
M33453.m	rs17484315	C	G	0.0326	0.0618	0.0116	1.07E-07	28.3832
M33453.m	rs1786356	A	G	0.606	0.023	0.005	4.23E-06	21.16
M33453.m	rs1978273	A	G	0.4362	-0.0165	0.0036	3.51E-06	21.0069
M33453.m	rs2471644	A	G	0.6142	0.0177	0.0036	6.5E-07	24.1736
M33453.m	rs2649049	T	C	0.5773	-0.0164	0.0036	4.63E-06	20.7531
M33453.m	rs2733118	C	G	0.118	0.0271	0.006	5.49E-06	20.4003
M33453.m	rs2746997	A	G	0.4486	0.0161	0.0035	6E-06	21.16
M33453.m	rs4807122	T	C	0.386	0.018	0.004	6.8E-06	20.25
M33453.m	rs6869232	A	G	0.8574	-0.0235	0.0053	8.05E-06	19.66
M33453.m	rs7036192	A	T	0.9301	0.0835	0.0152	3.5E-08	30.1777
M33453.m	rs7075080	A	G	0.6326	-0.0171	0.0036	2.61E-06	22.5625
M33453.m	rs8102864	A	C	0.5284	0.0171	0.0037	4.56E-06	21.3594
M33453.m	rs9874488	A	G	0.293	0.0215	0.0048	8.68E-06	20.0629
M33477.m	rs10119850	A	G	0.5446	0.0088	0.0019	2.6E-06	21.4515
M33477.m	rs10189479	A	C	0.4262	-0.0081	0.0018	5.91E-06	20.25
M33477.m	rs10263766	T	C	0.2442	-0.0106	0.0018	7.47E-09	34.679

M33477.m	rs10765992	A	C	0.6859	0.0082	0.0018	5.13E-06	20.7531
M33477.m	rs10832688	A	G	0.547	-0.009	0.002	6.8E-06	20.25
M33477.m	rs1093661	T	G	0.675	0.009	0.002	6.8E-06	20.25
M33477.m	rs11054848	A	G	0.3623	-0.0086	0.0018	2.85E-06	22.8272
M33477.m	rs11065987	A	G	0.5821	-0.0088	0.0018	8.3E-07	23.9012
M33477.m	rs11160549	A	G	0.2939	-0.0088	0.0018	1.54E-06	23.9012
M33477.m	rs11241111	A	G	0.7231	0.0095	0.0018	2.15E-07	27.8549
M33477.m	rs12096170	A	G	0.4016	0.0082	0.0018	4.97E-06	20.7531
M33477.m	rs12481269	T	C	0.9208	-0.0158	0.0035	8.56E-06	20.3788
M33477.m	rs1260326	T	C	0.4042	0.0086	0.0018	1.36E-06	22.8272
M33477.m	rs12629138	T	G	0.063	0.019	0.0036	1.82E-07	27.8549
M33477.m	rs12749761	A	G	0.271	-0.0087	0.0019	5.49E-06	20.9668
M33477.m	rs13022722	A	G	0.3471	0.0094	0.0018	2.46E-07	27.2716
M33477.m	rs13385804	A	C	0.7023	-0.0081	0.0018	9.39E-06	20.25
M33477.m	rs191149	A	C	0.7214	0.009	0.0018	9.14E-07	25
M33477.m	rs201252	T	C	0.5963	0.0086	0.0018	1.61E-06	22.8272
M33477.m	rs2035058	A	G	0.8888	-0.0122	0.0028	9.98E-06	18.9847
M33477.m	rs2101874	T	C	0.7429	0.0089	0.0018	1.1E-06	24.4475
M33477.m	rs2391217	A	C	0.1069	0.0149	0.0027	4.88E-08	30.454
M33477.m	rs244523	A	T	0.2872	-0.02	0.0044	6.53E-06	20.6612
M33477.m	rs2487521	T	C	0.6858	0.0084	0.0018	3.54E-06	21.7778
M33477.m	rs270144	T	C	0.6901	-0.0082	0.0018	6.9E-06	20.7531
M33477.m	rs398462	T	C	0.5177	0.0087	0.0018	1.39E-06	23.3611
M33477.m	rs4141184	T	C	0.2907	-0.0081	0.0018	8.46E-06	20.25
M33477.m	rs4505759	T	C	0.3074	-0.0089	0.0019	2.36E-06	21.9418
M33477.m	rs4687367	T	C	0.4659	0.0083	0.0018	4.21E-06	21.2623
M33477.m	rs4687717	T	C	0.4248	0.0133	0.0018	1.07E-13	54.5957
M33477.m	rs4687718	A	G	0.1137	-0.0178	0.0033	8.81E-08	29.0946
M33477.m	rs476093	A	C	0.7566	-0.0084	0.0018	4.98E-06	21.7778
M33477.m	rs4853388	A	G	0.2394	-0.0092	0.0018	5.38E-07	26.1235
M33477.m	rs612149	A	G	0.1538	0.0129	0.0027	2.04E-06	22.8272
M33477.m	rs6135736	A	G	0.248	-0.0087	0.0019	2.78E-06	20.9668
M33477.m	rs6426492	T	C	0.3336	-0.0081	0.0018	8.75E-06	20.25
M33477.m	rs6434958	C	G	0.1118	-0.0123	0.0027	7.1E-06	20.7531
M33477.m	rs6860069	T	C	0.29	0.0081	0.0018	7.14E-06	20.25
M33477.m	rs6948214	A	T	0.2357	0.0089	0.0018	1.36E-06	24.4475
M33477.m	rs7002795	T	C	0.1074	-0.0125	0.0028	9.11E-06	19.9298
M33477.m	rs7316944	C	G	0.2987	-0.0082	0.0018	6.25E-06	20.7531
M33477.m	rs735080	A	G	0.6074	0.008	0.0018	8.8E-06	19.7531
M33477.m	rs741301	T	C	0.6849	0.009	0.0018	8.12E-07	25
M33477.m	rs7767693	A	G	0.2941	0.0085	0.0018	2.73E-06	22.2994
M33477.m	rs8182062	A	G	0.7075	-0.0083	0.0018	4.75E-06	21.2623
M33477.m	rs951062	T	C	0.703	0.0083	0.0018	5.68E-06	21.2623
M33477.m	rs9555127	A	G	0.3316	-0.0097	0.0018	7E-08	29.0401
M33477.m	rs9677367	A	G	0.6892	0.0082	0.0018	5.39E-06	20.7531
M33477.m	rs9888676	A	G	0.2454	0.0084	0.0018	5.44E-06	21.7778
M33477.m	rs9990565	T	C	0.1528	-0.0119	0.0026	5.48E-06	20.9482
M33488.m	rs10186575	A	G	0.1328	0.0288	0.0063	5.1E-06	20.898
M33488.m	rs11149935	A	G	0.1083	-0.0349	0.007	6.24E-07	24.8573
M33488.m	rs11687801	T	C	0.3033	-0.0217	0.0045	1.38E-06	23.2538
M33488.m	rs12999094	T	G	0.8061	0.0245	0.0054	5.92E-06	20.5847
M33488.m	rs17522290	A	G	0.932	-0.0393	0.0089	9.91E-06	19.4987
M33488.m	rs2197399	A	G	0.5961	-0.023	0.0045	2.68E-07	26.1235
M33488.m	rs2325470	C	G	0.7906	-0.0548	0.0124	9.14E-06	19.5307
M33488.m	rs2466085	A	G	0.0186	0.1762	0.0354	6.7E-07	24.7745

M33488.m	rs3020376	A	G	0.7234	0.0544	0.0113	1.33E-06	23.1761
M33488.m	rs3024981	A	G	0.0147	-0.0862	0.0192	7.14E-06	20.1564
M33488.m	rs328833	A	G	0.6759	0.0233	0.0045	2.72E-07	26.8094
M33488.m	rs4374940	T	C	0.0286	0.1321	0.0292	5.89E-06	20.4663
M33488.m	rs555096	T	C	0.039	-0.0543	0.0117	3.86E-06	21.5391
M33488.m	rs6862546	T	C	0.1752	-0.0281	0.006	3.31E-06	21.9336
M33488.m	rs7227090	A	G	0.8661	0.0297	0.0063	2.28E-06	22.2245
M33488.m	rs730596	A	G	0.9335	0.0401	0.0089	6.43E-06	20.3006
M33488.m	rs745439	T	C	0.1388	-0.0462	0.0104	9.78E-06	19.7341
M33488.m	rs7942396	T	C	0.0664	-0.048	0.0089	7.68E-08	29.0872
M33507.m	rs10210072	T	G	0.9583	-0.0617	0.014	9.93E-06	19.4229
M33507.m	rs1061134	A	G	0.0946	-0.1458	0.0084	2.82E-67	301.27
M33507.m	rs1061135	A	G	0.4604	0.0689	0.005	2.33E-43	189.888
M33507.m	rs10748725	A	G	0.3536	-0.0505	0.0052	2.11E-22	94.314
M33507.m	rs10788312	T	C	0.3057	-0.0228	0.0051	8.32E-06	19.9862
M33507.m	rs10883057	C	G	0.2703	-0.0409	0.0054	3.25E-14	57.3666
M33507.m	rs11189692	T	C	0.7797	-0.0427	0.0061	2.26E-12	49
M33507.m	rs11189833	A	G	0.179	0.0274	0.0061	6.03E-06	20.1763
M33507.m	rs11595763	A	C	0.8295	-0.0863	0.0061	8.34E-46	200.153
M33507.m	rs11778205	A	G	0.6564	-0.0227	0.0051	8.92E-06	19.8112
M33507.m	rs11816373	T	C	0.0299	0.1273	0.0265	1.63E-06	23.0762
M33507.m	rs12415538	T	C	0.0188	-0.1204	0.0194	5.76E-10	38.5167
M33507.m	rs12780272	T	C	0.0376	0.08	0.0134	2.55E-09	35.6427
M33507.m	rs12814926	A	G	0.1985	0.0275	0.006	5.33E-06	21.0069
M33507.m	rs13410232	A	T	0.781	0.0291	0.0059	9.48E-07	24.3266
M33507.m	rs1365	A	C	0.9458	0.0752	0.0101	1.16E-13	55.4361
M33507.m	rs1372691	A	G	0.289	0.0458	0.0102	6.81E-06	20.1619
M33507.m	rs17109581	A	T	0.9614	0.274	0.0166	1.61E-61	272.449
M33507.m	rs17279437	A	G	0.0953	0.0458	0.0084	5.66E-08	29.7285
M33507.m	rs17453066	T	C	0.8327	-0.0368	0.007	1.17E-07	27.6376
M33507.m	rs17470570	A	G	0.9497	0.0538	0.0113	2E-06	22.6677
M33507.m	rs1889459	C	G	0.1628	-0.0412	0.0067	9.94E-10	37.8133
M33507.m	rs2118530	T	C	0.1842	0.0279	0.0059	2.64E-06	22.3617
M33507.m	rs2254969	A	G	0.5456	-0.0222	0.005	9.77E-06	19.7136
M33507.m	rs4238523	A	G	0.544	-0.0222	0.005	9.48E-06	19.7136
M33507.m	rs4303969	T	C	0.8003	-0.0289	0.0065	8.32E-06	19.7683
M33507.m	rs4917817	A	C	0.6783	0.0406	0.005	6.71E-16	65.9344
M33507.m	rs4919141	A	G	0.7786	0.0282	0.0059	1.73E-06	22.8452
M33507.m	rs4919221	T	C	0.7435	-0.0685	0.0052	2.7E-40	173.53
M33507.m	rs542386	A	C	0.0441	-0.0671	0.0123	4.56E-08	29.7601
M33507.m	rs6532828	A	G	0.7966	0.0319	0.006	9.77E-08	28.2669
M33507.m	rs6559527	A	G	0.8306	-0.0302	0.0066	5.01E-06	20.9376
M33507.m	rs7096654	T	C	0.6211	0.2537	0.0047	1E-200	2913.7
M33507.m	rs7302669	A	G	0.3296	-0.0232	0.0051	6.49E-06	20.6936
M33507.m	rs754586	T	C	0.4128	-0.0341	0.005	1.35E-11	46.5124
M33507.m	rs8101881	T	C	0.6089	0.0281	0.005	2.57E-08	31.5844
M33507.m	rs942800	A	C	0.4265	0.0362	0.0053	1.06E-11	46.6515
M33507.m	rs942812	A	C	0.0293	-0.1771	0.0141	4.24E-36	157.761
M33507.m	rs9488349	A	C	0.9829	0.0861	0.0188	4.56E-06	20.9745
M33507.m	rs9952576	T	G	0.3194	-0.0228	0.0051	8.4E-06	19.9862
M33508.m	rs10469966	A	G	0.2526	0.1024	0.0068	1.12E-51	226.768
M33508.m	rs10492598	A	C	0.0227	0.1605	0.0352	5.08E-06	20.7905
M33508.m	rs11676273	A	G	0.2042	-0.0348	0.0075	3.86E-06	21.5296
M33508.m	rs12295872	A	G	0.018	0.2913	0.0539	6.67E-08	29.2081
M33508.m	rs1280976	T	C	0.9163	-0.0523	0.0115	5.7E-06	20.6827

M33508.m	rs1450233	A	T	0.3174	-0.0584	0.0116	4.32E-07	25.346
M33508.m	rs1551345	T	C	0.924	-0.0527	0.0118	7.38E-06	19.9461
M33508.m	rs17109581	A	T	0.9697	0.1313	0.0259	4.12E-07	25.6998
M33508.m	rs1802376	A	G	0.0427	0.0875	0.0193	5.95E-06	20.5542
M33508.m	rs1864490	T	G	0.7198	-0.0324	0.0068	1.66E-06	22.7024
M33508.m	rs2070581	A	G	0.6852	-0.0399	0.0067	3.18E-09	35.4647
M33508.m	rs42789	A	G	0.426	-0.0278	0.0062	7.33E-06	20.1051
M33508.m	rs4488133	A	T	0.7014	0.0985	0.0067	1.26E-48	216.134
M33508.m	rs4899659	C	G	0.3679	-0.0311	0.0066	2.42E-06	22.2041
M33508.m	rs4919209	T	G	0.2024	-0.0463	0.0081	1.11E-08	32.6732
M33508.m	rs4919220	A	G	0.731	-0.0328	0.0067	8.82E-07	23.9661
M33508.m	rs6023015	C	G	0.4176	-0.0267	0.006	8.67E-06	19.8025
M33508.m	rs6781351	A	C	0.8686	-0.0792	0.0163	1.14E-06	23.6089
M33508.m	rs7073497	T	C	0.0267	-0.115	0.0228	4.33E-07	25.4405
M33508.m	rs7600291	C	G	0.908	-0.0661	0.0094	2.59E-12	49.4478
M33509.m	rs12953402	T	C	0.1905	0.022	0.0044	7.33E-07	25
M33509.m	rs12991373	A	G	0.4195	0.0154	0.0035	9.13E-06	19.36
M33509.m	rs16939910	A	T	0.2762	-0.0333	0.007	1.92E-06	22.6304
M33509.m	rs17421232	C	G	0.2887	-0.0191	0.0042	6.18E-06	20.6808
M33509.m	rs281796	A	C	0.8247	-0.0356	0.0078	5.62E-06	20.831
M33509.m	rs537721	T	C	0.9625	-0.0417	0.009	3.89E-06	21.4678
M33509.m	rs6430553	T	C	0.4007	-0.0204	0.0035	4.81E-09	33.9722
M33509.m	rs7600589	C	G	0.7048	0.0164	0.0036	5.59E-06	20.7531
M33509.m	rs9648853	T	C	0.6009	-0.0161	0.0035	3.67E-06	21.16
M33510.m	rs10928510	A	G	0.8526	0.0187	0.0041	5.13E-06	20.8025
M33510.m	rs11230225	T	C	0.8811	0.0366	0.0082	8.16E-06	19.9221
M33510.m	rs12429935	T	C	0.9468	-0.0586	0.0129	5.31E-06	20.6355
M33510.m	rs12795652	A	T	0.8826	-0.0203	0.0044	3.34E-06	21.2856
M33510.m	rs12953402	T	C	0.1893	0.0165	0.0035	2.29E-06	22.2245
M33510.m	rs13021657	T	C	0.0179	-0.0519	0.0117	9.43E-06	19.6772
M33510.m	rs1421106	T	G	0.9228	-0.0236	0.0049	1.79E-06	23.197
M33510.m	rs1674726	A	G	0.3501	-0.0137	0.0028	8.82E-07	23.9401
M33510.m	rs16830291	T	G	0.3419	-0.0121	0.0027	5.55E-06	20.0837
M33510.m	rs16939910	A	T	0.2749	-0.0292	0.0062	2.52E-06	22.1811
M33510.m	rs17754631	A	G	0.7461	-0.0161	0.0034	2.25E-06	22.423
M33510.m	rs2328991	C	G	0.1132	0.0204	0.0043	1.88E-06	22.5073
M33510.m	rs248670	T	C	0.6181	0.0122	0.0027	4.78E-06	20.417
M33510.m	rs303031	A	G	0.5388	-0.0125	0.0026	1.72E-06	23.1139
M33510.m	rs3783853	A	G	0.4188	0.0124	0.0027	3.27E-06	21.0919
M33510.m	rs4073619	T	G	0.5405	-0.0119	0.0026	6.83E-06	20.9482
M33510.m	rs4878060	T	G	0.6539	-0.0123	0.0027	6.17E-06	20.7531
M33510.m	rs6055874	T	C	0.4882	0.0125	0.0026	2.15E-06	23.1139
M33510.m	rs6430553	T	C	0.393	-0.0181	0.0026	6.29E-12	48.463
M33510.m	rs673260	A	G	0.1835	-0.0174	0.0034	4.21E-07	26.1903
M33510.m	rs7133996	T	C	0.2736	-0.0151	0.0033	5.83E-06	20.9376
M33510.m	rs7327971	A	G	0.3546	-0.0125	0.0026	2.25E-06	23.1139
M33510.m	rs733017	T	C	0.6092	-0.0118	0.0026	6.94E-06	20.5976
M33510.m	rs935270	A	G	0.4556	-0.0135	0.0027	4.66E-07	25
M33510.m	rs9451877	A	G	0.9703	-0.0943	0.0198	1.92E-06	22.6826
M33510.m	rs9825012	T	C	0.9575	0.0434	0.0097	7.7E-06	20.0187
M33515.m	rs10016594	A	C	0.5815	-0.0085	0.0018	3.12E-06	22.2994
M33515.m	rs10087179	T	C	0.6135	-0.0092	0.0018	5.13E-07	26.1235
M33515.m	rs1039386	A	G	0.5733	-0.0088	0.0018	1.36E-06	23.9012
M33515.m	rs10868025	A	G	0.6116	0.0088	0.0018	1.52E-06	23.9012
M33515.m	rs10976882	A	G	0.6202	0.0086	0.0018	2.79E-06	22.8272

M33515.m	rs11022904	A	G	0.154	-0.014	0.0027	1.86E-07	26.8861
M33515.m	rs1110277	A	G	0.675	-0.0098	0.0019	1.47E-07	26.6039
M33515.m	rs11660401	T	C	0.604	0.0095	0.0018	2.21E-07	27.8549
M33515.m	rs11806477	T	C	0.3749	0.0083	0.0018	6.1E-06	21.2623
M33515.m	rs1218924	A	G	0.6201	0.0094	0.0019	3.39E-07	24.4765
M33515.m	rs12231049	A	G	0.922	0.0164	0.0036	4.6E-06	20.7531
M33515.m	rs12287384	T	C	0.0473	-0.0231	0.0052	8.39E-06	19.7341
M33515.m	rs1373219	T	C	0.5411	0.0082	0.0018	8.22E-06	20.7531
M33515.m	rs1924466	A	C	0.4544	0.0082	0.0018	8.17E-06	20.7531
M33515.m	rs2067434	T	C	0.4351	-0.0083	0.0018	5.24E-06	21.2623
M33515.m	rs2160860	A	T	0.6141	0.012	0.0018	7.35E-11	44.4444
M33515.m	rs2278908	T	C	0.359	0.0087	0.0018	2.55E-06	23.3611
M33515.m	rs2280821	T	G	0.508	0.0091	0.0018	6.54E-07	25.5586
M33515.m	rs2329083	A	T	0.6344	-0.0096	0.0018	1.9E-07	28.4444
M33515.m	rs2494461	A	C	0.6292	0.0083	0.0018	6.27E-06	21.2623
M33515.m	rs2555110	A	G	0.861	-0.0122	0.0028	9.51E-06	18.9847
M33515.m	rs2580344	T	C	0.6243	0.0083	0.0018	6E-06	21.2623
M33515.m	rs2715313	A	G	0.3788	-0.0084	0.0018	4.6E-06	21.7778
M33515.m	rs402710	T	C	0.3378	0.0086	0.0019	4.16E-06	20.4875
M33515.m	rs403018	A	G	0.6712	-0.0084	0.0018	5.36E-06	21.7778
M33515.m	rs4509324	A	G	0.9671	-0.0277	0.006	3.27E-06	21.3136
M33515.m	rs4626675	T	C	0.466	0.01	0.002	5.73E-07	25
M33515.m	rs4634107	T	G	0.5163	-0.0093	0.0018	3.25E-07	26.6944
M33515.m	rs4669966	A	G	0.5646	0.0087	0.0018	1.86E-06	23.3611
M33515.m	rs4766046	A	C	0.5752	-0.0086	0.0018	2.69E-06	22.8272
M33515.m	rs4823474	T	C	0.5551	0.009	0.0018	8.28E-07	25
M33515.m	rs4843718	A	G	0.2413	0.0158	0.0026	1.71E-09	36.929
M33515.m	rs4890333	A	G	0.5104	-0.0086	0.0018	2.06E-06	22.8272
M33515.m	rs4902201	A	G	0.3616	0.0095	0.0018	2.21E-07	27.8549
M33515.m	rs4980965	T	C	0.5402	-0.0089	0.0018	1.28E-06	24.4475
M33515.m	rs6536380	T	C	0.6346	0.0093	0.0018	4.49E-07	26.6944
M33515.m	rs6694467	A	G	0.8153	0.0121	0.0026	4.44E-06	21.6583
M33515.m	rs6764891	A	G	0.3475	0.0086	0.0018	2.89E-06	22.8272
M33515.m	rs7684565	T	C	0.4972	0.0081	0.0018	8.67E-06	20.25
M33515.m	rs7922903	T	G	0.0945	-0.0168	0.0035	2.1E-06	23.04
M33515.m	rs792369	A	C	0.659	-0.0083	0.0018	6.65E-06	21.2623
M33515.m	rs8045299	A	G	0.3403	0.0089	0.0019	1.81E-06	21.9418
M33515.m	rs9827101	A	C	0.5367	0.0084	0.0018	4.9E-06	21.7778
M33515.m	rs9972361	A	G	0.3351	-0.0087	0.0018	2.32E-06	23.3611
M33531.m	rs10247235	T	G	0.319	-0.0272	0.0062	9.41E-06	19.2466
M33531.m	rs10966901	A	G	0.6275	0.0273	0.0061	8.07E-06	20.0293
M33531.m	rs11623436	T	C	0.4589	-0.0296	0.0062	1.48E-06	22.7929
M33531.m	rs12368489	T	C	0.135	0.0376	0.0081	3.21E-06	21.5479
M33531.m	rs12641265	C	G	0.9569	-0.0635	0.0143	8.81E-06	19.7186
M33531.m	rs17081093	A	G	0.8144	0.0357	0.0078	4.93E-06	20.9482
M33531.m	rs6129197	A	G	0.8768	0.0418	0.009	3.79E-06	21.5709
M33531.m	rs6849330	T	G	0.0204	-0.0937	0.0208	6.96E-06	20.2933
M33531.m	rs7338260	T	C	0.0857	0.0515	0.0114	5.94E-06	20.4082
M33531.m	rs7855332	T	G	0.5306	0.0563	0.0127	8.71E-06	19.6521
M33587.m	rs11133892	A	G	0.4421	0.0184	0.0039	2.13E-06	22.259
M33587.m	rs1115214	A	G	0.961	0.0451	0.0102	9.61E-06	19.5503
M33587.m	rs12336893	T	G	0.9592	0.0425	0.0095	7.11E-06	20.0139
M33587.m	rs13417885	A	G	0.9333	0.034	0.0073	3.41E-06	21.6926
M33587.m	rs1348656	T	C	0.7979	-0.0246	0.0047	1.99E-07	27.3952
M33587.m	rs2017823	A	G	0.8406	0.022	0.0049	6.37E-06	20.1583

M33587.m	rs2527641	A	G	0.5656	-0.018	0.0039	4.94E-06	21.3018
M33587.m	rs35350	A	G	0.9697	0.0664	0.0137	1.23E-06	23.4906
M33587.m	rs6465164	A	G	0.1492	-0.0394	0.0086	5.06E-06	20.9892
M33587.m	rs7260113	T	C	0.4442	-0.0173	0.0039	9.49E-06	19.6772
M33587.m	rs7314742	T	G	0.2658	-0.0187	0.0041	4.53E-06	20.8025
M33587.m	rs9906465	A	G	0.9319	-0.0364	0.0074	8.97E-07	24.1958
M33609.m	rs11830144	T	G	0.0928	0.088	0.0185	2E-06	22.6267
M33609.m	rs12335217	T	C	0.013	0.323	0.057	1.46E-08	32.1111
M33609.m	rs12633114	A	G	0.6036	-0.0693	0.0156	9.22E-06	19.7341
M33609.m	rs12769591	A	G	0.969	-0.194	0.043	6.43E-06	20.3548
M33609.m	rs1391470	T	C	0.0161	0.2838	0.0602	2.45E-06	22.2245
M33609.m	rs1530045	A	G	0.2868	-0.0557	0.0126	9.62E-06	19.542
M33609.m	rs17065159	A	G	0.0388	0.1289	0.0275	2.81E-06	21.9705
M33609.m	rs17124948	T	C	0.011	0.31	0.07	9.49E-06	19.6122
M33609.m	rs17433244	A	C	0.023	0.328	0.065	4.51E-07	25.4637
M33609.m	rs17665024	A	G	0.7991	-0.0628	0.014	6.87E-06	20.1216
M33609.m	rs1899124	T	C	0.051	0.304	0.055	3.25E-08	30.5507
M33609.m	rs2622243	A	G	0.4311	0.0538	0.0121	9.6E-06	19.7694
M33609.m	rs2801848	T	C	0.1289	0.0764	0.0155	8.58E-07	24.2954
M33609.m	rs4687396	A	C	0.9791	-0.1545	0.0342	6.08E-06	20.4082
M33609.m	rs542663	T	C	0.8073	-0.0674	0.0127	1.12E-07	28.1652
M33609.m	rs548027	A	G	0.0208	0.2591	0.0512	4.17E-07	25.6091
M33609.m	rs6978556	T	C	0.0175	0.1712	0.0381	7.08E-06	20.191
M33609.m	rs7239639	T	C	0.938	-0.254	0.05	3.77E-07	25.8064
M33609.m	rs756247	A	T	0.9396	-0.1619	0.0348	3.24E-06	21.6439
M33609.m	rs813108	A	G	0.0115	0.4574	0.1026	8.32E-06	19.8746
M33610.m	rs10425480	A	G	0.0157	0.5394	0.0954	1.59E-08	31.9687
M33610.m	rs10836708	C	G	0.326	-0.1272	0.028	5.53E-06	20.6376
M33610.m	rs11747400	A	T	0.0366	0.2269	0.0461	8.78E-07	24.2252
M33610.m	rs11938671	T	G	0.0205	0.2083	0.0458	5.38E-06	20.6846
M33610.m	rs12498013	T	C	0.0395	0.3027	0.0618	9.79E-07	23.991
M33610.m	rs1408509	A	G	0.3568	0.0526	0.011	1.78E-06	22.8658
M33610.m	rs1572704	A	G	0.0272	0.3904	0.0764	3.29E-07	26.1116
M33610.m	rs1596834	A	C	0.1181	0.1944	0.0435	7.87E-06	19.9717
M33610.m	rs16829299	T	G	0.0196	0.3861	0.087	9.12E-06	19.6952
M33610.m	rs17594255	A	T	0.1399	-0.0742	0.0156	2.1E-06	22.6234
M33610.m	rs2803893	A	G	0.7876	-0.0621	0.0138	7.09E-06	20.25
M33610.m	rs321188	A	G	0.9806	-0.536	0.1117	1.59E-06	23.0263
M33610.m	rs357118	A	G	0.8019	0.1438	0.0308	3.03E-06	21.798
M33610.m	rs4291236	C	G	0.0549	0.2588	0.057	5.54E-06	20.6148
M33610.m	rs4458182	A	T	0.0854	-0.0862	0.0189	5.3E-06	20.8013
M33610.m	rs5022652	A	G	0.1582	0.0649	0.014	3.84E-06	21.4898
M33610.m	rs601208	T	C	0.6648	0.0518	0.0111	3.04E-06	21.7778
M33610.m	rs6679176	T	C	0.9608	-0.3414	0.0696	9.2E-07	24.0607
M33610.m	rs6803591	T	C	0.0253	0.3527	0.0781	6.25E-06	20.3943
M33610.m	rs7105058	A	G	0.4597	0.0532	0.0115	3.36E-06	21.4007
M33610.m	rs7147680	A	G	0.0114	0.5941	0.1197	6.94E-07	24.6338
M33610.m	rs7185611	T	C	0.98	-0.4842	0.0987	9.41E-07	24.0666
M33610.m	rs7222177	T	C	0.0108	0.5557	0.122	5.2E-06	20.7473
M33610.m	rs7870108	T	G	0.0102	0.6206	0.118	1.46E-07	27.6605
M33610.m	rs8003714	T	G	0.017	0.4616	0.0995	3.46E-06	21.5221
M33610.m	rs8036131	T	G	0.7378	0.0608	0.013	3.15E-06	21.8736
M33610.m	rs8082167	T	C	0.0288	0.4457	0.0774	8.49E-09	33.1591
M33610.m	rs913080	T	C	0.37	0.1166	0.0245	1.97E-06	22.6498
M33610.m	rs9833839	T	C	0.0127	0.2858	0.062	3.96E-06	21.2491

M33627.m	rs12278387	T	C	0.7351	-0.0183	0.0041	6.54E-06	19.9221
M33627.m	rs12521058	A	C	0.402	-0.0197	0.0041	1.5E-06	23.0869
M33627.m	rs12763437	A	G	0.0834	0.0273	0.0061	8.68E-06	20.0293
M33627.m	rs1460191	A	C	0.1654	0.0207	0.0046	5.38E-06	20.25
M33627.m	rs1852749	A	T	0.942	-0.0352	0.0077	4.8E-06	20.898
M33627.m	rs3923017	T	C	0.0439	-0.0469	0.0103	5.31E-06	20.7334
M33627.m	rs4696727	T	C	0.649	0.0167	0.0036	2.88E-06	21.5193
M33627.m	rs562210	A	C	0.6864	0.0169	0.0037	4.72E-06	20.8627
M33627.m	rs6432256	T	C	0.7655	-0.0228	0.0051	7.48E-06	19.9862
M33627.m	rs9521789	T	C	0.6021	0.0175	0.0037	2.29E-06	22.3703
M33633.m	rs11934821	A	G	0.8122	0.0354	0.0079	7.8E-06	20.0795
M33633.m	rs12071172	A	G	0.0265	0.0934	0.0206	5.58E-06	20.557
M33633.m	rs1246172	A	G	0.2836	0.0328	0.007	2.52E-06	21.9559
M33633.m	rs13376835	A	G	0.8266	-0.0401	0.0082	1.13E-06	23.9145
M33633.m	rs1434384	A	G	0.5567	-0.0305	0.0062	7.15E-07	24.2001
M33633.m	rs17090140	A	G	0.0502	0.0636	0.0142	7.07E-06	20.0603
M33633.m	rs2283530	A	G	0.0497	0.1788	0.0401	8.26E-06	19.8814
M33633.m	rs2933259	T	C	0.5499	-0.0278	0.0062	6.53E-06	20.1051
M33633.m	rs6599251	T	G	0.553	-0.028	0.0062	5.41E-06	20.3954
M33633.m	rs6917152	A	C	0.0265	0.0893	0.0195	4.92E-06	20.9717
M33633.m	rs7231703	A	T	0.3913	0.035	0.0069	3.67E-07	25.7299
M33637.m	rs10186642	A	G	0.0229	-0.1988	0.0436	5.12E-06	20.7903
M33637.m	rs10228942	A	T	0.8711	0.0711	0.0159	7.75E-06	19.9961
M33637.m	rs10794793	T	C	0.3454	-0.0202	0.0045	6.82E-06	20.1501
M33637.m	rs12723593	C	G	0.7407	0.0234	0.0053	8.46E-06	19.4931
M33637.m	rs1993342	A	C	0.2758	-0.0533	0.0113	2.45E-06	22.2483
M33637.m	rs2171596	A	C	0.6593	-0.0204	0.0045	5.38E-06	20.5511
M33637.m	rs262749	A	C	0.6982	0.0208	0.0046	5.33E-06	20.4461
M33637.m	rs719203	T	C	0.3283	-0.0201	0.0045	9.27E-06	19.9511
M33637.m	rs8096862	T	C	0.2292	-0.0528	0.0119	9.85E-06	19.6867
M33637.m	rs830133	A	G	0.0673	0.0885	0.0198	7.51E-06	19.9782
M33638.m	rs10166510	A	G	0.6837	-0.0211	0.0044	2.06E-06	22.9964
M33638.m	rs10861380	A	G	0.7004	-0.0214	0.0045	1.59E-06	22.6153
M33638.m	rs10977422	A	G	0.8653	-0.0271	0.0061	9.63E-06	19.7369
M33638.m	rs11154157	T	C	0.676	0.0201	0.0045	6.23E-06	19.9511
M33638.m	rs1165189	A	C	0.7496	-0.0254	0.0053	1.46E-06	22.9676
M33638.m	rs1383950	T	C	0.9813	0.1548	0.0332	3.13E-06	21.7403
M33638.m	rs2776982	A	C	0.053	0.0447	0.0097	4.52E-06	21.2359
M33638.m	rs3024298	A	C	0.9784	0.0729	0.0159	4.32E-06	21.0214
M33638.m	rs902092	A	G	0.1236	-0.0642	0.0145	9.42E-06	19.6035
M33638.m	rs9375094	T	C	0.0286	-0.0678	0.014	1.28E-06	23.4533
M33652.m	rs10142427	A	C	0.6825	-0.0328	0.0062	1.33E-07	27.9875
M33652.m	rs10783282	A	G	0.2235	0.032	0.0071	5.86E-06	20.3134
M33652.m	rs11148029	A	G	0.8748	-0.0405	0.0086	2.74E-06	22.1775
M33652.m	rs11163350	T	C	0.3153	0.0272	0.0061	8.7E-06	19.8828
M33652.m	rs1374486	A	G	0.2098	-0.0319	0.007	5.52E-06	20.7676
M33652.m	rs16928165	A	G	0.974	-0.0885	0.0197	7.22E-06	20.1815
M33652.m	rs4915719	A	G	0.5398	-0.027	0.0061	8.19E-06	19.5915
M33652.m	rs8050423	A	G	0.0297	0.1772	0.0391	5.72E-06	20.5387
M33652.m	rs9962038	T	C	0.2175	0.0316	0.007	7.15E-06	20.3788
M33653.m	rs10168416	C	G	0.6733	-0.0267	0.0057	2.42E-06	21.9418
M33653.m	rs10768365	A	G	0.9629	0.1071	0.0241	9.04E-06	19.749
M33653.m	rs10881815	T	C	0.5581	-0.0267	0.0058	3.54E-06	21.1917
M33653.m	rs11062646	T	G	0.0564	-0.1164	0.026	7.36E-06	20.0428
M33653.m	rs12404165	A	G	0.1106	0.0396	0.0086	4.16E-06	21.2028

M33653.m	rs12692453	A	G	0.2156	0.0287	0.0065	9.31E-06	19.4956
M33653.m	rs17151367	C	G	0.1884	-0.0343	0.0073	2.83E-06	22.0771
M33653.m	rs174773	C	G	0.9424	-0.0832	0.0187	8.22E-06	19.7954
M33653.m	rs2540742	A	G	0.642	-0.0232	0.0051	5.55E-06	20.6936
M33653.m	rs4680	A	G	0.508	0.0263	0.0051	2.28E-07	26.5932
M33653.m	rs7173557	A	G	0.8224	-0.031	0.0066	3.17E-06	22.0615
M33653.m	rs7427160	T	C	0.5044	-0.0223	0.005	9.63E-06	19.8916
M33653.m	rs961787	T	C	0.3948	0.0254	0.0051	5.14E-07	24.8043
M33658.m	rs10074013	A	G	0.2515	0.0424	0.0085	5.48E-07	24.8825
M33658.m	rs10779577	A	G	0.209	0.0427	0.0093	4.07E-06	21.0809
M33658.m	rs11046265	T	C	0.2995	-0.0431	0.0092	2.53E-06	21.9472
M33658.m	rs13146757	T	C	0.0199	0.1467	0.03	1.02E-06	23.9121
M33658.m	rs17358216	T	C	0.0313	0.1099	0.0233	2.36E-06	22.2476
M33658.m	rs1892513	A	G	0.5107	-0.0346	0.0074	3.18E-06	21.8619
M33658.m	rs4437239	T	C	0.7829	-0.0378	0.0085	9.78E-06	19.7763
M33658.m	rs6502323	A	T	0.2996	0.0434	0.0083	1.56E-07	27.3416
M33658.m	rs7956425	A	G	0.8522	-0.093	0.0191	1.21E-06	23.7082
M33666.m	rs1035688	A	C	0.653	0.0107	0.0024	9.4E-06	19.8767
M33666.m	rs11725128	A	C	0.0325	-0.0306	0.0069	9.22E-06	19.6673
M33666.m	rs11825174	T	C	0.0223	-0.0584	0.013	6.84E-06	20.1808
M33666.m	rs13111443	T	C	0.7906	0.0124	0.0025	1.04E-06	24.6016
M33666.m	rs1443751	T	C	0.7823	0.0114	0.0025	5.36E-06	20.7936
M33666.m	rs1568846	A	G	0.5288	-0.0115	0.0024	1.74E-06	22.9601
M33666.m	rs16965354	A	G	0.035	-0.0304	0.0065	2.78E-06	21.8736
M33666.m	rs17130035	T	C	0.9127	-0.018	0.004	7.79E-06	20.25
M33666.m	rs17423920	T	C	0.91	0.0203	0.0041	5.47E-07	24.5146
M33666.m	rs2218152	T	G	0.4105	-0.0112	0.0025	5.78E-06	20.0704
M33666.m	rs2484984	A	C	0.3857	0.0191	0.0031	4.84E-10	37.9615
M33666.m	rs2567470	T	C	0.1528	-0.0248	0.0053	2.72E-06	21.8953
M33666.m	rs282115	A	G	0.0711	0.0237	0.0041	9.63E-09	33.414
M33666.m	rs2980782	C	G	0.3307	-0.013	0.0024	6.71E-08	29.3403
M33666.m	rs3124018	A	G	0.9208	-0.0196	0.0041	1.78E-06	22.8531
M33666.m	rs4148421	T	C	0.446	0.0153	0.0024	1.42E-10	40.6406
M33666.m	rs4707530	T	C	0.2328	0.0112	0.0025	7.69E-06	20.0704
M33666.m	rs6047134	A	G	0.2672	-0.0131	0.0025	8.93E-08	27.4576
M33666.m	rs6060270	T	G	0.2057	0.0134	0.0027	8.58E-07	24.631
M33666.m	rs6550017	T	C	0.0823	-0.0179	0.004	6.84E-06	20.0256
M33666.m	rs6804368	A	G	0.5714	0.0221	0.0024	8.17E-21	84.7934
M33666.m	rs7009544	T	C	0.7345	-0.011	0.0025	9.06E-06	19.36
M33666.m	rs7082340	A	G	0.5881	0.0105	0.0024	9.95E-06	19.1406
M33666.m	rs7429997	T	C	0.385	-0.011	0.0024	4.22E-06	21.0069
M33666.m	rs7775554	A	C	0.5279	0.019	0.0024	1.35E-15	62.6736
M33666.m	rs7843729	C	G	0.8642	-0.0142	0.0032	9.58E-06	19.6914
M33666.m	rs8002180	T	C	0.7203	-0.0239	0.0024	8.23E-23	99.1684
M33666.m	rs842456	A	G	0.7803	-0.0217	0.0046	1.76E-06	22.2538
M33666.m	rs9561817	T	C	0.0685	0.0243	0.0046	1.34E-07	27.906
M33675.m	rs10060182	A	G	0.3009	-0.0274	0.0062	9.45E-06	19.5307
M33675.m	rs11681355	A	G	0.318	0.0275	0.0061	7.56E-06	20.3238
M33675.m	rs12503398	A	G	0.7238	-0.0307	0.0069	8.68E-06	19.7961
M33675.m	rs1428261	T	C	0.6371	0.0285	0.0061	3.25E-06	21.8288
M33675.m	rs2957661	T	C	0.3698	0.0308	0.0061	4.34E-07	25.4942
M33675.m	rs4573623	T	C	0.5476	-0.0535	0.0117	4.69E-06	20.9091
M33675.m	rs6003815	T	C	0.4411	0.0243	0.0054	6.58E-06	20.25
M33675.m	rs6764543	A	G	0.4829	-0.0256	0.0054	1.94E-06	22.4746
M33683.m	rs10005516	A	G	0.0302	0.1169	0.0263	8.68E-06	19.7568

M33683.m	rs11218454	A	G	0.5504	-0.0442	0.0094	2.49E-06	22.11
M33683.m	rs12470581	A	G	0.0118	0.405	0.0878	3.99E-06	21.2775
M33683.m	rs13291283	T	C	0.0306	0.1277	0.0284	6.77E-06	20.2183
M33683.m	rs16851075	A	G	0.9762	-0.2919	0.0659	9.33E-06	19.6199
M33683.m	rs17115294	T	C	0.9457	-0.0966	0.0208	3.31E-06	21.5689
M33683.m	rs2074116	A	G	0.7665	-0.0503	0.0108	3.61E-06	21.6914
M33683.m	rs6450591	A	G	0.0819	-0.0871	0.0192	5.77E-06	20.5795
M33683.m	rs6823047	A	C	0.7299	-0.0982	0.0194	3.91E-07	25.6224
M33683.m	rs6930522	A	G	0.6305	-0.0509	0.0105	1.18E-06	23.4994
M33683.m	rs723124	T	C	0.0634	0.1622	0.0357	5.4E-06	20.6426
M33683.m	rs8085059	A	G	0.4278	-0.1427	0.0273	1.69E-07	27.3226
M33751.m	rs10510506	A	C	0.9801	-0.747	0.1682	8.98E-06	19.7237
M33751.m	rs11563459	T	C	0.8849	-0.0914	0.0202	5.78E-06	20.4734
M33751.m	rs12658525	T	C	0.0382	0.1682	0.0363	3.59E-06	21.4703
M33751.m	rs1322668	C	G	0.136	-0.0975	0.0211	3.92E-06	21.3523
M33751.m	rs1345223	T	C	0.9084	-0.1071	0.024	7.91E-06	19.9139
M33751.m	rs1535321	A	G	0.7808	-0.0732	0.0164	7.84E-06	19.9221
M33751.m	rs16843794	T	C	0.974	-0.7079	0.1428	7.1E-07	24.5747
M33751.m	rs17082709	A	C	0.9586	-0.167	0.0374	8.06E-06	19.9384
M33751.m	rs17091635	A	G	0.027	0.173	0.037	2.93E-06	21.8619
M33751.m	rs2173524	T	G	0.3588	0.0622	0.0135	4.02E-06	21.2282
M33751.m	rs2292787	A	G	0.0326	-0.6793	0.126	6.93E-08	29.0658
M33751.m	rs2859567	A	C	0.947	-0.1461	0.0315	3.52E-06	21.5119
M33751.m	rs29766	A	G	0.0862	0.1091	0.0242	6.39E-06	20.3244
M33751.m	rs4691942	A	G	0.8916	-0.2972	0.0644	3.91E-06	21.2974
M33751.m	rs4822679	A	G	0.9873	-0.934	0.2025	3.96E-06	21.2737
M33751.m	rs5998862	A	C	0.9487	-0.6716	0.141	1.91E-06	22.6873
M33751.m	rs6758453	T	C	0.4234	0.0656	0.0144	4.99E-06	20.7531
M33751.m	rs6938476	T	C	0.224	0.0705	0.0155	5.38E-06	20.6878
M33751.m	rs7150140	T	C	0.9873	-0.934	0.2025	3.96E-06	21.2737
M33751.m	rs7211635	A	G	0.8038	0.2648	0.0596	8.97E-06	19.7398
M33751.m	rs7334694	T	C	0.4609	-0.0643	0.0135	1.86E-06	22.6858
M33751.m	rs7665221	T	C	0.8107	-0.0796	0.0174	4.71E-06	20.928
M33751.m	rs7925072	T	C	0.822	-0.0826	0.0173	1.78E-06	22.7965
M33751.m	rs7934379	T	C	0.0127	0.934	0.2025	3.96E-06	21.2737
M33751.m	rs843172	T	G	0.9755	-0.7724	0.1682	4.37E-06	21.0879
M33751.m	rs922025	A	G	0.1942	-0.0846	0.018	2.51E-06	22.09
M33751.m	rs9451887	A	G	0.9873	-0.934	0.2025	3.96E-06	21.2737
M33751.m	rs9466463	C	G	0.0541	0.1372	0.0291	2.37E-06	22.2291
M33782.m	rs11066410	A	C	0.0516	0.0893	0.0188	2.11E-06	22.5625
M33782.m	rs1160835	T	C	0.0342	0.2555	0.0548	3.07E-06	21.7381
M33782.m	rs12405135	A	G	0.0929	0.0697	0.0147	2.06E-06	22.4818
M33782.m	rs12670780	A	G	0.8761	-0.1499	0.0294	3.3E-07	25.9961
M33782.m	rs17220921	T	C	0.0707	0.0757	0.0167	5.72E-06	20.5475
M33782.m	rs1986163	T	C	0.0335	0.264	0.0578	4.88E-06	20.8618
M33782.m	rs2623476	A	C	0.2405	0.0446	0.0099	6.56E-06	20.2955
M33782.m	rs4107301	A	G	0.1196	0.0605	0.0128	2.39E-06	22.3404
M33782.m	rs4695275	A	T	0.1015	0.1561	0.0333	2.73E-06	21.9744
M33782.m	rs7132897	A	T	0.0742	0.1811	0.0389	3.2E-06	21.6739
M33782.m	rs7544128	C	G	0.7224	0.0432	0.0092	2.8E-06	22.0491
M33782.m	rs756316	T	C	0.6015	-0.0373	0.0083	6.95E-06	20.1958
M33782.m	rs777828	A	G	0.9747	0.1299	0.0291	8.09E-06	19.9266
M33782.m	rs7828959	A	G	0.3477	0.0402	0.009	8.95E-06	19.9511
M33801.m	rs12066643	T	C	0.0891	-0.0482	0.0106	4.94E-06	20.6768
M33801.m	rs2073824	A	G	0.6508	0.0311	0.006	2.01E-07	26.8669

M33801.m	rs601338	A	G	0.4316	0.0413	0.0062	2.52E-11	44.3728
M33801.m	rs651007	T	C	0.2099	0.0653	0.0071	6.34E-20	84.5882
M33801.m	rs6597604	A	G	0.6976	-0.0294	0.0065	5.73E-06	20.4582
M33801.m	rs6893667	T	C	0.551	0.0319	0.0071	7.19E-06	20.1867
M33801.m	rs7579137	A	G	0.5493	-0.027	0.0059	4.77E-06	20.9423
M33801.m	rs9308699	T	C	0.0742	-0.0501	0.0113	9.62E-06	19.6571
M33801.m	rs9684934	T	G	0.0187	0.0951	0.0209	5.17E-06	20.7047
M33821.m	rs10035040	A	G	0.2331	-0.0331	0.0074	8.39E-06	20.0075
M33821.m	rs10187244	A	T	0.7692	-0.0184	0.0036	2.96E-07	26.1235
M33821.m	rs10899114	A	G	0.8976	0.026	0.0052	4.58E-07	25
M33821.m	rs12062130	T	C	0.9849	-0.0639	0.0133	1.49E-06	23.0833
M33821.m	rs12431406	C	G	0.0744	-0.0274	0.0062	8.39E-06	19.5307
M33821.m	rs12449476	T	C	0.2067	-0.0162	0.0036	8.1E-06	20.25
M33821.m	rs17013280	A	G	0.0898	-0.0493	0.0109	5.9E-06	20.4569
M33821.m	rs17085774	A	G	0.0693	-0.0276	0.0061	6.88E-06	20.4719
M33821.m	rs174455	A	G	0.6459	-0.0182	0.0035	1.92E-07	27.04
M33821.m	rs2482705	A	G	0.1531	-0.0201	0.0044	4.06E-06	20.8683
M33821.m	rs2720495	C	G	0.3666	0.0154	0.0035	9.61E-06	19.36
M33821.m	rs272461	A	G	0.2709	0.0159	0.0035	6.26E-06	20.6376
M33821.m	rs3198697	T	C	0.4174	0.0193	0.0034	1.65E-08	32.2223
M33821.m	rs4504394	T	C	0.2788	0.0318	0.0069	3.97E-06	21.2401
M33821.m	rs6817243	A	G	0.2809	0.0164	0.0035	3.29E-06	21.9559
M33821.m	rs7181157	C	G	0.2136	0.0167	0.0036	4.83E-06	21.5193
M33821.m	rs748196	T	G	0.4603	0.016	0.0034	3.33E-06	22.1453
M33821.m	rs7659812	A	G	0.5527	-0.0161	0.0035	3.55E-06	21.16
M33821.m	rs9321466	C	G	0.9628	0.0376	0.008	2.74E-06	22.09
M33821.m	rs968567	T	C	0.1798	0.04	0.0045	2.84E-19	79.0123
M33821.m	rs980142	A	G	0.1644	-0.023	0.005	4.44E-06	21.16
M33822.m	rs11773405	T	C	0.7497	-0.0169	0.0037	5.3E-06	20.8627
M33822.m	rs12631637	C	G	0.6794	0.0169	0.0037	3.73E-06	20.8627
M33822.m	rs12727188	T	G	0.9165	-0.0277	0.0062	8.8E-06	19.9607
M33822.m	rs13121822	A	G	0.2121	0.0207	0.0044	2.23E-06	22.1327
M33822.m	rs2269543	T	C	0.2719	-0.0162	0.0036	8.66E-06	20.25
M33822.m	rs6727350	A	T	0.1525	-0.041	0.009	5E-06	20.7531
M33822.m	rs7151939	T	G	0.1357	-0.0224	0.0046	1.22E-06	23.7127
M33822.m	rs7737048	T	C	0.08	0.0306	0.0066	3.92E-06	21.4959
M33833.m	rs10031835	T	C	0.694	-0.0439	0.0088	6.52E-07	24.8865
M33833.m	rs11078645	A	G	0.8453	0.0332	0.0075	9.62E-06	19.5954
M33833.m	rs12715789	A	G	0.8197	0.0214	0.0047	5.16E-06	20.7316
M33833.m	rs685747	C	G	0.9581	-0.0431	0.0092	2.96E-06	21.9472
M33833.m	rs7096907	T	C	0.181	-0.022	0.0046	2.15E-06	22.8733
M33833.m	rs7239714	A	G	0.7264	-0.0382	0.0086	9.6E-06	19.7301
M33833.m	rs7575509	T	C	0.4488	0.035	0.0076	4.64E-06	21.2084
M33833.m	rs7937	T	C	0.5585	-0.0165	0.0037	8.79E-06	19.8868
M33835.m	rs10261620	T	G	0.6829	-0.041	0.0087	2.27E-06	22.209
M33835.m	rs1041323	T	C	0.3811	-0.0729	0.0156	3E-06	21.8376
M33835.m	rs13103561	T	C	0.7102	-0.0418	0.0085	9.87E-07	24.1833
M33835.m	rs1402168	T	G	0.8834	0.1077	0.0239	6.49E-06	20.3065
M33835.m	rs2028829	T	C	0.9566	-0.0848	0.019	7.74E-06	19.9198
M33835.m	rs4530331	A	G	0.3391	-0.0367	0.0079	3.39E-06	21.5813
M33835.m	rs542320	A	T	0.3812	0.0853	0.0162	1.52E-07	27.7248
M33835.m	rs9317917	A	C	0.1628	-0.0479	0.0105	5.3E-06	20.811
M33835.m	rs9349529	A	C	0.5228	-0.0348	0.0078	8.84E-06	19.9053
M33864.m	rs10088108	A	G	0.809	-0.0465	0.0102	5.18E-06	20.7829
M33864.m	rs10136315	A	G	0.5986	-0.0378	0.0079	1.86E-06	22.8944

M33864.m	rs12986863	T	C	0.2858	0.0404	0.0088	5.01E-06	21.0764
M33864.m	rs1360605	C	G	0.9704	0.1092	0.0234	2.96E-06	21.7778
M33864.m	rs2789615	A	G	0.1728	-0.047	0.0106	8.92E-06	19.66
M33864.m	rs4781436	A	G	0.0141	0.2121	0.0478	9.23E-06	19.6891
M33864.m	rs4947639	T	G	0.1513	0.0561	0.0125	6.69E-06	20.1421
M33864.m	rs6925144	T	C	0.5429	0.0527	0.0116	5.76E-06	20.6398
M33864.m	rs924931	T	C	0.5401	-0.0353	0.0078	6.14E-06	20.4814
M33871.m	rs10072514	C	G	0.2388	0.0218	0.0048	5.04E-06	20.6267
M33871.m	rs10884473	A	G	0.9203	-0.0298	0.0067	9.11E-06	19.7826
M33871.m	rs12210045	A	G	0.4239	-0.0171	0.0036	2.13E-06	22.5625
M33871.m	rs13098081	C	G	0.9	0.0291	0.0065	6.86E-06	20.0428
M33871.m	rs1535	A	G	0.662	-0.0203	0.0035	6.15E-09	33.64
M33871.m	rs247197	T	C	0.382	-0.019	0.004	2.03E-06	22.5625
M33871.m	rs249619	T	C	0.567	-0.0325	0.0069	2.61E-06	22.1855
M33871.m	rs4256853	T	C	0.9893	-0.1412	0.0319	9.58E-06	19.5924
M33871.m	rs432642	A	T	0.1441	-0.0237	0.0052	4.09E-06	20.7726
M33871.m	rs6034091	A	G	0.0124	0.1302	0.0288	6.14E-06	20.4379
M33871.m	rs6426630	A	G	0.0286	-0.0885	0.0195	5.37E-06	20.5976
M33871.m	rs6593732	T	C	0.4672	-0.0164	0.0034	1.63E-06	23.2664
M33871.m	rs8104825	A	G	0.4384	0.0153	0.0034	7.29E-06	20.25
M33877.m	rs10832313	A	G	0.9183	-0.1452	0.0322	6.67E-06	20.3339
M33877.m	rs11714789	T	C	0.9731	-0.3581	0.0753	1.96E-06	22.6162
M33877.m	rs11993732	T	C	0.9774	-0.2732	0.0605	6.3E-06	20.3916
M33877.m	rs12411368	T	C	0.0333	0.3186	0.0619	2.66E-07	26.4917
M33877.m	rs12414068	A	G	0.9608	-0.2351	0.0527	8.19E-06	19.9014
M33877.m	rs13082822	T	G	0.8387	-0.1452	0.0322	6.73E-06	20.3339
M33877.m	rs13225659	A	T	0.8762	-0.1616	0.0311	2.08E-07	26.9999
M33877.m	rs13294186	A	G	0.9887	-0.6236	0.1343	3.43E-06	21.5606
M33877.m	rs1383681	A	G	0.4097	0.086	0.0193	8.08E-06	19.8556
M33877.m	rs16875784	A	G	0.0114	0.5363	0.1079	6.74E-07	24.7043
M33877.m	rs16982831	A	G	0.0284	0.3127	0.0704	8.86E-06	19.7292
M33877.m	rs16994356	T	G	0.9773	-0.4144	0.0763	5.6E-08	29.4979
M33877.m	rs17006323	A	G	0.9636	-0.1619	0.0345	2.62E-06	22.0219
M33877.m	rs17073398	T	C	0.0276	0.4078	0.0883	3.87E-06	21.3291
M33877.m	rs17115338	T	C	0.9866	-0.6712	0.1168	9.04E-09	33.0231
M33877.m	rs17259313	C	G	0.0169	0.3357	0.0698	1.51E-06	23.1309
M33877.m	rs244904	A	G	0.5145	-0.0916	0.0188	1.15E-06	23.7397
M33877.m	rs2826321	A	G	0.9847	-0.5286	0.1043	4E-07	25.6854
M33877.m	rs3752559	T	G	0.0114	0.5132	0.1086	2.29E-06	22.3313
M33877.m	rs3791160	T	C	0.0205	0.4005	0.0856	2.87E-06	21.8906
M33877.m	rs4785153	A	G	0.0201	0.3758	0.0832	6.35E-06	20.4017
M33877.m	rs4897530	T	C	0.0228	0.4842	0.1018	1.97E-06	22.6232
M33877.m	rs6109922	A	T	0.9822	-0.4676	0.0984	2.01E-06	22.5818
M33877.m	rs6759985	T	G	0.0471	0.2189	0.0481	5.33E-06	20.711
M33877.m	rs6794638	A	T	0.9677	-0.3484	0.0788	9.7E-06	19.5481
M33877.m	rs7908275	A	C	0.979	-0.3564	0.0747	1.81E-06	22.7632
M33877.m	rs7996156	T	C	0.0169	0.4229	0.0956	9.68E-06	19.5686
M33877.m	rs827283	A	G	0.7613	-0.1151	0.0256	6.95E-06	20.2149
M33877.m	rs9288697	T	G	0.0503	0.2691	0.0603	8.11E-06	19.9156
M33877.m	rs9317012	T	C	0.8991	-0.1562	0.0317	8.49E-07	24.2797
M33883.m	rs1053871	T	C	0.615	0.0527	0.0111	2.14E-06	22.5411
M33883.m	rs10869731	T	C	0.7861	-0.0647	0.0146	9.01E-06	19.6383
M33883.m	rs11110563	T	G	0.417	-0.0485	0.011	9.88E-06	19.4401
M33883.m	rs11163633	A	C	0.4597	0.0508	0.0112	5.6E-06	20.5727
M33883.m	rs12263193	A	G	0.7306	-0.0584	0.0129	6.34E-06	20.4949

M33883.m	rs12429941	A	G	0.029	-0.1462	0.033	9.29E-06	19.6276
M33883.m	rs138832	A	G	0.7832	0.0779	0.0156	6.18E-07	24.9359
M33883.m	rs1397793	A	G	0.6623	-0.0564	0.0122	3.65E-06	21.3717
M33883.m	rs1494950	A	G	0.9762	0.1843	0.0367	5.31E-07	25.2185
M33883.m	rs1671152	T	G	0.1503	-0.0666	0.0151	9.75E-06	19.4534
M33883.m	rs2271316	C	G	0.3822	0.1133	0.0113	1.79E-23	100.532
M33883.m	rs312462	A	G	0.0968	-0.0898	0.0183	8.76E-07	24.0797
M33883.m	rs6580981	A	G	0.4501	-0.0551	0.0113	1.12E-06	23.7764
M33883.m	rs684448	T	G	0.1797	-0.0616	0.0138	8.12E-06	19.9252
M33883.m	rs953628	T	G	0.2013	-0.0607	0.0134	5.74E-06	20.5195
M33884.m	rs11717259	A	G	0.6186	-0.0201	0.0044	4.33E-06	20.8683
M33884.m	rs11883258	C	G	0.3339	0.0208	0.0043	1.05E-06	23.3986
M33884.m	rs12068349	A	G	0.0257	0.0556	0.0125	9.23E-06	19.7847
M33884.m	rs2123517	T	C	0.023	0.1158	0.024	1.46E-06	23.2806
M33884.m	rs2915420	T	C	0.1613	-0.0261	0.0056	3.19E-06	21.7223
M33884.m	rs3770914	T	C	0.8905	0.0537	0.0116	3.94E-06	21.4305
M33884.m	rs393228	T	G	0.0697	0.034	0.0075	5.62E-06	20.5511
M33884.m	rs6685187	A	G	0.4088	-0.0432	0.0041	2.88E-26	111.02
M33884.m	rs7621575	A	C	0.1276	-0.0257	0.0058	7.76E-06	19.6341
M33884.m	rs7826057	A	G	0.6661	-0.0345	0.0078	9.43E-06	19.5636
M33884.m	rs8037595	T	C	0.9141	-0.0329	0.0074	9.15E-06	19.7664
M33884.m	rs9552232	A	G	0.0599	0.076	0.0172	9.68E-06	19.5241
M33884.m	rs9654430	A	G	0.5156	-0.0189	0.0042	5.92E-06	20.25
M33885.m	rs10282242	C	G	0.1667	0.1019	0.0222	4.45E-06	21.0689
M33885.m	rs1122407	A	G	0.9521	-0.0802	0.0181	9.72E-06	19.6332
M33885.m	rs11679513	A	G	0.0743	-0.0702	0.0147	1.89E-06	22.8055
M33885.m	rs12050606	C	G	0.0791	-0.0662	0.0146	5.73E-06	20.5594
M33885.m	rs1511938	A	G	0.5175	-0.0366	0.0078	2.82E-06	22.0178
M33885.m	rs1810195	A	C	0.9065	-0.0608	0.0132	3.97E-06	21.2158
M33885.m	rs335952	T	C	0.1902	-0.0494	0.0098	4.05E-07	25.4098
M33885.m	rs4698919	T	G	0.0145	-0.1672	0.0376	8.64E-06	19.7741
M33885.m	rs7191710	A	T	0.0535	0.1589	0.0357	8.64E-06	19.8112
M33885.m	rs7655244	T	G	0.0616	0.0735	0.0164	7.15E-06	20.0857
M33885.m	rs7718486	T	C	0.7269	0.0426	0.0091	2.68E-06	21.9147
M33885.m	rs9839976	A	G	0.0718	-0.1468	0.0312	2.53E-06	22.1382
M33892.m	rs10023843	T	G	0.9859	0.0606	0.0125	1.27E-06	23.5031
M33892.m	rs10152981	A	G	0.6622	-0.0132	0.0029	5.99E-06	20.7182
M33892.m	rs10232298	A	G	0.3412	-0.0129	0.0029	8.69E-06	19.7872
M33892.m	rs1026686	T	C	0.8064	0.017	0.0038	9.68E-06	20.0139
M33892.m	rs1057744	T	C	0.5174	0.0129	0.0029	9.65E-06	19.7872
M33892.m	rs10764832	T	C	0.788	-0.0177	0.0038	3.78E-06	21.696
M33892.m	rs10938365	A	C	0.4014	0.0147	0.0029	4.12E-07	25.6944
M33892.m	rs11902058	A	C	0.986	0.0622	0.0132	2.27E-06	22.2041
M33892.m	rs12037134	A	G	0.9401	-0.0291	0.0066	9.37E-06	19.4401
M33892.m	rs12299255	A	G	0.8452	-0.0174	0.0039	6.34E-06	19.9053
M33892.m	rs12356268	A	G	0.9452	-0.0297	0.0067	8.31E-06	19.65
M33892.m	rs12521683	A	G	0.6013	-0.0133	0.0029	5.05E-06	21.0333
M33892.m	rs12716182	A	T	0.2885	0.0175	0.0038	3.9E-06	21.2084
M33892.m	rs1544149	T	C	0.6652	0.013	0.0029	7.22E-06	20.0951
M33892.m	rs167675	C	G	0.371	0.013	0.0029	7.54E-06	20.0951
M33892.m	rs17380093	T	C	0.5345	-0.0135	0.0029	2.75E-06	21.6706
M33892.m	rs17420616	C	G	0.0545	0.0307	0.0067	4.16E-06	20.9955
M33892.m	rs17468022	T	C	0.4198	0.014	0.0029	1.49E-06	23.3056
M33892.m	rs1861512	T	C	0.3349	-0.0134	0.0029	3.81E-06	21.3508
M33892.m	rs1995777	T	C	0.5594	-0.014	0.0029	1.37E-06	23.3056

M33892.m	rs20301	A	G	0.5617	0.0135	0.0029	2.98E-06	21.6706
M33892.m	rs2116833	A	G	0.35	-0.0155	0.0029	1.05E-07	28.5672
M33892.m	rs2889961	A	G	0.3641	0.0136	0.0029	2.7E-06	21.9929
M33892.m	rs3007381	T	G	0.6033	0.0131	0.0029	6.43E-06	20.4055
M33892.m	rs3783687	A	C	0.9851	-0.212	0.0468	5.94E-06	20.5201
M33892.m	rs4467622	C	G	0.5956	-0.0134	0.0029	3.53E-06	21.3508
M33892.m	rs6882710	A	G	0.5077	-0.0132	0.0029	5.26E-06	20.7182
M33892.m	rs6891011	A	G	0.8862	-0.0244	0.0048	3.64E-07	25.8403
M33892.m	rs717355	T	G	0.6339	0.0136	0.0029	2.82E-06	21.9929
M33892.m	rs7584770	C	G	0.0203	0.1604	0.0359	7.73E-06	19.9627
M33892.m	rs891527	A	T	0.5847	0.0138	0.0029	2.06E-06	22.6445
M33901.m	rs10177768	A	G	0.6419	-0.0197	0.0044	8.01E-06	20.046
M33901.m	rs10505870	A	G	0.2702	0.0251	0.0053	2.1E-06	22.4283
M33901.m	rs12829704	A	G	0.1892	-0.0564	0.0061	1.47E-20	85.4867
M33901.m	rs12867367	A	C	0.7789	-0.0236	0.0052	5.44E-06	20.5976
M33901.m	rs12877392	A	C	0.8993	-0.0437	0.0096	5.85E-06	20.7215
M33901.m	rs13054841	A	T	0.8357	0.0293	0.0061	1.27E-06	23.0715
M33901.m	rs16853746	T	C	0.9739	0.0739	0.0155	1.98E-06	22.7314
M33901.m	rs17057341	A	G	0.0187	0.0771	0.0174	9.62E-06	19.6341
M33901.m	rs17192023	A	G	0.1157	-0.0334	0.0075	9.13E-06	19.8322
M33901.m	rs1854521	A	G	0.4968	0.0213	0.0044	1.17E-06	23.4344
M33901.m	rs2291073	T	G	0.9215	0.0428	0.0086	5.81E-07	24.768
M33901.m	rs2432026	A	G	0.0587	-0.0451	0.0097	3.49E-06	21.6177
M33901.m	rs2547231	A	C	0.833	0.0409	0.0061	2.82E-11	44.9559
M33901.m	rs2867882	A	G	0.4696	0.0215	0.0044	9.66E-07	23.8765
M33901.m	rs4149056	T	C	0.832	-0.0805	0.0058	9.49E-44	192.635
M33901.m	rs4402594	T	C	0.4067	-0.0451	0.0091	7.99E-07	24.5624
M33901.m	rs4953276	A	C	0.1272	-0.032	0.0068	2.42E-06	22.1453
M33901.m	rs5758873	A	G	0.4519	0.0196	0.0044	6.9E-06	19.843
M33901.m	rs6565488	A	C	0.1172	0.0331	0.007	1.98E-06	22.3594
M33901.m	rs6584331	T	C	0.4873	0.0204	0.0043	2.55E-06	22.5073
M33901.m	rs6987078	A	G	0.2224	0.0237	0.0052	5.33E-06	20.7726
M33901.m	rs9864709	T	G	0.2158	-0.0238	0.0053	5.74E-06	20.1652
M33910.m	rs10272008	T	C	0.022	0.101	0.022	4.41E-06	21.0764
M33910.m	rs10483863	A	G	0.4704	-0.0191	0.0043	9.49E-06	19.7301
M33910.m	rs10487813	A	C	0.4438	-0.0199	0.0043	3.94E-06	21.4175
M33910.m	rs10491617	T	G	0.9743	-0.0841	0.0183	4.32E-06	21.1198
M33910.m	rs11594263	A	G	0.0971	0.0339	0.0074	5.22E-06	20.9863
M33910.m	rs11671495	A	G	0.0411	0.1123	0.0247	5.23E-06	20.6712
M33910.m	rs13361985	A	G	0.4413	0.0195	0.0043	6.53E-06	20.5652
M33910.m	rs1344998	T	C	0.5529	-0.0191	0.0043	9.88E-06	19.7301
M33910.m	rs17503355	A	G	0.0288	0.0777	0.0171	5.41E-06	20.6467
M33910.m	rs1952103	T	G	0.7585	-0.0244	0.0052	3.07E-06	22.0178
M33910.m	rs2246344	T	G	0.7708	-0.0261	0.0052	4.6E-07	25.1927
M33910.m	rs4571932	A	G	0.8822	0.0302	0.0068	8.91E-06	19.724
M33910.m	rs7645103	A	G	0.3571	-0.0236	0.005	2.27E-06	22.2784
M33910.m	rs9532194	A	G	0.1881	0.0288	0.0064	5.91E-06	20.25
M33935.x	rs11896399	T	C	0.0255	0.122	0.0244	5.47E-07	25
M33935.x	rs12485393	A	G	0.3273	0.0403	0.0081	6.08E-07	24.7537
M33935.x	rs16880327	T	G	0.978	0.1136	0.025	5.76E-06	20.6479
M33935.x	rs17137472	T	C	0.5085	-0.0386	0.0076	3.86E-07	25.7957
M33935.x	rs1741285	A	G	0.4861	-0.0351	0.0078	5.95E-06	20.25
M33935.x	rs506506	A	G	0.5029	0.0341	0.0074	4.16E-06	21.2347
M33935.x	rs6074527	T	C	0.7046	0.0419	0.0087	1.43E-06	23.1947
M33935.x	rs7183091	T	C	0.7327	0.0366	0.0082	8.83E-06	19.9221

M33935.x	rs768846	T	C	0.9819	0.1284	0.0285	6.67E-06	20.2974
M33935.x	rs8085038	A	C	0.1003	-0.0991	0.0219	6.32E-06	20.4767
M33935.x	rs9348689	T	G	0.5152	0.0379	0.0084	5.79E-06	20.3573
M33936.m	rs10868458	A	T	0.3461	-0.0165	0.0035	1.92E-06	22.2245
M33936.m	rs10873878	T	G	0.8609	-0.0283	0.0053	8.86E-08	28.5116
M33936.m	rs17304141	T	G	0.9786	-0.1417	0.0147	4.07E-22	92.9191
M33936.m	rs17638535	A	G	0.0208	-0.0652	0.0132	8.75E-07	24.3976
M33936.m	rs2133336	A	G	0.3986	0.0152	0.0034	9.56E-06	19.9862
M33936.m	rs2720162	T	G	0.6076	-0.0154	0.0034	7.31E-06	20.5156
M33936.m	rs274567	T	C	0.37	0.0158	0.0034	3.93E-06	21.5952
M33936.m	rs3743527	T	C	0.2204	0.0194	0.0042	4.7E-06	21.3356
M33936.m	rs4672596	T	C	0.3979	0.0152	0.0034	9.6E-06	19.9862
M33936.m	rs5745515	T	C	0.0972	0.0468	0.0069	8.24E-12	46.0038
M33936.m	rs5745542	C	G	0.8975	-0.046	0.0058	2.17E-15	62.9013
M33936.m	rs6816650	A	T	0.8503	0.0251	0.0051	9.23E-07	24.2218
M33936.m	rs6849801	C	G	0.6966	-0.0276	0.0041	1.6E-11	45.3159
M33936.m	rs6907864	T	C	0.1108	0.0261	0.0058	5.79E-06	20.25
M33936.m	rs7130402	A	G	0.9349	0.0324	0.0073	9.88E-06	19.699
M33936.m	rs721204	A	G	0.1116	0.0465	0.0057	6.29E-16	66.5512
M33936.m	rs7246133	T	C	0.6581	0.0205	0.0046	9.48E-06	19.8606
M33936.m	rs7334793	T	C	0.4953	-0.0152	0.0034	8.04E-06	19.9862
M33936.m	rs7534288	T	C	0.0272	-0.0574	0.0116	7.89E-07	24.4854
M33936.m	rs7552404	A	G	0.6984	0.0746	0.004	1.27E-77	347.823
M33936.m	rs8396	T	C	0.6972	0.0478	0.0041	1.38E-31	135.921
M33936.m	rs884760	T	C	0.277	0.0188	0.0042	7.16E-06	20.0363
M33936.m	rs888860	A	C	0.3513	-0.0181	0.0037	9.39E-07	23.9306
M33937.m	rs11151591	A	C	0.7667	0.0213	0.0046	4.11E-06	21.4409
M33937.m	rs11953823	T	C	0.3065	0.0326	0.0072	6.54E-06	20.5008
M33937.m	rs12141041	T	C	0.4692	-0.0253	0.0036	1.77E-12	49.3897
M33937.m	rs16892052	C	G	0.0265	0.0775	0.0168	3.81E-06	21.2806
M33937.m	rs2378733	A	G	0.1266	0.0214	0.0047	5.07E-06	20.7316
M33937.m	rs2403254	T	C	0.5505	-0.0409	0.0036	1.26E-30	129.075
M33937.m	rs2679896	T	C	0.2902	0.0169	0.0037	3.96E-06	20.8627
M33937.m	rs2906973	A	T	0.9593	-0.0468	0.0095	8.26E-07	24.2686
M33937.m	rs3125596	A	G	0.9148	-0.0542	0.0115	2.39E-06	22.2128
M33937.m	rs320265	T	C	0.0748	-0.0331	0.007	2.45E-06	22.3594
M33937.m	rs3821477	T	C	0.927	-0.0288	0.0063	5.35E-06	20.898
M33937.m	rs4328432	A	G	0.1812	0.0207	0.0046	5.5E-06	20.25
M33937.m	rs7092160	A	C	0.7194	-0.0168	0.0037	5.47E-06	20.6165
M33937.m	rs7933681	A	G	0.094	0.0267	0.0059	5.57E-06	20.4795
M33937.m	rs7938942	T	C	0.5934	-0.018	0.0036	7.19E-07	25
M33937.m	rs893971	A	G	0.5882	-0.0197	0.0036	4.05E-08	29.9452
M33939.m	rs10492430	T	C	0.0203	-0.036	0.0079	5.15E-06	20.7659
M33939.m	rs11655421	A	T	0.1996	0.0145	0.0032	5.82E-06	20.5322
M33939.m	rs11692169	T	C	0.2324	0.0122	0.0027	4.7E-06	20.417
M33939.m	rs12857329	T	C	0.8752	0.0168	0.0034	9.75E-07	24.4152
M33939.m	rs1869289	A	C	0.2164	-0.0121	0.0026	4.31E-06	21.6583
M33939.m	rs2103694	A	G	0.1336	0.0151	0.0034	9.3E-06	19.724
M33939.m	rs3891367	T	C	0.8915	0.0358	0.0075	1.73E-06	22.7847
M33939.m	rs4666270	T	G	0.8392	-0.0138	0.0028	1.02E-06	24.2908
M33939.m	rs4831776	T	C	0.9508	0.0237	0.0052	6.28E-06	20.7726
M33939.m	rs508482	T	G	0.8237	0.0131	0.0027	1.12E-06	23.5405
M33939.m	rs6453164	A	G	0.288	0.0231	0.0052	8.65E-06	19.7341
M33939.m	rs817338	T	C	0.8271	0.0121	0.0027	7.06E-06	20.0837
M33939.m	rs9397729	A	T	0.264	0.0167	0.0038	9.99E-06	19.3137

M33941.m	rs10036208	T	C	0.3421	0.0199	0.0041	1.49E-06	23.558
M33941.m	rs11722868	A	G	0.3074	0.0312	0.0041	4.86E-14	57.9084
M33941.m	rs1220578	A	G	0.0778	-0.0323	0.0072	6.89E-06	20.1252
M33941.m	rs12932433	T	G	0.7139	0.0186	0.0041	6.34E-06	20.5806
M33941.m	rs17157494	A	G	0.074	0.0353	0.0075	2.21E-06	22.1527
M33941.m	rs17304141	T	G	0.9785	-0.0982	0.0154	1.87E-10	40.6613
M33941.m	rs17638535	A	G	0.0209	-0.072	0.014	2.48E-07	26.449
M33941.m	rs2062541	A	G	0.6144	0.0225	0.004	2.89E-08	31.6406
M33941.m	rs2140779	T	C	0.1483	-0.0247	0.0056	9.87E-06	19.4544
M33941.m	rs2156436	T	C	0.0307	0.1445	0.0302	1.73E-06	22.894
M33941.m	rs5745515	T	C	0.0963	0.0375	0.007	7.99E-08	28.699
M33941.m	rs5745542	C	G	0.8972	-0.035	0.0064	5.54E-08	29.9072
M33941.m	rs6475272	T	C	0.2277	-0.0235	0.0048	9.07E-07	23.9692
M33941.m	rs6537038	A	G	0.4014	-0.0188	0.0041	4.93E-06	21.0256
M33941.m	rs6816650	A	T	0.8513	0.0259	0.0058	7.66E-06	19.9408
M33941.m	rs721204	A	G	0.1121	0.0345	0.0064	6.51E-08	29.0588
M33941.m	rs7534288	T	C	0.0273	-0.0567	0.0124	4.35E-06	20.9085
M33941.m	rs7552404	A	G	0.6984	0.0563	0.0041	3.32E-43	188.56
M33941.m	rs7819483	A	G	0.7179	-0.0187	0.0042	7.46E-06	19.8237
M33941.m	rs8396	T	C	0.6969	0.0533	0.0041	8.69E-38	169
M33941.m	rs9556	T	C	0.3103	-0.0189	0.0042	6.08E-06	20.25
M33955.m	rs10059896	T	C	0.786	-0.0083	0.0018	5.63E-06	21.2623
M33955.m	rs10283250	A	C	0.1254	0.0119	0.0027	9.12E-06	19.4252
M33955.m	rs10814589	A	G	0.3122	0.0086	0.0018	1.53E-06	22.8272
M33955.m	rs10913710	A	G	0.212	0.0081	0.0018	8.9E-06	20.25
M33955.m	rs10953026	A	G	0.6177	-0.008	0.0018	5.85E-06	19.7531
M33955.m	rs10966017	A	G	0.2684	-0.0085	0.0018	3.23E-06	22.2994
M33955.m	rs11654527	T	G	0.1988	0.0081	0.0018	8.24E-06	20.25
M33955.m	rs1235119	A	T	0.8949	-0.013	0.0027	1.08E-06	23.1824
M33955.m	rs12880914	T	C	0.7389	-0.0085	0.0018	2.45E-06	22.2994
M33955.m	rs1433098	T	G	0.6439	0.008	0.0018	8.14E-06	19.7531
M33955.m	rs1442583	A	G	0.0594	-0.017	0.0036	1.94E-06	22.2994
M33955.m	rs17814868	A	G	0.7346	-0.0081	0.0018	8.4E-06	20.25
M33955.m	rs1822906	A	G	0.2032	0.0099	0.0018	8.79E-08	30.25
M33955.m	rs1875897	A	G	0.257	0.009	0.002	6.8E-06	20.25
M33955.m	rs2273534	T	C	0.947	0.019	0.0037	4.2E-07	26.3696
M33955.m	rs2490289	T	C	0.2222	0.0096	0.0018	1.53E-07	28.4444
M33955.m	rs2748805	T	C	0.3004	0.0085	0.0018	2.31E-06	22.2994
M33955.m	rs353694	A	G	0.4518	-0.0085	0.0018	4.09E-06	22.2994
M33955.m	rs3735474	A	G	0.9341	-0.0165	0.0036	3.97E-06	21.0069
M33955.m	rs3851441	C	G	0.2195	-0.0087	0.0018	2.3E-06	23.3611
M33955.m	rs4414129	T	C	0.9342	-0.0161	0.0036	8E-06	20.0008
M33955.m	rs4459845	A	C	0.8009	0.0084	0.0018	5.08E-06	21.7778
M33955.m	rs474566	A	G	0.8714	0.0116	0.0026	9.86E-06	19.9053
M33955.m	rs476092	A	C	0.2852	0.0083	0.0018	3.25E-06	21.2623
M33955.m	rs6714201	A	G	0.483	0.0081	0.0018	4.41E-06	20.25
M33955.m	rs6777718	T	G	0.2783	-0.0086	0.0019	3.51E-06	20.4875
M33955.m	rs7124459	T	C	0.3757	-0.008	0.0018	7.58E-06	19.7531
M33955.m	rs7547153	A	G	0.5893	-0.0086	0.0018	1.13E-06	22.8272
M33955.m	rs7759176	T	G	0.2976	0.0082	0.0018	4.35E-06	20.7531
M33955.m	rs8028440	T	C	0.2136	0.0095	0.0019	3.15E-07	25
M33955.m	rs804572	T	G	0.0607	-0.0372	0.0082	5.72E-06	20.5806
M33955.m	rs8052052	T	G	0.2037	-0.0096	0.0018	1.86E-07	28.4444
M33955.m	rs9318232	T	C	0.2312	-0.0104	0.002	1.05E-07	27.04
M33955.m	rs9880529	T	C	0.8762	0.0117	0.0026	7.47E-06	20.25

M33957.m	rs10019315	T	C	0.4852	0.0173	0.0039	7.26E-06	19.6772
M33957.m	rs10849438	T	G	0.8893	0.0287	0.0065	9.68E-06	19.4956
M33957.m	rs12100883	T	C	0.1216	-0.0266	0.006	9.11E-06	19.6544
M33957.m	rs12201199	A	T	0.9375	-0.0391	0.0079	7.65E-07	24.4962
M33957.m	rs3824359	T	C	0.8602	0.0241	0.0054	8.74E-06	19.918
M33957.m	rs6530980	T	C	0.0364	0.0491	0.0107	4.02E-06	21.0569
M33957.m	rs6573597	A	G	0.0874	-0.0327	0.0072	6.33E-06	20.6267
M33957.m	rs6833678	T	C	0.3165	-0.018	0.0039	3.5E-06	21.3018
M33957.m	rs7727774	A	G	0.4534	0.0267	0.0059	6.38E-06	20.4795
M33957.m	rs8081778	T	C	0.1587	0.0259	0.0052	7.06E-07	24.8081
M33960.m	rs10019315	T	C	0.4837	0.0121	0.0026	2.99E-06	21.6583
M33960.m	rs10465080	A	T	0.2818	-0.0121	0.0026	4.69E-06	21.6583
M33960.m	rs12202351	T	C	0.7073	0.0118	0.0026	7.14E-06	20.5976
M33960.m	rs12605650	T	C	0.3031	0.012	0.0026	5.13E-06	21.3018
M33960.m	rs1366823	A	G	0.6447	0.0118	0.0026	6.94E-06	20.5976
M33960.m	rs1405992	A	C	0.06	0.0263	0.0059	7.9E-06	19.8704
M33960.m	rs16968711	A	G	0.8679	0.0205	0.0041	4.65E-07	25
M33960.m	rs17089560	T	C	0.0209	0.0468	0.0092	3.39E-07	25.8771
M33960.m	rs303610	A	T	0.2958	-0.0117	0.0026	8.88E-06	20.25
M33960.m	rs4132201	A	G	0.395	-0.0127	0.0026	9.4E-07	23.8595
M33960.m	rs4736583	A	C	0.6256	-0.012	0.0026	4.89E-06	21.3018
M33960.m	rs476092	A	C	0.2865	0.012	0.0026	5.09E-06	21.3018
M33960.m	rs4817185	C	G	0.1025	0.0373	0.0084	7.78E-06	19.7178
M33960.m	rs6061764	A	G	0.314	-0.014	0.003	3.06E-06	21.7778
M33960.m	rs6977605	A	C	0.9311	-0.0242	0.005	1.67E-06	23.4256
M33960.m	rs9489697	T	C	0.4207	0.0116	0.0026	8.36E-06	19.9053
M33960.m	rs969948	T	C	0.6023	-0.0117	0.0026	5.74E-06	20.25
M33961.m	rs11100892	A	G	0.4863	0.0161	0.0034	2.61E-06	22.423
M33961.m	rs12412179	T	G	0.0972	-0.025	0.0056	8.75E-06	19.9298
M33961.m	rs1397264	T	C	0.8746	-0.023	0.0051	5.66E-06	20.3383
M33961.m	rs1405992	A	C	0.0625	0.0338	0.0074	5.29E-06	20.8627
M33961.m	rs1489866	T	G	0.8208	0.0205	0.0043	1.72E-06	22.7285
M33961.m	rs1595416	A	G	0.2639	0.0164	0.0036	6.93E-06	20.7531
M33961.m	rs2076521	T	C	0.0211	-0.0928	0.0208	8.28E-06	19.9053
M33961.m	rs3844028	A	C	0.2257	0.0182	0.0041	7.96E-06	19.7049
M33961.m	rs4082919	T	G	0.5103	-0.0156	0.0035	7.29E-06	19.8661
M33961.m	rs476092	A	C	0.2962	0.0179	0.0035	4.51E-07	26.1559
M33961.m	rs7305796	A	G	0.8905	0.0338	0.0074	4.3E-06	20.8627
M33961.m	rs748196	T	G	0.4608	0.0152	0.0034	8.15E-06	19.9862
M33961.m	rs8004616	A	G	0.9877	-0.0766	0.0172	8.56E-06	19.8336
M33968.m	rs1037422	A	G	0.2223	-0.0446	0.0083	7.41E-08	28.8744
M33968.m	rs10742833	T	C	0.0697	-0.0337	0.0075	7.82E-06	20.19
M33968.m	rs11133486	T	G	0.2844	-0.0189	0.0042	6.58E-06	20.25
M33968.m	rs11709408	A	G	0.3576	0.0198	0.0044	5.63E-06	20.25
M33968.m	rs11941943	A	G	0.2281	-0.0202	0.0043	2.82E-06	22.0681
M33968.m	rs12484413	A	G	0.8721	0.0492	0.0104	2.04E-06	22.3802
M33968.m	rs17711814	T	C	0.2564	0.0214	0.0043	5.09E-07	24.768
M33968.m	rs2194101	A	G	0.8777	0.0323	0.0069	3.41E-06	21.9133
M33968.m	rs2279501	C	G	0.5933	0.0221	0.0041	5.6E-08	29.0547
M33968.m	rs2501242	T	G	0.2274	-0.0216	0.0048	8.24E-06	20.25
M33968.m	rs2915420	T	C	0.1599	-0.0231	0.005	3.98E-06	21.3444
M33968.m	rs3748771	T	G	0.9879	-0.1431	0.0322	8.89E-06	19.75
M33968.m	rs4668061	A	G	0.0379	-0.0583	0.0127	4.67E-06	21.0732
M33968.m	rs603424	A	G	0.1825	-0.0308	0.0049	4.27E-10	39.5102
M33968.m	rs6544157	A	G	0.9013	-0.0292	0.0061	2.01E-06	22.9143

M33968.m	rs7164415	C	G	0.9751	0.0958	0.0212	5.95E-06	20.4202
M33968.m	rs728797	T	C	0.07	0.04	0.009	8.81E-06	19.7531
M33968.m	rs8088457	A	G	0.0307	0.0557	0.0114	9.12E-07	23.8727
M33968.m	rs9888349	T	C	0.7735	-0.0191	0.0043	8.38E-06	19.7301
M33969.m	rs11236516	T	C	0.897	0.0283	0.0058	1.24E-06	23.8077
M33969.m	rs12583004	T	C	0.0726	-0.0314	0.0071	9.9E-06	19.5588
M33969.m	rs174601	T	C	0.3398	-0.0336	0.0042	7.93E-16	64
M33969.m	rs2265047	A	G	0.2807	0.0335	0.0075	8.71E-06	19.9511
M33969.m	rs2427612	T	C	0.364	-0.0286	0.0061	3.01E-06	21.9823
M33969.m	rs3750045	T	C	0.9018	0.0266	0.006	9.08E-06	19.6544
M33969.m	rs4529031	T	C	0.9555	-0.0841	0.0186	6.41E-06	20.444
M33969.m	rs4811800	A	G	0.484	-0.0212	0.0045	2.52E-06	22.1946
M33969.m	rs6473533	T	G	0.807	-0.0217	0.0049	9.08E-06	19.6122
M33969.m	rs6986249	T	C	0.4413	-0.0177	0.0036	6.3E-07	24.1736
M33969.m	rs7142028	A	C	0.5693	-0.0161	0.0035	4.06E-06	21.16
M33971.m	rs10098688	A	C	0.3373	0.0242	0.0054	7.4E-06	20.0837
M33971.m	rs11118120	A	C	0.8498	-0.0209	0.0047	9.83E-06	19.7741
M33971.m	rs1348656	T	C	0.7978	-0.0195	0.0041	1.67E-06	22.6205
M33971.m	rs220992	A	G	0.4501	0.0155	0.0033	2.69E-06	22.0615
M33971.m	rs4129516	T	C	0.7271	-0.0156	0.0035	7.13E-06	19.8661
M33971.m	rs4288115	A	T	0.3119	-0.0172	0.0038	7.2E-06	20.4875
M33972.m	rs10004910	A	G	0.5629	0.017	0.0034	5.58E-07	25
M33972.m	rs10098688	A	C	0.337	0.0263	0.0059	8.25E-06	19.8704
M33972.m	rs1348656	T	C	0.798	-0.0232	0.0042	3.46E-08	30.5125
M33972.m	rs35350	A	G	0.9681	0.0621	0.0131	2.16E-06	22.4719
M33972.m	rs530942	A	G	0.86	-0.0373	0.0081	4.69E-06	21.2055
M33972.m	rs703138	T	G	0.3743	0.0152	0.0034	8.62E-06	19.9862
M33972.m	rs7677730	T	C	0.4936	0.0151	0.0034	7.58E-06	19.724
M33973.m	rs10228787	T	C	0.0145	-0.2245	0.0409	3.97E-08	30.1291
M33973.m	rs11981478	T	C	0.0861	-0.1844	0.01	2.8E-75	340.034
M33973.m	rs13222543	T	C	0.0203	-0.3474	0.0241	3.31E-47	207.79
M33973.m	rs1419352	C	G	0.3905	0.0291	0.0063	3.72E-06	21.3356
M33973.m	rs1474102	T	C	0.3367	-0.0297	0.006	6.34E-07	24.5025
M33973.m	rs17638060	T	G	0.2995	-0.0314	0.0069	5.13E-06	20.7091
M33973.m	rs1939769	A	G	0.0643	0.0622	0.012	2.06E-07	26.8669
M33973.m	rs2141160	T	G	0.2462	0.0368	0.0066	2.37E-08	31.0891
M33973.m	rs4439540	A	G	0.7403	0.0524	0.0109	1.45E-06	23.1105
M33973.m	rs474229	T	G	0.6017	0.0492	0.0058	3.51E-17	71.9572
M33973.m	rs6465737	T	G	0.223	-0.0378	0.0067	1.91E-08	31.8298
M33973.m	rs6578042	T	C	0.857	0.047	0.0104	5.93E-06	20.4234
M33973.m	rs7078211	A	G	0.8445	-0.0404	0.0076	1.26E-07	28.2576
M33973.m	rs7152358	A	G	0.889	0.0428	0.0093	3.83E-06	21.1798
M33973.m	rs7778571	A	G	0.7541	0.0449	0.0066	8.91E-12	46.2812
M33973.m	rs807019	T	C	0.1495	0.0372	0.0084	9.53E-06	19.6122
M33973.m	rs9676475	T	C	0.2129	0.0344	0.0074	3.05E-06	21.6099
M34035.m	rs1034879	A	C	0.0455	0.0765	0.0172	8.89E-06	19.7818
M34035.m	rs13132193	A	T	0.2154	0.0317	0.0068	3.54E-06	21.7321
M34035.m	rs1348656	T	C	0.798	-0.019	0.0043	9.69E-06	19.5241
M34035.m	rs2242415	A	C	0.6232	0.0162	0.0035	4.36E-06	21.4237
M34035.m	rs6125916	T	G	0.1936	-0.0204	0.0043	1.8E-06	22.5073
M34035.m	rs6125916	T	G	0.1936	-0.0204	0.0043	1.8E-06	22.5073
M34040.m	rs10209727	A	G	0.1471	0.0248	0.0054	5.04E-06	21.0919
M34040.m	rs1165209	A	G	0.5531	-0.0226	0.0036	5.13E-10	39.4105
M34040.m	rs1318987	A	C	0.157	-0.0547	0.0054	6.6E-24	102.609
M34040.m	rs13538	A	G	0.7828	0.1186	0.0044	7.9E-157	726.548

M34040.m	rs1466245	T	C	0.0511	0.0481	0.0081	2.78E-09	35.2631
M34040.m	rs17009399	C	G	0.0575	-0.0808	0.0081	1.56E-23	99.5068
M34040.m	rs17348756	T	C	0.1083	0.0355	0.0062	1.35E-08	32.7849
M34040.m	rs17349853	T	G	0.9295	-0.0435	0.0085	2.92E-07	26.1903
M34040.m	rs1933041	A	G	0.6651	-0.0182	0.0037	7.23E-07	24.1958
M34040.m	rs2469215	T	C	0.1051	-0.0307	0.0062	6.7E-07	24.5185
M34040.m	rs4363421	A	G	0.7027	0.017	0.0038	7.07E-06	20.0139
M34040.m	rs4434099	T	C	0.8282	-0.0216	0.0046	2.82E-06	22.0491
M34040.m	rs4447076	A	G	0.6145	-0.0175	0.0037	1.94E-06	22.3703
M34040.m	rs4865204	T	C	0.481	-0.0329	0.0071	3.77E-06	21.4721
M34040.m	rs550353	A	G	0.1911	0.0205	0.0046	7.24E-06	19.8606
M34040.m	rs6689147	T	C	0.2377	0.0226	0.0044	3.86E-07	26.3822
M34040.m	rs6710726	A	G	0.6575	0.0296	0.0037	1.27E-15	64
M34040.m	rs7607175	T	C	0.9247	0.0345	0.0075	4.55E-06	21.16
M34040.m	rs7718044	T	C	0.4678	0.0172	0.0036	2.3E-06	22.8272
M34040.m	rs862225	T	C	0.0912	-0.0291	0.0066	9.28E-06	19.4401
M34062.m	rs10161949	A	G	0.0146	-0.0737	0.0163	6.25E-06	20.4437
M34062.m	rs10497685	A	G	0.8809	0.0082	0.0018	9.2E-06	20.7531
M34062.m	rs1080101	T	C	0.2695	0.0156	0.0034	3.72E-06	21.0519
M34062.m	rs10906643	A	G	0.8721	0.0081	0.0018	7.66E-06	20.25
M34062.m	rs11029744	T	G	0.0206	0.0209	0.0046	5.55E-06	20.6432
M34062.m	rs11076175	A	G	0.845	0.011	0.002	3.8E-08	30.25
M34062.m	rs11583844	A	C	0.9439	0.0134	0.0027	6.34E-07	24.631
M34062.m	rs12658008	T	G	0.2091	0.0083	0.0018	2.66E-06	21.2623
M34062.m	rs12884251	A	G	0.1395	0.0081	0.0018	8.02E-06	20.25
M34062.m	rs17105481	A	G	0.8711	0.0087	0.0019	3.1E-06	20.9668
M34062.m	rs17150366	A	T	0.8838	0.0083	0.0018	6.17E-06	21.2623
M34062.m	rs17352	T	G	0.8945	0.0233	0.005	3.13E-06	21.7156
M34062.m	rs1926815	A	C	0.1804	-0.0084	0.0018	1.85E-06	21.7778
M34062.m	rs1938299	A	G	0.889	0.009	0.002	6.8E-06	20.25
M34062.m	rs2029490	A	G	0.2811	0.0082	0.0018	5.6E-06	20.7531
M34062.m	rs2162481	T	C	0.1224	-0.0083	0.0018	6.89E-06	21.2623
M34062.m	rs2301783	A	C	0.9151	0.0122	0.0027	4.9E-06	20.417
M34062.m	rs2780242	T	C	0.1179	-0.0117	0.0019	7.08E-10	37.9197
M34062.m	rs325614	A	G	0.9673	0.0171	0.0036	2.63E-06	22.5625
M34062.m	rs3729908	T	C	0.1234	0.009	0.0018	9.91E-07	25
M34062.m	rs3764387	A	G	0.1754	-0.0087	0.0018	1.61E-06	23.3611
M34062.m	rs4394864	T	C	0.0645	-0.0119	0.0026	5.52E-06	20.9482
M34062.m	rs4748538	T	C	0.88	-0.0083	0.0018	7.41E-06	21.2623
M34062.m	rs4964963	A	G	0.8192	-0.008	0.0018	6.19E-06	19.7531
M34062.m	rs559422	A	C	0.9115	0.0119	0.0026	5.77E-06	20.9482
M34062.m	rs5999780	T	C	0.1528	0.0081	0.0018	7.21E-06	20.25
M34062.m	rs6124759	C	G	0.8895	0.0084	0.0019	6.5E-06	19.5457
M34062.m	rs6749150	A	G	0.8699	0.0094	0.0018	3.52E-07	27.2716
M34062.m	rs6898299	T	G	0.1197	0.0082	0.0018	7.5E-06	20.7531
M34062.m	rs7167675	C	G	0.9875	-0.06	0.013	3.68E-06	21.3018
M34062.m	rs9973286	C	G	0.8771	-0.0093	0.0018	3.23E-07	26.6944
M34106.m	rs11594597	C	G	0.1287	0.0336	0.0074	6.1E-06	20.6165
M34106.m	rs1188542	T	C	0.6033	-0.0256	0.0056	5.1E-06	20.898
M34106.m	rs12588138	C	G	0.0374	0.0694	0.0139	6.33E-07	24.9281
M34106.m	rs1552059	A	G	0.0942	0.0519	0.0104	6.38E-07	24.9039
M34106.m	rs16924889	A	G	0.0992	-0.0636	0.014	5.95E-06	20.6376
M34106.m	rs2228069	A	G	0.6086	-0.0226	0.0051	8.74E-06	19.6371
M34106.m	rs2798002	A	G	0.0628	-0.1151	0.0255	6.48E-06	20.3737
M34106.m	rs28384	T	C	0.2933	0.0241	0.0052	4.46E-06	21.4797

M34106.m	rs28898590	T	G	0.0617	-0.059	0.0107	4.01E-08	30.4044
M34106.m	rs28900385	T	C	0.9666	-0.0705	0.0132	8.69E-08	28.5253
M34106.m	rs2992439	A	G	0.7289	-0.0271	0.0058	3.01E-06	21.8315
M34106.m	rs4149000	T	C	0.1369	0.0336	0.0073	4.63E-06	21.1852
M34106.m	rs4281899	T	G	0.7416	0.0341	0.0059	6.26E-09	33.4045
M34106.m	rs4627421	T	C	0.2337	0.0267	0.0059	7.19E-06	20.4795
M34106.m	rs4736435	A	C	0.2466	0.0497	0.0111	8E-06	20.0478
M34106.m	rs6723936	A	G	0.6407	0.0294	0.0051	1.11E-08	33.2318
M34106.m	rs6848587	T	C	0.1655	-0.0311	0.0067	3.7E-06	21.5462
M34106.m	rs6911308	T	G	0.5796	-0.0251	0.0052	1.34E-06	23.2992
M34106.m	rs887829	T	C	0.3243	0.081	0.0051	1.79E-56	252.249
M34109.x	rs1024561	A	G	0.0787	-0.2369	0.0525	6.37E-06	20.3616
M34109.x	rs10519822	T	C	0.9788	-0.9974	0.2217	6.82E-06	20.2399
M34109.x	rs11842833	A	G	0.9424	0.2985	0.0647	3.97E-06	21.2853
M34109.x	rs12047385	T	C	0.9679	0.298	0.0657	5.8E-06	20.5732
M34109.x	rs12462688	A	T	0.988	0.95	0.192	7.5E-07	24.4819
M34109.x	rs12483918	T	C	0.9829	0.4285	0.0916	2.9E-06	21.8832
M34109.x	rs12661060	T	C	0.1122	-0.1644	0.0358	4.4E-06	21.0881
M34109.x	rs12702537	A	G	0.986	0.925	0.19	1.13E-06	23.7015
M34109.x	rs12937280	T	C	0.0146	-0.568	0.1243	4.89E-06	20.8812
M34109.x	rs12969690	A	T	0.984	0.447	0.098	5.09E-06	20.8048
M34109.x	rs13049288	A	T	0.011	-0.957	0.192	6.22E-07	24.844
M34109.x	rs13190410	T	G	0.1061	0.169	0.0363	3.14E-06	21.6751
M34109.x	rs13394534	A	G	0.0248	-0.3573	0.0768	3.27E-06	21.6443
M34109.x	rs1565932	A	T	0.9122	0.2624	0.0564	3.34E-06	21.6456
M34109.x	rs1652045	T	C	0.8547	0.3852	0.0855	6.59E-06	20.2974
M34109.x	rs16902795	C	G	0.0235	-0.3693	0.0821	6.87E-06	20.2336
M34109.x	rs17124948	T	C	0.011	-0.692	0.137	4.39E-07	25.5136
M34109.x	rs2002691	A	C	0.9844	0.5054	0.1006	5E-07	25.2391
M34109.x	rs2255960	C	G	0.01	-0.931	0.192	1.24E-06	23.5124
M34109.x	rs2582067	A	G	0.977	0.91	0.187	1.14E-06	23.681
M34109.x	rs2588032	T	G	0.0324	-0.5461	0.1229	8.91E-06	19.7442
M34109.x	rs2663073	A	G	0.927	0.4837	0.1064	5.42E-06	20.6666
M34109.x	rs2928923	A	G	0.979	0.4932	0.0901	4.38E-08	29.9638
M34109.x	rs4448374	T	C	0.0283	-0.2957	0.0619	1.8E-06	22.8203
M34109.x	rs6745020	A	G	0.011	-0.932	0.191	1.06E-06	23.8103
M34109.x	rs6812391	A	G	0.0441	-0.7293	0.1606	5.61E-06	20.6216
M34109.x	rs6944023	A	C	0.0219	-0.9465	0.1878	4.67E-07	25.401
M34109.x	rs7088085	A	G	0.984	0.885	0.186	1.96E-06	22.6392
M34109.x	rs7912030	A	G	0.979	0.821	0.18	5.09E-06	20.8037
M34109.x	rs7967284	A	G	0.4004	-0.1045	0.0235	9.02E-06	19.7741
M34109.x	rs7970370	T	C	0.0206	-0.5726	0.1168	9.37E-07	24.0335
M34109.x	rs9465476	T	C	0.14	-0.43	0.0885	1.19E-06	23.6075
M34109.x	rs9558761	C	G	0.0177	-0.5296	0.1107	1.71E-06	22.8876
M34109.x	rs9929954	T	G	0.7364	0.1231	0.0252	9.87E-07	23.8624
M34112.m	rs1072961	T	C	0.2668	-0.0336	0.0075	6.63E-06	20.0704
M34112.m	rs10875436	C	G	0.6582	-0.0257	0.0057	7.69E-06	20.329
M34112.m	rs1412535	T	C	0.5882	-0.0281	0.0057	8.9E-07	24.3032
M34112.m	rs17064491	A	T	0.0225	0.2812	0.0611	4.2E-06	21.1811
M34112.m	rs17745320	T	C	0.9628	0.0722	0.0158	4.97E-06	20.8814
M34112.m	rs2076434	A	G	0.8443	0.0342	0.0076	6.08E-06	20.25
M34112.m	rs2705534	T	G	0.9192	0.0517	0.0105	7.9E-07	24.2439
M34112.m	rs2821373	T	C	0.9257	0.0561	0.0114	8.61E-07	24.2168
M34112.m	rs2839011	T	C	0.2016	0.0356	0.0078	5.33E-06	20.831
M34112.m	rs4455656	A	G	0.1597	-0.0343	0.0076	6.88E-06	20.3686

M34112.m	rs4750514	A	G	0.5822	0.0269	0.0057	2.6E-06	22.2718
M34112.m	rs4850779	A	G	0.415	-0.0263	0.0057	4.4E-06	21.2893
M34112.m	rs6502243	T	G	0.5849	-0.0267	0.0058	3.48E-06	21.1917
M34112.m	rs6596884	T	C	0.4477	0.0332	0.0074	6.91E-06	20.1286
M34112.m	rs7836759	A	C	0.3393	-0.026	0.0058	6.61E-06	20.0951
M34112.m	rs7971484	T	G	0.1909	-0.0323	0.0067	1.54E-06	23.241
M34112.m	rs9297054	T	C	0.5207	-0.0255	0.0057	9.22E-06	20.0139
M34112.m	rs9319761	T	G	0.9697	-0.0869	0.0179	1.21E-06	23.5686
M34123.m	rs10410999	A	C	0.9182	0.0201	0.0044	6.06E-06	20.8683
M34123.m	rs1158932	A	C	0.5676	0.0122	0.0027	4.6E-06	20.417
M34123.m	rs12470772	T	C	0.4174	-0.0123	0.0028	8.43E-06	19.2972
M34123.m	rs164100	T	C	0.3925	0.0238	0.0054	9.25E-06	19.4252
M34123.m	rs17256713	T	C	0.0762	0.0207	0.0045	3.7E-06	21.16
M34123.m	rs202815	A	G	0.3799	-0.0123	0.0027	3.49E-06	20.7531
M34123.m	rs2187448	T	C	0.1296	-0.0186	0.0036	1.79E-07	26.6944
M34123.m	rs2234518	A	G	0.2653	0.012	0.0027	8.74E-06	19.7531
M34123.m	rs2235649	T	C	0.2159	-0.0184	0.0027	1.95E-11	46.4417
M34123.m	rs251456	A	G	0.1523	0.0159	0.0035	6.63E-06	20.6376
M34123.m	rs27419	T	C	0.6409	-0.0126	0.0027	2.6E-06	21.7778
M34123.m	rs3773977	T	C	0.7484	-0.0159	0.0034	2.63E-06	21.8694
M34123.m	rs3782411	T	C	0.3241	-0.0124	0.0028	7.53E-06	19.6122
M34123.m	rs4129624	T	G	0.9127	0.0243	0.0044	3.92E-08	30.5005
M34123.m	rs4271495	A	G	0.1	0.0233	0.0053	9.54E-06	19.3268
M34123.m	rs4675874	A	G	0.7094	-0.0243	0.0026	3.13E-20	87.3506
M34123.m	rs4738400	T	C	0.2947	0.0133	0.0027	6.33E-07	24.2647
M34123.m	rs523932	T	C	0.9868	0.054	0.011	8.3E-07	24.0992
M34123.m	rs6034944	C	G	0.9395	0.0587	0.013	5.81E-06	20.3887
M34123.m	rs6431170	A	C	0.2833	0.0125	0.0027	3.75E-06	21.4335
M34123.m	rs7483614	T	C	0.8665	0.0186	0.004	3.97E-06	21.6225
M34123.m	rs7590032	A	G	0.791	0.023	0.0027	5.49E-17	72.5652
M34123.m	rs878013	C	G	0.1689	0.0163	0.0034	1.97E-06	22.9836
M34214.m	rs10829734	C	G	0.8464	0.016	0.0035	4.9E-06	20.898
M34214.m	rs11045834	T	C	0.2911	-0.0171	0.0026	1.16E-10	43.2559
M34214.m	rs11045975	A	G	0.1919	-0.0151	0.0034	8.69E-06	19.724
M34214.m	rs11163340	A	G	0.3481	0.0118	0.0026	5.79E-06	20.5976
M34214.m	rs1260333	A	G	0.4472	0.0125	0.0026	1.26E-06	23.1139
M34214.m	rs12613874	A	G	0.6712	0.0125	0.0027	2.78E-06	21.4335
M34214.m	rs17064388	A	G	0.102	-0.0195	0.0042	4.41E-06	21.5561
M34214.m	rs17315876	A	G	0.4379	0.0118	0.0026	5.55E-06	20.5976
M34214.m	rs174578	A	T	0.3409	-0.0266	0.0026	3.02E-24	104.669
M34214.m	rs1871395	A	G	0.841	-0.0395	0.0034	3.66E-31	134.97
M34214.m	rs4521036	A	G	0.1388	0.0171	0.0035	1.33E-06	23.8702
M34214.m	rs4943187	C	G	0.9593	0.0343	0.0077	7.84E-06	19.843
M34214.m	rs4973862	T	C	0.6928	-0.0119	0.0026	5.92E-06	20.9482
M34214.m	rs6973089	A	T	0.9182	0.0408	0.0091	7.64E-06	20.1019
M34214.m	rs7267516	T	C	0.4894	-0.012	0.0026	2.6E-06	21.3018
M34214.m	rs7303969	T	C	0.8242	-0.0171	0.0036	2.64E-06	22.5625
M34214.m	rs7394871	A	C	0.0343	-0.0401	0.0086	3.6E-06	21.7416
M34214.m	rs7903596	A	G	0.988	-0.1278	0.0285	7.09E-06	20.1081
M34214.m	rs8736	T	C	0.4379	-0.0227	0.0026	8.45E-18	76.2263
M34214.m	rs9326061	C	G	0.8497	-0.0188	0.0034	4.39E-08	30.5744
M34214.m	rs9997	T	C	0.6544	0.012	0.0026	3.89E-06	21.3018
M34221.m	rs10927006	T	C	0.8659	0.0278	0.006	3.24E-06	21.4678
M34221.m	rs11966563	C	G	0.8182	0.0305	0.0063	1.31E-06	23.4379
M34221.m	rs17170000	C	G	0.8052	0.0245	0.0052	2E-06	22.1986

M34221.m	rs2267575	A	T	0.8736	-0.0423	0.0086	8.73E-07	24.1927
M34221.m	rs2275353	A	G	0.1625	0.0244	0.0053	4.51E-06	21.1947
M34221.m	rs335170	A	C	0.4021	0.0191	0.0043	7.51E-06	19.7301
M34221.m	rs4955937	A	G	0.5451	-0.0207	0.0044	2.17E-06	22.1327
M34221.m	rs6456737	T	C	0.2462	0.0228	0.0045	3.03E-07	25.6711
M34221.m	rs7148806	A	G	0.8294	-0.0474	0.0107	9.78E-06	19.6241
M34221.m	rs7759825	T	C	0.698	0.0222	0.0044	3.43E-07	25.4566
M34221.m	rs9569679	T	G	0.149	0.0278	0.006	3.62E-06	21.4678
M34221.m	rs9809853	T	C	0.7459	0.0196	0.0044	9.83E-06	19.843
M34244.m	rs1077835	A	G	0.7976	-0.0208	0.0035	1.82E-09	35.3176
M34244.m	rs10956695	T	C	0.4838	0.013	0.0027	1.43E-06	23.1824
M34244.m	rs10956723	T	C	0.9414	-0.0342	0.007	1.19E-06	23.8702
M34244.m	rs11183616	A	C	0.8054	-0.0155	0.0035	9.02E-06	19.6122
M34244.m	rs11228715	A	G	0.6124	-0.0123	0.0027	4.14E-06	20.7531
M34244.m	rs11811387	A	T	0.8227	0.016	0.0036	7.04E-06	19.7531
M34244.m	rs1260326	T	C	0.4046	0.0136	0.0027	2.83E-07	25.3717
M34244.m	rs12886864	A	G	0.3911	-0.0121	0.0027	7.09E-06	20.0837
M34244.m	rs13009980	A	G	0.019	-0.062	0.012	2.38E-07	26.6944
M34244.m	rs1341296	T	C	0.4596	0.0127	0.0027	2.79E-06	22.1248
M34244.m	rs1391971	A	G	0.1795	-0.0161	0.0035	3.9E-06	21.16
M34244.m	rs1532085	A	G	0.3838	0.0157	0.0027	5.1E-09	33.8121
M34244.m	rs17448272	A	G	0.9163	0.0232	0.005	3.92E-06	21.5296
M34244.m	rs1822378	T	C	0.6721	0.0315	0.0063	6.37E-07	25
M34244.m	rs1883995	A	G	0.6271	0.0121	0.0027	6.57E-06	20.0837
M34244.m	rs2444830	T	C	0.6646	0.0137	0.0027	4.52E-07	25.7462
M34244.m	rs3807839	T	C	0.2604	0.0162	0.0035	4.4E-06	21.4237
M34244.m	rs4566308	C	G	0.3608	-0.0119	0.0027	9.44E-06	19.4252
M34244.m	rs6510986	T	C	0.907	0.027	0.006	6.8E-06	20.25
M34244.m	rs7200919	A	G	0.4102	-0.0139	0.0027	2.01E-07	26.5034
M34244.m	rs7969341	A	G	0.833	-0.0213	0.0035	1.01E-09	37.0359
M34244.m	rs914702	C	G	0.1627	0.0356	0.008	7.5E-06	19.8025
M34244.m	rs945631	A	G	0.0183	-0.0497	0.0112	9.27E-06	19.6914
M34244.m	rs987693	A	G	0.4537	-0.012	0.0027	8E-06	19.7531
M34245.m	rs10512731	C	G	0.8047	0.0261	0.0054	1.15E-06	23.3611
M34245.m	rs10977991	A	G	0.9105	0.0326	0.0073	8.88E-06	19.943
M34245.m	rs11068695	A	G	0.0667	-0.0395	0.0083	2.06E-06	22.6484
M34245.m	rs12234425	A	C	0.8416	-0.0243	0.0055	9.33E-06	19.5203
M34245.m	rs12886782	A	G	0.2265	-0.0206	0.0046	8.11E-06	20.0548
M34245.m	rs1461941	C	G	0.6294	0.0226	0.0045	5.01E-07	25.2227
M34245.m	rs1630427	C	G	0.1047	-0.0289	0.0064	7.48E-06	20.3909
M34245.m	rs168190	T	C	0.4522	0.0465	0.0097	1.81E-06	22.9807
M34245.m	rs16852455	T	G	0.4794	-0.0164	0.0037	8.82E-06	19.6465
M34245.m	rs2070895	A	G	0.2076	0.025	0.0054	3.09E-06	21.4335
M34245.m	rs2912035	T	G	0.6198	0.0218	0.0045	1.3E-06	23.4686
M34245.m	rs2980872	A	G	0.461	-0.018	0.004	6.8E-06	20.25
M34245.m	rs308589	A	G	0.4721	-0.0196	0.0044	9.2E-06	19.843
M34245.m	rs311496	T	C	0.0866	-0.0338	0.0072	3.05E-06	22.0378
M34245.m	rs317279	A	G	0.6525	0.0202	0.0045	7.62E-06	20.1501
M34245.m	rs6004798	T	C	0.3908	-0.0201	0.0045	7.14E-06	19.9511
M34245.m	rs7796052	A	T	0.7667	0.021	0.0046	4.84E-06	20.8412
M34245.m	rs807741	T	G	0.0918	-0.0805	0.0167	1.38E-06	23.2359
M34245.m	rs923673	A	C	0.7212	0.0235	0.0046	2.98E-07	26.0988
M34283.m	rs10148180	C	G	0.9353	-0.026	0.005	2.51E-07	27.04
M34283.m	rs1028777	A	C	0.6523	-0.0086	0.0018	1.52E-06	22.8272
M34283.m	rs10444467	T	C	0.5177	0.0086	0.0018	1.71E-06	22.8272

M34283.m	rs10503601	T	C	0.9489	-0.0214	0.0047	6.17E-06	20.7316
M34283.m	rs11126005	A	G	0.3729	0.0083	0.0018	2.61E-06	21.2623
M34283.m	rs11170195	T	C	0.619	0.0088	0.0018	5.99E-07	23.9012
M34283.m	rs1146975	A	G	0.3702	0.0085	0.0018	1.55E-06	22.2994
M34283.m	rs11614753	T	C	0.9887	-0.0958	0.02	1.6E-06	22.9441
M34283.m	rs11778747	T	C	0.2583	-0.0194	0.0043	5.25E-06	20.3548
M34283.m	rs11801253	A	G	0.5459	-0.0094	0.0019	5.68E-07	24.4765
M34283.m	rs11865240	T	G	0.5259	-0.0085	0.0018	2.38E-06	22.2994
M34283.m	rs12136030	T	C	0.3726	-0.008	0.0018	6.23E-06	19.7531
M34283.m	rs12241172	T	C	0.695	0.0082	0.0018	7.58E-06	20.7531
M34283.m	rs12327010	A	G	0.6533	-0.0105	0.0024	8.45E-06	19.1406
M34283.m	rs12663966	A	T	0.3638	-0.0083	0.0018	3.64E-06	21.2623
M34283.m	rs12934578	A	G	0.3816	-0.0079	0.0018	9.98E-06	19.2623
M34283.m	rs13174601	T	C	0.9576	0.0604	0.0134	6.15E-06	20.3172
M34283.m	rs1324707	A	G	0.506	-0.009	0.002	6.8E-06	20.25
M34283.m	rs13259125	T	C	0.3366	-0.0082	0.0018	4.02E-06	20.7531
M34283.m	rs13269603	T	C	0.1598	-0.0114	0.0026	9.61E-06	19.2249
M34283.m	rs1338571	A	G	0.5069	0.0091	0.0018	3.07E-07	25.5586
M34283.m	rs1531507	A	G	0.2007	0.0127	0.0026	6.67E-07	23.8595
M34283.m	rs1536873	A	G	0.4478	-0.0093	0.0018	1.67E-07	26.6944
M34283.m	rs17345286	T	C	0.7364	0.0124	0.0025	5.07E-07	24.6016
M34283.m	rs1797387	A	G	0.49	-0.008	0.0018	6.71E-06	19.7531
M34283.m	rs188611	A	G	0.4253	0.0085	0.0018	1.67E-06	22.2994
M34283.m	rs1957025	A	G	0.4666	-0.0079	0.0018	8.61E-06	19.2623
M34283.m	rs2011069	A	G	0.37	0.027	0.002	1.56E-41	182.25
M34283.m	rs2245730	A	C	0.5574	-0.0082	0.0018	3.82E-06	20.7531
M34283.m	rs2372474	A	G	0.4893	0.0083	0.0018	2.24E-06	21.2623
M34283.m	rs2511225	A	C	0.6408	0.009	0.0018	5.53E-07	25
M34283.m	rs3017098	A	G	0.5517	0.0107	0.0018	1.48E-09	35.3364
M34283.m	rs313125	A	G	0.1782	-0.0117	0.0026	5.13E-06	20.25
M34283.m	rs4305952	C	G	0.6467	0.0084	0.0018	2.65E-06	21.7778
M34283.m	rs4690523	T	C	0.5242	-0.009	0.0018	3.48E-07	25
M34283.m	rs4888479	A	G	0.8234	0.0117	0.0025	4.24E-06	21.9024
M34283.m	rs4896596	T	C	0.4122	0.0082	0.0018	3.64E-06	20.7531
M34283.m	rs512721	A	G	0.1071	-0.0147	0.0033	9.28E-06	19.843
M34283.m	rs6683094	A	G	0.6238	0.0084	0.0018	2.72E-06	21.7778
M34283.m	rs6755681	T	C	0.0501	-0.0211	0.0043	8.62E-07	24.0784
M34283.m	rs7161226	A	C	0.9809	-0.0652	0.0134	1.1E-06	23.6748
M34283.m	rs7445640	T	G	0.5795	0.008	0.0018	6.95E-06	19.7531
M34283.m	rs7822643	A	G	0.8305	0.0115	0.0026	8.14E-06	19.5636
M34283.m	rs8008020	T	C	0.4143	-0.0117	0.0018	5.89E-11	42.25
M34289.m	rs10434643	A	G	0.8432	-0.0255	0.0057	8.39E-06	20.0139
M34289.m	rs10489084	T	C	0.7357	0.0223	0.0049	4.2E-06	20.7118
M34289.m	rs11532037	A	G	0.9564	-0.0535	0.011	1.17E-06	23.655
M34289.m	rs11634716	A	G	0.3458	-0.0206	0.0044	2.84E-06	21.9194
M34289.m	rs13228763	C	G	0.0663	-0.0582	0.0127	4.71E-06	21.0009
M34289.m	rs16215	T	C	0.9711	0.0694	0.0141	8.05E-07	24.2259
M34289.m	rs16841556	A	C	0.6765	-0.0212	0.0046	4.48E-06	21.2401
M34289.m	rs16853271	T	G	0.8831	0.0298	0.0065	5.04E-06	21.0187
M34289.m	rs16908783	A	G	0.2086	-0.0255	0.0051	5.52E-07	25
M34289.m	rs1821897	C	G	0.7792	0.0248	0.0052	1.49E-06	22.7456
M34289.m	rs2172831	A	G	0.0644	-0.0691	0.0156	9.24E-06	19.6204
M34289.m	rs4714588	T	C	0.7866	0.0231	0.005	4.86E-06	21.3444
M34289.m	rs574773	T	C	0.0733	-0.036	0.0079	5.97E-06	20.7659
M34289.m	rs6929665	A	G	0.6775	-0.0207	0.0047	8.92E-06	19.3975

M34289.m	rs7164146	T	C	0.1221	0.0282	0.0063	7.54E-06	20.0363
M34306.m	rs10835866	A	G	0.9826	0.1318	0.0283	3.31E-06	21.6899
M34306.m	rs1124386	T	C	0.0768	-0.0209	0.0045	3.22E-06	21.5709
M34306.m	rs11681014	A	G	0.3403	-0.013	0.0027	1.27E-06	23.1824
M34306.m	rs11925980	T	C	0.0325	-0.0341	0.0074	4.08E-06	21.2347
M34306.m	rs12465802	A	G	0.2879	-0.0302	0.0026	2.35E-30	134.917
M34306.m	rs12475101	A	C	0.0269	-0.0369	0.0077	1.56E-06	22.9653
M34306.m	rs12598924	T	G	0.2613	-0.0127	0.0027	2.82E-06	22.1248
M34306.m	rs12903172	T	C	0.6383	0.0118	0.0026	7.87E-06	20.5976
M34306.m	rs12996558	C	G	0.7649	-0.013	0.0027	2.15E-06	23.1824
M34306.m	rs13005339	A	G	0.283	-0.0147	0.0027	2.7E-08	29.642
M34306.m	rs1389923	T	G	0.2697	0.0122	0.0027	7.18E-06	20.417
M34306.m	rs1452253	A	C	0.6045	0.0173	0.0032	6.6E-08	29.2275
M34306.m	rs17355487	T	C	0.3071	-0.0122	0.0027	5.01E-06	20.417
M34306.m	rs21806	T	C	0.3173	-0.0137	0.0028	1.17E-06	23.9401
M34306.m	rs2271196	T	C	0.9802	-0.0445	0.01	9.13E-06	19.8025
M34306.m	rs2377324	T	C	0.7038	-0.0121	0.0027	7.39E-06	20.0837
M34306.m	rs2550251	T	G	0.3941	-0.0123	0.0027	5.51E-06	20.7531
M34306.m	rs3800993	T	C	0.1701	0.0224	0.0036	4.06E-10	38.716
M34306.m	rs4622179	T	C	0.7749	-0.0125	0.0027	4.73E-06	21.4335
M34306.m	rs7071570	T	C	0.0701	0.0214	0.0046	2.66E-06	21.6427
M34306.m	rs7080146	T	C	0.705	0.0121	0.0027	8E-06	20.0837
M34306.m	rs7120732	T	C	0.018	-0.055	0.011	5.73E-07	25
M34306.m	rs7568884	T	C	0.2496	-0.0183	0.0026	5.39E-12	49.5399
M34306.m	rs7570971	A	C	0.3257	-0.0292	0.0026	3.03E-28	126.13
M34306.m	rs9389095	T	C	0.3794	-0.012	0.0027	6.97E-06	19.7531
M34306.m	rs9393409	A	G	0.3342	-0.0127	0.0028	4.11E-06	20.5727
M34306.m	rs9972792	A	C	0.6339	0.0124	0.0027	3.18E-06	21.0919
M34314.m	rs10030952	T	C	0.3662	-0.0704	0.014	4.7E-07	25.2865
M34314.m	rs10826860	T	C	0.6363	-0.039	0.0082	1.74E-06	22.6205
M34314.m	rs16856300	A	C	0.6138	-0.0372	0.0083	7.46E-06	20.0877
M34314.m	rs4454960	C	G	0.8989	-0.0745	0.0165	6.47E-06	20.3866
M34314.m	rs4953342	A	G	0.7182	-0.0432	0.0096	7.2E-06	20.25
M34314.m	rs5993792	T	C	0.7247	-0.0687	0.0154	8.11E-06	19.9009
M34314.m	rs6575039	T	C	0.7656	0.0456	0.0102	7.54E-06	19.9862
M34314.m	rs6597533	A	G	0.9771	0.1306	0.0283	4.04E-06	21.2968
M34314.m	rs9345410	C	G	0.9419	0.0835	0.0186	7.56E-06	20.1533
M34314.m	rs9935968	A	G	0.3282	0.0425	0.0096	8.91E-06	19.5991
M34322.m	rs10243870	T	G	0.841	-0.0784	0.0177	9.18E-06	19.6194
M34322.m	rs10485760	T	C	0.9733	-0.2299	0.0437	1.48E-07	27.6767
M34322.m	rs10811676	T	C	0.0885	0.1666	0.0372	7.56E-06	20.0569
M34322.m	rs11059438	T	C	0.0133	0.3901	0.0759	2.74E-07	26.4161
M34322.m	rs1107458	T	C	0.9867	-0.3698	0.0773	1.7E-06	22.8863
M34322.m	rs1111835	A	G	0.027	0.2508	0.0563	8.52E-06	19.8444
M34322.m	rs11656024	A	G	0.2014	0.1074	0.0236	5.45E-06	20.7102
M34322.m	rs11817310	T	C	0.9575	-0.17	0.0367	3.61E-06	21.4568
M34322.m	rs11878582	C	G	0.986	-0.3746	0.0799	2.79E-06	21.9807
M34322.m	rs12070861	T	C	0.9323	-0.1619	0.0338	1.7E-06	22.9435
M34322.m	rs12092233	T	G	0.0133	0.3663	0.0775	2.28E-06	22.3393
M34322.m	rs12639034	T	C	0.975	-0.1844	0.0394	2.84E-06	21.9043
M34322.m	rs12646855	A	G	0.06	0.1563	0.0347	6.71E-06	20.2889
M34322.m	rs13176512	A	G	0.0839	0.1108	0.0251	9.78E-06	19.4864
M34322.m	rs13354441	A	G	0.8928	-0.087	0.0175	6.33E-07	24.7151
M34322.m	rs13429065	T	C	0.0343	0.2346	0.0503	3.08E-06	21.753
M34322.m	rs1447600	T	C	0.81	0.11	0.023	1.73E-06	22.8733

M34322.m	rs159314	A	G	0.054	0.1866	0.041	5.44E-06	20.7136
M34322.m	rs17066980	T	C	0.0473	0.1974	0.0433	5.16E-06	20.7835
M34322.m	rs17157982	T	C	0.98	-0.2906	0.0644	6.34E-06	20.362
M34322.m	rs17619273	A	G	0.1542	0.071	0.0154	4.02E-06	21.2557
M34322.m	rs17797732	T	C	0.0526	0.1238	0.028	9.53E-06	19.549
M34322.m	rs1824189	T	C	0.6979	-0.0631	0.0138	5.25E-06	20.9074
M34322.m	rs197712	T	C	0.8636	0.098	0.0214	4.67E-06	20.9713
M34322.m	rs2280350	T	C	0.0187	0.4061	0.0917	9.57E-06	19.6122
M34322.m	rs2425619	C	G	0.1195	0.0968	0.0219	9.91E-06	19.5372
M34322.m	rs3813761	T	C	0.9476	-0.189	0.0424	8.27E-06	19.8697
M34322.m	rs418889	A	G	0.0143	0.3821	0.0801	1.86E-06	22.7556
M34322.m	rs4589435	T	C	0.7069	-0.064	0.0142	6.2E-06	20.3134
M34322.m	rs6562537	A	G	0.9859	-0.3483	0.0787	9.55E-06	19.5865
M34322.m	rs6961104	T	C	0.9667	-0.247	0.0493	5.56E-07	25.1015
M34322.m	rs7009700	C	G	0.9503	-0.1461	0.0315	3.54E-06	21.5119
M34322.m	rs707922	T	G	0.04	0.2854	0.0406	2.12E-12	49.4147
M34322.m	rs726389	T	C	0.9733	-0.31	0.0521	2.71E-09	35.4036
M34322.m	rs736289	T	C	0.6138	0.0663	0.0149	8.8E-06	19.7995
M34322.m	rs745932	A	G	0.1263	-0.0846	0.0179	2.25E-06	22.3375
M34322.m	rs8014529	A	G	0.9782	-0.3141	0.0637	8.21E-07	24.314
M34322.m	rs8096899	T	G	0.026	0.3152	0.0707	8.39E-06	19.8762
M34322.m	rs9375892	T	G	0.8819	-0.0867	0.0182	2.02E-06	22.6932
M34322.m	rs9577914	T	C	0.6467	-0.0739	0.0166	8.48E-06	19.8186
M34327.m	rs10916770	A	T	0.8056	0.0837	0.0183	5.05E-06	20.9194
M34327.m	rs12200779	A	G	0.0859	0.0871	0.0191	5.1E-06	20.7955
M34327.m	rs12466783	C	G	0.9759	-0.1784	0.0401	8.56E-06	19.7925
M34327.m	rs12748135	T	C	0.8469	-0.0701	0.0157	7.54E-06	19.9359
M34327.m	rs1373562	T	C	0.1574	0.0713	0.0155	3.89E-06	21.16
M34327.m	rs2826581	T	G	0.789	-0.0695	0.0139	5.58E-07	25
M34327.m	rs2925759	T	C	0.1897	-0.067	0.0148	6.28E-06	20.494
M34327.m	rs7036822	A	G	0.807	-0.0663	0.0142	3.12E-06	21.7997
M34327.m	rs898219	T	G	0.6202	-0.0664	0.0138	1.44E-06	23.1514
M34329.m	rs1000702	T	G	0.4182	-0.0418	0.0092	5.39E-06	20.6432
M34329.m	rs10481772	T	C	0.8431	0.0552	0.0124	9.13E-06	19.8169
M34329.m	rs10971203	T	C	0.4075	-0.0436	0.0092	2.09E-06	22.4594
M34329.m	rs11077095	A	G	0.3208	0.0694	0.0155	7.51E-06	20.0473
M34329.m	rs11097262	T	C	0.1593	0.0557	0.0122	5.11E-06	20.8445
M34329.m	rs12046325	A	C	0.7637	-0.0493	0.0107	4.14E-06	21.2288
M34329.m	rs1360637	A	G	0.0171	0.2353	0.0529	8.79E-06	19.7848
M34329.m	rs16855886	T	G	0.0407	0.1068	0.0235	5.32E-06	20.6541
M34329.m	rs2876587	T	C	0.1046	0.0684	0.015	5.01E-06	20.7936
M34329.m	rs472038	C	G	0.077	0.111	0.0242	4.49E-06	21.0385
M34329.m	rs7655049	T	C	0.1616	-0.0553	0.0118	2.91E-06	21.9627
M34329.m	rs9965173	A	G	0.8936	0.0643	0.0146	9.91E-06	19.3962
M34336.m	rs1005464	A	G	0.229	0.0141	0.003	2.2E-06	22.09
M34336.m	rs10064959	C	G	0.0226	0.2452	0.0549	7.87E-06	19.9479
M34336.m	rs11039351	T	C	0.0406	0.0338	0.0069	8.47E-07	23.9958
M34336.m	rs11058897	T	C	0.0551	-0.027	0.0059	4.76E-06	20.9423
M34336.m	rs12456708	T	G	0.2598	-0.0133	0.003	6.93E-06	19.6544
M34336.m	rs13419959	T	C	0.7317	-0.0158	0.003	9.57E-08	27.7378
M34336.m	rs16963974	A	G	0.0772	-0.0305	0.0068	6.13E-06	20.1179
M34336.m	rs17144083	A	T	0.057	-0.0282	0.0059	1.89E-06	22.8452
M34336.m	rs17478715	C	G	0.9209	-0.0218	0.0049	9.78E-06	19.7934
M34336.m	rs17521554	T	C	0.0166	0.3198	0.0697	4.46E-06	21.0519
M34336.m	rs2380875	T	G	0.0551	0.2035	0.0427	1.86E-06	22.7129

M34336.m	rs4533654	T	G	0.5883	-0.0155	0.003	1.61E-07	26.6944
M34336.m	rs606567	A	G	0.8204	0.0177	0.0039	6.6E-06	20.5976
M34336.m	rs7029730	A	C	0.2493	-0.0142	0.003	1.57E-06	22.4044
M34336.m	rs7203112	A	C	0.016	-0.049	0.011	8.41E-06	19.843
M34336.m	rs7256113	A	G	0.7703	-0.0134	0.003	6.38E-06	19.9511
M34336.m	rs7607574	A	G	0.6407	0.0134	0.003	6.54E-06	19.9511
M34336.m	rs7795256	T	C	0.2434	-0.0135	0.003	5.33E-06	20.25
M34336.m	rs893712	T	C	0.7669	-0.0152	0.003	2.99E-07	25.6711
M34336.m	rs894127	A	G	0.1321	0.0208	0.0039	1.23E-07	28.4444
M34336.m	rs9462736	T	C	0.3451	0.0742	0.0162	4.91E-06	20.9787
M34338.m	rs10020998	A	G	0.1116	-0.1357	0.0293	3.69E-06	21.4499
M34338.m	rs10213999	A	C	0.2991	-0.0914	0.0203	6.8E-06	20.2722
M34338.m	rs10408650	T	C	0.0449	0.2407	0.0529	5.25E-06	20.7034
M34338.m	rs10995163	A	C	0.9644	0.3473	0.0764	5.44E-06	20.6644
M34338.m	rs11573590	A	G	0.0204	-0.3896	0.0805	1.32E-06	23.4232
M34338.m	rs11694339	A	G	0.0329	0.2881	0.0643	7.33E-06	20.0754
M34338.m	rs11725533	A	G	0.9605	-0.2122	0.0467	5.43E-06	20.647
M34338.m	rs12071018	T	C	0.2889	0.1843	0.0387	1.96E-06	22.6793
M34338.m	rs12326998	A	C	0.011	-1.038	0.18	8.09E-09	33.2544
M34338.m	rs12525617	T	C	0.0207	-0.257	0.0582	9.93E-06	19.4994
M34338.m	rs12626169	C	G	0.0363	0.2276	0.0464	9.37E-07	24.0607
M34338.m	rs12702615	A	G	0.011	0.892	0.179	6.25E-07	24.8327
M34338.m	rs12769714	T	C	0.011	-1.008	0.18	2.14E-08	31.36
M34338.m	rs13028212	C	G	0.9809	-0.3375	0.0747	6.3E-06	20.413
M34338.m	rs13094553	A	G	0.985	0.975	0.177	3.62E-08	30.3433
M34338.m	rs13121716	A	C	0.9794	-0.3919	0.0822	1.86E-06	22.7304
M34338.m	rs13147512	T	C	0.028	0.787	0.167	2.45E-06	22.2084
M34338.m	rs1618850	T	G	0.6732	-0.0911	0.0197	3.77E-06	21.3848
M34338.m	rs16831763	A	G	0.988	0.44	0.081	5.57E-08	29.5077
M34338.m	rs16896783	A	G	0.989	1.024	0.179	1.06E-08	32.7261
M34338.m	rs16928617	T	C	0.0213	-0.3934	0.0846	3.28E-06	21.6236
M34338.m	rs17415487	A	G	0.989	-0.898	0.179	5.26E-07	25.1679
M34338.m	rs17784966	T	G	0.975	-0.3165	0.0703	6.76E-06	20.2692
M34338.m	rs17796969	T	C	0.9439	0.202	0.038	1.06E-07	28.2576
M34338.m	rs1912320	A	G	0.0355	0.3974	0.0662	1.98E-09	36.0363
M34338.m	rs1954396	A	G	0.9704	-0.2752	0.0574	1.65E-06	22.9865
M34338.m	rs207703	T	C	0.9769	0.2845	0.0561	3.94E-07	25.7181
M34338.m	rs2212841	T	C	0.015	0.87	0.177	8.87E-07	24.1597
M34338.m	rs2390633	A	C	0.0402	-0.2461	0.0513	1.58E-06	23.0138
M34338.m	rs2830801	A	G	0.023	-1.072	0.179	2.11E-09	35.866
M34338.m	rs3734021	A	G	0.052	-0.301	0.067	7.04E-06	20.1829
M34338.m	rs3735210	T	C	0.985	-0.873	0.177	8.13E-07	24.3266
M34338.m	rs3749229	T	C	0.014	0.88	0.18	1.01E-06	23.9012
M34338.m	rs3750318	T	C	0.012	0.895	0.179	5.73E-07	25
M34338.m	rs4976704	T	C	0.989	-0.905	0.181	5.73E-07	25
M34338.m	rs6511851	C	G	0.986	-0.584	0.128	5.06E-06	20.8164
M34338.m	rs693833	A	C	0.983	0.591	0.127	3.26E-06	21.6555
M34338.m	rs7954487	C	G	0.9869	0.4415	0.0939	2.57E-06	22.107
M34338.m	rs8089543	T	C	0.011	-1.029	0.179	9E-09	33.0464
M34338.m	rs8187736	T	C	0.0534	0.2254	0.0481	2.73E-06	21.9593
M34338.m	rs9566930	T	C	0.015	-0.989	0.176	1.92E-08	31.5767
M34338.m	rs9812186	A	G	0.9771	-0.3327	0.0722	4.04E-06	21.234
M34338.m	rs9826397	A	G	0.9782	-0.2895	0.0639	5.8E-06	20.5256
M34339.m	rs10171231	T	C	0.0634	0.1402	0.0292	1.51E-06	23.0532
M34339.m	rs10496053	T	C	0.9228	-0.0906	0.0199	5.42E-06	20.7277

M34339.m	rs1054363	T	G	0.019	0.2377	0.052	4.84E-06	20.8954
M34339.m	rs11871671	T	C	0.2673	0.0537	0.0113	2.18E-06	22.5835
M34339.m	rs1188240	A	C	0.5129	0.0721	0.0154	2.93E-06	21.9194
M34339.m	rs12701171	T	G	0.9455	-0.11	0.0214	2.59E-07	26.4215
M34339.m	rs13052926	A	G	0.8527	-0.0663	0.0149	9.05E-06	19.7995
M34339.m	rs17216158	A	T	0.9738	-0.1713	0.0346	7.13E-07	24.5111
M34339.m	rs17218189	T	C	0.0628	0.1528	0.0342	7.78E-06	19.9616
M34339.m	rs17646069	T	C	0.9899	-0.2932	0.0599	1.01E-06	23.9593
M34339.m	rs3810529	T	C	0.0437	0.1173	0.024	1.04E-06	23.8877
M34339.m	rs610810	T	G	0.988	-0.298	0.064	3.22E-06	21.6807
M34339.m	rs6794734	A	C	0.6977	-0.049	0.0111	9.81E-06	19.4871
M34339.m	rs6837910	A	G	0.7634	0.0522	0.0118	9.5E-06	19.5694
M34339.m	rs749427	A	G	0.0625	0.1032	0.023	7.04E-06	20.1328
M34339.m	rs862029	A	G	0.0214	0.2654	0.051	1.92E-07	27.0808
M34339.m	rs9667032	T	G	0.0244	0.3286	0.0624	1.38E-07	27.731
M34339.m	rs9831047	A	G	0.069	0.0881	0.0198	8.43E-06	19.798
M34344.m	rs10509709	C	G	0.9525	-0.1004	0.0189	1.09E-07	28.2191
M34344.m	rs11778877	T	C	0.9184	-0.063	0.0141	8.41E-06	19.9638
M34344.m	rs12194922	T	C	0.0118	0.5355	0.1186	6.35E-06	20.3868
M34344.m	rs1791914	A	G	0.2847	-0.0566	0.0126	6.63E-06	20.1786
M34344.m	rs226818	A	G	0.9006	0.0564	0.0127	8.29E-06	19.722
M34344.m	rs2513248	T	C	0.083	0.0662	0.0142	3.01E-06	21.734
M34344.m	rs3748400	T	C	0.7691	-0.0396	0.009	9.88E-06	19.36
M34344.m	rs595526	A	G	0.5806	0.0799	0.018	9.39E-06	19.7037
M34344.m	rs7524781	A	G	0.1738	0.1192	0.0225	1.2E-07	28.0664
M34344.m	rs9561023	A	G	0.0941	0.064	0.0139	4.19E-06	21.1997
M34344.m	rs9870766	A	G	0.1247	0.1228	0.0277	9.14E-06	19.6534
M34344.m	rs9971507	A	G	0.0347	0.322	0.0712	6.17E-06	20.4528
M34350.m	rs10839562	C	G	0.8384	-0.0797	0.0162	9.03E-07	24.204
M34350.m	rs10893653	A	G	0.9309	-0.0771	0.0175	9.87E-06	19.4103
M34350.m	rs12592330	T	C	0.8495	-0.1292	0.0285	5.86E-06	20.5511
M34350.m	rs17819012	T	G	0.0876	0.0717	0.0155	3.9E-06	21.3981
M34350.m	rs3093135	A	T	0.8288	0.0569	0.0118	1.29E-06	23.252
M34350.m	rs35620	C	G	0.8372	0.056	0.0118	2.23E-06	22.5223
M34350.m	rs3917538	A	G	0.2133	-0.0586	0.011	9.75E-08	28.3798
M34350.m	rs4001461	T	C	0.4402	0.0513	0.009	1.39E-08	32.49
M34350.m	rs4148343	A	C	0.1539	-0.0535	0.0119	6.45E-06	20.2122
M34350.m	rs4631721	A	G	0.6423	0.043	0.0091	2.49E-06	22.3282
M34350.m	rs516834	T	C	0.6097	0.045	0.0097	3.86E-06	21.5219
M34350.m	rs6793840	A	G	0.5465	0.0411	0.0091	5.72E-06	20.3986
M34350.m	rs7551986	A	T	0.0797	-0.08	0.0173	3.8E-06	21.3839
M34350.m	rs9538657	A	C	0.0228	0.4491	0.0943	1.93E-06	22.681
M34359.m	rs10752061	A	G	0.669	-0.009	0.0018	4.61E-07	25
M34359.m	rs10880751	A	G	0.488	-0.0083	0.0018	2.51E-06	21.2623
M34359.m	rs10898067	T	C	0.6618	0.0079	0.0018	9.83E-06	19.2623
M34359.m	rs11052940	A	G	0.412	-0.0078	0.0018	9.78E-06	18.7778
M34359.m	rs11843600	A	G	0.5105	-0.0082	0.0018	2.85E-06	20.7531
M34359.m	rs12190347	A	G	0.2984	-0.008	0.0018	9.64E-06	19.7531
M34359.m	rs12331965	A	C	0.4969	0.0081	0.0018	4E-06	20.25
M34359.m	rs12516976	C	G	0.9076	0.0155	0.0034	6.47E-06	20.7829
M34359.m	rs13212916	A	G	0.6812	-0.0083	0.0018	2.92E-06	21.2623
M34359.m	rs13298337	T	C	0.6276	0.0096	0.0018	1.05E-07	28.4444
M34359.m	rs13381824	C	G	0.6082	0.0086	0.0018	1.51E-06	22.8272
M34359.m	rs1352907	T	C	0.1679	0.0122	0.0026	4.02E-06	22.0178
M34359.m	rs136230	A	G	0.5971	-0.0081	0.0018	5.83E-06	20.25

M34359.m	rs1478747	A	G	0.214	0.018	0.0037	1.27E-06	23.6669
M34359.m	rs17575455	A	C	0.3094	-0.0091	0.0018	3.57E-07	25.5586
M34359.m	rs1761768	A	G	0.3771	0.0088	0.0018	6.07E-07	23.9012
M34359.m	rs17638873	T	C	0.9074	0.0173	0.0034	2.44E-07	25.8901
M34359.m	rs1905351	T	C	0.3924	0.0093	0.0018	1.38E-07	26.6944
M34359.m	rs1910775	A	G	0.0927	0.0297	0.0065	4.34E-06	20.8779
M34359.m	rs2154355	T	C	0.8541	-0.0116	0.0026	8.1E-06	19.9053
M34359.m	rs2281071	A	G	0.346	0.009	0.002	6.8E-06	20.25
M34359.m	rs2444272	T	C	0.6637	0.008	0.0018	6.87E-06	19.7531
M34359.m	rs2541164	A	G	0.2961	-0.0081	0.0018	6.77E-06	20.25
M34359.m	rs2712549	A	G	0.472	0.0083	0.0018	3.22E-06	21.2623
M34359.m	rs2760379	T	C	0.347	0.0081	0.0018	7.12E-06	20.25
M34359.m	rs2861609	A	G	0.3031	-0.0094	0.0018	1.63E-07	27.2716
M34359.m	rs2881130	A	G	0.3385	-0.0094	0.0018	1.25E-07	27.2716
M34359.m	rs293064	T	C	0.4572	-0.0079	0.0018	8.58E-06	19.2623
M34359.m	rs310325	T	C	0.4417	-0.0083	0.0018	2.68E-06	21.2623
M34359.m	rs3875143	A	C	0.6316	0.009	0.0019	1.76E-06	22.4377
M34359.m	rs4281086	A	C	0.667	0.0082	0.0018	4.77E-06	20.7531
M34359.m	rs4664490	C	G	0.3876	0.0082	0.0018	4.47E-06	20.7531
M34359.m	rs4905134	A	G	0.5097	-0.0096	0.0018	6.15E-08	28.4444
M34359.m	rs4925536	A	G	0.8988	0.0146	0.0032	7.17E-06	20.8164
M34359.m	rs6123329	T	C	0.5275	-0.0079	0.0018	7.65E-06	19.2623
M34359.m	rs6151429	T	C	0.9293	-0.0249	0.0035	5.38E-13	50.6131
M34359.m	rs6507247	A	G	0.4643	-0.0191	0.0038	4.43E-07	25.2639
M34359.m	rs6687408	C	G	0.828	0.0115	0.0025	5.18E-06	21.16
M34359.m	rs6925949	A	G	0.1696	-0.0226	0.0051	8.71E-06	19.6371
M34359.m	rs749514	A	G	0.3055	-0.009	0.0018	4.71E-07	25
M34359.m	rs7763535	A	C	0.7642	0.0121	0.0026	2.06E-06	21.6583
M34359.m	rs8030512	T	C	0.5209	-0.008	0.0018	7E-06	19.7531
M34359.m	rs8071165	T	G	0.0115	-0.1013	0.0222	5.09E-06	20.8215
M34359.m	rs9322898	T	C	0.318	-0.009	0.002	6.8E-06	20.25
M34359.m	rs973634	T	C	0.505	0.0087	0.0018	1E-06	23.3611
M34365.x	rs10071202	A	G	0.4118	0.2107	0.0472	8.02E-06	19.9271
M34365.x	rs10091404	C	G	0.0842	0.2816	0.0635	9.32E-06	19.6661
M34365.x	rs10146370	C	G	0.247	0.1099	0.0226	1.22E-06	23.6471
M34365.x	rs10182315	T	C	0.2222	0.1755	0.0393	8E-06	19.942
M34365.x	rs1019662	T	C	0.8047	-0.1799	0.0395	5.14E-06	20.7428
M34365.x	rs10244572	A	G	0.8206	-0.2133	0.0434	8.89E-07	24.1547
M34365.x	rs10473625	A	C	0.4135	-0.1725	0.0389	9.17E-06	19.6643
M34365.x	rs10787147	T	C	0.7552	-0.0993	0.0222	7.79E-06	20.0075
M34365.x	rs10892025	A	G	0.4724	0.1609	0.0299	7.01E-08	28.9581
M34365.x	rs10920206	T	C	0.9239	-0.3266	0.0727	7.02E-06	20.182
M34365.x	rs11019895	A	G	0.9167	-0.2809	0.0634	9.29E-06	19.6302
M34365.x	rs11180679	A	G	0.3844	0.1652	0.0366	6.31E-06	20.3731
M34365.x	rs1163858	A	G	0.3056	0.1821	0.0386	2.36E-06	22.2559
M34365.x	rs11772501	T	C	0.1441	0.1134	0.0252	6.92E-06	20.25
M34365.x	rs11924743	A	G	0.6176	-0.1786	0.0343	1.89E-07	27.1128
M34365.x	rs11965227	A	C	0.3865	0.0965	0.0187	2.53E-07	26.63
M34365.x	rs12005759	A	G	0.4099	0.0909	0.0197	4.02E-06	21.291
M34365.x	rs12051514	A	G	0.0791	0.1668	0.0344	1.24E-06	23.5112
M34365.x	rs12074764	T	C	0.9521	-0.2212	0.0498	9.08E-06	19.7293
M34365.x	rs12191555	T	C	0.2583	-0.1094	0.0229	1.79E-06	22.8225
M34365.x	rs12221514	T	G	0.9281	-0.1711	0.0358	1.81E-06	22.842
M34365.x	rs12357028	A	G	0.1144	0.1173	0.0255	4.16E-06	21.16
M34365.x	rs12498092	A	G	0.5278	-0.1601	0.03	9.17E-08	28.48

M34365.x	rs12697346	T	C	0.8333	-0.2064	0.0326	2.54E-10	40.0852
M34365.x	rs1284853	A	G	0.0842	0.2815	0.0635	9.26E-06	19.6521
M34365.x	rs132382	A	G	0.9158	-0.2814	0.0635	9.29E-06	19.6382
M34365.x	rs13270412	T	C	0.8578	-0.1222	0.0248	8E-07	24.2795
M34365.x	rs157009	T	C	0.3529	-0.2438	0.0512	1.92E-06	22.674
M34365.x	rs1606973	A	C	0.6944	-0.184	0.038	1.32E-06	23.446
M34365.x	rs1614887	A	G	0.4688	0.1758	0.0359	1.01E-06	23.98
M34365.x	rs17357891	C	G	0.901	-0.1895	0.0377	4.94E-07	25.266
M34365.x	rs17401989	A	G	0.0527	0.2037	0.044	3.66E-06	21.4327
M34365.x	rs17736971	T	G	0.0841	0.2815	0.0635	9.38E-06	19.6521
M34365.x	rs1806807	A	G	0.772	-0.1364	0.0291	2.69E-06	21.9706
M34365.x	rs1941075	T	C	0.7538	-0.1094	0.0239	4.71E-06	20.9526
M34365.x	rs2075384	T	C	0.9158	-0.2814	0.0635	9.29E-06	19.6382
M34365.x	rs2309461	T	C	0.7222	-0.2016	0.0305	3.95E-11	43.6899
M34365.x	rs2402511	A	G	0.3786	0.2034	0.0366	2.82E-08	30.8844
M34365.x	rs2408190	A	G	0.5271	-0.0867	0.0176	7.87E-07	24.2668
M34365.x	rs3743526	C	G	0.8333	-0.1861	0.0395	2.51E-06	22.1972
M34365.x	rs3745836	A	G	0.9167	-0.2809	0.0634	9.29E-06	19.6302
M34365.x	rs3759903	T	G	0.1612	0.1253	0.0269	3.09E-06	21.6969
M34365.x	rs3912204	A	G	0.0833	0.2809	0.0634	9.29E-06	19.6302
M34365.x	rs4282172	A	G	0.4375	-0.1904	0.0403	2.3E-06	22.3215
M34365.x	rs4413146	T	G	0.8985	-0.1504	0.0319	2.39E-06	22.2287
M34365.x	rs4420497	T	C	0.0833	0.2809	0.0634	9.29E-06	19.6302
M34365.x	rs4477518	A	G	0.4979	-0.239	0.0509	2.61E-06	22.0475
M34365.x	rs451979	T	C	0.4193	-0.1967	0.0422	3.14E-06	21.7262
M34365.x	rs4748086	A	G	0.0833	0.2809	0.0634	9.29E-06	19.6302
M34365.x	rs4765467	A	T	0.0842	0.2815	0.0635	9.25E-06	19.6521
M34365.x	rs606242	T	C	0.6853	-0.0934	0.02	3E-06	21.8089
M34365.x	rs6464160	T	G	0.2552	0.1498	0.0278	6.94E-08	29.0358
M34365.x	rs6553660	A	T	0.1075	0.1448	0.0324	7.67E-06	19.9732
M34365.x	rs6773303	A	G	0.4576	0.0923	0.0205	6.47E-06	20.272
M34365.x	rs679622	A	T	0.1193	0.2562	0.0573	7.92E-06	19.9917
M34365.x	rs6830940	T	C	0.75	-0.1767	0.0386	4.67E-06	20.9555
M34365.x	rs7090161	A	C	0.9727	-0.2355	0.0532	9.71E-06	19.5956
M34365.x	rs7463317	A	G	0.108	0.2216	0.0423	1.6E-07	27.4447
M34365.x	rs7678362	T	C	0.6921	-0.1818	0.0395	4.09E-06	21.1833
M34365.x	rs7693137	T	C	0.5556	-0.148	0.0303	1.02E-06	23.8582
M34365.x	rs7721259	T	C	0.1111	0.2104	0.0401	1.53E-07	27.5298
M34365.x	rs7759527	T	C	0.1004	0.212	0.0479	9.46E-06	19.5885
M34365.x	rs7771614	T	C	0.5	-0.162	0.034	1.85E-06	22.7024
M34365.x	rs7900257	T	G	0.3056	0.159	0.0335	2.02E-06	22.5271
M34365.x	rs8105343	A	G	0.8192	-0.2048	0.0459	7.95E-06	19.9083
M34365.x	rs820860	T	C	0.6111	-0.1715	0.0362	2.1E-06	22.4446
M34365.x	rs9815685	A	G	0.1775	0.1928	0.0392	8.95E-07	24.1903
M34365.x	rs9864332	A	C	0.1111	0.254	0.0562	6.16E-06	20.4265
M34384.m	rs17630756	A	G	0.0279	0.1768	0.0391	5.99E-06	20.4461
M34384.m	rs2688892	T	C	0.2396	0.0688	0.015	4.47E-06	21.0375
M34384.m	rs3741793	C	G	0.2267	-0.0684	0.0154	9.22E-06	19.7274
M34384.m	rs4708385	A	G	0.3704	-0.0608	0.0133	5.2E-06	20.898
M34384.m	rs7975440	T	C	0.1425	-0.0885	0.0187	2.31E-06	22.3977
M34384.m	rs9319364	T	C	0.3038	0.0919	0.0207	8.75E-06	19.7102
M34384.m	rs9443691	A	G	0.1464	-0.0809	0.0182	8.68E-06	19.7585
M34384.x	rs17105180	A	C	0.9297	0.0895	0.0187	1.71E-06	22.9067
M34384.x	rs2016612	T	C	0.5694	-0.0451	0.0099	5.34E-06	20.7531
M34384.x	rs3762968	A	C	0.9507	0.1001	0.0224	7.97E-06	19.9697

M34384.x	rs7666233	A	G	0.8801	0.087	0.0193	6.39E-06	20.32
M34384.x	rs7936649	T	G	0.2825	0.0579	0.0105	3.14E-08	30.4073
M34384.x	rs7965333	C	G	0.0735	-0.0863	0.0191	6.12E-06	20.4153
M34389.x	rs11129047	A	C	0.7309	0.0205	0.0045	6.28E-06	20.7531
M34389.x	rs11664183	A	G	0.8302	-0.0244	0.0054	6.66E-06	20.417
M34389.x	rs1445437	A	G	0.4167	0.0199	0.0045	7.87E-06	19.556
M34389.x	rs1587148	C	G	0.9124	0.0504	0.0083	1.55E-09	36.8727
M34389.x	rs2151358	A	G	0.7211	-0.02	0.0045	9.47E-06	19.7531
M34389.x	rs332176	A	G	0.7408	-0.0215	0.0046	2.52E-06	21.8455
M34389.x	rs3825895	T	C	0.4752	0.0207	0.0046	8.32E-06	20.25
M34389.x	rs4921914	T	C	0.7848	0.0879	0.0054	1.09E-60	264.966
M34389.x	rs494075	A	C	0.4921	-0.0201	0.0045	8.48E-06	19.9511
M34389.x	rs6803606	T	C	0.7239	-0.0216	0.0045	2.06E-06	23.04
M34389.x	rs704852	A	C	0.758	-0.023	0.005	4.23E-06	21.16
M34389.x	rs7844866	A	G	0.945	0.0657	0.01	4.72E-11	43.1649
M34389.x	rs7905007	T	G	0.1012	-0.0317	0.0071	7.27E-06	19.9343
M34389.x	rs9657488	T	C	0.3734	-0.0241	0.0045	1.01E-07	28.682
M34389.x	rs9837363	T	C	0.4628	-0.0206	0.0045	3.6E-06	20.956
M34389.x	rs9910557	A	G	0.4874	-0.0215	0.0045	1.62E-06	22.8272
M34390.x	rs12711494	A	T	0.2932	-0.0265	0.006	9.88E-06	19.5069
M34390.x	rs12777985	T	C	0.2212	-0.0291	0.0061	2.14E-06	22.7576
M34390.x	rs1820124	A	G	0.2432	0.0275	0.0061	5.59E-06	20.3238
M34390.x	rs1892651	A	G	0.4236	0.0496	0.0105	2.4E-06	22.3144
M34390.x	rs2163783	T	C	0.2984	-0.03	0.0054	2.98E-08	30.8642
M34390.x	rs4331333	T	C	0.6194	-0.0257	0.0053	1.16E-06	23.5133
M34390.x	rs4805131	A	G	0.7825	-0.028	0.0062	5.64E-06	20.3954
M34390.x	rs8069788	T	C	0.4918	-0.0486	0.0106	4.25E-06	21.0214
M34390.x	rs9305620	A	G	0.3537	-0.0487	0.0107	5.52E-06	20.7153
M34390.x	rs999445	T	C	0.1044	0.0354	0.008	9.1E-06	19.5806
M34395.x	rs10244178	A	G	0.6552	0.0204	0.0044	3.73E-06	21.4959
M34395.x	rs10492511	T	C	0.1563	-0.0238	0.0053	7.65E-06	20.1652
M34395.x	rs10738265	T	C	0.1757	0.0261	0.0053	8.02E-07	24.251
M34395.x	rs12176630	A	C	0.0505	0.0413	0.0093	8.22E-06	19.7212
M34395.x	rs13263584	A	T	0.9529	0.0577	0.0097	2.75E-09	35.3841
M34395.x	rs1495741	A	G	0.7841	0.057	0.0052	1.04E-27	120.155
M34395.x	rs16881070	T	G	0.7582	0.0198	0.0045	8.84E-06	19.36
M34395.x	rs17004392	A	C	0.0314	0.111	0.0245	5.73E-06	20.5264
M34395.x	rs202534	A	G	0.5576	-0.042	0.0083	3.65E-07	25.606
M34395.x	rs3852445	C	G	0.1284	0.0284	0.0061	2.95E-06	21.6759
M34395.x	rs4244070	T	C	0.8314	0.0502	0.0112	7.77E-06	20.0896
M34395.x	rs4356633	A	C	0.1592	-0.0249	0.0053	2.73E-06	22.0723
M34395.x	rs7705239	A	G	0.8521	0.0254	0.0054	2.3E-06	22.1248
M34395.x	rs7801805	A	C	0.7322	0.0199	0.0045	8.15E-06	19.556
M34395.x	rs7973154	C	G	0.0265	-0.0595	0.0131	5.31E-06	20.6296
M34395.x	rs8043508	A	G	0.9848	-0.0773	0.017	5.38E-06	20.6757
M34395.x	rs9846511	A	C	0.2559	-0.0205	0.0045	4.25E-06	20.7531
M34395.x	rs9907459	T	C	0.9803	-0.2136	0.0453	2.38E-06	22.2334
M34400.x	rs10959207	T	C	0.814	-0.0265	0.006	9.39E-06	19.5069
M34400.x	rs11586387	A	T	0.9458	0.1381	0.0312	9.46E-06	19.592
M34400.x	rs12505728	T	C	0.1409	-0.0917	0.0185	7.38E-07	24.5694
M34400.x	rs1395489	A	G	0.1479	-0.0564	0.0125	6.88E-06	20.3581
M34400.x	rs3763790	T	G	0.1012	0.0317	0.0071	8.91E-06	19.9343
M34400.x	rs4358841	A	G	0.9839	0.079	0.0169	3E-06	21.8515
M34400.x	rs4398579	A	C	0.3581	-0.023	0.0051	7.53E-06	20.3383
M34400.x	rs4724785	T	C	0.6803	0.0198	0.0045	9.59E-06	19.36

M34400.x	rs4780314	C	G	0.7909	-0.0245	0.0054	5.05E-06	20.5847
M34400.x	rs624649	T	G	0.2058	-0.0237	0.0053	8.84E-06	19.9961
M34400.x	rs739163	T	C	0.3656	0.0262	0.0053	6.09E-07	24.4372
M34400.x	rs9333273	A	G	0.0869	0.0554	0.0119	3.54E-06	21.6733
M34404.x	rs10916110	T	C	0.8253	-0.1021	0.0222	4.24E-06	21.1517
M34404.x	rs10955109	A	C	0.4839	-0.0717	0.0157	5.14E-06	20.8564
M34404.x	rs11885739	A	T	0.6877	0.0824	0.018	4.69E-06	20.956
M34404.x	rs11997201	C	G	0.3709	-0.0807	0.0166	1.13E-06	23.6337
M34404.x	rs1944577	T	C	0.2625	-0.0733	0.0135	5.42E-08	29.4809
M34404.x	rs3913646	T	C	0.9822	-0.2774	0.0593	2.94E-06	21.8828
M34404.x	rs6911611	A	T	0.9734	0.1861	0.0405	4.25E-06	21.1146
M34404.x	rs7042159	A	C	0.2478	0.0683	0.0143	1.71E-06	22.8123
M34404.x	rs9415646	T	G	0.691	-0.088	0.0191	4E-06	21.2275
M34404.x	rs9945857	A	G	0.0947	0.1688	0.0321	1.51E-07	27.6525
M34407.m	rs10520079	A	G	0.3469	-0.0167	0.0034	1.36E-06	24.1254
M34407.m	rs10889028	A	G	0.4193	-0.0135	0.0027	7.12E-07	25
M34407.m	rs10947808	T	C	0.8105	-0.0174	0.0036	1.21E-06	23.3611
M34407.m	rs11051552	A	G	0.5217	-0.0121	0.0027	8.47E-06	20.0837
M34407.m	rs11950562	A	C	0.5231	0.0362	0.0027	2.31E-41	179.759
M34407.m	rs12494255	A	G	0.7684	0.0308	0.0066	2.72E-06	21.7778
M34407.m	rs13075137	T	C	0.4448	-0.0242	0.0054	7.51E-06	20.0837
M34407.m	rs16874160	A	G	0.9858	0.137	0.0272	4.49E-07	25.369
M34407.m	rs17672041	T	C	0.1198	0.0298	0.0044	1.31E-11	45.8698
M34407.m	rs2069812	A	G	0.2933	-0.0176	0.0034	1.7E-07	26.7958
M34407.m	rs2291449	A	G	0.9135	-0.0288	0.0052	2.35E-08	30.6746
M34407.m	rs2662314	T	C	0.1347	0.0301	0.0044	4.86E-12	46.798
M34407.m	rs2852371	A	G	0.5831	0.0126	0.0027	3.97E-06	21.7778
M34407.m	rs2963485	T	C	0.1869	-0.0175	0.0036	1.06E-06	23.6304
M34407.m	rs383911	A	G	0.5551	-0.0126	0.0027	2.98E-06	21.7778
M34407.m	rs4463295	A	G	0.5733	0.013	0.0027	1.6E-06	23.1824
M34407.m	rs4505564	T	C	0.7913	0.0163	0.0036	4.94E-06	20.5008
M34407.m	rs4706020	A	G	0.3834	-0.0214	0.0034	3.8E-10	39.6159
M34407.m	rs6596022	T	C	0.964	0.0454	0.0078	6.82E-09	33.8784
M34407.m	rs7106005	T	C	0.8158	0.0204	0.0041	7.66E-07	24.7567
M34407.m	rs7668227	A	G	0.9017	0.0238	0.0053	7.86E-06	20.1652
M34407.m	rs7711551	T	C	0.9689	0.0484	0.0089	5.19E-08	29.574
M34407.m	rs8007846	A	G	0.5509	0.0122	0.0027	8.39E-06	20.417
M34407.m	rs891404	A	G	0.4842	0.0122	0.0027	7.49E-06	20.417
M34407.m	rs9635324	A	G	0.6075	0.0337	0.0027	1.89E-35	155.787
M34407.m	rs9775485	T	C	0.5852	-0.0144	0.0028	2.01E-07	26.449
M34409.m	rs1188542	T	C	0.603	-0.0154	0.0033	3.2E-06	21.7778
M34409.m	rs1790796	T	C	0.8393	0.0192	0.0043	8.52E-06	19.9373
M34409.m	rs212051	T	C	0.2796	-0.0176	0.0034	3.08E-07	26.7958
M34409.m	rs254488	A	G	0.1157	-0.0229	0.005	4.41E-06	20.9764
M34409.m	rs272881	A	G	0.617	-0.0214	0.0033	1.11E-10	42.0533
M34409.m	rs7015612	A	G	0.4132	0.015	0.0034	8.17E-06	19.4637
M34416.m	rs10429330	T	C	0.2704	0.0329	0.0067	9.14E-07	24.1125
M34416.m	rs10995782	T	C	0.9786	-0.0915	0.0201	5.21E-06	20.7229
M34416.m	rs11106512	C	G	0.3694	0.0302	0.0068	9.06E-06	19.724
M34416.m	rs11250064	A	C	0.3131	-0.0168	0.0036	2.47E-06	21.7778
M34416.m	rs12219186	T	C	0.9516	0.0407	0.0087	2.67E-06	21.8852
M34416.m	rs1260326	T	C	0.4087	0.0167	0.0034	1.15E-06	24.1254
M34416.m	rs12981207	A	C	0.0814	0.0496	0.011	6.16E-06	20.3319
M34416.m	rs1522209	T	G	0.3257	0.0159	0.0036	8.28E-06	19.5069
M34416.m	rs1532085	A	G	0.3796	0.0178	0.0035	4.02E-07	25.8645

M34416.m	rs2043422	A	G	0.2928	0.0165	0.0036	4.06E-06	21.0069
M34416.m	rs588136	T	C	0.7961	-0.0293	0.0043	8.3E-12	46.43
M34416.m	rs6036745	A	G	0.4659	-0.0166	0.0034	1.51E-06	23.8374
M34416.m	rs6466344	C	G	0.2679	-0.0173	0.0036	1.22E-06	23.0934
M34416.m	rs6990912	A	C	0.3602	-0.016	0.0035	4.81E-06	20.898
M34419.m	rs1073744	T	G	0.2369	0.0127	0.0026	1.63E-06	23.8595
M34419.m	rs11729080	A	G	0.1591	-0.0128	0.0028	3.43E-06	20.898
M34419.m	rs11981474	A	C	0.824	-0.0286	0.0059	1.48E-06	23.4978
M34419.m	rs12448314	A	G	0.9776	0.0346	0.0077	6.99E-06	20.1916
M34419.m	rs12745827	T	G	0.8446	0.0128	0.0028	4.76E-06	20.898
M34419.m	rs17300762	A	G	0.94	0.022	0.0044	7.23E-07	25
M34419.m	rs174548	C	G	0.6987	-0.0134	0.0026	2.04E-07	26.5621
M34419.m	rs17794487	T	C	0.1987	-0.0127	0.0027	2.29E-06	22.1248
M34419.m	rs1979024	T	C	0.8685	0.017	0.0034	6.92E-07	25
M34419.m	rs2966481	T	C	0.4801	-0.02	0.0045	8.25E-06	19.7531
M34419.m	rs4789188	A	C	0.1628	0.0134	0.0027	5.66E-07	24.631
M34419.m	rs6791775	C	G	0.9835	0.0888	0.0201	9.54E-06	19.5179
M34419.m	rs6997983	A	G	0.0916	-0.0161	0.0036	6.15E-06	20.0008
M34419.m	rs8085108	T	C	0.1945	-0.0135	0.0027	7.86E-07	25
M34419.m	rs980142	A	G	0.1607	-0.0153	0.0034	6.21E-06	20.25
M34420.m	rs10074559	A	G	0.4717	-0.0492	0.0107	4.54E-06	21.1428
M34420.m	rs10144441	A	G	0.7294	-0.0488	0.0109	7.92E-06	20.0441
M34420.m	rs11082534	A	T	0.9001	-0.0759	0.0171	8.65E-06	19.7011
M34420.m	rs11751412	T	G	0.7454	-0.0508	0.0114	8.26E-06	19.8572
M34420.m	rs12083531	T	C	0.2656	0.0527	0.0117	6.82E-06	20.2885
M34420.m	rs12511874	T	C	0.7088	0.0636	0.0109	4.86E-09	34.0456
M34420.m	rs12996938	A	G	0.0536	0.1606	0.0356	6.57E-06	20.3512
M34420.m	rs13133050	A	C	0.3229	-0.0521	0.011	2.06E-06	22.4331
M34420.m	rs17088876	A	G	0.9182	0.0856	0.018	2.06E-06	22.6153
M34420.m	rs17163774	T	G	0.6204	0.0437	0.0098	7.66E-06	19.8843
M34420.m	rs17346455	T	C	0.9542	-0.1344	0.0278	1.3E-06	23.3727
M34420.m	rs2390196	A	G	0.1233	-0.0699	0.0149	2.94E-06	22.0081
M34420.m	rs2518028	T	C	0.3114	0.0655	0.0147	8.36E-06	19.854
M34420.m	rs2731672	T	C	0.257	-0.169	0.017	2.76E-23	98.827
M34420.m	rs2803054	A	C	0.5973	-0.0457	0.0099	4.23E-06	21.3089
M34420.m	rs2938675	A	C	0.3923	0.0476	0.0097	9.79E-07	24.0808
M34420.m	rs3822056	T	G	0.1064	0.0757	0.0162	2.84E-06	21.8354
M34420.m	rs4253243	T	C	0.9103	0.1107	0.0175	2.35E-10	40.0147
M34420.m	rs4253311	A	G	0.4869	-0.1412	0.0097	3.56E-48	211.898
M34420.m	rs4743627	A	T	0.896	0.0804	0.0175	4.6E-06	21.1075
M34420.m	rs4899048	A	G	0.0941	-0.0959	0.018	9.37E-08	28.3852
M34420.m	rs5030062	A	C	0.6147	-0.0718	0.01	5.94E-13	51.5524
M34420.m	rs6943248	A	T	0.0297	0.1334	0.0298	7.58E-06	20.0391
M34420.m	rs754739	A	C	0.2707	-0.053	0.0113	2.79E-06	21.9986
M34420.m	rs7732930	T	C	0.1463	-0.0734	0.0142	2.29E-07	26.7187
M34420.m	rs9394200	T	C	0.0244	0.1613	0.0338	1.88E-06	22.7738
M34420.m	rs961928	T	C	0.3628	0.0487	0.0101	1.58E-06	23.2496
M34441.m	rs10900892	A	T	0.3431	0.0469	0.0096	1.12E-06	23.8673
M34441.m	rs11153306	A	G	0.4827	-0.0194	0.0044	8.83E-06	19.4401
M34441.m	rs11683662	A	G	0.6195	0.0202	0.0044	4.38E-06	21.0764
M34441.m	rs12311571	T	C	0.8478	-0.0287	0.0062	3.26E-06	21.4279
M34441.m	rs13233654	A	G	0.9528	0.0485	0.0107	5.67E-06	20.5455
M34441.m	rs1327014	A	G	0.8126	-0.0243	0.0055	8.81E-06	19.5203
M34441.m	rs1566888	T	C	0.8498	0.0362	0.0082	9.86E-06	19.489
M34441.m	rs1702447	A	G	0.2904	0.022	0.0045	8.34E-07	23.9012

M34441.m	rs17033081	T	C	0.3485	-0.0484	0.0094	3.02E-07	26.5115
M34441.m	rs2850377	T	C	0.41	0.02	0.0044	5.52E-06	20.6612
M34441.m	rs2903269	T	C	0.2157	-0.0237	0.0053	7.66E-06	19.9961
M34441.m	rs640631	T	C	0.904	0.0335	0.0073	4.42E-06	21.0593
M34441.m	rs6824135	T	C	0.1356	-0.0601	0.0131	4.8E-06	21.0478
M34441.m	rs7074531	T	C	0.6964	0.0249	0.0052	1.43E-06	22.9294
M34441.m	rs802424	C	G	0.3093	-0.048	0.01	1.52E-06	23.04
M34441.m	rs895397	A	G	0.3465	-0.0196	0.0044	8.51E-06	19.843
M34453.m	rs10483129	A	G	0.0205	0.0346	0.0069	5.61E-07	25.1451
M34453.m	rs6429539	A	G	0.0308	-0.0261	0.0056	3.54E-06	21.7223
M34453.m	rs812573	A	G	0.2035	-0.0056	0.0013	8.66E-06	18.5562
M34456.m	rs10793993	T	G	0.6284	-0.0129	0.0027	2.55E-06	22.8272
M34456.m	rs12593036	A	G	0.6948	0.0123	0.0028	9.02E-06	19.2972
M34456.m	rs1333111	A	G	0.3146	-0.0136	0.0028	9.84E-07	23.5918
M34456.m	rs1372856	A	G	0.3629	-0.0124	0.0028	6.91E-06	19.6122
M34456.m	rs1506682	A	C	0.1331	0.0416	0.009	3.87E-06	21.3649
M34456.m	rs165582	A	G	0.1156	0.0234	0.0046	3.31E-07	25.8771
M34456.m	rs17202087	A	G	0.8307	0.0165	0.0037	6.16E-06	19.8868
M34456.m	rs1800600	A	G	0.6929	0.0128	0.0028	4.06E-06	20.898
M34456.m	rs2867882	A	G	0.4688	0.0123	0.0028	7.31E-06	19.2972
M34456.m	rs358374	A	G	0.1773	0.0169	0.0037	4.96E-06	20.8627
M34456.m	rs3800552	A	T	0.3513	-0.0128	0.0028	3.3E-06	20.898
M34456.m	rs4505228	T	C	0.8488	-0.0172	0.0037	3.82E-06	21.6099
M34456.m	rs4918082	A	G	0.7174	-0.0123	0.0028	8.83E-06	19.2972
M34456.m	rs4977436	T	C	0.3913	-0.0125	0.0028	6.77E-06	19.9298
M34456.m	rs6049700	T	G	0.2893	-0.0141	0.0028	4.95E-07	25.3584
M34456.m	rs6430457	A	C	0.6797	-0.0128	0.0028	3.82E-06	20.898
M34456.m	rs6785727	A	G	0.5467	0.0126	0.0027	4.52E-06	21.7778
M34456.m	rs6820313	T	G	0.5168	0.0121	0.0027	9.88E-06	20.0837
M34456.m	rs6964878	A	T	0.6693	0.0123	0.0028	8.87E-06	19.2972
M34456.m	rs715	T	C	0.7162	0.0158	0.0035	6.57E-06	20.3788
M34456.m	rs7215587	T	G	0.1312	0.0214	0.0045	1.55E-06	22.6153
M34456.m	rs7924532	A	T	0.6705	-0.013	0.0028	2.49E-06	21.5561
M34456.m	rs7961249	T	C	0.1505	-0.0419	0.0092	5.18E-06	20.7421
M34456.m	rs8002491	A	G	0.8713	-0.02	0.0045	7.8E-06	19.7531
M34456.m	rs918174	T	C	0.687	-0.0142	0.0028	5.61E-07	25.7194
M34456.m	rs9696823	A	G	0.6502	-0.0127	0.0028	4.13E-06	20.5727
M34469.m	rs10275746	T	C	0.4335	0.0191	0.0042	4.95E-06	20.6808
M34469.m	rs10756216	T	C	0.2358	0.0268	0.005	6.63E-08	28.7296
M34469.m	rs11905798	A	G	0.0405	-0.0997	0.0212	2.5E-06	22.1166
M34469.m	rs1195892	T	C	0.5662	-0.0188	0.0042	6.16E-06	20.0363
M34469.m	rs1235385	T	C	0.167	0.0257	0.0057	5.66E-06	20.329
M34469.m	rs12580426	A	G	0.9345	0.0431	0.0083	2.2E-07	26.9649
M34469.m	rs12810144	T	C	0.079	0.0754	0.0162	3.35E-06	21.6627
M34469.m	rs133565	A	G	0.3318	-0.0189	0.0042	8.51E-06	20.25
M34469.m	rs4630827	T	C	0.201	0.0226	0.005	7.34E-06	20.4304
M34469.m	rs8086010	A	C	0.6663	-0.0192	0.0042	5.48E-06	20.898
M34469.m	rs9635567	T	C	0.7787	0.0227	0.005	5.73E-06	20.6116
M34481.m	rs10945673	T	C	0.182	-0.0276	0.0043	1.45E-10	41.1985
M34481.m	rs10993993	T	C	0.9702	-0.0469	0.0095	8E-07	24.3724
M34481.m	rs11123838	A	G	0.24	-0.0168	0.0037	4.24E-06	20.6165
M34481.m	rs1171615	T	C	0.7791	-0.0476	0.0039	3.19E-34	148.965
M34481.m	rs1171650	T	C	0.2361	-0.0228	0.0036	2.1E-10	40.1111
M34481.m	rs1173242	T	C	0.7205	-0.0163	0.0036	5.11E-06	20.5008
M34481.m	rs12238759	T	C	0.0429	0.036	0.0079	5.12E-06	20.7659

M34481.m	rs12527129	A	C	0.9801	0.0607	0.0131	3.83E-06	21.4701
M34481.m	rs12527129	A	C	0.9801	0.0607	0.0131	3.83E-06	21.4701
M34481.m	rs13220412	C	G	0.0415	-0.0585	0.0085	6.15E-12	47.3668
M34481.m	rs1367211	T	C	0.2698	-0.0273	0.0035	1.39E-14	60.84
M34481.m	rs1641387	T	C	0.7786	0.0355	0.0077	4.23E-06	21.2557
M34481.m	rs17588242	T	C	0.7801	-0.0311	0.0059	1.26E-07	27.7854
M34481.m	rs2453573	T	C	0.8177	-0.0202	0.0044	3.68E-06	21.0764
M34481.m	rs316020	A	G	0.0937	-0.1799	0.0053	1E-200	1152.15
M34481.m	rs316032	T	C	0.6143	-0.0247	0.0034	6.9E-13	52.776
M34481.m	rs316174	A	G	0.4819	-0.0314	0.0034	7.02E-20	85.2907
M34481.m	rs3799344	T	C	0.4519	0.0197	0.0034	8.94E-09	33.5718
M34481.m	rs3912161	T	C	0.9396	-0.035	0.0071	9.26E-07	24.3007
M34481.m	rs4948238	A	G	0.7019	-0.0182	0.0035	2.26E-07	27.04
M34481.m	rs532440	T	G	0.2927	0.0237	0.0035	1.93E-11	45.8522
M34481.m	rs628031	A	G	0.4122	0.0203	0.0035	6.19E-09	33.64
M34481.m	rs743263	A	G	0.3225	0.0294	0.0066	8.01E-06	19.843
M34481.m	rs7761031	A	G	0.96	-0.0406	0.0081	5.87E-07	25.1236
M34481.m	rs791174	T	G	0.2498	-0.0388	0.0036	4.69E-27	116.16
M34481.m	rs9456505	A	G	0.0665	-0.0445	0.0062	7.5E-13	51.5153
M34499.m	rs11822948	T	C	0.8079	0.0477	0.0105	5.69E-06	20.6376
M34499.m	rs12443081	A	G	0.0904	-0.0726	0.0147	7.92E-07	24.3915
M34499.m	rs17198824	A	G	0.9879	-0.3408	0.0698	1.06E-06	23.839
M34499.m	rs1936458	A	G	0.3221	-0.0393	0.0089	9.97E-06	19.4987
M34499.m	rs2984639	A	G	0.7403	-0.0397	0.0089	8.6E-06	19.8976
M34499.m	rs34170	T	C	0.9477	0.1342	0.0284	2.32E-06	22.329
M34499.m	rs7152969	A	G	0.2096	0.0431	0.0097	8.01E-06	19.7429
M34513.m	rs10024907	A	C	0.1598	0.0377	0.0078	1.3E-06	23.3611
M34513.m	rs10836652	A	G	0.2061	0.0319	0.0069	4.41E-06	21.3739
M34513.m	rs11044220	C	G	0.6326	-0.0274	0.006	5.49E-06	20.8544
M34513.m	rs1234318	A	G	0.645	0.037	0.008	3.75E-06	21.3906
M34513.m	rs12419430	T	C	0.3944	-0.0277	0.006	4.05E-06	21.3136
M34513.m	rs12521113	T	G	0.1521	0.0374	0.0078	1.46E-06	22.9908
M34513.m	rs12882240	A	G	0.05	0.0597	0.0134	8.54E-06	19.849
M34513.m	rs2018451	A	G	0.284	-0.0561	0.0127	9.28E-06	19.5127
M34513.m	rs2816252	T	C	0.066	0.082	0.017	1.41E-06	23.2664
M34513.m	rs2974445	T	C	0.5529	0.0279	0.006	3.11E-06	21.6225
M34513.m	rs317894	A	G	0.3357	-0.0281	0.0061	3.45E-06	21.2204
M34513.m	rs4901004	T	C	0.8716	-0.0385	0.0086	7.99E-06	20.0412
M34513.m	rs8058752	T	G	0.9762	-0.1604	0.0361	8.88E-06	19.7421
M34516.m	rs10179884	T	C	0.168	0.2058	0.0412	5.9E-07	24.9515
M34516.m	rs1045530	C	G	0.6148	0.0969	0.0218	8.79E-06	19.7576
M34516.m	rs10463012	A	G	0.9883	-0.796	0.1506	1.24E-07	27.9368
M34516.m	rs11171791	T	C	0.957	0.3447	0.0761	5.86E-06	20.517
M34516.m	rs11686391	T	C	0.8106	-0.1328	0.0285	3.27E-06	21.7123
M34516.m	rs11893356	A	G	0.0161	0.5065	0.1086	3.08E-06	21.752
M34516.m	rs11947369	T	C	0.727	-0.1138	0.0253	7.07E-06	20.2322
M34516.m	rs12441816	A	T	0.0435	0.2952	0.061	1.31E-06	23.4193
M34516.m	rs1396287	A	G	0.8863	-0.1717	0.0382	6.95E-06	20.2029
M34516.m	rs1504269	A	T	0.9766	-0.4943	0.1107	7.96E-06	19.9382
M34516.m	rs1546239	A	T	0.6914	-0.1774	0.0398	8.26E-06	19.8674
M34516.m	rs16955210	A	G	0.9818	-0.5039	0.1088	3.66E-06	21.4502
M34516.m	rs17083481	T	C	0.2175	-0.1292	0.0279	3.63E-06	21.4445
M34516.m	rs6111973	T	C	0.2105	0.1379	0.0288	1.65E-06	22.9268
M34516.m	rs6541298	A	G	0.0117	0.8157	0.1497	5.09E-08	29.6905
M34516.m	rs7549636	A	G	0.271	0.1145	0.0254	6.36E-06	20.3209

M34516.m	rs7655071	C	G	0.1519	0.1543	0.0316	1.04E-06	23.8428
M34516.m	rs907609	T	C	0.1354	0.1592	0.0352	6.18E-06	20.4551
M34527.m	rs10790819	C	G	0.0904	-0.0255	0.0058	9.77E-06	19.3297
M34527.m	rs10797373	A	G	0.6912	-0.0148	0.0033	8.16E-06	20.1139
M34527.m	rs11040799	A	T	0.7347	0.0159	0.0034	2.73E-06	21.8694
M34527.m	rs11666603	T	C	0.2406	0.0234	0.004	6.96E-09	34.2225
M34527.m	rs12456616	T	G	0.9367	-0.032	0.0072	9.96E-06	19.7531
M34527.m	rs13264997	A	G	0.2032	-0.0293	0.0063	3.46E-06	21.6299
M34527.m	rs17614939	A	G	0.782	0.0204	0.0035	5.67E-09	33.9722
M34527.m	rs1939769	A	G	0.0643	0.0333	0.0064	2.1E-07	27.0725
M34527.m	rs2035647	A	G	0.2052	-0.0292	0.0034	2.18E-17	73.7578
M34527.m	rs2076196	A	G	0.1888	-0.0162	0.0036	6.46E-06	20.25
M34527.m	rs2609167	T	C	0.6694	-0.0147	0.0033	9.64E-06	19.843
M34527.m	rs322906	T	G	0.4573	-0.0235	0.0052	5.84E-06	20.4234
M34527.m	rs430200	T	G	0.7744	0.0201	0.0035	6.45E-09	32.9804
M34527.m	rs4953640	T	C	0.1512	0.0189	0.0041	4.16E-06	21.2499
M34527.m	rs6062634	A	C	0.4048	-0.0154	0.0034	7.16E-06	20.5156
M34527.m	rs659599	A	G	0.5251	0.0117	0.0026	9.44E-06	20.25
M34527.m	rs903446	A	G	0.5714	-0.0124	0.0027	3.29E-06	21.0919
M34530.m	rs10074057	A	G	0.8297	-0.0373	0.0082	5.24E-06	20.6914
M34530.m	rs10829406	A	G	0.1231	0.0434	0.0092	2.32E-06	22.2538
M34530.m	rs10887481	T	C	0.0266	0.2627	0.0581	6.06E-06	20.4441
M34530.m	rs11577980	T	C	0.0809	0.0503	0.0114	9.86E-06	19.4682
M34530.m	rs11946836	T	C	0.0115	0.2917	0.0653	8.07E-06	19.9548
M34530.m	rs11962139	A	G	0.037	0.0753	0.0155	1.12E-06	23.6008
M34530.m	rs16991685	A	T	0.0738	0.0494	0.0111	9.34E-06	19.8065
M34530.m	rs17287098	A	G	0.1496	0.0375	0.0084	7.15E-06	19.9298
M34530.m	rs7733505	A	C	0.0163	0.2162	0.0485	8.31E-06	19.8714
M34530.m	rs9277380	C	G	0.7148	0.0631	0.014	6.78E-06	20.3143
M34533.m	rs10505869	T	G	0.665	-0.0288	0.0054	9.92E-08	28.4444
M34533.m	rs10741653	A	C	0.5909	-0.0239	0.0054	9.27E-06	19.5888
M34533.m	rs11019976	C	G	0.0717	0.1331	0.0234	1.36E-08	32.3537
M34533.m	rs11190334	A	G	0.035	-0.065	0.0146	8.42E-06	19.8208
M34533.m	rs12026640	A	C	0.9001	-0.0392	0.0089	9.54E-06	19.3996
M34533.m	rs1351284	A	C	0.6287	0.0254	0.0054	2.63E-06	22.1248
M34533.m	rs1473500	T	C	0.0202	-0.0891	0.0189	2.36E-06	22.2245
M34533.m	rs2218275	A	T	0.9535	0.0582	0.0129	6.6E-06	20.3548
M34533.m	rs2243133	T	C	0.018	-0.0968	0.0219	9.98E-06	19.5372
M34533.m	rs2326675	A	C	0.7231	0.0284	0.0063	6.39E-06	20.3215
M34533.m	rs2429426	A	G	0.7093	0.025	0.0055	4.7E-06	20.6612
M34533.m	rs2547231	A	C	0.8319	0.059	0.0072	3.52E-16	67.1489
M34533.m	rs279941	A	C	0.1171	-0.0478	0.0081	4.31E-09	34.8246
M34533.m	rs4764937	T	G	0.6365	-0.0246	0.0054	5.87E-06	20.7531
M34533.m	rs4802397	T	C	0.8551	-0.0365	0.0075	1.2E-06	23.6844
M34533.m	rs9324832	T	C	0.0964	-0.0395	0.0089	9.32E-06	19.6976
M34534.m	rs10979297	A	T	0.6724	-0.0233	0.0052	6.86E-06	20.0773
M34534.m	rs12902624	C	G	0.8789	0.0332	0.0072	4.27E-06	21.2623
M34534.m	rs12928583	A	G	0.544	0.0242	0.0055	8.98E-06	19.36
M34534.m	rs1385234	A	T	0.3159	0.022	0.0049	7E-06	20.1583
M34534.m	rs16966673	T	C	0.0263	0.115	0.0257	7.89E-06	20.023
M34534.m	rs274559	A	G	0.6171	-0.0233	0.0048	1.5E-06	23.5629
M34534.m	rs4140469	A	G	0.7605	-0.025	0.0056	8.98E-06	19.9298
M34534.m	rs4690909	C	G	0.6902	0.0301	0.0049	1.06E-09	37.7347
M34534.m	rs4836309	T	C	0.5412	-0.0225	0.0048	2.38E-06	21.9727
M34534.m	rs4900991	C	G	0.5971	0.0212	0.0048	9.88E-06	19.5069

M34534.m	rs7162018	A	G	0.4943	-0.0219	0.0048	4.54E-06	20.8164
M34534.m	rs943087	T	C	0.1704	0.0306	0.0063	1.4E-06	23.5918
M34534.m	rs9880529	T	C	0.8725	0.0327	0.0071	3.84E-06	21.2119
M34535.m	rs10801685	T	G	0.0209	-0.4744	0.1045	5.67E-06	20.609
M34535.m	rs11070217	T	C	0.4394	0.0435	0.0091	1.55E-06	22.8505
M34535.m	rs11743823	T	C	0.2041	0.0545	0.0114	1.64E-06	22.8551
M34535.m	rs2941589	A	G	0.8527	0.0598	0.0127	2.58E-06	22.1715
M34535.m	rs705795	T	C	0.2579	0.0551	0.0104	1.13E-07	28.0696
M34535.m	rs8180157	A	G	0.3926	-0.041	0.0091	6.97E-06	20.2995
M34535.m	rs9634561	A	G	0.1388	0.0664	0.0149	7.77E-06	19.8593
M34539.m	rs10010466	T	G	0.6845	0.0214	0.0043	6.53E-07	24.768
M34539.m	rs11042916	T	C	0.2479	0.0226	0.0044	2.8E-07	26.3822
M34539.m	rs11117202	T	C	0.6066	-0.0353	0.008	9.76E-06	19.4702
M34539.m	rs1171614	T	C	0.2232	-0.0263	0.0047	2.55E-08	31.3124
M34539.m	rs12083933	T	C	0.1261	0.0274	0.006	4.5E-06	20.8544
M34539.m	rs12136754	A	C	0.3343	0.026	0.0043	1.24E-09	36.5603
M34539.m	rs12323577	A	G	0.0169	-0.0737	0.0165	8.33E-06	19.9511
M34539.m	rs12621754	T	C	0.0102	-0.1607	0.0363	9.47E-06	19.5983
M34539.m	rs1490956	A	G	0.8464	0.0362	0.0079	4.02E-06	20.9973
M34539.m	rs1711005	A	G	0.1356	-0.0279	0.0059	2.08E-06	22.3617
M34539.m	rs17187240	T	C	0.9712	-0.0607	0.0132	3.91E-06	21.1461
M34539.m	rs2026781	C	G	0.7702	0.0351	0.0076	3.83E-06	21.3298
M34539.m	rs2230604	T	C	0.0848	0.0309	0.007	1E-05	19.4859
M34539.m	rs2246344	T	G	0.7691	-0.0197	0.0044	7.93E-06	20.046
M34539.m	rs3751563	A	G	0.1086	0.0288	0.0062	3.03E-06	21.5775
M34539.m	rs6802120	T	C	0.1586	0.0241	0.0052	3.99E-06	21.4797
M34539.m	rs7340748	A	G	0.0539	0.0437	0.0097	5.92E-06	20.2964
M34539.m	rs837767	A	G	0.1777	0.0238	0.0054	9.6E-06	19.4252
M34539.m	rs9348403	T	C	0.6666	-0.0207	0.0043	1.61E-06	23.1741
M34539.m	rs9832461	A	G	0.7731	-0.0203	0.0044	4.1E-06	21.2856
M34674.m	rs12150661	A	G	0.0778	0.0593	0.0134	9.21E-06	19.5839
M34674.m	rs16879493	A	C	0.9479	0.052	0.0106	9.81E-07	24.0655
M34674.m	rs17423134	A	G	0.0656	0.0498	0.0108	3.75E-06	21.2623
M34674.m	rs174532	A	G	0.2915	0.0296	0.0064	4.15E-06	21.3906
M34674.m	rs2099919	T	C	0.9196	0.0432	0.0098	9.72E-06	19.4319
M34674.m	rs2172784	T	C	0.8464	0.0323	0.0072	7.5E-06	20.1252
M34674.m	rs2727271	A	T	0.8882	0.0551	0.0083	2.74E-11	44.0704
M34674.m	rs4452305	A	G	0.9776	-0.0807	0.0181	8.48E-06	19.8788
M34674.m	rs6436677	T	C	0.0189	0.1131	0.0241	2.66E-06	22.0237
M34674.m	rs6947774	T	C	0.3078	0.025	0.0055	6.65E-06	20.6612
M34674.m	rs7008266	A	G	0.4515	-0.0231	0.0052	9.33E-06	19.7341
M34674.m	rs8178281	A	G	0.9728	-0.0849	0.0185	4.3E-06	21.0607
M34674.m	rs9806159	T	G	0.1395	-0.05	0.0097	2.56E-07	26.5703
M34732.m	rs1456197	####	C	0.5846	-0.0169	0.0038	6.43E-06	19.7791
M34732.m	rs4680092	####	G	0.2203	-0.0169	0.0036	2.19E-06	22.0378
M34732.m	rs4861137	####	C	0.6136	0.016	0.0036	6.91E-06	19.7531
M34732.m	rs6907935	####	C	0.6945	-0.0172	0.0037	3.81E-06	21.6099
M34761.m	rs10833309	A	G	0.1893	-0.0258	0.0055	2.43E-06	22.0046
M34761.m	rs11919637	A	G	0.7317	0.0195	0.0043	5.96E-06	20.5652
M34761.m	rs12443738	T	G	0.1232	0.0269	0.006	8.29E-06	20.1003
M34761.m	rs12717560	A	G	0.2719	-0.0222	0.0044	6.05E-07	25.4566
M34761.m	rs13019537	C	G	0.8426	0.0289	0.0058	5.35E-07	24.8279
M34761.m	rs1419916	T	C	0.3141	0.0201	0.0042	2.27E-06	22.9031
M34761.m	rs1477025	T	C	0.6553	0.0215	0.0042	3.08E-07	26.2046
M34761.m	rs17597208	A	G	0.8973	-0.0302	0.0066	5.01E-06	20.9376

M34761.m	rs2162518	A	C	0.8896	-0.0305	0.0065	3.05E-06	22.0178
M34761.m	rs2878733	T	C	0.703	0.021	0.0043	1.18E-06	23.8507
M34761.m	rs512251	A	T	0.8615	0.028	0.0058	1.44E-06	23.3056
M34761.m	rs5997515	T	C	0.0469	-0.0508	0.0109	3.27E-06	21.7207
M34761.m	rs6075489	A	G	0.7191	0.0245	0.005	8.65E-07	24.01
M34761.m	rs633290	A	G	0.6791	-0.0217	0.0042	2.13E-07	26.6944
M34761.m	rs6544157	A	G	0.9024	-0.0329	0.0069	1.83E-06	22.7349
M34761.m	rs7130402	A	G	0.9351	0.0367	0.0081	6.6E-06	20.5287
M34761.m	rs8015976	A	G	0.7244	-0.0199	0.0042	2.58E-06	22.4495
M34761.m	rs8022857	T	C	0.9764	0.0772	0.0136	1.45E-08	32.2223
M34761.m	rs9788090	C	G	0.7338	0.0248	0.0054	5.22E-06	21.0919
M34878.m	rs11102783	A	G	0.1984	0.0379	0.0081	3.16E-06	21.8932
M34878.m	rs11752994	T	C	0.0666	0.0927	0.0191	1.26E-06	23.5555
M34878.m	rs1384609	T	C	0.474	-0.046	0.01	4.23E-06	21.16
M34878.m	rs16960359	A	G	0.1019	-0.0433	0.0098	9.3E-06	19.522
M34878.m	rs16964257	T	C	0.9549	-0.0669	0.0145	4.02E-06	21.2871
M34878.m	rs1859570	T	C	0.0272	0.1451	0.0309	2.65E-06	22.0505
M34878.m	rs2800903	T	C	0.4619	-0.026	0.0059	9.19E-06	19.4197
M34878.m	rs32281	T	C	0.9844	-0.1422	0.0293	1.22E-06	23.554
M34878.m	rs7942717	A	G	0.9543	-0.1094	0.0235	3.18E-06	21.672
M34878.m	rs9344588	T	C	0.1831	0.0389	0.0082	2.31E-06	22.5046
M34878.m	rs9924628	A	C	0.3336	-0.0313	0.0068	3.56E-06	21.1871
M34912.m	rs10194375	A	C	0.3347	-0.0086	0.0019	6.48E-06	20.4875
M34912.m	rs10902096	T	C	0.1601	0.0124	0.0028	9.75E-06	19.6122
M34912.m	rs10934975	T	C	0.5406	0.0085	0.0019	6.32E-06	20.0139
M34912.m	rs11076175	A	G	0.845	0.017	0.003	1.46E-08	32.1111
M34912.m	rs11263765	A	G	0.7777	0.0132	0.0028	2.01E-06	22.2245
M34912.m	rs11606597	A	C	0.3632	-0.0092	0.0019	1.11E-06	23.446
M34912.m	rs11607529	T	C	0.5424	0.009	0.0019	1.82E-06	22.4377
M34912.m	rs11802105	A	G	0.5682	-0.0094	0.0019	6.8E-07	24.4765
M34912.m	rs11880261	T	C	0.2898	-0.0335	0.0063	1.3E-07	28.2754
M34912.m	rs12195633	T	C	0.6339	-0.0089	0.0019	2.49E-06	21.9418
M34912.m	rs12215840	T	C	0.5911	0.0085	0.0019	6.09E-06	20.0139
M34912.m	rs12286796	A	C	0.5493	-0.0088	0.0019	3.71E-06	21.4515
M34912.m	rs12708853	A	C	0.5148	-0.0092	0.0019	1.17E-06	23.446
M34912.m	rs13023630	T	G	0.4468	0.0086	0.0019	6.95E-06	20.4875
M34912.m	rs13261860	A	G	0.4078	-0.009	0.0019	1.92E-06	22.4377
M34912.m	rs1339995	A	C	0.4714	0.011	0.0019	1.63E-08	33.518
M34912.m	rs1347222	T	C	0.3688	0.009	0.0019	2.01E-06	22.4377
M34912.m	rs1374653	A	G	0.1325	-0.0125	0.0028	7.88E-06	19.9298
M34912.m	rs1422587	T	C	0.3521	0.0095	0.0019	4.87E-07	25
M34912.m	rs17049231	A	G	0.9798	-0.0325	0.0073	8.74E-06	19.8208
M34912.m	rs17406556	T	C	0.959	0.028	0.006	3.06E-06	21.7778
M34912.m	rs17712928	A	G	0.3432	0.0088	0.0019	3.59E-06	21.4515
M34912.m	rs1887820	A	G	0.6665	0.0085	0.0019	8.47E-06	20.0139
M34912.m	rs1933830	T	C	0.4953	-0.009	0.0019	1.87E-06	22.4377
M34912.m	rs202093	T	C	0.3918	0.0086	0.0019	4.99E-06	20.4875
M34912.m	rs2072583	A	G	0.185	0.0163	0.0036	6.42E-06	20.5008
M34912.m	rs2970366	A	G	0.1664	-0.0126	0.0028	4.95E-06	20.25
M34912.m	rs32487	A	G	0.8291	-0.0126	0.0028	6.25E-06	20.25
M34912.m	rs4244568	T	G	0.6271	-0.0086	0.0019	5.41E-06	20.4875
M34912.m	rs4520895	A	C	0.8582	0.013	0.0028	4.28E-06	21.5561
M34912.m	rs4583642	T	C	0.3636	-0.0085	0.0019	6.57E-06	20.0139
M34912.m	rs4674025	T	C	0.4561	-0.0091	0.0019	2.03E-06	22.9391
M34912.m	rs4676471	A	G	0.0254	0.0401	0.009	8.62E-06	19.852

M34912.m	rs4976671	C	G	0.9854	0.0989	0.0217	5.01E-06	20.7718
M34912.m	rs6796650	A	G	0.5569	-0.0092	0.0019	1.17E-06	23.446
M34912.m	rs6834482	T	C	0.6503	-0.0092	0.0019	1.45E-06	23.446
M34912.m	rs698015	T	C	0.6477	0.0085	0.0019	6.72E-06	20.0139
M34912.m	rs7022352	A	G	0.5336	0.0091	0.0019	1.48E-06	22.9391
M34912.m	rs762864	A	G	0.6215	-0.0085	0.0019	8.82E-06	20.0139
M34912.m	rs7755681	T	C	0.5943	0.0089	0.0019	2.58E-06	21.9418
M34912.m	rs922331	T	C	0.3698	0.0087	0.0019	6.14E-06	20.9668
M34912.m	rs9357080	A	G	0.9074	-0.0167	0.0037	5.32E-06	20.3718
M34912.m	rs9459207	A	G	0.0331	-0.0258	0.0056	3.36E-06	21.2258
M34912.m	rs9807799	T	C	0.1397	-0.0126	0.0028	6.56E-06	20.25
M34912.m	rs982702	T	C	0.3591	-0.0089	0.0019	2.49E-06	21.9418
M34912.m	rs9845510	T	C	0.5512	-0.0088	0.0019	2.92E-06	21.4515
M35114.m	rs12049480	T	G	0.744	0.0273	0.0057	1.81E-06	22.9391
M35114.m	rs12431841	T	C	0.014	0.0975	0.0215	6.12E-06	20.5652
M35114.m	rs1415540	C	G	0.2891	-0.0149	0.0034	9.57E-06	19.205
M35114.m	rs3949795	A	G	0.383	-0.0235	0.0053	8.08E-06	19.66
M35114.m	rs677302	T	C	0.4637	0.0228	0.0051	7.83E-06	19.9862
M35114.m	rs6915542	T	C	0.8413	-0.0184	0.0042	9.25E-06	19.1927
M35114.m	rs6969207	A	G	0.1665	-0.0332	0.0075	9.6E-06	19.5954
M35114.m	rs6981309	A	G	0.7122	0.015	0.0034	7.47E-06	19.4637
M35114.m	rs9513567	C	G	0.2905	0.0262	0.0055	2.08E-06	22.6922
M35114.m	rs9992384	T	G	0.2562	0.0157	0.0034	2.81E-06	21.3227
M35126.m	rs10031835	T	C	0.6951	-0.0494	0.0109	5.3E-06	20.54
M35126.m	rs12680735	A	G	0.9872	0.206	0.0414	6.44E-07	24.759
M35126.m	rs2025277	A	G	0.1872	-0.03	0.0061	7.93E-07	24.187
M35126.m	rs2073123	T	C	0.5214	0.0195	0.0044	9.62E-06	19.641
M35126.m	rs2217851	A	G	0.5917	0.0236	0.0051	3.01E-06	21.4133
M35126.m	rs3127434	A	G	0.489	0.0198	0.0044	7.05E-06	20.25
M35126.m	rs3911458	C	G	0.9839	-0.0845	0.019	8.35E-06	19.7791
M35126.m	rs4628823	T	C	0.9434	0.0468	0.0101	3.68E-06	21.4708
M35126.m	rs4797105	C	G	0.7968	0.0406	0.0074	3.59E-08	30.1015
M35126.m	rs4938200	T	C	0.4388	-0.0203	0.0044	4.31E-06	21.2856
M35126.m	rs551938	A	G	0.2537	-0.0236	0.0052	5.63E-06	20.5976
M35126.m	rs629187	A	G	0.8438	-0.0327	0.0061	1.03E-07	28.7366
M35126.m	rs6429216	A	G	0.464	-0.023	0.005	4.23E-06	21.16
M35126.m	rs6972443	C	G	0.8153	0.0299	0.0066	5.78E-06	20.5236
M35126.m	rs879092	A	T	0.9154	0.0434	0.0083	1.56E-07	27.3416
M35126.m	rs929005	A	T	0.1602	-0.0349	0.0075	3.18E-06	21.6535
M35126.m	rs973579	A	G	0.5112	-0.0204	0.0044	4.64E-06	21.4959
M35127.m	rs1095462	T	C	0.3553	-0.0252	0.005	4.13E-07	25.4016
M35127.m	rs10963693	C	G	0.5857	0.0113	0.0026	9.61E-06	18.8891
M35127.m	rs1136613	A	G	0.0194	-0.0781	0.0164	2.01E-06	22.6785
M35127.m	rs11920551	A	G	0.218	0.0174	0.0033	1.63E-07	27.8017
M35127.m	rs12703821	A	T	0.6236	-0.0217	0.0047	4.64E-06	21.3169
M35127.m	rs177387	A	G	0.5143	-0.0114	0.0025	6.19E-06	20.7936
M35127.m	rs1812081	T	G	0.3963	0.0114	0.0025	5.48E-06	20.7936
M35127.m	rs1896198	A	G	0.416	-0.0124	0.0025	8.67E-07	24.6016
M35127.m	rs1930131	T	C	0.821	-0.0159	0.0034	2.35E-06	21.8694
M35127.m	rs1957252	C	G	0.1428	0.0177	0.004	9.85E-06	19.5806
M35127.m	rs270771	T	C	0.0551	-0.0279	0.0061	4.02E-06	20.9194
M35127.m	rs3108879	T	C	0.6251	-0.0116	0.0026	5.88E-06	19.9053
M35127.m	rs4839333	A	T	0.8828	0.0186	0.004	2.91E-06	21.6225
M35127.m	rs645288	C	G	0.2747	-0.0141	0.0031	6.45E-06	20.6878
M35127.m	rs737842	A	G	0.5825	-0.012	0.0026	3.05E-06	21.3018

M35127.m	rs748651	T	C	0.5135	-0.0124	0.0027	4.06E-06	21.0919
M35127.m	rs9479367	A	G	0.4575	0.0112	0.0025	8.95E-06	20.0704
M35127.m	rs9525616	A	G	0.4711	0.0119	0.0025	2.92E-06	22.6576
M35127.m	rs9528023	T	C	0.2466	-0.0146	0.0031	3.36E-06	22.1811
M35127.m	rs9594738	T	C	0.4782	0.0137	0.0025	4.53E-08	30.0304
M35137.m	rs1015380	T	C	0.878	0.013	0.0028	2.93E-06	21.5561
M35137.m	rs10492449	T	G	0.6924	0.009	0.0019	2.31E-06	22.4377
M35137.m	rs10962395	T	C	0.5964	-0.0094	0.0019	9.52E-07	24.4765
M35137.m	rs1106609	T	C	0.6756	0.0087	0.0019	4.81E-06	20.9668
M35137.m	rs11131993	T	G	0.6998	-0.0097	0.0019	4.2E-07	26.0637
M35137.m	rs11132011	A	G	0.6439	-0.0085	0.0019	8.62E-06	20.0139
M35137.m	rs11657003	T	G	0.7079	-0.0091	0.0019	2.23E-06	22.9391
M35137.m	rs12499670	T	C	0.3638	-0.0088	0.0019	3.7E-06	21.4515
M35137.m	rs12520768	A	C	0.3051	0.0085	0.0019	8.1E-06	20.0139
M35137.m	rs13202369	A	G	0.6956	0.0085	0.0019	9.49E-06	20.0139
M35137.m	rs1464136	A	C	0.7294	0.0087	0.0019	5.32E-06	20.9668
M35137.m	rs1468977	A	G	0.6067	0.0084	0.0019	9.58E-06	19.5457
M35137.m	rs16986182	C	G	0.9857	-0.1275	0.0267	1.81E-06	22.8033
M35137.m	rs17113525	T	G	0.8744	-0.0132	0.0029	3.68E-06	20.7182
M35137.m	rs17340319	C	G	0.9347	0.0217	0.0044	8.78E-07	24.3228
M35137.m	rs2036152	A	G	0.3028	-0.0084	0.0019	9.42E-06	19.5457
M35137.m	rs2064443	A	G	0.5417	-0.0087	0.0019	4.22E-06	20.9668
M35137.m	rs2214626	C	G	0.6223	-0.0087	0.0019	4.28E-06	20.9668
M35137.m	rs2351921	T	C	0.3571	0.0085	0.0019	7.48E-06	20.0139
M35137.m	rs2468526	T	C	0.6827	0.0097	0.0019	3.48E-07	26.0637
M35137.m	rs2928949	T	C	0.6858	0.0092	0.0019	1.52E-06	23.446
M35137.m	rs2966713	A	G	0.3387	-0.0087	0.0019	5.9E-06	20.9668
M35137.m	rs2969990	A	T	0.3814	0.0089	0.0019	2.82E-06	21.9418
M35137.m	rs2982694	T	G	0.8716	-0.041	0.0087	2.49E-06	22.209
M35137.m	rs3121910	T	G	0.7221	-0.0091	0.0019	1.99E-06	22.9391
M35137.m	rs3801969	T	G	0.4459	-0.0087	0.0019	4.72E-06	20.9668
M35137.m	rs3843430	T	C	0.371	-0.0094	0.0019	5.81E-07	24.4765
M35137.m	rs4142154	A	G	0.3524	0.0086	0.0019	7.38E-06	20.4875
M35137.m	rs4326611	T	C	0.4285	-0.0087	0.0019	6.06E-06	20.9668
M35137.m	rs4398300	A	G	0.6235	0.0091	0.0019	1.84E-06	22.9391
M35137.m	rs4659909	A	G	0.1755	-0.0128	0.0028	5.06E-06	20.898
M35137.m	rs4908655	C	G	0.2906	-0.0085	0.0019	8.42E-06	20.0139
M35137.m	rs4975842	C	G	0.4075	0.0085	0.0019	7.49E-06	20.0139
M35137.m	rs550328	A	G	0.2687	0.0099	0.0019	2.54E-07	27.1496
M35137.m	rs595286	A	G	0.5466	-0.0095	0.0019	8.62E-07	25
M35137.m	rs6026567	A	G	0.5792	-0.0096	0.0019	5.87E-07	25.5291
M35137.m	rs618791	T	C	0.6833	-0.0087	0.0019	4.42E-06	20.9668
M35137.m	rs6564173	A	G	0.3642	0.0086	0.0019	5.4E-06	20.4875
M35137.m	rs6659436	T	C	0.0333	0.0246	0.0055	7.43E-06	20.0053
M35137.m	rs6786214	A	G	0.3424	-0.0085	0.0019	8.88E-06	20.0139
M35137.m	rs6788007	T	C	0.4017	0.009	0.0019	2.17E-06	22.4377
M35137.m	rs6836081	T	C	0.3887	-0.0085	0.0019	6.85E-06	20.0139
M35137.m	rs6871718	T	C	0.665	-0.0091	0.0019	1.75E-06	22.9391
M35137.m	rs696958	A	G	0.3585	-0.0089	0.0019	3.05E-06	21.9418
M35137.m	rs6979908	A	G	0.1274	-0.0132	0.0028	3.05E-06	22.2245
M35137.m	rs7179088	T	C	0.3056	0.0098	0.0019	2.86E-07	26.6039
M35137.m	rs7200218	C	G	0.6479	-0.0085	0.0019	8.27E-06	20.0139
M35137.m	rs744432	A	C	0.4475	-0.0094	0.0019	6.34E-07	24.4765
M35137.m	rs7446715	A	G	0.6732	-0.01	0.0019	1.81E-07	27.7008
M35137.m	rs7505882	T	C	0.4807	-0.0087	0.0019	5.43E-06	20.9668

M35137.m	rs764134	C	G	0.6366	-0.0085	0.0019	7.37E-06	20.0139
M35137.m	rs7749940	A	C	0.3736	0.0109	0.0019	8.3E-09	32.9114
M35137.m	rs7770207	T	C	0.689	-0.0088	0.0019	3.94E-06	21.4515
M35137.m	rs7927428	A	C	0.6695	-0.0085	0.0019	7.58E-06	20.0139
M35137.m	rs8039730	T	C	0.3197	-0.0085	0.0019	8.56E-06	20.0139
M35137.m	rs820140	T	C	0.4898	-0.0087	0.0019	4.65E-06	20.9668
M35137.m	rs9297758	A	G	0.3801	-0.0094	0.0019	8.02E-07	24.4765
M35137.m	rs9649625	A	C	0.2883	-0.0097	0.0019	5.81E-07	26.0637
M35159.m	rs11150072	A	G	0.7148	0.0394	0.0083	2.38E-06	22.5339
M35159.m	rs12260890	T	C	0.9341	-0.0596	0.0131	5.63E-06	20.699
M35159.m	rs12895662	T	C	0.6305	-0.0302	0.0062	1.24E-06	23.7263
M35159.m	rs17624641	C	G	0.2139	-0.0325	0.007	3.81E-06	21.5561
M35159.m	rs2688	T	G	0.6039	0.0321	0.0067	1.46E-06	22.9541
M35159.m	rs4340260	T	C	0.4537	-0.0363	0.0077	2.67E-06	22.2245
M35159.m	rs4758441	A	G	0.2648	-0.0295	0.0065	5.31E-06	20.5976
M35159.m	rs5760492	A	G	0.333	-0.0492	0.007	2.34E-12	49.4008
M35159.m	rs7218918	A	G	0.4081	-0.0296	0.0065	4.63E-06	20.7375
M35160.m	rs1028374	A	G	0.1409	0.0264	0.0053	5.88E-07	24.8117
M35160.m	rs1348656	T	C	0.7981	-0.0209	0.0043	1.34E-06	23.6241
M35160.m	rs17777742	A	C	0.5951	0.0161	0.0036	9.97E-06	20.0008
M35160.m	rs2458177	C	G	0.3897	0.0158	0.0035	8.47E-06	20.3788
M35160.m	rs2485904	A	C	0.1563	0.0251	0.0052	1.4E-06	23.2992
M35160.m	rs360020	A	G	0.6799	0.0196	0.0042	2.48E-06	21.7778
M35160.m	rs3748771	T	G	0.9877	-0.1245	0.0281	9.09E-06	19.6303
M35160.m	rs4703415	T	C	0.1469	-0.0226	0.005	6.55E-06	20.4304
M35160.m	rs7115681	T	C	0.4669	-0.0184	0.0041	8.18E-06	20.1404
M35160.m	rs7672265	T	G	0.1877	0.0196	0.0044	7.95E-06	19.843
M35160.m	rs9600082	A	C	0.194	0.0206	0.0046	7.87E-06	20.0548
M35186.m	rs10044369	T	C	0.763	0.0172	0.0034	3.26E-07	25.5917
M35186.m	rs1010377	T	C	0.3033	0.0119	0.0026	6.76E-06	20.9482
M35186.m	rs10468017	T	C	0.2875	0.0171	0.0027	1.29E-10	40.1111
M35186.m	rs11045906	A	G	0.7483	0.0154	0.0027	7.46E-09	32.5322
M35186.m	rs11048586	A	G	0.7449	-0.0128	0.0027	1.85E-06	22.4746
M35186.m	rs11081670	T	C	0.3179	0.0121	0.0026	3.7E-06	21.6583
M35186.m	rs1161762	T	C	0.7232	0.0123	0.0027	4.02E-06	20.7531
M35186.m	rs17031728	C	G	0.8162	-0.0181	0.0034	7.68E-08	28.34
M35186.m	rs17152841	T	C	0.7203	-0.0119	0.0026	5.93E-06	20.9482
M35186.m	rs174578	A	T	0.341	-0.0354	0.0026	6.12E-42	185.379
M35186.m	rs1984049	A	C	0.8108	0.0307	0.0068	5.33E-06	20.3826
M35186.m	rs198476	A	G	0.4805	0.0134	0.0026	1.56E-07	26.5621
M35186.m	rs2033371	A	G	0.2609	-0.0119	0.0027	7.89E-06	19.4252
M35186.m	rs2070895	A	G	0.2085	0.0176	0.0033	1.28E-07	28.4444
M35186.m	rs2072583	A	G	0.1954	0.0186	0.0042	8.71E-06	19.6122
M35186.m	rs2392820	A	G	0.28	0.0123	0.0027	3.86E-06	20.7531
M35186.m	rs2504453	A	T	0.8969	0.02	0.0043	3.35E-06	21.6333
M35186.m	rs35544	A	G	0.8264	0.0158	0.0034	4.26E-06	21.5952
M35186.m	rs381227	T	C	0.287	-0.0121	0.0027	6.3E-06	20.0837
M35186.m	rs39741	A	G	0.0945	-0.0199	0.0043	4.51E-06	21.4175
M35186.m	rs4149056	T	C	0.8405	-0.0294	0.0034	2.66E-18	74.7716
M35186.m	rs4257232	A	G	0.5675	0.012	0.0026	4.17E-06	21.3018
M35186.m	rs4530945	T	C	0.732	-0.0117	0.0026	9.98E-06	20.25
M35186.m	rs6564097	T	C	0.3496	0.0116	0.0026	9.67E-06	19.9053
M35186.m	rs6722402	T	G	0.7106	-0.0124	0.0027	2.99E-06	21.0919
M35186.m	rs7307046	A	G	0.4072	0.0122	0.0026	2.33E-06	22.0178
M35186.m	rs7599943	T	C	0.3473	-0.012	0.0026	5.17E-06	21.3018

M35186.m	rs9624896	T	C	0.2909	-0.0125	0.0027	2.4E-06	21.4335
M35187.m	rs10505870	A	G	0.2722	0.0396	0.0074	7.34E-08	28.637
M35187.m	rs11045823	A	G	0.163	-0.106	0.0083	6.22E-37	163.101
M35187.m	rs12160763	A	G	0.9499	0.0681	0.0142	1.78E-06	22.9995
M35187.m	rs12317268	A	G	0.8337	-0.1377	0.0079	2.09E-67	303.818
M35187.m	rs12810446	T	C	0.272	-0.047	0.0072	5.29E-11	42.6119
M35187.m	rs13276952	A	C	0.3037	0.0657	0.0146	6.5E-06	20.25
M35187.m	rs296381	T	C	0.8302	0.053	0.0082	1.07E-10	41.7757
M35187.m	rs4084390	C	G	0.238	0.0323	0.0072	7.24E-06	20.1252
M35187.m	rs7137767	A	C	0.5556	0.0313	0.0066	2.04E-06	22.4906
M35187.m	rs7975166	A	G	0.047	-0.077	0.0147	1.68E-07	27.4376
M35187.m	rs8063217	T	C	0.0594	0.1278	0.0279	4.7E-06	20.9823
M35187.m	rs932055	T	C	0.1909	-0.0333	0.0075	9.4E-06	19.7136
M35189.m	rs11758894	T	C	0.0242	0.0766	0.017	6.36E-06	20.303
M35189.m	rs12566232	A	C	0.7095	-0.0446	0.005	5.69E-19	79.5664
M35189.m	rs13003126	T	C	0.1214	0.0346	0.0068	3.63E-07	25.8901
M35189.m	rs1509821	T	C	0.1091	0.0574	0.0076	5.29E-14	57.0422
M35189.m	rs16842610	T	G	0.1989	0.0313	0.0059	9.89E-08	28.1439
M35189.m	rs1693551	T	C	0.5342	-0.0197	0.0043	4.79E-06	20.9892
M35189.m	rs17743577	T	C	0.8014	-0.0269	0.0058	3.74E-06	21.5104
M35189.m	rs17865343	A	G	0.089	-0.0412	0.0083	6.16E-07	24.6399
M35189.m	rs1947110	A	G	0.284	0.0235	0.0051	3.34E-06	21.2322
M35189.m	rs2048073	A	G	0.1579	-0.03	0.0065	4.13E-06	21.3018
M35189.m	rs2302539	A	G	0.4002	0.0492	0.0044	1.37E-28	125.033
M35189.m	rs2493599	T	C	0.4969	0.0191	0.0043	9.6E-06	19.7301
M35189.m	rs2539861	A	C	0.9191	0.057	0.0085	2.21E-11	44.9689
M35189.m	rs3738934	T	C	0.6231	-0.1056	0.0043	1.2E-134	603.102
M35189.m	rs3742758	A	G	0.0191	0.0739	0.0159	3.45E-06	21.602
M35189.m	rs4672574	T	C	0.6361	0.0329	0.0049	1.99E-11	45.0816
M35189.m	rs4687148	T	G	0.9265	0.0489	0.0104	2.65E-06	22.1081
M35189.m	rs6733319	A	G	0.7679	0.0251	0.0053	1.88E-06	22.4283
M35189.m	rs6740556	A	G	0.2467	0.0236	0.0051	3.83E-06	21.4133
M35189.m	rs7740539	T	C	0.7104	-0.0227	0.005	6.73E-06	20.6116
M35189.m	rs7741438	A	G	0.4744	0.0255	0.005	3.38E-07	26.01
M35189.m	rs992029	T	C	0.0981	-0.0422	0.0077	3.93E-08	30.0361
M35193.m	rs10211153	T	G	0.4131	-0.0128	0.0027	2.9E-06	22.4746
M35193.m	rs11150996	T	C	0.6181	-0.0158	0.0034	3.54E-06	21.5952
M35193.m	rs12071803	A	G	0.3609	-0.0122	0.0028	9.92E-06	18.9847
M35193.m	rs12352994	T	C	0.8104	0.0184	0.0036	2.54E-07	26.1235
M35193.m	rs13390982	T	C	0.9232	0.024	0.0053	6.56E-06	20.5055
M35193.m	rs16865980	A	G	0.2149	-0.0162	0.0036	6.32E-06	20.25
M35193.m	rs1853936	T	C	0.1762	0.021	0.0043	8.96E-07	23.8507
M35193.m	rs1991108	T	C	0.1597	-0.0183	0.0037	5.77E-07	24.4624
M35193.m	rs2062541	A	G	0.615	0.0196	0.0027	5.76E-13	52.6968
M35193.m	rs2278350	A	C	0.3247	-0.0159	0.0035	4.62E-06	20.6376
M35193.m	rs2326409	T	C	0.1422	0.023	0.0045	2.86E-07	26.1235
M35193.m	rs2326537	A	T	0.0294	-0.1064	0.0206	2.43E-07	26.6777
M35193.m	rs2378668	A	C	0.3623	0.0138	0.0027	5.04E-07	26.1235
M35193.m	rs274567	T	C	0.3687	0.0144	0.0027	1.23E-07	28.4444
M35193.m	rs3752192	A	G	0.5526	0.0124	0.0027	5.05E-06	21.0919
M35193.m	rs4536931	C	G	0.0288	-0.0494	0.0102	1.37E-06	23.456
M35193.m	rs4960438	T	C	0.6378	-0.0125	0.0027	4.75E-06	21.4335
M35193.m	rs583881	T	C	0.057	0.0317	0.0064	8.22E-07	24.5334
M35193.m	rs6107820	A	G	0.5819	-0.0121	0.0027	9.95E-06	20.0837
M35193.m	rs6597030	T	C	0.0839	-0.0374	0.0053	1.61E-12	49.7957

M35193.m	rs6734857	A	T	0.1629	-0.0221	0.005	8.51E-06	19.5364
M35193.m	rs6817210	A	C	0.5169	-0.0123	0.0027	6.58E-06	20.7531
M35193.m	rs6973126	T	C	0.5122	0.0122	0.0027	7.06E-06	20.417
M35193.m	rs7942744	T	C	0.556	0.0124	0.0027	6.42E-06	21.0919
M35193.m	rs9392556	T	C	0.6581	-0.0358	0.0028	4.03E-38	163.474
M35193.m	rs9588850	A	G	0.2225	0.0159	0.0036	8.82E-06	19.5069
M35193.m	rs9871214	T	G	0.1789	-0.0161	0.0036	8.39E-06	20.0008
M35240.m	rs10274162	T	G	0.4586	-0.0136	0.003	5.25E-06	20.5511
M35240.m	rs1151428	A	G	0.1545	-0.0178	0.0039	4.77E-06	20.831
M35240.m	rs11726946	A	G	0.8837	-0.0187	0.0042	9.32E-06	19.8237
M35240.m	rs1188774	T	C	0.0826	-0.0222	0.005	9.96E-06	19.7136
M35240.m	rs17022603	A	G	0.9828	-0.0771	0.0164	2.46E-06	22.1015
M35240.m	rs1981524	T	C	0.2032	0.015	0.0033	4.71E-06	20.6612
M35240.m	rs6981610	A	G	0.708	0.0141	0.0032	7.91E-06	19.415
M35253.m	rs10019315	T	C	0.4833	0.0134	0.0026	3.23E-07	26.5621
M35253.m	rs10951614	C	G	0.3777	-0.013	0.0027	1.02E-06	23.1824
M35253.m	rs11078257	A	G	0.3384	0.0124	0.0027	3.09E-06	21.0919
M35253.m	rs11949991	C	G	0.8327	0.0159	0.0035	5.61E-06	20.6376
M35253.m	rs12155847	T	C	0.6503	0.0124	0.0027	3.64E-06	21.0919
M35253.m	rs12588013	T	G	0.3744	0.0117	0.0026	8.57E-06	20.25
M35253.m	rs12726916	T	G	0.1774	-0.0159	0.0035	4.04E-06	20.6376
M35253.m	rs17176375	A	G	0.1078	0.0211	0.0043	8.44E-07	24.0784
M35253.m	rs1745299	T	G	0.0125	0.1406	0.0311	6.09E-06	20.4385
M35253.m	rs1954482	A	G	0.3157	-0.0122	0.0027	4.43E-06	20.417
M35253.m	rs2122506	A	G	0.3253	0.0118	0.0026	9.05E-06	20.5976
M35253.m	rs2236434	C	G	0.6878	-0.0124	0.0027	3.71E-06	21.0919
M35253.m	rs2479415	T	C	0.5265	0.0121	0.0027	8.98E-06	20.0837
M35253.m	rs329498	T	G	0.3106	-0.0121	0.0027	5.67E-06	20.0837
M35253.m	rs4507280	A	T	0.1488	0.0161	0.0035	3.49E-06	21.16
M35253.m	rs485868	A	G	0.7155	-0.0135	0.0027	4.36E-07	25
M35253.m	rs4895424	A	G	0.3837	0.0124	0.0026	2.94E-06	22.7456
M35253.m	rs4905	A	G	0.7122	0.0124	0.0027	2.92E-06	21.0919
M35253.m	rs6817243	A	G	0.2809	0.0125	0.0027	2.47E-06	21.4335
M35253.m	rs736396	T	C	0.3554	-0.0122	0.0027	4.68E-06	20.417
M35253.m	rs748196	T	G	0.4604	0.0117	0.0026	7.34E-06	20.25
M35253.m	rs7767262	T	C	0.9628	0.0324	0.0069	2.64E-06	22.0491
M35253.m	rs7987737	T	C	0.986	-0.0578	0.0126	4.48E-06	21.0433
M35253.m	rs9293713	A	G	0.0653	-0.0242	0.0052	3.39E-06	21.6583
M35254.m	rs10892968	T	G	0.8214	0.0155	0.0035	8.34E-06	19.6122
M35254.m	rs11049103	A	G	0.1677	0.0161	0.0036	6.38E-06	20.0008
M35254.m	rs11078257	A	G	0.3385	0.0132	0.0027	8.95E-07	23.9012
M35254.m	rs11100892	A	G	0.4833	0.0134	0.0026	3.85E-07	26.5621
M35254.m	rs1263632	T	C	0.4945	-0.0119	0.0026	6.16E-06	20.9482
M35254.m	rs13149488	A	G	0.5433	0.014	0.0026	1.24E-07	28.9941
M35254.m	rs17084522	A	G	0.0965	0.0233	0.0051	4.74E-06	20.8724
M35254.m	rs17751407	T	G	0.0263	-0.0477	0.0104	4.52E-06	21.0363
M35254.m	rs17832491	T	C	0.0521	0.0271	0.006	6.16E-06	20.4003
M35254.m	rs2319973	A	C	0.3507	0.0125	0.0027	2.82E-06	21.4335
M35254.m	rs3808756	A	G	0.6465	0.0122	0.0027	5.65E-06	20.417
M35254.m	rs4736583	A	C	0.626	-0.0121	0.0027	5.69E-06	20.0837
M35254.m	rs486052	T	G	0.6521	0.0117	0.0026	9.46E-06	20.25
M35254.m	rs530792	T	C	0.1536	0.0159	0.0036	9.46E-06	19.5069
M35254.m	rs6061764	A	G	0.314	-0.014	0.003	3.06E-06	21.7778
M35254.m	rs9470238	T	G	0.3575	-0.0127	0.0027	3.89E-06	22.1248
M35254.m	rs9483731	T	C	0.0382	-0.0325	0.007	3.17E-06	21.5561

M35255.m	rs10463157	T	C	0.2297	-0.0224	0.0043	2.39E-07	27.1368
M35255.m	rs11671900	A	C	0.7672	0.0205	0.0044	3.63E-06	21.7071
M35255.m	rs1431465	A	G	0.4602	0.0235	0.0053	9.41E-06	19.66
M35255.m	rs1667944	A	G	0.5248	-0.0274	0.0062	8.58E-06	19.5307
M35255.m	rs16962845	T	C	0.8866	-0.0356	0.008	9.85E-06	19.8025
M35255.m	rs17032984	T	G	0.103	0.0303	0.0066	4.7E-06	21.0764
M35255.m	rs2076060	C	G	0.2579	0.0219	0.0046	1.55E-06	22.6659
M35255.m	rs2121073	T	C	0.1209	-0.0293	0.0052	2.15E-08	31.7489
M35255.m	rs2490288	T	C	0.3319	0.0177	0.0037	1.54E-06	22.8846
M35255.m	rs476092	A	C	0.2942	0.0196	0.0037	1.52E-07	28.0614
M35255.m	rs7277840	A	G	0.5377	0.0266	0.0052	3.76E-07	26.1672
M35255.m	rs7733625	T	G	0.2771	-0.017	0.0038	7.69E-06	20.0139
M35255.m	rs9857115	A	C	0.538	0.0168	0.0037	4.24E-06	20.6165
M35257.m	rs10231439	T	C	0.0747	-0.0246	0.0054	6.26E-06	20.7531
M35257.m	rs1058337	T	G	0.7966	0.0161	0.0036	5.58E-06	20.0008
M35257.m	rs11152132	A	G	0.8149	-0.0162	0.0036	6.23E-06	20.25
M35257.m	rs11712564	T	C	0.311	0.0164	0.0034	1.5E-06	23.2664
M35257.m	rs12997174	T	C	0.5646	0.0121	0.0027	6.79E-06	20.0837
M35257.m	rs13168489	T	C	0.2029	0.0164	0.0035	2.68E-06	21.9559
M35257.m	rs1397182	A	G	0.1245	0.0189	0.0043	9.78E-06	19.3191
M35257.m	rs17156176	T	C	0.9129	0.029	0.0062	3.3E-06	21.8783
M35257.m	rs17160851	T	G	0.9515	0.0335	0.0071	2.05E-06	22.2624
M35257.m	rs17300762	A	G	0.9399	0.03	0.0061	7.48E-07	24.187
M35257.m	rs174450	T	G	0.5386	-0.0164	0.0027	9.76E-10	36.8944
M35257.m	rs17700084	T	C	0.2073	0.0164	0.0035	2.83E-06	21.9559
M35257.m	rs2073234	A	G	0.6908	0.0157	0.0034	4.46E-06	21.3227
M35257.m	rs2627272	T	C	0.8848	-0.0195	0.0043	6.49E-06	20.5652
M35257.m	rs2851682	A	G	0.915	-0.0244	0.0052	3.47E-06	22.0178
M35257.m	rs416324	A	G	0.5573	-0.0126	0.0027	2.1E-06	21.7778
M35257.m	rs4270639	A	G	0.7232	-0.0168	0.0036	3.78E-06	21.7778
M35257.m	rs7588373	A	C	0.7215	0.0159	0.0034	2.97E-06	21.8694
M35257.m	rs8008572	T	C	0.7565	-0.0154	0.0034	6.69E-06	20.5156
M35257.m	rs847391	A	G	0.9514	0.039	0.0082	2.16E-06	22.6205
M35257.m	rs9405761	A	T	0.8788	0.0206	0.0044	2.95E-06	21.9194
M35257.m	rs9788021	A	T	0.158	0.0184	0.0042	9.92E-06	19.1927
M35270.m	rs10069896	A	C	0.1781	0.0104	0.0018	1.42E-08	33.3827
M35270.m	rs10519864	A	G	0.2313	0.0081	0.0018	7.04E-06	20.25
M35270.m	rs10852002	T	C	0.1049	-0.0118	0.0027	9.82E-06	19.1001
M35270.m	rs10892306	A	T	0.0417	-0.0219	0.0047	3.23E-06	21.7116
M35270.m	rs11184821	A	G	0.0247	-0.0255	0.0054	2.61E-06	22.2994
M35270.m	rs11822822	A	G	0.7848	0.0084	0.0018	4.3E-06	21.7778
M35270.m	rs11979077	A	G	0.7762	-0.0091	0.0018	5.12E-07	25.5586
M35270.m	rs1229658	A	G	0.2306	-0.0083	0.0018	6.13E-06	21.2623
M35270.m	rs12403949	A	C	0.0874	-0.0144	0.0028	1.59E-07	26.449
M35270.m	rs13188321	T	C	0.1925	-0.0086	0.0019	3.54E-06	20.4875
M35270.m	rs1331927	C	G	0.7984	-0.0085	0.0018	3.82E-06	22.2994
M35270.m	rs1462120	T	C	0.0656	0.0182	0.0036	3.28E-07	25.5586
M35270.m	rs17625634	T	C	0.1889	-0.009	0.0019	1.32E-06	22.4377
M35270.m	rs17764539	A	G	0.1973	0.009	0.0018	9.31E-07	25
M35270.m	rs1883261	A	G	0.4801	0.0084	0.0018	1.92E-06	21.7778
M35270.m	rs197926	A	G	0.5181	-0.0175	0.0037	2.19E-06	22.3703
M35270.m	rs1994003	A	C	0.1798	0.0082	0.0018	9.74E-06	20.7531
M35270.m	rs2011637	A	C	0.7366	0.0083	0.0018	4.55E-06	21.2623
M35270.m	rs2078875	T	C	0.2436	-0.0083	0.0018	4.46E-06	21.2623
M35270.m	rs2267292	T	C	0.2242	-0.0088	0.0018	1.03E-06	23.9012

M35270.m	rs2395911	T	G	0.7477	0.0081	0.0018	6.76E-06	20.25
M35270.m	rs26764	C	G	0.7599	0.0086	0.0019	3.71E-06	20.4875
M35270.m	rs2727985	C	G	0.7599	-0.0088	0.0018	2.03E-06	23.9012
M35270.m	rs2834543	T	C	0.7707	-0.0082	0.0018	7.42E-06	20.7531
M35270.m	rs3095652	A	G	0.8201	0.0083	0.0018	6.01E-06	21.2623
M35270.m	rs3770124	T	C	0.802	-0.0085	0.0018	3.23E-06	22.2994
M35270.m	rs4690150	C	G	0.7283	-0.008	0.0018	8.3E-06	19.7531
M35270.m	rs4766517	C	G	0.59	-0.008	0.0018	5.59E-06	19.7531
M35270.m	rs484080	T	C	0.0506	-0.0182	0.0036	5.87E-07	25.5586
M35270.m	rs4886189	A	G	0.4854	0.0086	0.0018	1.77E-06	22.8272
M35270.m	rs6559771	C	G	0.775	0.0084	0.0018	4.92E-06	21.7778
M35270.m	rs6784156	C	G	0.7475	-0.0082	0.0018	5.61E-06	20.7531
M35270.m	rs708496	T	C	0.5445	-0.012	0.0026	4.39E-06	21.3018
M35270.m	rs7153218	A	G	0.2736	0.0113	0.0024	3.27E-06	22.1684
M35270.m	rs7508059	A	G	0.1808	0.0093	0.0019	6.27E-07	23.9584
M35270.m	rs7670227	A	C	0.2569	-0.0084	0.0018	3.66E-06	21.7778
M35270.m	rs7815445	A	G	0.9218	-0.013	0.0028	2.42E-06	21.5561
M35270.m	rs795213	A	G	0.3339	0.01	0.0018	2.95E-08	30.8642
M35270.m	rs8069296	T	C	0.7988	0.0082	0.0018	9.97E-06	20.7531
M35270.m	rs852030	A	G	0.1953	0.0091	0.0018	6.32E-07	25.5586
M35270.m	rs9351602	T	C	0.2627	-0.0086	0.0018	1.36E-06	22.8272
M35270.m	rs9395466	A	G	0.2647	-0.008	0.0018	9.92E-06	19.7531
M35305.m	rs10019662	A	G	0.441	-0.0344	0.0075	5.21E-06	21.0375
M35305.m	rs10415849	T	C	0.1248	-0.024	0.0053	5.79E-06	20.5055
M35305.m	rs10491073	T	C	0.0171	0.1279	0.0267	1.64E-06	22.9466
M35305.m	rs12210574	T	C	0.4836	-0.0167	0.0036	3.06E-06	21.5193
M35305.m	rs1386540	T	C	0.3484	-0.0361	0.0074	1.14E-06	23.7986
M35305.m	rs1507362	T	C	0.3901	0.0333	0.0071	3.02E-06	21.9974
M35305.m	rs1554690	A	C	0.5949	0.0158	0.0035	8.17E-06	20.3788
M35305.m	rs2702042	T	C	0.35	-0.0157	0.0035	8.9E-06	20.1216
M35305.m	rs3993153	T	G	0.8085	0.0197	0.0045	9.92E-06	19.1649
M35305.m	rs7800039	A	G	0.3424	-0.0169	0.0036	2.13E-06	22.0378
M35305.m	rs8083552	A	G	0.3479	-0.0161	0.0036	5.69E-06	20.0008
M35320.x	rs11210343	A	G	0.5311	-0.0169	0.0038	8.97E-06	19.7791
M35320.x	rs11889985	T	C	0.7004	0.0493	0.0104	2.12E-06	22.4712
M35320.x	rs12660977	T	G	0.1558	0.0323	0.0072	7.11E-06	20.1252
M35320.x	rs16982373	A	G	0.0111	-0.2028	0.0451	7.02E-06	20.2201
M35320.x	rs4741433	T	G	0.438	0.018	0.0038	2.64E-06	22.4377
M35320.x	rs623248	A	G	0.8882	0.0258	0.0057	7.08E-06	20.4875
M35320.x	rs645013	T	C	0.3471	0.0173	0.0039	7.69E-06	19.6772
M35320.x	rs6897900	A	C	0.6537	0.0174	0.0039	6.4E-06	19.9053
M35320.x	rs7249061	T	C	0.5464	0.0201	0.0039	2.62E-07	26.5621
M35320.x	rs769805	T	C	0.1812	0.0248	0.0056	8.79E-06	19.6122
M35320.x	rs841225	T	C	0.5927	-0.0173	0.0038	5.37E-06	20.7265
M35320.x	rs9362985	T	C	0.3161	-0.0175	0.0039	6.48E-06	20.1348
M35322.x	rs10514235	A	G	0.04	0.1745	0.0332	1.49E-07	27.6258
M35322.x	rs10972586	A	T	0.0992	0.0839	0.0187	7.22E-06	20.1299
M35322.x	rs11011929	T	C	0.0435	-0.1477	0.0307	1.52E-06	23.1464
M35322.x	rs11119746	C	G	0.9047	-0.0949	0.0211	6.69E-06	20.2287
M35322.x	rs11924406	T	C	0.8796	-0.0692	0.0156	8.67E-06	19.6772
M35322.x	rs12436835	A	G	0.5918	0.0522	0.0114	5.04E-06	20.9668
M35322.x	rs1561164	A	G	0.0405	0.1346	0.03	7.45E-06	20.1302
M35322.x	rs17151588	A	C	0.6597	0.0514	0.011	3.11E-06	21.8344
M35322.x	rs17448763	A	G	0.226	0.0588	0.0132	8.54E-06	19.843
M35322.x	rs2819083	A	G	0.3744	0.059	0.0124	1.96E-06	22.6392

M35322.x	rs3212446	T	C	0.6284	0.0516	0.0111	3.41E-06	21.6099
M35322.x	rs385748	T	C	0.9894	-0.3443	0.0711	1.28E-06	23.4496
M35322.x	rs4751073	T	C	0.9844	-0.3234	0.073	9.43E-06	19.6261
M35322.x	rs6914080	T	C	0.6217	0.056	0.0123	5.02E-06	20.7284
M35322.x	rs724467	T	C	0.2576	0.0602	0.0129	2.92E-06	21.7778
M35322.x	rs748276	A	G	0.0832	0.0879	0.0189	3.35E-06	21.6299
M35322.x	rs905176	A	G	0.3698	-0.0576	0.012	1.5E-06	23.04
M35326.m	rs10155015	A	G	0.3436	-0.0078	0.0017	7.23E-06	21.0519
M35326.m	rs10488759	T	C	0.675	0.0097	0.0019	2.13E-07	26.0637
M35326.m	rs10778622	A	C	0.3766	0.0081	0.0017	3.82E-06	22.7024
M35326.m	rs11188904	A	G	0.6306	0.0082	0.0017	2.77E-06	23.2664
M35326.m	rs11623995	A	G	0.3453	-0.0086	0.0018	9.23E-07	22.8272
M35326.m	rs11653272	T	G	0.721	-0.0087	0.0018	1.05E-06	23.3611
M35326.m	rs1187601	T	C	0.1123	-0.0128	0.0026	1.15E-06	24.2367
M35326.m	rs12049798	A	G	0.1285	0.0132	0.0025	1.99E-07	27.8784
M35326.m	rs12246027	A	G	0.2666	0.0115	0.0018	1.05E-10	40.8179
M35326.m	rs12587899	T	C	0.9619	0.0416	0.009	3.91E-06	21.3649
M35326.m	rs12597210	T	C	0.7036	-0.008	0.0018	5.91E-06	19.7531
M35326.m	rs13004622	A	C	0.5442	0.0079	0.0017	4.07E-06	21.5952
M35326.m	rs13137632	T	C	0.3423	0.0079	0.0017	6.26E-06	21.5952
M35326.m	rs1398096	T	C	0.7377	0.0082	0.0018	4.41E-06	20.7531
M35326.m	rs1571011	A	C	0.5825	-0.0083	0.0017	2.28E-06	23.8374
M35326.m	rs17447104	A	T	0.8434	0.0116	0.0025	4.8E-06	21.5296
M35326.m	rs180106	A	G	0.3045	0.008	0.0018	4.83E-06	19.7531
M35326.m	rs1838137	A	G	0.6843	0.0079	0.0017	6.7E-06	21.5952
M35326.m	rs1882903	C	G	0.2012	0.011	0.0025	6.96E-06	19.36
M35326.m	rs2061282	A	G	0.0735	-0.0171	0.0034	5.26E-07	25.295
M35326.m	rs2149211	A	G	0.435	-0.009	0.002	6.8E-06	20.25
M35326.m	rs2163788	A	C	0.7176	0.008	0.0018	6.49E-06	19.7531
M35326.m	rs2460405	A	G	0.36	-0.0077	0.0017	8.87E-06	20.5156
M35326.m	rs268290	A	C	0.0174	-0.061	0.0136	7.58E-06	20.1179
M35326.m	rs4693661	A	G	0.1547	0.0117	0.0025	3.24E-06	21.9024
M35326.m	rs4765845	A	G	0.4785	0.0081	0.0018	5.57E-06	20.25
M35326.m	rs4803892	T	C	0.7313	-0.0082	0.0018	7.13E-06	20.7531
M35326.m	rs4878377	A	T	0.9346	0.0154	0.0035	8.59E-06	19.36
M35326.m	rs565666	T	C	0.3033	-0.0088	0.0018	5.12E-07	23.9012
M35326.m	rs6054468	T	G	0.0819	-0.0169	0.0036	2.5E-06	22.0378
M35326.m	rs622034	T	C	0.128	0.0141	0.0026	4.2E-08	29.4098
M35326.m	rs6682959	A	T	0.6386	-0.008	0.0018	5.76E-06	19.7531
M35326.m	rs6712429	T	C	0.358	-0.0079	0.0018	6.15E-06	19.2623
M35326.m	rs6912426	A	G	0.3535	-0.0089	0.0017	3.19E-07	27.4083
M35326.m	rs7117598	T	C	0.6692	0.0082	0.0018	3.74E-06	20.7531
M35326.m	rs7327332	T	G	0.6628	-0.0082	0.0017	2.73E-06	23.2664
M35326.m	rs762941	A	G	0.1129	0.0127	0.0026	1.06E-06	23.8595
M35326.m	rs7706802	A	G	0.4621	-0.0189	0.0042	7.06E-06	20.25
M35326.m	rs7897584	A	C	0.7453	0.0079	0.0018	8.92E-06	19.2623
M35326.m	rs924321	T	C	0.305	0.0115	0.0023	5.74E-07	25
M35326.m	rs9290704	T	G	0.5814	-0.0085	0.0017	8.98E-07	25
M35326.m	rs952108	A	G	0.4705	0.0083	0.0018	2.14E-06	21.2623
M35326.m	rs9951062	A	G	0.7122	0.0087	0.0018	1.05E-06	23.3611
M35327.m	rs10025675	T	G	0.1997	-0.0086	0.0018	1.27E-06	22.8272
M35327.m	rs1029481	A	T	0.7306	-0.0089	0.0018	8.81E-07	24.4475
M35327.m	rs11062756	A	G	0.7472	-0.0079	0.0017	5.07E-06	21.5952
M35327.m	rs11100274	A	G	0.8163	-0.009	0.0018	7.74E-07	25
M35327.m	rs11592475	T	C	0.7736	-0.0081	0.0018	5.27E-06	20.25

M35327.m	rs12126220	A	G	0.2807	0.0082	0.0017	3.01E-06	23.2664
M35327.m	rs12571259	T	C	0.0192	-0.0264	0.0058	5.39E-06	20.7182
M35327.m	rs17122683	A	G	0.1379	-0.0216	0.0045	1.47E-06	23.04
M35327.m	rs17654276	A	G	0.9626	-0.0199	0.0043	4.01E-06	21.4175
M35327.m	rs1798613	A	G	0.2257	0.009	0.0018	3.74E-07	25
M35327.m	rs1855534	T	C	0.1952	0.008	0.0018	9.07E-06	19.7531
M35327.m	rs1978526	T	G	0.9681	-0.0213	0.0048	8.69E-06	19.6914
M35327.m	rs2498680	A	G	0.81	0.009	0.002	6.8E-06	20.25
M35327.m	rs340310	A	C	0.6649	-0.0078	0.0017	5.08E-06	21.0519
M35327.m	rs4307594	T	C	0.6911	0.0084	0.0017	9.86E-07	24.4152
M35327.m	rs450852	A	G	0.3243	0.0082	0.0017	1.91E-06	23.2664
M35327.m	rs6477329	A	T	0.6832	0.0087	0.0018	9.49E-07	23.3611
M35327.m	rs731908	A	C	0.193	-0.0085	0.0018	2.73E-06	22.2994
M35327.m	rs7816986	A	G	0.7989	-0.008	0.0018	5.22E-06	19.7531
M35327.m	rs9787087	A	G	0.1893	0.0086	0.0018	1.47E-06	22.8272
M35331.m	rs10108139	T	C	0.6059	0.0142	0.0031	6.45E-06	20.9823
M35331.m	rs10987651	A	G	0.1032	0.0346	0.0077	7.52E-06	20.1916
M35331.m	rs17301396	T	C	0.147	-0.0202	0.0045	7.17E-06	20.1501
M35331.m	rs17305086	T	G	0.178	-0.0181	0.0041	8.21E-06	19.489
M35331.m	rs17815776	A	C	0.9885	-0.1079	0.0238	5.94E-06	20.5537
M35331.m	rs6695423	A	G	0.7148	-0.0146	0.0033	7.48E-06	19.5739
M35331.m	rs764419	T	G	0.1973	-0.0187	0.0041	4.99E-06	20.8025
M35331.m	rs9885127	T	C	0.9868	-0.1433	0.0281	3.32E-07	26.0064
M35397.m	rs10955167	A	G	0.7713	0.0078	0.0017	4.7E-06	21.0519
M35397.m	rs11681627	T	G	0.4034	-0.0076	0.0016	2.99E-06	22.5625
M35397.m	rs12154661	T	C	0.7624	0.0077	0.0017	4.65E-06	20.5156
M35397.m	rs12512765	T	C	0.7813	0.0091	0.0017	9.07E-08	28.654
M35397.m	rs12528756	A	G	0.1278	0.0108	0.0024	6.2E-06	20.25
M35397.m	rs12613833	T	C	0.7597	-0.008	0.0017	1.93E-06	22.1453
M35397.m	rs1877564	T	C	0.7887	0.0081	0.0017	2.2E-06	22.7024
M35397.m	rs1878022	T	C	0.6634	-0.0074	0.0016	6.26E-06	21.3906
M35397.m	rs1943505	T	C	0.1067	0.0113	0.0024	3.2E-06	22.1684
M35397.m	rs1995087	A	G	0.3435	-0.0074	0.0016	6.1E-06	21.3906
M35397.m	rs2075158	T	C	0.3188	-0.0076	0.0016	3.23E-06	22.5625
M35397.m	rs2090103	A	G	0.2395	-0.0086	0.0017	3.37E-07	25.5917
M35397.m	rs2421332	T	C	0.7667	-0.014	0.0031	5.92E-06	20.3954
M35397.m	rs2835977	A	G	0.2484	-0.0076	0.0017	5.23E-06	19.9862
M35397.m	rs2841549	A	G	0.762	-0.0084	0.0017	4.47E-07	24.4152
M35397.m	rs3781638	T	G	0.5337	0.008	0.0016	4.32E-07	25
M35397.m	rs3786099	T	C	0.207	-0.009	0.002	6.8E-06	20.25
M35397.m	rs4269090	T	C	0.226	0.009	0.002	6.8E-06	20.25
M35397.m	rs4690099	T	C	0.2323	0.0079	0.0017	2.72E-06	21.5952
M35397.m	rs4898845	A	G	0.0654	-0.0161	0.0034	2.03E-06	22.423
M35397.m	rs4917204	A	G	0.7723	-0.0077	0.0017	5.08E-06	20.5156
M35397.m	rs572132	A	T	0.9365	-0.025	0.0055	5.17E-06	20.6612
M35397.m	rs662966	C	G	0.416	0.0084	0.0016	1.43E-07	27.5625
M35397.m	rs6892541	A	G	0.8315	0.0166	0.0037	6.54E-06	20.1286
M35397.m	rs6963604	A	G	0.2522	0.0079	0.0017	2.2E-06	21.5952
M35397.m	rs6998531	T	C	0.2087	0.0078	0.0017	4.34E-06	21.0519
M35397.m	rs7190694	C	G	0.2487	-0.0076	0.0017	5.57E-06	19.9862
M35397.m	rs7546483	C	G	0.7027	0.0084	0.0017	6.2E-07	24.4152
M35397.m	rs7551359	A	C	0.7125	0.0087	0.0017	2.43E-07	26.1903
M35397.m	rs7674482	T	C	0.7627	0.0076	0.0017	5.51E-06	19.9862
M35397.m	rs7812354	C	G	0.9508	0.0172	0.0038	6.29E-06	20.4875
M35397.m	rs7988423	T	C	0.9525	0.0267	0.0058	4.53E-06	21.1917

M35431.m	rs10139470	T	G	0.2538	-0.0156	0.0032	1.47E-06	23.7656
M35431.m	rs10484836	T	C	0.0654	0.0208	0.0044	2.98E-06	22.3471
M35431.m	rs10866705	A	C	0.7567	-0.0133	0.0026	3.47E-07	26.1672
M35431.m	rs10896604	A	G	0.1112	0.0162	0.0037	8.9E-06	19.1702
M35431.m	rs11006251	T	C	0.201	0.0129	0.0027	1.55E-06	22.8272
M35431.m	rs11057695	T	C	0.0567	0.0291	0.0062	2.79E-06	22.0294
M35431.m	rs11639506	A	G	0.9736	-0.034	0.0075	5.69E-06	20.5511
M35431.m	rs12477840	T	C	0.1863	0.0122	0.0027	5.89E-06	20.417
M35431.m	rs12929328	C	G	0.8542	-0.0206	0.0046	8.89E-06	20.0548
M35431.m	rs17098575	A	C	0.9522	-0.027	0.0056	1.54E-06	23.2462
M35431.m	rs17145256	A	G	0.0292	0.0312	0.0069	6.58E-06	20.4461
M35431.m	rs1838932	T	C	0.5849	-0.0128	0.0026	5.07E-07	24.2367
M35431.m	rs2367533	T	C	0.9593	0.0263	0.0059	8.01E-06	19.8704
M35431.m	rs2410833	T	C	0.9447	-0.0237	0.0053	8.27E-06	19.9961
M35431.m	rs2552122	A	C	0.6693	0.0115	0.0026	6.29E-06	19.5636
M35431.m	rs2665348	A	C	0.187	-0.0123	0.0027	7.01E-06	20.7531
M35431.m	rs4626843	A	G	0.0341	0.0292	0.0065	7.39E-06	20.1808
M35431.m	rs4648860	T	C	0.1928	0.0124	0.0027	4.72E-06	21.0919
M35431.m	rs4719207	T	C	0.211	-0.014	0.003	3.06E-06	21.7778
M35431.m	rs4730501	A	G	0.239	-0.0121	0.0026	4.18E-06	21.6583
M35431.m	rs662138	C	G	0.8312	0.0187	0.0034	3.65E-08	30.25
M35431.m	rs6764615	C	G	0.8298	0.0159	0.0033	2.17E-06	23.2149
M35431.m	rs715	T	C	0.7163	0.0123	0.0026	2.4E-06	22.3802
M35431.m	rs7946997	T	C	0.2974	-0.0123	0.0026	2.97E-06	22.3802
M35431.m	rs9492253	A	G	0.8019	-0.0119	0.0027	8.62E-06	19.4252
M35431.m	rs9642829	A	G	0.9744	-0.0346	0.0075	3.61E-06	21.2828
M35433.m	rs11180280	A	G	0.9821	0.123	0.0272	6.01E-06	20.449
M35433.m	rs11945259	T	C	0.0592	-0.033	0.0071	3.82E-06	21.6029
M35433.m	rs12896375	A	T	0.1961	0.0205	0.0045	5.72E-06	20.7531
M35433.m	rs1499667	A	G	0.1418	-0.0209	0.0047	8.91E-06	19.7741
M35433.m	rs2270968	T	G	0.2634	0.0307	0.0037	1.31E-16	68.8451
M35433.m	rs274570	T	C	0.2962	0.0224	0.0037	1.78E-09	36.6516
M35433.m	rs6025080	T	C	0.9782	-0.1125	0.0238	2.22E-06	22.3435
M35433.m	rs9405175	A	G	0.178	-0.024	0.0053	5.83E-06	20.5055
M35439.m	rs10003471	T	C	0.112	0.02	0.004	5.73E-07	25
M35439.m	rs10108023	A	G	0.2564	0.0117	0.0026	9.81E-06	20.25
M35439.m	rs10241456	A	G	0.5409	0.0117	0.0026	7.64E-06	20.25
M35439.m	rs1059264	T	C	0.3254	-0.0149	0.0027	5.57E-08	30.454
M35439.m	rs10895511	T	C	0.9515	-0.0532	0.0114	2.9E-06	21.7778
M35439.m	rs10918734	A	T	0.9478	0.0237	0.0052	6.02E-06	20.7726
M35439.m	rs10945649	A	T	0.1286	0.0181	0.0035	2.7E-07	26.7437
M35439.m	rs11085835	T	C	0.2018	0.0154	0.0027	1.04E-08	32.5322
M35439.m	rs11667602	A	C	0.239	-0.015	0.003	5.73E-07	25
M35439.m	rs11780215	A	G	0.8469	0.0157	0.0034	3.67E-06	21.3227
M35439.m	rs12192135	A	G	0.262	-0.0121	0.0026	4.75E-06	21.6583
M35439.m	rs12202023	T	C	0.7878	-0.0137	0.0027	3.91E-07	25.7462
M35439.m	rs12521174	A	G	0.9524	0.0267	0.006	7.27E-06	19.8025
M35439.m	rs12621141	A	G	0.4155	-0.0128	0.0026	9.23E-07	24.2367
M35439.m	rs12625248	T	G	0.8274	-0.0297	0.0066	7.16E-06	20.25
M35439.m	rs12745456	T	C	0.7303	0.0119	0.0027	7.32E-06	19.4252
M35439.m	rs12927959	A	G	0.725	-0.0241	0.0027	7.1E-19	79.6722
M35439.m	rs13375749	T	C	0.7801	0.0297	0.0027	2.8E-28	121
M35439.m	rs1486945	T	G	0.7403	-0.0118	0.0027	8.57E-06	19.1001
M35439.m	rs1524307	T	C	0.4846	0.025	0.0053	1.96E-06	22.2499
M35439.m	rs16837061	A	G	0.7977	0.0119	0.0027	9.78E-06	19.4252

M35439.m	rs17608635	A	G	0.2319	-0.0134	0.0027	5.01E-07	24.631
M35439.m	rs17641971	T	C	0.6608	-0.0252	0.0026	3.35E-22	93.9408
M35439.m	rs1981524	T	C	0.197	0.016	0.0027	3.15E-09	35.1166
M35439.m	rs2070630	C	G	0.2942	-0.0138	0.0026	1.37E-07	28.1716
M35439.m	rs2291449	A	G	0.914	-0.0276	0.0043	1.44E-10	41.1985
M35439.m	rs246234	C	G	0.2958	-0.0199	0.0026	2.56E-14	58.5814
M35439.m	rs3798167	T	G	0.2046	-0.0133	0.0027	7.31E-07	24.2647
M35439.m	rs3809197	T	C	0.0135	0.1072	0.021	3.25E-07	26.0586
M35439.m	rs4804715	A	G	0.7044	0.0134	0.0026	2.85E-07	26.5621
M35439.m	rs4804736	A	G	0.161	-0.018	0.004	6.8E-06	20.25
M35439.m	rs573812	T	C	0.8073	0.0122	0.0027	6.12E-06	20.417
M35439.m	rs6745507	C	G	0.6155	-0.0119	0.0026	3.65E-06	20.9482
M35439.m	rs683369	C	G	0.7735	-0.0142	0.0027	1.07E-07	27.6598
M35439.m	rs715	T	C	0.7174	0.0215	0.0026	3.5E-16	68.3802
M35439.m	rs7633111	T	C	0.5766	-0.0125	0.0026	1.93E-06	23.1139
M35439.m	rs8012	A	G	0.4414	0.0374	0.0027	6.36E-45	191.874
M35439.m	rs8056893	A	C	0.7336	-0.0302	0.0026	2.23E-30	134.917
M35439.m	rs807532	T	C	0.0687	0.0202	0.0045	7.46E-06	20.1501
M35439.m	rs896388	A	G	0.077	0.031	0.0047	3.94E-11	43.5038
M35439.m	rs931068	T	C	0.6556	0.0115	0.0026	9.21E-06	19.5636
M35439.m	rs9573172	A	G	0.9488	-0.0233	0.0053	9.49E-06	19.3268
M35451.m	rs1028074	T	C	0.8585	-0.0838	0.0148	1.52E-08	32.0601
M35451.m	rs10504124	A	G	0.817	-0.0588	0.0127	3.76E-06	21.4362
M35451.m	rs10766596	A	G	0.2938	-0.1244	0.0244	3.26E-07	25.9933
M35451.m	rs11155259	A	G	0.066	-0.0914	0.0201	5.74E-06	20.6776
M35451.m	rs11814841	T	C	0.9778	-0.1595	0.0339	2.48E-06	22.1372
M35451.m	rs12107009	T	C	0.0368	0.1196	0.0268	7.91E-06	19.9156
M35451.m	rs12522706	A	G	0.0337	0.2998	0.0666	6.84E-06	20.2635
M35451.m	rs12795805	T	C	0.7791	0.0524	0.0118	9.68E-06	19.7196
M35451.m	rs12935880	A	G	0.0392	0.381	0.075	3.72E-07	25.8064
M35451.m	rs1495673	A	C	0.3834	-0.056	0.0127	9.76E-06	19.4432
M35451.m	rs17142700	T	C	0.8725	-0.1807	0.0403	7.27E-06	20.1051
M35451.m	rs2209436	T	C	0.0297	0.138	0.0306	6.62E-06	20.3383
M35451.m	rs2663644	A	G	0.0123	0.5247	0.1033	3.81E-07	25.8001
M35451.m	rs2728817	A	T	0.0954	0.1986	0.04	6.95E-07	24.6512
M35451.m	rs2850065	T	C	0.0146	0.5411	0.1066	3.87E-07	25.7656
M35451.m	rs6542859	T	C	0.0339	0.1378	0.0268	2.68E-07	26.438
M35451.m	rs6698967	A	G	0.9723	-0.3177	0.0704	6.49E-06	20.3652
M35451.m	rs7025875	A	T	0.9753	-0.1843	0.0409	6.54E-06	20.305
M35464.m	rs10051810	A	G	0.9747	0.039	0.0088	9.99E-06	19.641
M35464.m	rs10115026	A	G	0.6952	0.0115	0.0025	4.66E-06	21.16
M35464.m	rs12127701	A	G	0.9446	-0.0263	0.0059	9.56E-06	19.8704
M35464.m	rs1479402	T	C	0.0137	0.0525	0.0112	2.83E-06	21.9727
M35464.m	rs17782118	T	C	0.1467	0.0164	0.0034	1.05E-06	23.2664
M35464.m	rs2041073	T	C	0.5366	0.0178	0.0025	3.41E-13	50.6944
M35464.m	rs2067776	T	C	0.5198	0.0118	0.0025	3.41E-06	22.2784
M35464.m	rs2088148	T	G	0.341	0.0123	0.0025	1.08E-06	24.2064
M35464.m	rs2332601	T	C	0.9837	-0.0439	0.0099	9.81E-06	19.6634
M35464.m	rs2592126	A	G	0.4137	-0.0114	0.0025	4.75E-06	20.7936
M35464.m	rs2633279	A	G	0.6235	0.0112	0.0025	6.66E-06	20.0704
M35464.m	rs2973167	A	C	0.7175	0.0137	0.0031	7.29E-06	19.5307
M35464.m	rs398172	A	G	0.3361	-0.0126	0.0025	4.04E-07	25.4016
M35464.m	rs4475212	T	C	0.4307	-0.0109	0.0025	9.59E-06	19.0096
M35464.m	rs4789522	A	C	0.666	0.0119	0.0025	2.08E-06	22.6576
M35464.m	rs806044	T	G	0.6844	-0.0116	0.0025	4.07E-06	21.5296

M35464.m	rs887733	T	C	0.5524	0.0119	0.0024	1.02E-06	24.5851
M35472.m	rs10982735	A	G	0.627	0.023	0.005	4.23E-06	21.16
M35472.m	rs11259235	A	G	0.8943	-0.0347	0.0074	2.39E-06	21.9885
M35472.m	rs11709408	A	G	0.3574	0.0241	0.0052	3.45E-06	21.4797
M35472.m	rs12482287	T	G	0.9753	-0.1167	0.0259	6.59E-06	20.3022
M35472.m	rs13084271	A	G	0.4129	-0.0192	0.0042	5.54E-06	20.898
M35472.m	rs1411737	T	C	0.7834	0.0243	0.0054	5.82E-06	20.25
M35472.m	rs17047839	T	G	0.9243	0.0768	0.0149	2.51E-07	26.5675
M35472.m	rs17280469	T	C	0.2358	-0.0242	0.005	1.31E-06	23.4256
M35472.m	rs17339031	T	C	0.9567	0.0481	0.0108	9.03E-06	19.8355
M35472.m	rs17639286	T	C	0.2643	0.026	0.0058	8.99E-06	20.0951
M35472.m	rs1853936	T	C	0.1776	0.0285	0.0058	7.19E-07	24.1454
M35472.m	rs274567	T	C	0.3704	0.0257	0.0042	1.27E-09	37.4427
M35472.m	rs397175	A	G	0.1373	0.032	0.0066	1.36E-06	23.5078
M35472.m	rs4573205	T	G	0.8691	-0.0308	0.0067	4.33E-06	21.1325
M35472.m	rs6899136	A	G	0.0868	0.0345	0.0077	7.35E-06	20.0751
M35472.m	rs7164415	C	G	0.9749	0.1228	0.0242	3.84E-07	25.7493
M35472.m	rs8031307	A	G	0.8267	-0.052	0.0104	5.14E-07	25
M35472.m	rs9293241	A	G	0.4237	-0.0191	0.0043	7.95E-06	19.7301
M35527.m	rs11932470	A	G	0.4287	0.0361	0.0076	2.42E-06	22.5625
M35527.m	rs12422389	T	C	0.9413	-0.0801	0.018	8.59E-06	19.8025
M35527.m	rs1439737	T	C	0.5832	0.0368	0.0075	1.08E-06	24.0754
M35527.m	rs1551864	T	C	0.3052	-0.038	0.0084	5.9E-06	20.4649
M35527.m	rs1948766	T	G	0.2853	-0.037	0.0083	8.25E-06	19.8723
M35527.m	rs8054116	A	C	0.4278	0.0352	0.008	9.83E-06	19.36
M35527.m	rs9417900	T	C	0.2502	0.0385	0.0085	6.25E-06	20.5156
M35527.m	rs9580319	T	C	0.9296	-0.0641	0.014	4.44E-06	20.9633
M35527.x	rs10806924	A	G	0.5678	0.0204	0.0045	7.57E-06	20.5511
M35527.x	rs10894263	A	C	0.5633	0.025	0.0055	4.82E-06	20.6612
M35527.x	rs12470898	A	G	0.4536	-0.0209	0.0045	3.44E-06	21.5709
M35527.x	rs13002542	C	G	0.3638	-0.0205	0.0046	7.75E-06	19.8606
M35527.x	rs2221389	T	G	0.1341	-0.0293	0.0064	5.29E-06	20.9592
M35527.x	rs4587865	A	G	0.0675	0.0428	0.0088	1.27E-06	23.655
M35527.x	rs7147228	T	C	0.4575	0.0201	0.0045	7.94E-06	19.9511
M35527.x	rs752299	A	C	0.7326	0.0256	0.0053	1.64E-06	23.3307
M35527.x	rs7954059	T	C	0.4298	0.0207	0.0046	7.69E-06	20.25
M35527.x	rs9311289	A	G	0.2369	-0.0255	0.0055	3.35E-06	21.4959
M35527.x	rs9322446	A	G	0.1323	-0.0312	0.007	7.2E-06	19.8661
M35551.m	rs10082970	T	C	0.1881	-0.0451	0.0088	2.99E-07	26.2656
M35551.m	rs10818386	T	C	0.1967	0.0395	0.0089	8.68E-06	19.6976
M35551.m	rs1159296	T	C	0.7444	-0.061	0.0135	6.6E-06	20.417
M35551.m	rs12189736	T	C	0.8963	-0.0642	0.0114	1.56E-08	31.7147
M35551.m	rs12527313	A	C	0.0471	0.0738	0.0167	9.64E-06	19.529
M35551.m	rs17167889	T	C	0.9594	-0.0852	0.0176	1.3E-06	23.4344
M35551.m	rs215934	T	C	0.1585	0.043	0.0093	3.58E-06	21.3782
M35551.m	rs4841110	A	G	0.5869	0.0342	0.0073	2.6E-06	21.9486
M35551.m	rs7171302	C	G	0.2838	-0.0438	0.0083	1.54E-07	27.8479
M35551.m	rs7326215	T	C	0.8718	0.0495	0.0103	1.49E-06	23.096
M35551.m	rs9784679	T	C	0.2789	0.0363	0.008	6.2E-06	20.5889
M35551.m	rs9849918	A	C	0.6538	0.0363	0.0073	7.74E-07	24.7268
M35626.m	rs1493534	A	C	0.8645	0.0219	0.0048	4.97E-06	20.8164
M35626.m	rs2490288	T	C	0.3284	0.0163	0.0037	8.06E-06	19.4076
M35626.m	rs249619	T	C	0.5624	-0.0319	0.0072	8.87E-06	19.6298
M35626.m	rs3813782	A	G	0.1154	0.0535	0.0118	5.81E-06	20.5562
M35626.m	rs7710231	T	C	0.7247	-0.0168	0.0038	9.63E-06	19.5457

M35626.m	rs780093	T	C	0.3958	0.019	0.0036	1.33E-07	27.8549
M35628.m	rs10468017	T	C	0.2863	0.0164	0.0037	8.06E-06	19.6465
M35628.m	rs10759726	A	G	0.4548	0.0163	0.0036	5.79E-06	20.5008
M35628.m	rs1260326	T	C	0.4073	0.0178	0.0035	4.51E-07	25.8645
M35628.m	rs12918754	A	G	0.5774	0.0163	0.0036	5.89E-06	20.5008
M35628.m	rs1470121	A	G	0.3619	0.0167	0.0035	2.36E-06	22.7665
M35628.m	rs2070895	A	G	0.2092	0.0266	0.0044	1.1E-09	36.5475
M35628.m	rs2179170	T	C	0.1945	0.0194	0.0044	9.67E-06	19.4401
M35628.m	rs2199680	A	T	0.8332	-0.0218	0.0044	8.49E-07	24.5475
M35628.m	rs7583683	A	G	0.8612	0.021	0.0047	7.24E-06	19.9638
M35628.m	rs9657544	C	G	0.702	-0.016	0.0036	9.23E-06	19.7531
M35631.m	rs10089452	A	C	0.5399	-0.0126	0.0028	6.53E-06	20.25
M35631.m	rs10468017	T	C	0.2879	0.0187	0.0027	3.33E-12	47.9684
M35631.m	rs1260326	T	C	0.4052	0.0139	0.0026	9.31E-08	28.5814
M35631.m	rs12805521	T	C	0.985	0.0999	0.022	5.86E-06	20.6199
M35631.m	rs1533302	A	G	0.4472	-0.014	0.0026	1.09E-07	28.9941
M35631.m	rs17109340	A	G	0.9737	-0.0884	0.0197	7.28E-06	20.1359
M35631.m	rs179070	A	G	0.4773	0.0119	0.0027	7.43E-06	19.4252
M35631.m	rs184943	A	T	0.7036	-0.0119	0.0026	7.23E-06	20.9482
M35631.m	rs2070895	A	G	0.208	0.0297	0.0034	1.36E-18	76.3054
M35631.m	rs2161134	A	G	0.6204	0.0117	0.0026	6.93E-06	20.25
M35631.m	rs2179170	T	C	0.1941	0.0155	0.0034	5.02E-06	20.7829
M35631.m	rs2278476	T	C	0.4871	0.0119	0.0026	5E-06	20.9482
M35631.m	rs2305779	A	G	0.1237	0.0196	0.0042	2.79E-06	21.7778
M35631.m	rs2477833	T	C	0.1056	-0.0201	0.0044	4.81E-06	20.8683
M35631.m	rs2606621	A	G	0.2991	-0.013	0.0027	9.59E-07	23.1824
M35631.m	rs2762662	A	G	0.0601	-0.0577	0.0115	5.19E-07	25.1742
M35631.m	rs4315100	T	C	0.6818	-0.0117	0.0027	9.51E-06	18.7778
M35631.m	rs4459648	A	T	0.6753	-0.0119	0.0027	6.64E-06	19.4252
M35631.m	rs4687841	A	G	0.6302	0.0122	0.0026	3.34E-06	22.0178
M35631.m	rs4812575	A	G	0.3039	0.012	0.0027	6.39E-06	19.7531
M35631.m	rs6799366	T	C	0.3135	0.0123	0.0026	3.5E-06	22.3802
M35631.m	rs6897246	A	G	0.3294	0.0118	0.0027	9.29E-06	19.1001
M35631.m	rs7290374	A	G	0.4047	0.0125	0.0027	3.64E-06	21.4335
M35631.m	rs7403602	T	C	0.5361	-0.0117	0.0026	7.15E-06	20.25
M35631.m	rs7706198	A	G	0.1782	-0.0344	0.0075	4.55E-06	21.0375
M35631.m	rs7966920	T	C	0.834	-0.0154	0.0035	9.02E-06	19.36
M35631.m	rs937725	T	C	0.3218	-0.0134	0.0027	4.08E-07	24.631
M35631.m	rs9854668	A	C	0.702	0.0125	0.0026	2.52E-06	23.1139
M35635.m	rs12461874	A	C	0.2704	0.0569	0.0121	2.7E-06	22.1133
M35635.m	rs12480837	A	G	0.0804	-0.081	0.0173	2.89E-06	21.9219
M35635.m	rs12537257	A	G	0.1088	0.0912	0.0194	2.48E-06	22.0997
M35635.m	rs17059417	A	G	0.3922	0.1061	0.0227	2.86E-06	21.8464
M35635.m	rs2459791	T	C	0.4055	0.0552	0.0121	5.24E-06	20.8117
M35635.m	rs3846659	C	G	0.1357	0.0691	0.0155	8.64E-06	19.8743
M35635.m	rs4796899	T	G	0.098	0.093	0.0201	3.55E-06	21.4079
M35635.m	rs6500859	A	T	0.699	-0.0511	0.0111	4.56E-06	21.1932
M35635.m	rs7110135	C	G	0.6932	0.0523	0.0113	4E-06	21.4213
M35635.m	rs883595	A	G	0.8953	-0.1659	0.0341	1.14E-06	23.6692
M35669.m	rs10505870	A	G	0.2706	0.0245	0.0055	9.3E-06	19.843
M35669.m	rs11045821	A	G	0.1644	-0.0737	0.0065	4.6E-30	128.561
M35669.m	rs12228798	T	C	0.2736	-0.031	0.0055	2.26E-08	31.7686
M35669.m	rs12317268	A	G	0.8342	-0.1008	0.0062	4.16E-60	264.325
M35669.m	rs12485842	T	C	0.0771	0.102	0.0224	5.4E-06	20.735
M35669.m	rs13221063	A	G	0.8289	-0.0294	0.0064	4.78E-06	21.1025

M35669.m	rs16967085	C	G	0.3734	-0.0207	0.0046	6.34E-06	20.25
M35669.m	rs17244095	A	C	0.1095	-0.0439	0.0095	4E-06	21.3541
M35669.m	rs17564907	A	G	0.9315	-0.045	0.0097	3.97E-06	21.5219
M35669.m	rs17661642	C	G	0.9412	-0.0441	0.0097	5.57E-06	20.6697
M35669.m	rs1910186	T	G	0.877	-0.0321	0.0072	8.9E-06	19.8767
M35669.m	rs1988344	T	C	0.9117	-0.0362	0.0081	7.95E-06	19.9732
M35669.m	rs2127434	A	G	0.7956	0.0284	0.0061	3.23E-06	21.6759
M35669.m	rs2554887	A	T	0.683	0.0282	0.0053	1.29E-07	28.3104
M35669.m	rs2566347	A	C	0.2063	-0.0262	0.0056	2.44E-06	21.889
M35669.m	rs2857468	A	T	0.0729	-0.054	0.0104	1.93E-07	26.9601
M35669.m	rs4149028	T	C	0.9319	0.0477	0.0096	6.5E-07	24.6885
M35669.m	rs4551320	T	G	0.0759	0.0434	0.0093	2.88E-06	21.7778
M35669.m	rs4799429	T	C	0.48	-0.0213	0.0046	3.27E-06	21.4409
M35669.m	rs6663731	A	T	0.8857	0.0624	0.0081	1.39E-14	59.3471
M35669.m	rs7023732	A	G	0.0811	0.0369	0.0083	8.07E-06	19.765
M35669.m	rs704622	T	C	0.9863	-0.3467	0.0712	1.13E-06	23.7109
M35669.m	rs7233515	A	G	0.3974	-0.0211	0.0046	4.74E-06	21.0402
M35669.m	rs8192287	T	G	0.0436	0.0522	0.0115	5.84E-06	20.6037
M35669.m	rs9262	C	G	0.5925	-0.0226	0.0046	1.01E-06	24.138
M35675.m	rs10180218	A	T	0.3776	-0.0085	0.0019	4.78E-06	20.0139
M35675.m	rs10203537	A	T	0.6315	0.0082	0.0019	8.75E-06	18.626
M35675.m	rs10841406	A	G	0.3964	-0.0092	0.0018	4.94E-07	26.1235
M35675.m	rs11731427	C	G	0.9058	0.0169	0.0035	1.69E-06	23.3151
M35675.m	rs11988968	T	C	0.7043	-0.0086	0.0019	4.15E-06	20.4875
M35675.m	rs12151923	T	C	0.7465	-0.0087	0.0019	3.53E-06	20.9668
M35675.m	rs1235145	T	C	0.4209	-0.0081	0.0018	9.08E-06	20.25
M35675.m	rs12476705	C	G	0.2856	-0.0084	0.0019	6.38E-06	19.5457
M35675.m	rs12698117	A	G	0.3013	0.0085	0.0018	4.15E-06	22.2994
M35675.m	rs13059174	T	C	0.2947	0.0085	0.0019	5.02E-06	20.0139
M35675.m	rs13073301	A	G	0.5896	0.0086	0.0018	2.7E-06	22.8272
M35675.m	rs1470254	A	G	0.2515	-0.0084	0.0019	6.77E-06	19.5457
M35675.m	rs1547984	T	G	0.6311	0.0201	0.0045	6.43E-06	19.9511
M35675.m	rs158896	T	C	0.2658	0.0089	0.0019	1.38E-06	21.9418
M35675.m	rs1608132	T	C	0.2504	0.0084	0.0019	6.61E-06	19.5457
M35675.m	rs16999682	C	G	0.9821	-0.0344	0.0076	6.03E-06	20.4875
M35675.m	rs17391668	A	G	0.1367	0.0128	0.0027	2.26E-06	22.4746
M35675.m	rs1864480	T	C	0.7711	-0.0137	0.0026	1.57E-07	27.7648
M35675.m	rs1880030	A	G	0.4317	-0.0082	0.0018	5.81E-06	20.7531
M35675.m	rs2167730	T	C	0.6541	-0.0087	0.0018	2.32E-06	23.3611
M35675.m	rs2177207	T	C	0.9801	-0.0927	0.0191	1.21E-06	23.5555
M35675.m	rs2285688	A	G	0.705	0.0097	0.0019	1.82E-07	26.0637
M35675.m	rs2344567	T	G	0.2843	0.0225	0.005	6.35E-06	20.25
M35675.m	rs25846	T	C	0.3109	-0.009	0.0019	1.62E-06	22.4377
M35675.m	rs2811692	T	C	0.3247	0.0084	0.0019	9.47E-06	19.5457
M35675.m	rs2901027	A	T	0.341	-0.0085	0.0018	4.1E-06	22.2994
M35675.m	rs4132537	A	G	0.4952	-0.0098	0.0018	8.29E-08	29.642
M35675.m	rs4482740	A	G	0.3507	0.0084	0.0018	4.53E-06	21.7778
M35675.m	rs4643071	T	C	0.6782	-0.0092	0.0018	5.07E-07	26.1235
M35675.m	rs4670599	A	G	0.5558	-0.0088	0.0018	1.29E-06	23.9012
M35675.m	rs4935623	A	G	0.4782	-0.0086	0.0018	2.28E-06	22.8272
M35675.m	rs4984992	C	G	0.6116	0.0081	0.0018	7.83E-06	20.25
M35675.m	rs6075489	A	G	0.7179	0.0094	0.0019	5.03E-07	24.4765
M35675.m	rs6133587	T	G	0.719	0.009	0.002	6.8E-06	20.25
M35675.m	rs6564157	T	C	0.4074	-0.0087	0.0018	2.12E-06	23.3611
M35675.m	rs679959	A	G	0.3159	0.0087	0.0019	2.79E-06	20.9668

M35675.m	rs6851835	A	G	0.5236	0.0081	0.0018	8.89E-06	20.25
M35675.m	rs7152842	A	G	0.7508	0.0092	0.0019	6.13E-07	23.446
M35675.m	rs7223436	A	G	0.2539	-0.0087	0.0019	3.37E-06	20.9668
M35675.m	rs7253352	A	G	0.4862	0.0084	0.0019	7.98E-06	19.5457
M35675.m	rs728634	T	C	0.03	0.0556	0.0125	8.86E-06	19.7847
M35675.m	rs742914	A	C	0.7235	-0.0083	0.0018	7.46E-06	21.2623
M35675.m	rs7930371	T	C	0.3408	-0.0096	0.0018	1.82E-07	28.4444
M35675.m	rs9404466	T	C	0.2671	0.0105	0.0018	1.26E-08	34.0278
M35675.m	rs9410293	A	T	0.7111	0.0089	0.0019	4.67E-06	21.9418
M35675.m	rs9541642	A	G	0.6955	-0.0082	0.0018	8.36E-06	20.7531
M35675.m	rs9543004	T	C	0.6185	-0.0095	0.0018	2.16E-07	27.8549
M35675.m	rs9602121	T	C	0.6481	-0.0088	0.0018	1.86E-06	23.9012
M35678.m	rs10505870	A	G	0.2707	0.0252	0.0053	1.89E-06	22.6073
M35678.m	rs10770126	A	G	0.5547	0.0205	0.0044	2.68E-06	21.7071
M35678.m	rs10869648	A	G	0.4086	-0.0211	0.0044	1.37E-06	22.9964
M35678.m	rs11045818	A	G	0.1612	-0.073	0.0061	1.61E-32	143.214
M35678.m	rs11720619	T	C	0.2508	0.0233	0.0051	4.86E-06	20.8724
M35678.m	rs12070845	A	G	0.0702	0.0414	0.0089	3.16E-06	21.6382
M35678.m	rs12228798	T	C	0.2742	-0.0311	0.0053	3.27E-09	34.4325
M35678.m	rs12464103	A	G	0.1572	0.0297	0.006	8.24E-07	24.5025
M35678.m	rs12714232	T	C	0.5232	-0.02	0.0044	7.07E-06	20.6612
M35678.m	rs13293473	C	G	0.9111	-0.037	0.0076	1.3E-06	23.7015
M35678.m	rs1578700	T	C	0.1191	0.0303	0.0068	8.45E-06	19.8549
M35678.m	rs17644535	C	G	0.9834	-0.2588	0.0515	4.99E-07	25.2531
M35678.m	rs1871395	A	G	0.8326	-0.0893	0.0059	1.65E-52	229.086
M35678.m	rs2286411	A	G	0.1197	0.0327	0.0073	8.31E-06	20.0655
M35678.m	rs2857468	A	T	0.082	-0.0615	0.0094	6.06E-11	42.805
M35678.m	rs3805895	A	C	0.9764	0.0645	0.0145	8.57E-06	19.7872
M35678.m	rs4972811	T	C	0.428	0.0204	0.0044	4.22E-06	21.4959
M35678.m	rs6663731	A	T	0.8823	0.0452	0.0069	5.49E-11	42.912
M35678.m	rs6870033	A	T	0.7179	-0.0238	0.0052	4.14E-06	20.9482
M35678.m	rs7023732	A	G	0.0795	0.0396	0.0079	6.35E-07	25.1267
M35678.m	rs7265772	A	G	0.1918	0.024	0.0054	9.36E-06	19.7531
M35678.m	rs749008	A	G	0.6386	-0.0197	0.0044	7.65E-06	20.046
M35678.m	rs7523522	T	C	0.6548	-0.0213	0.0044	1.32E-06	23.4344
M35678.m	rs7926241	A	G	0.0152	0.0867	0.0187	3.56E-06	21.4959
M35678.m	rs9304337	A	G	0.5538	0.0206	0.0044	2.26E-06	21.9194
M35678.m	rs9487538	A	G	0.0402	0.0504	0.0113	7.67E-06	19.8932
M35678.m	rs9587762	T	C	0.187	0.0273	0.0061	7.77E-06	20.0293
M35718.m	rs10418189	T	C	0.2672	-0.0116	0.0026	8.3E-06	19.9053
M35718.m	rs10842318	T	G	0.1288	0.0155	0.0034	5.84E-06	20.7829
M35718.m	rs11636541	T	G	0.1055	0.0192	0.0042	3.99E-06	20.898
M35718.m	rs1195226	T	C	0.8642	0.0154	0.0035	8.15E-06	19.36
M35718.m	rs12573896	A	G	0.9175	0.0196	0.0044	7.04E-06	19.843
M35718.m	rs13247156	T	C	0.9195	-0.0196	0.0044	7.78E-06	19.843
M35718.m	rs1326611	A	T	0.6424	0.0114	0.0026	9.67E-06	19.2249
M35718.m	rs1477025	T	C	0.6548	0.0131	0.0026	4.8E-07	25.3861
M35718.m	rs1491024	A	G	0.7762	-0.0127	0.0027	2.89E-06	22.1248
M35718.m	rs174455	A	G	0.6459	-0.0147	0.0026	1.51E-08	31.966
M35718.m	rs1752812	A	G	0.755	-0.0125	0.0026	2.11E-06	23.1139
M35718.m	rs1816995	A	G	0.7712	0.0264	0.0058	5.86E-06	20.7182
M35718.m	rs1899398	T	C	0.799	0.0123	0.0027	4.94E-06	20.7531
M35718.m	rs4978408	A	G	0.066	-0.0243	0.0052	3.11E-06	21.8376
M35718.m	rs509360	A	G	0.332	-0.0127	0.0027	2.75E-06	22.1248
M35718.m	rs6498540	A	G	0.7042	0.0172	0.0026	2.55E-11	43.7633

M35718.m	rs6957801	A	G	0.0198	0.0443	0.0094	2.37E-06	22.2102
M35718.m	rs7142493	T	C	0.7195	-0.0129	0.0026	1.11E-06	24.6169
M35718.m	rs732647	A	G	0.0598	0.0246	0.0051	1.49E-06	23.2664
M35718.m	rs7615671	T	G	0.6922	0.0253	0.0053	1.52E-06	22.7871
M35718.m	rs7748665	A	G	0.9206	-0.0212	0.0043	6.71E-07	24.3072
M35718.m	rs7784712	T	C	0.0761	0.0197	0.0044	7E-06	20.046
M35718.m	rs908259	A	G	0.2156	0.0119	0.0027	8.69E-06	19.4252
M35718.m	rs9373682	T	C	0.2404	-0.0128	0.0026	1.15E-06	24.2367
M35718.m	rs968567	T	C	0.1796	0.0329	0.0035	2.14E-21	88.36
M35754.m	rs11004433	T	C	0.8602	0.0128	0.0029	9.05E-06	19.4816
M35754.m	rs11008861	A	G	0.4749	-0.0092	0.0019	1.53E-06	23.446
M35754.m	rs12022877	A	G	0.8244	-0.0403	0.0087	4.17E-06	21.4571
M35754.m	rs12145429	A	G	0.3757	0.0099	0.0019	2.22E-07	27.1496
M35754.m	rs12259030	A	G	0.1453	0.014	0.0028	9.07E-07	25
M35754.m	rs1256061	T	G	0.4673	-0.0085	0.0019	9.28E-06	20.0139
M35754.m	rs12570090	T	C	0.5847	-0.0094	0.0019	9.18E-07	24.4765
M35754.m	rs12969887	A	C	0.5532	0.0085	0.0019	9.5E-06	20.0139
M35754.m	rs13228785	A	C	0.4235	-0.0089	0.0019	3.32E-06	21.9418
M35754.m	rs165558	T	C	0.5657	-0.0091	0.0019	2E-06	22.9391
M35754.m	rs16962157	T	C	0.9219	-0.0192	0.0038	4.57E-07	25.5291
M35754.m	rs1722784	A	G	0.5093	-0.0091	0.0019	2.4E-06	22.9391
M35754.m	rs1869578	A	G	0.5791	-0.0086	0.0019	7.61E-06	20.4875
M35754.m	rs2041264	T	C	0.9069	-0.0179	0.0038	2.53E-06	22.1891
M35754.m	rs2216327	C	G	0.9359	-0.0246	0.0055	8.26E-06	20.0053
M35754.m	rs2273068	T	C	0.1355	-0.0127	0.0029	8.87E-06	19.1784
M35754.m	rs2683822	T	C	0.6125	-0.009	0.0019	3.46E-06	22.4377
M35754.m	rs2708257	A	C	0.6508	-0.0108	0.0019	2.17E-08	32.3102
M35754.m	rs3110907	C	G	0.8988	-0.0199	0.0037	1.04E-07	28.927
M35754.m	rs4641528	T	C	0.5683	0.0094	0.0019	9.44E-07	24.4765
M35754.m	rs4792953	T	C	0.3951	0.0094	0.0019	8.92E-07	24.4765
M35754.m	rs4852787	A	C	0.4642	-0.0101	0.002	2.86E-07	25.5025
M35754.m	rs4968679	T	C	0.4992	-0.009	0.0019	3.09E-06	22.4377
M35754.m	rs6475283	T	C	0.2824	0.0343	0.0075	5.12E-06	20.9154
M35754.m	rs6486785	A	G	0.0193	0.0371	0.0082	5.99E-06	20.4701
M35754.m	rs6827956	A	G	0.4111	0.0089	0.0019	3.66E-06	21.9418
M35754.m	rs6828013	T	C	0.0729	0.017	0.0038	8.79E-06	20.0139
M35754.m	rs6832838	T	C	0.5177	0.0098	0.0019	3.36E-07	26.6039
M35754.m	rs6895134	C	G	0.011	0.057	0.012	2.03E-06	22.5625
M35754.m	rs6991001	T	C	0.0961	0.017	0.0037	5.03E-06	21.1103
M35754.m	rs7036147	T	C	0.4125	-0.009	0.0019	2.86E-06	22.4377
M35754.m	rs7039918	T	C	0.8007	0.0132	0.0028	2.76E-06	22.2245
M35754.m	rs7147975	A	G	0.4965	-0.01	0.0019	2.44E-07	27.7008
M35754.m	rs7520333	A	G	0.554	-0.0091	0.0019	2.3E-06	22.9391
M35754.m	rs7814471	A	G	0.4642	-0.0092	0.0019	1.78E-06	23.446
M35754.m	rs7862610	A	C	0.4276	0.0093	0.0019	1.36E-06	23.9584
M35754.m	rs791320	T	C	0.5496	-0.009	0.0019	2.57E-06	22.4377
M35754.m	rs8103504	T	C	0.5809	0.0092	0.0019	1.62E-06	23.446
M35754.m	rs819909	A	G	0.5236	0.0088	0.0019	4.24E-06	21.4515
M35754.m	rs884957	A	G	0.6492	-0.0085	0.0019	9.54E-06	20.0139
M35754.m	rs939358	T	C	0.4477	-0.0086	0.0019	8.16E-06	20.4875
M35754.m	rs9563725	T	C	0.4437	-0.0087	0.0019	6.02E-06	20.9668
M35754.m	rs9605002	A	G	0.1	-0.0178	0.0039	4.24E-06	20.831
M35754.m	rs970719	T	C	0.4248	0.0087	0.0019	6.44E-06	20.9668
M35854.m	rs10108610	A	G	0.138	-0.0464	0.0104	8.01E-06	19.9053
M35854.m	rs12147158	T	C	0.9406	0.0378	0.0085	9.49E-06	19.7763

M35854.m	rs12451101	T	C	0.0806	-0.059	0.013	5.77E-06	20.5976
M35854.m	rs1961334	A	G	0.4773	-0.0198	0.0037	6.03E-08	28.637
M35854.m	rs3785236	A	C	0.2425	-0.0173	0.0038	5.25E-06	20.7265
M35854.m	rs422249	T	C	0.309	-0.017	0.0037	5.44E-06	21.1103
M35854.m	rs4669658	T	C	0.3738	0.0163	0.0037	8.14E-06	19.4076
M35854.m	rs4880258	A	G	0.5404	0.0164	0.0037	8.1E-06	19.6465
M35854.m	rs6730977	A	G	0.6251	0.0177	0.0037	1.38E-06	22.8846
M35854.m	rs7586148	T	C	0.5142	-0.0324	0.0071	5.53E-06	20.8244
M35854.m	rs7863959	T	C	0.5363	0.0165	0.0037	6.99E-06	19.8868
M35854.m	rs917046	T	G	0.9531	-0.0369	0.0082	6.71E-06	20.25
M35854.m	rs9960323	T	C	0.2721	0.0178	0.0038	2.37E-06	21.9418
M35977.m	rs11053152	T	C	0.012	0.102	0.021	1.19E-06	23.5918
M35977.m	rs12449543	T	C	0.4462	0.016	0.0036	8.25E-06	19.7531
M35977.m	rs12547884	A	G	0.7663	-0.0175	0.0039	6.32E-06	20.1348
M35977.m	rs12623684	A	G	0.0629	0.0334	0.0071	2.59E-06	22.1297
M35977.m	rs1728918	A	G	0.3054	0.0265	0.0054	8.14E-07	24.0826
M35977.m	rs2068834	T	C	0.7218	-0.0168	0.0037	4.61E-06	20.6165
M35977.m	rs4567273	A	C	0.2608	0.0251	0.0056	8.97E-06	20.0896
M35977.m	rs4719788	T	C	0.0317	-0.0926	0.0202	4.59E-06	21.0145
M35977.m	rs6582090	T	G	0.745	-0.0168	0.0038	7.42E-06	19.5457
M35977.m	rs9831949	T	G	0.0277	-0.0693	0.0134	2.38E-07	26.7459
M35978.m	rs1022302	T	C	0.3866	-0.0223	0.005	9.91E-06	19.8916
M35978.m	rs10267258	T	C	0.8786	-0.0305	0.0067	4.71E-06	20.7229
M35978.m	rs10433495	T	C	0.9624	0.055	0.0122	6.67E-06	20.3238
M35978.m	rs11184443	T	C	0.2471	0.025	0.0056	8.83E-06	19.9298
M35978.m	rs12681727	A	G	0.1333	-0.0357	0.008	7.14E-06	19.9139
M35978.m	rs12778305	T	G	0.37	0.0278	0.0063	8.9E-06	19.4719
M35978.m	rs1418811	T	G	0.8452	0.0275	0.0061	5.93E-06	20.3238
M35978.m	rs1556831	T	G	0.7471	0.0226	0.0051	9.19E-06	19.6371
M35978.m	rs16928398	T	C	0.9707	0.0623	0.0136	4.57E-06	20.9845
M35978.m	rs16936190	C	G	0.0247	-0.1014	0.0223	5.41E-06	20.676
M35978.m	rs2921448	A	G	0.2771	0.0227	0.005	5.25E-06	20.6116
M35978.m	rs3789107	A	C	0.8931	0.0636	0.0133	1.9E-06	22.8671
M35978.m	rs4665383	C	G	0.7261	-0.0254	0.0051	5.49E-07	24.8043
M35978.m	rs4854073	T	C	0.4114	-0.0224	0.0046	1.25E-06	23.7127
M35978.m	rs6751559	T	G	0.8962	-0.0357	0.0071	5.48E-07	25.2825
M35978.m	rs6899809	T	C	0.0436	-0.0554	0.0123	7.2E-06	20.2866
M35978.m	rs7619989	C	G	0.602	-0.0308	0.0068	5.82E-06	20.5156
M35978.m	rs9877943	A	G	0.2282	0.0244	0.0054	5.99E-06	20.417
M36009.m	rs1035798	A	G	0.2645	-0.0276	0.0062	9.19E-06	19.8169
M36009.m	rs11603772	A	G	0.269	-0.0292	0.0062	2.32E-06	22.1811
M36009.m	rs11760565	T	C	0.1482	-0.0356	0.008	8.13E-06	19.8025
M36009.m	rs1185800	T	C	0.8867	-0.0408	0.0086	2.28E-06	22.5073
M36009.m	rs12622653	A	G	0.7765	-0.0314	0.0067	2.89E-06	21.9639
M36009.m	rs16951745	A	G	0.1023	0.0449	0.009	6.09E-07	24.889
M36009.m	rs1836049	T	G	0.9791	-0.0926	0.0205	6.48E-06	20.404
M36009.m	rs2243168	A	T	0.9071	0.0524	0.0104	4.88E-07	25.3861
M36009.m	rs2467285	T	C	0.6632	0.0266	0.006	9.54E-06	19.6544
M36009.m	rs4351	A	G	0.4739	-0.0343	0.0053	1.19E-10	41.8829
M36009.m	rs6060489	A	C	0.0753	0.0524	0.0115	4.89E-06	20.7619
M36009.m	rs6124466	T	C	0.8283	-0.0359	0.0073	7.67E-07	24.1848
M36009.m	rs743745	T	G	0.1734	0.0319	0.007	6E-06	20.7676
M36009.m	rs7697282	T	C	0.9785	0.0997	0.0192	1.95E-07	26.9642
M36095.x	rs10765034	T	C	0.1892	-0.0798	0.016	6.34E-07	24.8752
M36095.x	rs2163880	A	G	0.9321	0.1237	0.0259	1.78E-06	22.8108

M36095.x	rs5765217	A	G	0.2914	-0.0573	0.0122	2.73E-06	22.0592
M36095.x	rs7092545	A	C	0.1856	0.1246	0.028	8.62E-06	19.8025
M36095.x	rs8021829	C	G	0.9613	-0.1757	0.0394	8.29E-06	19.8862
M36097.x	rs11670792	T	C	0.8964	0.5691	0.1157	8.77E-07	24.1942
M36097.x	rs11697457	A	G	0.9745	1.0751	0.243	9.64E-06	19.5743
M36097.x	rs11792920	A	G	0.1274	-0.4893	0.1084	6.39E-06	20.3747
M36097.x	rs12005871	A	T	0.8885	0.5499	0.1218	6.39E-06	20.3832
M36097.x	rs12199693	A	T	0.7393	0.4221	0.092	4.5E-06	21.0501
M36097.x	rs12650232	T	G	0.9469	0.5126	0.1139	6.82E-06	20.254
M36097.x	rs12822507	A	G	0.6722	0.26	0.0583	8.14E-06	19.8888
M36097.x	rs12886515	A	T	0.8922	0.3885	0.0876	9.19E-06	19.6686
M36097.x	rs13235582	T	C	0.227	0.3204	0.0695	4.04E-06	21.2528
M36097.x	rs1479400	T	C	0.8279	0.5062	0.094	7.19E-08	28.9994
M36097.x	rs162882	C	G	0.9132	0.685	0.1362	4.95E-07	25.2945
M36097.x	rs16870710	A	C	0.0674	-0.5095	0.1061	1.55E-06	23.0599
M36097.x	rs16878933	A	T	0.0909	-0.444	0.0928	1.74E-06	22.8913
M36097.x	rs16897568	T	C	0.8063	0.2875	0.0638	6.7E-06	20.3065
M36097.x	rs17126976	A	G	0.8598	0.4543	0.0966	2.59E-06	22.1173
M36097.x	rs1865661	A	T	0.9836	1.4423	0.2889	5.95E-07	24.9239
M36097.x	rs2956731	T	G	0.931	0.4689	0.0934	5.18E-07	25.2038
M36097.x	rs4374185	T	C	0.5959	-0.3303	0.0727	5.53E-06	20.6418
M36097.x	rs4387456	A	G	0.9863	1.6505	0.3371	9.78E-07	23.9725
M36097.x	rs6080482	A	G	0.2177	-0.2681	0.0605	9.48E-06	19.6373
M36097.x	rs6829571	A	G	0.7541	-0.4173	0.0905	3.95E-06	21.2618
M36097.x	rs6956438	A	G	0.8991	-0.4219	0.0917	4.2E-06	21.168
M36097.x	rs7794551	T	G	0.8542	0.4628	0.1047	9.81E-06	19.5386
M36097.x	rs7950414	A	G	0.0385	-0.7051	0.1581	8.25E-06	19.8901
M36097.x	rs8101059	A	G	0.4901	0.2683	0.0582	4.05E-06	21.2518
M36097.x	rs873413	T	C	0.5579	0.3898	0.0824	2.27E-06	22.3784
M36097.x	rs882014	A	G	0.0175	-1.0082	0.2226	5.94E-06	20.5137
M36097.x	rs9586074	A	C	0.9835	1.3057	0.2944	9.2E-06	19.6703
M36097.x	rs9787093	A	G	0.0167	-1.323	0.2938	6.69E-06	20.2776
M36097.x	rs9961807	T	C	0.2187	-0.32	0.0638	5.35E-07	25.157
M36098.x	rs1165165	T	C	0.2392	0.0439	0.0079	2.37E-08	30.8798
M36098.x	rs12021584	A	G	0.3846	0.0311	0.0069	6E-06	20.3153
M36098.x	rs12189736	T	C	0.8964	-0.0505	0.0105	1.52E-06	23.1315
M36098.x	rs2399250	A	G	0.8481	0.0416	0.0093	8.23E-06	20.0088
M36098.x	rs2581383	C	G	0.6655	0.0364	0.0079	3.72E-06	21.2299
M36098.x	rs2674357	C	G	0.8039	-0.0775	0.0166	2.96E-06	21.7965
M36098.x	rs4141381	T	G	0.985	-0.144	0.032	6.8E-06	20.25
M36098.x	rs4322806	C	G	0.037	0.0813	0.0174	2.94E-06	21.8315
M36098.x	rs6552978	A	G	0.808	-0.0735	0.0162	5.7E-06	20.5847
M36098.x	rs791232	T	C	0.491	0.031	0.0069	7.63E-06	20.1848
M36098.x	rs9616173	T	C	0.0924	-0.0518	0.0113	4.33E-06	21.0137
M36099.x	rs11931868	A	T	0.7962	0.0441	0.0099	7.79E-06	19.843
M36099.x	rs12412942	A	G	0.5839	-0.0363	0.0082	8.68E-06	19.5968
M36099.x	rs1248821	T	C	0.2512	-0.038	0.0085	7.1E-06	19.9862
M36099.x	rs16864115	T	C	0.0154	0.1474	0.0325	5.88E-06	20.5697
M36099.x	rs17294478	T	C	0.148	0.069	0.013	1.11E-07	28.1716
M36099.x	rs3935503	T	G	0.7581	-0.0398	0.0085	2.66E-06	21.9244
M36099.x	rs951918	A	G	0.8704	0.0546	0.0119	4.67E-06	21.0519
M36103.m	rs10511749	A	T	0.9606	0.068	0.0142	1.73E-06	22.932
M36103.m	rs12579447	A	T	0.0531	0.0536	0.0121	9.59E-06	19.6227
M36103.m	rs13098279	A	G	0.203	-0.0305	0.0068	7.48E-06	20.1179
M36103.m	rs1358498	T	C	0.5824	-0.024	0.0053	5.01E-06	20.5055

M36103.m	rs16984516	A	G	0.9848	0.202	0.0419	1.41E-06	23.2421
M36103.m	rs17125342	T	C	0.0709	-0.0526	0.011	1.61E-06	22.8658
M36103.m	rs2072901	A	G	0.8764	0.0392	0.0083	2.47E-06	22.3057
M36103.m	rs2280042	A	G	0.0129	-0.2177	0.0462	2.48E-06	22.2041
M36103.m	rs2488856	A	C	0.9066	0.08	0.018	8.64E-06	19.7531
M36103.m	rs2819047	A	G	0.2125	-0.0373	0.0069	5.82E-08	29.2226
M36103.m	rs478259	A	G	0.858	-0.0393	0.0078	5.27E-07	25.3861
M36103.m	rs727145	T	C	0.3735	0.0231	0.0052	9.99E-06	19.7341
M36103.m	rs727276	T	C	0.9671	0.1628	0.0341	1.73E-06	22.7929
M36103.m	rs7332616	T	C	0.7928	-0.032	0.007	5.18E-06	20.898
M36103.m	rs9348951	A	C	0.4014	0.0239	0.0052	5.05E-06	21.1246
M36115.m	rs11079672	T	C	0.9555	-0.0819	0.018	5.27E-06	20.7025
M36115.m	rs12494751	A	C	0.1264	0.0429	0.0094	4.94E-06	20.8285
M36115.m	rs12617093	T	C	0.4512	-0.0348	0.0076	4.81E-06	20.9668
M36115.m	rs13105073	A	T	0.9779	0.1236	0.0261	2.16E-06	22.4262
M36115.m	rs16992910	A	G	0.0528	0.0646	0.0141	4.79E-06	20.9907
M36115.m	rs2357258	A	C	0.6387	-0.037	0.0079	2.54E-06	21.9356
M36115.m	rs4351	A	G	0.475	-0.0581	0.006	3.64E-22	93.7669
M36115.m	rs463106	T	C	0.6825	0.0367	0.0081	6.29E-06	20.5287
M36115.m	rs4968748	A	G	0.2526	-0.0363	0.0071	3.34E-07	26.1395
M36115.m	rs651007	T	C	0.2089	-0.0461	0.0075	9.58E-10	37.7815
M36115.m	rs7229244	A	C	0.7276	-0.0328	0.0072	5.67E-06	20.7531
M36115.m	rs7992673	T	C	0.922	0.161	0.035	4.23E-06	21.16
M36115.m	rs9479657	T	C	0.0711	-0.053	0.0118	7.75E-06	20.1738
M36131.m	rs10738470	T	C	0.1772	0.0335	0.0074	6.34E-06	20.494
M36131.m	rs10764178	A	T	0.9265	-0.0579	0.0119	1.22E-06	23.6735
M36131.m	rs11773897	T	G	0.4957	0.0276	0.0062	7.51E-06	19.8169
M36131.m	rs12256082	A	G	0.9155	0.0559	0.0124	6.69E-06	20.3226
M36131.m	rs12599099	T	C	0.0768	0.0581	0.0131	9.32E-06	19.6702
M36131.m	rs13105073	A	T	0.9767	-0.1302	0.0227	9.18E-09	32.8981
M36131.m	rs1411176	T	C	0.3811	-0.0359	0.0074	1.28E-06	23.5356
M36131.m	rs16959731	T	C	0.1032	-0.0484	0.0106	5.56E-06	20.8487
M36131.m	rs16973170	T	C	0.8753	-0.0383	0.0086	8.62E-06	19.8336
M36131.m	rs2068007	T	C	0.9124	0.0374	0.0082	5.49E-06	20.8025
M36131.m	rs2254145	A	G	0.2138	-0.0316	0.0069	4.29E-06	20.9737
M36131.m	rs2881913	A	C	0.449	0.0315	0.0057	3.99E-08	30.5402
M36131.m	rs2955245	T	C	0.3085	-0.0346	0.0064	6.39E-08	29.2275
M36131.m	rs4351	A	G	0.4732	-0.0437	0.0057	1.17E-14	58.7778
M36131.m	rs4403944	T	G	0.0458	-0.0638	0.0134	2.04E-06	22.669
M36131.m	rs4486099	A	C	0.1884	-0.0391	0.0086	5.79E-06	20.6708
M36131.m	rs890554	C	G	0.6895	-0.0283	0.0062	5.75E-06	20.8348
M36134.m	rs10973519	T	C	0.3511	-0.0296	0.0067	9.78E-06	19.5179
M36134.m	rs12266302	A	C	0.955	-0.1028	0.0188	4.71E-08	29.9
M36134.m	rs12404009	A	G	0.9894	0.2036	0.0434	2.74E-06	22.0078
M36134.m	rs1889157	A	G	0.4713	-0.0342	0.0076	6.44E-06	20.25
M36134.m	rs4362	T	C	0.5281	0.0612	0.0064	1.37E-21	91.4414
M36134.m	rs4897622	A	G	0.1147	-0.0574	0.0125	4.1E-06	21.0865
M36134.m	rs4954715	A	G	0.0853	-0.0527	0.0111	2.28E-06	22.5411
M36134.m	rs6089829	A	G	0.3531	0.0294	0.0064	4.75E-06	21.1025
M36134.m	rs651007	T	C	0.2089	-0.0407	0.0077	1.46E-07	27.9388
M36134.m	rs7099968	T	C	0.0466	-0.0556	0.0122	4.66E-06	20.7697
M36134.m	rs7187550	T	C	0.1217	0.0473	0.0101	2.67E-06	21.9321
M36134.m	rs7474722	T	C	0.9636	-0.0757	0.0166	5.29E-06	20.7958
M36134.m	rs7812350	T	C	0.7854	0.0344	0.0076	6.29E-06	20.4875
M36134.m	rs7980973	T	G	0.7914	-0.0381	0.0078	1.1E-06	23.8595

M36230.m	rs11066661	A	C	0.4127	-0.0297	0.0063	2.18E-06	22.2245
M36230.m	rs1106806	A	C	0.3883	-0.0284	0.0063	6.78E-06	20.3215
M36230.m	rs11079672	T	C	0.9546	-0.0993	0.0185	8.17E-08	28.8108
M36230.m	rs13025191	C	G	0.8014	-0.0364	0.0081	6.72E-06	20.1945
M36230.m	rs13105073	A	T	0.9793	0.1256	0.0281	8.01E-06	19.9787
M36230.m	rs17121435	A	G	0.9807	0.1236	0.0256	1.36E-06	23.3108
M36230.m	rs1909355	T	C	0.0954	-0.0385	0.0086	8.54E-06	20.0412
M36230.m	rs205208	T	C	0.096	-0.0495	0.0107	4.11E-06	21.4014
M36230.m	rs2278161	T	C	0.7661	-0.0341	0.0073	3.25E-06	21.8204
M36230.m	rs297192	T	G	0.4024	0.031	0.0068	4.54E-06	20.7829
M36230.m	rs35060330	T	C	0.3289	0.0356	0.008	9.45E-06	19.8025
M36230.m	rs4351	A	G	0.4635	-0.0529	0.0063	3.11E-17	70.5067
M36230.m	rs4949966	T	C	0.874	0.0468	0.0092	3.56E-07	25.8771
M36230.m	rs6070840	A	C	0.2034	0.0397	0.0084	2.48E-06	22.3369
M36230.m	rs6124466	T	C	0.828	-0.04	0.0085	2.36E-06	22.1453
M36230.m	rs649129	T	C	0.2141	-0.0437	0.0077	1.26E-08	32.2093
M36230.m	rs6533515	A	G	0.2112	-0.035	0.0078	7.04E-06	20.1348
M36230.m	rs779741	A	C	0.574	-0.0285	0.0064	8.21E-06	19.8303
M36230.m	rs7840544	A	G	0.9725	0.0947	0.0208	5.39E-06	20.7288
M36230.m	rs7981197	A	G	0.9196	-0.0512	0.0115	8.07E-06	19.8219
M36230.m	rs9574536	T	C	0.3565	-0.0517	0.0113	5.06E-06	20.9326
M36300.m	rs10894263	A	C	0.5535	0.0161	0.0035	4.84E-06	21.16
M36300.m	rs10899916	T	C	0.3837	0.0244	0.0051	2.07E-06	22.8897
M36300.m	rs11585904	C	G	0.1296	0.0219	0.0049	9.23E-06	19.9754
M36300.m	rs11670375	A	G	0.864	-0.0208	0.0044	2.71E-06	22.3471
M36300.m	rs1374273	A	G	0.6234	0.0254	0.0052	1.05E-06	23.8595
M36300.m	rs1449969	A	G	0.7512	-0.0156	0.0034	5.28E-06	21.0519
M36300.m	rs1557339	A	C	0.7675	0.0275	0.006	4.42E-06	21.0069
M36300.m	rs4654649	T	C	0.5518	0.0144	0.0032	8.49E-06	20.25
M36300.m	rs6913176	T	C	0.9804	0.082	0.0177	3.8E-06	21.4625
M36376.m	rs1192458	A	G	0.6223	0.034	0.0071	1.89E-06	22.932
M36376.m	rs1893285	A	G	0.5927	-0.0331	0.0072	4.81E-06	21.1345
M36376.m	rs2174099	C	G	0.1469	0.0471	0.01	2.6E-06	22.1841
M36376.m	rs2874702	T	C	0.4189	-0.0496	0.0096	2.47E-07	26.6944
M36376.m	rs7021014	T	G	0.7224	0.0334	0.0074	6.59E-06	20.3718
M36399.m	rs1075106	A	C	0.7777	0.0371	0.0083	8.52E-06	19.9798
M36399.m	rs10889714	T	C	0.3474	0.0216	0.0047	4.74E-06	21.1209
M36399.m	rs11613221	C	G	0.8706	0.0334	0.0072	4.05E-06	21.5193
M36399.m	rs12771324	A	T	0.9842	-0.0928	0.0202	4.43E-06	21.1054
M36399.m	rs13100602	T	G	0.3761	-0.0219	0.0047	3.14E-06	21.7116
M36399.m	rs13125176	C	G	0.1863	-0.0406	0.0089	5.47E-06	20.81
M36399.m	rs1469456	T	C	0.7562	-0.0263	0.0054	1.36E-06	23.7205
M36399.m	rs1893625	A	T	0.2284	-0.0238	0.0054	9.86E-06	19.4252
M36399.m	rs2216028	A	G	0.3782	-0.0209	0.0047	7.82E-06	19.7741
M36399.m	rs2311717	A	T	0.6829	0.0364	0.0075	1.3E-06	23.5548
M36399.m	rs2842707	C	G	0.399	0.057	0.012	2.03E-06	22.5625
M36399.m	rs4986894	T	C	0.838	-0.0421	0.0061	5.55E-12	47.6326
M36399.m	rs967942	T	C	0.2883	0.0255	0.0049	1.64E-07	27.0825
M36468.m	rs10753990	A	C	0.3849	-0.0326	0.0069	2.5E-06	22.3222
M36468.m	rs11786174	A	C	0.1501	-0.0519	0.0112	3.61E-06	21.4733
M36468.m	rs11927423	A	G	0.9586	0.08	0.0175	4.77E-06	20.898
M36468.m	rs12962966	T	C	0.17	0.0453	0.0095	1.83E-06	22.7378
M36468.m	rs12966200	A	T	0.8443	0.0434	0.0095	5.3E-06	20.8705
M36468.m	rs12974040	T	C	0.2168	-0.0503	0.0103	1.14E-06	23.8485
M36468.m	rs1479073	T	C	0.7131	0.035	0.0077	5.97E-06	20.6612

M36468.m	rs151316	T	C	0.183	0.082	0.017	1.41E-06	23.2664
M36468.m	rs1783235	A	G	0.7188	0.0347	0.0077	6.59E-06	20.3085
M36468.m	rs2074881	T	C	0.1936	0.0574	0.0121	1.95E-06	22.5037
M36468.m	rs2404867	A	C	0.7977	0.053	0.0114	3.14E-06	21.6143
M36468.m	rs327759	T	C	0.2319	0.042	0.0086	1.02E-06	23.8507
M36468.m	rs3846241	A	G	0.404	-0.0478	0.0105	5.75E-06	20.7242
M36468.m	rs6999153	A	G	0.5597	0.0313	0.0069	5.97E-06	20.5774
M36468.m	rs7211140	A	G	0.4598	0.0358	0.0074	1.41E-06	23.4047
M36468.m	rs802524	T	C	0.9577	0.0875	0.0182	1.47E-06	23.1139
M36515.m	rs12626113	T	C	0.2385	-0.0058	0.0012	9.78E-07	23.3611
M36515.m	rs12630256	T	C	0.0162	-0.0262	0.0056	3.09E-06	21.889
M36515.m	rs169291	C	G	0.7729	0.0052	0.001	3.1E-07	27.04
M36515.m	rs17087080	T	C	0.975	0.0143	0.0032	8.47E-06	19.9697
M36515.m	rs1849217	T	C	0.8178	0.007	0.0014	8.98E-07	25
M36515.m	rs2839579	A	G	0.312	0.0043	0.0009	4.45E-06	22.8272
M36515.m	rs2850161	T	G	0.3664	0.0047	0.001	4.84E-06	22.09
M36515.m	rs6541210	A	G	0.4489	-0.004	0.0009	8.42E-06	19.7531
M36515.m	rs6860185	T	C	0.8821	-0.0079	0.0018	6.51E-06	19.2623
M36515.m	rs8184882	T	C	0.6224	0.0043	0.0009	7.27E-06	22.8272
M36552.m	rs11711582	A	G	0.4815	-0.0077	0.0017	5.19E-06	20.5156
M36552.m	rs11730219	T	C	0.495	-0.0078	0.0017	3.42E-06	21.0519
M36552.m	rs1279831	A	G	0.5589	0.0077	0.0017	4.74E-06	20.5156
M36552.m	rs12823314	T	C	0.0408	-0.0221	0.0047	2.28E-06	22.11
M36552.m	rs1537987	T	C	0.5417	0.0076	0.0017	5.79E-06	19.9862
M36552.m	rs16985567	T	C	0.5111	-0.0139	0.0031	8.32E-06	20.1051
M36552.m	rs17516635	A	G	0.017	0.055	0.0123	7.49E-06	19.9947
M36552.m	rs2028012	T	C	0.1636	-0.0123	0.0024	4.78E-07	26.2656
M36552.m	rs2383896	A	G	0.523	0.0076	0.0017	6.77E-06	19.9862
M36552.m	rs2615697	T	C	0.8166	-0.0108	0.0024	7.72E-06	20.25
M36552.m	rs2997247	T	C	0.8295	0.0115	0.0024	1.77E-06	22.9601
M36552.m	rs329033	A	C	0.8429	-0.0133	0.0029	4.22E-06	21.0333
M36552.m	rs4621813	T	C	0.184	0.0107	0.0024	7.42E-06	19.8767
M36552.m	rs4684437	T	C	0.158	-0.012	0.0025	1.34E-06	23.04
M36552.m	rs4782451	A	C	0.4897	-0.0078	0.0017	4.05E-06	21.0519
M36552.m	rs6535384	A	G	0.5194	0.0078	0.0017	4.14E-06	21.0519
M36552.m	rs7037029	A	G	0.7501	0.0108	0.0024	7.21E-06	20.25
M36552.m	rs7251886	T	C	0.4441	-0.0075	0.0017	9.47E-06	19.4637
M36552.m	rs7822232	A	C	0.9203	0.0299	0.0038	5.12E-15	61.912
M36552.m	rs7910605	A	G	0.876	-0.0138	0.003	5.45E-06	21.16
M36553.m	rs10770797	T	C	0.6906	0.0181	0.0029	3.81E-10	38.9548
M36553.m	rs11045834	T	C	0.2885	-0.0232	0.0029	8.62E-16	64
M36553.m	rs11581618	A	G	0.3437	-0.013	0.0029	6.59E-06	20.0951
M36553.m	rs12317268	A	G	0.8428	-0.0536	0.0038	2.2E-45	198.958
M36553.m	rs1434665	A	T	0.7194	-0.0129	0.0029	7.91E-06	19.7872
M36553.m	rs17145556	T	C	0.951	-0.033	0.0067	7.79E-07	24.2593
M36553.m	rs17651844	A	G	0.031	0.0441	0.0092	1.52E-06	22.9774
M36553.m	rs17832807	C	G	0.1092	0.0233	0.0048	1.55E-06	23.5629
M36553.m	rs1910202	A	G	0.968	-0.1449	0.032	5.82E-06	20.5039
M36553.m	rs2581320	T	G	0.8846	0.0214	0.0047	6.2E-06	20.7316
M36553.m	rs3763941	T	C	0.047	0.0336	0.0076	9E-06	19.5457
M36553.m	rs3790310	A	T	0.8295	0.0177	0.0038	3.46E-06	21.696
M36553.m	rs4610008	T	C	0.6607	-0.0562	0.0123	5.2E-06	20.8767
M36553.m	rs4657463	C	G	0.6945	0.0136	0.0029	2.8E-06	21.9929
M36553.m	rs4762693	A	G	0.2859	0.0172	0.0037	4.07E-06	21.6099
M36553.m	rs594084	T	C	0.6304	-0.0131	0.0029	5.55E-06	20.4055

M36553.m	rs6563831	T	C	0.652	0.0136	0.0029	2.33E-06	21.9929
M36553.m	rs6771638	T	G	0.2911	-0.0132	0.0029	5.38E-06	20.7182
M36553.m	rs6859143	A	G	0.7032	0.0134	0.0029	3.55E-06	21.3508
M36553.m	rs7499206	T	C	0.29	0.014	0.0029	1.4E-06	23.3056
M36553.m	rs7978273	T	C	0.9008	0.0214	0.0048	7.93E-06	19.8767
M36553.m	rs948847	T	G	0.5518	-0.0134	0.0029	3.01E-06	21.3508
M36559.m	rs11045510	T	C	0.1391	-0.0379	0.0077	8.66E-07	24.2269
M36559.m	rs11722928	T	C	0.8291	-0.0307	0.0066	3.74E-06	21.6366
M36559.m	rs11921520	A	G	0.1844	-0.0303	0.0066	4.99E-06	21.0764
M36559.m	rs12281635	A	G	0.6214	0.023	0.0052	8.97E-06	19.5636
M36559.m	rs12726438	T	C	0.1121	0.037	0.0083	8.65E-06	19.8723
M36559.m	rs13159652	T	C	0.2052	0.031	0.0067	3.34E-06	21.4079
M36559.m	rs13255137	T	C	0.0791	0.0441	0.0095	3.24E-06	21.5491
M36559.m	rs13408475	A	G	0.1647	0.0594	0.0127	2.98E-06	21.8759
M36559.m	rs1439977	T	C	0.3281	-0.0254	0.0052	1.1E-06	23.8595
M36559.m	rs17112260	T	C	0.9839	-0.1723	0.0384	7.2E-06	20.133
M36559.m	rs17152472	T	C	0.0133	0.2502	0.05	5.58E-07	25.04
M36559.m	rs17163287	A	C	0.7217	-0.0273	0.006	4.61E-06	20.7025
M36559.m	rs1871395	A	G	0.8221	-0.0319	0.0066	1.13E-06	23.3611
M36559.m	rs2954636	T	C	0.1715	0.0317	0.0068	3.18E-06	21.7321
M36559.m	rs4795411	A	G	0.9339	-0.0466	0.0104	6.9E-06	20.0773
M36559.m	rs6938391	A	G	0.0262	0.0802	0.0165	1.19E-06	23.6255
M36559.m	rs7574665	A	G	0.9858	-0.2023	0.0432	2.79E-06	21.9293
M36559.m	rs9922902	C	G	0.0778	0.0635	0.0141	6.45E-06	20.2819
M36559.m	rs9931063	A	G	0.8768	0.0395	0.0086	4.32E-06	21.0959
M36585.m	rs10916130	T	C	0.8277	-0.0401	0.0087	3.86E-06	21.2447
M36585.m	rs13142346	T	G	0.2825	0.0339	0.0069	8.46E-07	24.138
M36585.m	rs1681886	A	G	0.7189	0.0311	0.0069	6.37E-06	20.3153
M36585.m	rs17366517	A	G	0.9343	-0.0584	0.0128	4.75E-06	20.8164
M36585.m	rs2130048	T	G	0.9023	-0.051	0.011	3.45E-06	21.4959
M36585.m	rs2499690	A	G	0.9593	-0.0766	0.0167	4.37E-06	21.039
M36585.m	rs4074801	T	C	0.5824	-0.0296	0.0067	8.48E-06	19.5179
M36585.m	rs6138327	C	G	0.98	-0.1123	0.0242	3.52E-06	21.5342
M36585.m	rs6761802	A	G	0.3637	-0.0334	0.0067	6.53E-07	24.851
M36585.m	rs7220100	A	G	0.8574	-0.044	0.0098	7.5E-06	20.1583
M36585.m	rs9399651	C	G	0.8405	0.0389	0.0086	5.64E-06	20.4598
M36589.m	rs10131962	A	G	0.6038	-0.0295	0.0064	4.05E-06	21.2463
M36589.m	rs10459871	C	G	0.9167	-0.0559	0.0118	2.28E-06	22.4419
M36589.m	rs10951634	A	C	0.4616	0.0288	0.0063	5.68E-06	20.898
M36589.m	rs11688377	T	C	0.4786	-0.0655	0.0144	5.31E-06	20.6899
M36589.m	rs12412323	T	C	0.9827	-0.117	0.0264	9.63E-06	19.641
M36589.m	rs16945428	T	C	0.1006	-0.0497	0.0113	9.92E-06	19.3444
M36589.m	rs17102948	A	G	0.9519	0.0836	0.0164	3.51E-07	25.9851
M36589.m	rs1965169	T	C	0.1956	0.0848	0.0186	4.89E-06	20.7858
M36589.m	rs1990049	A	G	0.9439	-0.0753	0.0169	8.84E-06	19.8526
M36589.m	rs2251480	T	G	0.7426	0.0375	0.0085	9.84E-06	19.4637
M36589.m	rs2512378	T	C	0.4444	0.0314	0.0071	8.48E-06	19.5588
M36589.m	rs2824447	A	G	0.6792	-0.035	0.0072	1.21E-06	23.6304
M36589.m	rs4447447	A	G	0.047	0.2522	0.0526	1.65E-06	22.9889
M36589.m	rs4490450	A	G	0.6949	0.0346	0.0072	1.47E-06	23.0934
M36589.m	rs4715354	A	G	0.4812	0.0296	0.0063	3.04E-06	22.0751
M36589.m	rs749275	A	C	0.1139	0.0495	0.0107	3.54E-06	21.4014
M36589.m	rs7666614	A	G	0.1545	-0.0463	0.0097	1.81E-06	22.7834
M36589.m	rs7969341	A	G	0.8289	-0.0479	0.0081	2.9E-09	34.9704
M36589.m	rs877682	T	C	0.9622	0.0795	0.0177	7E-06	20.1738

M36673.m	rs10045813	A	G	0.2081	0.0235	0.0045	2.27E-07	27.2716
M36673.m	rs11698602	A	G	0.828	-0.0235	0.0047	6.7E-07	25
M36673.m	rs12680421	T	C	0.2899	0.0176	0.0039	7.77E-06	20.3655
M36673.m	rs13155413	A	C	0.2859	0.0177	0.004	9.98E-06	19.5806
M36673.m	rs1390370	A	G	0.8174	0.0235	0.005	2.62E-06	22.09
M36673.m	rs1554057	A	G	0.9059	0.0295	0.0062	1.78E-06	22.6392
M36673.m	rs17573425	A	G	0.1497	0.0237	0.005	1.7E-06	22.4676
M36673.m	rs1878640	T	C	0.911	0.0303	0.0067	5.41E-06	20.452
M36673.m	rs3935974	A	G	0.8298	-0.0208	0.0047	9.41E-06	19.5853
M36673.m	rs4846407	T	C	0.3452	-0.0175	0.0039	5.6E-06	20.1348
M36673.m	rs4931556	A	G	0.8633	-0.0236	0.0053	9.39E-06	19.8277
M36673.m	rs659974	A	G	0.1642	0.0235	0.0051	4.61E-06	21.2322
M36738.m	rs10145517	A	G	0.7599	-0.0373	0.0079	2.46E-06	22.2927
M36738.m	rs11044362	C	G	0.9662	0.0982	0.0204	1.41E-06	23.172
M36738.m	rs12098538	T	C	0.0626	0.0642	0.0124	2.21E-07	26.8057
M36738.m	rs12143405	T	G	0.2971	-0.0328	0.0073	6.05E-06	20.1884
M36738.m	rs1491633	A	G	0.0765	0.059	0.0118	5.48E-07	25
M36738.m	rs17249529	T	G	0.3177	0.0322	0.0066	1.22E-06	23.8026
M36738.m	rs1889603	A	G	0.747	-0.086	0.019	6E-06	20.4875
M36738.m	rs1959363	T	C	0.1197	-0.0447	0.0094	2.06E-06	22.6131
M36738.m	rs4930211	T	G	0.9025	0.0477	0.0103	3.3E-06	21.4468
M36738.m	rs690111	T	G	0.2596	-0.037	0.0075	8.3E-07	24.3378
M36754.m	rs11578466	C	G	0.8688	-0.0251	0.0056	8.4E-06	20.0896
M36754.m	rs11733714	T	C	0.0461	-0.0704	0.0149	2.47E-06	22.324
M36754.m	rs11852566	T	C	0.1197	-0.0253	0.0057	7.91E-06	19.7011
M36754.m	rs12829704	A	G	0.1927	-0.042	0.0045	9.49E-21	87.1111
M36754.m	rs17618395	A	G	0.5998	0.0162	0.0035	5.1E-06	21.4237
M36754.m	rs1871395	A	G	0.8367	-0.0309	0.005	8.68E-10	38.1924
M36754.m	rs2108622	T	C	0.288	-0.0248	0.0044	1.2E-08	31.7686
M36754.m	rs2161480	C	G	0.9525	0.07	0.0157	8.79E-06	19.8791
M36754.m	rs2857468	A	T	0.0922	-0.0389	0.0071	4.71E-08	30.0181
M36754.m	rs359428	A	G	0.0344	-0.0471	0.01	2.44E-06	22.1841
M36754.m	rs3794989	T	C	0.3285	0.0165	0.0036	4.95E-06	21.0069
M36754.m	rs631871	A	G	0.5197	-0.017	0.0036	1.76E-06	22.2994
M36756.m	rs10425651	A	G	0.9597	-0.0522	0.0114	4.96E-06	20.9668
M36756.m	rs1054812	A	G	0.6283	-0.0203	0.0044	4.28E-06	21.2856
M36756.m	rs10794603	A	G	0.4222	-0.0195	0.0044	8.14E-06	19.641
M36756.m	rs11674647	C	G	0.1049	-0.0381	0.0075	3.46E-07	25.8064
M36756.m	rs13060187	T	C	0.5699	-0.0217	0.0044	8.17E-07	24.3228
M36756.m	rs1433673	A	G	0.3426	0.0428	0.0094	5.33E-06	20.7316
M36756.m	rs1942651	T	C	0.6705	-0.0242	0.0051	2.14E-06	22.516
M36756.m	rs2076562	T	C	0.3736	-0.0219	0.0044	6.36E-07	24.7732
M36756.m	rs2081670	T	C	0.1437	0.0284	0.0063	6.77E-06	20.3215
M36756.m	rs518385	A	G	0.5033	-0.0203	0.0044	3.29E-06	21.2856
M36756.m	rs7048966	C	G	0.1755	0.0278	0.006	3.71E-06	21.4678
M36776.m	rs10064153	A	C	0.7322	-0.0119	0.0026	3.78E-06	20.9482
M36776.m	rs10501675	A	C	0.0254	-0.0346	0.007	7.05E-07	24.4318
M36776.m	rs11593054	T	G	0.2479	0.0123	0.0025	1.12E-06	24.2064
M36776.m	rs11627218	A	T	0.7766	0.013	0.0026	6.46E-07	25
M36776.m	rs12217848	A	G	0.287	0.0118	0.0026	5.74E-06	20.5976
M36776.m	rs12509356	C	G	0.161	0.018	0.004	6.8E-06	20.25
M36776.m	rs1428365	T	C	0.9228	-0.0192	0.0042	4.52E-06	20.898
M36776.m	rs16750	A	G	0.8674	-0.015	0.0034	7.59E-06	19.4637
M36776.m	rs2146548	T	G	0.7266	0.012	0.0027	7E-06	19.7531
M36776.m	rs2625530	A	G	0.2039	0.0123	0.0026	2.41E-06	22.3802

M36776.m	rs6755317	A	G	0.9729	0.0357	0.0075	2.03E-06	22.6576
M36776.m	rs7225312	A	T	0.8749	-0.0162	0.0035	3.16E-06	21.4237
M36776.m	rs9369302	T	C	0.7183	0.0118	0.0026	4.15E-06	20.5976
M36776.m	rs9830523	T	C	0.9753	-0.0346	0.0076	5.7E-06	20.7265
M36776.m	rs9953686	T	C	0.8128	0.0118	0.0026	6.93E-06	20.5976
M36776.m	rs9954696	A	T	0.7848	0.0121	0.0027	5.36E-06	20.0837
M36802.m	rs10870198	A	G	0.613	-0.019	0.0041	3.7E-06	21.4753
M36802.m	rs11253967	T	C	0.8515	0.0192	0.0043	7.98E-06	19.9373
M36802.m	rs17286243	A	G	0.7403	0.0197	0.0043	4.25E-06	20.9892
M36802.m	rs17631300	C	G	0.0174	0.0637	0.0143	8.13E-06	19.843
M36802.m	rs190756	A	G	0.2755	-0.0167	0.0036	3.37E-06	21.5193
M36802.m	rs327837	A	G	0.376	0.0142	0.0031	4.52E-06	20.9823
M36802.m	rs4729705	T	C	0.539	0.0138	0.003	5.06E-06	21.16
M36802.m	rs4976017	T	C	0.0472	0.031	0.0069	7.79E-06	20.1848
M36802.m	rs7035656	C	G	0.107	-0.0303	0.0068	7.33E-06	19.8549
M36802.m	rs8031716	T	C	0.555	-0.0145	0.003	1.45E-06	23.3611
M36808.m	rs10494658	T	G	0.8351	-0.0087	0.0018	2.04E-06	23.3611
M36808.m	rs10791748	A	C	0.0747	0.0132	0.0027	9.72E-07	23.9012
M36808.m	rs11199575	A	G	0.2001	0.0088	0.0018	8.7E-07	23.9012
M36808.m	rs11232962	T	G	0.2475	0.0176	0.0037	1.6E-06	22.6267
M36808.m	rs11638718	A	G	0.7783	0.0081	0.0018	4.62E-06	20.25
M36808.m	rs11679051	T	C	0.7521	0.0081	0.0018	4.14E-06	20.25
M36808.m	rs11745804	T	G	0.2292	-0.0079	0.0018	8.79E-06	19.2623
M36808.m	rs12563233	T	G	0.1915	-0.0079	0.0018	9.2E-06	19.2623
M36808.m	rs12582388	T	C	0.0454	0.036	0.0078	3.64E-06	21.3018
M36808.m	rs13022471	A	G	0.8262	0.0081	0.0018	6.5E-06	20.25
M36808.m	rs13099034	A	C	0.181	0.0083	0.0018	4.09E-06	21.2623
M36808.m	rs17087080	T	C	0.9749	-0.0429	0.0092	3.12E-06	21.744
M36808.m	rs1756740	A	G	0.2327	0.0077	0.0017	9.16E-06	20.5156
M36808.m	rs1850396	A	T	0.251	-0.008	0.0018	4.67E-06	19.7531
M36808.m	rs2062632	T	C	0.7848	0.008	0.0018	7.49E-06	19.7531
M36808.m	rs2305929	A	G	0.819	0.0084	0.0018	2.62E-06	21.7778
M36808.m	rs27852	A	C	0.2959	-0.0083	0.0018	2.18E-06	21.2623
M36808.m	rs300585	A	G	0.3107	0.0084	0.0017	1.19E-06	24.4152
M36808.m	rs3014614	T	G	0.922	-0.014	0.003	3.06E-06	21.7778
M36808.m	rs37379	A	G	0.0867	0.0147	0.0027	3.43E-08	29.642
M36808.m	rs3797510	C	G	0.8426	-0.0085	0.0018	2.71E-06	22.2994
M36808.m	rs4518383	A	G	0.8068	-0.0083	0.0018	3.89E-06	21.2623
M36808.m	rs4654403	T	C	0.0556	0.0168	0.0038	8.51E-06	19.5457
M36808.m	rs510573	T	G	0.6641	-0.0085	0.0017	9.11E-07	25
M36808.m	rs6107895	A	G	0.1405	-0.0249	0.0046	6.18E-08	29.301
M36808.m	rs6118296	A	G	0.0777	0.0131	0.0027	1.02E-06	23.5405
M36808.m	rs6538186	T	C	0.2552	0.0091	0.0018	1.95E-07	25.5586
M36808.m	rs6688105	T	C	0.703	-0.0079	0.0017	4.86E-06	21.5952
M36808.m	rs6774259	A	G	0.829	-0.0082	0.0018	5.13E-06	20.7531
M36808.m	rs6886404	A	C	0.7536	0.008	0.0018	5.24E-06	19.7531
M36808.m	rs7129047	A	C	0.1751	-0.0081	0.0018	7.21E-06	20.25
M36808.m	rs7519669	A	G	0.1935	0.0082	0.0018	4.37E-06	20.7531
M36808.m	rs7580094	A	G	0.7814	0.0083	0.0018	3.46E-06	21.2623
M36808.m	rs8107012	T	G	0.8077	-0.008	0.0018	9.12E-06	19.7531
M36850.m	rs11045834	T	C	0.2898	-0.0285	0.0063	7.2E-06	20.4649
M36850.m	rs1231385	T	C	0.513	-0.0288	0.0062	3.38E-06	21.5775
M36850.m	rs12365253	T	C	0.1707	-0.0359	0.008	7.78E-06	20.1377
M36850.m	rs12611993	T	C	0.245	-0.0627	0.0134	2.87E-06	21.894
M36850.m	rs16888843	T	C	0.0145	0.2982	0.065	4.43E-06	21.0469

M36850.m	rs2053585	A	G	0.6736	0.0556	0.0123	6.58E-06	20.4333
M36850.m	rs2609982	T	C	0.2865	-0.0294	0.0063	3.1E-06	21.7778
M36850.m	rs4148987	A	G	0.3217	-0.0317	0.0063	4.09E-07	25.3185
M36850.m	rs4149056	T	C	0.8396	-0.0412	0.0078	1.52E-07	27.9001
M36850.m	rs4579381	T	C	0.6475	-0.0278	0.0062	0.000007	20.1051
M36850.m	rs7500975	A	G	0.0376	-0.0833	0.0178	2.87E-06	21.9003
M36850.m	rs9904760	A	G	0.19	-0.0322	0.0072	8.12E-06	20.0008
M37004.m	rs10045874	T	G	0.0213	-0.0935	0.0204	4.78E-06	21.0069
M37004.m	rs10095556	A	G	0.8043	0.0264	0.0059	8.21E-06	20.0218
M37004.m	rs10748128	T	G	0.3647	0.0232	0.0052	8.65E-06	19.9053
M37004.m	rs17085679	A	T	0.1407	-0.0295	0.0066	7.95E-06	19.9782
M37004.m	rs17103130	A	G	0.0712	0.044	0.0097	5.98E-06	20.576
M37004.m	rs17583672	T	C	0.9631	0.0633	0.0141	7.57E-06	20.1544
M37004.m	rs195113	T	C	0.6742	0.0221	0.0049	8.04E-06	20.3419
M37004.m	rs9561537	A	G	0.5764	0.0221	0.0048	4.04E-06	21.1984
M37004.m	rs982583	T	G	0.0485	0.0473	0.0106	7.61E-06	19.9118
M37058.m	rs10082842	T	C	0.393	0.009	0.002	6.8E-06	20.25
M37058.m	rs1014079	A	C	0.3961	-0.0084	0.0019	6.39E-06	19.5457
M37058.m	rs1016145	T	C	0.5665	0.0092	0.0019	2.03E-06	23.446
M37058.m	rs1026192	A	C	0.1617	-0.0123	0.0027	6.54E-06	20.7531
M37058.m	rs1076230	A	G	0.532	0.009	0.002	6.8E-06	20.25
M37058.m	rs10809039	A	C	0.6121	-0.0089	0.0019	1.75E-06	21.9418
M37058.m	rs10835105	T	G	0.3498	-0.0084	0.0019	5.82E-06	19.5457
M37058.m	rs10848775	T	C	0.392	0.009	0.002	6.8E-06	20.25
M37058.m	rs10988217	A	G	0.3883	-0.0164	0.0019	1.36E-18	74.5042
M37058.m	rs1127203	T	C	0.0953	0.0178	0.0036	1.03E-06	24.4475
M37058.m	rs11637751	A	G	0.1659	0.0155	0.0027	1.77E-08	32.9561
M37058.m	rs1164883	A	G	0.5161	-0.0085	0.0019	6.38E-06	20.0139
M37058.m	rs1168556	A	G	0.6358	0.0089	0.0019	1.47E-06	21.9418
M37058.m	rs11713787	A	G	0.6291	-0.009	0.0019	1.7E-06	22.4377
M37058.m	rs12114477	A	G	0.4738	-0.009	0.0018	1.22E-06	25
M37058.m	rs12282416	T	C	0.475	0.009	0.0019	1.24E-06	22.4377
M37058.m	rs12513213	A	G	0.222	0.0119	0.0027	8.4E-06	19.4252
M37058.m	rs12899230	T	C	0.9213	-0.0247	0.0036	1.39E-11	47.0748
M37058.m	rs13397032	T	C	0.4316	-0.0085	0.0018	4.27E-06	22.2994
M37058.m	rs1472631	A	G	0.4919	0.0366	0.0018	1.85E-88	413.444
M37058.m	rs1727198	T	C	0.556	-0.009	0.002	6.8E-06	20.25
M37058.m	rs1764966	A	G	0.3934	0.0083	0.0019	7E-06	19.0831
M37058.m	rs17806888	T	C	0.8806	-0.0224	0.0034	7.15E-11	43.4048
M37058.m	rs17813064	A	G	0.0897	-0.0165	0.0037	7.05E-06	19.8868
M37058.m	rs195717	A	G	0.3882	0.0082	0.0019	9.03E-06	18.626
M37058.m	rs2206426	A	G	0.4177	-0.0084	0.0018	4.92E-06	21.7778
M37058.m	rs2374752	A	C	0.6354	-0.0084	0.0019	6.32E-06	19.5457
M37058.m	rs242725	A	G	0.358	-0.0086	0.0019	3.51E-06	20.4875
M37058.m	rs267939	T	C	0.6348	-0.0087	0.0019	2.59E-06	20.9668
M37058.m	rs2686513	T	C	0.552	-0.011	0.002	3.8E-08	30.25
M37058.m	rs2705088	T	G	0.7887	-0.0125	0.0027	4.17E-06	21.4335
M37058.m	rs2729786	A	C	0.099	0.0238	0.0036	3.97E-11	43.7068
M37058.m	rs327737	T	C	0.577	0.0098	0.0019	1.18E-07	26.6039
M37058.m	rs367213	A	G	0.5133	-0.0084	0.0018	4.74E-06	21.7778
M37058.m	rs4548847	A	G	0.5209	0.0082	0.0018	9.56E-06	20.7531
M37058.m	rs4837828	A	G	0.6656	-0.0089	0.0019	1.54E-06	21.9418
M37058.m	rs6425496	T	C	0.5429	0.0089	0.0019	1.63E-06	21.9418
M37058.m	rs6460991	A	G	0.473	0.0083	0.0019	8.53E-06	19.0831
M37058.m	rs6470127	A	G	0.6245	0.0087	0.0019	2.87E-06	20.9668

M37058.m	rs6703518	T	G	0.4273	-0.0108	0.0019	5.85E-09	32.3102
M37058.m	rs6774623	A	G	0.3843	-0.0084	0.0019	6.65E-06	19.5457
M37058.m	rs6958124	A	T	0.3835	-0.0085	0.0019	4.65E-06	20.0139
M37058.m	rs7039633	T	C	0.0286	0.0272	0.0061	9.59E-06	19.8828
M37058.m	rs7177	A	C	0.5419	-0.0088	0.0019	2.74E-06	21.4515
M37058.m	rs7999481	A	G	0.4612	0.0083	0.0019	8.18E-06	19.0831
M37058.m	rs8060756	A	G	0.3703	0.0102	0.0018	4.07E-08	32.1111
M37058.m	rs901273	A	G	0.5034	0.0107	0.0019	7.49E-09	31.7147
M37097.m	rs10079835	T	G	0.0301	-0.1332	0.0258	2.33E-07	26.6544
M37097.m	rs12320124	A	C	0.0146	-0.2943	0.0664	9.38E-06	19.6447
M37097.m	rs13184973	A	T	0.103	-0.0763	0.0138	3.29E-08	30.5697
M37097.m	rs2212465	A	T	0.0432	-0.0927	0.0207	7.3E-06	20.0548
M37097.m	rs2255908	A	G	0.6525	0.0466	0.0088	1.04E-07	28.0418
M37097.m	rs2269821	A	G	0.1204	-0.0573	0.013	9.55E-06	19.4278
M37097.m	rs2405522	A	G	0.1672	-0.1264	0.0113	4.49E-29	125.123
M37097.m	rs2548993	A	G	0.7341	0.058	0.0097	1.89E-09	35.753
M37097.m	rs3821142	T	C	0.5584	0.0747	0.0157	2.09E-06	22.6382
M37097.m	rs4792110	T	G	0.9378	0.114	0.0252	6.31E-06	20.4649
M37097.m	rs4898658	A	C	0.0682	-0.0777	0.0164	2.05E-06	22.4468
M37097.m	rs719672	A	C	0.8137	0.0917	0.0196	2.91E-06	21.889
M37097.m	rs7582926	T	C	0.7138	-0.0437	0.0095	3.89E-06	21.16
M37097.m	rs7733814	T	C	0.0581	-0.1034	0.0184	1.89E-08	31.5795
M37097.m	rs9651696	T	C	0.556	-0.0756	0.0156	1.2E-06	23.4852
M37104.m	rs10463762	T	C	0.1434	-0.0372	0.008	3.37E-06	21.6225
M37104.m	rs10813501	T	C	0.3812	-0.0281	0.0061	4.23E-06	21.2204
M37104.m	rs1246726	T	C	0.4786	0.0263	0.0054	1.09E-06	23.7205
M37104.m	rs13178969	T	C	0.8388	-0.0355	0.0079	6.47E-06	20.1931
M37104.m	rs1322179	T	C	0.1489	0.0411	0.008	3.09E-07	26.3939
M37104.m	rs17345545	A	C	0.7426	-0.0277	0.0062	9.08E-06	19.9607
M37104.m	rs17483594	T	C	0.9827	0.1024	0.022	3.16E-06	21.6648
M37104.m	rs2582379	A	G	0.2503	-0.0277	0.0063	9.49E-06	19.3321
M37104.m	rs4923384	T	C	0.7967	-0.0337	0.0074	4.9E-06	20.7394
M37104.m	rs626747	T	C	0.4816	0.0246	0.0055	6.95E-06	20.0053
M37104.m	rs631791	A	G	0.3017	0.0628	0.0132	2.06E-06	22.6345
M37104.m	rs7754899	A	G	0.0573	0.059	0.0127	3.33E-06	21.5822
M37104.m	rs7829309	A	G	0.424	0.0252	0.0055	4.07E-06	20.9931
M37104.m	rs8180176	T	C	0.4591	0.0264	0.0054	8.5E-07	23.9012
M37104.m	rs9833886	C	G	0.0137	0.2264	0.0497	5.21E-06	20.751
M37112.m	rs12587408	A	C	0.1352	-0.0888	0.0192	3.86E-06	21.3906
M37112.m	rs1294383	A	G	0.116	-0.0843	0.019	9.47E-06	19.6856
M37112.m	rs1560661	T	C	0.6472	0.1129	0.0236	1.77E-06	22.8857
M37112.m	rs1582016	T	C	0.2424	0.0681	0.0151	6.09E-06	20.3395
M37112.m	rs17180851	A	T	0.864	0.0837	0.0186	6.76E-06	20.25
M37112.m	rs2707795	T	G	0.972	-0.3579	0.0788	5.64E-06	20.6287
M37112.m	rs328637	A	G	0.1638	-0.0864	0.0173	6.13E-07	24.9422
M37112.m	rs3908007	A	G	0.2651	-0.1406	0.0318	9.57E-06	19.5486
M37112.m	rs4331456	T	C	0.2107	-0.1664	0.0366	5.52E-06	20.6702
M37112.m	rs4453613	T	C	0.3355	0.06	0.0133	6.83E-06	20.3516
M37112.m	rs4761693	T	C	0.6193	0.0643	0.0131	9.65E-07	24.0924
M37112.m	rs6050798	T	C	0.4924	-0.0655	0.0132	7.03E-07	24.6226
M37112.m	rs7797103	T	C	0.7905	-0.159	0.0334	1.94E-06	22.6622
M37190.m	rs10267212	T	C	0.7003	-0.0407	0.0068	1.66E-09	35.8237
M37190.m	rs10278040	A	G	0.0419	-0.1901	0.0168	1.17E-29	128.04
M37190.m	rs10836291	T	C	0.9	0.0461	0.0104	9.84E-06	19.6488
M37190.m	rs11180748	A	G	0.1661	0.0398	0.0082	1.33E-06	23.558

M37190.m	rs11186574	A	G	0.9729	-0.1609	0.0346	3.4E-06	21.6252
M37190.m	rs12879878	T	C	0.5575	-0.0299	0.0066	5.17E-06	20.5236
M37190.m	rs13222543	T	C	0.0201	-0.263	0.0282	1.18E-20	86.9788
M37190.m	rs16850831	A	G	0.1266	0.0429	0.0092	3.12E-06	21.744
M37190.m	rs2547231	A	C	0.8298	0.081	0.0084	3.35E-22	92.9847
M37190.m	rs3214031	A	C	0.7088	-0.0301	0.0067	6.05E-06	20.1829
M37190.m	rs4149056	T	C	0.8386	-0.0509	0.0086	4E-09	35.0299
M37190.m	rs4149452	T	C	0.2625	0.0522	0.0074	1.71E-12	49.7597
M37190.m	rs4372938	T	C	0.5433	0.0299	0.0066	6.09E-06	20.5236
M37190.m	rs4802397	T	C	0.8582	-0.0485	0.0097	5.84E-07	25
M37190.m	rs650849	T	G	0.6496	0.03	0.0066	5.64E-06	20.6612
M37190.m	rs6584331	T	C	0.4848	0.0334	0.0064	1.84E-07	27.2354
M37190.m	rs666518	T	C	0.0891	-0.0523	0.0114	4.24E-06	21.0472
M37190.m	rs7078211	A	G	0.8439	-0.0418	0.0084	6.41E-07	24.7625
M37190.m	rs7831994	T	C	0.0356	0.1316	0.0293	6.91E-06	20.1733
M37190.m	rs8187707	T	C	0.0533	-0.0631	0.0138	5.09E-06	20.9074
M37190.m	rs9602130	A	C	0.3642	0.0579	0.0116	5.72E-07	24.9139
M37202.m	rs10021313	T	C	0.9892	-0.2423	0.051	2.01E-06	22.5718
M37202.m	rs1046371	C	G	0.2251	0.0487	0.0103	2.18E-06	22.3555
M37202.m	rs10847490	A	G	0.7519	0.0326	0.0072	5.89E-06	20.5008
M37202.m	rs10852236	A	T	0.5885	0.0524	0.0109	1.41E-06	23.1105
M37202.m	rs11669552	A	G	0.5468	-0.0395	0.0065	1.47E-09	36.929
M37202.m	rs11761528	T	C	0.074	-0.0696	0.012	6.27E-09	33.64
M37202.m	rs13222543	T	C	0.0199	-0.1199	0.0262	4.63E-06	20.9429
M37202.m	rs13325508	T	C	0.9736	0.099	0.0221	7.28E-06	20.0672
M37202.m	rs17243501	T	C	0.1932	-0.0363	0.0079	4.54E-06	21.1134
M37202.m	rs1825496	A	G	0.5326	-0.0484	0.0108	8.22E-06	20.0837
M37202.m	rs2130589	A	G	0.3137	0.0345	0.007	9.18E-07	24.2908
M37202.m	rs2762353	A	G	0.4552	0.0318	0.0064	6.7E-07	24.6885
M37202.m	rs2847326	A	T	0.2374	0.0373	0.0073	3.37E-07	26.1079
M37202.m	rs296396	T	C	0.1651	-0.1754	0.0086	1.48E-92	415.97
M37202.m	rs4149056	T	C	0.8382	-0.0414	0.0085	1.17E-06	23.7226
M37202.m	rs4149452	T	C	0.2655	0.0773	0.0078	3.01E-23	98.2132
M37202.m	rs4334280	A	G	0.3817	-0.0291	0.0064	6.1E-06	20.6741
M37202.m	rs4398451	T	C	0.5546	-0.0345	0.0064	7.26E-08	29.0588
M37202.m	rs4802397	T	C	0.8586	-0.0621	0.0096	1.09E-10	41.8447
M37202.m	rs6588480	A	G	0.1493	-0.044	0.0092	1.82E-06	22.8733
M37202.m	rs6900835	T	C	0.2175	-0.0363	0.0079	4.16E-06	21.1134
M37202.m	rs7172197	A	G	0.5376	-0.0305	0.0065	3.16E-06	22.0178
M37202.m	rs7259671	T	C	0.3201	0.0435	0.0066	3.83E-11	43.4401
M37202.m	rs7968170	T	C	0.4284	-0.04	0.0086	3.16E-06	21.6333
M37202.m	rs933775	A	G	0.7146	0.0337	0.0074	4.81E-06	20.7394
M37202.m	rs9410301	T	C	0.5784	0.0472	0.0106	8.68E-06	19.8277
M37202.m	rs9983470	T	C	0.0285	-0.0847	0.0191	9.05E-06	19.6653
M37203.m	rs10082235	T	C	0.0735	-0.0385	0.0084	4.09E-06	21.0069
M37203.m	rs11045818	A	G	0.1636	-0.0285	0.0057	5.87E-07	25
M37203.m	rs12508354	T	G	0.328	-0.0232	0.0047	8.24E-07	24.3658
M37203.m	rs12521994	A	G	0.9559	0.05	0.0109	4.46E-06	21.042
M37203.m	rs12525109	A	G	0.771	0.0244	0.005	8.91E-07	23.8144
M37203.m	rs1337985	T	C	0.627	-0.0184	0.0041	7.64E-06	20.1404
M37203.m	rs13419511	A	G	0.5995	-0.0187	0.0041	5.78E-06	20.8025
M37203.m	rs1476117	A	G	0.5513	0.0187	0.0041	5.86E-06	20.8025
M37203.m	rs2295709	T	C	0.7937	0.0228	0.005	4.81E-06	20.7936
M37203.m	rs2762353	A	G	0.4536	0.0299	0.0041	2.51E-13	53.1832
M37203.m	rs296396	T	C	0.1648	-0.0298	0.0056	1.26E-07	28.3176

M37203.m	rs3733475	T	G	0.3611	-0.0221	0.0047	2.78E-06	22.11
M37203.m	rs4149056	T	C	0.8386	-0.0485	0.0055	1.33E-18	77.7603
M37203.m	rs6584331	T	C	0.4843	0.0211	0.004	1.77E-07	27.8256
M37203.m	rs6755484	T	C	0.4847	-0.0191	0.0041	3.11E-06	21.702
M37203.m	rs7183977	T	C	0.6755	-0.0233	0.0047	7.19E-07	24.5763
M37203.m	rs7321271	C	G	0.2543	0.0217	0.0049	8.96E-06	19.6122
M37203.m	rs9445285	T	C	0.1465	0.03	0.0068	9.33E-06	19.4637
M37203.m	rs966968	T	C	0.7093	0.0214	0.0048	7.86E-06	19.8767
M37203.m	rs9938618	A	G	0.943	-0.0452	0.0096	2.74E-06	22.1684
M37253.m	rs10196007	T	C	0.8157	-0.032	0.0065	7.63E-07	24.2367
M37253.m	rs10966201	T	C	0.6555	0.0151	0.0034	7.11E-06	19.724
M37253.m	rs11793331	T	C	0.0242	0.0619	0.0136	5.09E-06	20.7159
M37253.m	rs1568101	T	C	0.912	-0.0255	0.0057	8.62E-06	20.0139
M37253.m	rs16977497	A	G	0.0448	0.0538	0.0122	9.91E-06	19.4467
M37253.m	rs6064820	T	C	0.7229	0.0155	0.0034	4.01E-06	20.7829
M37253.m	rs6484669	T	C	0.7412	-0.0196	0.0044	7.75E-06	19.843
M37253.m	rs7702827	C	G	0.3005	-0.0152	0.0034	6.29E-06	19.9862
M37253.m	rs7725624	A	C	0.0865	-0.044	0.0094	3.02E-06	21.9104
M37253.m	rs8176023	A	G	0.9284	-0.0411	0.0087	2.46E-06	22.3175
M37253.m	rs890519	A	C	0.4158	0.0147	0.0033	9.06E-06	19.843
M37253.m	rs933341	A	G	0.2404	0.0166	0.0035	1.56E-06	22.4947
M37253.m	rs9544785	A	C	0.3614	-0.015	0.0033	6.63E-06	20.6612
M37459.m	rs12727717	####	C	0.0307	-0.1065	0.0226	2.37E-06	22.2066
M37459.m	rs4823691	####	C	0.9681	-0.1683	0.0378	8.67E-06	19.8237
M37459.m	rs6082720	####	C	0.0112	0.2245	0.05	7.03E-06	20.1601
M37459.x	rs11880474	T	C	0.9652	0.0685	0.0153	7.4E-06	20.0446
M37459.x	rs1281546	A	T	0.6689	-0.0271	0.0058	2.9E-06	21.8315
M37459.x	rs2188553	A	G	0.2009	0.035	0.0067	1.95E-07	27.2889
M37459.x	rs2285395	A	G	0.053	-0.0553	0.0123	7.56E-06	20.2134
M37459.x	rs4823691	T	C	0.9681	-0.1683	0.0378	8.67E-06	19.8237
M37459.x	rs6046183	A	T	0.1847	0.0302	0.0068	8.71E-06	19.724
M37459.x	rs6082720	T	C	0.0112	0.2245	0.05	7.03E-06	20.1601
M37459.x	rs6939019	A	G	0.4996	-0.0234	0.0051	5.26E-06	21.0519
M37459.x	rs7765368	A	G	0.899	-0.0408	0.009	5.17E-06	20.5511
M37459.x	rs9317679	T	C	0.8808	-0.0437	0.0097	6.05E-06	20.2964
M37506.m	rs10180510	T	C	0.4607	0.0089	0.0017	3.75E-07	27.4083
M37506.m	rs11222450	T	C	0.596	-0.0078	0.0018	9.18E-06	18.7778
M37506.m	rs11627310	A	G	0.251	0.0084	0.0019	7.06E-06	19.5457
M37506.m	rs11682640	A	G	0.5871	0.0082	0.0018	2.66E-06	20.7531
M37506.m	rs11692794	T	C	0.432	-0.008	0.0018	6.68E-06	19.7531
M37506.m	rs11782386	T	C	0.1092	-0.0137	0.0027	3.09E-07	25.7462
M37506.m	rs11938858	T	C	0.3657	-0.0084	0.0018	1.59E-06	21.7778
M37506.m	rs12478048	A	G	0.2527	0.0097	0.0019	1.49E-07	26.0637
M37506.m	rs12713852	A	G	0.6774	0.0081	0.0018	5.24E-06	20.25
M37506.m	rs13078757	T	G	0.71	-0.008	0.0018	7.86E-06	19.7531
M37506.m	rs13236645	A	G	0.7326	0.0085	0.0018	2.48E-06	22.2994
M37506.m	rs1432288	T	G	0.1421	0.0125	0.0026	1.18E-06	23.1139
M37506.m	rs1445447	A	G	0.7448	-0.0083	0.0018	3.91E-06	21.2623
M37506.m	rs1491540	A	C	0.6535	0.0078	0.0018	9.5E-06	18.7778
M37506.m	rs17310169	T	C	0.1509	-0.0116	0.0026	8.38E-06	19.9053
M37506.m	rs174541	T	C	0.6443	0.0079	0.0018	8.26E-06	19.2623
M37506.m	rs17539845	A	G	0.0938	-0.0152	0.0033	4.8E-06	21.2158
M37506.m	rs17734019	A	C	0.9604	0.0232	0.0049	2.03E-06	22.4173
M37506.m	rs1789679	A	G	0.689	0.0092	0.0018	2.92E-07	26.1235
M37506.m	rs2227543	T	C	0.4291	0.008	0.0018	7.09E-06	19.7531

M37506.m	rs2269282	T	C	0.6994	0.0081	0.0018	4.53E-06	20.25
M37506.m	rs2294070	A	T	0.7404	-0.0083	0.0018	3.87E-06	21.2623
M37506.m	rs2979471	A	T	0.2882	-0.009	0.0018	5.34E-07	25
M37506.m	rs3733593	A	G	0.4373	0.008	0.0017	4.66E-06	22.1453
M37506.m	rs39578	A	G	0.729	-0.01	0.002	5.73E-07	25
M37506.m	rs420422	T	C	0.542	-0.009	0.0018	3.01E-07	25
M37506.m	rs445925	A	G	0.079	-0.024	0.004	1.97E-09	36
M37506.m	rs4749219	A	G	0.2784	0.0082	0.0018	7.02E-06	20.7531
M37506.m	rs484061	A	G	0.5129	-0.0081	0.0017	3.62E-06	22.7024
M37506.m	rs534863	T	C	0.3246	-0.0086	0.0018	1.48E-06	22.8272
M37506.m	rs559346	T	C	0.4193	0.0079	0.0017	6.62E-06	21.5952
M37506.m	rs6043498	T	G	0.0688	-0.0172	0.0034	4.81E-07	25.5917
M37506.m	rs633695	A	G	0.7183	-0.01	0.0018	2.47E-08	30.8642
M37506.m	rs6511720	T	G	0.1203	-0.0134	0.0028	1.33E-06	22.9031
M37506.m	rs6589371	T	C	0.1212	0.0123	0.0026	3.24E-06	22.3802
M37506.m	rs6708367	A	G	0.7078	0.0092	0.0018	3.09E-07	26.1235
M37506.m	rs6745464	T	C	0.5494	-0.0084	0.0017	1.39E-06	24.4152
M37506.m	rs6911080	T	C	0.7145	-0.0089	0.0018	5.23E-07	24.4475
M37506.m	rs7185774	T	C	0.3408	0.0085	0.0018	1.26E-06	22.2994
M37506.m	rs7213136	A	G	0.6033	0.0079	0.0018	8.93E-06	19.2623
M37506.m	rs7632727	T	C	0.8935	0.0127	0.0027	2.01E-06	22.1248
M37506.m	rs8059670	T	C	0.4431	-0.0091	0.0017	1.88E-07	28.654
M37506.m	rs9266813	C	G	0.7349	-0.0088	0.0018	1.02E-06	23.9012
M37506.m	rs9572325	T	G	0.4286	0.0085	0.0018	1.88E-06	22.2994
M37506.m	rs9655220	A	G	0.6451	0.0079	0.0018	8.12E-06	19.2623
M37506.m	rs9853219	A	C	0.6488	0.0079	0.0018	6.95E-06	19.2623
M37506.m	rs9922615	T	C	0.9445	0.0187	0.0041	6.28E-06	20.8025
M37506.m	rs9947662	A	G	0.2582	0.0081	0.0018	7.46E-06	20.25
M38150.m	rs2672893	A	G	0.1652	-0.0218	0.0047	4.35E-06	21.5138
M38150.m	rs4656083	A	G	0.4386	0.0345	0.0068	4.09E-07	25.7407
M38150.m	rs6089828	T	C	0.6506	-0.0165	0.0036	5.86E-06	21.0069
M38150.m	rs749902	T	C	0.3362	0.0168	0.0037	4.61E-06	20.6165
M38178.m	rs10873878	T	G	0.8608	-0.0266	0.0056	1.65E-06	22.5625
M38178.m	rs10910157	T	C	0.4179	-0.0164	0.0036	6.24E-06	20.7531
M38178.m	rs11161521	T	C	0.6983	0.0608	0.0036	1.22E-63	285.235
M38178.m	rs11749983	T	C	0.3334	0.0162	0.0037	8.96E-06	19.1702
M38178.m	rs12143716	A	G	0.17	0.0283	0.0047	1.38E-09	36.2558
M38178.m	rs13390807	A	G	0.0557	0.0395	0.0078	4.12E-07	25.6451
M38178.m	rs17304141	T	G	0.9792	-0.1197	0.0143	5.93E-17	70.0674
M38178.m	rs17638535	A	G	0.0205	-0.068	0.0128	1.16E-07	28.2227
M38178.m	rs193538	T	G	0.7047	0.019	0.0037	2.27E-07	26.3696
M38178.m	rs5745542	C	G	0.8975	-0.0312	0.0061	3.1E-07	26.1607
M38178.m	rs6779957	A	G	0.6456	0.0163	0.0036	7.86E-06	20.5008
M38178.m	rs6971411	A	G	0.1381	0.0247	0.0053	2.92E-06	21.7191
M38178.m	rs721204	A	G	0.1119	0.0424	0.006	2.22E-12	49.9378
M38178.m	rs7534288	T	C	0.0272	-0.055	0.0116	2.01E-06	22.4807
M38178.m	rs8396	T	C	0.6961	0.0262	0.0037	8.96E-13	50.1417
M38768.m	rs10753083	T	G	0.4023	0.0107	0.0024	9.35E-06	19.8767
M38768.m	rs11118895	T	C	0.4678	0.021	0.0041	2.7E-07	26.2344
M38768.m	rs1190713	T	G	0.94	0.031	0.007	9.49E-06	19.6122
M38768.m	rs12429177	T	C	0.0186	-0.0657	0.0148	9.72E-06	19.7064
M38768.m	rs12967799	A	C	0.079	0.0215	0.0047	4.43E-06	20.9258
M38768.m	rs17155949	T	C	0.975	0.0641	0.0142	6.59E-06	20.377
M38768.m	rs1995777	T	C	0.556	-0.011	0.0024	4.66E-06	21.0069
M38768.m	rs3751196	A	G	0.0984	0.0221	0.0046	1.99E-06	23.0818

M38768.m	rs3851552	T	C	0.6232	0.0126	0.0026	1.47E-06	23.4852
M38768.m	rs4109037	A	T	0.1531	0.017	0.0037	3.75E-06	21.1103
M38768.m	rs4765789	T	C	0.4153	-0.0112	0.0024	3.88E-06	21.7778
M38768.m	rs6544157	A	G	0.9035	-0.0186	0.0042	7.83E-06	19.6122
M38768.m	rs7187181	T	C	0.0635	0.0263	0.0057	3.34E-06	21.2893
M38768.m	rs7681360	A	G	0.5819	0.0129	0.0024	1.08E-07	28.8906
M38768.m	rs9809122	A	G	0.531	-0.0113	0.0024	2.93E-06	22.1684

Supplementary Table 4: The instrumental variables associated with serum inflammatory factors.

EXPOSURE	SNP	EA	NEA	EAF	BETA	SE	P	F
IL10	rs10457128	G	A	0.361	0.0865	0.0172	5.24E-07	25.2915427
IL10	rs10493718	C	A	0.709	0.11	0.0222	7.16E-07	24.5515786
IL10	rs11206302	C	T	0.08	0.1189	0.0251	2.20E-06	22.4396597
IL10	rs2086656	C	T	0.305	0.0789	0.0171	3.78E-06	21.2893198
IL10	rs282258	C	T	0.575	-0.0992	0.0162	1.00E-09	37.4967231
IL10	rs3025021	C	T	0.632	-0.0947	0.0195	1.46E-06	23.5847206
IL10	rs35794877	C	A	0.508	-0.0794	0.0167	1.80E-06	22.6051848
IL10	rs41282660	G	A	0.132	0.1194	0.0255	3.72E-06	21.9244291
IL10	rs4349809	G	T	0.451	-0.2853	0.0165	5.77E-67	298.975537
IL10	rs465757	G	A	0.333	-0.084	0.0174	1.17E-06	23.3055886
IL10	rs4741748	G	A	0.428	0.0793	0.017	2.79E-06	21.759481
IL10	rs7088799	G	T	0.434	0.0852	0.0167	3.23E-07	26.0283266
IL12p70	rs10738760	G	A	0.497	-0.0896	0.0156	1.02E-08	32.9888231
IL12p70	rs13209117	G	A	0.716	-0.1002	0.0186	5.57E-08	29.0208117
IL12p70	rs17229494	G	A	0.087	0.1172	0.0257	4.93E-06	20.796439
IL12p70	rs200757	C	T	0.877	0.0985	0.0214	4.15E-06	21.1858023
IL12p70	rs282258	C	T	0.575	-0.073	0.0156	3.21E-06	21.8976003
IL12p70	rs41282644	G	A	0.936	-0.1473	0.0304	1.05E-06	23.4778502
IL12p70	rs4349809	G	T	0.451	-0.3777	0.0159	2.56E-124	564.286579
IL12p70	rs4734879	G	A	0.292	-0.0918	0.0189	1.22E-06	23.5918367
IL12p70	rs7088799	G	T	0.434	0.0998	0.0161	6.29E-10	38.4245978
IL12p70	rs71361173	G	T	0.113	-0.111	0.0239	3.06E-06	21.5700005
IL12p70	rs72831623	G	A	0.947	-0.1913	0.037	2.42E-07	26.7316947
IL12p70	rs782107	G	A	0.445	-0.075	0.0156	1.60E-06	23.1139053
IL12p70	rs79121401	C	T	0.019	-0.5548	0.1206	4.24E-06	21.1630515
IL13	rs117795020	G	A	0.982	0.3522	0.0716	9.86E-07	24.196506
IL13	rs12623722	G	A	0.718	0.1185	0.0258	4.19E-06	21.0958626
IL13	rs139083458	C	T	0.984	-0.9902	0.2107	2.81E-06	22.085985
IL13	rs142167313	C	T	0.019	0.313	0.0617	3.98E-07	25.7346548
IL13	rs27949	C	T	0.369	0.1168	0.0252	3.43E-06	21.4824893
IL13	rs6799107	C	T	0.214	0.1459	0.0301	1.25E-06	23.4951159
IL13	rs7073807	C	T	0.883	-0.1682	0.0356	2.37E-06	22.3229706
IL13	rs75995699	G	A	0.976	-0.3319	0.0698	2.64E-06	22.6101612
IL13	rs9472168	G	A	0.449	-0.4244	0.0248	1.08E-65	292.851457
IL16	rs116135478	G	A	0.018	-0.8206	0.166	3.66E-06	24.4369415
IL16	rs117217798	C	T	0.931	0.2036	0.0444	4.15E-06	21.0275952
IL16	rs117916513	G	A	0.979	0.502	0.0986	3.79E-07	25.9211106
IL16	rs1255143	C	T	0.434	-0.1306	0.0242	7.10E-08	29.1243084
IL16	rs12765671	G	A	0.983	0.6023	0.1318	4.84E-06	20.883097
IL16	rs144691581	G	A	0.985	-0.4882	0.0967	4.20E-07	25.4884016
IL16	rs1801020	G	A	0.775	-0.1733	0.0272	4.53E-10	40.5937635
IL16	rs4253283	C	T	0.7	-0.146	0.0262	1.75E-08	31.0529689
IL16	rs4513633	C	A	0.124	0.2239	0.0453	7.44E-07	24.4293428
IL16	rs4778636	G	A	0.925	0.7272	0.0633	1.11E-30	131.977629
IL16	rs9706053	C	T	0.984	-0.4582	0.0932	7.01E-07	24.1700943
IL17	rs117029961	G	A	0.989	-0.4585	0.1015	4.94E-06	20.4054697
IL17	rs117556572	C	T	0.989	0.5102	0.1099	3.28E-06	21.551898
IL17	rs1530455	C	T	0.592	-0.108	0.0173	4.87E-10	38.9722343
IL17	rs17106604	C	T	0.876	-0.1129	0.0225	6.37E-07	25.1780938

IL17	rs17282552	C	T	0.027	0.2001	0.0405	8.21E-07	24.4109191
IL17	rs184080173	C	T	0.063	-0.2384	0.0471	4.19E-07	25.6195023
IL17	rs187475560	C	T	0.988	0.2434	0.052	3.29E-06	21.9096006
IL17	rs34120897	C	A	0.097	0.1055	0.0232	3.63E-06	20.6789722
IL17	rs62191444	G	T	0.834	0.1136	0.0247	4.22E-06	21.1525513
IL17	rs78296352	G	T	0.971	-0.3027	0.0646	4.27E-06	21.9563329
IL17	rs78612928	C	T	0.197	-0.1037	0.0222	2.62E-06	21.8198401
IL17	rs79462337	G	T	0.02	-0.2097	0.0435	1.41E-06	23.2390488
IL18	rs10414578	C	T	0.879	0.1771	0.035	4.16E-07	25.6036
IL18	rs115267715	C	T	0.982	-0.4508	0.08	1.72E-08	31.753225
IL18	rs116383510	C	A	0.02	0.5426	0.1056	3.00E-07	26.4016909
IL18	rs11700536	C	T	0.638	-0.1156	0.025	4.21E-06	21.381376
IL18	rs117266781	C	T	0.989	-0.6841	0.1468	3.15E-06	21.7163619
IL18	rs144841621	C	T	0.989	-0.518	0.1141	3.81E-06	20.6104859
IL18	rs17229943	C	A	0.05	0.312	0.0463	1.62E-11	45.4095508
IL18	rs1852105	C	T	0.951	-0.3036	0.0661	4.32E-06	21.0960242
IL18	rs1979967	C	T	0.741	-0.1402	0.0286	9.45E-07	24.0305638
IL18	rs2729385	G	A	0.675	-0.1231	0.0262	3.79E-06	22.0756512
IL18	rs385076	C	T	0.647	0.2432	0.0248	1.66E-22	96.1664932
IL18	rs4482818	G	A	0.359	-0.1286	0.0244	1.45E-07	27.7780838
IL18	rs658805	G	A	0.687	-0.1226	0.0244	4.94E-07	25.2465063
IL18	rs71478720	C	T	0.782	0.2669	0.0276	3.71E-22	93.5145059
IL18	rs78623212	C	T	0.976	-0.8705	0.1778	6.71E-07	23.9703314
IL18	rs78716465	G	A	0.962	-0.3265	0.0682	1.63E-06	22.9191033
IL1b	rs115242021	C	A	0.919	-0.2326	0.0414	5.07E-07	31.5659409
IL1b	rs143319329	C	T	0.976	-0.2801	0.0715	2.00E-06	15.3466693
IL1b	rs1942793	G	T	0.526	-0.0717	0.0187	4.98E-06	14.7012783
IL1b	rs61335305	C	A	0.989	-0.2966	0.0724	1.90E-06	16.7828287
IL1b	rs62015704	G	A	0.121	-0.1082	0.0283	2.09E-06	14.6177877
IL1b	rs9898641	C	T	0.36	0.2032	0.0454	3.59E-06	20.0325254
IL1ra	rs1054402	C	T	0.734	-0.1311	0.027	1.13E-06	23.5764198
IL1ra	rs11627423	C	A	0.39	-0.1171	0.0247	2.12E-06	22.4760445
IL1ra	rs12121840	C	T	0.916	-0.2692	0.0571	2.43E-06	22.2268488
IL1ra	rs139005642	AAAAG	A	0.147	-0.1315	0.0277	1.97E-06	22.5367853
IL1ra	rs2809154	C	T	0.838	0.1786	0.0388	3.74E-06	21.1884632
IL1ra	rs56134659	G	A	0.474	0.1117	0.0237	2.44E-06	22.2131247
IL1ra	rs61335305	C	A	0.989	-0.4453	0.0908	1.00E-06	24.0510307
IL1ra	rs9623661	C	T	0.908	0.1966	0.0426	3.86E-06	21.2984417
IL2	rs10903540	G	A	0.034	0.1581	0.0343	4.19E-06	21.2459179
IL2	rs12051139	C	T	0.401	0.1131	0.0247	4.76E-06	20.966759
IL2	rs13412535	G	A	0.774	-0.1764	0.0332	1.18E-07	28.2306576
IL2	rs1534019	C	T	0.556	0.1187	0.0248	1.58E-06	22.9085751
IL2	rs170117	C	T	0.88	0.1617	0.0349	3.87E-06	21.4668927
IL2	rs2807544	G	A	0.566	-0.1175	0.0253	3.41E-06	21.5692325
IL2	rs4634519	G	A	0.312	0.1261	0.0269	2.77E-06	21.9748345
IL2	rs61335305	C	A	0.989	-0.4514	0.0918	7.32E-07	24.1789673
IL2	rs62124990	G	T	0.984	0.6961	0.1495	3.22E-06	21.6800801
IL2	rs7615304	G	A	0.651	0.1172	0.0242	1.21E-06	23.4544089
IL2	rs80336398	C	T	0.021	-0.4001	0.0858	2.82E-06	21.745156
IL2ra	rs11241559	G	T	0.781	0.1264	0.0266	2.00E-06	22.5803607
IL2ra	rs115360066	G	A	0.11	-0.1867	0.0379	8.06E-07	24.2666718

IL2ra	rs117244812	G	A	0.986	0.7064	0.1488	2.10E-06	22.5369696
IL2ra	rs12722497	C	A	0.856	-0.6279	0.0485	1.57E-38	167.609059
IL2ra	rs185231391	C	T	0.015	-0.8503	0.1809	1.47E-06	22.0936375
IL2ra	rs4733117	C	A	0.164	-0.1369	0.0292	2.63E-06	21.9806835
IL2ra	rs61705228	C	T	0.963	-0.3303	0.0716	3.99E-06	21.2809545
IL2ra	rs62135626	C	T	0.885	0.1661	0.035	1.99E-06	22.5218041
IL4	rs10512267	C	T	0.33	0.0824	0.0161	2.94E-07	26.1940512
IL4	rs116705532	G	T	0.017	0.4678	0.0978	1.76E-06	22.8792996
IL4	rs117146485	C	T	0.015	0.2924	0.0629	2.71E-06	21.6099343
IL4	rs17713451	G	A	0.835	-0.1274	0.0253	4.97E-07	25.3569967
IL4	rs2332982	G	A	0.142	-0.1118	0.0219	3.20E-07	26.0612581
IL4	rs6765768	G	A	0.392	-0.0795	0.0167	2.00E-06	22.6621607
IL4	rs73023729	G	A	0.977	0.1796	0.0366	9.03E-07	24.079668
IL4	rs7613691	G	A	0.053	-0.1775	0.0384	4.05E-06	21.3665432
IL4	rs79597994	C	T	0.972	0.5831	0.127	4.32E-06	21.08039
IL4	rs9508291	C	T	0.065	0.1676	0.0359	3.03E-06	21.7951133
IL4	rs9941733	G	A	0.175	-0.114	0.0229	6.88E-07	24.7821361
IL5	rs11680908	G	A	0.055	-0.2634	0.0554	2.03E-06	22.6053904
IL5	rs6737109	C	T	0.425	-0.116	0.0247	2.40E-06	22.0557623
IL5	rs72831687	G	A	0.983	0.5239	0.1109	1.69E-06	22.3168916
IL5	rs73040130	C	T	0.065	-0.2638	0.0529	6.00E-07	24.86785
IL5	rs7767396	G	A	0.451	-0.1515	0.0246	7.69E-10	37.9275729
IL6	rs114373846	C	T	0.987	-0.422	0.0904	3.32E-06	21.7915459
IL6	rs12024374	G	A	0.884	-0.1096	0.0236	3.35E-06	21.5673657
IL6	rs1333040	C	T	0.426	-0.0738	0.0158	3.17E-06	21.8171767
IL6	rs13412535	G	A	0.774	0.1164	0.0215	7.34E-08	29.3108924
IL6	rs72831623	G	A	0.947	-0.1973	0.0372	1.08E-07	28.1299066
IL6	rs73273528	C	T	0.966	-0.2672	0.0553	9.58E-07	23.3465464
IL6	rs76856708	C	T	0.038	-0.3289	0.07	2.61E-06	22.0765735
IL7	rs117509142	C	T	0.057	0.327	0.0688	1.99E-06	22.5901247
IL7	rs141425475	C	T	0.029	0.4781	0.1016	2.53E-06	22.143694
IL7	rs144701438	G	A	0.946	0.4819	0.0989	9.75E-07	23.742217
IL7	rs17091524	C	T	0.043	-0.4924	0.1013	1.91E-06	23.6274688
IL7	rs218260	C	T	0.788	0.1337	0.0286	2.93E-06	21.8539904
IL7	rs28793375	C	T	0.833	-0.1638	0.0361	4.46E-06	20.5879636
IL7	rs4320361	G	T	0.548	0.3245	0.0249	6.87E-39	169.836374
IL7	rs62006410	C	T	0.785	0.1557	0.0303	3.39E-07	26.4053524
IL7	rs75904417	C	A	0.141	0.1698	0.0349	1.16E-06	23.6714313
IL7	rs77318030	C	T	0.052	0.2921	0.0632	3.74E-06	21.3613629
IL7	rs77981494	C	T	0.016	0.5178	0.1064	1.07E-06	23.6832247
IL7	rs78346957	G	A	0.977	-0.4588	0.1007	4.51E-06	20.7581133
IL8	rs11634944	C	T	0.347	0.1214	0.0252	1.29E-06	23.2079239
IL8	rs12075	G	A	0.398	-0.12	0.0236	3.88E-07	25.8546395
IL8	rs141926526	C	A	0.048	0.6149	0.1308	2.57E-06	22.1000623
IL8	rs2673604	C	A	0.299	0.1266	0.0255	7.02E-07	24.6483045
IL9	rs117935362	G	A	0.985	0.5107	0.1114	4.85E-06	21.0165456
IL9	rs41294750	C	T	0.972	-0.3514	0.0748	2.37E-06	22.0698976
IL9	rs4880409	C	T	0.028	0.3355	0.0723	3.50E-06	21.5331941
IL9	rs61867538	C	T	0.973	-0.3566	0.0774	3.93E-06	21.2266157
IL9	rs7232268	G	A	0.952	-0.2759	0.0587	2.53E-06	22.0916014
IL9	rs7242404	G	A	0.702	0.1228	0.0264	3.27E-06	21.6365932

IL9	rs76963786	C	T	0.914	0.2865	0.0557	4.50E-07	26.4568943
IL10	rs10809307	C	T	0.696	-0.1305	0.0282	3.64E-06	21.4152331
IL10	rs113831257	G	A	0.958	-0.3592	0.0644	2.53E-08	31.1100652
IL10	rs11626201	C	A	0.366	-0.1162	0.0245	1.93E-06	22.4946939
IL10	rs143799975	G	A	0.013	0.7984	0.1637	1.00E-06	23.7872205
IL10	rs181236764	C	T	0.957	-0.4504	0.0964	3.22E-06	21.8294451
IL10	rs34383175	C	T	0.973	0.3153	0.0657	1.51E-06	23.0312337
IL10	rs397816	C	T	0.426	-0.1237	0.0249	7.90E-07	24.6797471
IL10	rs75970138	G	A	0.987	0.485	0.104	1.53E-06	21.7478735
IL10	rs7645625	G	T	0.432	0.1086	0.0237	4.41E-06	20.9972761
IL10	rs79848609	C	A	0.032	-0.2603	0.0537	8.75E-07	23.4963155
IL10	rs8112909	G	A	0.192	0.1426	0.0299	1.94E-06	22.7455621
IL10	rs9450351	C	T	0.062	0.2768	0.0489	1.48E-08	32.0416191
MIP1a	rs10835056	G	T	0.756	-0.1194	0.0254	2.60E-06	22.0974022
MIP1a	rs116615337	G	A	0.291	-0.1278	0.0279	4.82E-06	20.9823101
MIP1a	rs12690897	G	A	0.719	-0.1248	0.0262	2.11E-06	22.6895869
MIP1a	rs184154340	G	A	0.959	-0.331	0.0693	1.86E-06	22.8133739
MIP1a	rs34771762	G	A	0.074	-0.249	0.0523	2.13E-06	22.6670664
MIP1a	rs57786342	G	A	0.784	-0.1314	0.0285	4.06E-06	21.2569529
MIP1a	rs60198979	G	A	0.914	0.2146	0.0458	2.62E-06	21.9547491
MIP1a	rs6900267	C	A	0.075	0.2429	0.0519	2.89E-06	21.9038428
MIP1a	rs7232268	G	A	0.952	-0.2821	0.0599	2.55E-06	22.1795396
MIP1b	rs113010081	C	T	0.108	0.5954	0.0236	3.85E-140	636.493034
MIP1b	rs113877493	C	T	0.878	0.6124	0.0218	1.62E-173	789.146031
MIP1b	rs116237296	G	A	0.984	-0.5437	0.1115	7.23E-07	23.7776501
MIP1b	rs117453826	G	A	0.016	0.5774	0.0593	5.07E-22	94.8078226
MIP1b	rs12490293	C	T	0.392	-0.0866	0.0168	2.12E-07	26.5715703
MIP1b	rs141102180	G	T	0.982	-0.3225	0.0393	1.08E-16	67.34019
MIP1b	rs1437220	C	T	0.05	-0.1478	0.0315	3.53E-06	22.0154598
MIP1b	rs1564708	C	T	0.73	0.1744	0.0188	2.87E-20	86.0552286
MIP1b	rs17138331	G	A	0.094	0.1391	0.0295	2.26E-06	22.2336225
MIP1b	rs2411161	C	T	0.051	-0.1714	0.0367	3.14E-06	21.8116995
MIP1b	rs281749	C	T	0.708	-0.0799	0.0171	3.17E-06	21.8323929
MIP1b	rs3760440	G	A	0.331	-0.1236	0.0162	2.75E-14	58.2112483
MIP1b	rs62242409	C	T	0.743	-0.1282	0.0193	3.43E-11	44.1226342
MIP1b	rs72791296	C	T	0.941	-0.2369	0.0466	3.78E-07	25.8439141
MIP1b	rs72799710	C	T	0.834	0.1014	0.0218	3.21E-06	21.6353001
MIP1b	rs74810984	C	T	0.022	-0.2206	0.0474	1.96E-06	21.6597945
MIP1b	rs76582507	G	A	0.973	-0.3175	0.0677	3.26E-06	21.9942989
MIP1b	rs76583883	G	T	0.968	0.2317	0.0511	4.99E-06	20.559392
MIP1b	rs76776296	G	A	0.037	-0.2997	0.0598	5.55E-07	25.1171939
MIP1b	rs9793308	G	A	0.372	-0.0835	0.0178	2.52E-06	22.0055864
MIP1b	rs9850846	G	A	0.444	-0.072	0.0157	4.48E-06	21.0312792
Eotaxin	rs11087905	C	A	0.658	-0.0941	0.0189	5.48E-07	24.7888077
Eotaxin	rs112347425	C	T	0.893	-0.158	0.0277	8.65E-09	32.5352865
Eotaxin	rs12075	G	A	0.398	-0.1671	0.0156	1.33E-26	114.737056
Eotaxin	rs138125931	C	T	0.868	-0.1292	0.0254	3.59E-07	25.8736437
Eotaxin	rs1476670	C	A	0.793	0.1007	0.0217	3.51E-06	21.5347321
Eotaxin	rs2024050	G	A	0.9	-0.1728	0.0303	1.10E-08	32.5238702
Eotaxin	rs2210755	C	T	0.081	0.1104	0.0242	4.85E-06	20.8116932
Eotaxin	rs2211994	C	T	0.75	-0.0885	0.0177	6.08E-07	25

Eotaxin	rs2228467	C	T	0.07	0.4163	0.0292	2.27E-46	203.257752
Eotaxin	rs2419841	C	T	0.119	0.1277	0.0279	4.98E-06	20.9494868
Eotaxin	rs5746492	G	A	0.181	-0.0954	0.0207	3.96E-06	21.2400756
Eotaxin	rs5754733	C	A	0.212	0.1042	0.0214	1.06E-06	23.7087082
Eotaxin	rs59808887	C	T	0.923	0.1673	0.0358	2.91E-06	21.838652
Eotaxin	rs75426604	C	A	0.869	0.1366	0.0291	2.53E-06	22.03512
Eotaxin	rs79722574	C	T	0.84	0.1113	0.0228	1.06E-06	23.829813
Eotaxin	rs80341932	G	A	0.292	-0.1016	0.0205	6.69E-07	24.562903
Eotaxin	rs9317045	C	A	0.146	-0.1182	0.0237	5.82E-07	24.873578
MCP1	rs10145849	G	A	0.651	0.0755	0.0162	3.41E-06	21.7202027
MCP1	rs10744620	C	T	0.639	-0.0788	0.0161	9.91E-07	23.9552486
MCP1	rs111995966	G	T	0.026	-0.1452	0.031	2.53E-06	21.9386472
MCP1	rs12073356	G	A	0.915	0.1426	0.0311	4.17E-06	21.0241416
MCP1	rs12075	G	A	0.398	-0.2185	0.0155	1.44E-44	198.719043
MCP1	rs146522229	C	T	0.976	0.5976	0.1177	3.56E-07	25.7791297
MCP1	rs2036297	G	A	0.657	-0.119	0.016	1.09E-13	55.3164063
MCP1	rs2288370	C	T	0.396	0.1031	0.0163	2.25E-10	40.0075652
MCP1	rs2712431	C	A	0.285	0.0787	0.0172	4.76E-06	20.9359451
MCP1	rs56212190	C	T	0.945	-0.181	0.0373	9.85E-07	23.5472116
MCP1	rs7197349	G	A	0.137	-0.0968	0.0206	2.62E-06	22.0808747
MCP1	rs7517040	G	A	0.718	0.0987	0.0191	2.44E-07	26.7034621
MCP1	rs7632755	G	A	0.92	-0.2938	0.0316	1.18E-20	86.4429178
MCP1	rs9317045	C	A	0.146	-0.1134	0.0236	1.52E-06	23.0888394
MCP3	rs10892381	C	T	0.3	-0.2412	0.0476	3.56E-07	25.6767884
MCP3	rs62492260	G	T	0.851	0.2788	0.058	1.54E-06	23.1062545
MCP3	rs73669117	G	A	0.015	0.6238	0.131	2.56E-06	22.6750446
MIG	rs111607343	G	A	0.962	0.521	0.1119	2.83E-06	21.6778034
MIG	rs11177248	G	A	0.939	-0.3073	0.067	4.45E-06	21.0365984
MIG	rs112337562	G	T	0.017	0.37	0.0796	2.98E-06	21.6061463
MIG	rs112861654	G	A	0.087	0.2765	0.0529	1.81E-07	27.3198888
MIG	rs117831247	C	T	0.981	0.8334	0.1754	2.16E-06	22.5760425
MIG	rs139010077	C	T	0.989	-0.4322	0.095	3.55E-06	20.6977108
MIG	rs1796086	C	T	0.094	0.2096	0.0403	2.23E-07	27.0503236
MIG	rs41272086	G	A	0.915	0.2226	0.0415	7.43E-08	28.770945
MIG	rs55876513	G	T	0.248	-0.166	0.0255	8.23E-11	42.3775471
MIG	rs5752128	C	T	0.095	0.1685	0.0369	4.34E-06	20.8519694
MIG	rs62562991	G	A	0.98	-0.6236	0.126	8.40E-07	24.4946435
MIG	rs6679677	C	A	0.909	-0.162	0.0329	8.86E-07	24.2458957
MIG	rs77086208	C	T	0.981	-0.3226	0.0698	3.83E-06	21.3608181
MIG	rs816960	C	T	0.741	0.1224	0.0244	5.01E-07	25.1642032
CTACK	rs116303454	G	A	0.971	-0.383	0.0816	3.27E-06	22.0301386
CTACK	rs135564	G	A	0.741	0.1893	0.0268	2.43E-12	49.8920834
CTACK	rs145902143	G	A	0.038	0.2838	0.0581	1.03E-06	23.8601142
CTACK	rs17321950	C	T	0.028	-0.5262	0.1122	3.46E-06	21.9945952
CTACK	rs2070074	G	A	0.102	-0.4467	0.0374	1.79E-32	142.655559
CTACK	rs3766110	C	A	0.233	0.1287	0.0278	3.86E-06	21.4322369
CTACK	rs55764737	C	T	0.023	-0.5313	0.0972	4.62E-08	29.8776959
CTACK	rs57338032	G	A	0.18	-0.1583	0.0317	6.23E-07	24.9369483
CTACK	rs7333764	C	T	0.973	-0.2773	0.0593	2.85E-06	21.8670578
CTACK	rs76395525	G	A	0.985	-0.5277	0.1083	9.55E-07	23.7419986
RANTES	rs112072646	G	A	0.969	-0.4286	0.0862	6.48E-07	24.7223529

RANTES	rs147509526	C	T	0.987	0.358	0.0717	6.93E-07	24.9303136
RANTES	rs2251660	C	A	0.145	-0.1829	0.0359	3.83E-07	25.9560447
RANTES	rs4940620	G	A	0.064	0.2494	0.054	3.54E-06	21.3307133
RANTES	rs62438851	G	A	0.135	0.1957	0.0414	2.33E-06	22.3450314
RANTES	rs7000423	C	T	0.674	0.1318	0.0253	1.82E-07	27.1387461
RANTES	rs72793342	G	A	0.792	0.1487	0.0308	1.48E-06	23.3088316
RANTES	rs74472919	C	T	0.981	-0.3313	0.0605	3.97E-08	29.9869381
RANTES	rs75613039	C	T	0.969	-0.37	0.081	4.81E-06	20.8657217
RANTES	rs818452	C	T	0.948	-0.2381	0.0505	2.36E-06	22.2298245
GROa	rs1113500	G	T	0.37	-0.1174	0.0244	1.57E-06	23.1502956
GROa	rs118158560	G	A	0.937	-0.2703	0.0594	3.42E-06	20.7070962
GROa	rs12075	G	A	0.398	-0.3751	0.0237	1.24E-55	250.494063
GROa	rs140734053	G	A	0.975	-0.7257	0.1561	3.58E-06	21.6126709
GROa	rs185768063	G	A	0.014	-0.3998	0.076	1.46E-07	27.6731371
GROa	rs188345231	C	T	0.988	-0.623	0.1323	4.34E-06	22.1746312
GROa	rs508977	G	T	0.234	0.3802	0.028	7.56E-42	184.377602
GROa	rs62024303	G	A	0.046	0.3053	0.0666	4.41E-06	21.0138269
GROa	rs78653452	G	T	0.988	0.7362	0.1558	1.21E-06	22.3283545
SDF1a	rs10474392	G	A	0.731	-0.0962	0.0178	1.24E-06	29.2085595
SDF1a	rs12407262	G	A	0.866	-0.1179	0.0266	3.99E-06	19.6455566
SDF1a	rs13400104	G	A	0.18	0.0647	0.0189	4.53E-06	11.7188489
SDF1a	rs139840550	G	A	0.952	-0.1834	0.0549	3.79E-06	11.1597374
SDF1a	rs149893336	G	A	0.02	0.5034	0.1081	4.52E-06	21.6857793
SDF1a	rs16872470	G	A	0.013	-0.5319	0.1025	2.81E-07	26.9285054
SDF1a	rs4581824	G	T	0.311	0.0701	0.0173	3.05E-06	16.4188914
SDF1a	rs482700	G	A	0.286	0.0893	0.0203	1.57E-06	19.351331
SDF1a	rs67689854	C	A	0.885	0.0681	0.0195	3.07E-06	12.196213
TNFa	rs10834997	G	A	0.36	0.1247	0.0258	1.33E-06	23.3611111
TNFa	rs111332265	G	A	0.072	0.3766	0.0754	6.63E-07	24.9469777
TNFa	rs8121916	C	A	0.774	-0.1306	0.0278	2.72E-06	22.0697169
TNFb	rs10925040	C	T	0.611	-0.1755	0.0373	2.67E-06	22.137908
TNFb	rs753274	C	T	0.464	0.1736	0.0371	2.77E-06	21.8953364
TNFb	rs7629875	G	A	0.058	-0.3766	0.0774	1.37E-06	23.6743852
TNFb	rs78296352	G	T	0.971	-1.2215	0.1366	4.76E-21	79.9623491
TRAIL	rs11618126	G	A	0.015	-0.8908	0.1914	1.46E-06	21.6609026
TRAIL	rs11657269	G	A	0.119	-0.1188	0.026	4.78E-06	20.8778698
TRAIL	rs11699445	G	T	0.387	-0.0746	0.0161	3.27E-06	21.4696964
TRAIL	rs13115587	C	A	0.917	-0.1019	0.0218	3.10E-06	21.849192
TRAIL	rs13185784	G	A	0.71	-0.0846	0.0183	3.90E-06	21.3716743
TRAIL	rs13278062	G	T	0.497	-0.0801	0.0157	3.57E-07	26.0294941
TRAIL	rs141603697	C	T	0.987	-0.7202	0.0741	6.61E-22	94.4647584
TRAIL	rs146783010	G	A	0.011	0.6016	0.135	4.83E-06	19.8585767
TRAIL	rs148051545	C	T	0.987	0.3921	0.0848	3.86E-06	21.3797184
TRAIL	rs193112415	C	T	0.014	1.0421	0.0623	2.15E-62	279.796771
TRAIL	rs57396456	C	T	0.034	0.5626	0.0518	1.25E-27	117.961405
TRAIL	rs62093514	C	T	0.974	-1.0618	0.0552	6.86E-82	370.004739
TRAIL	rs6878823	C	T	0.772	0.0879	0.0189	3.48E-06	21.6298816
TRAIL	rs73039026	C	A	0.016	0.2999	0.0635	2.02E-06	22.3051671
TRAIL	rs74488044	G	A	0.94	-0.3489	0.0335	2.61E-25	108.470671
TRAIL	rs747324	C	T	0.641	0.0855	0.0178	1.61E-06	23.0723709
TRAIL	rs74778900	C	T	0.986	-0.5906	0.0532	2.59E-28	123.243386

TRAIL	rs75928541	G	A	0.983	-0.275	0.0593	4.24E-06	21.5058197
TRAIL	rs79287178	G	A	0.974	0.4317	0.0421	9.12E-25	105.147731
MIF	rs113218956	G	A	0.989	0.8948	0.1879	2.26E-06	22.6776679
MIF	rs118055855	C	T	0.013	-0.6907	0.15	4.13E-06	21.2029551
MIF	rs12594190	G	A	0.247	-0.1355	0.0267	3.70E-07	25.7546746
MIF	rs13142904	C	T	0.931	0.223	0.0425	2.56E-07	27.5316263
MIF	rs141009259	C	T	0.013	0.6178	0.1322	2.47E-06	21.8390075
MIF	rs5751777	C	T	0.389	0.1342	0.0249	7.09E-08	29.0473379
MIF	rs78098071	C	T	0.019	0.4867	0.0918	1.78E-07	28.108478
IFNg	rs112783231	G	A	0.971	0.2408	0.0511	1.96E-06	22.2060424
IFNg	rs113600793	C	A	0.96	-0.1829	0.0373	8.95E-07	24.0441676
IFNg	rs115729819	G	A	0.021	-0.2484	0.0515	1.38E-06	23.2642323
IFNg	rs11843756	G	T	0.029	-0.184	0.0393	3.09E-06	21.9205045
IFNg	rs12420286	C	T	0.029	-0.2376	0.0501	2.08E-06	22.4914482
IFNg	rs1867282	C	T	0.674	-0.0774	0.0166	3.15E-06	21.7403106
IFNg	rs2073438	G	A	0.729	-0.0898	0.0188	1.68E-06	22.8158669
IFNg	rs4730203	G	A	0.862	0.0983	0.0203	1.32E-06	23.4484943
IFNg	rs6902031	G	A	0.888	0.1166	0.0242	1.41E-06	23.214876
IFNg	rs7073753	C	T	0.567	-0.0821	0.0167	8.39E-07	24.1687045
IFNg	rs74148555	C	T	0.947	0.3732	0.0774	2.64E-06	23.2488432
IFNg	rs78296352	G	T	0.971	-0.343	0.0652	1.38E-07	27.6753453

Supplementary Table 4: The instrumental variables associated with serum inflammatory factors.

EXPOSURE	SNP	EA	NEA	EAF	BETA	SE	P	F
IL10	rs10457128	G	A	0.361	0.0865	0.0172	5.24E-07	25.2915427
IL10	rs10493718	C	A	0.709	0.11	0.0222	7.16E-07	24.5515786
IL10	rs11206302	C	T	0.08	0.1189	0.0251	2.20E-06	22.4396597
IL10	rs2086656	C	T	0.305	0.0789	0.0171	3.78E-06	21.2893198
IL10	rs282258	C	T	0.575	-0.0992	0.0162	1.00E-09	37.4967231
IL10	rs3025021	C	T	0.632	-0.0947	0.0195	1.46E-06	23.5847206
IL10	rs35794877	C	A	0.508	-0.0794	0.0167	1.80E-06	22.6051848
IL10	rs41282660	G	A	0.132	0.1194	0.0255	3.72E-06	21.9244291
IL10	rs4349809	G	T	0.451	-0.2853	0.0165	5.77E-67	298.975537
IL10	rs465757	G	A	0.333	-0.084	0.0174	1.17E-06	23.3055886
IL10	rs4741748	G	A	0.428	0.0793	0.017	2.79E-06	21.759481
IL10	rs7088799	G	T	0.434	0.0852	0.0167	3.23E-07	26.0283266
IL12p70	rs10738760	G	A	0.497	-0.0896	0.0156	1.02E-08	32.9888231
IL12p70	rs13209117	G	A	0.716	-0.1002	0.0186	5.57E-08	29.0208117
IL12p70	rs17229494	G	A	0.087	0.1172	0.0257	4.93E-06	20.796439
IL12p70	rs200757	C	T	0.877	0.0985	0.0214	4.15E-06	21.1858023
IL12p70	rs282258	C	T	0.575	-0.073	0.0156	3.21E-06	21.8976003
IL12p70	rs41282644	G	A	0.936	-0.1473	0.0304	1.05E-06	23.4778502
IL12p70	rs4349809	G	T	0.451	-0.3777	0.0159	2.56E-124	564.286579
IL12p70	rs4734879	G	A	0.292	-0.0918	0.0189	1.22E-06	23.5918367
IL12p70	rs7088799	G	T	0.434	0.0998	0.0161	6.29E-10	38.4245978
IL12p70	rs71361173	G	T	0.113	-0.111	0.0239	3.06E-06	21.5700005
IL12p70	rs72831623	G	A	0.947	-0.1913	0.037	2.42E-07	26.7316947
IL12p70	rs782107	G	A	0.445	-0.075	0.0156	1.60E-06	23.1139053
IL12p70	rs79121401	C	T	0.019	-0.5548	0.1206	4.24E-06	21.1630515
IL13	rs117795020	G	A	0.982	0.3522	0.0716	9.86E-07	24.196506
IL13	rs12623722	G	A	0.718	0.1185	0.0258	4.19E-06	21.0958626
IL13	rs139083458	C	T	0.984	-0.9902	0.2107	2.81E-06	22.085985
IL13	rs142167313	C	T	0.019	0.313	0.0617	3.98E-07	25.7346548
IL13	rs27949	C	T	0.369	0.1168	0.0252	3.43E-06	21.4824893
IL13	rs6799107	C	T	0.214	0.1459	0.0301	1.25E-06	23.4951159
IL13	rs7073807	C	T	0.883	-0.1682	0.0356	2.37E-06	22.3229706
IL13	rs75995699	G	A	0.976	-0.3319	0.0698	2.64E-06	22.6101612
IL13	rs9472168	G	A	0.449	-0.4244	0.0248	1.08E-65	292.851457
IL16	rs116135478	G	A	0.018	-0.8206	0.166	3.66E-06	24.4369415
IL16	rs117217798	C	T	0.931	0.2036	0.0444	4.15E-06	21.0275952
IL16	rs117916513	G	A	0.979	0.502	0.0986	3.79E-07	25.9211106
IL16	rs1255143	C	T	0.434	-0.1306	0.0242	7.10E-08	29.1243084
IL16	rs12765671	G	A	0.983	0.6023	0.1318	4.84E-06	20.883097
IL16	rs144691581	G	A	0.985	-0.4882	0.0967	4.20E-07	25.4884016
IL16	rs1801020	G	A	0.775	-0.1733	0.0272	4.53E-10	40.5937635
IL16	rs4253283	C	T	0.7	-0.146	0.0262	1.75E-08	31.0529689
IL16	rs4513633	C	A	0.124	0.2239	0.0453	7.44E-07	24.4293428
IL16	rs4778636	G	A	0.925	0.7272	0.0633	1.11E-30	131.977629
IL16	rs9706053	C	T	0.984	-0.4582	0.0932	7.01E-07	24.1700943
IL17	rs117029961	G	A	0.989	-0.4585	0.1015	4.94E-06	20.4054697
IL17	rs117556572	C	T	0.989	0.5102	0.1099	3.28E-06	21.551898
IL17	rs1530455	C	T	0.592	-0.108	0.0173	4.87E-10	38.9722343
IL17	rs17106604	C	T	0.876	-0.1129	0.0225	6.37E-07	25.1780938

IL17	rs17282552	C	T	0.027	0.2001	0.0405	8.21E-07	24.4109191
IL17	rs184080173	C	T	0.063	-0.2384	0.0471	4.19E-07	25.6195023
IL17	rs187475560	C	T	0.988	0.2434	0.052	3.29E-06	21.9096006
IL17	rs34120897	C	A	0.097	0.1055	0.0232	3.63E-06	20.6789722
IL17	rs62191444	G	T	0.834	0.1136	0.0247	4.22E-06	21.1525513
IL17	rs78296352	G	T	0.971	-0.3027	0.0646	4.27E-06	21.9563329
IL17	rs78612928	C	T	0.197	-0.1037	0.0222	2.62E-06	21.8198401
IL17	rs79462337	G	T	0.02	-0.2097	0.0435	1.41E-06	23.2390488
IL18	rs10414578	C	T	0.879	0.1771	0.035	4.16E-07	25.6036
IL18	rs115267715	C	T	0.982	-0.4508	0.08	1.72E-08	31.753225
IL18	rs116383510	C	A	0.02	0.5426	0.1056	3.00E-07	26.4016909
IL18	rs11700536	C	T	0.638	-0.1156	0.025	4.21E-06	21.381376
IL18	rs117266781	C	T	0.989	-0.6841	0.1468	3.15E-06	21.7163619
IL18	rs144841621	C	T	0.989	-0.518	0.1141	3.81E-06	20.6104859
IL18	rs17229943	C	A	0.05	0.312	0.0463	1.62E-11	45.4095508
IL18	rs1852105	C	T	0.951	-0.3036	0.0661	4.32E-06	21.0960242
IL18	rs1979967	C	T	0.741	-0.1402	0.0286	9.45E-07	24.0305638
IL18	rs2729385	G	A	0.675	-0.1231	0.0262	3.79E-06	22.0756512
IL18	rs385076	C	T	0.647	0.2432	0.0248	1.66E-22	96.1664932
IL18	rs4482818	G	A	0.359	-0.1286	0.0244	1.45E-07	27.7780838
IL18	rs658805	G	A	0.687	-0.1226	0.0244	4.94E-07	25.2465063
IL18	rs71478720	C	T	0.782	0.2669	0.0276	3.71E-22	93.5145059
IL18	rs78623212	C	T	0.976	-0.8705	0.1778	6.71E-07	23.9703314
IL18	rs78716465	G	A	0.962	-0.3265	0.0682	1.63E-06	22.9191033
IL1b	rs115242021	C	A	0.919	-0.2326	0.0414	5.07E-07	31.5659409
IL1b	rs143319329	C	T	0.976	-0.2801	0.0715	2.00E-06	15.3466693
IL1b	rs1942793	G	T	0.526	-0.0717	0.0187	4.98E-06	14.7012783
IL1b	rs61335305	C	A	0.989	-0.2966	0.0724	1.90E-06	16.7828287
IL1b	rs62015704	G	A	0.121	-0.1082	0.0283	2.09E-06	14.6177877
IL1b	rs9898641	C	T	0.36	0.2032	0.0454	3.59E-06	20.0325254
IL1ra	rs1054402	C	T	0.734	-0.1311	0.027	1.13E-06	23.5764198
IL1ra	rs11627423	C	A	0.39	-0.1171	0.0247	2.12E-06	22.4760445
IL1ra	rs12121840	C	T	0.916	-0.2692	0.0571	2.43E-06	22.2268488
IL1ra	rs139005642	AAAAG	A	0.147	-0.1315	0.0277	1.97E-06	22.5367853
IL1ra	rs2809154	C	T	0.838	0.1786	0.0388	3.74E-06	21.1884632
IL1ra	rs56134659	G	A	0.474	0.1117	0.0237	2.44E-06	22.2131247
IL1ra	rs61335305	C	A	0.989	-0.4453	0.0908	1.00E-06	24.0510307
IL1ra	rs9623661	C	T	0.908	0.1966	0.0426	3.86E-06	21.2984417
IL2	rs10903540	G	A	0.034	0.1581	0.0343	4.19E-06	21.2459179
IL2	rs12051139	C	T	0.401	0.1131	0.0247	4.76E-06	20.966759
IL2	rs13412535	G	A	0.774	-0.1764	0.0332	1.18E-07	28.2306576
IL2	rs1534019	C	T	0.556	0.1187	0.0248	1.58E-06	22.9085751
IL2	rs170117	C	T	0.88	0.1617	0.0349	3.87E-06	21.4668927
IL2	rs2807544	G	A	0.566	-0.1175	0.0253	3.41E-06	21.5692325
IL2	rs4634519	G	A	0.312	0.1261	0.0269	2.77E-06	21.9748345
IL2	rs61335305	C	A	0.989	-0.4514	0.0918	7.32E-07	24.1789673
IL2	rs62124990	G	T	0.984	0.6961	0.1495	3.22E-06	21.6800801
IL2	rs7615304	G	A	0.651	0.1172	0.0242	1.21E-06	23.4544089
IL2	rs80336398	C	T	0.021	-0.4001	0.0858	2.82E-06	21.745156
IL2ra	rs11241559	G	T	0.781	0.1264	0.0266	2.00E-06	22.5803607
IL2ra	rs115360066	G	A	0.11	-0.1867	0.0379	8.06E-07	24.2666718

IL2ra	rs117244812	G	A	0.986	0.7064	0.1488	2.10E-06	22.5369696
IL2ra	rs12722497	C	A	0.856	-0.6279	0.0485	1.57E-38	167.609059
IL2ra	rs185231391	C	T	0.015	-0.8503	0.1809	1.47E-06	22.0936375
IL2ra	rs4733117	C	A	0.164	-0.1369	0.0292	2.63E-06	21.9806835
IL2ra	rs61705228	C	T	0.963	-0.3303	0.0716	3.99E-06	21.2809545
IL2ra	rs62135626	C	T	0.885	0.1661	0.035	1.99E-06	22.5218041
IL4	rs10512267	C	T	0.33	0.0824	0.0161	2.94E-07	26.1940512
IL4	rs116705532	G	T	0.017	0.4678	0.0978	1.76E-06	22.8792996
IL4	rs117146485	C	T	0.015	0.2924	0.0629	2.71E-06	21.6099343
IL4	rs17713451	G	A	0.835	-0.1274	0.0253	4.97E-07	25.3569967
IL4	rs2332982	G	A	0.142	-0.1118	0.0219	3.20E-07	26.0612581
IL4	rs6765768	G	A	0.392	-0.0795	0.0167	2.00E-06	22.6621607
IL4	rs73023729	G	A	0.977	0.1796	0.0366	9.03E-07	24.079668
IL4	rs7613691	G	A	0.053	-0.1775	0.0384	4.05E-06	21.3665432
IL4	rs79597994	C	T	0.972	0.5831	0.127	4.32E-06	21.08039
IL4	rs9508291	C	T	0.065	0.1676	0.0359	3.03E-06	21.7951133
IL4	rs9941733	G	A	0.175	-0.114	0.0229	6.88E-07	24.7821361
IL5	rs11680908	G	A	0.055	-0.2634	0.0554	2.03E-06	22.6053904
IL5	rs6737109	C	T	0.425	-0.116	0.0247	2.40E-06	22.0557623
IL5	rs72831687	G	A	0.983	0.5239	0.1109	1.69E-06	22.3168916
IL5	rs73040130	C	T	0.065	-0.2638	0.0529	6.00E-07	24.86785
IL5	rs7767396	G	A	0.451	-0.1515	0.0246	7.69E-10	37.9275729
IL6	rs114373846	C	T	0.987	-0.422	0.0904	3.32E-06	21.7915459
IL6	rs12024374	G	A	0.884	-0.1096	0.0236	3.35E-06	21.5673657
IL6	rs1333040	C	T	0.426	-0.0738	0.0158	3.17E-06	21.8171767
IL6	rs13412535	G	A	0.774	0.1164	0.0215	7.34E-08	29.3108924
IL6	rs72831623	G	A	0.947	-0.1973	0.0372	1.08E-07	28.1299066
IL6	rs73273528	C	T	0.966	-0.2672	0.0553	9.58E-07	23.3465464
IL6	rs76856708	C	T	0.038	-0.3289	0.07	2.61E-06	22.0765735
IL7	rs117509142	C	T	0.057	0.327	0.0688	1.99E-06	22.5901247
IL7	rs141425475	C	T	0.029	0.4781	0.1016	2.53E-06	22.143694
IL7	rs144701438	G	A	0.946	0.4819	0.0989	9.75E-07	23.742217
IL7	rs17091524	C	T	0.043	-0.4924	0.1013	1.91E-06	23.6274688
IL7	rs218260	C	T	0.788	0.1337	0.0286	2.93E-06	21.8539904
IL7	rs28793375	C	T	0.833	-0.1638	0.0361	4.46E-06	20.5879636
IL7	rs4320361	G	T	0.548	0.3245	0.0249	6.87E-39	169.836374
IL7	rs62006410	C	T	0.785	0.1557	0.0303	3.39E-07	26.4053524
IL7	rs75904417	C	A	0.141	0.1698	0.0349	1.16E-06	23.6714313
IL7	rs77318030	C	T	0.052	0.2921	0.0632	3.74E-06	21.3613629
IL7	rs77981494	C	T	0.016	0.5178	0.1064	1.07E-06	23.6832247
IL7	rs78346957	G	A	0.977	-0.4588	0.1007	4.51E-06	20.7581133
IL8	rs11634944	C	T	0.347	0.1214	0.0252	1.29E-06	23.2079239
IL8	rs12075	G	A	0.398	-0.12	0.0236	3.88E-07	25.8546395
IL8	rs141926526	C	A	0.048	0.6149	0.1308	2.57E-06	22.1000623
IL8	rs2673604	C	A	0.299	0.1266	0.0255	7.02E-07	24.6483045
IL9	rs117935362	G	A	0.985	0.5107	0.1114	4.85E-06	21.0165456
IL9	rs41294750	C	T	0.972	-0.3514	0.0748	2.37E-06	22.0698976
IL9	rs4880409	C	T	0.028	0.3355	0.0723	3.50E-06	21.5331941
IL9	rs61867538	C	T	0.973	-0.3566	0.0774	3.93E-06	21.2266157
IL9	rs7232268	G	A	0.952	-0.2759	0.0587	2.53E-06	22.0916014
IL9	rs7242404	G	A	0.702	0.1228	0.0264	3.27E-06	21.6365932

IL9	rs76963786	C	T	0.2865	0.914		0.0557	4.50E-07	26.4568943
IL10	rs10809307	C	T	0.1305	0.696	-	0.0282	3.64E-06	21.4152331
IL10	rs113831257	G	A	0.3592	0.958	-	0.0644	2.53E-08	31.1100652
IL10	rs11626201	C	A	0.1162	0.366	-	0.0245	1.93E-06	22.4946939
IL10	rs143799975	G	A	0.7984	0.013		0.1637	1.00E-06	23.7872205
IL10	rs181236764	C	T	0.4504	0.957	-	0.0964	3.22E-06	21.8294451
IL10	rs34383175	C	T	0.3153	0.973		0.0657	1.51E-06	23.0312337
IL10	rs397816	C	T	0.1237	0.426	-	0.0249	7.90E-07	24.6797471
IL10	rs75970138	G	A	0.485	0.987		0.104	1.53E-06	21.7478735
IL10	rs7645625	G	T	0.1086	0.432		0.0237	4.41E-06	20.9972761
IL10	rs79848609	C	A	0.2603	0.032	-	0.0537	8.75E-07	23.4963155
IL10	rs8112909	G	A	0.1426	0.192		0.0299	1.94E-06	22.7455621
IL10	rs9450351	C	T	0.2768	0.062		0.0489	1.48E-08	32.0416191
MIP1a	rs10835056	G	T	0.1194	0.756	-	0.0254	2.60E-06	22.0974022
MIP1a	rs116615337	G	A	0.1278	0.291	-	0.0279	4.82E-06	20.9823101
MIP1a	rs12690897	G	A	0.1248	0.719	-	0.0262	2.11E-06	22.6895869
MIP1a	rs184154340	G	A	0.331	0.959	-	0.0693	1.86E-06	22.8133739
MIP1a	rs34771762	G	A	0.249	0.074	-	0.0523	2.13E-06	22.6670664
MIP1a	rs57786342	G	A	0.1314	0.784	-	0.0285	4.06E-06	21.2569529
MIP1a	rs60198979	G	A	0.2146	0.914		0.0458	2.62E-06	21.9547491
MIP1a	rs6900267	C	A	0.2429	0.075		0.0519	2.89E-06	21.9038428
MIP1a	rs7232268	G	A	0.2821	0.952	-	0.0599	2.55E-06	22.1795396
MIP1b	rs113010081	C	T	0.5954	0.108		0.0236	3.85E-140	636.493034
MIP1b	rs113877493	C	T	0.6124	0.878		0.0218	1.62E-173	789.146031
MIP1b	rs116237296	G	A	0.5437	0.984	-	0.1115	7.23E-07	23.7776501
MIP1b	rs117453826	G	A	0.5774	0.016		0.0593	5.07E-22	94.8078226
MIP1b	rs12490293	C	T	0.0866	0.392	-	0.0168	2.12E-07	26.5715703
MIP1b	rs141102180	G	T	0.3225	0.982	-	0.0393	1.08E-16	67.34019
MIP1b	rs1437220	C	T	0.1478	0.05	-	0.0315	3.53E-06	22.0154598
MIP1b	rs1564708	C	T	0.1744	0.73		0.0188	2.87E-20	86.0552286
MIP1b	rs17138331	G	A	0.1391	0.094		0.0295	2.26E-06	22.2336225
MIP1b	rs2411161	C	T	0.1714	0.051	-	0.0367	3.14E-06	21.8116995
MIP1b	rs281749	C	T	0.0799	0.708	-	0.0171	3.17E-06	21.8323929
MIP1b	rs3760440	G	A	0.1236	0.331	-	0.0162	2.75E-14	58.2112483
MIP1b	rs62242409	C	T	0.1282	0.743	-	0.0193	3.43E-11	44.1226342

MIP1b	rs72791296	C	T<br>0.2369	0.941	-	0.0466	3.78E-07	25.8439141
MIP1b	rs72799710	C	T<br>0.1014	0.834		0.0218	3.21E-06	21.6353001
MIP1b	rs74810984	C	T<br>0.2206	0.022	-	0.0474	1.96E-06	21.6597945
MIP1b	rs76582507	G	A<br>0.3175	0.973	-	0.0677	3.26E-06	21.9942989
MIP1b	rs76583883	G	T<br>0.2317	0.968		0.0511	4.99E-06	20.559392
MIP1b	rs76776296	G	A<br>0.2997	0.037	-	0.0598	5.55E-07	25.1171939
MIP1b	rs9793308	G	A<br>0.0835	0.372	-	0.0178	2.52E-06	22.0055864
MIP1b	rs9850846	G	A<br>0.072	0.444	-	0.0157	4.48E-06	21.0312792
Eotaxin	rs11087905	C	A<br>0.0941	0.658	-	0.0189	5.48E-07	24.7888077
Eotaxin	rs112347425	C	T<br>0.158	0.893	-	0.0277	8.65E-09	32.5352865
Eotaxin	rs12075	G	A<br>0.1671	0.398	-	0.0156	1.33E-26	114.737056
Eotaxin	rs138125931	C	T<br>0.1292	0.868	-	0.0254	3.59E-07	25.8736437
Eotaxin	rs1476670	C	A<br>0.1007	0.793		0.0217	3.51E-06	21.5347321
Eotaxin	rs2024050	G	A<br>0.1728	0.9	-	0.0303	1.10E-08	32.5238702
Eotaxin	rs2210755	C	T<br>0.1104	0.081		0.0242	4.85E-06	20.8116932
Eotaxin	rs2211994	C	T<br>0.0885	0.75	-	0.0177	6.08E-07	25

Eotaxin	rs2228467	C	T	0.07	0.4163	0.0292	2.27E-46	203.257752
Eotaxin	rs2419841	C	T	0.119	0.1277	0.0279	4.98E-06	20.9494868
Eotaxin	rs5746492	G	A	0.181	-0.0954	0.0207	3.96E-06	21.2400756
Eotaxin	rs5754733	C	A	0.212	0.1042	0.0214	1.06E-06	23.7087082
Eotaxin	rs59808887	C	T	0.923	0.1673	0.0358	2.91E-06	21.838652
Eotaxin	rs75426604	C	A	0.869	0.1366	0.0291	2.53E-06	22.03512
Eotaxin	rs79722574	C	T	0.84	0.1113	0.0228	1.06E-06	23.829813
Eotaxin	rs80341932	G	A	0.292	-0.1016	0.0205	6.69E-07	24.562903
Eotaxin	rs9317045	C	A	0.146	-0.1182	0.0237	5.82E-07	24.873578
MCP1	rs10145849	G	A	0.651	0.0755	0.0162	3.41E-06	21.7202027
MCP1	rs10744620	C	T	0.639	-0.0788	0.0161	9.91E-07	23.9552486
MCP1	rs111995966	G	T	0.026	-0.1452	0.031	2.53E-06	21.9386472
MCP1	rs12073356	G	A	0.915	0.1426	0.0311	4.17E-06	21.0241416
MCP1	rs12075	G	A	0.398	-0.2185	0.0155	1.44E-44	198.719043
MCP1	rs146522229	C	T	0.976	0.5976	0.1177	3.56E-07	25.7791297
MCP1	rs2036297	G	A	0.657	-0.119	0.016	1.09E-13	55.3164063
MCP1	rs2288370	C	T	0.396	0.1031	0.0163	2.25E-10	40.0075652
MCP1	rs2712431	C	A	0.285	0.0787	0.0172	4.76E-06	20.9359451
MCP1	rs56212190	C	T	0.945	-0.181	0.0373	9.85E-07	23.5472116
MCP1	rs7197349	G	A	0.137	-0.0968	0.0206	2.62E-06	22.0808747
MCP1	rs7517040	G	A	0.718	0.0987	0.0191	2.44E-07	26.7034621
MCP1	rs7632755	G	A	0.92	-0.2938	0.0316	1.18E-20	86.4429178
MCP1	rs9317045	C	A	0.146	-0.1134	0.0236	1.52E-06	23.0888394
MCP3	rs10892381	C	T	0.3	-0.2412	0.0476	3.56E-07	25.6767884
MCP3	rs62492260	G	T	0.851	0.2788	0.058	1.54E-06	23.1062545
MCP3	rs73669117	G	A	0.015	0.6238	0.131	2.56E-06	22.6750446
MIG	rs111607343	G	A	0.962	0.521	0.1119	2.83E-06	21.6778034
MIG	rs11177248	G	A	0.939	-0.3073	0.067	4.45E-06	21.0365984
MIG	rs112337562	G	T	0.017	0.37	0.0796	2.98E-06	21.6061463
MIG	rs112861654	G	A	0.087	0.2765	0.0529	1.81E-07	27.3198888
MIG	rs117831247	C	T	0.981	0.8334	0.1754	2.16E-06	22.5760425
MIG	rs139010077	C	T	0.989	-0.4322	0.095	3.55E-06	20.6977108
MIG	rs1796086	C	T	0.094	0.2096	0.0403	2.23E-07	27.0503236
MIG	rs41272086	G	A	0.915	0.2226	0.0415	7.43E-08	28.770945
MIG	rs55876513	G	T	0.248	-0.166	0.0255	8.23E-11	42.3775471
MIG	rs5752128	C	T	0.095	0.1685	0.0369	4.34E-06	20.8519694
MIG	rs62562991	G	A	0.98	-0.6236	0.126	8.40E-07	24.4946435
MIG	rs6679677	C	A	0.909	-0.162	0.0329	8.86E-07	24.2458957
MIG	rs77086208	C	T	0.981	-0.3226	0.0698	3.83E-06	21.3608181
MIG	rs816960	C	T	0.741	0.1224	0.0244	5.01E-07	25.1642032
CTACK	rs116303454	G	A	0.971	-0.383	0.0816	3.27E-06	22.0301386
CTACK	rs135564	G	A	0.741	0.1893	0.0268	2.43E-12	49.8920834
CTACK	rs145902143	G	A	0.038	0.2838	0.0581	1.03E-06	23.8601142
CTACK	rs17321950	C	T	0.028	-0.5262	0.1122	3.46E-06	21.9945952
CTACK	rs2070074	G	A	0.102	-0.4467	0.0374	1.79E-32	142.655559
CTACK	rs3766110	C	A	0.233	0.1287	0.0278	3.86E-06	21.4322369
CTACK	rs55764737	C	T	0.023	-0.5313	0.0972	4.62E-08	29.8776959
CTACK	rs57338032	G	A	0.18	-0.1583	0.0317	6.23E-07	24.9369483
CTACK	rs7333764	C	T	0.973	-0.2773	0.0593	2.85E-06	21.8670578
CTACK	rs76395525	G	A	0.985	-0.5277	0.1083	9.55E-07	23.7419986
RANTES	rs112072646	G	A	0.969	-0.4286	0.0862	6.48E-07	24.7223529

RANTES	rs147509526	C	T	0.987	0.358	0.0717	6.93E-07	24.9303136
RANTES	rs2251660	C	A	0.145	-0.1829	0.0359	3.83E-07	25.9560447
RANTES	rs4940620	G	A	0.064	0.2494	0.054	3.54E-06	21.3307133
RANTES	rs62438851	G	A	0.135	0.1957	0.0414	2.33E-06	22.3450314
RANTES	rs7000423	C	T	0.674	0.1318	0.0253	1.82E-07	27.1387461
RANTES	rs72793342	G	A	0.792	0.1487	0.0308	1.48E-06	23.3088316
RANTES	rs74472919	C	T	0.981	-0.3313	0.0605	3.97E-08	29.9869381
RANTES	rs75613039	C	T	0.969	-0.37	0.081	4.81E-06	20.8657217
RANTES	rs818452	C	T	0.948	-0.2381	0.0505	2.36E-06	22.2298245
GROa	rs1113500	G	T	0.37	-0.1174	0.0244	1.57E-06	23.1502956
GROa	rs118158560	G	A	0.937	-0.2703	0.0594	3.42E-06	20.7070962
GROa	rs12075	G	A	0.398	-0.3751	0.0237	1.24E-55	250.494063
GROa	rs140734053	G	A	0.975	-0.7257	0.1561	3.58E-06	21.6126709
GROa	rs185768063	G	A	0.014	-0.3998	0.076	1.46E-07	27.6731371
GROa	rs188345231	C	T	0.988	-0.623	0.1323	4.34E-06	22.1746312
GROa	rs508977	G	T	0.234	0.3802	0.028	7.56E-42	184.377602
GROa	rs62024303	G	A	0.046	0.3053	0.0666	4.41E-06	21.0138269
GROa	rs78653452	G	T	0.988	0.7362	0.1558	1.21E-06	22.3283545
SDF1a	rs10474392	G	A	0.731	-0.0962	0.0178	1.24E-06	29.2085595
SDF1a	rs12407262	G	A	0.866	-0.1179	0.0266	3.99E-06	19.6455566
SDF1a	rs13400104	G	A	0.18	0.0647	0.0189	4.53E-06	11.7188489
SDF1a	rs139840550	G	A	0.952	-0.1834	0.0549	3.79E-06	11.1597374
SDF1a	rs149893336	G	A	0.02	0.5034	0.1081	4.52E-06	21.6857793
SDF1a	rs16872470	G	A	0.013	-0.5319	0.1025	2.81E-07	26.9285054
SDF1a	rs4581824	G	T	0.311	0.0701	0.0173	3.05E-06	16.4188914
SDF1a	rs482700	G	A	0.286	0.0893	0.0203	1.57E-06	19.351331
SDF1a	rs67689854	C	A	0.885	0.0681	0.0195	3.07E-06	12.196213
TNFa	rs10834997	G	A	0.36	0.1247	0.0258	1.33E-06	23.3611111
TNFa	rs111332265	G	A	0.072	0.3766	0.0754	6.63E-07	24.9469777
TNFa	rs8121916	C	A	0.774	-0.1306	0.0278	2.72E-06	22.0697169
TNFb	rs10925040	C	T	0.611	-0.1755	0.0373	2.67E-06	22.137908
TNFb	rs753274	C	T	0.464	0.1736	0.0371	2.77E-06	21.8953364
TNFb	rs7629875	G	A	0.058	-0.3766	0.0774	1.37E-06	23.6743852
TNFb	rs78296352	G	T	0.971	-1.2215	0.1366	4.76E-21	79.9623491
TRAIL	rs11618126	G	A	0.015	-0.8908	0.1914	1.46E-06	21.6609026
TRAIL	rs11657269	G	A	0.119	-0.1188	0.026	4.78E-06	20.8778698
TRAIL	rs11699445	G	T	0.387	-0.0746	0.0161	3.27E-06	21.4696964
TRAIL	rs13115587	C	A	0.917	-0.1019	0.0218	3.10E-06	21.849192
TRAIL	rs13185784	G	A	0.71	-0.0846	0.0183	3.90E-06	21.3716743
TRAIL	rs13278062	G	T	0.497	-0.0801	0.0157	3.57E-07	26.0294941
TRAIL	rs141603697	C	T	0.987	-0.7202	0.0741	6.61E-22	94.4647584
TRAIL	rs146783010	G	A	0.011	0.6016	0.135	4.83E-06	19.8585767
TRAIL	rs148051545	C	T	0.987	0.3921	0.0848	3.86E-06	21.3797184
TRAIL	rs193112415	C	T	0.014	1.0421	0.0623	2.15E-62	279.796771
TRAIL	rs57396456	C	T	0.034	0.5626	0.0518	1.25E-27	117.961405
TRAIL	rs62093514	C	T	0.974	-1.0618	0.0552	6.86E-82	370.004739
TRAIL	rs6878823	C	T	0.772	0.0879	0.0189	3.48E-06	21.6298816
TRAIL	rs73039026	C	A	0.016	0.2999	0.0635	2.02E-06	22.3051671
TRAIL	rs74488044	G	A	0.94	-0.3489	0.0335	2.61E-25	108.470671
TRAIL	rs747324	C	T	0.641	0.0855	0.0178	1.61E-06	23.0723709
TRAIL	rs74778900	C	T	0.986	-0.5906	0.0532	2.59E-28	123.243386

TRAIL	rs75928541	G	A	0.983	-0.275	0.0593	4.24E-06	21.5058197
TRAIL	rs79287178	G	A	0.974	0.4317	0.0421	9.12E-25	105.147731
MIF	rs113218956	G	A	0.989	0.8948	0.1879	2.26E-06	22.6776679
MIF	rs118055855	C	T	0.013	-0.6907	0.15	4.13E-06	21.2029551
MIF	rs12594190	G	A	0.247	-0.1355	0.0267	3.70E-07	25.7546746
MIF	rs13142904	C	T	0.931	0.223	0.0425	2.56E-07	27.5316263
MIF	rs141009259	C	T	0.013	0.6178	0.1322	2.47E-06	21.8390075
MIF	rs5751777	C	T	0.389	0.1342	0.0249	7.09E-08	29.0473379
MIF	rs78098071	C	T	0.019	0.4867	0.0918	1.78E-07	28.108478
IFNg	rs112783231	G	A	0.971	0.2408	0.0511	1.96E-06	22.2060424
IFNg	rs113600793	C	A	0.96	-0.1829	0.0373	8.95E-07	24.0441676
IFNg	rs115729819	G	A	0.021	-0.2484	0.0515	1.38E-06	23.2642323
IFNg	rs11843756	G	T	0.029	-0.184	0.0393	3.09E-06	21.9205045
IFNg	rs12420286	C	T	0.029	-0.2376	0.0501	2.08E-06	22.4914482
IFNg	rs1867282	C	T	0.674	-0.0774	0.0166	3.15E-06	21.7403106
IFNg	rs2073438	G	A	0.729	-0.0898	0.0188	1.68E-06	22.8158669
IFNg	rs4730203	G	A	0.862	0.0983	0.0203	1.32E-06	23.4484943
IFNg	rs6902031	G	A	0.888	0.1166	0.0242	1.41E-06	23.214876
IFNg	rs7073753	C	T	0.567	-0.0821	0.0167	8.39E-07	24.1687045
IFNg	rs74148555	C	T	0.947	0.3732	0.0774	2.64E-06	23.2488432
IFNg	rs78296352	G	T	0.971	-0.343	0.0652	1.38E-07	27.6753453

Supplementary Table 5: A total of 792 confounding factors for this study.

snp	trait
rs1260326	Granulocyte count
rs1260326	Granulocyte percentage of myeloid white cells
rs1260326	Hematocrit
rs1260326	Hemoglobin concentration
rs1260326	High light scatter percentage of red cells
rs1260326	High light scatter reticulocyte count
rs1260326	Immature fraction of reticulocytes
rs1260326	Lymphocyte count
rs1260326	Mean corpuscular hemoglobin concentration
rs1260326	Mean platelet volume
rs1260326	Monocyte percentage of white cells
rs1260326	Myeloid white cell count
rs1260326	Neutrophil count
rs1260326	Neutrophil percentage of granulocytes
rs1260326	Platelet count
rs1260326	Plateletcrit
rs1260326	Red blood cell count
rs1260326	Red cell distribution width
rs1260326	Reticulocyte count
rs1260326	Reticulocyte fraction of red cells
rs1260326	Sum basophil neutrophil counts
rs1260326	Sum neutrophil eosinophil counts
rs1260326	White blood cell count
rs1260326	DPA 22:5n3
rs1260326	Palmitic acid 16:0
rs1260326	Palmitoleic acid 16:1n7
rs1260326	log eGFR creatinine in non diabetics
rs1260326	log eGFR creatinine
rs1260326	log eGFR creatinine
rs1260326	Type II diabetes
rs1260326	Height
rs1260326	Height
rs1260326	Height
rs1260326	Hip circumference in males
rs1260326	Low density lipoprotein
rs1260326	Total cholesterol
rs1260326	Total cholesterol
rs1260326	Triglycerides
rs1260326	Triglycerides
rs1260326	2 hour glucose
rs1260326	2 hour glucose tolerance test
rs1260326	2 hour glucose tolerance test
rs1260326	APOA1 apolipoprotein AI
rs1260326	APOA1 assay lipoprotein fraction concentration
rs1260326	APOA1 assay lipoprotein fraction concentration in fasting sample
rs1260326	APOB apolipoprotein B
rs1260326	APOC3 apolipoprotein C III
rs1260326	Albumin
rs1260326	Alkaline phosphatase ALP in plasma
rs1260326	Breast size bra cup size in women
rs1260326	C reactive protein
rs1260326	C reactive protein
rs1260326	C reactive protein
rs1260326	C2orf16 gene expression in liver
rs1260326	Crohns disease
rs1260326	EIF2B4 gene expression in peripheral blood leucocytes
rs1260326	FVII
rs1260326	FVII activity
rs1260326	FVII in plasma
rs1260326	Fasting blood glucose
rs1260326	Fasting blood glucose
rs1260326	Fasting blood glucose
rs1260326	Fasting blood glucose
rs1260326	Fasting insulin
rs1260326	Gamma glutamyl transferase
rs1260326	HDL cholesterol mean size lipoprotein fraction concentration
rs1260326	HDL cholesterol small lipoprotein fraction concentration
rs1260326	HDL cholesterol total lipoprotein fraction concentration
rs1260326	HDL cholesterol total lipoprotein fraction concentration in fasting sample
rs1260326	HOMA IR

rs1260326	HOMA IR
rs1260326	HOMA IR
rs1260326	Hypertriglyceridemia
rs1260326	Hypertriglyceridemia
rs1260326	IFT172 expression in Lymphocytes lymphoblastoid cell lines tissue
rs1260326	IFT172 gene expression in adipose tissue
rs1260326	IFT172 gene expression in human liver
rs1260326	IFT172 gene expression in human omental fat
rs1260326	IFT172 gene expression in liver
rs1260326	IFT172 gene expression in peripheral blood leucocytes
rs1260326	IFT172 trancript levels in human subcutaneous fat
rs1260326	Lipoprotein associated phospholipase A2 mass Lp2
rs1260326	Metabolic syndrome domains Elevated plasma glucose
rs1260326	Metabolic syndrome domains Multivariate analysis
rs1260326	NRBP1 gene expression in peripheral blood leucocytes
rs1260326	Plasma C reactive protein female
rs1260326	Plasma beta trace protein levels
rs1260326	Plasma docosapentaenoic acid levels
rs1260326	Plasma palmitoleic acid
rs1260326	Plasma protein C levels
rs1260326	Platelet count PLT
rs1260326	Serum albumin
rs1260326	Serum albumin
rs1260326	Serum creatinine
rs1260326	Serum creatinine estimated glomerular filtration rate eGFR
rs1260326	Serum creatinine estimated glomerular filtration rate eGFR
rs1260326	Serum creatinine estimated glomerular filtration rate eGFR age 65
rs1260326	Serum creatinine estimated glomerular filtration rate eGFR females
rs1260326	Serum creatinine estimated glomerular filtration rate eGFR no diabetes
rs1260326	Serum creatinine estimated glomerular filtration rate eGFR no hypertension
rs1260326	Serum creatinine estimated glomerular filtration rate eGFR without diabetes
rs1260326	Serum creatinine estimated glomerular filtration rate eGFR without hypertension
rs1260326	Serum urate
rs1260326	Serum urate
rs1260326	Serum urate
rs1260326	Total cholesterol
rs1260326	Total cholesterol
rs1260326	Total cholesterol
rs1260326	Total protein
rs1260326	Triglycerides
rs1260326	Triglycerides
rs1260326	Triglycerides
rs1260326	Triglycerides
rs1260326	Triglycerides
rs1260326	Triglycerides
rs1260326	Triglycerides
rs1260326	Triglycerides
rs1260326	Triglycerides
rs1260326	Triglycerides
rs1260326	Triglycerides
rs1260326	Triglycerides
rs1260326	Triglycerides by NMR lipoprotein fraction
rs1260326	Triglycerides by NMR lipoprotein fraction in fasting sample
rs1260326	Triglycerides change with statins
rs1260326	Triglycerides females
rs1260326	Triglycerides in females
rs1260326	Triglycerides in males
rs1260326	Triglycerides lipoprotein fraction
rs1260326	Triglycerides lipoprotein fraction in fasting sample
rs1260326	Triglycerides ln
rs1260326	Triglycerides ln
rs1260326	Triglycerides log mmoll
rs1260326	Triglycerides males
rs1260326	Triglycerides mmoll
rs1260326	Uric acid
rs1260326	VLDL cholesterol large lipoprotein fraction concentration
rs1260326	VLDL cholesterol large lipoprotein fraction concentration in fasting sample
rs1260326	VLDL cholesterol mean size lipoprotein fraction concentration
rs1260326	VLDL cholesterol mean size lipoprotein fraction concentration in fasting sample
rs1260326	VLDL cholesterol medium lipoprotein fraction concentration
rs1260326	VLDL cholesterol medium lipoprotein fraction concentration in fasting sample
rs1260326	VLDL cholesterol total lipoprotein fraction concentration

rs1260326	Serum urate
rs1260326	log Leptin
rs1260326	log Leptin adjusted for BMI
rs1260326	2 hour fasting glucose
rs1260326	2 hour fasting glucose
rs1260326	Fasting glucose
rs1260326	Fasting glucose
rs1260326	log Fasting insulin
rs1260326	log Fasting insulin adjusted for BMI
rs1260326	log HOMA-IR
rs1260326	Alcohol consumption
rs1260326	Alcohol consumption in current drinkers
rs1260326	Blood metabolite levels
rs1260326	Blood metabolite levels
rs1260326	Blood metabolite levels
rs1260326	Blood metabolite ratios
rs1260326	C reactive protein levels
rs1260326	C reactive protein levels or total cholesterol levels pleiotropy
rs1260326	C reactive protein levels or triglyceride levels pleiotropy
rs1260326	Caffeine metabolism plasma 17 dimethylxanthine paraxanthine to 137 trimethylxanthine caffeine ratio
rs1260326	Cardiovascular disease risk factors
rs1260326	Cholesterol total
rs1260326	Cholesterol total
rs1260326	Cholesterol total
rs1260326	Chronic kidney disease
rs1260326	Coffee consumption cups per day
rs1260326	Crohns disease
rs1260326	Gallstone disease
rs1260326	Glomerular filtration rate
rs1260326	Glomerular filtration rate
rs1260326	Glomerular filtration rate creatinine
rs1260326	Glomerular filtration rate in non diabetics creatinine
rs1260326	Glycemic traits pregnancy
rs1260326	Glycemic traits pregnancy
rs1260326	Gout
rs1260326	Gout
rs1260326	Granulocyte count
rs1260326	Hematocrit
rs1260326	Hematological and biochemical traits
rs1260326	High light scatter reticulocyte percentage of red cells
rs1260326	Hypertriglyceridemia
rs1260326	Hypertriglyceridemia
rs1260326	Inflammatory bowel disease
rs1260326	Inflammatory bowel disease
rs1260326	Lipid metabolism phenotypes
rs1260326	Lipid metabolism phenotypes
rs1260326	Lipid metabolism phenotypes
rs1260326	Lipid metabolism phenotypes
rs1260326	Lipid metabolism phenotypes
rs1260326	Lipid metabolism phenotypes
rs1260326	Lipid traits
rs1260326	Lipoprotein associated phospholipase A2 activity and mass
rs1260326	Liver enzyme levels gamma glutamyl transferase
rs1260326	Lymphocyte counts
rs1260326	Metabolic traits
rs1260326	Metabolite levels
rs1260326	Metabolite levels
rs1260326	Metabolite levels small molecules and protein measures
rs1260326	Myeloid white cell count
rs1260326	Neutrophil count
rs1260326	Non albumin protein levels
rs1260326	Nonalcoholic fatty liver disease
rs1260326	Plasma lactate levels
rs1260326	Plasma lactate levels
rs1260326	Platelet count
rs1260326	Platelet counts
rs1260326	Plateletcrit
rs1260326	Red cell distribution width
rs1260326	Renal overload gout
rs1260326	Resting heart rate
rs1260326	Serum albumin level
rs1260326	Serum albumin level

rs1260326	Serum albumin level
rs1260326	Serum alpha1 antitrypsin levels
rs1260326	Serum total protein level
rs1260326	Serum total protein level
rs1260326	Sum basophil neutrophil counts
rs1260326	Sum neutrophil eosinophil counts
rs1260326	Triglyceride levels
rs1260326	Triglycerides
rs1260326	Triglycerides
rs1260326	Triglycerides
rs1260326	Triglycerides
rs1260326	Triglycerides
rs1260326	Two hour glucose challenge
rs1260326	Urate levels
rs1260326	Urate levels in overweight individuals
rs1260326	Urate levels in overweight individuals
rs1260326	Waist circumference and related phenotypes
rs1260326	White blood cell count
rs1260326	Alcohol intake frequency
rs1260326	Alcohol intake versus 10 years previously
rs1260326	Arm fat-free mass left
rs1260326	Arm fat-free mass right
rs1260326	Arm predicted mass left
rs1260326	Arm predicted mass right
rs1260326	Basal metabolic rate
rs1260326	Cholelithiasis
rs1260326	Comparative height size at age 10
rs1260326	Daytime dozing or sleeping
rs1260326	Diabetes diagnosed by doctor
rs1260326	Height
rs1260326	Hip circumference
rs1260326	Impedance of arm left
rs1260326	Impedance of arm right
rs1260326	Impedance of leg left
rs1260326	Impedance of leg right
rs1260326	Impedance of whole body
rs1260326	Leg fat-free mass left
rs1260326	Leg fat-free mass right
rs1260326	Leg predicted mass left
rs1260326	Leg predicted mass right
rs1260326	Medication for cholesterol, blood pressure or diabetes: cholesterol lowering medication
rs1260326	No treatment with medication for cholesterol, blood pressure, diabetes, or take exogenous hormones
rs1260326	Number of self-reported non-cancer illnesses
rs1260326	Pulse rate
rs1260326	Pulse rate
rs1260326	Relative age of first facial hair
rs1260326	Self-reported diabetes
rs1260326	Self-reported gout
rs1260326	Self-reported high cholesterol
rs1260326	Sitting height
rs1260326	Sodium in urine
rs1260326	Treatment with allopurinol
rs1260326	Treatment with atorvastatin
rs1260326	Treatment with cholesterol lowering medication
rs1260326	Treatment with lipitor 10mg tablet
rs1260326	Treatment with metformin
rs1260326	Treatment with simvastatin
rs1260326	Trunk fat-free mass
rs1260326	Trunk predicted mass
rs1260326	Weight
rs1260326	Whole body fat-free mass
rs1260326	Whole body water mass
rs1260326	Triglycerides
rs1260326	Testosterone
rs1260326	Age at menopause
rs1260326	Triglycerides
rs1260326	Blood proteins
rs1260326	C reactive protein
rs1260326	Cholesterol
rs1260326	Gamma glutamyltransferase
rs1260326	Glucose tolerance test
rs1260326	Hypertriglyceridemia

rs1260326	Kidney diseases
rs1260326	Metabolism
rs1260326	Triglycerides
rs1260326	Triglycerides
rs1260326	Triglycerides
rs1260326	Triglycerides
rs1260326	Triglycerides
rs1260326	Triglycerides
rs17041868	Eosinophil percentage of granulocytes
rs17041868	Eosinophil percentage of white cells
rs17041868	Hip circumference
rs17041868	Pulse rate
rs1751956	Forced vital capacity
rs17602430	Height
rs17602430	Birth weight of first child
rs17602430	Height
rs1832664	Atopic dermatitis
rs2657879	Fasting blood glucose
rs2657879	Fasting glucose
rs2657879	Amino acid levels
rs2657879	Blood metabolite levels
rs2657879	Blood metabolite levels
rs2657879	Metabolic traits
rs2657879	Metabolite levels small molecules and protein measures
rs2657879	Metabolism
rs3094600	Basophil count
rs3094600	Eosinophil count
rs3094600	Granulocyte count
rs3094600	Hematocrit
rs3094600	Hemoglobin concentration
rs3094600	High light scatter percentage of red cells
rs3094600	High light scatter reticulocyte count
rs3094600	Lymphocyte count
rs3094600	Lymphocyte percentage of white cells
rs3094600	Monocyte count
rs3094600	Myeloid white cell count
rs3094600	Neutrophil count
rs3094600	Platelet count
rs3094600	Plateletcrit
rs3094600	Red blood cell count
rs3094600	Reticulocyte count
rs3094600	Reticulocyte fraction of red cells
rs3094600	Sum basophil neutrophil counts
rs3094600	Sum eosinophil basophil counts
rs3094600	Sum neutrophil eosinophil counts
rs3094600	White blood cell count
rs3094600	IgA deficiency
rs3094600	Triglycerides
rs3094600	Primary sclerosing cholangitis
rs3094600	Arm fat-free mass left
rs3094600	Arm fat-free mass right
rs3094600	Arm predicted mass left
rs3094600	Arm predicted mass right
rs3094600	Basal metabolic rate
rs3094600	Comparative body size at age 10
rs3094600	Doctor diagnosed sarcoidosis
rs3094600	Forced expiratory volume in 1-second, predicted percentage
rs3094600	Gonarthrosis
rs3094600	Impedance of leg left
rs3094600	Impedance of leg right
rs3094600	Impedance of whole body
rs3094600	Intestinal malabsorption
rs3094600	Leg fat-free mass left
rs3094600	Leg fat-free mass right
rs3094600	Leg predicted mass left
rs3094600	Leg predicted mass right
rs3094600	Long-standing illness, disability or infirmity
rs3094600	Medication for cholesterol, blood pressure or diabetes: insulin
rs3094600	Medication for pain relief, constipation, heartburn: none of the above
rs3094600	Medication for pain relief, constipation, heartburn: paracetamol
rs3094600	Mouth or teeth dental problems: dentures
rs3094600	Number of days or week of moderate physical activity 10+ minutes

rs3094600	Number of incorrect matches in round
rs3094600	Other serious medical condition or disability diagnosed by doctor
rs3094600	Self-reported adrenocortical insufficiency or addisons disease
rs3094600	Self-reported ankylosing spondylitis
rs3094600	Self-reported fracture shaft of femur
rs3094600	Self-reported hyperthyroidism or thyrotoxicosis
rs3094600	Self-reported hypothyroidism or myxoedema
rs3094600	Self-reported malabsorption or coeliac disease
rs3094600	Self-reported psoriasis
rs3094600	Self-reported sarcoidosis
rs3094600	Self-reported sjogrens syndrome or sicca syndrome
rs3094600	Self-reported systemic lupus erythematosis or sle
rs3094600	Self-reported type 1 diabetes
rs3094600	Sitting height
rs3094600	Started insulin within one year diagnosis of diabetes
rs3094600	Treatment with carbimazole
rs3094600	Treatment with insulin
rs3094600	Treatment with insulin product
rs3094600	Treatment with levothyroxine sodium
rs3094600	Treatment with thyroxine product
rs3094600	Trunk fat-free mass
rs3094600	Trunk predicted mass
rs3094600	Unspecified haematuria
rs3094600	Weight
rs3094600	Whole body fat-free mass
rs3094600	Whole body water mass
rs3094600	Rheumatoid arthritis
rs3094600	Schizophrenia
rs35592	Pulse rate
rs3931670	Basophil count
rs3931670	Granulocyte count
rs3931670	Granulocyte percentage of myeloid white cells
rs3931670	Lymphocyte count
rs3931670	Mean corpuscular hemoglobin
rs3931670	Mean corpuscular volume
rs3931670	Monocyte percentage of white cells
rs3931670	Myeloid white cell count
rs3931670	Neutrophil count
rs3931670	Platelet count
rs3931670	Platelet distribution width
rs3931670	Plateletcrit
rs3931670	Red blood cell count
rs3931670	Sum basophil neutrophil counts
rs3931670	Sum neutrophil eosinophil counts
rs3931670	White blood cell count
rs3931670	Height
rs3931670	Height
rs3931670	Height
rs3931670	Low density lipoprotein
rs3931670	Low density lipoprotein
rs3931670	Total cholesterol
rs3931670	Total cholesterol
rs3931670	Height
rs3931670	LDL cholesterol
rs3931670	Rheumatoid arthritis
rs3931670	Total cholesterol
rs3931670	Primary sclerosing cholangitis
rs3931670	Arm fat-free mass left
rs3931670	Arm fat-free mass right
rs3931670	Arm predicted mass left
rs3931670	Arm predicted mass right
rs3931670	Asthma
rs3931670	Basal metabolic rate
rs3931670	Comparative height size at age 10
rs3931670	Forced vital capacity
rs3931670	Forced vital capacity, best measure
rs3931670	Hayfever, allergic rhinitis or eczema
rs3931670	Height
rs3931670	Intestinal malabsorption
rs3931670	Leg fat-free mass left
rs3931670	Leg fat-free mass right
rs3931670	Leg predicted mass left

rs3931670	Leg predicted mass right
rs3931670	Medication for pain relief, constipation, heartburn: none of the above
rs3931670	No blood clot, bronchitis, emphysema, asthma, rhinitis, eczema or allergy diagnosed by doctor
rs3931670	Psoriasis
rs3931670	Self-reported ankylosing spondylitis
rs3931670	Self-reported asthma
rs3931670	Self-reported malabsorption or coeliac disease
rs3931670	Self-reported psoriasis
rs3931670	Self-reported psoriatic arthropathy
rs3931670	Sitting height
rs3931670	Started insulin within one year diagnosis of diabetes
rs3931670	Treatment with calcipotriol
rs3931670	Treatment with dovobet ointment
rs3931670	Treatment with dovonex 50micrograms or g cream
rs3931670	Treatment with insulin
rs3931670	Treatment with insulin product
rs3931670	Trunk fat-free mass
rs3931670	Trunk predicted mass
rs3931670	Whole body fat-free mass
rs3931670	Whole body water mass
rs3931670	Rheumatoid arthritis
rs3931670	Rheumatoid arthritis
rs3931670	Rheumatoid arthritis
rs4587207	Mean platelet volume
rs4587207	Height
rs4587207	HIV 1 control
rs4587207	Rheumatoid arthritis
rs4587207	Type 1 diabetes combined control dataset
rs4587207	Sleep duration
rs4587207	Frequency of tenseness or restlessness in last 2 weeks
rs4587207	Intestinal malabsorption
rs4587207	No blood clot, bronchitis, emphysema, asthma, rhinitis, eczema or allergy diagnosed by doctor
rs4587207	Psoriasis
rs4587207	Self-reported hyperthyroidism or thyrotoxicosis
rs4587207	Self-reported hypothyroidism or myxoedema
rs4587207	Self-reported malabsorption or coeliac disease
rs4587207	Self-reported psoriasis
rs4587207	Self-reported psoriatic arthropathy
rs4587207	Started insulin within one year diagnosis of diabetes
rs4587207	Treatment with calcipotriol
rs4587207	Treatment with dovobet ointment
rs4587207	Treatment with insulin product
rs4587207	Treatment with levothyroxine sodium
rs4587207	Treatment with thyroxine product
rs4587207	Rheumatoid arthritis
rs4587207	Rheumatoid arthritis
rs4587207	Rheumatoid arthritis
rs4981468	Pulse rate
rs12954899	Arm fat mass left
rs12954899	Arm fat mass right
rs12954899	Arm fat percentage left
rs12954899	Arm fat percentage right
rs12954899	Body fat percentage
rs12954899	Body mass index
rs12954899	Hip circumference
rs12954899	Leg fat mass left
rs12954899	Leg fat mass right
rs12954899	Leg fat percentage left
rs12954899	Leg fat percentage right
rs12954899	Leg fat-free mass left
rs12954899	Leg predicted mass left
rs12954899	Trunk fat mass
rs12954899	Trunk fat percentage
rs12954899	Waist circumference
rs12954899	Weight
rs12954899	Whole body fat mass
rs6854832	Forced vital capacity
rs6854832	Forced vital capacity, best measure
rs6854832	Sitting height
rs7150776	Late onset Alzheimers disease
rs10494322	Lymphocyte count
rs10494322	Monocyte count

rs10828691	Red cell distribution width
rs10828691	Height
rs10828691	Arm fat-free mass left
rs10828691	Arm fat-free mass right
rs10828691	Arm predicted mass left
rs10828691	Arm predicted mass right
rs10828691	Basal metabolic rate
rs10828691	Birth weight
rs10828691	Impedance of arm left
rs10828691	Impedance of arm right
rs10828691	Impedance of leg left
rs10828691	Impedance of leg right
rs10828691	Impedance of whole body
rs10828691	Leg fat-free mass left
rs10828691	Leg fat-free mass right
rs10828691	Leg predicted mass right
rs10828691	Trunk fat-free mass
rs10828691	Trunk predicted mass
rs10828691	Whole body fat-free mass
rs10828691	Whole body water mass
rs11921640	Home area population density: Scotland large urban area
rs13431652	Fasting blood glucose
rs13431652	Fasting blood glucose in non diabetics
rs13431652	HOMA B
rs13431652	Fasting glucose
rs13431652	log HOMA-B
rs13431652	HbA1C
rs13431652	HbA1c
rs13431652	HbA1c
rs16856261	Fasting blood glucose
rs16856261	Fasting glucose
rs16856261	Fasting glucose
rs16856261	HbA1c
rs16856261	HbA1c
rs17616642	Arm fat mass left
rs17616642	Arm fat mass right
rs17616642	Arm fat percentage left
rs17616642	Arm fat percentage right
rs17616642	Body fat percentage
rs17616642	Body mass index
rs17616642	Leg fat mass left
rs17616642	Leg fat mass right
rs17616642	Leg fat percentage left
rs17616642	Leg fat percentage right
rs17616642	Trunk fat mass
rs17616642	Trunk fat percentage
rs17616642	Waist circumference
rs17616642	Weight
rs17616642	Whole body fat mass
rs1857095	Insulin sensitivity index adjusted for BMI interaction
rs1857095	Modified Stumvoll Insulin Sensitivity Index interaction with BMI
rs4572706	Self-reported primary bone cancer
rs741160	Schizophrenia
rs9363753	Qualifications: college or university degree
rs9363753	Time spent watching television
rs9804190	Bipolar disorder
rs9804190	Bipolar disorder
rs9915927	Otosclerosis
rs10153390	Nap during day
rs11160112	Arm fat mass left
rs11160112	Arm fat mass right
rs11160112	Arm fat percentage left
rs11160112	Arm fat percentage right
rs11160112	Arm fat-free mass left
rs11160112	Arm fat-free mass right
rs11160112	Arm predicted mass left
rs11160112	Arm predicted mass right
rs11160112	Basal metabolic rate
rs11160112	Body fat percentage
rs11160112	Body mass index
rs11160112	Comparative body size at age 10
rs11160112	Hip circumference

rs11160112	Impedance of arm left
rs11160112	Impedance of arm right
rs11160112	Impedance of leg left
rs11160112	Impedance of leg right
rs11160112	Impedance of whole body
rs11160112	Leg fat mass left
rs11160112	Leg fat mass right
rs11160112	Leg fat percentage left
rs11160112	Leg fat percentage right
rs11160112	Leg fat-free mass left
rs11160112	Leg fat-free mass right
rs11160112	Leg predicted mass left
rs11160112	Leg predicted mass right
rs11160112	Trunk fat mass
rs11160112	Trunk fat percentage
rs11160112	Trunk fat-free mass
rs11160112	Trunk predicted mass
rs11160112	Waist circumference
rs11160112	Weight
rs11160112	Wheeze or whistling in the chest in last year
rs11160112	Whole body fat mass
rs11160112	Whole body fat-free mass
rs11160112	Whole body water mass
rs115174302	Rheumatoid arthritis
rs1277442	Red cell distribution width
rs1795963	Mean platelet volume
rs1795963	Waist circumference
rs493519	Mean platelet volume
rs493519	Platelet distribution width
rs493519	Plateletcrit
rs493519	Blood metabolite levels
rs493519	Arm fat-free mass left
rs493519	Arm fat-free mass right
rs493519	Arm predicted mass left
rs493519	Arm predicted mass right
rs493519	Basal metabolic rate
rs493519	Impedance of whole body
rs493519	Leg fat-free mass left
rs493519	Leg fat-free mass right
rs493519	Leg predicted mass left
rs493519	Leg predicted mass right
rs493519	Sitting height
rs493519	Trunk fat-free mass
rs493519	Trunk predicted mass
rs493519	Whole body fat-free mass
rs493519	Whole body water mass
rs6470588	Renal cell carcinoma
rs6860069	Eosinophil count
rs6860069	Eosinophil percentage of white cells
rs6860069	Hematocrit
rs6860069	Hemoglobin concentration
rs6860069	Red blood cell count
rs6860069	Sum eosinophil basophil counts
rs6860069	Chronic kidney disease
rs6860069	log eGFR creatinine in non diabetics
rs6860069	log eGFR creatinine
rs6860069	log eGFR creatinine
rs6860069	Allergic disease
rs6860069	Activated partial thromboplastin time
rs6860069	Factor XII antigen
rs6860069	Serum creatinine
rs6860069	Calculus of kidney and ureter
rs6860069	Hayfever, allergic rhinitis or eczema
rs6860069	Height
rs6860069	No blood clot, bronchitis, emphysema, asthma, rhinitis, eczema or allergy diagnosed by doctor
rs6860069	Number of self-reported non-cancer illnesses
rs6860069	Sitting height
rs7934036	Eosinophil count
rs7934036	Eosinophil percentage of granulocytes
rs7934036	Eosinophil percentage of white cells
rs7934036	Mean platelet volume
rs7934036	Neutrophil percentage of granulocytes

rs7934036	Sum eosinophil basophil counts
rs7934036	log eGFR creatinine in non diabetics
rs7934036	log eGFR creatinine
rs7934036	log eGFR creatinine
rs7934036	Atopic dermatitis
rs7934036	Allergic disease
rs7934036	Waist circumference adjusted for smoking in females
rs7934036	Serum creatinine
rs7934036	Serum urate
rs7934036	Serum urate
rs7934036	Comparative body size at age 10
rs7934036	Hayfever, allergic rhinitis or eczema
rs7934036	Heel bone mineral density
rs7934036	Heel bone mineral density left
rs7934036	Heel bone mineral density right
rs7934036	No blood clot, bronchitis, emphysema, asthma, rhinitis, eczema or allergy diagnosed by doctor
rs7934036	Self-reported gout
rs7934036	Self-reported hypertension
rs7934036	Treatment with allopurinol
rs7934036	Vascular or heart problems diagnosed by doctor: high blood pressure
rs967725	Treatment with indivina 1mg or 2.5mg tablet
rs11117784	Energy expenditure 24h
rs11675864	Arm fat-free mass left
rs11675864	Arm predicted mass left
rs11865472	Stabilized warfarin dose
rs4073054	APOA2 apolipoprotein AII
rs4073054	HDL cholesterol medium lipoprotein fraction concentration
rs4073054	HDL cholesterol medium lipoprotein fraction concentration in fasting sample
rs4073054	Blood metabolite levels
rs4073054	Overall health rating
rs10926975	Red cell distribution width
rs10926975	Height tails
rs10926975	Diastolic blood pressure
rs10926975	Medication for pain relief, constipation, heartburn: none of the above
rs10926975	Medication for pain relief, constipation, heartburn: paracetamol
rs10926975	Pain type experienced in last month: headache
rs10926975	Systemic sclerosis
rs10926975	Years of educational attainment
rs13293620	Self-reported pernicious anaemia
rs2710833	Nonalcoholic fatty liver disease Aspartate aminotransferase
rs2710833	Non alcoholic fatty liver disease histology AST
rs2710833	Nonalcoholic fatty liver disease
rs4602889	Arm fat-free mass left
rs4602889	Arm predicted mass left
rs4602889	Basal metabolic rate
rs4602889	Body mass index
rs4602889	Impedance of arm left
rs4602889	Impedance of arm right
rs4602889	Impedance of whole body
rs4602889	Trunk fat-free mass
rs4602889	Trunk predicted mass
rs4602889	Whole body fat-free mass
rs4602889	Whole body water mass
rs6003971	Height
rs6920798	Body fat percentage
rs6920798	Body mass index
rs6920798	Ever smoked
rs6920798	Leg fat mass left
rs6920798	Leg fat mass right
rs6920798	Leg fat percentage left
rs6920798	Leg fat percentage right
rs6920798	Long-standing illness, disability or infirmity
rs6920798	Past tobacco smoking
rs6920798	Smoking status: previous
rs6920798	Trunk fat percentage
rs6920798	Whole body fat mass
rs7127674	Open wound of forearm
rs9572772	Bipolar disorder combined control dataset
rs9572772	Cause of death: cerebrum, except lobes and ventricles
rs9572772	Cause of death: heart failure, unspecified
rs9572772	Home area population density: England or Wales - village, sparse
rs9572772	Scoliosis

rs9572772	Treatment with movelat cream
rs1572603	Self-reported kidney stone or ureter stone/bladder stone
rs10979172	Self-reported leukaemia
rs11043310	Mean platelet volume
rs11065202	Mean corpuscular hemoglobin
rs11065202	Mean corpuscular volume
rs11065202	Red blood cell count
rs11065202	Red cell distribution width
rs11065202	Mean corpuscular volume
rs11065202	Red blood cell count RBC
rs11065202	UNC119B Trancript NM032661 expression in Blood tissue
rs11065390	Cause of death: pharynx, unspecified
rs1171617	Height
rs1171617	Serum urate
rs1171617	Serum urate
rs1171617	Blood metabolite levels
rs1171617	Comparative height size at age 10
rs1171617	Forced expiratory volume in 1-second, predicted
rs1171617	Height
rs1171617	Leg fat-free mass right
rs1171617	Leg predicted mass right
rs1171617	Self-reported gout
rs1171617	Sitting height
rs1171617	Treatment with allopurinol
rs1171617	Trunk fat-free mass
rs1171617	Trunk predicted mass
rs1171617	Whole body fat-free mass
rs1171617	Whole body water mass
rs12304179	Mean platelet volume
rs12304179	Platelet distribution width
rs12304179	No-wear time bias adjusted acceleration standard deviation
rs12304179	Trunk fat percentage
rs12368199	Mean corpuscular hemoglobin
rs12368199	Mean corpuscular volume
rs12368199	Red cell distribution width
rs12369156	Mean corpuscular hemoglobin
rs12369156	Mean corpuscular volume
rs12369156	Arm fat mass left
rs12369156	Arm fat mass right
rs12369156	Arm fat percentage left
rs12369156	Arm fat percentage right
rs12369156	Arm fat-free mass left
rs12369156	Arm fat-free mass right
rs12369156	Arm predicted mass left
rs12369156	Arm predicted mass right
rs12369156	Basal metabolic rate
rs12369156	Body fat percentage
rs12369156	Height
rs12369156	Hip circumference
rs12369156	Leg fat mass left
rs12369156	Leg fat mass right
rs12369156	Leg fat percentage right
rs12369156	Leg fat-free mass left
rs12369156	Leg fat-free mass right
rs12369156	Leg predicted mass left
rs12369156	Leg predicted mass right
rs12369156	Treatment with cilest tablet
rs12369156	Trunk fat mass
rs12369156	Trunk fat percentage
rs12369156	Trunk fat-free mass
rs12369156	Trunk predicted mass
rs12369156	Waist circumference
rs12369156	Weight
rs12369156	Whole body fat mass
rs12369156	Whole body fat-free mass
rs12369156	Whole body water mass
rs12369156	Amyotrophic lateral sclerosis
rs12423303	Waist hip ratio
rs12423303	Waist hip ratio
rs12423303	Body fat percentage
rs12423303	Diastolic blood pressure
rs12423303	Trunk fat mass

rs12423303	Trunk fat percentage

Supplementary Table 6: Two-sample MR analysis results for psoriasis and male reproductive system diseases.

Exposure	Outcome	Nsnp	b	se	pval	or	or_lci95	or_uci95
Psoriasis	Male infertility	16	-0.07018	0.0536	0.190407	0.932223	0.839256	1.035489
	Abnormal spermatozoa	16	0.133405	0.047466	0.004946	1.142713	1.041198	1.254126
	Sexual dysfunction	16	-0.09933	0.152573	0.515007	0.90544	0.671408	1.221048
	Erectile dysfunction	16	0.003408	0.041847	0.935086	1.003414	0.924399	1.089183
	Testicular dysfunction	16	0.20261	0.082974	0.014612	1.224595	1.040791	1.440859
Psoriasis vulgaris	Male infertility	10	-0.09858	0.050775	0.052191	0.90612	0.820285	1.000937
	Abnormal spermatozoa	10	0.128312	0.045591	0.004887	1.136908	1.039722	1.243178
	Sexual dysfunction	10	-0.15721	0.142625	0.270343	0.854523	0.646129	1.130131
	Erectile dysfunction	10	-0.0113	0.0399	0.776986	0.988762	0.914384	1.06919
	Testicular dysfunction	10	0.042893	0.079002	0.587174	1.043826	0.894088	1.218642
Arthropathic psoriasis	Male infertility	4	-0.05895	0.060151	0.327062	0.942753	0.837908	1.060717
	Abnormal spermatozoa	4	0.106451	0.052905	0.044207	1.112323	1.002762	1.233855
	Sexual dysfunction	4	-0.20773	0.135647	0.125674	0.812428	0.622759	1.059863
	Erectile dysfunction	4	-0.03338	0.046764	0.475333	0.967169	0.882462	1.060008
	Testicular dysfunction	4	0.102083	0.093068	0.272702	1.107475	0.922811	1.329092
Guttate psoriasis	Male infertility	3	-0.03223	0.020361	0.113458	0.968286	0.930405	1.007709
	Abnormal spermatozoa	3	0.040319	0.018103	0.025932	1.041143	1.004849	1.078747
	Sexual dysfunction	3	-0.0528	0.041708	0.205562	0.948573	0.874114	1.029374
	Erectile dysfunction	3	0.005125	0.015972	0.74831	1.005138	0.974159	1.037102
	Testicular dysfunction	3	0.061167	0.03159	0.052836	1.063076	0.99925	1.130979
Other and unspecified psorias	Male infertility	3	-0.04081	0.042738	0.339607	0.96001	0.88287	1.04389
	Abnormal spermatozoa	3	0.08143	0.037474	0.029781	1.084837	1.008013	1.167515
	Sexual dysfunction	3	-0.09598	0.087241	0.271251	0.908481	0.765693	1.077896
	Erectile dysfunction	3	-0.01216	0.033875	0.719716	0.987918	0.924455	1.055738
	Testicular dysfunction	3	0.060637	0.066055	0.358637	1.062513	0.933483	1.209377
Psoriasis(UKB)	Abnormal spermatozoa	20	8.005413	3.584748	0.025537	2997.137	2.662609	3373694
	Testicular dysfunction	20	7.13067	6.630164	0.282156	1249.714	0.002839	5.5E+08

Supplementary Table 7: Two-sample MR analysis results for the causal effect of male reproductive health on psoriasis.

Exposure	Outcome	nsnp	b	se	pval	or	or_lci95	or_uci95
Male infertility	Psoriasis	14	0.008441255	0.02055509	0.68131824	1.008476983	0.968655044	1.049936023
	Psoriasis vulgaris	14	0.022525023	0.025600002	0.378922358	1.022780627	0.972727807	1.075408971
	Arthropathic psoriasis	14	0.017648854	0.032758908	0.590059534	1.017805515	0.95450862	1.085299854
	Guttate psoriasis	14	0.09058147	0.103502073	0.381483721	1.094810698	0.893791643	1.341040135
	Other and unspecified psoriasis	14	0.046065365	0.05024904	0.359278272	1.047142855	0.94892762	1.155523495
Abnormal spermatozoa	Psoriasis	12	0.029306964	0.031336021	0.349660299	1.029740639	0.968398466	1.094968467
	Psoriasis vulgaris	12	0.040192562	0.040946379	0.326301817	1.041011214	0.960729543	1.12800148
	Arthropathic psoriasis	12	0.036872681	0.04417951	0.40393707	1.037560911	0.951496607	1.131409861
	Guttate psoriasis	12	-0.111924005	0.139762248	0.42323751	0.894112203	0.679867053	1.175872001
	Other and unspecified psoriasis	12	-0.046535132	0.064107754	0.467906643	0.954531025	0.841822301	1.082329937
Sexual dysfunction	Psoriasis	4	-0.023271526	0.019498576	0.232674113	0.976997168	0.940363539	1.015057929
	Psoriasis vulgaris	4	-0.051090948	0.024284583	0.035392149	0.950192248	0.906024682	0.996512928
	Arthropathic psoriasis	4	-0.022858024	0.045257459	0.613512174	0.977401242	0.894435359	1.06806286
	Guttate psoriasis	4	0.019524968	0.094090408	0.835609366	1.019716827	0.847985085	1.226227235
	Other and unspecified psoriasis	4	0.00550246	0.0450562	0.902800522	1.005517626	0.920528154	1.098353909
Erectile dysfunction	Psoriasis	13	-0.071505658	0.029712254	0.016101576	0.93099101	0.878322294	0.986818014
	Psoriasis vulgaris	13	-0.049806546	0.036990308	0.178149193	0.951413461	0.884876183	1.022953936
	Arthropathic psoriasis	13	-0.091853937	0.054253662	0.090446738	0.912238384	0.820213084	1.01458863
	Guttate psoriasis	13	-0.084368915	0.158909597	0.595471463	0.919092127	0.673119956	1.254947696
	Other and unspecified psoriasis	13	-0.114282928	0.068752228	0.09646401	0.892005547	0.779550899	1.020682417
	Psoriasis(UKB)	9	-0.000125704	0.000350544	0.719895898	0.999874304	0.99918756	1.000561519
Testicular dysfunction	Psoriasis	4	0.000423208	0.024613898	0.986281942	1.000423298	0.953305336	1.049870107
	Psoriasis vulgaris	4	0.009369769	0.034779192	0.787616257	1.009413802	0.942897737	1.080622197
	Arthropathic psoriasis	4	0.005235555	0.03953149	0.894636185	1.005249285	0.93030186	1.08623466
	Guttate psoriasis	4	-0.077855269	0.119071273	0.51320565	0.925098307	0.732541734	1.168270472
	Other and unspecified psoriasis	4	0.082808089	0.056673176	0.143973902	1.086333309	0.972124559	1.213959721

Supplementary Table 8: The causal effect of serum metabolites on abnormal spermatozoa.

Exposure	Outcome	nsnp	b	se	pval	or	or_lci95	or_uci95
M00053.m	Abnormal spermatozoa	8	3.456399157	2.25201	0.12483	31.7026	0.38384	2618.45
M00054.m	Abnormal spermatozoa	196	-0.418722324	0.72974	0.5661	0.65789	0.15739	2.7499
M00059.m	Abnormal spermatozoa	7	1.052634075	2.47913	0.67113	2.86519	0.02223	369.348
M00060.m	Abnormal spermatozoa	214	0.900769242	0.71423	0.20724	2.4615	0.60707	9.98073
M00063.m	Abnormal spermatozoa	48	0.017976404	0.87819	0.98367	1.01814	0.18208	5.693
M00064.m	Abnormal spermatozoa	6	2.17061066	3.94367	0.58204	8.76363	0.00385	19934.3
M00513.m	Abnormal spermatozoa	26	-1.964638786	1.43964	0.17236	0.14021	0.00834	2.35621
M00527.m	Abnormal spermatozoa	12	1.135465982	1.20247	0.34503	3.11262	0.29482	32.8617
M00542.m	Abnormal spermatozoa	10	0.427086128	0.33327	0.20002	1.53278	0.79762	2.94555
M00553.x	Abnormal spermatozoa	16	-0.041617115	0.10834	0.70089	0.95924	0.77572	1.18618
M00569.x	Abnormal spermatozoa	13	-0.047869194	0.24666	0.84612	0.95326	0.58782	1.54587
M00575.m	Abnormal spermatozoa	5	-0.079679776	0.59777	0.89396	0.92341	0.28613	2.98009
M00577.m	Abnormal spermatozoa	3	-1.227947014	1.88532	0.51484	0.29289	0.00728	11.7904
M00584.m	Abnormal spermatozoa	29	-0.250618742	0.61535	0.6838	0.77832	0.233	2.59989
M00599.m	Abnormal spermatozoa	14	-0.803299793	0.68667	0.24206	0.44785	0.11658	1.72041
M00606.m	Abnormal spermatozoa	22	0.328275531	1.34937	0.80779	1.38857	0.09862	19.5512
M01105.m	Abnormal spermatozoa	17	0.354199464	1.05928	0.73809	1.42504	0.17871	11.3632
M01107.m	Abnormal spermatozoa	19	-0.251943376	0.65588	0.70088	0.77729	0.21493	2.81109
M01110.m	Abnormal spermatozoa	30	-1.01444274	0.58699	0.08395	0.3626	0.11476	1.14574
M01114.m	Abnormal spermatozoa	17	0.349031551	0.31414	0.26654	1.41769	0.76591	2.62413
M01121.m	Abnormal spermatozoa	8	-0.580535846	1.11341	0.60209	0.5596	0.06311	4.96172
M01123.m	Abnormal spermatozoa	13	0.077325312	0.1994	0.69817	1.08039	0.73089	1.59703
M01125.m	Abnormal spermatozoa	18	0.989978354	1.53682	0.51946	2.69118	0.13236	54.7157
M01284.m	Abnormal spermatozoa	20	0.263820129	1.02968	0.79778	1.30189	0.17302	9.79622
M01299.m	Abnormal spermatozoa	35	0.585182574	1.00417	0.56006	1.79532	0.25083	12.8501
M01301.m	Abnormal spermatozoa	25	-0.585180202	1.3726	0.66987	0.55701	0.0378	8.20806
M01302.m	Abnormal spermatozoa	22	0.830008757	1.69393	0.62414	2.29334	0.0829	63.441
M01303.m	Abnormal spermatozoa	17	1.690300509	1.27511	0.18497	5.42111	0.44534	65.9904
M01336.m	Abnormal spermatozoa	28	-0.864031579	0.96307	0.36963	0.42146	0.06382	2.78315
M01356.m	Abnormal spermatozoa	16	0.401456567	0.7613	0.59796	1.494	0.33599	6.64325
M01358.m	Abnormal spermatozoa	44	0.394445928	0.92105	0.66847	1.48356	0.24394	9.02241
M01359.m	Abnormal spermatozoa	21	0.43987464	1.00661	0.66212	1.55251	0.21587	11.1656
M01361.m	Abnormal spermatozoa	20	0.580674204	0.95225	0.542	1.78724	0.27644	11.5547
M01365.m	Abnormal spermatozoa	25	-0.204734887	0.69729	0.76905	0.81486	0.20775	3.19618
M01444.m	Abnormal spermatozoa	16	0.468399477	0.60754	0.44072	1.59744	0.4856	5.25498
M01493.m	Abnormal spermatozoa	11	-0.581389385	1.36421	0.66998	0.55912	0.03857	8.10488
M01494.m	Abnormal spermatozoa	25	0.149821972	0.90654	0.86873	1.16163	0.19652	6.86644
M01508.m	Abnormal spermatozoa	25	-0.738593706	0.62031	0.23378	0.47779	0.14165	1.61157
M01515.x	Abnormal spermatozoa	18	-0.107525094	0.07828	0.16954	0.89805	0.77032	1.04696
M01558.m	Abnormal spermatozoa	41	0.69514794	0.75794	0.35906	2.00401	0.45366	8.85257
M01561.m	Abnormal spermatozoa	11	-0.436900737	0.80001	0.58499	0.64604	0.13467	3.09915
M01564.m	Abnormal spermatozoa	49	-0.278828197	0.77216	0.71802	0.75667	0.16658	3.43702
M01572.m	Abnormal spermatozoa	17	-0.307273923	1.03057	0.76558	0.73545	0.09757	5.54361
M01573.m	Abnormal spermatozoa	13	-0.230797325	0.35073	0.51051	0.7939	0.39922	1.57875
M01585.m	Abnormal spermatozoa	23	2.881830787	1.65911	0.08239	17.8469	0.69072	461.131
M01604.m	Abnormal spermatozoa	26	-1.464415607	1.0665	0.16972	0.23121	0.02859	1.86998
M01605.m	Abnormal spermatozoa	11	0.471238239	0.51658	0.36165	1.60198	0.58202	4.40936
M01638.m	Abnormal spermatozoa	20	1.1574114	1.12914	0.30535	3.18169	0.34794	29.0941
M01640.m	Abnormal spermatozoa	14	-0.116538284	0.15925	0.46429	0.89	0.65138	1.21603
M01644.m	Abnormal spermatozoa	37	-0.504753322	0.80189	0.52905	0.60365	0.12537	2.9065
M01645.m	Abnormal spermatozoa	41	-0.34265883	0.83307	0.68084	0.70988	0.13869	3.63337
M01649.m	Abnormal spermatozoa	6	-0.80101424	2.08685	0.7011	0.44887	0.00751	26.8217
M01670.m	Abnormal spermatozoa	14	0.362388719	1.15168	0.75302	1.43676	0.15033	13.7314
M01712.m	Abnormal spermatozoa	13	0.354493534	1.25907	0.77829	1.42546	0.12084	16.8151
M01769.m	Abnormal spermatozoa	40	0.690144827	0.97991	0.48125	1.994	0.29215	13.6096
M01898.m	Abnormal spermatozoa	36	0.185284906	0.83669	0.82474	1.20356	0.23349	6.20405

M02132.m	Abnormal spermatozoa	48	-0.375966924	0.80617	0.64096	0.68663	0.14142	3.33382
M02137.m	Abnormal spermatozoa	32	-0.167301592	0.26829	0.53289	0.84594	0.5	1.43124
M02342.m	Abnormal spermatozoa	16	0.565627484	0.5519	0.30543	1.76055	0.59684	5.19325
M02730.m	Abnormal spermatozoa	23	2.186386154	0.79197	0.00577	8.90298	1.88538	42.0408
M02734.m	Abnormal spermatozoa	48	0.094737707	0.75581	0.90025	1.09937	0.24991	4.83616
M03127.m	Abnormal spermatozoa	24	1.012091719	0.7142	0.15645	2.75135	0.67859	11.1554
M03141.m	Abnormal spermatozoa	29	-1.182398721	0.71528	0.09832	0.30654	0.07545	1.24552
M03147.m	Abnormal spermatozoa	3	0.387658134	2.15689	0.85736	1.47353	0.0215	101.004
M10642.m	Abnormal spermatozoa	32	-0.694331674	0.75693	0.35898	0.49941	0.11328	2.20174
M11438.m	Abnormal spermatozoa	4	-0.911153278	2.712	0.73689	0.40206	0.00198	81.8073
M12017.m	Abnormal spermatozoa	16	1.165636568	0.81926	0.15479	3.20796	0.64397	15.9806
M12035.m	Abnormal spermatozoa	37	-1.349392088	0.93331	0.14823	0.2594	0.04164	1.61591
M12067.m	Abnormal spermatozoa	28	0.682901687	1.24871	0.58446	1.97961	0.17126	22.8825
M12129.m	Abnormal spermatozoa	25	0.50975676	0.8488	0.54813	1.66489	0.31541	8.78815
M12261.m	Abnormal spermatozoa	13	-0.157923495	0.27197	0.56146	0.85392	0.50108	1.45519
M12593.m	Abnormal spermatozoa	25	1.536658971	1.29694	0.23608	4.64903	0.36592	59.0665
M12626.m	Abnormal spermatozoa	9	0.101300092	1.48189	0.9455	1.10661	0.06062	20.2025
M12768.m	Abnormal spermatozoa	20	-0.388622683	0.66838	0.56095	0.67799	0.18293	2.51282
M12774.m	Abnormal spermatozoa	33	1.111621216	0.77448	0.1512	3.03928	0.66607	13.8682
M15122.m	Abnormal spermatozoa	18	1.317509801	0.83619	0.11512	3.73411	0.72512	19.2294
M15140.m	Abnormal spermatozoa	45	-0.695088346	0.8491	0.413	0.49903	0.09448	2.63567
M15335.m	Abnormal spermatozoa	13	-0.140495849	0.31103	0.65148	0.86893	0.47231	1.59859
M15365.m	Abnormal spermatozoa	13	-0.244646626	1.24748	0.84452	0.78298	0.0679	9.02874
M15488.m	Abnormal spermatozoa	18	0.804467556	1.76764	0.64903	2.23551	0.06994	71.4534
M15500.m	Abnormal spermatozoa	232	-0.507256202	0.65928	0.44165	0.60215	0.16539	2.19224
M15506.m	Abnormal spermatozoa	25	0.043340976	1.57414	0.97803	1.04429	0.04774	22.8433
M15630.m	Abnormal spermatozoa	25	0.318659382	0.25513	0.21167	1.37528	0.8341	2.26759
M15650.m	Abnormal spermatozoa	8	-2.23338618	2.87596	0.43741	0.10716	0.00038	30.0688
M15676.m	Abnormal spermatozoa	35	1.535196727	0.83103	0.0647	4.64224	0.91063	23.6653
M15677.m	Abnormal spermatozoa	8	0.102107935	0.40366	0.8003	1.1075	0.50205	2.44312
M15749.m	Abnormal spermatozoa	16	0.530967747	0.45252	0.24065	1.70058	0.70049	4.12849
M15753.x	Abnormal spermatozoa	15	-0.02432144	0.44368	0.95628	0.97597	0.40904	2.32868
M15778.x	Abnormal spermatozoa	41	-0.633604868	0.94963	0.50463	0.53068	0.08251	3.41324
M15958.m	Abnormal spermatozoa	10	-0.188419958	0.59417	0.75116	0.82827	0.25847	2.65421
M15990.m	Abnormal spermatozoa	19	0.031298867	0.42838	0.94176	1.03179	0.4456	2.38913
M15996.m	Abnormal spermatozoa	4	-2.566847636	1.30897	0.04988	0.07678	0.0059	0.99874
M16634.m	Abnormal spermatozoa	10	-1.230466152	0.71675	0.08603	0.29216	0.0717	1.19049
M16816.m	Abnormal spermatozoa	14	0.974698897	1.08761	0.37015	2.65037	0.31442	22.3408
M16818.m	Abnormal spermatozoa	11	0.471679864	0.77279	0.54163	1.60268	0.3524	7.28894
M16821.m	Abnormal spermatozoa	22	-1.028619634	0.68897	0.13544	0.3575	0.09264	1.37955
M16822.m	Abnormal spermatozoa	21	1.198375369	0.88435	0.17539	3.31473	0.5857	18.7595

M16823.m	Abnormal spermatozoa	11	0.258935866	0.19832	0.19167	1.29555	0.8783	1.91103
M17799.x	Abnormal spermatozoa	129	-0.08924664	0.06741	0.18553	0.91462	0.80142	1.04381
M17805.m	Abnormal spermatozoa	9	-0.319057044	0.96208	0.74017	0.72683	0.11028	4.79047
M17807.m	Abnormal spermatozoa	24	0.539426921	0.33423	0.10654	1.71502	0.89078	3.30196
M17945.m	Abnormal spermatozoa	36	-0.287930324	0.75429	0.70267	0.74981	0.17096	3.28867
M18254.x	Abnormal spermatozoa	13	-0.280816004	0.37892	0.45864	0.75517	0.35933	1.58704
M18281.x	Abnormal spermatozoa	13	0.038236327	0.13026	0.7691	1.03898	0.80488	1.34116
M18283.m	Abnormal spermatozoa	10	-0.752869038	0.56054	0.17923	0.47101	0.157	1.4131
M18335.x	Abnormal spermatozoa	6	0.672230216	0.40423	0.09631	1.9586	0.88687	4.32544
M18349.m	Abnormal spermatozoa	18	-0.470202074	0.71611	0.51143	0.62488	0.15354	2.54305
M18357.m	Abnormal spermatozoa	7	-0.30470889	0.59696	0.60975	0.73734	0.22883	2.37581
M18369.m	Abnormal spermatozoa	37	-0.685500325	0.97234	0.48081	0.50384	0.07492	3.38816
M18392.x	Abnormal spermatozoa	5	0.432191372	0.76189	0.57054	1.54063	0.34607	6.85857
M18394.x	Abnormal spermatozoa	15	-0.129196735	0.35519	0.71605	0.8788	0.43807	1.76293
M18467.m	Abnormal spermatozoa	12	-1.344192355	0.72694	0.06444	0.26075	0.06273	1.08394
M18474.m	Abnormal spermatozoa	13	0.055275802	0.13479	0.68174	1.05683	0.81147	1.37639
M18476.m	Abnormal spermatozoa	9	-0.602126965	0.60247	0.31759	0.54765	0.16814	1.78376

M18477.m	Abnormal spermatozoa	7	0.020242127	0.25877	0.93765	1.02045	0.6145	1.69457
M18494.m	Abnormal spermatozoa	13	0.026443832	0.32607	0.93536	1.0268	0.54191	1.94554
M18497.m	Abnormal spermatozoa	16	-0.178849143	0.22496	0.4266	0.83623	0.53807	1.29962
M18929.m	Abnormal spermatozoa	13	1.235150238	0.98301	0.20893	3.4389	0.5008	23.6141
M19323.m	Abnormal spermatozoa	6	0.174160879	1.0765	0.87148	1.19025	0.14431	9.81689
M19324.m	Abnormal spermatozoa	7	0.321961087	1.04548	0.75812	1.37983	0.17779	10.7092
M19362.m	Abnormal spermatozoa	23	-0.38848269	1.03537	0.7075	0.67808	0.08912	5.15955
M19364.m	Abnormal spermatozoa	22	0.316861329	0.57588	0.58216	1.37281	0.44403	4.2443
M19368.m	Abnormal spermatozoa	12	-0.840712804	1.49559	0.57403	0.4314	0.023	8.09007
M19396.m	Abnormal spermatozoa	8	-0.755136502	0.7914	0.33999	0.46995	0.09963	2.21668
M19414.m	Abnormal spermatozoa	14	0.014174031	0.89939	0.98743	1.01427	0.17401	5.912
M19415.m	Abnormal spermatozoa	6	0.035640943	1.01601	0.97202	1.03628	0.14146	7.59141
M19934.m	Abnormal spermatozoa	40	0.708509716	0.7156	0.32213	2.03096	0.49954	8.25724
M20489.m	Abnormal spermatozoa	38	2.192469476	0.94039	0.01973	8.95731	1.41809	56.5786
M20675.m	Abnormal spermatozoa	37	0.795343837	0.56364	0.15822	2.2152	0.7339	6.68635
M20699.x	Abnormal spermatozoa	27	0.012799722	0.49018	0.97917	1.01288	0.38753	2.64733
M21044.m	Abnormal spermatozoa	18	0.404110022	0.70417	0.56605	1.49797	0.37679	5.95528
M21047.m	Abnormal spermatozoa	18	-0.936972273	1.1689	0.42279	0.39181	0.03964	3.87316
M21049.m	Abnormal spermatozoa	15	-0.313846585	0.28845	0.27657	0.73063	0.41511	1.28597
M21127.m	Abnormal spermatozoa	13	1.212581766	0.95028	0.20195	3.36215	0.52206	21.6527
M21151.x	Abnormal spermatozoa	10	-0.132700962	0.22346	0.55262	0.87573	0.56514	1.35701
M21184.m	Abnormal spermatozoa	15	-0.257591483	0.51107	0.61424	0.77291	0.28386	2.10455
M21188.m	Abnormal spermatozoa	25	-1.003646996	0.71242	0.1589	0.36654	0.09072	1.48098
M21630.m	Abnormal spermatozoa	27	-0.769334346	0.65787	0.24223	0.46332	0.12761	1.68218
M22030.m	Abnormal spermatozoa	20	-1.606187027	0.80322	0.04553	0.20065	0.04157	0.96862
M22032.m	Abnormal spermatozoa	16	-0.677039428	0.90307	0.45343	0.50812	0.08655	2.98317
M22116.m	Abnormal spermatozoa	14	-0.354884077	1.12503	0.75242	0.70125	0.07731	6.36095
M22130.m	Abnormal spermatozoa	20	1.36956233	0.77384	0.07676	3.93363	0.86315	17.9267
M22138.m	Abnormal spermatozoa	8	0.623793515	0.78925	0.42932	1.86599	0.39727	8.76464
M22175.m	Abnormal spermatozoa	8	-0.335762027	0.86775	0.6988	0.71479	0.13048	3.91581
M22177.m	Abnormal spermatozoa	61	0.457554755	0.80715	0.5708	1.58021	0.32483	7.68733
M22189.m	Abnormal spermatozoa	9	0.042143995	0.88453	0.962	1.04304	0.18424	5.90512
M22481.m	Abnormal spermatozoa	38	-0.373911954	0.49675	0.45162	0.68804	0.25988	1.82162
M22548.m	Abnormal spermatozoa	15	2.621484522	1.10599	0.01778	13.7561	1.5742	120.207
M22649.m	Abnormal spermatozoa	13	1.36641088	1.31711	0.29954	3.92125	0.29667	51.8293
M22842.m	Abnormal spermatozoa	9	0.003028074	0.22901	0.98945	1.00303	0.6403	1.57126
M24074.m	Abnormal spermatozoa	20	0.43407484	0.94532	0.6461	1.54353	0.24202	9.84435
M25459.m	Abnormal spermatozoa	32	0.173279683	0.53257	0.7449	1.1892	0.41872	3.37742
M25599.m	Abnormal spermatozoa	21	-0.121857856	0.76247	0.87302	0.88527	0.19863	3.94554
M27256.m	Abnormal spermatozoa	37	-0.420096456	1.15831	0.71684	0.65698	0.06786	6.361
M27273.m	Abnormal spermatozoa	14	-0.324333467	0.97158	0.73851	0.72301	0.10768	4.85475

M27278.m	Abnormal spermatozoa	23	-0.389498189	0.74697	0.60206	0.6774	0.15668	2.92873
M27447.m	Abnormal spermatozoa	14	0.393456231	0.39807	0.32295	1.48209	0.67926	3.23383
M27513.m	Abnormal spermatozoa	22	0.672193989	0.50201	0.18057	1.95853	0.73216	5.23905
M27531.m	Abnormal spermatozoa	17	-0.135040522	0.25328	0.59391	0.87368	0.53181	1.43531
M27672.m	Abnormal spermatozoa	5	-0.532442937	0.74805	0.47661	0.58717	0.13552	2.54402
M27710.m	Abnormal spermatozoa	22	0.164587209	0.32039	0.60745	1.17891	0.62916	2.20901
M27716.m	Abnormal spermatozoa	19	-0.051497246	0.20971	0.80602	0.94981	0.62969	1.43266
M27718.m	Abnormal spermatozoa	11	-0.005629309	0.58448	0.99232	0.99439	0.31626	3.1266
M27722.m	Abnormal spermatozoa	8	-0.729648287	0.76411	0.33963	0.48208	0.10782	2.15548
M27728.x	Abnormal spermatozoa	32	-0.120888361	0.52026	0.81626	0.88613	0.31963	2.45672
M27738.m	Abnormal spermatozoa	18	-0.04736485	0.37916	0.90059	0.95374	0.45361	2.00527
M30805.m	Abnormal spermatozoa	17	-1.161178456	0.6217	0.0618	0.31312	0.09258	1.05903
M31453.m	Abnormal spermatozoa	19	-1.963300152	0.66182	0.00301	0.14039	0.03837	0.51369
M31522.m	Abnormal spermatozoa	4	0.183471575	0.53484	0.73157	1.20138	0.42113	3.42728
M31536.m	Abnormal spermatozoa	28	-0.044489217	0.08369	0.59499	0.95649	0.81179	1.12698
M31536.x	Abnormal spermatozoa	23	0.013243289	0.13229	0.92026	0.98684	0.76145	1.27896
M31548.m	Abnormal spermatozoa	13	-0.770611772	0.48678	0.1134	0.46273	0.17823	1.20139

M31555.m	Abnormal spermatozoa	12	0.595653175	0.50923	0.24212	1.81422	0.66869	4.92216
M31591.m	Abnormal spermatozoa	30	0.079979345	0.1376	0.56108	1.08326	0.82719	1.41861
M31787.m	Abnormal spermatozoa	14	0.223242179	0.26377	0.39735	1.25012	0.74547	2.09642
M32197.m	Abnormal spermatozoa	23	0.526037654	1.1387	0.64411	1.69221	0.18162	15.7666
M32198.m	Abnormal spermatozoa	22	0.020382443	0.72663	0.97762	1.02059	0.24566	4.24009
M32315.m	Abnormal spermatozoa	38	0.564507967	0.77148	0.46434	1.75858	0.38768	7.97732
M32319.m	Abnormal spermatozoa	6	-0.550597069	0.78176	0.48124	0.57661	0.12458	2.66885
M32322.m	Abnormal spermatozoa	2	-1.811163553	1.918	0.34502	0.16346	0.00381	7.0155
M32328.m	Abnormal spermatozoa	25	0.283991604	0.42004	0.49897	1.32842	0.58317	3.02606
M32338.m	Abnormal spermatozoa	36	0.126359527	0.42716	0.76737	1.13469	0.49121	2.62111
M32339.m	Abnormal spermatozoa	41	1.261981579	0.83952	0.13278	3.53241	0.68149	18.3099
M32346.m	Abnormal spermatozoa	14	0.085465228	0.28669	0.76562	1.08922	0.62099	1.91051
M32348.m	Abnormal spermatozoa	48	-0.538865905	0.75457	0.47514	0.58341	0.13295	2.56019
M32379.m	Abnormal spermatozoa	13	-0.437047573	0.51311	0.39435	0.64594	0.23628	1.76588
M32388.m	Abnormal spermatozoa	7	1.870647698	0.67488	0.00557	6.4925	1.72957	24.3717
M32393.m	Abnormal spermatozoa	15	0.543952662	1.06976	0.61111	1.7228	0.21166	14.0227
M32405.m	Abnormal spermatozoa	18	0.32833104	0.49667	0.50857	1.38865	0.52458	3.67595
M32412.m	Abnormal spermatozoa	52	0.344305659	0.16159	0.03311	1.41101	1.02797	1.93678
M32418.m	Abnormal spermatozoa	16	-0.079988415	0.51593	0.87679	0.92313	0.33581	2.53763
M32425.m	Abnormal spermatozoa	23	0.359924397	0.38749	0.35296	1.43322	0.67062	3.06302
M32445.x	Abnormal spermatozoa	14	-0.065313645	0.43861	0.88162	0.93677	0.39654	2.21303
M32452.m	Abnormal spermatozoa	40	0.097377856	0.63991	0.87905	1.10228	0.31448	3.86359
M32489.m	Abnormal spermatozoa	37	0.051200264	1.01107	0.95961	1.05253	0.14508	7.63615
M32492.m	Abnormal spermatozoa	46	-1.226961582	0.61838	0.04724	0.29318	0.08725	0.98517
M32497.m	Abnormal spermatozoa	33	-0.058735632	0.35662	0.86918	0.94296	0.46874	1.89694
M32504.m	Abnormal spermatozoa	11	-0.17535624	0.64123	0.78449	0.83916	0.23879	2.94894
M32518.m	Abnormal spermatozoa	42	-1.365151692	0.92708	0.14088	0.25534	0.04149	1.57132
M32549.m	Abnormal spermatozoa	11	0.144406354	0.39784	0.71662	1.15535	0.52974	2.51979
M32553.m	Abnormal spermatozoa	17	0.418829863	0.46638	0.36916	1.52018	0.6094	3.79215
M32557.m	Abnormal spermatozoa	14	-0.047953241	0.60697	0.93703	0.95318	0.29008	3.1321
M32560.m	Abnormal spermatozoa	13	-0.018428834	0.25255	0.94183	0.98174	0.59844	1.61055
M32564.m	Abnormal spermatozoa	20	0.150739884	0.26796	0.57375	1.16269	0.68766	1.96589
M32578.m	Abnormal spermatozoa	20	-0.301472273	0.3956	0.44602	0.73973	0.34067	1.60624
M32586.m	Abnormal spermatozoa	18	0.209281943	0.28302	0.45963	1.23279	0.70791	2.14686
M32587.m	Abnormal spermatozoa	14	-1.22361613	0.71833	0.08849	0.29416	0.07197	1.20239
M32593.m	Abnormal spermatozoa	12	-0.457222178	0.54702	0.40325	0.63304	0.21667	1.84955
M32616.m	Abnormal spermatozoa	12	-0.253944196	0.3144	0.41925	0.77574	0.41888	1.4366
M32632.m	Abnormal spermatozoa	29	0.948377498	0.44155	0.03173	2.58152	1.08647	6.13383
M32634.m	Abnormal spermatozoa	45	-0.132332026	0.75592	0.86103	0.87605	0.1991	3.85464
M32635.m	Abnormal spermatozoa	15	0.343456947	0.82751	0.6781	1.40981	0.27847	7.13754
M32644.m	Abnormal spermatozoa	39	0.485439707	0.8624	0.57351	1.62489	0.29973	8.80875

M32651.m	Abnormal spermatozoa	26	1.023550189	0.66126	0.12165	2.78306	0.76146	10.1718
M32654.m	Abnormal spermatozoa	29	-0.120938878	0.69503	0.86186	0.88609	0.22691	3.4602
M32672.m	Abnormal spermatozoa	20	-0.424789505	0.43988	0.33419	0.65391	0.27611	1.54862
M32675.m	Abnormal spermatozoa	24	-0.779324954	1.40769	0.57984	0.45872	0.02906	7.24089
M32691.m	Abnormal spermatozoa	18	0.114321289	0.35264	0.7458	1.12111	0.56167	2.2378
M32698.m	Abnormal spermatozoa	18	-0.041549787	0.94811	0.96504	0.9593	0.14959	6.15177
M32709.m	Abnormal spermatozoa	32	-0.211795486	0.49112	0.66629	0.80913	0.30901	2.11869
M32729.m	Abnormal spermatozoa	48	0.105620339	0.77932	0.89219	1.1114	0.24127	5.11966
M32735.m	Abnormal spermatozoa	18	-0.179646446	0.34896	0.60669	0.83557	0.42164	1.65584
M32739.m	Abnormal spermatozoa	8	-1.697447233	1.2703	0.18147	0.18315	0.01519	2.20857
M32740.m	Abnormal spermatozoa	51	-0.616610454	0.79782	0.4396	0.53977	0.113	2.57825
M32753.m	Abnormal spermatozoa	13	-0.674537553	0.43583	0.12169	0.50939	0.2168	1.19684
M32754.m	Abnormal spermatozoa	15	0.044608022	0.21306	0.83416	1.04562	0.68867	1.58758
M32755.m	Abnormal spermatozoa	24	0.721056968	0.45553	0.11345	2.05661	0.84216	5.02237
M32757.m	Abnormal spermatozoa	21	0.074219764	0.2801	0.79103	1.07704	0.62202	1.86493
M32758.m	Abnormal spermatozoa	22	0.088977385	0.33179	0.78857	1.09306	0.57045	2.09445
M32759.m	Abnormal spermatozoa	25	0.193351166	0.28842	0.50261	1.21331	0.68939	2.13538

M32761.m	Abnormal spermatozoa	19	0.380730531	0.56142	0.49767	1.46335	0.48692	4.39787
M32762.m	Abnormal spermatozoa	15	0.190261179	0.28173	0.49947	1.20957	0.69633	2.10109
M32769.m	Abnormal spermatozoa	5	-0.640881354	0.65013	0.32424	0.52683	0.14732	1.88394
M32786.m	Abnormal spermatozoa	19	0.233446706	0.33075	0.48031	1.26295	0.66046	2.41505
M32787.m	Abnormal spermatozoa	19	0.848647131	0.50928	0.09564	2.33648	0.86109	6.33979
M32795.m	Abnormal spermatozoa	14	0.501758643	0.37921	0.18578	1.65162	0.78546	3.47295
M32800.m	Abnormal spermatozoa	12	-0.480500368	0.23249	0.03876	0.61847	0.39212	0.97548
M32802.m	Abnormal spermatozoa	7	-0.254535352	0.65866	0.69917	0.77528	0.2132	2.81916
M32808.m	Abnormal spermatozoa	17	0.182445711	0.30673	0.55197	1.20015	0.65787	2.18942
M32814.m	Abnormal spermatozoa	14	0.340435453	1.11616	0.76036	1.40556	0.15767	12.5298
M32836.m	Abnormal spermatozoa	7	-0.502383417	0.58298	0.38882	0.60509	0.19301	1.89696
M32838.m	Abnormal spermatozoa	11	0.023039035	0.70396	0.97389	1.02331	0.2575	4.06661
M32846.m	Abnormal spermatozoa	25	0.22762978	0.14323	0.11199	1.25562	0.94829	1.66255
M32847.m	Abnormal spermatozoa	21	0.347121282	0.3552	0.32844	1.41499	0.70535	2.83859
M32854.m	Abnormal spermatozoa	7	0.410541506	0.37595	0.27483	1.50763	0.72157	3.15001
M32855.m	Abnormal spermatozoa	23	0.454243605	0.29401	0.12235	1.57498	0.88513	2.8025
M32857.m	Abnormal spermatozoa	8	0.109719673	0.52656	0.83494	1.11597	0.39759	3.13232
M32863.m	Abnormal spermatozoa	15	-0.119034088	0.18504	0.52004	0.88778	0.61773	1.27589
M32867.m	Abnormal spermatozoa	21	-1.518113334	1.70601	0.37354	0.21912	0.00774	6.20691
M32869.m	Abnormal spermatozoa	17	0.288129511	0.49976	0.56425	1.33393	0.50088	3.55251
M32910.m	Abnormal spermatozoa	61	-0.4412727	0.51462	0.39119	0.64322	0.23459	1.76365
M32980.m	Abnormal spermatozoa	12	-1.006326643	0.74681	0.17782	0.36556	0.08458	1.57998
M33009.m	Abnormal spermatozoa	6	1.192382079	0.66945	0.07489	3.29492	0.88715	12.2374
M33009.x	Abnormal spermatozoa	20	0.512054265	0.4557	0.26116	1.66872	0.6831	4.07646
M33084.m	Abnormal spermatozoa	11	-0.204158367	0.41457	0.62239	0.81533	0.36178	1.83748
M33131.m	Abnormal spermatozoa	13	1.538586532	0.60706	0.01126	4.658	1.41728	15.3089
M33132.m	Abnormal spermatozoa	39	-0.493006156	0.94731	0.60277	0.61079	0.09539	3.91073
M33137.m	Abnormal spermatozoa	15	0.005265248	0.2227	0.98114	1.00528	0.64972	1.55543
M33138.m	Abnormal spermatozoa	26	0.052250746	0.34724	0.88039	1.05364	0.53348	2.08097
M33140.m	Abnormal spermatozoa	12	0.472491757	0.80678	0.55811	1.60399	0.32995	7.79737
M33144.m	Abnormal spermatozoa	9	-0.003845002	0.29338	0.98954	0.99616	0.56053	1.77034
M33150.m	Abnormal spermatozoa	11	0.075761056	0.23915	0.7514	1.0787	0.67505	1.72373
M33163.m	Abnormal spermatozoa	19	0.384775283	1.02962	0.70862	1.46928	0.19529	11.0544
M33165.m	Abnormal spermatozoa	14	-0.375539312	0.57691	0.51508	0.68692	0.22173	2.12803
M33173.x	Abnormal spermatozoa	49	0.003264055	0.02248	0.88458	1.00327	0.96002	1.04847
M33178.x	Abnormal spermatozoa	500	-0.024556937	0.01795	0.17123	0.97574	0.94201	1.01068
M33188.m	Abnormal spermatozoa	3	0.213511591	0.80566	0.791	1.23802	0.25523	6.00509
M33190.m	Abnormal spermatozoa	21	0.153364485	0.20541	0.4553	1.16575	0.77939	1.74364
M33192.m	Abnormal spermatozoa	13	0.269238206	0.25396	0.28907	1.30897	0.79571	2.15328
M33194.m	Abnormal spermatozoa	13	-0.087569224	0.24641	0.7223	0.91616	0.56523	1.48496
M33195.m	Abnormal spermatozoa	7	0.333793825	0.31977	0.29655	1.39626	0.74606	2.6131

M33197.m	Abnormal spermatozoa	9	0.147255078	0.23001	0.52203	1.15865	0.73819	1.81861
M33203.m	Abnormal spermatozoa	17	-0.269892033	0.15375	0.07919	0.76346	0.56482	1.03196
M33204.m	Abnormal spermatozoa	13	-1.600811809	0.85428	0.06095	0.20173	0.03781	1.07635
M33221.m	Abnormal spermatozoa	18	0.45769161	0.48418	0.34451	1.58042	0.61183	4.08239
M33228.m	Abnormal spermatozoa	24	-0.459586759	0.56185	0.41336	0.63154	0.20997	1.89959
M33230.m	Abnormal spermatozoa	14	1.421251768	1.12116	0.20492	4.1423	0.46014	37.29
M33250.m	Abnormal spermatozoa	23	0.143800604	0.28623	0.61539	1.15465	0.65888	2.02346
M33353.m	Abnormal spermatozoa	21	-0.170677571	0.17397	0.32657	0.84309	0.5995	1.18567
M33359.m	Abnormal spermatozoa	9	0.21976742	0.37931	0.56233	1.24579	0.59234	2.62011
M33363.m	Abnormal spermatozoa	9	-0.496597493	0.72568	0.49377	0.6086	0.14676	2.52373
M33364.m	Abnormal spermatozoa	10	0.214114342	0.87212	0.80606	1.23876	0.22419	6.84471
M33380.m	Abnormal spermatozoa	42	1.350662145	0.76392	0.07705	3.85998	0.86362	17.2524
M33384.x	Abnormal spermatozoa	12	0.170837462	0.11116	0.12434	1.1863	0.95405	1.47509
M33389.m	Abnormal spermatozoa	57	0.168812095	0.74316	0.8203	1.1839	0.27588	5.08049
M33390.m	Abnormal spermatozoa	15	-0.086633554	0.31625	0.78413	0.91701	0.49337	1.70442
M33391.m	Abnormal spermatozoa	16	0.185049189	0.09526	0.05208	1.20328	0.99833	1.45029
M33408.m	Abnormal spermatozoa	14	-0.220513654	0.3397	0.51625	0.80211	0.41217	1.56096

M33415.m	Abnormal spermatozoa	25	0.112757331	0.23868	0.63662	1.11936	0.70114	1.78704
M33420.m	Abnormal spermatozoa	13	0.363650334	0.40368	0.36767	1.43857	0.6521	3.17358
M33422.m	Abnormal spermatozoa	38	0.545383846	0.97839	0.57723	1.72527	0.25353	11.7404
M33423.x	Abnormal spermatozoa	58	-0.019843418	0.01344	0.13987	0.98035	0.95486	1.00652
M33441.m	Abnormal spermatozoa	17	-0.008281059	0.42567	0.98448	0.99175	0.43059	2.28425
M33442.m	Abnormal spermatozoa	28	0.594358267	1.45165	0.68222	1.81187	0.10531	31.1745
M33443.m	Abnormal spermatozoa	10	0.346850643	1.02935	0.73615	1.41461	0.18812	10.6374
M33447.m	Abnormal spermatozoa	11	0.273567846	0.78436	0.72726	1.31465	0.28258	6.11603
M33453.m	Abnormal spermatozoa	17	0.092859299	0.61325	0.87964	1.09731	0.32986	3.65034
M33477.m	Abnormal spermatozoa	50	-0.35629012	0.79289	0.65318	0.70027	0.14803	3.31277
M33488.m	Abnormal spermatozoa	18	0.103343118	0.37772	0.78439	1.10887	0.52889	2.32488
M33507.m	Abnormal spermatozoa	40	-0.11032494	0.12674	0.38404	0.89554	0.69856	1.14807
M33508.m	Abnormal spermatozoa	20	-0.353567638	0.24093	0.14224	0.70218	0.43789	1.12599
M33509.m	Abnormal spermatozoa	9	1.063331512	0.8186	0.19396	2.896	0.58209	14.4081
M33510.m	Abnormal spermatozoa	26	-0.493741508	0.68969	0.47406	0.61034	0.15794	2.35855
M33515.m	Abnormal spermatozoa	44	-0.261010174	0.8552	0.76021	0.77027	0.14411	4.11723
M33531.m	Abnormal spermatozoa	10	0.525896523	0.49137	0.2845	1.69198	0.64585	4.43254
M33587.m	Abnormal spermatozoa	12	0.031522313	0.82929	0.96968	1.03202	0.20313	5.24319
M33609.m	Abnormal spermatozoa	20	0.050849856	0.12868	0.69271	1.05216	0.81762	1.35399
M33610.m	Abnormal spermatozoa	29	-0.001333623	0.08542	0.98754	0.99867	0.84472	1.18068
M33627.m	Abnormal spermatozoa	10	1.16599785	0.85491	0.1726	3.20912	0.60072	17.1435
M33633.m	Abnormal spermatozoa	11	-0.238866623	0.45231	0.59743	0.78752	0.32453	1.91106
M33637.m	Abnormal spermatozoa	10	0.305248914	0.51677	0.55473	1.35696	0.49282	3.73637
M33638.m	Abnormal spermatozoa	10	0.357188867	0.60993	0.55813	1.42931	0.43246	4.72399
M33652.m	Abnormal spermatozoa	9	0.739733145	0.45087	0.10086	2.09538	0.86591	5.0705
M33653.m	Abnormal spermatozoa	13	0.180500236	0.51179	0.72433	1.19782	0.43928	3.26614
M33658.m	Abnormal spermatozoa	9	-0.474981312	0.40778	0.2441	0.6219	0.27965	1.38301
M33666.m	Abnormal spermatozoa	29	0.216029814	0.6635	0.74473	1.24114	0.33809	4.55622
M33675.m	Abnormal spermatozoa	8	0.253259932	0.54225	0.64046	1.28822	0.44506	3.72873
M33683.m	Abnormal spermatozoa	12	0.072143021	0.16687	0.66551	1.07481	0.77497	1.49066
M33751.m	Abnormal spermatozoa	28	-0.045620969	0.07598	0.54822	0.9554	0.82321	1.10883
M33782.m	Abnormal spermatozoa	14	0.088694863	0.29512	0.76376	1.09275	0.61279	1.94863
M33801.m	Abnormal spermatozoa	9	0.32216714	0.45791	0.48171	1.38012	0.56252	3.38607
M33821.m	Abnormal spermatozoa	21	0.497543938	0.58887	0.39816	1.64468	0.51859	5.216
M33822.m	Abnormal spermatozoa	8	0.278496988	0.94071	0.76719	1.32114	0.20903	8.35028
M33833.m	Abnormal spermatozoa	8	-0.276605308	0.61418	0.65244	0.75835	0.22755	2.52737
M33835.m	Abnormal spermatozoa	9	0.205156142	0.30571	0.50217	1.22772	0.67433	2.23523
M33864.m	Abnormal spermatozoa	9	-0.121225622	0.36698	0.74115	0.88583	0.43149	1.81857
M33871.m	Abnormal spermatozoa	13	-0.517096611	0.66087	0.43395	0.59625	0.16326	2.17759
M33877.m	Abnormal spermatozoa	30	0.030802119	0.0815	0.70549	1.03128	0.87902	1.20991
M33883.m	Abnormal spermatozoa	15	0.008372345	0.2147	0.96889	1.00841	0.66203	1.53601

M33884.m	Abnormal spermatozoa	13	-0.823644139	0.46391	0.07583	0.43883	0.17677	1.0894
M33885.m	Abnormal spermatozoa	12	0.251479312	0.28482	0.37726	1.28593	0.73583	2.24728
M33892.m	Abnormal spermatozoa	31	-0.176641445	0.51011	0.72913	0.83808	0.30837	2.2777
M33901.m	Abnormal spermatozoa	22	0.173842468	0.35929	0.62849	1.18987	0.58839	2.40619
M33910.m	Abnormal spermatozoa	14	-0.857424411	0.497	0.08449	0.42425	0.16017	1.12378
M33935.x	Abnormal spermatozoa	11	0.282058911	0.46617	0.54515	1.32586	0.53172	3.30607
M33936.m	Abnormal spermatozoa	23	0.134526534	0.39148	0.73112	1.144	0.53112	2.4641
M33937.m	Abnormal spermatozoa	16	-0.445954173	0.5529	0.41992	0.64021	0.21661	1.89219
M33939.m	Abnormal spermatozoa	13	0.54585255	0.98866	0.58087	1.72608	0.2486	11.9846
M33941.m	Abnormal spermatozoa	21	0.42602935	0.40391	0.29153	1.53117	0.69376	3.37938
M33955.m	Abnormal spermatozoa	34	-0.828142458	1.02486	0.41906	0.43686	0.05861	3.25624
M33957.m	Abnormal spermatozoa	10	0.404268878	0.82257	0.62309	1.49821	0.2988	7.51207
M33960.m	Abnormal spermatozoa	17	0.870163173	0.82563	0.29191	2.3873	0.47328	12.0419
M33961.m	Abnormal spermatozoa	13	-0.010662352	0.86209	0.99013	0.98939	0.18262	5.36034
M33968.m	Abnormal spermatozoa	19	0.497760934	0.47983	0.29956	1.64503	0.6423	4.21319
M33969.m	Abnormal spermatozoa	11	0.293424836	0.62105	0.63659	0.74571	0.22076	2.51891
M33971.m	Abnormal spermatozoa	6	-0.795221144	1.53138	0.60356	0.45148	0.02244	9.08185

M33972.m	Abnormal spermatozoa	7	-0.740857865	1.17421	0.52808	0.4767	0.04772	4.76164
M33973.m	Abnormal spermatozoa	17	0.220346769	0.20773	0.28881	1.24651	0.82961	1.87291
M34035.m	Abnormal spermatozoa	6	0.906770296	1.03495	0.38095	2.47631	0.32571	18.8267
M34040.m	Abnormal spermatozoa	20	0.42381585	0.42018	0.31314	1.52778	0.6705	3.48115
M34062.m	Abnormal spermatozoa	31	-0.797878549	1.1407	0.48426	0.45028	0.04814	4.2118
M34106.m	Abnormal spermatozoa	19	-0.058332606	0.39138	0.88152	0.94334	0.43804	2.03151
M34109.x	Abnormal spermatozoa	34	-0.014900223	0.03668	0.68457	0.98521	0.91687	1.05865
M34112.m	Abnormal spermatozoa	18	-0.360547205	0.36469	0.32284	0.69729	0.34118	1.4251
M34123.m	Abnormal spermatozoa	23	-0.099438412	0.67136	0.88225	0.90535	0.24285	3.37509
M34214.m	Abnormal spermatozoa	21	0.088163416	0.71025	0.90121	1.09217	0.27147	4.39404
M34221.m	Abnormal spermatozoa	12	-0.420302131	0.74912	0.57476	0.65685	0.15129	2.85187
M34244.m	Abnormal spermatozoa	24	-0.552591288	0.55984	0.32362	0.57546	0.19207	1.72408
M34245.m	Abnormal spermatozoa	19	0.269232883	0.49602	0.58727	1.30896	0.49512	3.46053
M34283.m	Abnormal spermatozoa	44	0.854820932	0.66695	0.19995	2.35095	0.6361	8.6888
M34289.m	Abnormal spermatozoa	15	-9.48783E-05	0.54027	0.99986	0.99991	0.34679	2.88302
M34306.m	Abnormal spermatozoa	27	0.526769108	0.31839	0.09803	1.69345	0.90731	3.16073
M34314.m	Abnormal spermatozoa	10	0.222910758	0.41832	0.59412	1.24971	0.55046	2.83721
M34322.m	Abnormal spermatozoa	40	0.05867258	0.09622	0.54202	1.06043	0.87816	1.28052
M34327.m	Abnormal spermatozoa	9	-0.427542843	0.33874	0.20689	0.65211	0.33572	1.26667
M34329.m	Abnormal spermatozoa	12	0.220180128	0.31833	0.48914	1.2463	0.66781	2.3259
M34336.m	Abnormal spermatozoa	21	0.040564774	0.26015	0.87609	1.0414	0.62543	1.73404
M34338.m	Abnormal spermatozoa	43	-0.008490647	0.03404	0.80305	0.99155	0.92754	1.05996
M34339.m	Abnormal spermatozoa	18	0.111777313	0.1796	0.53371	1.11826	0.78644	1.5901
M34344.m	Abnormal spermatozoa	12	-0.208164883	0.21858	0.34093	0.81207	0.52909	1.2464
M34350.m	Abnormal spermatozoa	14	0.050392451	0.23225	0.82823	1.05168	0.6671	1.65797
M34359.m	Abnormal spermatozoa	45	-0.561963837	0.70945	0.4283	0.57009	0.14192	2.29003
M34365.x	Abnormal spermatozoa	68	-0.017320609	0.03924	0.65894	0.98283	0.91007	1.0614
M34384.m	Abnormal spermatozoa	7	0.216723349	0.29015	0.45509	1.242	0.70331	2.1933
M34384.x	Abnormal spermatozoa	6	0.617007206	0.61456	0.31539	1.85337	0.5557	6.18142
M34389.x	Abnormal spermatozoa	16	-0.152999321	0.41678	0.71355	0.85813	0.37912	1.94235
M34390.x	Abnormal spermatozoa	10	0.012257475	0.45953	0.97872	1.01233	0.41131	2.49162
M34395.x	Abnormal spermatozoa	18	-0.456758504	0.44481	0.30448	0.63333	0.26485	1.51446
M34400.x	Abnormal spermatozoa	12	-0.175360272	0.4352	0.68699	0.83915	0.3576	1.96919
M34404.x	Abnormal spermatozoa	10	-0.528463548	0.21491	0.01393	0.58951	0.38686	0.89832
M34407.m	Abnormal spermatozoa	26	-0.423250011	0.43766	0.3335	0.65491	0.27774	1.54427
M34409.m	Abnormal spermatozoa	6	-0.61827114	1.18655	0.60232	0.53888	0.05266	5.51438
M34416.m	Abnormal spermatozoa	14	0.87808403	0.61758	0.15508	2.40628	0.71722	8.07317
M34419.m	Abnormal spermatozoa	15	1.916719702	1.11474	0.08554	6.79862	0.76478	60.4377
M34420.m	Abnormal spermatozoa	27	0.01311919	0.14763	0.92919	1.01321	0.75863	1.35321
M34441.m	Abnormal spermatozoa	16	0.395955167	0.42937	0.35643	1.4858	0.64044	3.44705
M34453.m	Abnormal spermatozoa	3	0.147032296	3.27571	0.9642	1.15839	0.00189	711.54

M34456.m	Abnormal spermatozoa	26	-0.360061047	0.64544	0.57695	0.69763	0.19689	2.47195
M34469.m	Abnormal spermatozoa	11	-0.059051621	0.54273	0.91336	0.94266	0.32537	2.73109
M34481.m	Abnormal spermatozoa	26	0.138274982	0.3403	0.6845	1.14829	0.58937	2.23727
M34499.m	Abnormal spermatozoa	7	-0.391230268	0.44969	0.3843	0.67622	0.28009	1.63259
M34513.m	Abnormal spermatozoa	13	0.272934554	0.36198	0.45084	1.31381	0.64627	2.67088
M34516.m	Abnormal spermatozoa	18	-0.059296349	0.09899	0.54918	0.94243	0.77622	1.14423
M34527.m	Abnormal spermatozoa	17	-1.527836632	0.68202	0.02508	0.217	0.05701	0.82607
M34530.m	Abnormal spermatozoa	10	0.07162937	0.33539	0.83088	1.07426	0.5567	2.07298
M34533.m	Abnormal spermatozoa	16	-0.181980733	0.47724	0.70297	0.83362	0.32714	2.12422
M34534.m	Abnormal spermatozoa	13	-0.468129774	0.57719	0.41733	0.62617	0.20201	1.94091
M34535.m	Abnormal spermatozoa	7	0.061766376	0.09997	0.53668	1.06371	0.87443	1.29396
M34539.m	Abnormal spermatozoa	20	0.04012295	0.37863	0.91561	1.04094	0.49559	2.18637
M34674.m	Abnormal spermatozoa	13	-0.304366045	0.49057	0.53497	0.73759	0.28199	1.92929
M34732.m	Abnormal spermatozoa	4	0.781412717	1.54909	0.61396	2.18456	0.10489	45.4964
M34761.m	Abnormal spermatozoa	19	-0.72551395	0.56748	0.20108	0.48408	0.15917	1.47217
M34878.m	Abnormal spermatozoa	11	-0.372686491	0.41556	0.36981	0.68888	0.30508	1.55552
M34912.m	Abnormal spermatozoa	46	-0.581811508	0.65955	0.3777	0.55889	0.15343	2.03582

M35114.m	Abnormal spermatozoa	10	-0.524696721	0.72287	0.46793	0.59173	0.14349	2.44031
M35126.m	Abnormal spermatozoa	17	0.226255034	0.37804	0.54951	1.2539	0.59768	2.63059
M35127.m	Abnormal spermatozoa	20	0.158266445	0.8036	0.84387	1.17148	0.24249	5.65944
M35137.m	Abnormal spermatozoa	58	0.400029865	0.53945	0.45836	1.49187	0.51825	4.2946
M35159.m	Abnormal spermatozoa	9	-0.771495367	0.47847	0.10687	0.46232	0.18099	1.18094
M35160.m	Abnormal spermatozoa	11	-0.684894876	1.10324	0.53473	0.50414	0.058	4.3818
M35186.m	Abnormal spermatozoa	28	-0.867480341	0.69432	0.21152	0.42001	0.10771	1.63784
M35187.m	Abnormal spermatozoa	12	0.197697683	0.2497	0.42851	1.21859	0.74698	1.98796
M35189.m	Abnormal spermatozoa	22	-0.013984247	0.29138	0.96172	0.98611	0.55706	1.74564
M35193.m	Abnormal spermatozoa	27	-0.561114137	0.60125	0.35069	0.57057	0.1756	1.85397
M35240.m	Abnormal spermatozoa	7	0.73144944	1.39865	0.601	2.07809	0.134	32.2271
M35253.m	Abnormal spermatozoa	24	0.46320704	0.69128	0.50281	1.58916	0.40996	6.16023
M35254.m	Abnormal spermatozoa	17	0.621097215	1.17027	0.59561	1.86097	0.18775	18.4456
M35255.m	Abnormal spermatozoa	13	-0.755700888	0.67076	0.2599	0.46968	0.12614	1.7489
M35257.m	Abnormal spermatozoa	22	2.051081336	0.6872	0.00284	7.77631	2.02216	29.9041
M35270.m	Abnormal spermatozoa	42	-1.194778717	0.93243	0.20007	0.30277	0.04869	1.88286
M35305.m	Abnormal spermatozoa	11	-0.021154445	0.634	0.97338	0.97907	0.28258	3.3922
M35320.x	Abnormal spermatozoa	12	0.455771215	0.53181	0.39143	1.57739	0.55623	4.47326
M35322.x	Abnormal spermatozoa	17	0.074455603	0.17876	0.67703	1.0773	0.75888	1.52931
M35326.m	Abnormal spermatozoa	43	1.020496732	1.03574	0.32448	2.77457	0.36438	21.1267
M35327.m	Abnormal spermatozoa	20	0.388910487	1.41123	0.78287	1.47537	0.09282	23.4513
M35331.m	Abnormal spermatozoa	8	-0.94550522	0.90298	0.29506	0.38848	0.06618	2.28038
M35397.m	Abnormal spermatozoa	32	-0.068672419	1.07667	0.94914	0.93363	0.11316	7.70299
M35431.m	Abnormal spermatozoa	26	-0.526900391	1.08211	0.62632	0.59043	0.0708	4.92358
M35433.m	Abnormal spermatozoa	8	-0.223491436	0.70355	0.75074	0.79972	0.2014	3.17548
M35439.m	Abnormal spermatozoa	42	-0.071159968	0.46503	0.87838	0.93131	0.37433	2.31708
M35451.m	Abnormal spermatozoa	18	0.015204346	0.10354	0.88326	1.01532	0.82883	1.24378
M35464.m	Abnormal spermatozoa	17	-1.334418255	1.01221	0.18739	0.26331	0.03621	1.9146
M35472.m	Abnormal spermatozoa	18	0.291143375	0.47707	0.54168	1.33796	0.52523	3.40828
M35527.m	Abnormal spermatozoa	8	0.328750902	0.48612	0.49887	1.38923	0.53577	3.60222
M35527.x	Abnormal spermatozoa	11	-0.543082884	0.67455	0.42076	0.58095	0.15487	2.17936
M35551.m	Abnormal spermatozoa	12	0.730274187	0.3872	0.05929	2.07565	0.97177	4.43347
M35626.m	Abnormal spermatozoa	6	-0.409683507	0.80462	0.61064	0.66386	0.13714	3.21354
M35628.m	Abnormal spermatozoa	10	-0.104564418	0.8971	0.90721	0.90072	0.15522	5.22659
M35631.m	Abnormal spermatozoa	28	0.697611751	0.53371	0.19118	2.00895	0.70577	5.71838
M35635.m	Abnormal spermatozoa	10	0.080265195	0.36246	0.82474	1.08357	0.53251	2.2049
M35669.m	Abnormal spermatozoa	25	0.301719331	0.28741	0.29382	1.35218	0.76981	2.37512
M35675.m	Abnormal spermatozoa	48	0.24545757	0.67386	0.71567	1.27821	0.3412	4.78849
M35678.m	Abnormal spermatozoa	27	0.159527722	0.27902	0.5675	1.17296	0.67885	2.02671
M35718.m	Abnormal spermatozoa	25	0.806759205	0.91665	0.3788	2.24063	0.37162	13.5095
M35754.m	Abnormal spermatozoa	44	0.182688001	0.61077	0.76486	1.20044	0.36261	3.97411

M35854.m	Abnormal spermatozoa	13	-0.252054393	0.62117	0.68491	0.7772	0.23003	2.62593
M35977.m	Abnormal spermatozoa	10	-0.176917411	0.70257	0.80118	0.83785	0.21141	3.3205
M35978.m	Abnormal spermatozoa	18	-0.316276708	0.46776	0.49895	0.72886	0.29139	1.8231
M36009.m	Abnormal spermatozoa	14	0.165698466	0.47283	0.72601	1.18022	0.46718	2.98155
M36095.x	Abnormal spermatozoa	5	-0.241582562	0.31723	0.44633	0.78538	0.42175	1.46256
M36097.x	Abnormal spermatozoa	30	0.005589692	0.0307	0.85552	1.00561	0.94688	1.06797
M36098.x	Abnormal spermatozoa	11	-0.13299453	0.3485	0.70274	0.87547	0.44218	1.73335
M36099.x	Abnormal spermatozoa	7	-0.584265749	0.43167	0.17589	0.55752	0.23923	1.29927
M36103.m	Abnormal spermatozoa	15	0.319774413	0.35025	0.36125	1.37682	0.69301	2.73537
M36115.m	Abnormal spermatozoa	13	0.229609513	0.26774	0.39112	1.25811	0.74441	2.1263
M36131.m	Abnormal spermatozoa	17	-0.471998691	0.39274	0.22943	0.62375	0.28887	1.34685
M36134.m	Abnormal spermatozoa	14	0.067016231	0.28306	0.81285	1.06931	0.61398	1.86231
M36230.m	Abnormal spermatozoa	21	0.311726201	0.39414	0.429	1.36578	0.63079	2.95717
M36300.m	Abnormal spermatozoa	9	-0.671768697	0.89388	0.45234	0.5108	0.08859	2.94538
M36376.m	Abnormal spermatozoa	5	-0.569520256	0.60356	0.34537	0.5658	0.17334	1.8468
M36399.m	Abnormal spermatozoa	13	0.395033267	0.66918	0.55497	1.48443	0.39989	5.51034
M36468.m	Abnormal spermatozoa	16	0.735346193	0.38424	0.05565	2.0862	0.98239	4.43024

M36515.m	Abnormal spermatozoa	10	-0.749395716	3.17825	0.8136	0.47265	0.00093	239.842
M36552.m	Abnormal spermatozoa	20	0.441871566	1.10411	0.689	1.55562	0.17868	13.5437
M36553.m	Abnormal spermatozoa	22	-0.232273095	0.63207	0.71326	0.79273	0.22967	2.73625
M36559.m	Abnormal spermatozoa	19	0.089678101	0.36721	0.80707	1.09382	0.53256	2.2466
M36585.m	Abnormal spermatozoa	11	-0.058991856	0.45132	0.89601	0.94271	0.38923	2.28324
M36589.m	Abnormal spermatozoa	19	0.247929211	0.24358	0.30874	1.28137	0.79494	2.06544
M36673.m	Abnormal spermatozoa	12	1.91826587	0.88179	0.0296	6.80914	1.20921	38.3428
M36738.m	Abnormal spermatozoa	10	0.337878801	0.40562	0.40485	1.40197	0.6331	3.10461
M36754.m	Abnormal spermatozoa	12	0.579947099	0.71396	0.41662	1.78594	0.44069	7.23774
M36756.m	Abnormal spermatozoa	11	-0.861149582	0.59564	0.14824	0.42268	0.13152	1.35839
M36776.m	Abnormal spermatozoa	16	0.662529199	1.09131	0.54379	1.93969	0.22845	16.4694
M36802.m	Abnormal spermatozoa	10	0.366878243	0.90938	0.68663	1.44322	0.2428	8.57852
M36808.m	Abnormal spermatozoa	34	0.316673179	1.30383	0.8081	1.37255	0.10658	17.6756
M36850.m	Abnormal spermatozoa	12	0.303173405	0.44507	0.49576	1.35415	0.566	3.23979
M37004.m	Abnormal spermatozoa	9	0.02713491	0.72719	0.97023	1.02751	0.24705	4.27347
M37058.m	Abnormal spermatozoa	47	0.701641796	0.69854	0.31517	2.01706	0.51299	7.93106
M37097.m	Abnormal spermatozoa	15	-0.239761664	0.21789	0.27117	0.78682	0.51333	1.206
M37104.m	Abnormal spermatozoa	15	0.536519723	0.36139	0.13765	1.71005	0.84215	3.47238
M37112.m	Abnormal spermatozoa	13	-0.042875662	0.14316	0.76456	0.95803	0.72364	1.26834
M37190.m	Abnormal spermatozoa	21	0.297282457	0.21172	0.16027	1.3462	0.88898	2.03857
M37202.m	Abnormal spermatozoa	27	0.089699016	0.20349	0.65935	1.09385	0.73408	1.62992
M37203.m	Abnormal spermatozoa	20	0.357944716	0.52713	0.49711	1.43039	0.50904	4.01933
M37253.m	Abnormal spermatozoa	13	0.441032652	0.68877	0.52196	1.55431	0.40294	5.99557
M37459.m	Abnormal spermatozoa	3	0.166656309	0.62971	0.79128	1.18135	0.34384	4.05883
M37459.x	Abnormal spermatozoa	10	-0.431284452	0.49193	0.38064	0.64967	0.24772	1.70386
M37506.m	Abnormal spermatozoa	48	0.752139252	0.83497	0.36769	2.12153	0.41296	10.899
M38150.m	Abnormal spermatozoa	4	-0.051309472	1.37994	0.97034	0.94998	0.06355	14.2018
M38178.m	Abnormal spermatozoa	15	0.399839442	0.52882	0.44959	1.49159	0.52906	4.20521
M38768.m	Abnormal spermatozoa	15	0.721498077	0.88162	0.41314	2.05751	0.36551	11.5822

Supplementary Table 9: The causal effect of serum inflammatory factors on abnormal spermatozoa.

Exposure	Outcome	nsnpb		se	pval	or	or_lci95	or_uci95
ebi-a-GCST004420	Abnormal spermatozoa	9	0.012276816	0.12979	0.92464	1.01235	0.78496	1.30561
ebi-a-GCST004421	Abnormal spermatozoa	7	-0.057423239	0.18039	0.75024	0.94419	0.66299	1.34467
ebi-a-GCST004422	Abnormal spermatozoa	10	0.069555967	0.08428	0.4092	1.07203	0.9088	1.26458
ebi-a-GCST004423	Abnormal spermatozoa	6	-0.319567729	0.16044	0.04639	0.72646	0.53045	0.99491
ebi-a-GCST004424	Abnormal spermatozoa	15	0.156770534	0.08297	0.05883	1.16973	0.99416	1.37629
ebi-a-GCST004425	Abnormal spermatozoa	4	0.282263265	0.21124	0.18147	1.32613	0.87655	2.0063
ebi-a-GCST004426	Abnormal spermatozoa	5	0.256638906	0.17104	0.13349	1.29258	0.92442	1.80737
ebi-a-GCST004428	Abnormal spermatozoa	14	-0.134024985	0.09237	0.14678	0.87457	0.72974	1.04814
ebi-a-GCST004429	Abnormal spermatozoa	9	-0.254283038	0.18704	0.17399	0.77547	0.53747	1.11887
ebi-a-GCST004430	Abnormal spermatozoa	10	0.002968788	0.09638	0.97543	1.00297	0.83032	1.21153
ebi-a-GCST004431	Abnormal spermatozoa	9	-0.061141161	0.13262	0.64478	0.94069	0.72537	1.21993
ebi-a-GCST004432	Abnormal spermatozoa	13	-0.033377037	0.11608	0.77371	0.96717	0.77036	1.21427
ebi-a-GCST004433	Abnormal spermatozoa	17	0.0865579	0.09427	0.35854	1.09041	0.90645	1.31171
ebi-a-GCST004435	Abnormal spermatozoa	14	0.033735348	0.14173	0.81186	1.03431	0.78344	1.36551
ebi-a-GCST004436	Abnormal spermatozoa	8	-0.021727037	0.16499	0.89523	0.97851	0.70814	1.3521
ebi-a-GCST004437	Abnormal spermatozoa	3	0.113254373	0.14044	0.42001	1.11992	0.85043	1.4748
ebi-a-GCST004438	Abnormal spermatozoa	14	-0.045724233	0.16361	0.77989	0.95531	0.69322	1.31647
ebi-a-GCST004439	Abnormal spermatozoa	10	0.067935394	0.10626	0.52263	1.0703	0.86906	1.31813
ebi-a-GCST004440	Abnormal spermatozoa	10	0.079480991	0.13477	0.55537	1.08272	0.83137	1.41007
ebi-a-GCST004441	Abnormal spermatozoa	15	0.108883216	0.08396	0.19468	1.11503	0.94585	1.31448
ebi-a-GCST004442	Abnormal spermatozoa	10	0.003220728	0.19449	0.98679	1.00323	0.68525	1.46876
ebi-a-GCST004443	Abnormal spermatozoa	9	-0.042713197	0.08945	0.63299	0.95819	0.8041	1.1418
ebi-a-GCST004444	Abnormal spermatozoa	10	0.073147551	0.12652	0.56315	1.07589	0.83961	1.37867
ebi-a-GCST004445	Abnormal spermatozoa	4	-0.184218892	0.20339	0.36506	0.83175	0.5583	1.23914
ebi-a-GCST004446	Abnormal spermatozoa	5	-0.209621461	0.24555	0.39328	0.81089	0.50113	1.31213
ebi-a-GCST004447	Abnormal spermatozoa	6	0.06571572	0.17004	0.69914	1.06792	0.76525	1.49032
ebi-a-GCST004448	Abnormal spermatozoa	5	0.113402892	0.20012	0.57093	1.12008	0.75667	1.65803
ebi-a-GCST004449	Abnormal spermatozoa	7	0.177323641	0.20149	0.37884	1.19402	0.80444	1.77226
ebi-a-GCST004450	Abnormal spermatozoa	6	-0.139262146	0.23705	0.55688	0.87	0.54669	1.38452
ebi-a-GCST004451	Abnormal spermatozoa	9	-0.082809671	0.12004	0.49028	0.92053	0.72754	1.1647
ebi-a-GCST004452	Abnormal spermatozoa	5	-0.055327802	0.16822	0.74223	0.94617	0.68042	1.31572
ebi-a-GCST004453	Abnormal spermatozoa	9	-0.24022657	0.21627	0.26667	0.78645	0.51473	1.20161
ebi-a-GCST004454	Abnormal spermatozoa	6	0.094244499	0.11309	0.40466	1.09883	0.88036	1.37151
ebi-a-GCST004455	Abnormal spermatozoa	9	-0.1190173	0.12156	0.32755	0.88779	0.69958	1.12665
ebi-a-GCST004456	Abnormal spermatozoa	9	0.141025364	0.27819	0.61219	1.15145	0.6675	1.9863
ebi-a-GCST004457	Abnormal spermatozoa	10	-0.01956732	0.07777	0.80135	0.98062	0.84198	1.1421
ebi-a-GCST004458	Abnormal spermatozoa	8	-0.351636878	0.193	0.06846	0.70354	0.48195	1.027
ebi-a-GCST004459	Abnormal spermatozoa	5	0.442512017	0.46478	0.34105	1.55661	0.62597	3.87089
ebi-a-GCST004460	Abnormal spermatozoa	16	-0.00222247	0.13619	0.98698	0.99778	0.76403	1.30305

Supplementary Table 10: Sensitivity analysis results of psoriasis on male reproductive system diseases.

Exposure	Outcome	Q_pval	MRegger_	MRegger_	MRPRESSO_GLOBAL
Psoriasis	Male infertility	0.848	0.017	0.603	0.833
	Abnormal spermatozoa	0.145	0.008	0.774	0.662
	Sexual dysfunction	0.016	0.074	0.427	NA
	Erectile dysfunction	0.621	-0.013	0.599	0.657
	Testicular dysfunction	0.992	0.065	0.198	0.994
Psoriasis vulgaris	Male infertility	0.619	0.068	0.165	0.442
	Abnormal spermatozoa	0.590	0.051	0.226	0.427
	Sexual dysfunction	0.049	-0.076	0.573	0.101
	Erectile dysfunction	0.931	-0.009	0.793	0.895
	Testicular dysfunction	0.766	-0.056	0.435	0.856
Arthropathic psoriasis	Male infertility	0.820	0.060	0.614	0.881
	Abnormal spermatozoa	0.477	0.068	0.525	0.590
	Sexual dysfunction	0.303	0.245	0.380	0.489
	Erectile dysfunction	0.941	0.040	0.662	0.884
	Testicular dysfunction	0.978	-0.045	0.801	0.978
Guttate psoriasis	Male infertility	0.853	-0.033	0.815	0.538
	Abnormal spermatozoa	0.759	0.065	0.624	0.403
	Sexual dysfunction	0.651	-0.189	0.553	0.234
	Erectile dysfunction	0.445	-0.108	0.424	0.563
	Testicular dysfunction	0.890	-0.026	0.904	0.851
Other and unspecified psoriasis	Male infertility	0.673	0.051	0.618	0.367
	Abnormal spermatozoa	0.790	0.042	0.630	0.395
	Sexual dysfunction	0.812	-0.030	0.876	0.268
	Erectile dysfunction	0.356	-0.032	0.751	0.552
	Testicular dysfunction	0.960	-0.029	0.843	0.663
Psoriasis (UKB)	Abnormal spermatozoa	0.515	-0.010	0.570	0.347
	Testicular dysfunction	0.342	-0.027	0.408	0.440

Supplementary Table 11: Sensitivity analysis results for MR analysis of male reproductive health on psoriasis.

Exposure	Outcome	Q_pval	MRegger_	MRegger_	MRPRESSO_GLOBAL
Male infertility	Psoriasis	0.517	-0.016	0.343	0.502
	Psoriasis vulgaris	0.885	-0.015	0.480	0.865
	Arthropathic psoriasis	0.553	0.002	0.920	0.550
	Guttate psoriasis	0.373	0.033	0.700	0.365
	Other and unspecified psoriasis	0.349	-0.017	0.678	0.335
Abnormal spermatozoa	Psoriasis	0.227	-0.033	0.225	0.276
	Psoriasis vulgaris	0.160	-0.034	0.342	0.212
	Arthropathic psoriasis	0.480	-0.017	0.659	0.402
	Guttate psoriasis	0.360	0.014	0.913	0.385
	Other and unspecified psoriasis	0.812	-0.073	0.201	0.841
Sexual dysfunction	Psoriasis	0.500	-0.030	0.474	0.526
	Psoriasis vulgaris	0.780	-0.024	0.637	0.799
	Arthropathic psoriasis	0.097	-0.065	0.528	0.188
	Guttate psoriasis	0.420	0.196	0.360	0.479
	Other and unspecified psoriasis	0.886	-0.010	0.913	0.904
Erectile dysfunction	Psoriasis	0.703	0.004	0.838	0.706
	Psoriasis vulgaris	0.603	0.023	0.404	0.612
	Arthropathic psoriasis	0.207	-0.004	0.931	0.227
	Guttate psoriasis	0.262	-0.048	0.691	0.257
	Other and unspecified psoriasis	0.932	0.043	0.400	0.938
	Psoriasis(UKB)	0.802	0.000	0.959	0.797
Testicular dysfunction	Psoriasis	0.571	-0.169	0.337	0.634
	Psoriasis vulgaris	0.274	-0.315	0.202	0.327
	Arthropathic psoriasis	0.656	-0.114	0.653	0.709
	Guttate psoriasis	0.793	0.439	0.570	0.821
	Other and unspecified psoriasis	0.905	-0.187	0.608	0.924

Supplementary Table 12: Sensitivity analysis results for analysis of serum metabolites on abnormal spermatozoa.

Exposure	Q_pval	MRegger_	MRegger_	MRPRESSO_GLOBAL
gamma-glutamylglutamine	0.7667	0.01639	0.49405	0.83
aspartate	0.26329	-0.13431	0.27177	0.424
glucose	0.80406	0.00331	0.87424	0.804
2-hydroxyisobutyrate	0.67981	0.01323	0.73224	0.633
X-09026	0.25194	-0.03815	0.58933	0.301
cysteine	0.67412	-0.01163	0.65851	0.706
dodecanedioate	0.94247	0.036	0.67741	0.913
butyrylcarnitine	0.93102	-0.0187	0.21063	0.939
caprylate (8:0)	0.77527	0.02467	0.09775	0.795
X-11315	0.92801	0.01001	0.58798	0.938
X-11483	0.97171	0.021	0.436	0.979
X-11786--methylcysteine	0.12357	-0.10768	0.19197	0.15
1,3,7-trimethylurate	0.36889	-0.06978	0.2927	0.423
X-12844	0.65236	-0.0215	0.654	0.661
2-linoleoylglycerophosphocholine	0.70309	0.01414	0.75358	0.748
X-14745	0.29549	0.17174	0.14148	0.298

Supplementary Table 13: Sensitivity analysis results for MR analysis of serum inflammatory factors on abnormal spermatozoa.

Exposure	Outcome	Q_pval	MRegger_	MRegger_	MRPRESSO_GLOBAL
Psoriasis vulgaris	X-09026	0.375	0.011	0.245	0.423
Arthropathic psoriasis	X-11786--methylcysteine	0.654	NA	NA	NA
Other and unspecified psoriasis	Aspartate	0.946	0.003	0.62	0.938
Other and unspecified psoriasis	Cysteine	0.862	-0.001	0.639	0.647
